# Broadly Accessible and Highly Active Hydrophosphination Catalysis With Simple Calcium Compounds in DMSO Solution

**DOI:** 10.1002/cssc.71145

**Published:** 2026-10-12

**Authors:** Moniruzzaman Moniruzzaman, Rory Waterman

**Affiliations:** ^1^ Department of Chemistry University of Vermont Burlington Vermont USA; ^2^ Gund Institute for Environment Burlington Vermont USA

**Keywords:** calcium, dielectric constant, hydrophosphination, phosphine, photocatalysis

## Abstract

Highly earth‐abundant and simple calcium precatalysts are reported that address the long‐standing challenge of broad scope in hydrophosphination catalysis. With more than 120 examples of hydrophosphination reactions, calcium catalysts are the broadest in scope, including traditional styrene substrates and activated alkenes (Michael acceptors), conjugated dienes, heterocumulenes, alkynes, unactivated alkenes, and even naturally occurring alkenes. Reactions are optimized to maximum conversion, and few examples have less than 90% conversion, making these the most active catalysts to date. Two features contribute to this exceptional scope and activity. The first feature is solvent. Reactions run in DMSO experience a step up in relative rate compared to those in lower‐dielectric solvents. Second, more challenging substrates are readily accessed under photocatalytic conditions, a feature already established for calcium precatalysts. The simple precatalyst, Ca(HMDS)_2_(THF)_2_ (HMDS  =  ^–^N(SiMe_3_)_2_, **1**), works for all reactions described, and mechanistic evidence points to a nucleophilic attack for amenable substrates. This work highlights critical advances in hydrophosphination (solvent effects, s‐block catalysts, and photocatalysis) to yield an abundant metal (calcium) in green solvent with low energy input to achieve hydrophosphination of the widest scope of substrates under operationally simple conditions.

## Graphical abstract

Simple calcium bis(amido) compounds are exceptionally potent hydrophosphination catalysts in DMSO solution. At ambient temperature or under irradiation, this catalyst successfully engages all manner of unsaturated substrates with primary and secondary phosphines with more than 100 examples of high conversion. These developments realize a promise of hydrophosphination, wide substrate access accessible and perfect atom economy, using the fifth most abundant element in the earth’s crust as a catalyst.

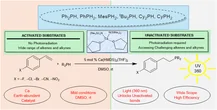

## 1 Introduction

The development of efficient, selective, and sustainable strategies for C─P bond formation has drawn considerable attention due to the indispensable roles of the organophosphorus compounds across human enterprise [1, 2, 3, 4, 5, 6, 7, 8, 9, 10, 11, 12, 13, 14, 15, 16, 17, 18]. Hydrophosphination remains an attractive synthetic method in such efforts due to perfect atom economy and the potential for versatile products [16, 19, 20, 21, 22, 23, 24, 25].

Despite these advantages, hydrophosphination suffers from several unresolved challenges that limit its broader utility [20, ‍26, 27]. At the core of all challenges for this catalysis is activity [2, 26], but a recent resurgence in s‐block catalysts for hydrophosphination has addressed aspects of this challenge [28, 34]. Most notably, reports of highly active alkali metal catalysts have exploded in the last few years, despite these being viable catalysts for decades [27]. Two key discoveries in this area inform the present study. First, reactivity increases with atomic number [35], for example, cesium‐based precatalysts have activity that dwarfs potassium‐based catalysts [28, 29], despite the latter being much longer known [36, 37, 38]. Second, charge separation, which can be enhanced with solvent effects or through additives to sequester the cation, promotes high activity [39, 40, 41, 42]. Charge separation is most prominent with alkali metal hydroxide catalysts in DMSO where NaOH can exhibit comparable activity as CsOH [43]. Group 2 catalysts are less well explored by comparison [16, ‍44, 45], but key findings stem back to reports from each the groups of Barrett and Hill [30, 31] and of Carpentier and Sarazin [33, 34, 46, 47, 48, 49, 50, 51, 52] in the early 2000s. Notably, a reinvestigation of calcium catalysts revealed that substantially improved activity could be observed under photocatalytic conditions [32], consistent with findings for d‐block metals [53, 54, 55, 56].

These factors have provided individual advances in hydrophosphination catalysts including the use of abundant s‐block metals, charge effects, solvent effects, and photocatalysis, each of which has provided a particular leap in activity. The convergence of these effects has not been explored, and it was our hypothesis that these effects could be combined for substantial gains in catalyst activity with the most abundant metals.

To that end, we have reinvestigated catalytic hydrophosphination using Ca(HMDS)_2_(THF)_2_ (HMDS = ^–^N(SiMe_3_)_2_, **1**). By transitioning to less hazardous DMSO [57, 58], Ca(HMDS)_2_(THF)_2_ provides the greatest activity known for activated alkenes, including cinnamates and acrylates as well as heterocumulenes. This activity even surpasses that of hydroxide salts in DMSO [43]. Closed‐shell photocatalysis remains available to Ca(HMDS)_2_(THF)_2_ in DMSO, and scores of unsaturated substrates including styrene derivatives, unactivated alkenes, internal and terminal alkynes, and naturally derived alkenes are viable under these conditions with some of the fastest times to complete conversion. The phosphine substrate scope can be expanded under photocatalytic conditions to include a range of primary and secondary phosphine substrates. With more than 100 examples of successful reactions (i.e., greater than 90% conversion), this commercially available, abundant catalyst addresses the most pointed, long‐standing challenge of hydrophosphination: activity for a broad range of substrates.

## 2 Results and Discussion

A comparison between solvents was undertaken with Ca(HMDS)_2_(THF)_2_ (**1**) to understand the impact of polarity on hydrophosphination catalysis because remarkable effects have been noted already [35, 43]. To make an effective comparison, standard conditions were used in which 0.5 mmol of unsaturated substrate and 0.5 mmol of aliphatic or aromatic phosphine were reacted in 0.4 mL of DMSO‐*d*_6_ with 5 mol% of **1** as precatalyst. As was reported before, photocatalytic conditions accelerate hydrophosphination, and reactions were irradiated with a lamp centered around 360 nm based on spectroscopic analysis of these reactions [53, 54, 59]. The initial focus was styrene, which has been a benchmark substrate in this reaction [26]. Recent solvent effect analyses have identified that there is greater catalytic activity in more polar solvents [35], and a greater set of solvents were not explored in this study for that reason. As a note, solvent effects beyond polarity, as approximated with dielectric constant, have been observed [19, ‍43, ‍60, ‍61]. Calcium catalysts for hydrophosphination have been proposed to proceed by both nucleophilic and insertion‐based mechanisms [30, 62], both of which would be anticipated to profit from increased solvent polarity. Indeed, comparison of the hydrophosphination of styrene with diphenylphosphine in various solvents gave a clear correlation of relative rate of conversion to dielectric constant (Figure 1). Because highly polar solvents were so relatively reactive, comparison was most effective by measuring approximate time to complete conversion as assessed by ^31^P{^1^H} NMR spectroscopy. The conversion times drop substantially with increasing dielectric constant. The best reaction times were measured with acetonitrile and DMSO. High reactivity from the former solvent was pleasantly surprising for this reaction, but the possibility of background reactions and improvement with DMSO led this study to proceed with the latter.

**FIGURE 1:**
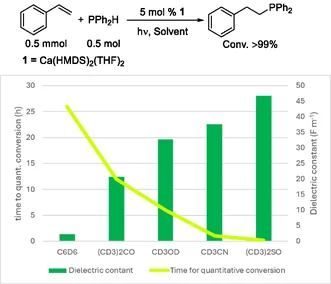
Solvent dielectric constant plotted against time to quantitative conversion for calcium‐catalyzed hydrophosphination of styrene.

The reactions in both acetonitrile and DMSO have similar approximate reaction times (Figure 1), but the DMSO reaction was run at ambient temperature without irradiation. That transformation is almost incomparably fast to originally reported benzene solution [32]. It is much faster than all other reported catalysts except group 1 bases that require, at most, a few hours for full conversion [29, ‍35]. Those significant enhancements in hydrophosphination [43] (and hydroarsination [63]) activity lean on DMSO solvent. It was therefore reasonable to anticipate greater reactivity for calcium to some extent, but part of the success of the hydroxide/DMSO reactions was the insoluble fraction of MOH [43]. However, the same kind of enhancement was observed here, which appears to be an unavoidable consequence of increased polarity. These recent studies demonstrate that styrene is no longer a good benchmark substrate, with catalysts now affording quantitative hydrophosphination in less time than an initial measurement by NMR spectroscopy can be made.

The direction of calcium catalysis studies has changed as a result of this observation. From a pragmatic standpoint, precatalyst **1** is simple and already used in catalysis [64, 65]. The high activity of **1** in proof‐of‐concept reactions in DMSO indicated that this system would provide transformative access to known and new hydrophosphination reactions, elevating the reaction in simplicity and efficiency with a highly earth‐abundant catalyst. This hypothesis has been borne out. The application of Ca(HMDS)_2_(THF)_2_ in DMSO solvent at ambient temperature with or without irradiation affords the widest substrate scope in the best reaction times reported.

### 2.1 Alkene Hydrophosphination With Diphenylphosphine

The starting point for analysis was steric and electronic variation on the styrene substrate, which gives the best opportunity to compare to reported catalysts [47, 66]. Like styrene itself, electron‐poor vinylarenes such as *para*‐fluoro‐ or *para*‐nitro‐styrene (**2a** and **8a**, Table 1) are completely consumed to product in less than an hour and require no direct irradiation. This behavior is anticipated based on prior study of calcium‐catalyzed hydrophosphination as well as related s‐block hydrophosphination catalysis [29, 32, 33]. In contrast, styrene derivatives featuring electron‐donating substituents were qualitatively slower, and full conversion in some instances required a reaction time of several hours under irradiation centered at 360 nm (Table 1, entries **10a**–**15a**). Under either photocatalytic or thermal conditions, this system exhibited broad functional group tolerance (Table 1), which is noteworthy given the relative basicity of Ca(HMDS)_2_(THF)_2_. More encumbered substrates such as α‐methyl styrene and 1,1‐diphenyl ethene required no irradiation for quantitative conversion under standard reaction conditions (Table 1, entries **19a** and **22a**). Yet, more encumbered *β*‐methyl styrene was demonstrably slower, requiring 3 h under irradiation for nearly quantitative conversion (Table 1, entry **20a**). The most challenging substrates on this list, *trans*‐stilbene and *β*‐bromostryene, required more than 24 h reaction time under irradiation (Table 1, entries **21a** and **23a**). These, and stilbene in particular, are examples of vexing alkene substrates [67], which profit from photocatalytic conditions, though there are still gains to be made.

**TABLE 1:** Catalytic hydrophosphination of vinyl arenes substrates with Ph_2_PH in DMSO‐*d*_6_ using 5 mol% of **1**.

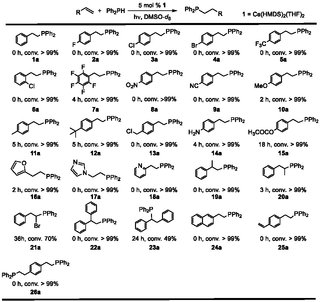

*Note*: Conversion determined by integration of ^31^P{^1^H} and ^1^H NMR spectra. For entry **26a**, 2 equiv. of Ph_2_PH was used.

Value‐added products can be prepared by this methodology. The P, N‐bidentate ligand, PyPhos (Table 1, entry **18a**), was prepared at ambient conditions in quantitative conversion from 2‐vinyl pyridine. In prior reports, 2‐vinyl pyridine undergoes rapid polymerization under similar conditions in benzene solution [30, 32], which represents improved chemical selectivity as a result of transiting to a greener solvent.

The 26 vinyl arene substrates shown in Table 1 represent the broadest substrate scope of any hydrophosphination analysis, tackling steric effects, electronic effects, and functional group tolerance. Nearly, all reactions afforded quantitative conversion in a matter of a few hours or less. These observations encouraged a robust investigation into the full extent of Ca(HMDS)_2_(THF)_2_ as a hydrophosphination precatalyst in DMSO solution.

Beyond vinyl arenes, heterocumulenes such as carbodiimides, as well as thiocyanates and imines, also exhibited immediate P–H addition (Table 2, entries **1b**–**6b**). Activated alkenes such as α, *β*‐unsaturated ketones and nitriles underwent instantaneous hydrophosphination under identical conditions (Table 2, entries **7b**–**11b**). The performance of unactivated alkenes was highly varied (Table 2, entries **13b**–**23b**). The best of these substrates was vinyltriethoxysilane (Table 2, entry **13b**), which was a positive surprise given that a trialkoxysilane or *tert*‐butyl substituent is not activating in a general sense, and those substrates afforded virtually no conversion (Table 2, entries **14b** and **15b**). Despite lengthy reaction times under irradiation, halogen, alcohol, and amine functional groups are tolerated with conversions better than those of terminal, linear alkenes (Table 2, entries **16b**–**23b**). For these substrates, rare instances of thermal reactions are also lengthy, and other photocatalysts have similar reaction times. They remain a challenge. Returning to more activated substrates, conjugated dienes are smoothly converted without irradiation (Table 2, entries **31b**–**33b**).

**TABLE 2:** Hydrophosphination of heterocumulenes, carbodiimides, α,*β*‐unsaturated ketones, unactivated alkenes, and dienes with Ph_2_PH in DMSO‐*d*_6_ using 5 mol% of **1**.

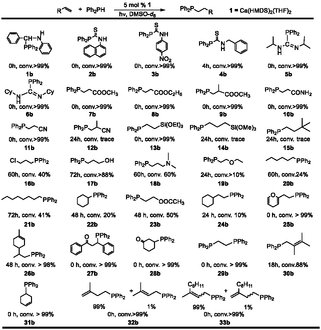

*Note*: Conversion determined by integration of ^31^P{^1^H} and ^1^H NMR spectra.

These examples include a pair of established ligands, Prophos and 1,2‐bis(diphenylphosphino)ethane (dppe) (Table 2, entry **17b** and **29b**), which demonstrate the potential for further use in the preparation of value‐added products.

### 2.2 Alkyne Hydrophosphination With Diphenylphosphine

In a previous report of calcium‐catalyzed hydrophosphination, terminal alkynes exhibited modest conversion in benzene solution, even under irradiation [32]. A striking enhancement in reactivity has been observed in DMSO solution for these substrates as well. All alkyne substrates screened, including terminal, internal, and those with withdrawing or donating substituents, were all completely converted within a period of hours at ambient temperature (Table 3). As with styrene substrates (vide supra), alkynes possessing electron‐withdrawing substituents or otherwise activated were converted in less than an hour without irradiation. Unactivated or sterically encumbered alkynes were converted in a period of several hours under irradiation, with the only exception being cyclohexylacetylene, which was completely consumed to **14c** over 24 h, but linear aliphatic alkynes were converted in 4 h or less (**15c** and **16c**).

**TABLE 3:** Hydrophosphination of alkynes with Ph_2_PH in DMSO‐*d*_6_ using 5 mol% of **1**.

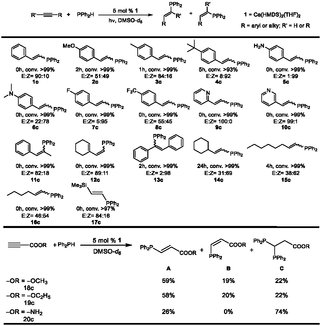

*Note*: Conversion determined by integration of ^31^P{^1^H} and ^1^H NMR spectra. Entries 18c, 19c, 20c have 22%, 22%, and 74% bis‐diphenyl phosphine products respectively.

Reactions had some preference for isomers, but *E*:*Z* ratios could not generally be characterized as selective. Indeed, the more selective reactions appear to be more fortuitous and substrate‐dependent. Reduced ratio values were observed for more electron‐rich substrates, which may reflect reduced selectivity over the course of longer reaction times than the electronics of substrate. These observations support the hypothesis that genuine selectivity could be achieved with judicious ligand choice. Ligand effects in photocatalytic reactions have recently been highlighted. In Steffen and coworkers’ recent exploration of photocatalytic hydrophosphination of alkynes by copper compounds, the *E*:*Z* ratios were variable, but ligand choice allowed for visible excitation [68]. More germane, several features were identified in that study that may lead to genuine regioselectivity [68]. Such factors may be extended to photocatalytic reactions with calcium as well.

### 2.3 Dialkylphosphine Substrates

Diphenylphosphine is heavily used in hydrophosphination studies, and calls have been made for other substrates [20]. Two challenging dialkyl phosphines, dicyclohexyl and di‐*tert*‐butylphosphines, have been explored here. These are particularly challenging substrates due to their steric demand. For dicyclohexylphosphine, eight unsaturated substrates were screened, and the trends for diphenyl phosphines were preserved in which electron‐poor alkenes reacted faster, and electron‐rich alkenes were slower to react. With some exceptions, reactions were still relatively prompt in those five examples and proceeded to quantitative conversion within an hour (Table 4, entries **1o**–**3o**, **5o**, and **8o**). Only three examples required irradiation and longer reaction times. For *p*‐methoxystyrene, this resulted in moderate conversion (Table 4, entry **4o**). Surprisingly, a pair of acrylate substrates required extended reaction time under irradiation to poor conversions as mixtures of the Markovnikov and anti‐Markovnikov products (Table 4, entries **6o** and **7o**). This would suggest that regioselectivity can be manipulated with steric factors, despite the modest bias for the anti‐Markovnikov product (vide infra), which is an idea impacting future calcium variants. As above, the reaction can be used for value‐added products, and the unsymmetrical diphosphinoethane **8o** is readily prepared under these conditions.

**TABLE 4:** Hydrophosphination of vinyl arene, Michael acceptors, vinyl diphenylphosphine with dicyclohexylphosphine in DMSO‐*d*_6_ using 5 mol% of **1**.

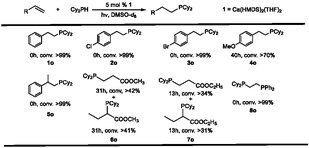

*Note*: Conversion determined by integration of ^31^P{^1^H} and ^1^H NMR spectra. No irradiation was required for entries **1o**–**3o**, **5o**, **8o.** Both Markovnikov and anti‐Markovnikov products were observed for **6o** and **7o**.

Di‐*tert*‐butylphosphine was also a viable substrate under standard catalytic conditions. For example, the hydrophosphination of styrene with equimolar di‐*tert*‐butylphosphine proceeded smoothly without irradiation (Table 5, entry **1p**). Of the five unsaturated substrates screened, most proceeded within 8 h or less to high conversions (Table 5). Irradiation and extended reaction time are needed for modest conversions with α‐methylstyrene (Table 5, entry **2p**), which highlights the increased steric demand of this substrate given the immediate conversion with dicyclohexylphosphine (Table 4, entry **5o**). A limit here was the rapid consumption of 2‐vinylpyrydine to apparent polymerization, resulting in poor hydrophosphination (Table 5, entry **4p**). Again, a value‐added diphosphinoethane, unsymmetrical **5p**, is readily prepared by irradiation under these conditions (Table 5).

**TABLE 5:** Hydrophosphination of vinyl arene substrates with di‐*tert*‐butylphosphine in DMSO‐*d*_6_ solution using 5 mol% of **1**.

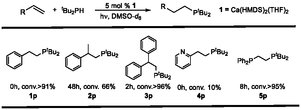

*Note*: Conversion determined by integration of ^31^P{^1^H} and ^1^H NMR spectra. No irradiation was required for entry **1p**. Vinyl pyridine (**4p**) was rapidly polymerized under reaction conditions. Some impurities were measured in these reactions by ^31^P{^1^H} and ^1^H NMR spectroscopy.

### 2.4 Primary Phosphines

Continuing the exploration of substrate scope, primary phosphine substrates were next screened, and excellent activity was observed with them. Reactions with primary phosphines were conducted with excess phosphine (up to 2 equiv.) to inhibit competitive double P─H bond activation and afford the secondary phosphine product. Double P─H bond activation is most problematic for phenylphosphine, and many reactions have trace quantities of the tertiary phosphine as a byproduct [54, 69, 70]. For example, hydrophosphination of *para*‐chloro styrene with 2 equiv. of phenylphosphine under standard reaction conditions generates approximately 2% of di‐(*p*‐chlorophenethyl) phenylphosphine along with the expected *para*‐chlorophenethyl phenylphosphine product (Table 6, entry **3d**).

**TABLE 6:** Catalytic hydrophosphination of vinyl arene substrates, Michael acceptors, dienes, and unactivated alkene with phenylphosphine using 5 mol% of **1** in DMSO‐*d*_6_.

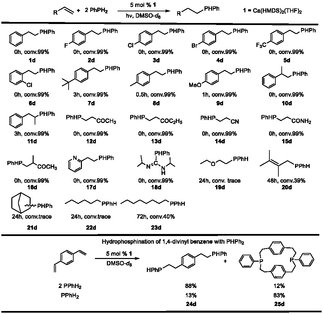

*Note*: Conversion determined by integration of ^31^P{^1^H} and ^1^H NMR spectra. All reactions have trace quantities of the double P─H bond activation product.

A range of vinyl arene substrates were evaluated. Treatment of alkyne with phenylphosphine (2 equiv.) in DMSO‐*d*_6_ with 5 mol% of **1** resulted in high conversions. As with diphenylphosphine, substrates bearing electron‐withdrawing substituents were converted without irradiation within the time required for NMR spectra to be collected (Table 6, entries **1d**–**6d**), whereas electron‐rich counterparts required irradiation but still achieved quantitative conversion within a period of several hours (Table 6, entries **7d**–**9d**). In all cases, some double P─H bond activation products were measured despite excess phosphine. Activated substrates proceeded rapidly without irradiation as well (Table 6, entries **12d**–**16d**). The reaction was slowed, but only slightly, by more encumbered substrates like *β*‐methylstyrene (Table 6, entry **11d**), and α‐methylstyrene was easily converted (Table 6, entry **10d**). Other varied but relatively activated substrates were easily converted, including a carbodiimide, a conjugated diene, and strained secondary alkene (Table 6, entries **18d**, **20d**, and **21d**). Unactivated alkenes generally necessitate prolonged irradiation, though notable exceptions such as ethoxyethene and 1‐hexene display very limited reactivity even under extended hours of irradiation (Table 6, entries **19d** and **22d**). Despite these limitations, phenylphosphine exhibited broad functional group tolerance and generally high activity with calcium.

Complex **1** was further employed for the hydrophosphination of 1,4‐divinylbenzene with phenylphosphine. Reaction with 1 equiv. of phenylphosphine afforded the macrocyclic product, 4,10‐diphenyl‐4,10‐diphospha‐1,7(1,4)‐dibenzene‐cyclododecaphane, in 83% conversion, a structurally attractive ligand scaffold for coordination chemistry (Table 6, entry **25d**). In contrast, treatment with 2 equiv. of phenylphosphine predominantly afforded the double hydrophosphination product of 1,4‐divinylbenzene in 88% conversion. Hydrophosphination of 2‐vinyl pyridine was also smooth (**17d**), demonstrating the reaction to be viable for the synthesis of ligands or ligand precursors.

Following the results obtained with alkenes, phenylphosphine was also screened with terminal and internal alkynes, delivering high conversions for aryl alkynes (Table 7, entries **1k**–**4k**), where only 1‐phenylpropyne required irradiation for an extended period (Table 7, entry **2k**). Unlike the reactions with diphenylphosphine, E:Z ratios were closer to equal, reinforcing that the observed selectivity is more related to substrate effects than catalyst. Internal aliphatic alkynes were ineffective substrates under these conditions (Table 7, entries **5k** and **6k**).

**TABLE 7:** Hydrophosphination of alkynes with phenylphosphine in DMSO‐*d*_6_ using 5 mol% of **1**.

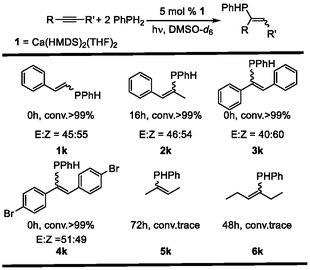

*Note*: Conversion determined by integration of ^31^P{^1^H} and ^1^H NMR spectra. All reactions have trace quantities of the double P─H bond activation product.

Primary alkyl phosphines, such as cyclohexylphosphine, have seen limited use in catalytic hydrophosphination compared to phenylphosphine [16, 20]. Precatalyst **1** efficiently executed the hydrophosphination of styrene, *para*‐chloro‐ and *para*‐cyanostyrene, acrylamide, and diisopropylcarbodiimide, delivering nearly quantitative conversions in all cases. (Table 8).

**TABLE 8:** Hydrophosphination of vinyl arene, carbodiimide, and Michael acceptor substrates with cyclohexylphosphine in DMSO‐*d*_6_ using 5 mol% of **1**.

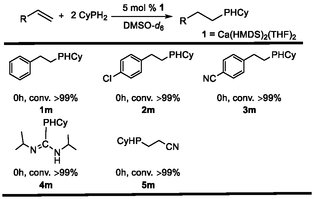

*Note*: Conversion determined by integration of ^31^P{^1^H} and ^1^H NMR spectra. All reactions have trace quantities of the double P─H bond activation product.

Another primary phosphine, mesitylphosphine, demonstrated good reactivity under calcium‐catalyzed conditions. Styrene, α‐methylstyrene, and phenylacetylene were all rapidly and near quantitatively converted to product without irradiation, though some double P─H bond activation product was observed (Table 9, entries **1n**–**3n**). Diphenylacetylene was a more stubborn substrate, but near‐quantitative conversion of this substrate was achieved under irradiation at 360 nm over 48 h (Table 9, entry **4n**). Like cyclohexylphosphine, greater steric encumbrance at the phosphine substrate can be tolerated with calcium in DMSO to good overall effect.

**TABLE 9:** Hydrophosphination of unsaturated compounds with mesitylphosphine in DMSO‐*d*_6_ using 5 mol% of **1**.

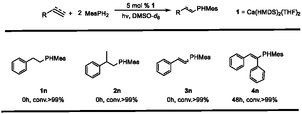

*Note*: Conversion determined by integration of ^31^P{^1^H} and ^1^H NMR spectra. All reactions have trace quantities of the double P─H bond activation product. No irradiation was required for entries **1n**–**3n**.

Catalytic hydrophosphination reactivity enhances down the group of the alkali metals, with increasing atomic size [28, ‍29]. Based on this trend, heavier group 2 metals were hypothesized to accelerate reactions that proceed more slowly with Ca in DMSO under irradiation. To investigate this possibility, 5 mol% Sr(HMDS)_2_ (HMDS = ^–^N(SiMe_3_)_2_) was employed in place of Ca(HMDS)_2_(THF)_2_ under the standard reaction conditions for several vinyl arenes bearing electron‐donating substituents on the phenyl ring along with *β*‐methyl styrene with Ph_2_PH. These substrates showed slower reactivity with catalyst **1**, requiring 2 h or more to reach quantitative conversion under irradiation. In contrast, all of the substrates exhibited faster reactivity with Sr(HMDS)_2_ (Table 10). Overall, these results demonstrate that group 2 metals exhibit a similar trend to the alkali metals, with increasing reactivity down the group.

**TABLE 10:** Hydrophosphination of alkenes with Ph_2_PH in DMSO‐*d*_6_ using 5 mol% Sr(HMDS)_2_.

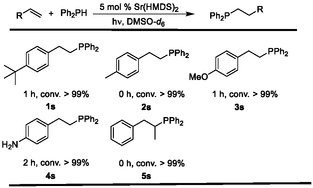

*Note*: Conversion was determined by integration of ^31^P{^1^H} and ^1^H NMR spectra.

### 2.5 Mechanistic Studies

A series of experiments reveal key features of this catalysis. First, the possibility of induced radical hydrophosphination in DMSO was considered due to the prominence of this reactivity [71, ‍72]. Reaction mixtures under irradiation provided no detectable radicals by EPR spectroscopy as well as spectra of permutations of catalysts and substrates, in contrast to prior examples of photo‐induced radical hydrophosphination with metal catalysts [32, 59, 73]. Some common radical traps are generally fraught for phosphine substrates [74], and this trend continues for calcium compounds and phenolic traps like BHT and hydroquinone. In catalytic runs with excess of either trap, the reactions cease. The inhibition appears to be selective reaction with the phenol because stoichiometric reaction of hydroquinone and Ca(HMDS)_2_(THF)_2_ reveals a direct interaction between hydroquinone and calcium based on a shift of the OH resonance in the ^1^H NMR spectrum (see Supporting Information).

Other radical traps support closed‐shell reactivity. For example, the hydrophosphination of styrene with diphenylphosphine is unaffected by excess cyclohexadiene. In reactions where (chloromethyl)cyclopropane has been added, hydrophosphination occurs, but a competitive nucleophilic substitution occurs on the additive (Scheme 1). Because that reaction is qualitatively faster than hydrophosphination, little conversion occurs until the relative concentration of chloromethylcyclopropane has diminished. Importantly, no ring‐opening is observed in these reactions.

**SCHEME 1:**
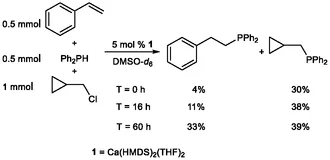
Hydrophosphination of styrene in the presence of (chloromethyl)cyclopropane under standard conditions.

The most telling reaction for a closed‐shell process was the hydrophosphination of α‐cyclopropyl styrene. Treatment of α‐cyclopropyl styrene with 1 equiv. of diphenylphosphine and 5 mol% of **1** in DMSO‐*d*_6_ resulted in complete conversion without irradiation. (Scheme 2). In contrast, a prior reaction with this substrate and a metal‐based radical initiator resulted in cyclopropane ring opening [59].

**SCHEME 2:**
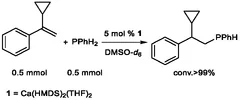
Successful hydrophosphination in the presence of a radical trap, α‐cyclopropyl styrene, under standard conditions.

Many hydrophosphination catalysts appear to undergo one of two canonical mechanisms, nucleophilic or insertion‐based (Figure ‍2) [2, 16]. The former is typified by conjugated or electron‐deficient substrates attacked by a nucleophilic metal‐phosphide intermediate [1]. Insertion‐based mechanism are the traditional organometallic reactivity of a migratory insertion of unsaturated substrate. Nearly all hydrophosphination catalysts afford anti‐Markovnikov selectivity [16], which can be rationalized in the generic mechanisms but makes the distinction between mechanisms more challenging.

**FIGURE 2:**
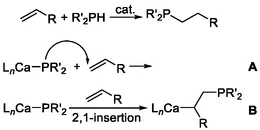
Illustration of nucleophilic (A) and insertion‐based (B) mechanisms for hydrophosphination with a generic alkene substrate.

The qualitative observation of nucleophilic character can be affirmed in a Hammett analysis that affords a ρ value of 0.21 (see the Supporting Information). Such results are consistent with the proposed nucleophilic mechanism for calcium hydrophosphination catalysts computed by Hunt and coworkers [62]. That assertion can be explicitly assessed by adapting the Glueck test for nucleophilic hydrophosphination [75]. That reaction traps a potential Zwitterionic intermediate with a potent electrophile, consistent with a nucleophilic mechanism [75]. In this example, methyl acrylate was the selected unsaturated substrate and 5 equiv. of benzaldehyde was the trapping agent. As anticipated from the Hammett analysis, a Zwitterionic intermediate is trapped as about 85% of the measured product, with the balance as hydrophosphination product (Scheme 3). The nucleophilic character in this catalysis is thus confirmed.

**SCHEME 3:**
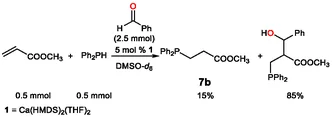
Experiment to trap a Zwitterionic intermediate using 5 equiv. of benzaldehyde.

However, the reactivity with unactivated alkenes cannot be explained by nucleophilic attack. Furthermore, nucleophilicity of phosphorus would be diminished under photochemical conditions due to the lowest anticipated transition being a ligand‐to‐metal charge transfer (LMCT) for a R_2_P–CaL_*n*_ intermediate. These observations suggest insertion‐based hydrophosphination, consistent with photocatalytic hydrophosphination reactions of copper and zirconium [54, 56], for unactivated substrates. This aspect of the mechanism is subject to ongoing study because greater understanding here is likely to inform achieving greater activity necessary for practical conversion of unactivated alkenes and many bio‐generated alkenes (e.g., derivatives of isoprene).

Deuterium‐labeling reactions were undertaken as well. In reactions with diphenylphosphine‐*d*_1_ (80% D incorporation), exclusive *β* addition of deuterium was measured in the hydrophosphination reaction with styrene, as assessed by a suite of NMR spectroscopic assessments (Scheme 4). This observation is inconsistent with both mechanistic hypotheses, but prior reactions with s‐block catalysts have also yielded the same selectivity, a factor attributed to kinetic trapping after a sigmatropic rearrangement.

**SCHEME 4:**
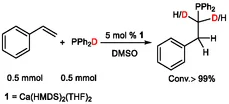
Deuterium labeling experiment using diphenylphosphine‐*d*_1_.

As an initial probe of photophysics, the UV–visible absorbance spectra of the equimolar (0.5 mmol) mixture of styrene, Ph_2_PH, and 5 mol% of **1** in DMSO were recorded. The spectrum revealed absorptions at 347, 371, and 475 nm. Despite this broad absorption profile, irradiation at 360 nm was demonstrated to be important for these reactions and selected due to the relatively high *λ*_max_ compared to that of other absorbances. There is strong absorbance at higher energy, but these transitions were avoided due to the potential for unintended photochemistry. The observed relative rate of the hydrophosphination of styrene (ca. 10 min) indicated that background photolytic hydrophosphination would not be competitive [19]. As was reported before [32], these simple calcium bisamide compounds have limited absorbance in the near UV, and the photoactive intermediate is a more complex species involving coordination of some if, not all reagents. Further study of these intermediates is underway.

Recently, Steffen has shown that the absorption of copper photocatalysis for hydrophosphination can be tuned with ligand effects [68]. Similar behavior would be important to future work with calcium, and a ligand‐supported derivative was tested to confirm that such behavior is possible. Initial runs with IPrCa[N(SiMe_3_)_2_]_2_ (IPr = 1,3‐bis(2,6‐diisopropylphenyl)imidazol‐2‐ylidene) give conversions akin to those of **1**, including under photochemical conditions.

For example, hydrophosphination of vinyl naphthalene and 2‐vinyl pyridine with Ph_2_PH in DMSO‐*d*_6_ using 2 mol% of IPrCa[N(SiMe_3_)_2_]_2_ resulted in quantitative conversion without irradiation at ambient temperature, while 4‐methyl styrene took 10 h photoirradiation under UV 360 nm for similar result (Equation (1)). It is clear that ligated calcium compounds are viable, and ligand effects will be the subject of future photochemical studies.



(1)

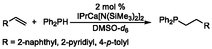




## 3 Conclusion

The decisive influence of DMSO underscores the importance of solvent‐mediated ion‐pair separation and transition‐state stabilization in main group catalysis. Photochemical activation at 360 nm UV light serves as a further kinetic enhancer that enables otherwise unreactive substrates to engage in the same fundamental pathway rather than introducing a distinct radical process. More importantly, the observation that photocatalysis is accelerated in polar solvent enhances the value of this increasingly general transformation. A detailed mechanistic investigation establishes that Ca(HMDS)_2_(THF)_2_‐catalyzed hydrophosphination in DMSO proceeds predominantly through a polar nucleophilic pathway involving phosphorus‐centered anionic intermediates. Benzaldehyde trapping, deuterium‐labeling studies, consistent 1,1‐regioselectivity, modest Hammett sensitivity, radical‐clock experiments, and EPR spectroscopy collectively exclude propagating radical mechanisms and instead support primarily nucleophilic addition followed by proton transfer as the dominant reaction manifold, though more organometallic‐like chemistry appears likely for photocatalytic reactions. Together, these findings provide a unified mechanistic framework that explains the extraordinary breadth of substrate scope observed across phosphines, alkenes, and alkynes.

## Funding

This work was supported by Division of Chemistry (CHE‐2348198).

## Conflicts of Interest

The authors declare no conflicts of interest.

## Supporting information

Experimental procedures and full spectroscopic data are given. Original data from the study is archived at www.uvm.edu/~waterman.

## Data Availability

The data that support the findings of this study are available from the corresponding author upon reasonable request.
