# Supplementary material for: Broadly Accessible and Highly Active Hydrophosphination Catalysis With Simple Calcium Compounds in DMSO Solution
Source: ChemSusChem. 2026 Oct 12;19(19):e71145. doi: 10.1002/cssc.71145 (PMC13656183; doi:10.1002/cssc.71145)
Supplement: Supplementary file 1 [file CSSC-19-e71145-s001.pdf]

Supporting information

for

Broadly Accessible and Highly Active Hydrophosphination Catalysis with Simple Calcium Compounds in DMSO Solution

Moniruzzaman Moniruzzaman,[a] and Rory Waterman[a][b]*

[a]Department of Chemistry, University of Vermont, Burlington, VT 05405, USA
[b]Gund Institute for Environment, Burlington, VT 05405, USA
*E-mail: rory.waterman@uvm.edu

Contents

Experimental section 3
General Considerations: 3
General procedure for hydrophosphination reactions: 3
General procedure for EPR measurements: 4
Control reaction NMR for hydrophosphination 5
Hydrophosphination of styrene with Ph_2PH in DMSO-d_6 (No catalyst) 5
Hydrophosphination of styrene with Ph_2PH in Acetone-d_6 7
Hydrophosphination of styrene with Ph_2PH in Methanol-d_4 13
Hydrophosphination of unsaturated compounds with Ph_2PH in DMSO using 5 mol% of **1** 17
Hydrophosphination of unsaturated compounds with phenyl phosphine using 5 mol% of **1** 173
Hydrophosphination of unsaturated compounds with cyclohexyl phosphine using 5 mol% **1** 235
Hydrophosphination of unsaturated compounds with dicyclohexyl phosphine using 5 mol% **1** 247
Hydrophosphination of unsaturated compounds with mesityl phosphine using 5 mol% **1** 260
Hydrophosphination of unsaturated compounds with ditertiary butyl phosphine using 5 mol% **1** 267
NMR spectra for the zwitter ionic intermediate reactions 275
Radical trapping experiments 280
NMR Spectra for chloromethyl cyclopropane as radical trapping reagent 280
NMR and HRMS spectra for radical trap reaction using α-cyclopropyl styrene 286
NMR spectra for radical trap experiment using BHT as radical scavenger 289
NMR spectra for radical trap experiment using hydroquinone as radical scavenger 291
NMR spectra for radical trap experiment using 1,4-cyclohexadiene as radical scavenger 294
NMR spectra for radical trap experiment using diphenyl amine as radical scavenger 296
NMR spectra for Hammett plot and Hammett Plot 298
NMR spectra for deuterium labelling reactions 303
EPR spectra for only catalyst and hydrophosphination reaction in DMSO at ambient conditions 308
UV-visible spectra for hydrophosphination reaction in DMSO 309
Hydrophosphination of vinyl arenes with Ph_2PH in DMSO-d_6 using 5 mol% NHCCa$(HMDS)_2$......310
References: 3106

Experimental section

General Considerations:

Air and moisture-sensitive reactions were conducted under a purified N_2 atmosphere using standard Schlenk line and glovebox techniques.

Ph_2PH was synthesized according to a modified literature procedure. [1] Phenyl Phosphine was used as received. $Ca(HMDS)_2(THF)_2$ (**1**), was synthesized following a modified literature method, and recrystallized multiple times from the same solvent. [2][3] DMSO-d_6, Acetone-d_6, methanol-d_4, benzene-d_6 were purified using literature procedure, underwent three freeze-pump-thaw cycles and were stored over activated 3 Å molecular sieves. Styrene and its derivatives were purified through a basic alumina column, subjected to three freeze-pump-thaw cycles, and further purified through activated alumina. Ethyl acrylate, methyl acrylate, acrylamide, acrylonitrile, methacrylonitrile, methylmethacrylate, all other alkenes, terminal and intermediate alkynes, 1,3-cyclohexadiene, limonene, isoprene, distilled over CaH_2 or sodium. All alkenes, alkynes and other substrates were stored at -40 °C after purification. NMR data were collected at 298 K on a Bruker AXR 500 MHz spectrometer, with spectra referenced to 1,3,5-trimethoxybenzene (δ = 3.73 ppm, in DMSO-d_6) for ^1H NMR or Ph_2PH (δ = -41 ppm) and $PhPH_2$ (δ = -123 ppm) for $^{31}P\{^1H\}$ NMR. EPR measurements were obtained using a Bruker EMXPlus Spectrometer. Measurements were taken at ambient temperature, and the parameters for the EPR measurements, which varied depending on the sample, are detailed in the Supporting Information.

The UV 360 nm bulb photoreactor used for photoirradiation is a Rexim G23 UV-A lamp, operating power of 9 W. All photocatalytic and non-photocatalytic reactions were run at ambient temperature with minimal additional heat from the lamps, as measured. NMR conversions were determined by integration of product signals relative to an internal standard of 1,3,5-trimethoxy benzene (TMB). UV visible spectra samples were prepared in a glovebox with 2 mm cuvettes with air-tight screw caps and spectra were recorded from a CARY100 Bio UV-Visible spectrophotometer.

General procedure for hydrophosphination reactions:

A scintillation vial was charged with 0.5 mmol of diphenyl phosphine or phenyl phosphine or other phosphines, followed by 0.5 mmol of alkene/alkynes and 0.11 mmol of 1,3,5-trimethoxybenzene. Subsequently, 0.4 ml of DMSO-d_6 was added to the vial, and 5 mol% of the catalyst **1** was introduced. The contents were mixed and then transferred to a PTFE-valved J-Young type NMR tube. The solution was irradiated with a 9 W UV 360 nm bulb for photocatalytic reactions. The reactions were monitored using $^{31}P\{^1H\}$ NMR and ^1H NMR spectroscopy at various time intervals. The photoreactor was maintained at a temperature of 27 °C. Control reactions conducted at ambient conditions at 22 °C showed minimal conversion to hydrophosphinated products. Both a photo of the setup used and an emission spectra for the 360 nm-centered lamp have been previously published.[4]

General procedure for EPR measurements:

A 0.5 mL aliquot of a 0.02 M solution of each catalyst in DMSO was transferred to a quartz EPR tube. EPR data were collected for each catalyst solution, as well as for solutions of styrene, diphenyl phosphine, and catalyst **1**. Hydrophosphination reactions were monitored via EPR without irradiation.

Key for the spectra:

*	Reactant peak
**	Product peak
***	Product peak (Double activation)

Control reaction NMR for hydrophosphination

Hydrophosphination of styrene with Ph_2PH in DMSO-d_6 (No catalyst)

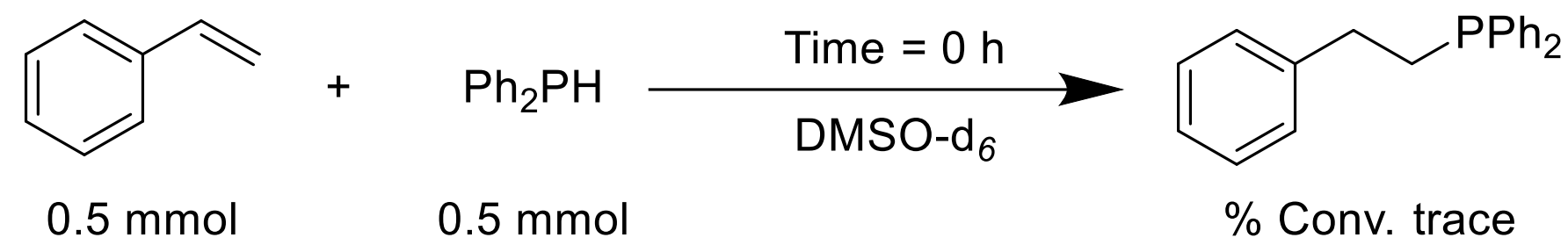

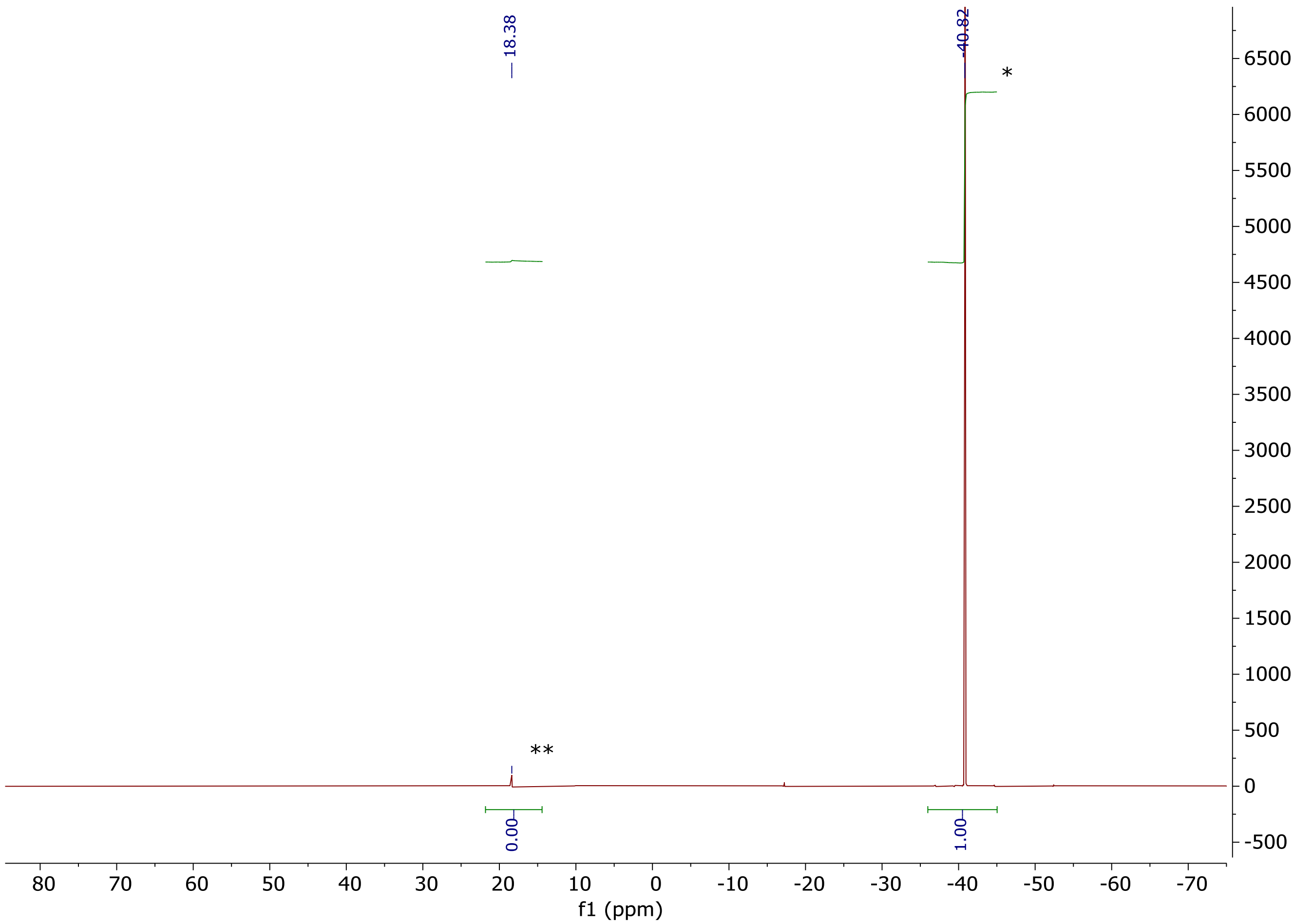

Figure S1: Hydrophosphination of styrene with Ph_2PH in DMSO-d_6 under ambient conditions– $^{31}P\{^1H\}$ NMR - 0 h (No catalyst)

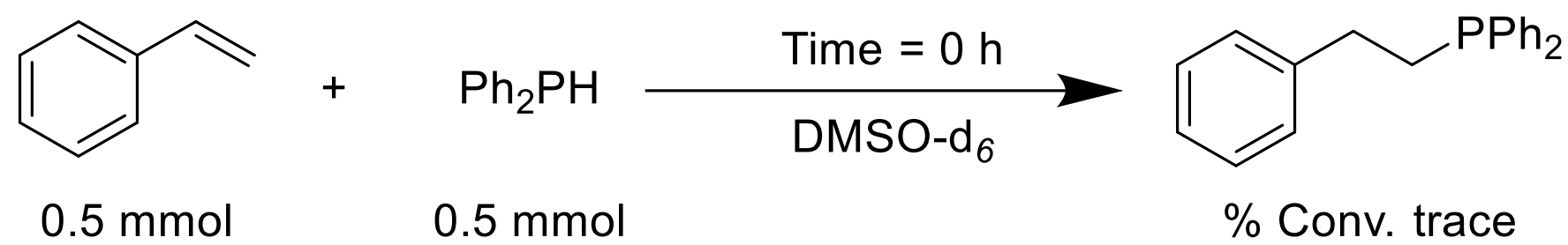

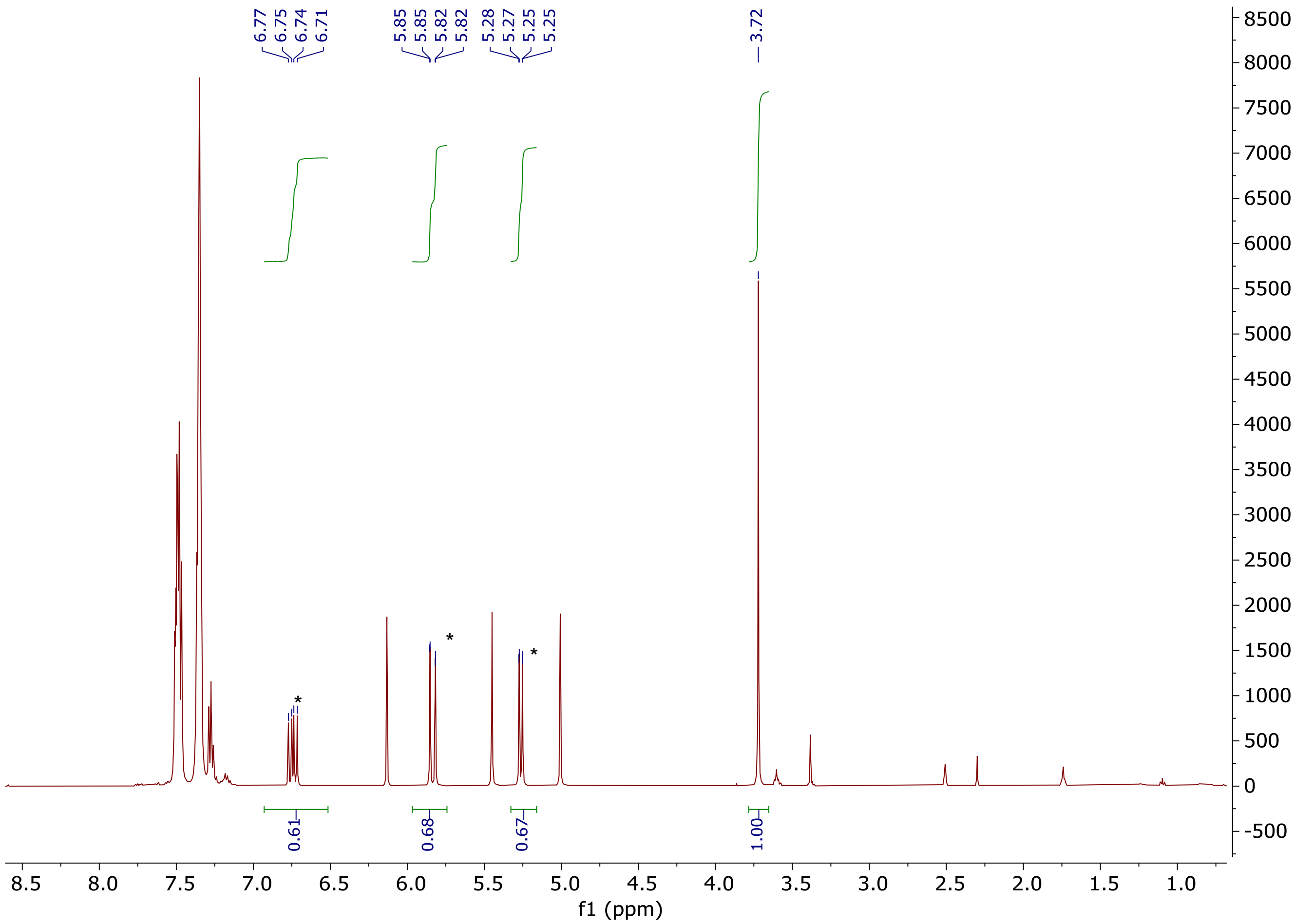

Figure S2: Hydrophosphination of styrene with Ph_2PH in DMSO-d_6 under ambient conditions - 0 h - ^1H NMR

Hydrophosphination of styrene with Ph_2PH in Benzene-d_6

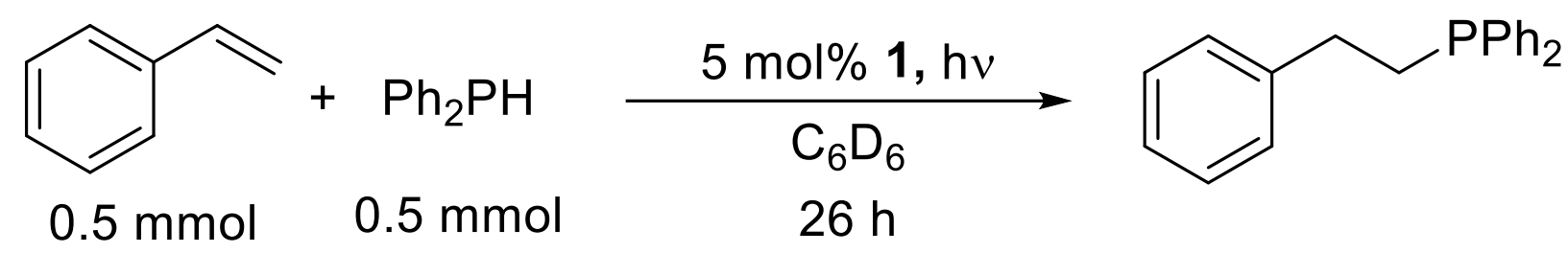

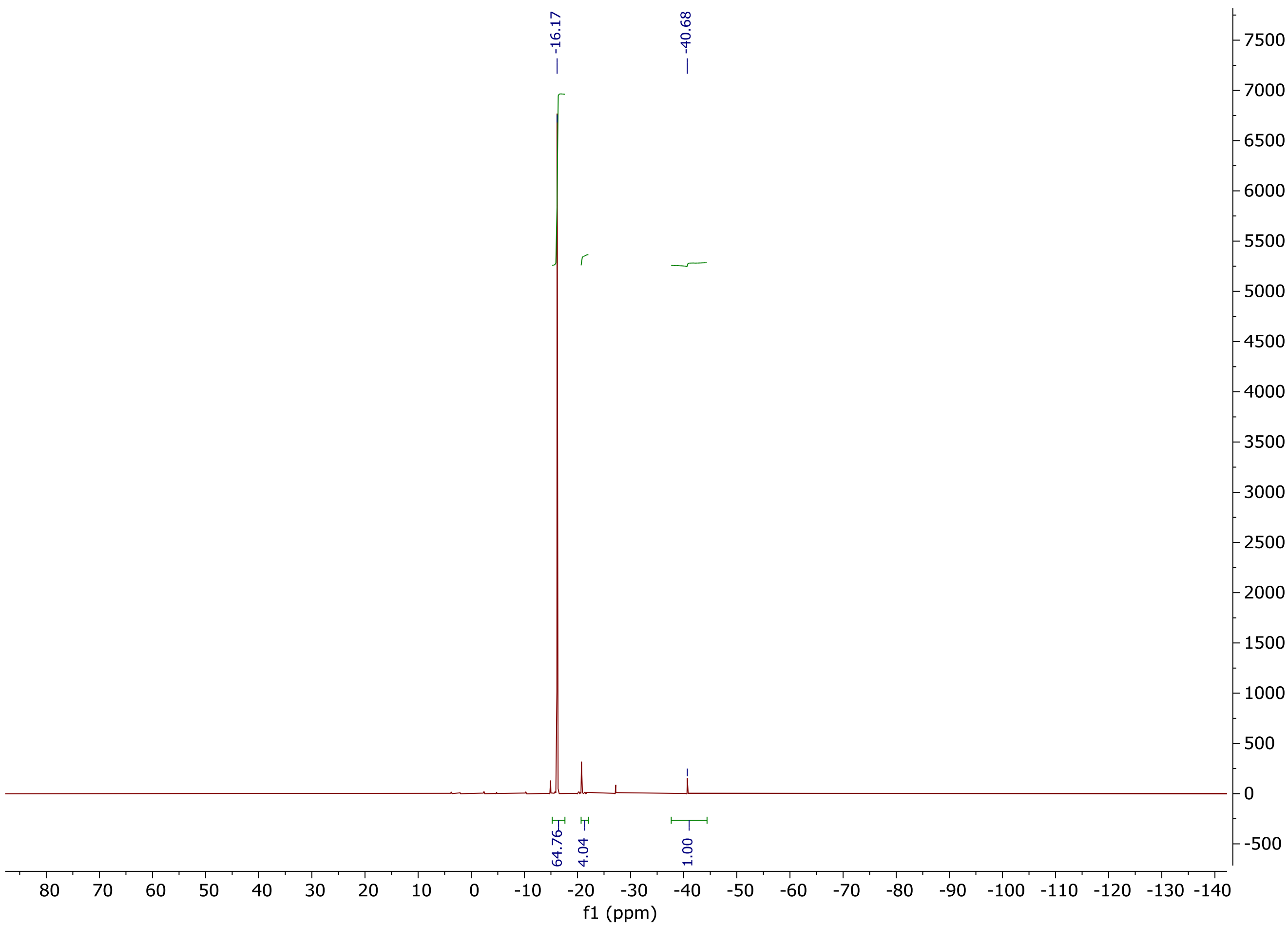

Figure S3: Hydrophosphination of styrene with Ph_2PH in benzene-d_6 using 5 mol% **1** under UV 360 nm irradiation - 26 h – $^{31}P\{^1H\}$ NMR

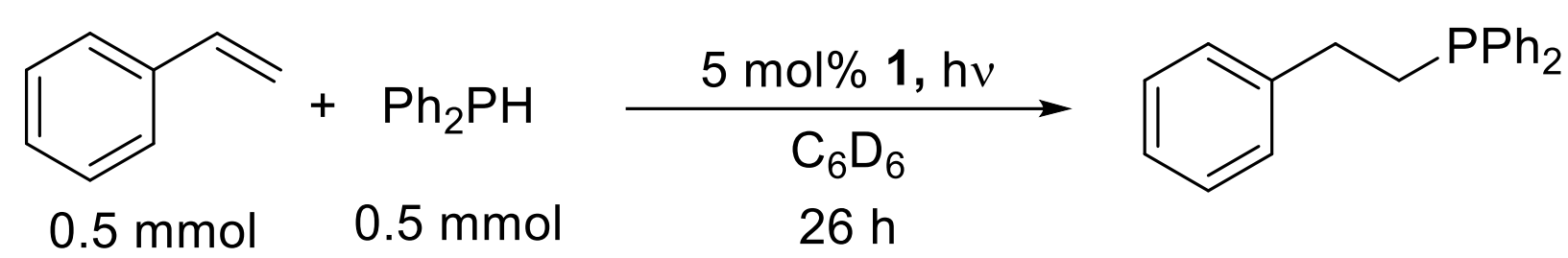

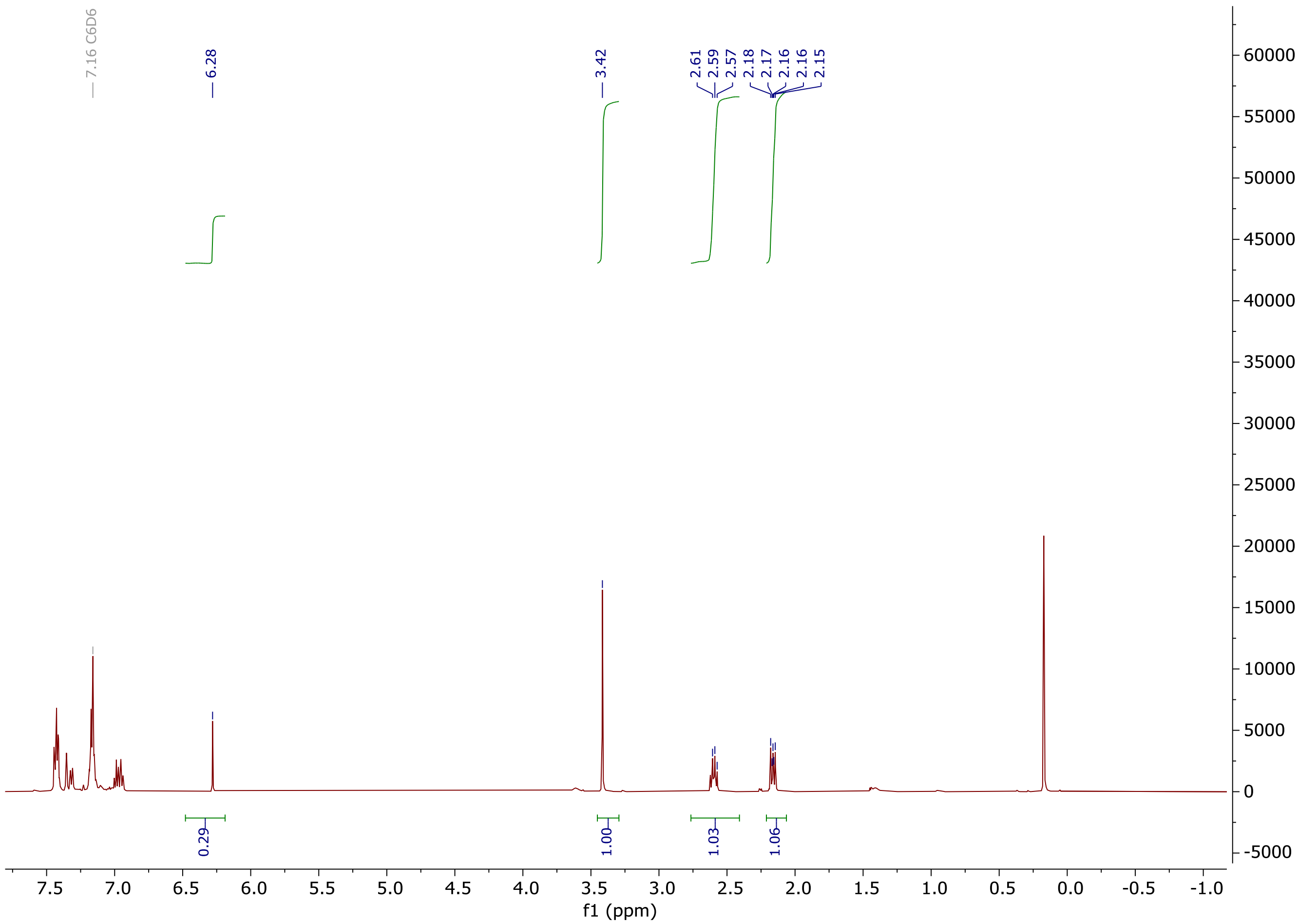

Figure S4: Hydrophosphination of styrene with Ph_2PH in benzene-d_6 using 5 mol% **1** under UV 360 nm irradiation – 26 h – ^{1}H NMR

Hydrophosphination of styrene with Ph_2PH in Acetone-d_6

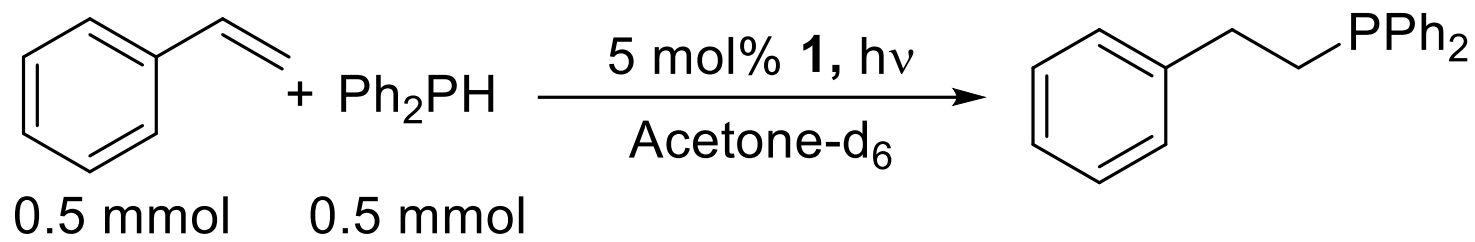

0 h

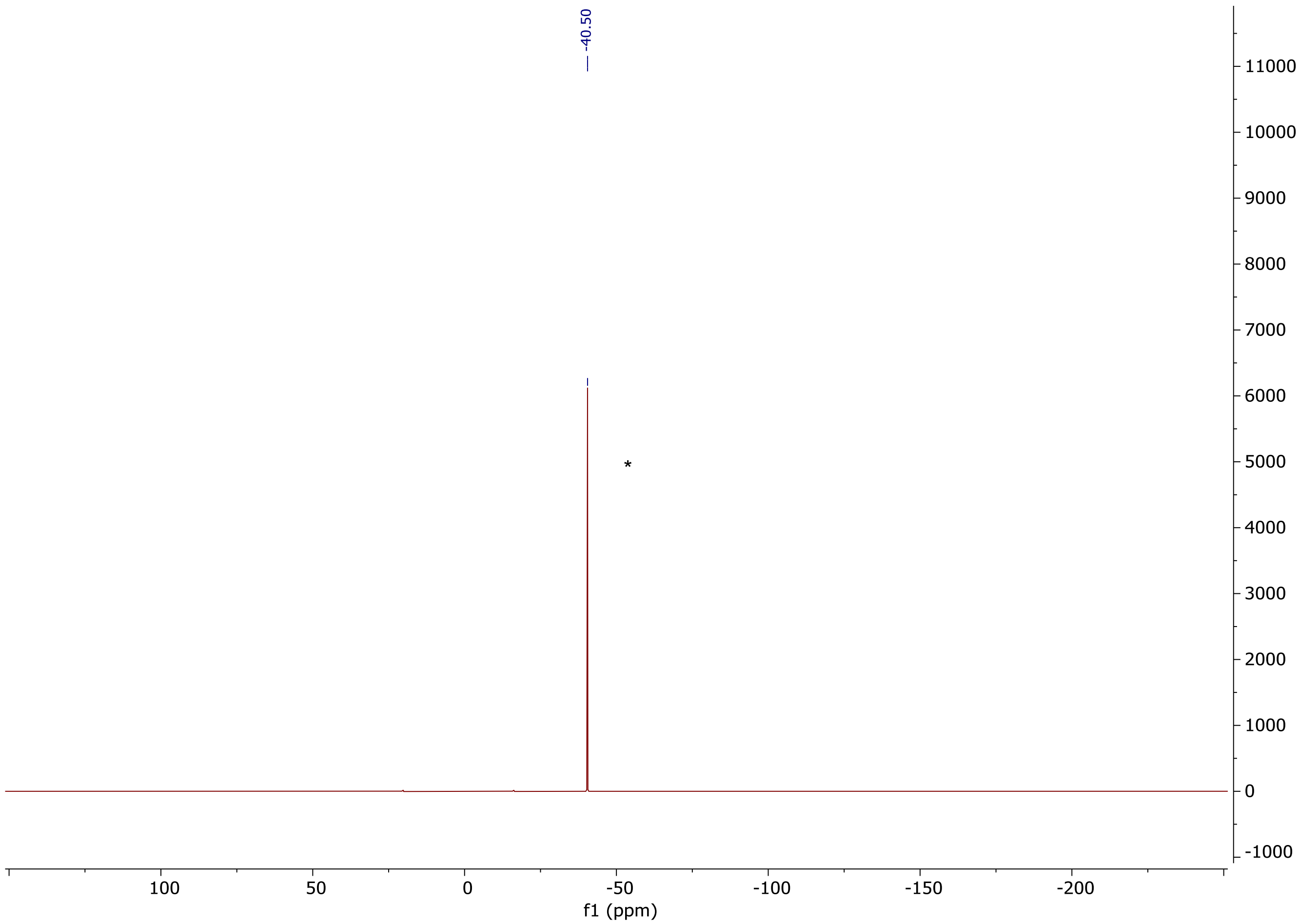

Figure S5: Hydrophosphination of styrene with Ph_2PH in acetone-d_6 using 5 mol% **1** under UV 360 nm irradiation – 0 h – $^{31}P\{^1H\}$ NMR

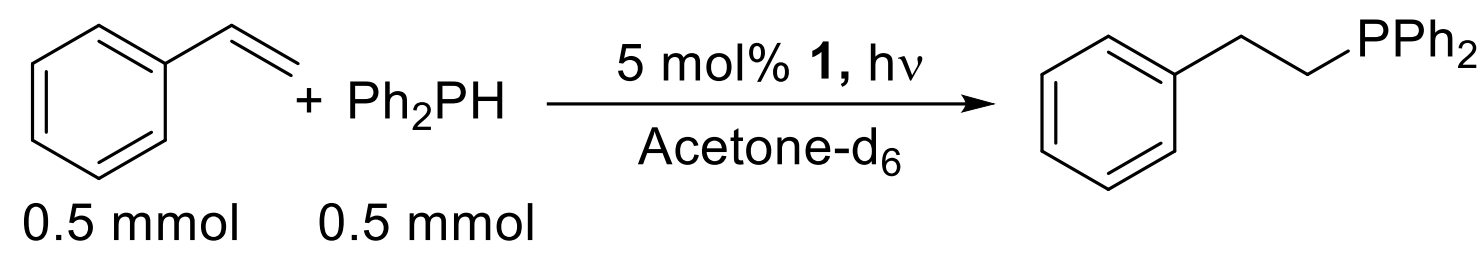

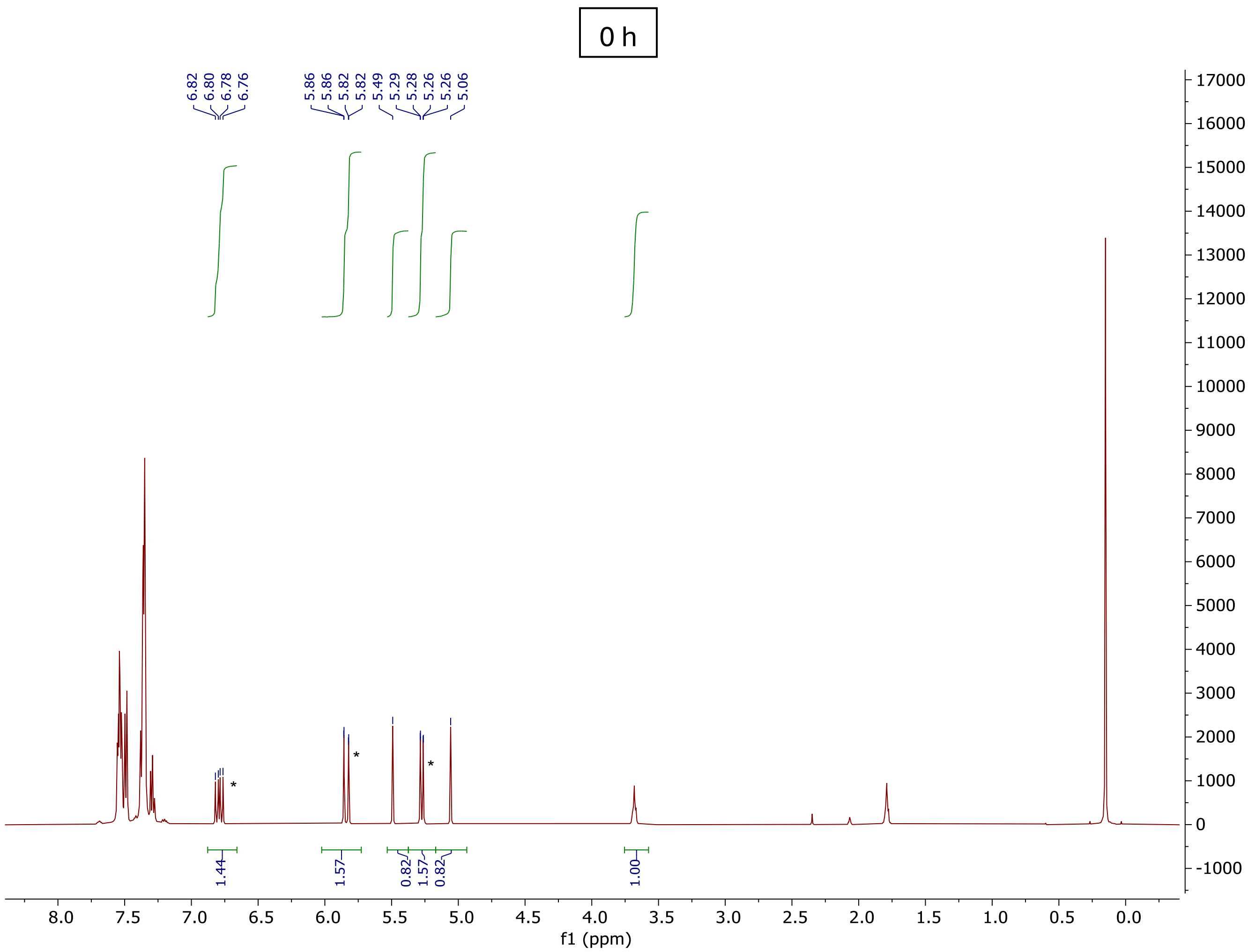

Figure S6: Hydrophosphination of styrene with Ph_2PH in acetone-d_6 using 5 mol% **1** under UV 360 nm irradiation - 0 h – ^{1}H NMR

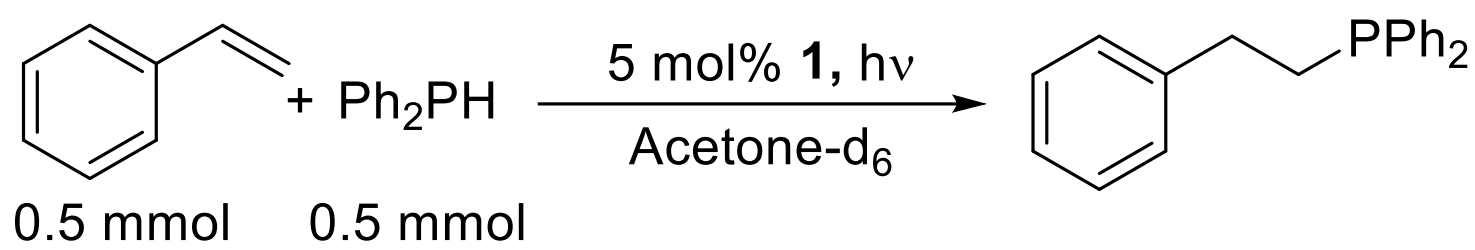

12 h

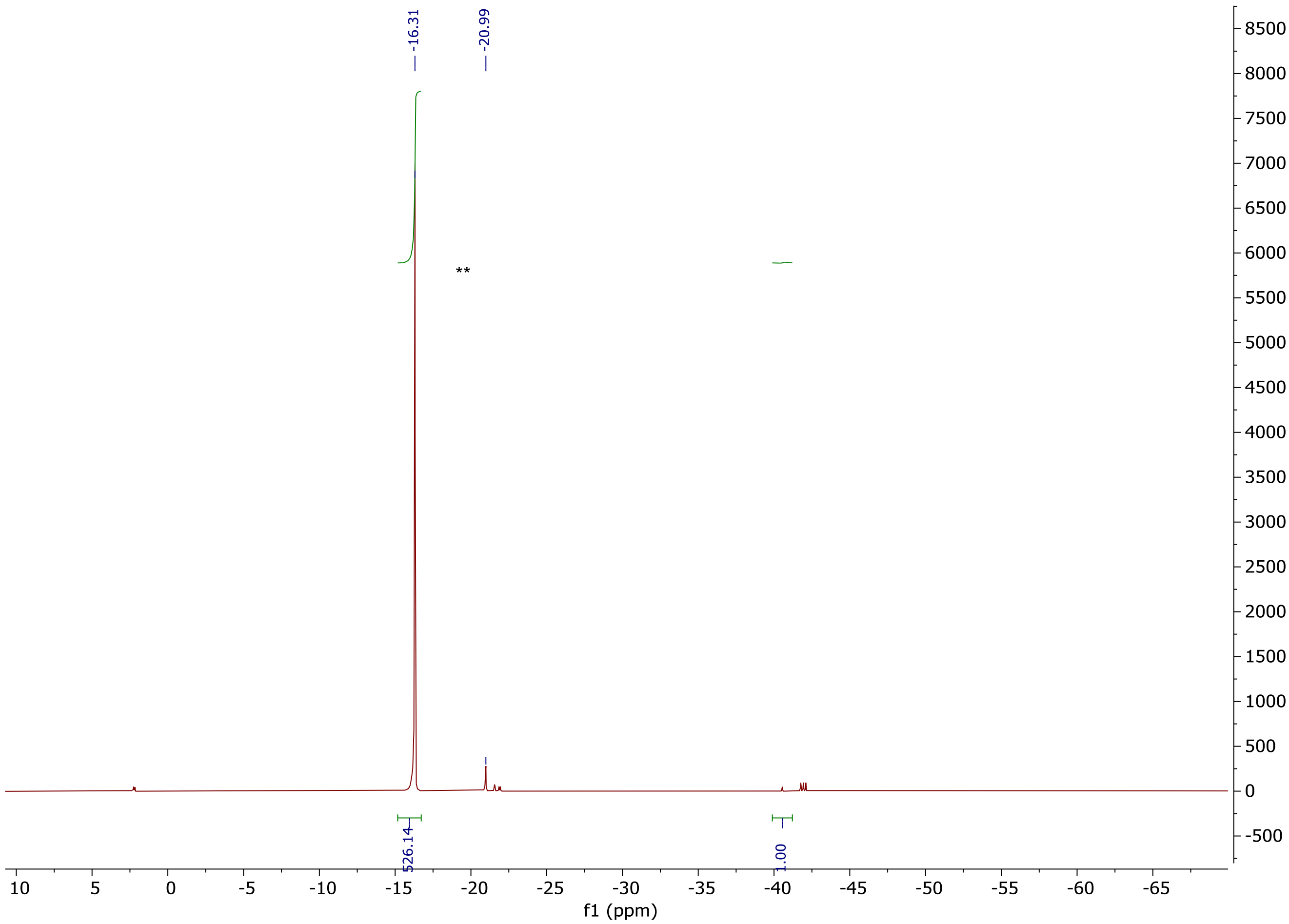

Figure S7: Hydrophosphination of styrene with Ph_2PH in acetone-d_6 using 5 mol% **1** under UV 360 nm irradiation -12 h – $^{31}P\{^1H\}$ NMR

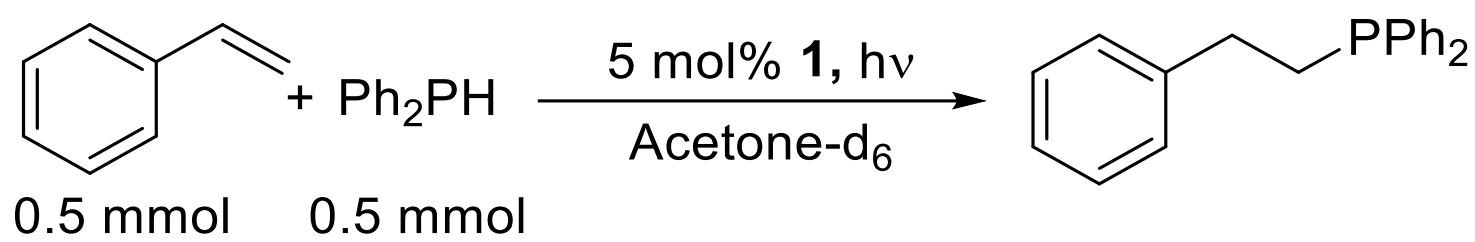

12 h

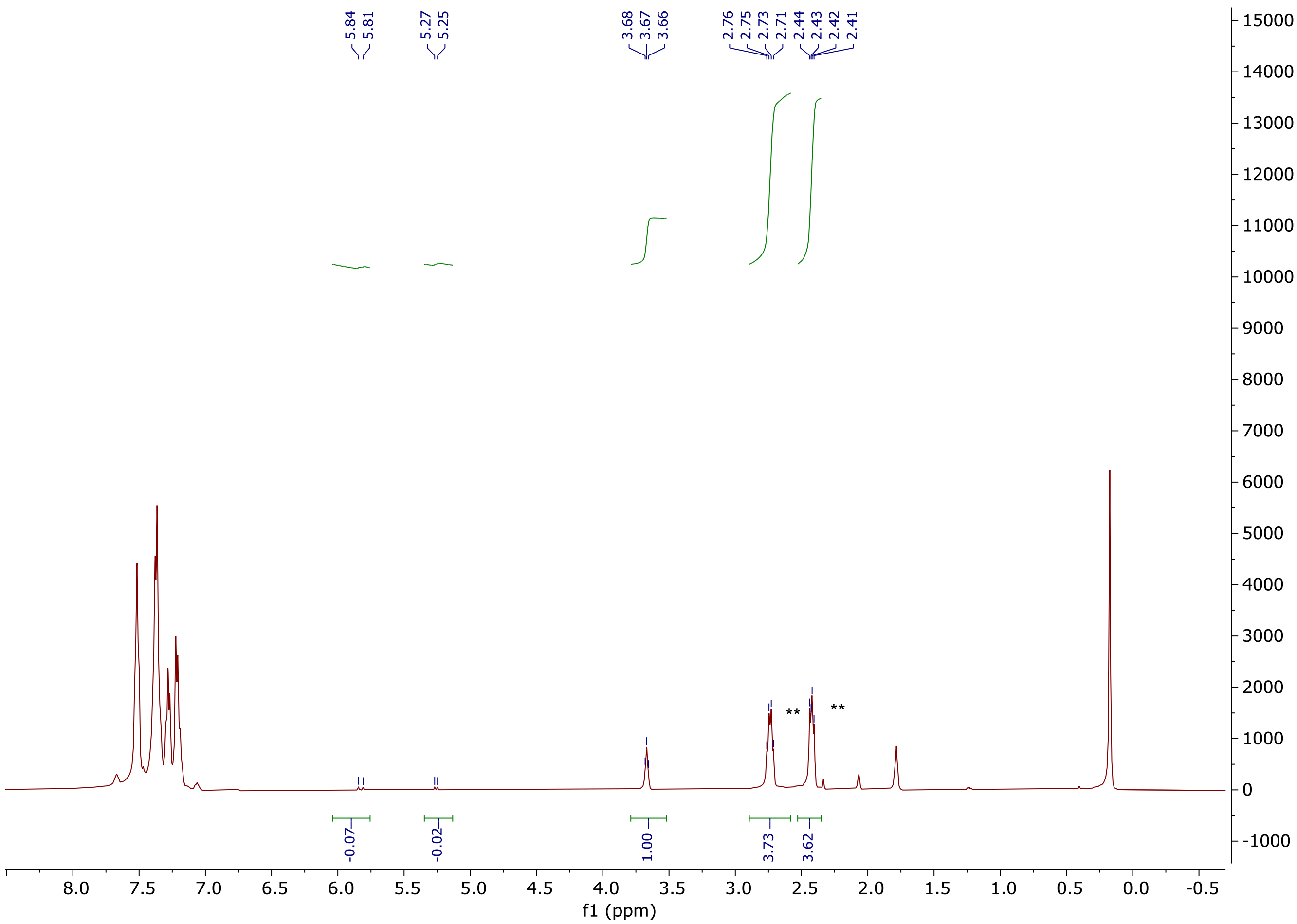

Figure S8: Hydrophosphination of styrene with Ph_2PH in acetone-d_6 using 5 mol% **1** under UV 360 nm irradiation -12 h – ^{1}H NMR

Hydrophosphination of styrene with Ph_2PH in Methanol-d_4

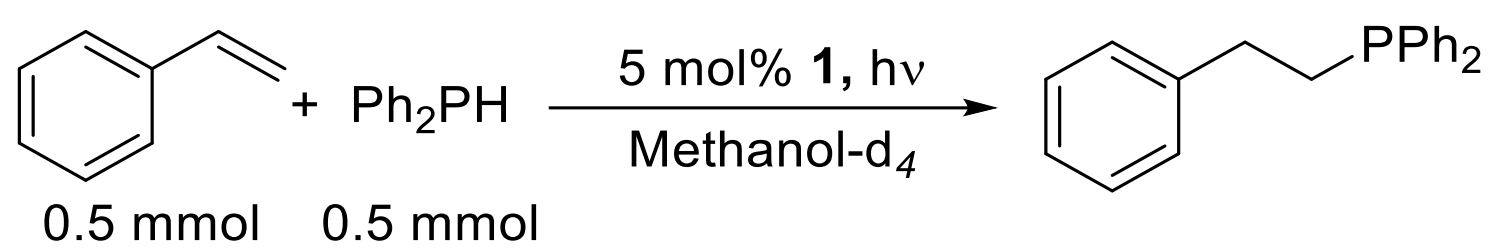

0 h

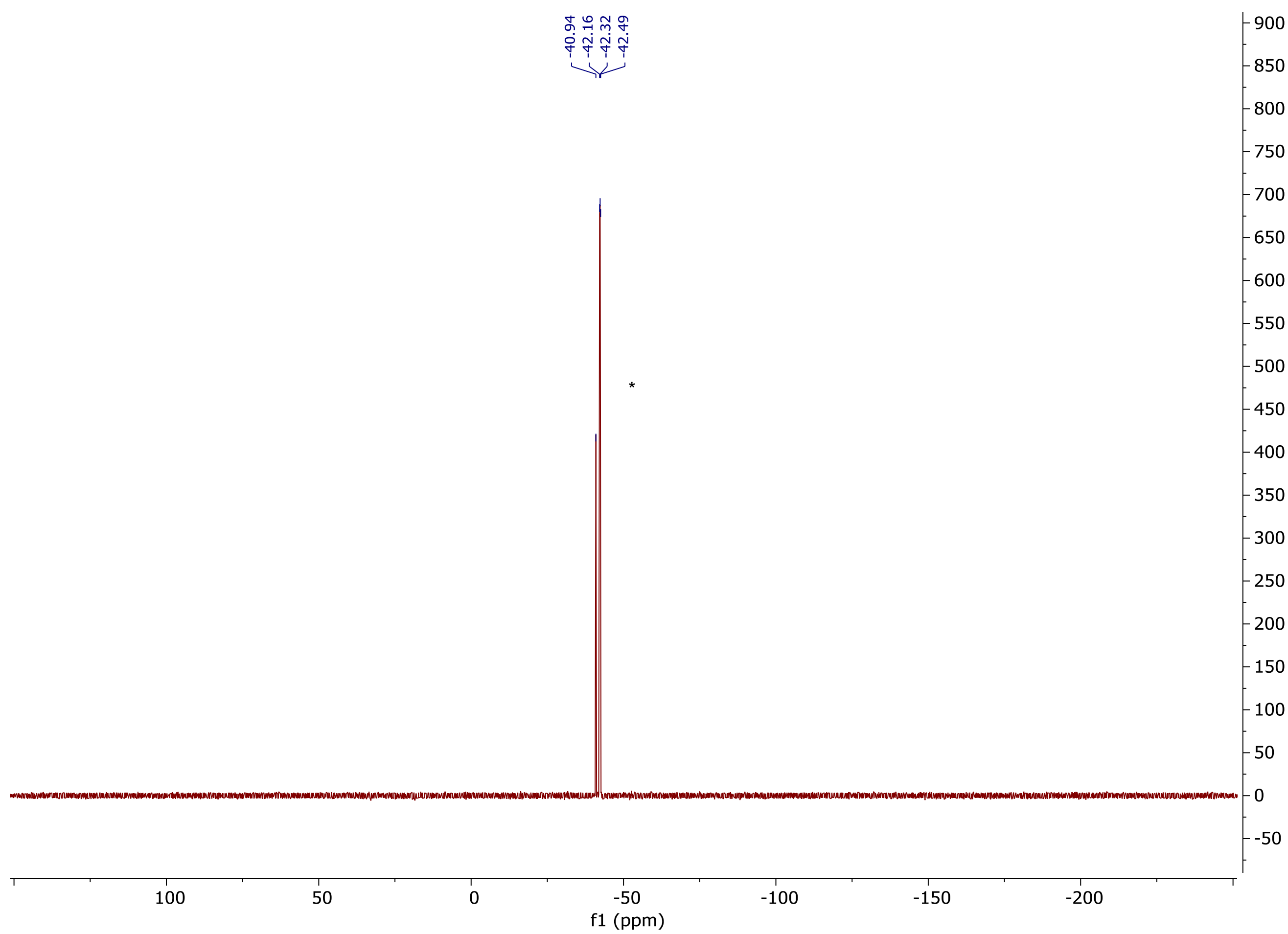

Figure S9: Hydrophosphination of styrene with Ph_2PH in methanol-d_4 using 5 mol% **1** under UV 360 nm irradiation - 0 h – $^{31}P\{^1H\}$ NMR

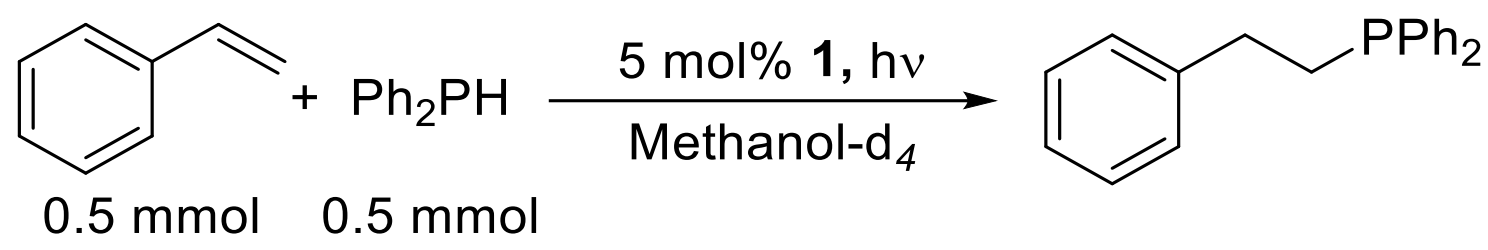

0 h

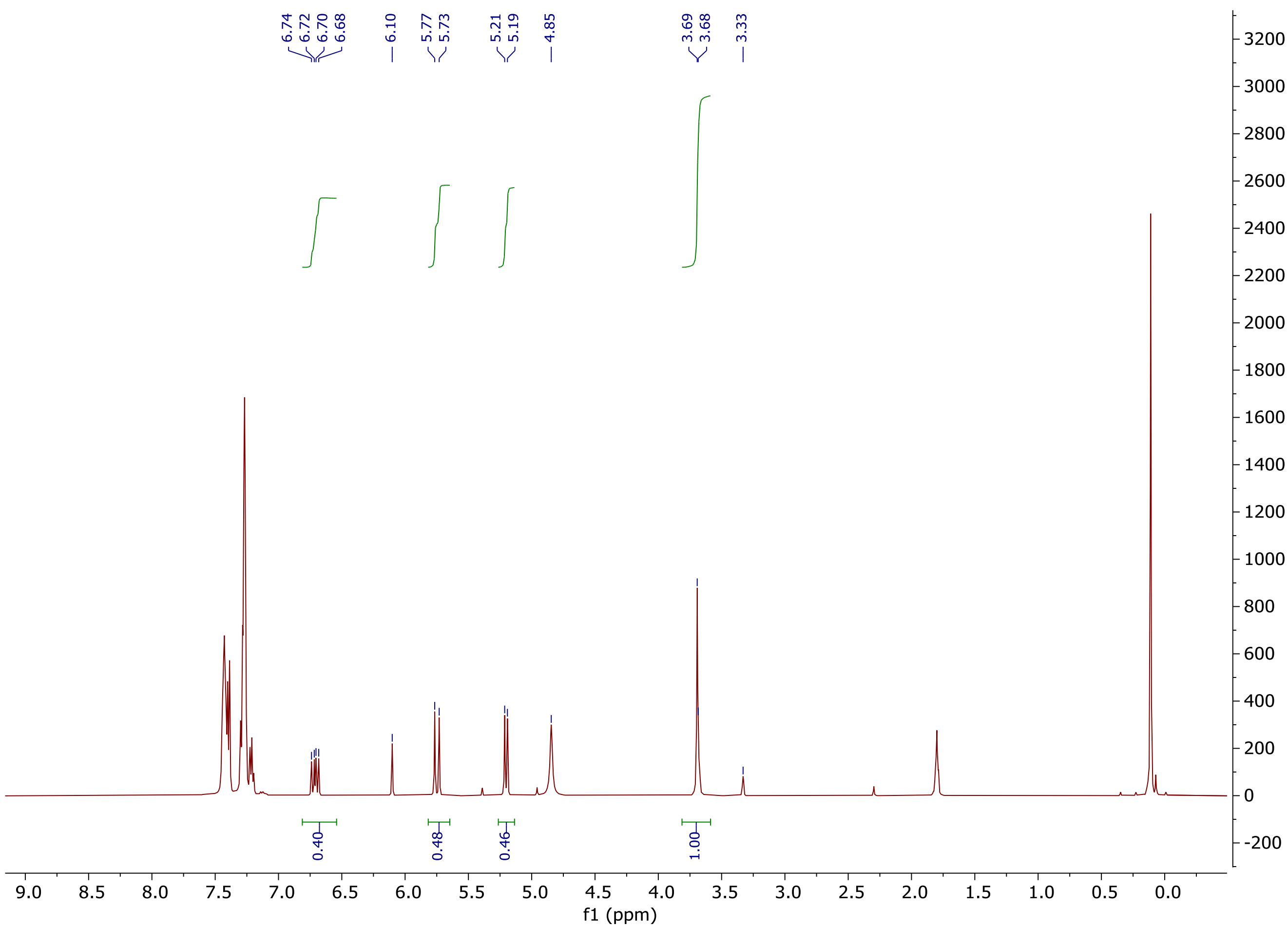

Figure S10: Hydrophosphination of styrene with Ph_2PH in methanol-d_4 using 5 mol% **1** under UV 360 nm irradiation - 0 h – ^1H NMR

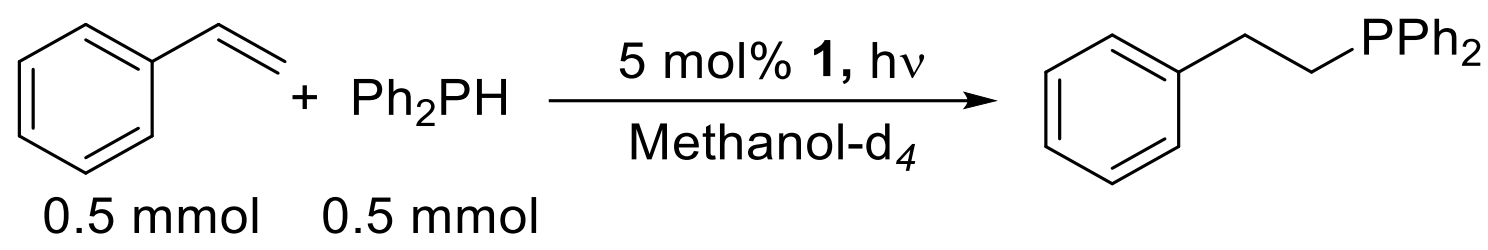

6 h

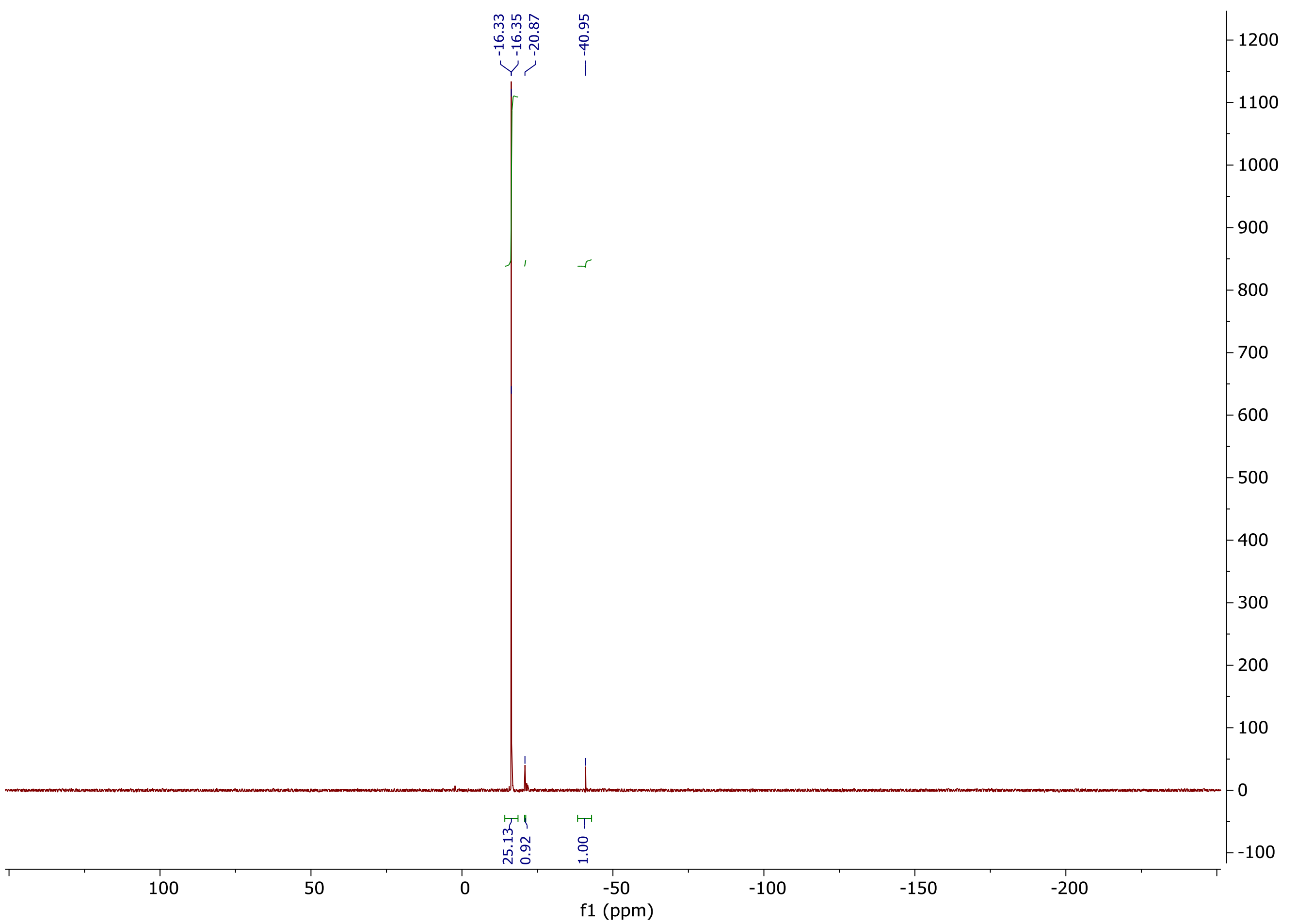

Figure S11: Hydrophosphination of styrene with Ph_2PH in methanol-d_4 using 5 mol% **1** under UV 360 nm irradiation – 6 h – $^{31}P\{^1H\}$ NMR

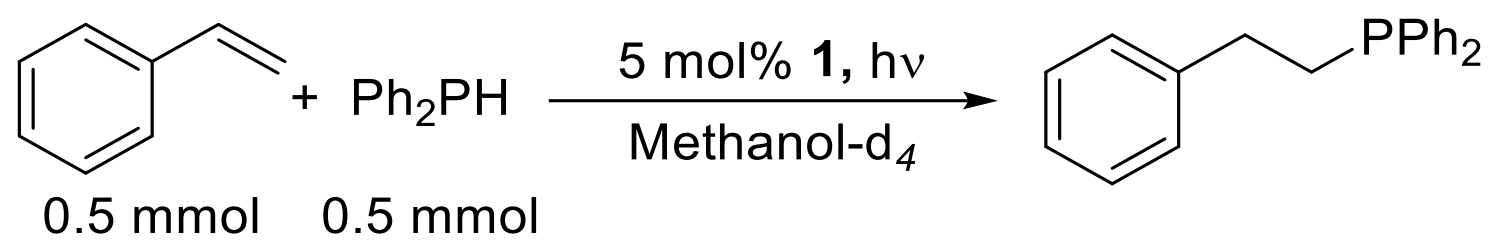

6 h

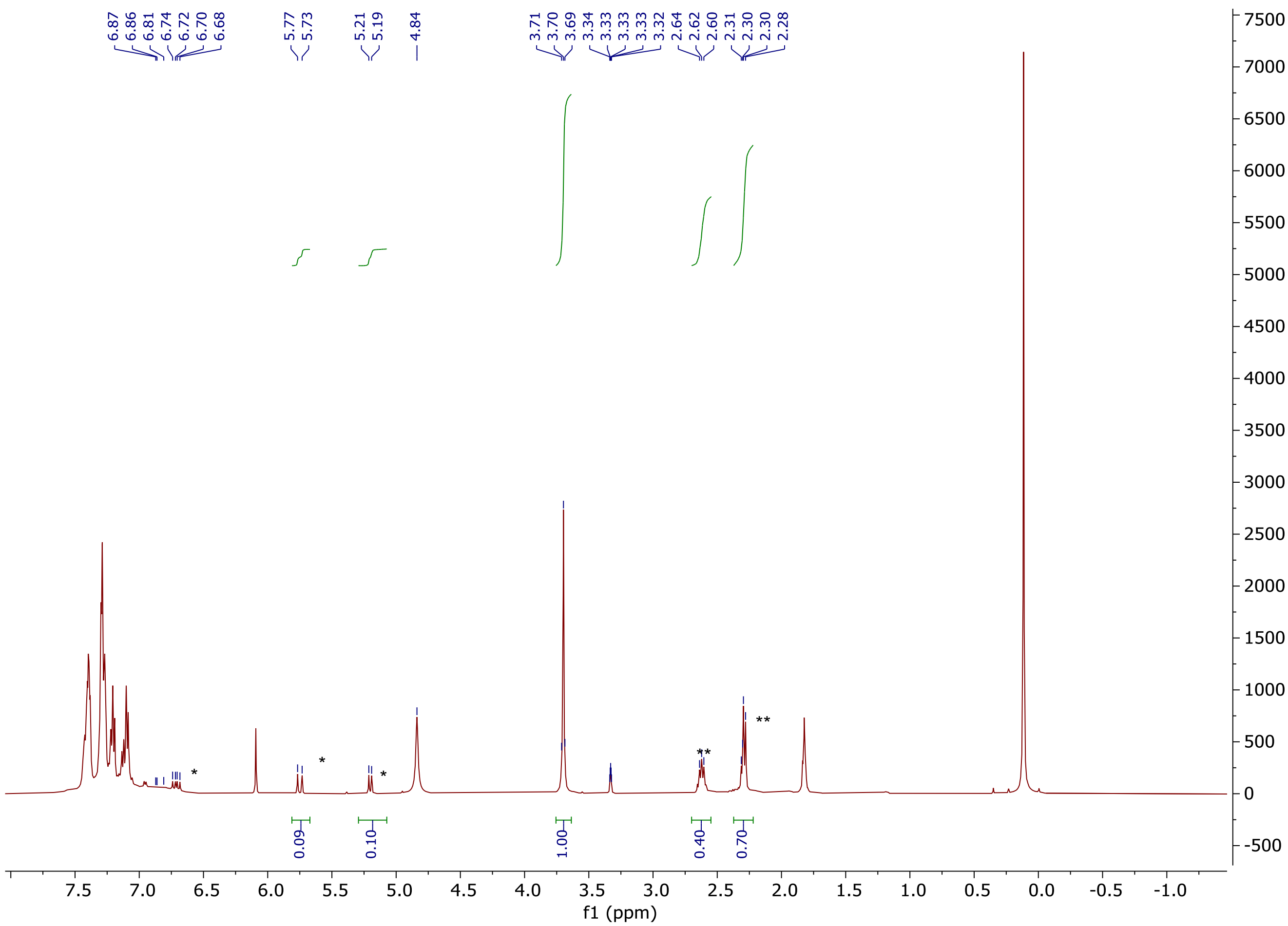

Figure S12: Hydrophosphination of styrene with Ph_2PH in methanol-d_4 using 5 mol% **1** under UV 360 nm irradiation – 6 h – ^1H NMR

Hydrophosphination of styrene with Ph_2PH in acetonitrile-d_3

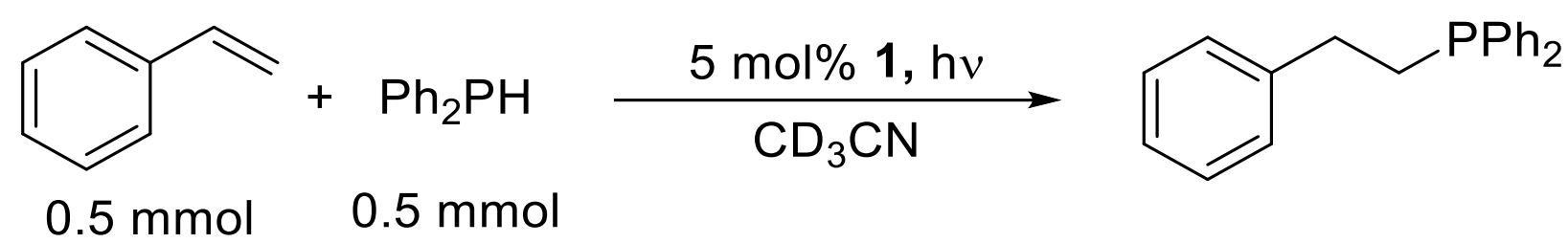

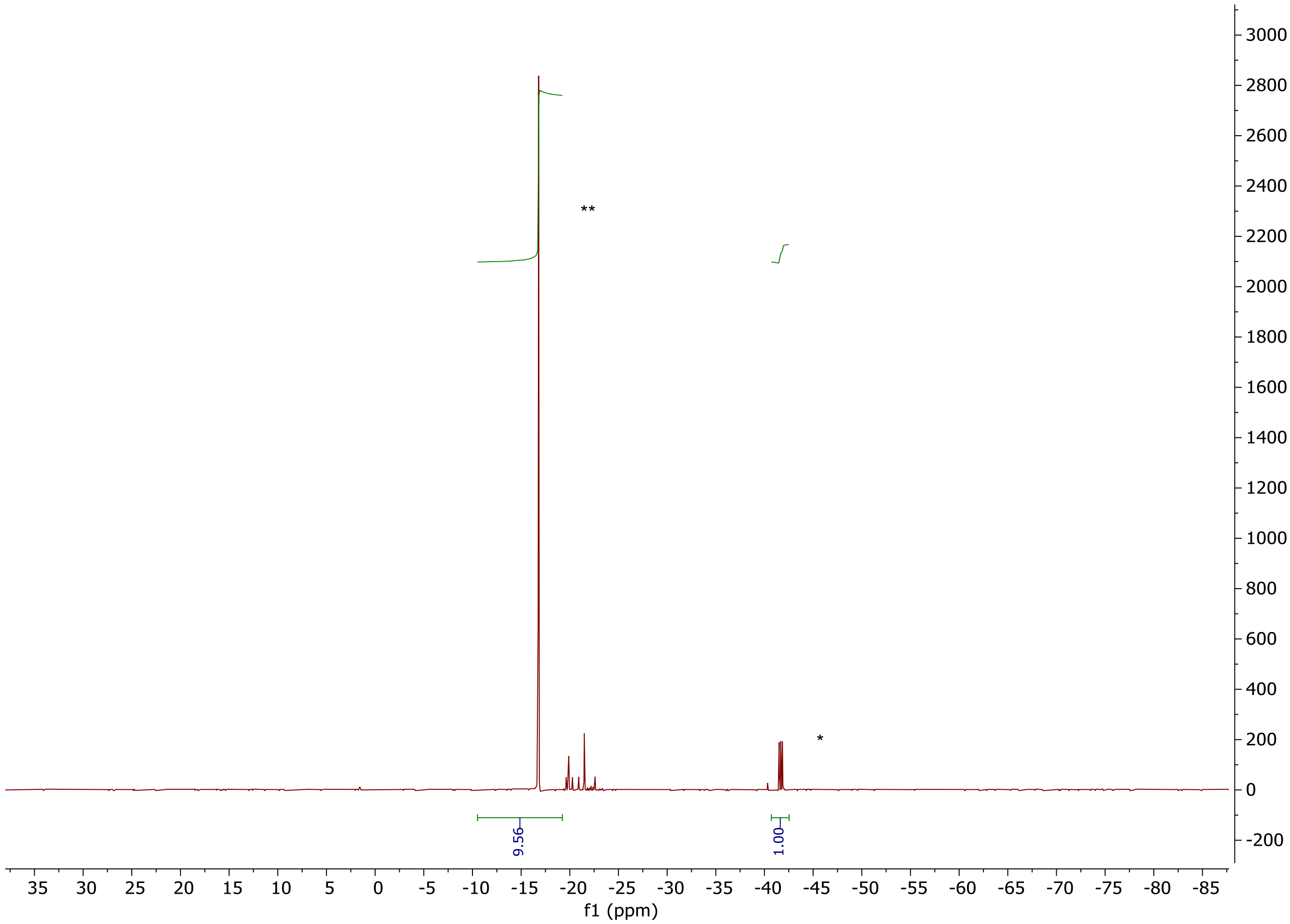

Figure S13: Hydrophosphination of styrene with Ph_2PH in acetonitrile-d_3 using 5 mol% **1** under UV 360 nm irradiation – 1 h – $^{31}P\{^1H\}$ NMR

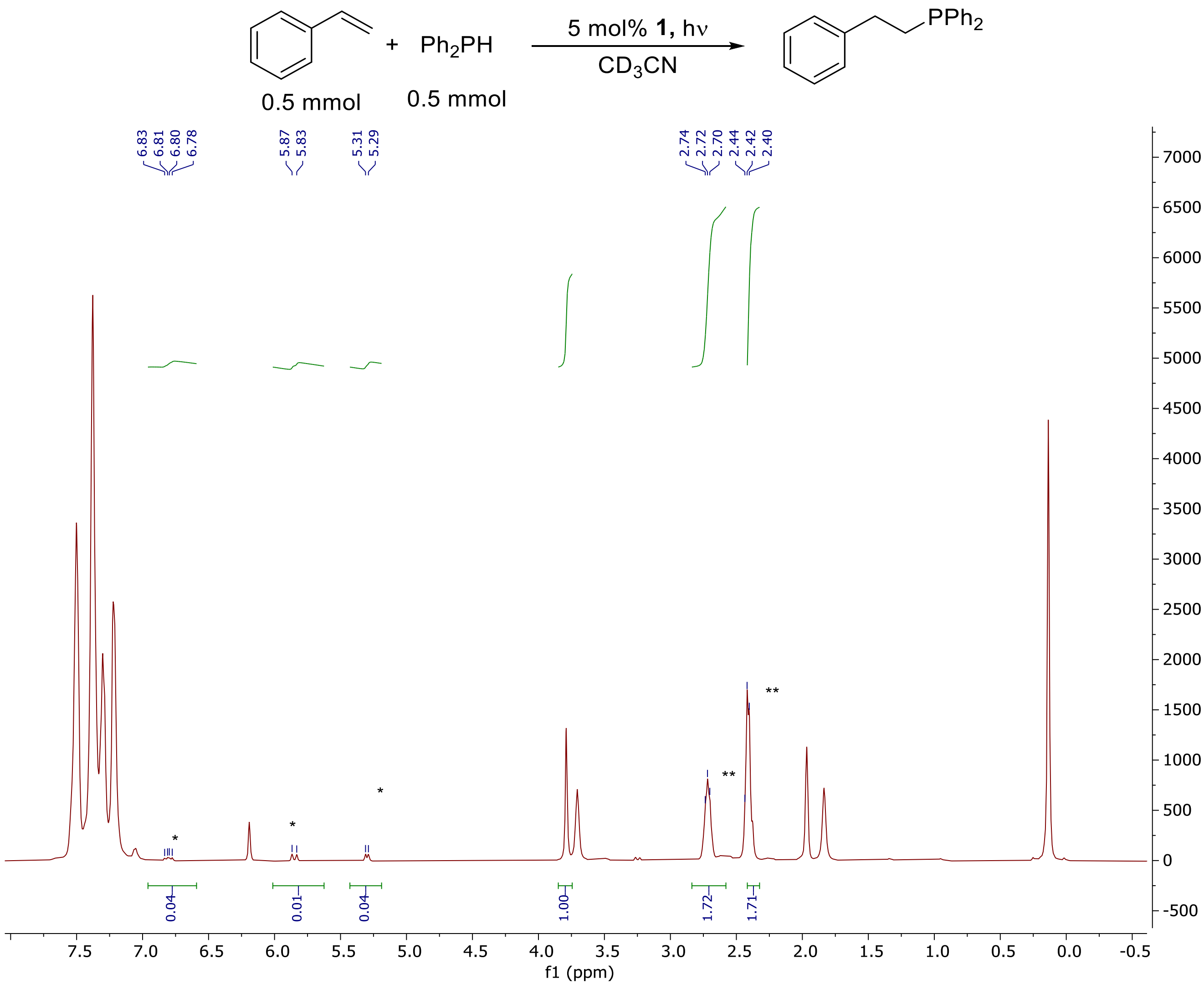

Figure S14: Hydrophosphination of styrene with Ph_2PH in acetonitrile-d_3 using 5 mol% **1** under UV 360 nm irradiation – 1 h – ^{1}H NMR

Hydrophosphination of unsaturated compounds with Ph_2PH in DMSO using 5 mol% of **1**

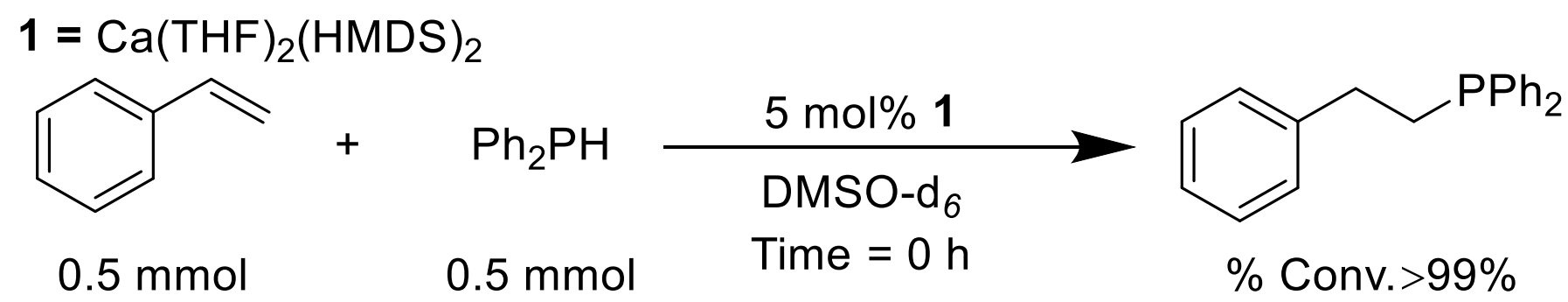

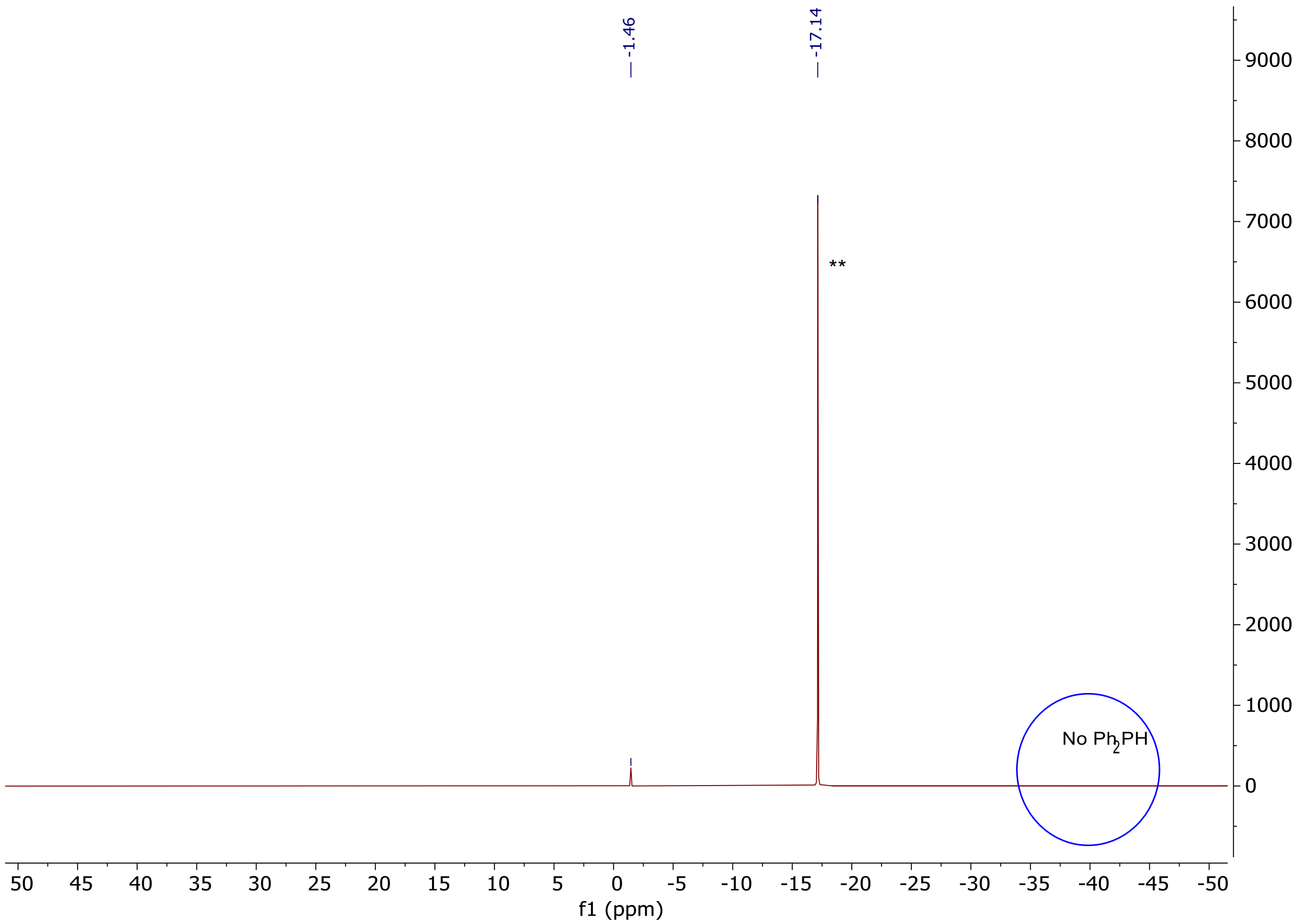

Figure S15: Hydrophosphination of styrene with Ph_2PH in DMSO-d_6 using 5 mol% **1**, and 1,3,5-trimethoxybenzene as internal standard – $^{31}P\{^1H\}$ NMR

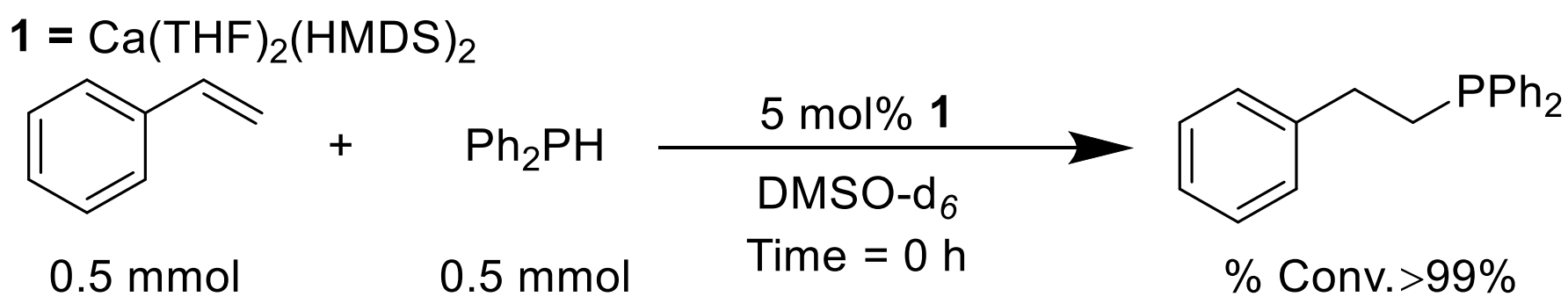

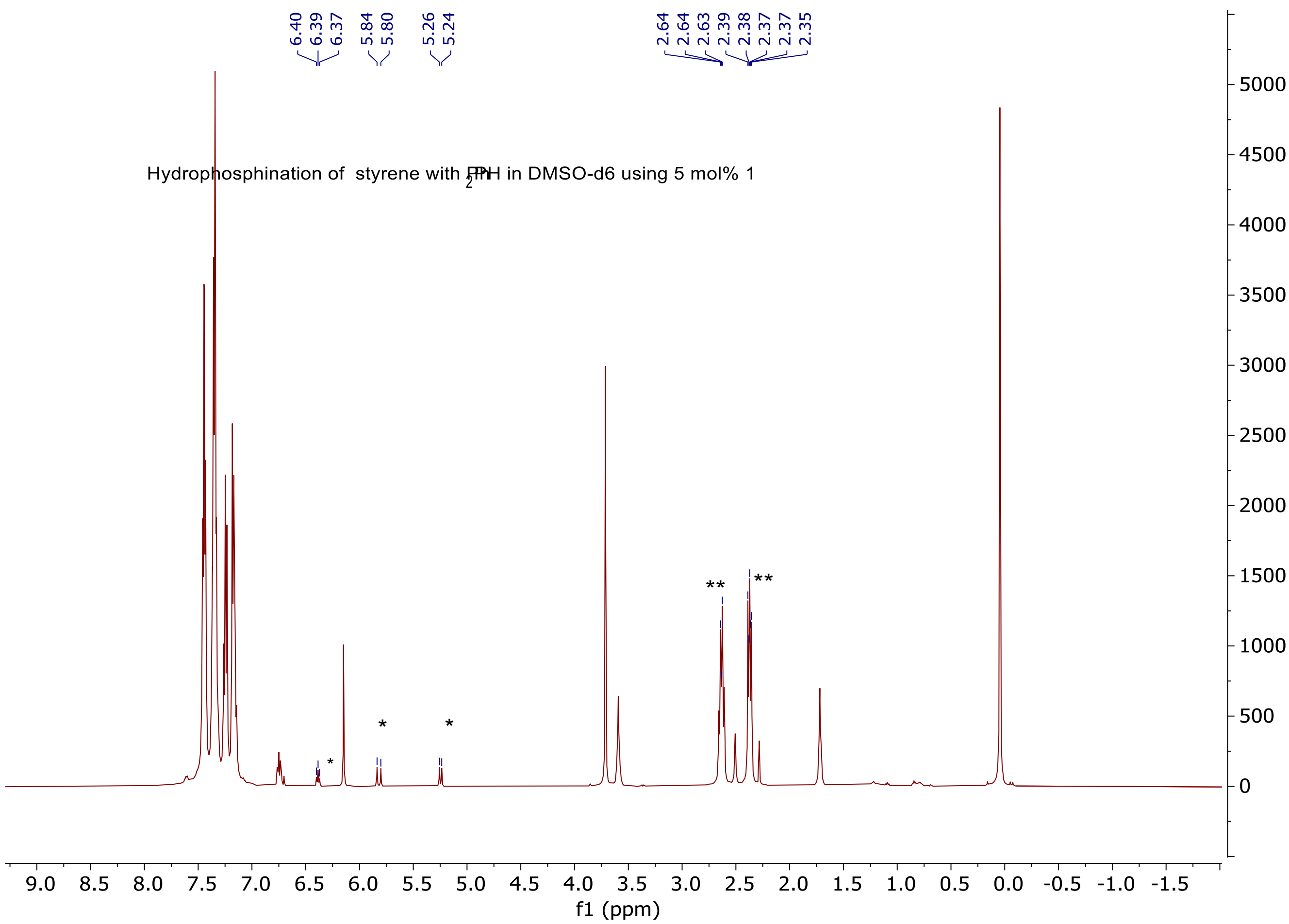

Figure S16: Hydrophosphination of styrene with Ph_2PH in DMSO-d_6 using 5 mol% **1**, and 1,3,5-trimethoxybenzene as internal standard – ^1H NMR

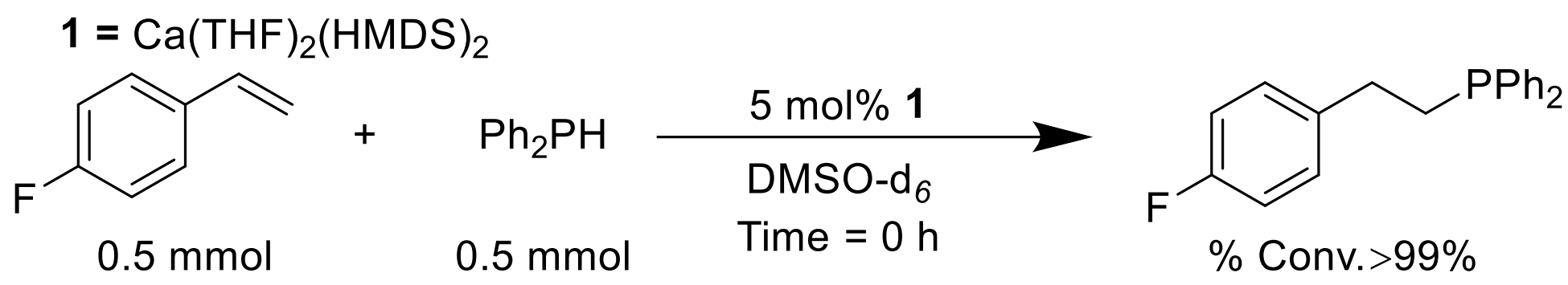

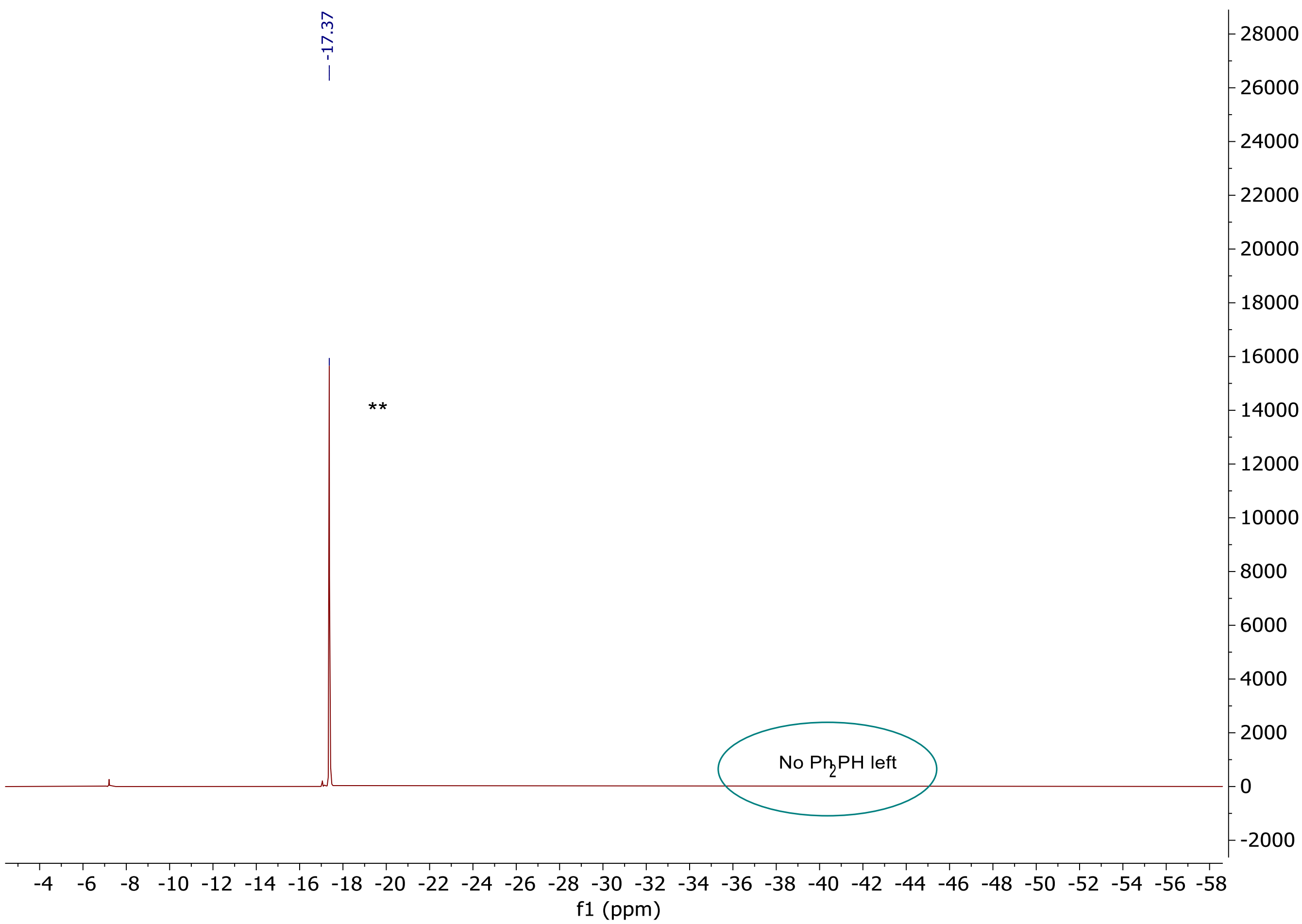

Figure S17: Hydrophosphination of 4-fluoro styrene with Ph_2PH in DMSO-d_6 at ambient conditions using 5 mol% **1,** and 1,3,5 – trimethoxybenzene as internal standard – 0 h – $^{31}P\{^1H\}$ NMR

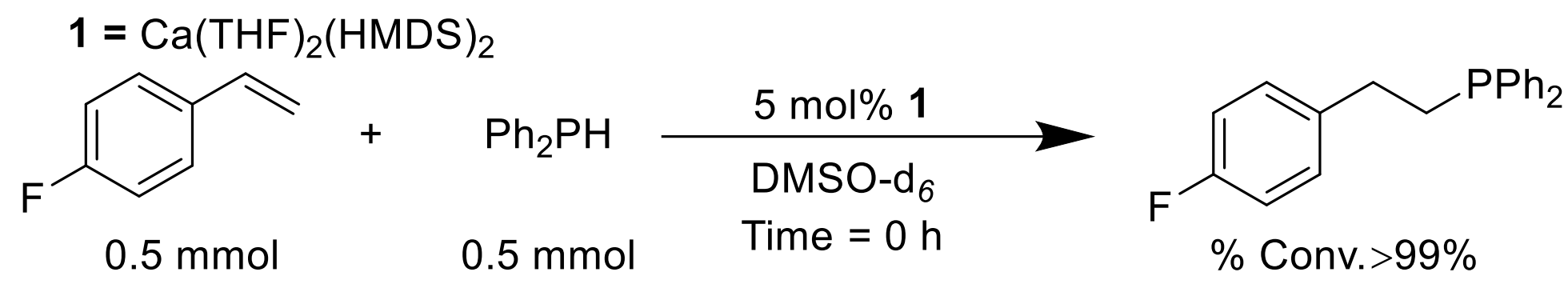

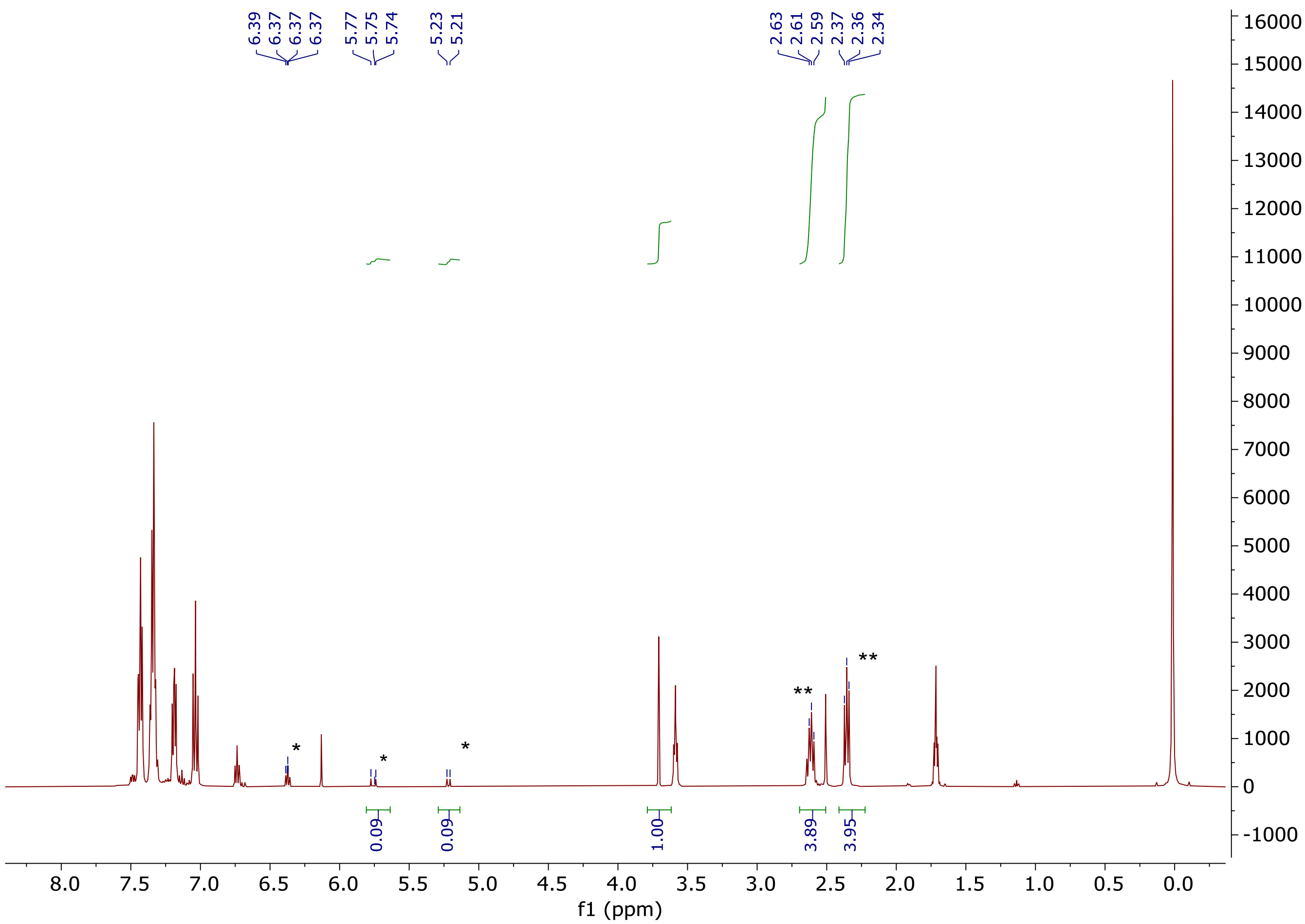

*

Figure S18: Hydrophosphination of 4-fluoro styrene with Ph_2PH in DMSO-d_6 at ambient conditions using 5 mol% **1,** and 1,3,5 – trimethoxybenzene as internal standard – 0 h – ^{1}H NMR

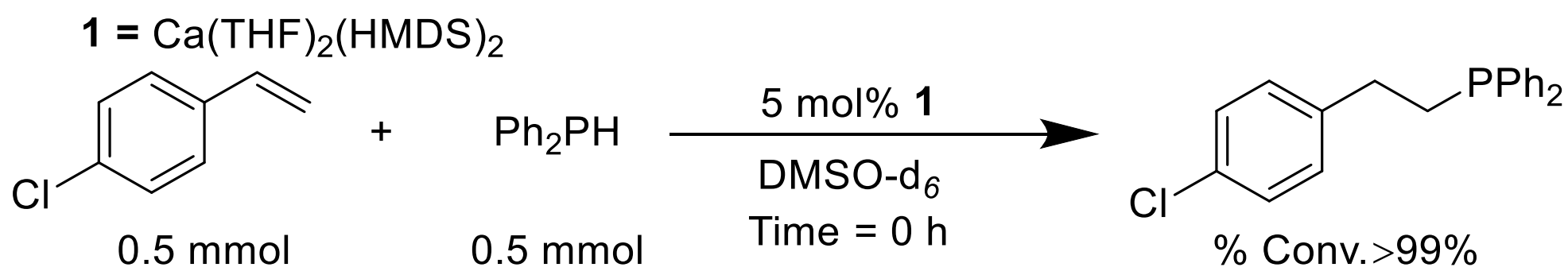

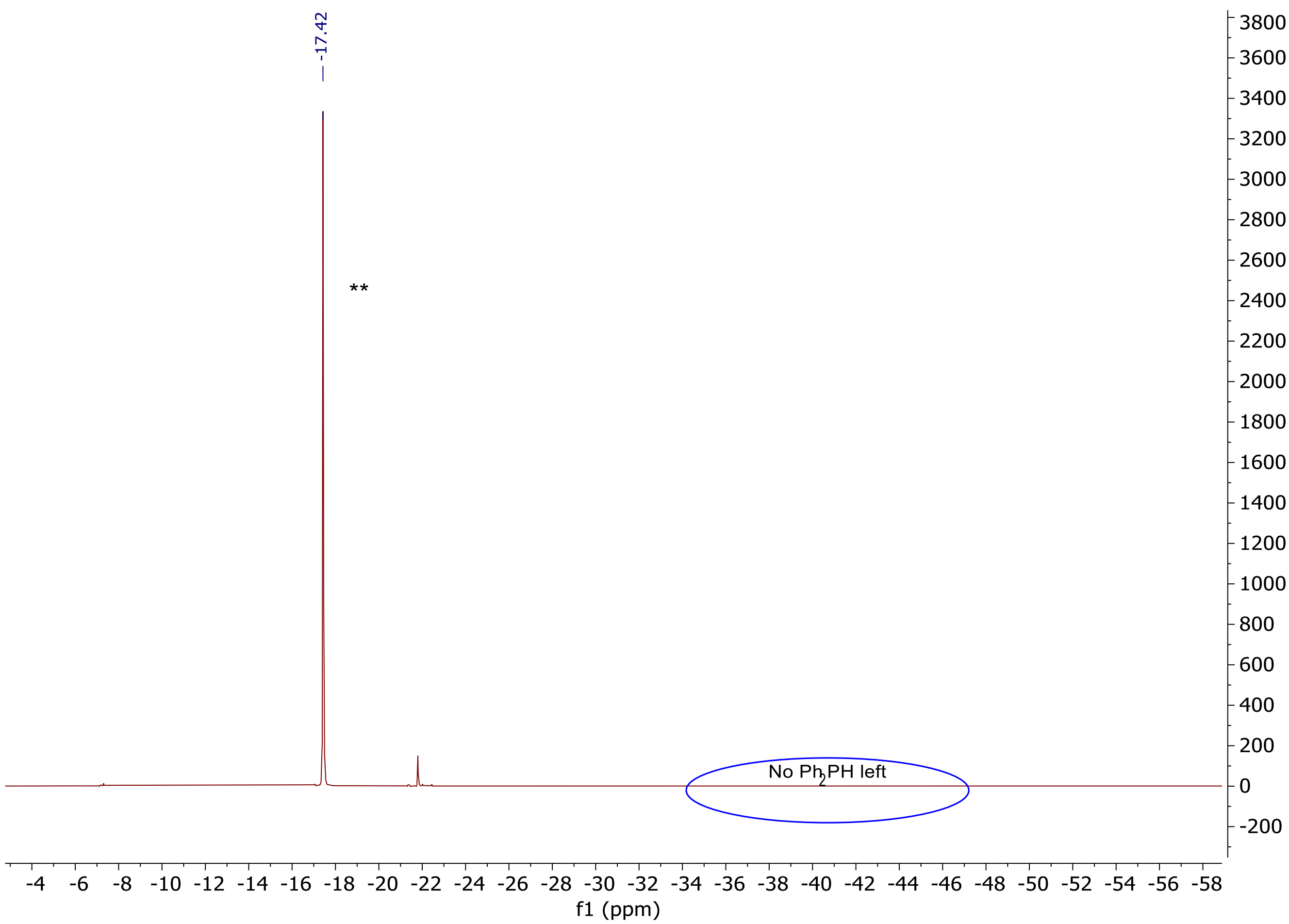

Figure S19: Hydrophosphination of 4-chloro styrene with Ph_2PH in DMSO-d_6 at ambient conditions using 5 mol% **1,** and 1,3,5 – trimethoxybenzene as internal standard – 0 h – $^{31}P\{^1H\}$ NMR

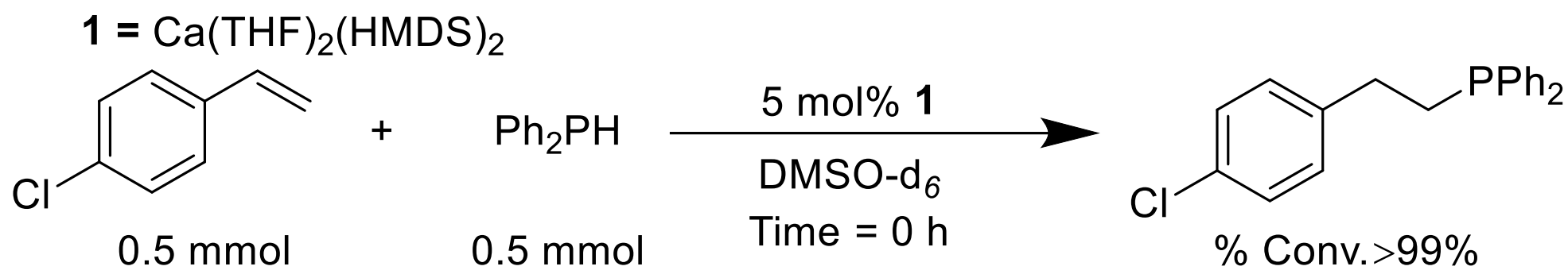

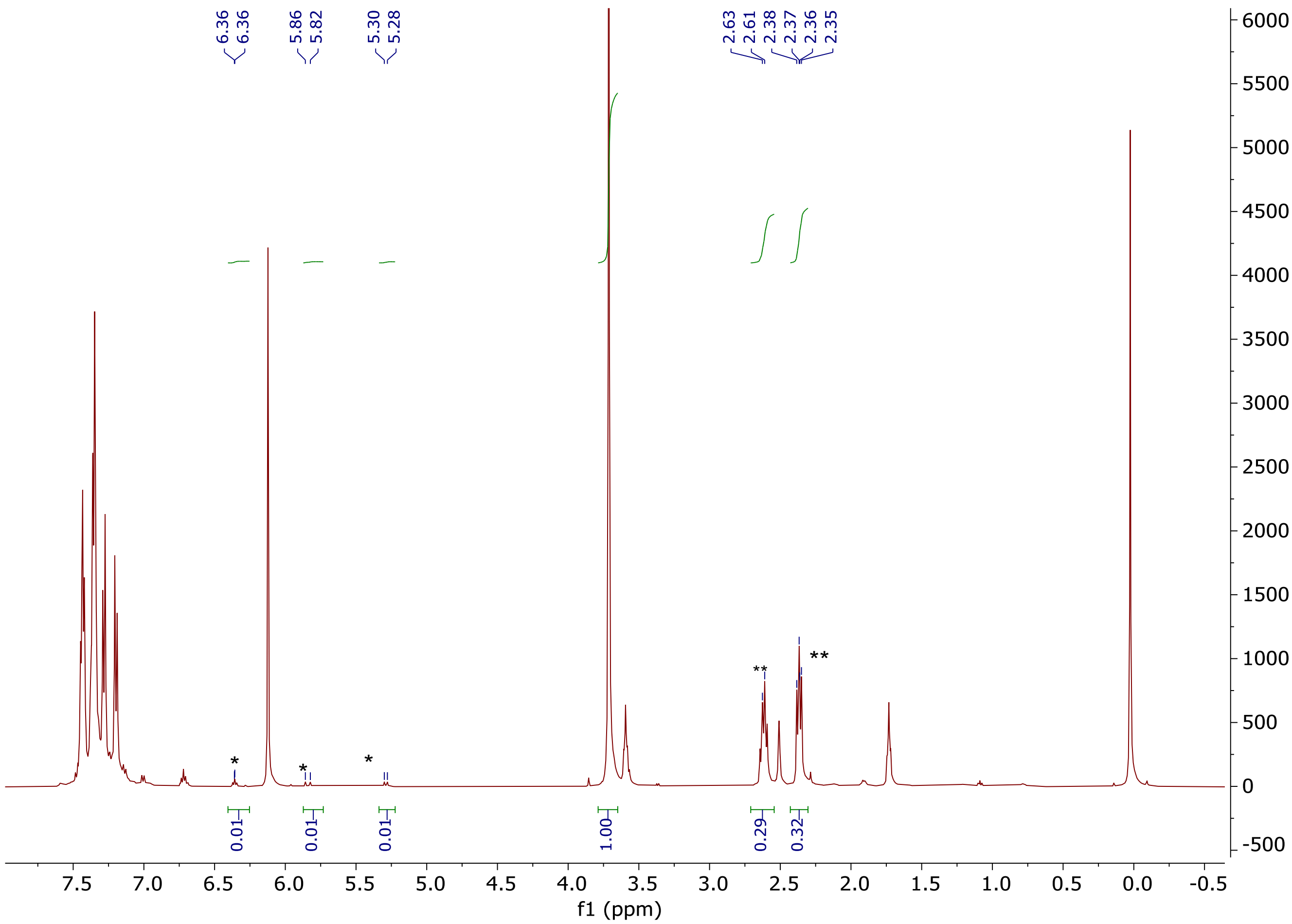

Figure S20: Hydrophosphination of 4-chloro styrene with Ph_2PH in DMSO-d_6 at ambient conditions using 5 mol% **1,** and 1,3,5 – trimethoxybenzene as internal standard – 0 h – ^{1}H NMR

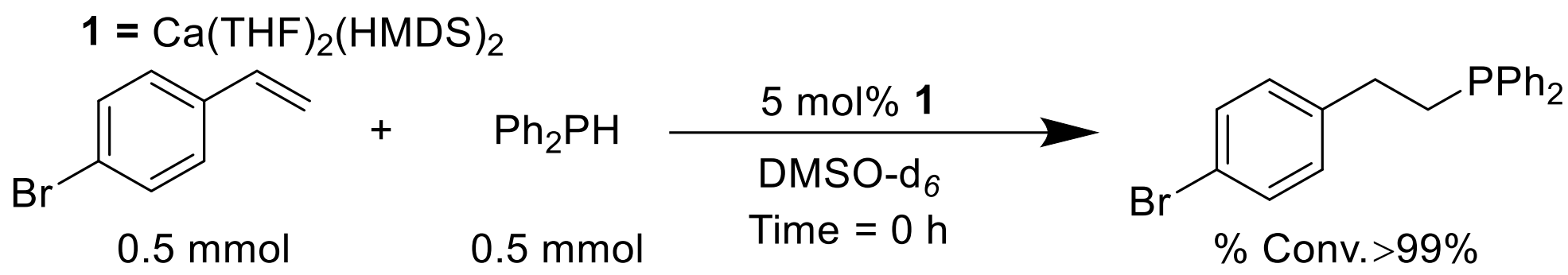

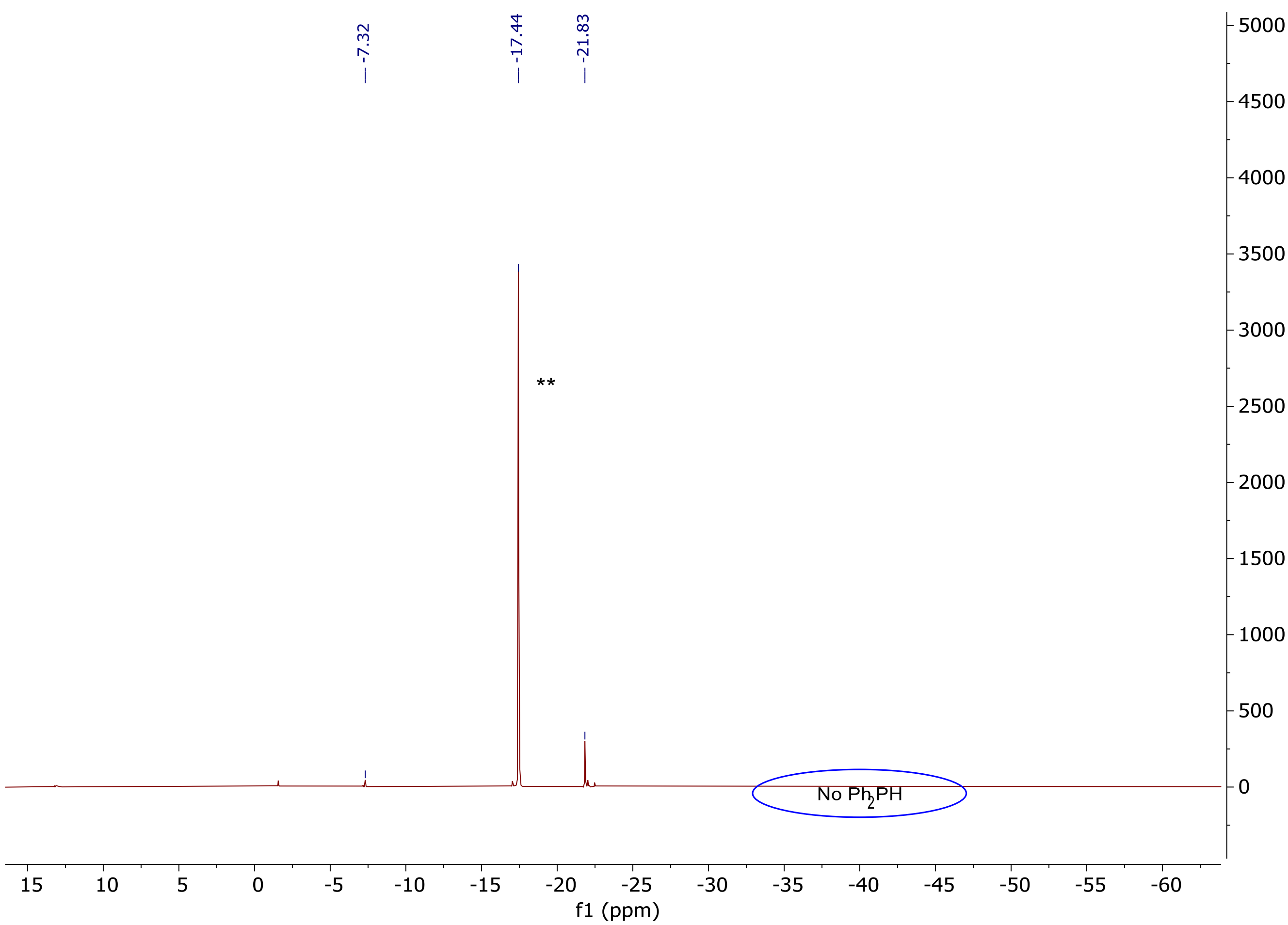

Figure S21: Hydrophosphination of 4-bromo styrene with Ph_2PH in DMSO-d_6 at ambient conditions using 5 mol% **1,** and 1,3,5 – trimethoxybenzene as internal standard – 0 h – $^{31}P\{^1H\}$ NMR

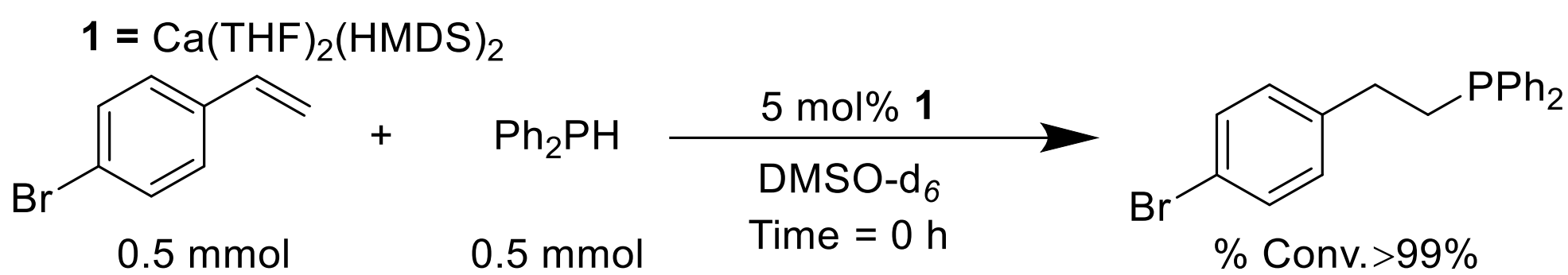

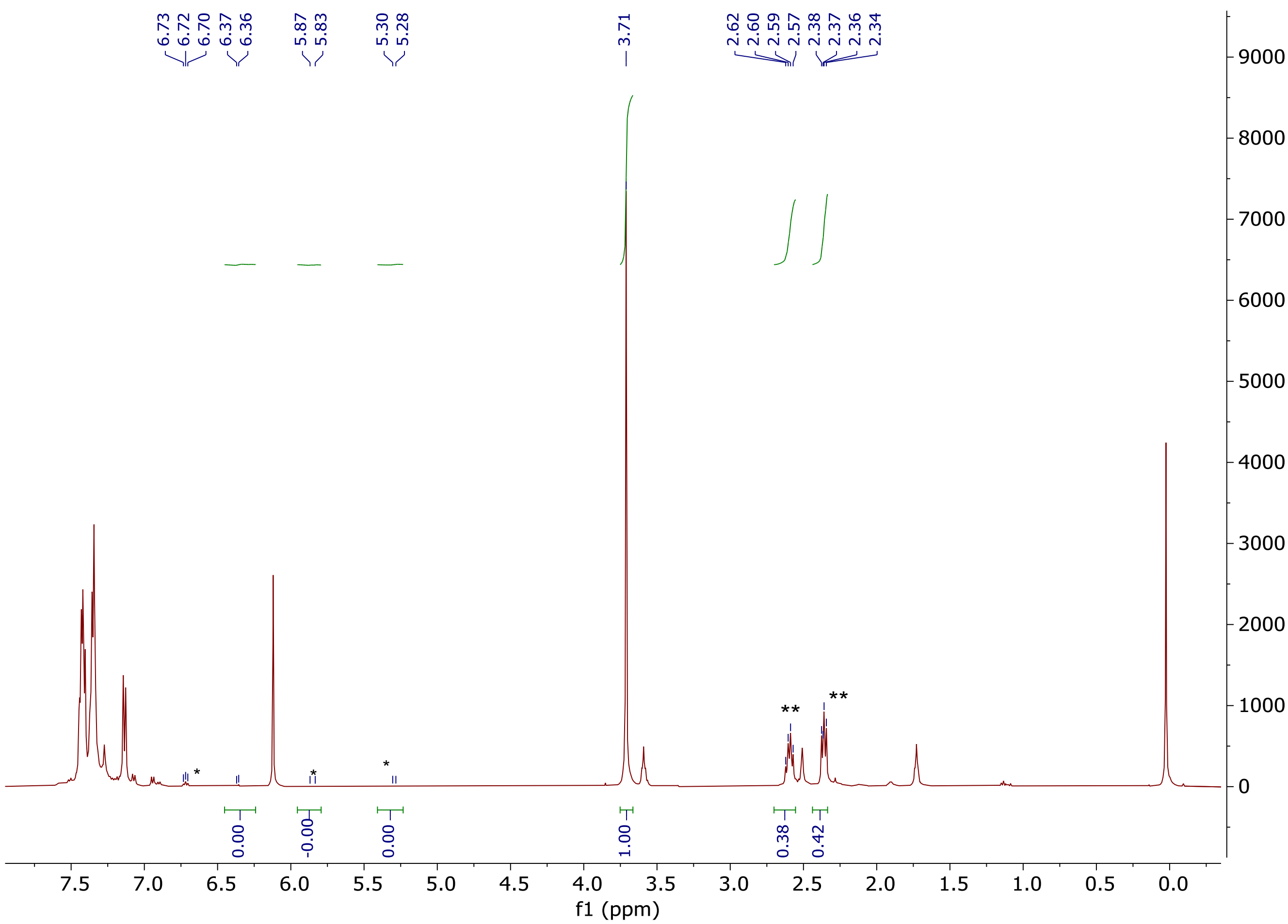

Figure S22: Hydrophosphination of 4-bromo styrene with Ph_2PH in DMSO-d_6 at ambient conditions using 5 mol% **1,** and 1,3,5 – trimethoxybenzene as internal standard – 0 h – ^{1}H NMR

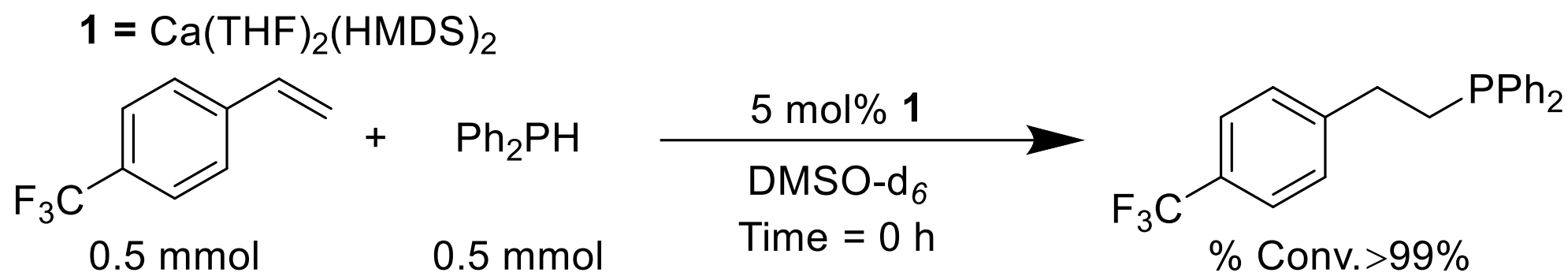

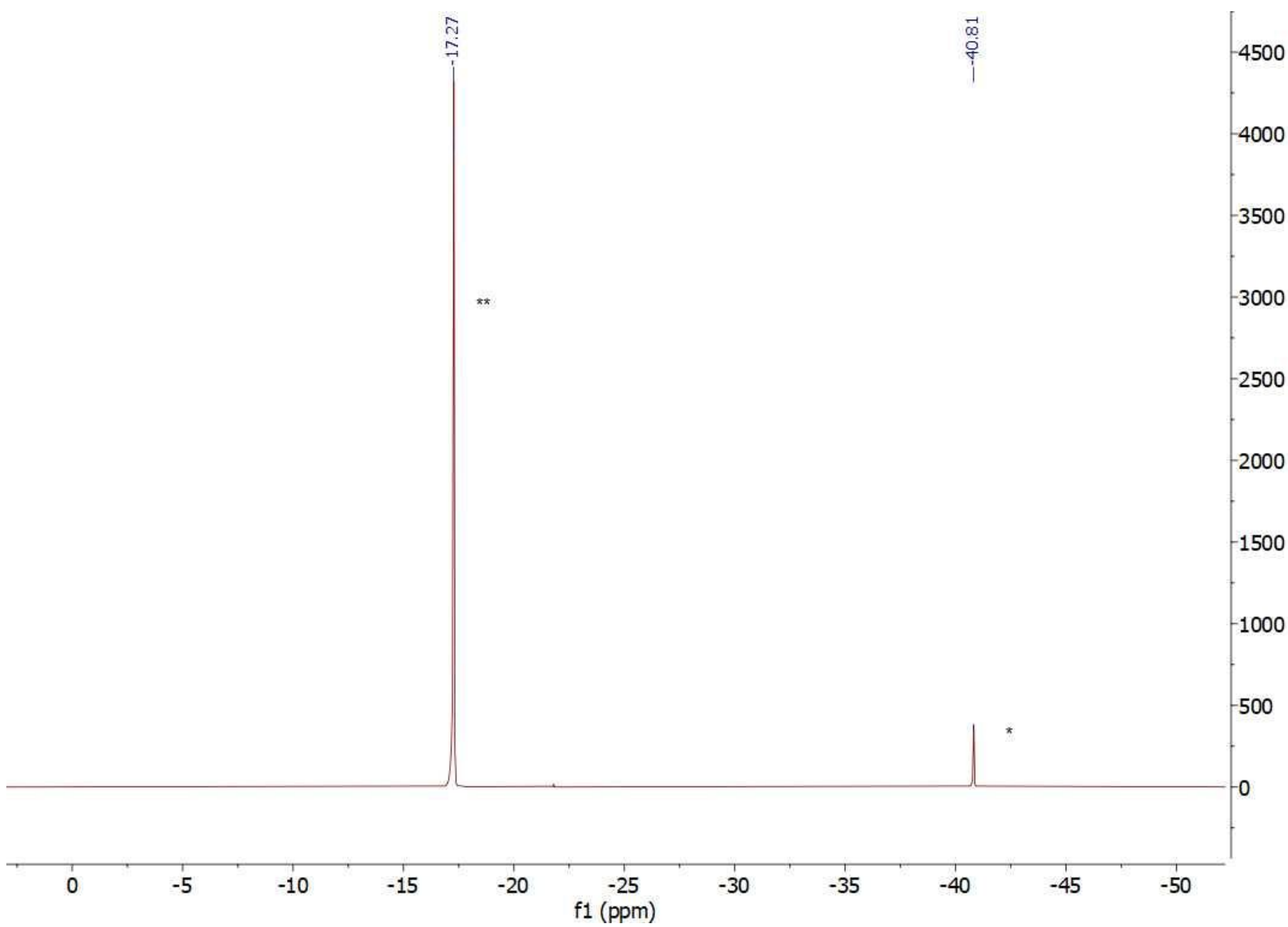

Figure S23: Hydrophosphination of 4-trifluoromethyl styrene with equimolar of Ph_2PH in DMSO-d_6 using 5 mol% **1** under ambient condition- 0 h – $^{31}P\{^1H\}$ NMR

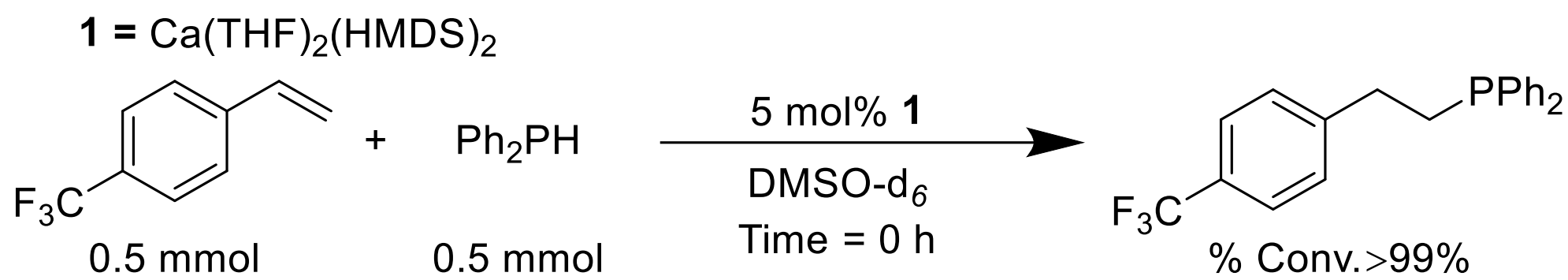

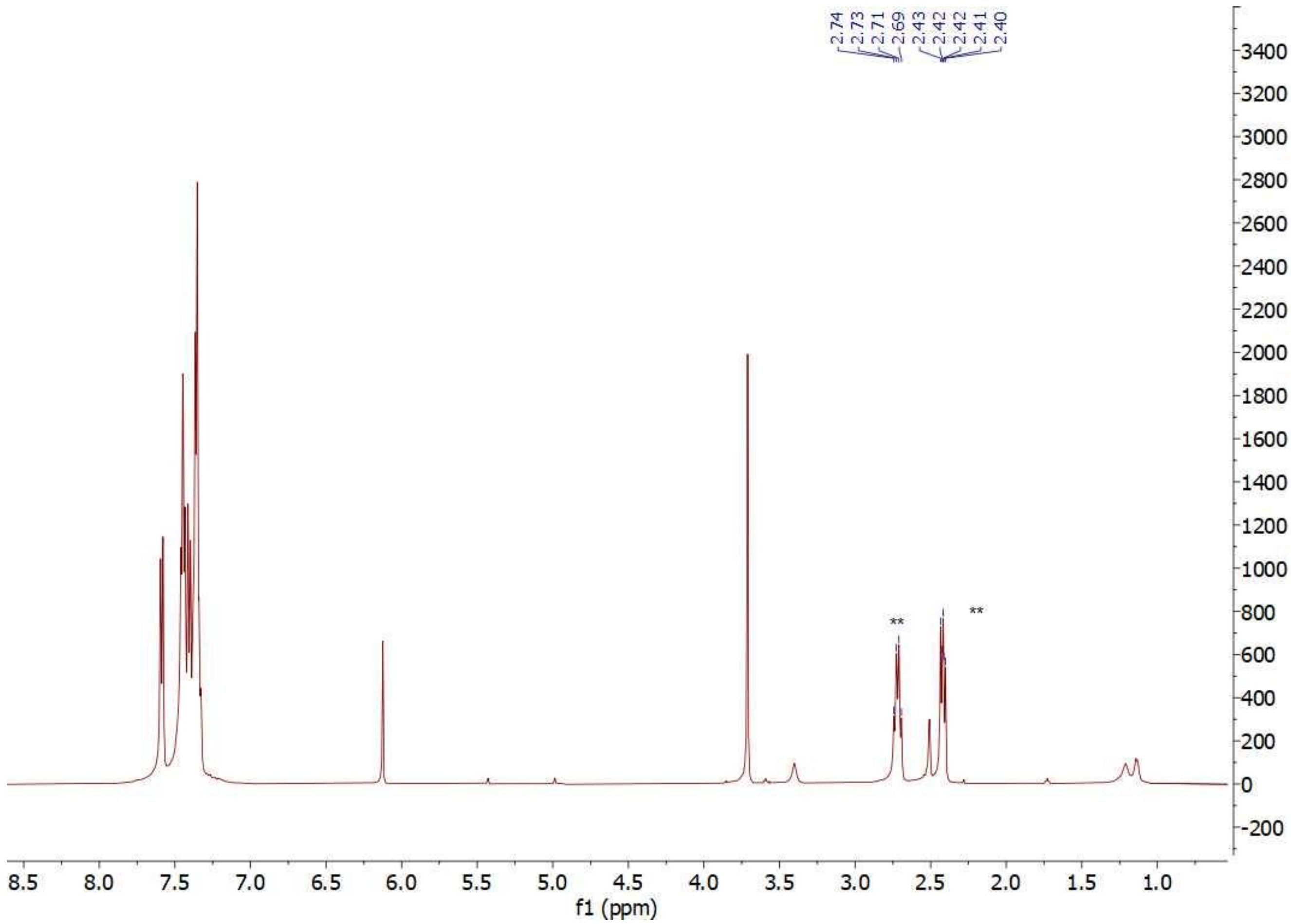

Figure S24: Hydrophosphination of 4-trifluoromethyl styrene with equimolar of Ph_2PH in DMSO-d_6 using 5 mol% **1** under ambient condition - 0 h – ^{1}H NMR

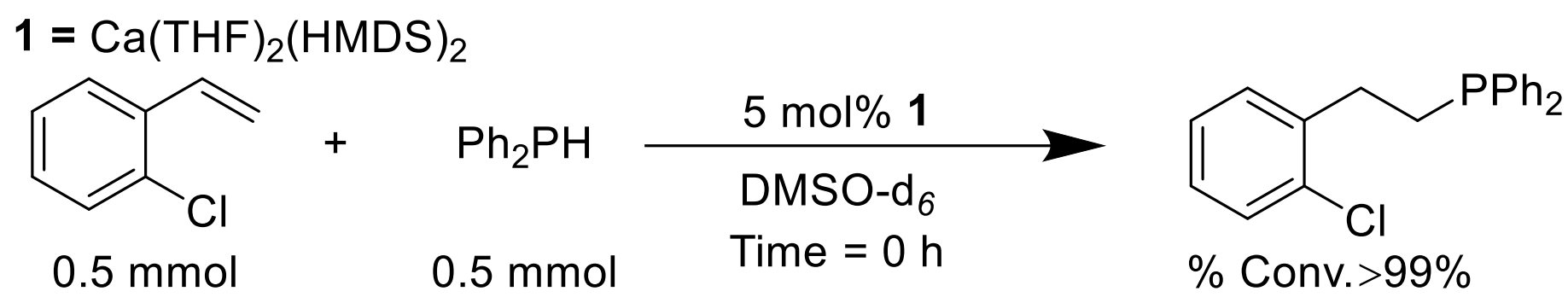

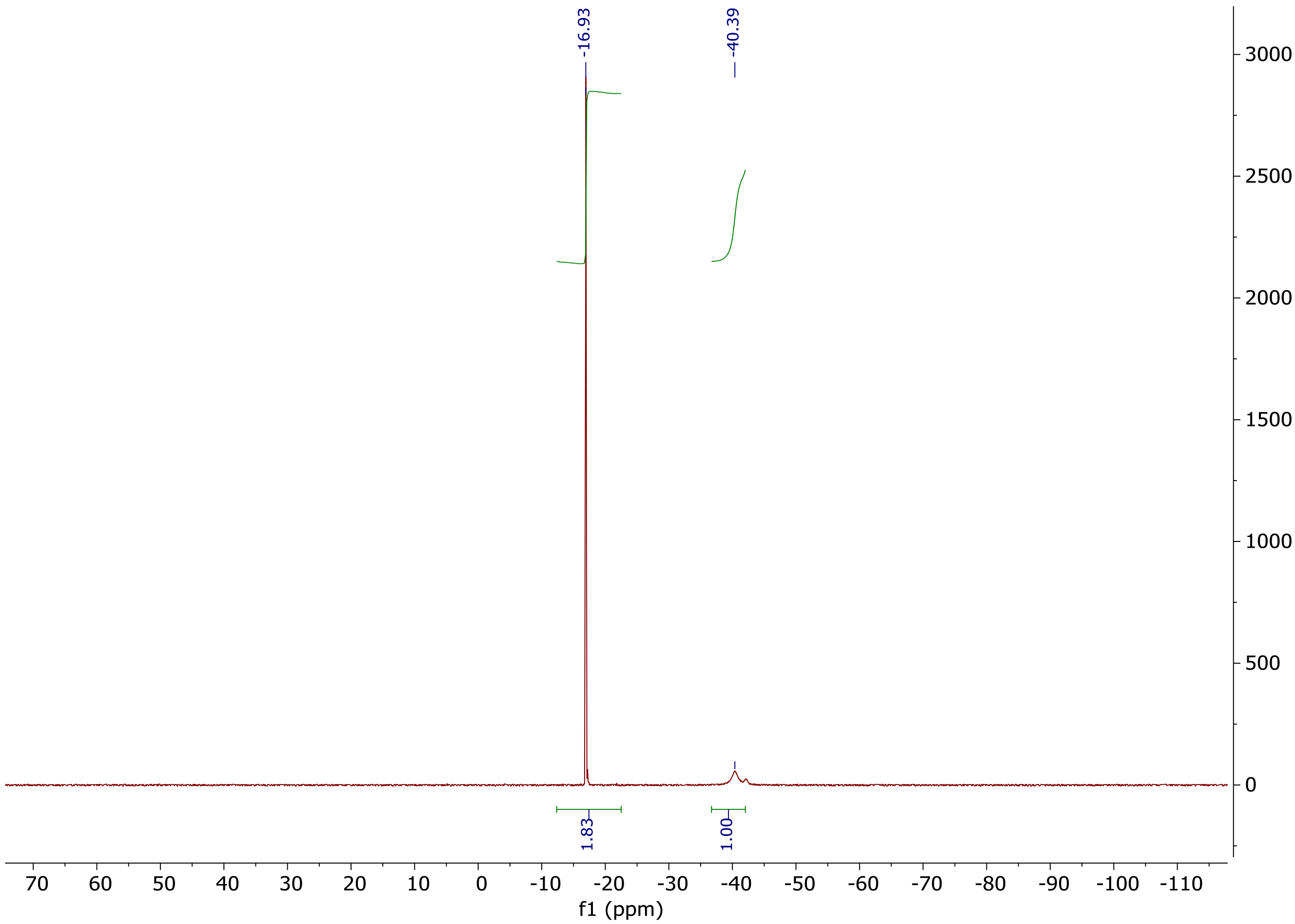

Figure S25: Hydrophosphination of 2-chloro styrene with Ph_2PH in DMSO-d_6 at ambient conditions using 5 mol% **1,** and 1,3,5 – trimethoxybenzene as internal standard – 0 h – $^{31}P\{^1H\}$ NMR

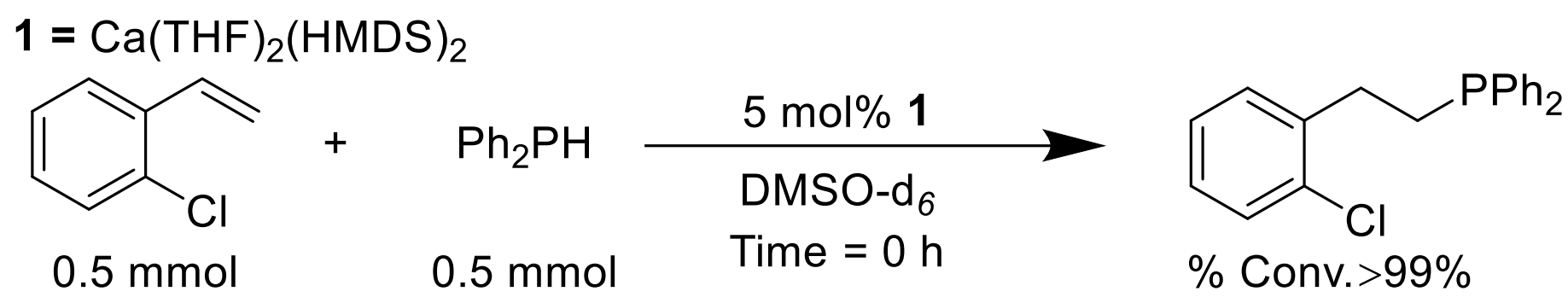

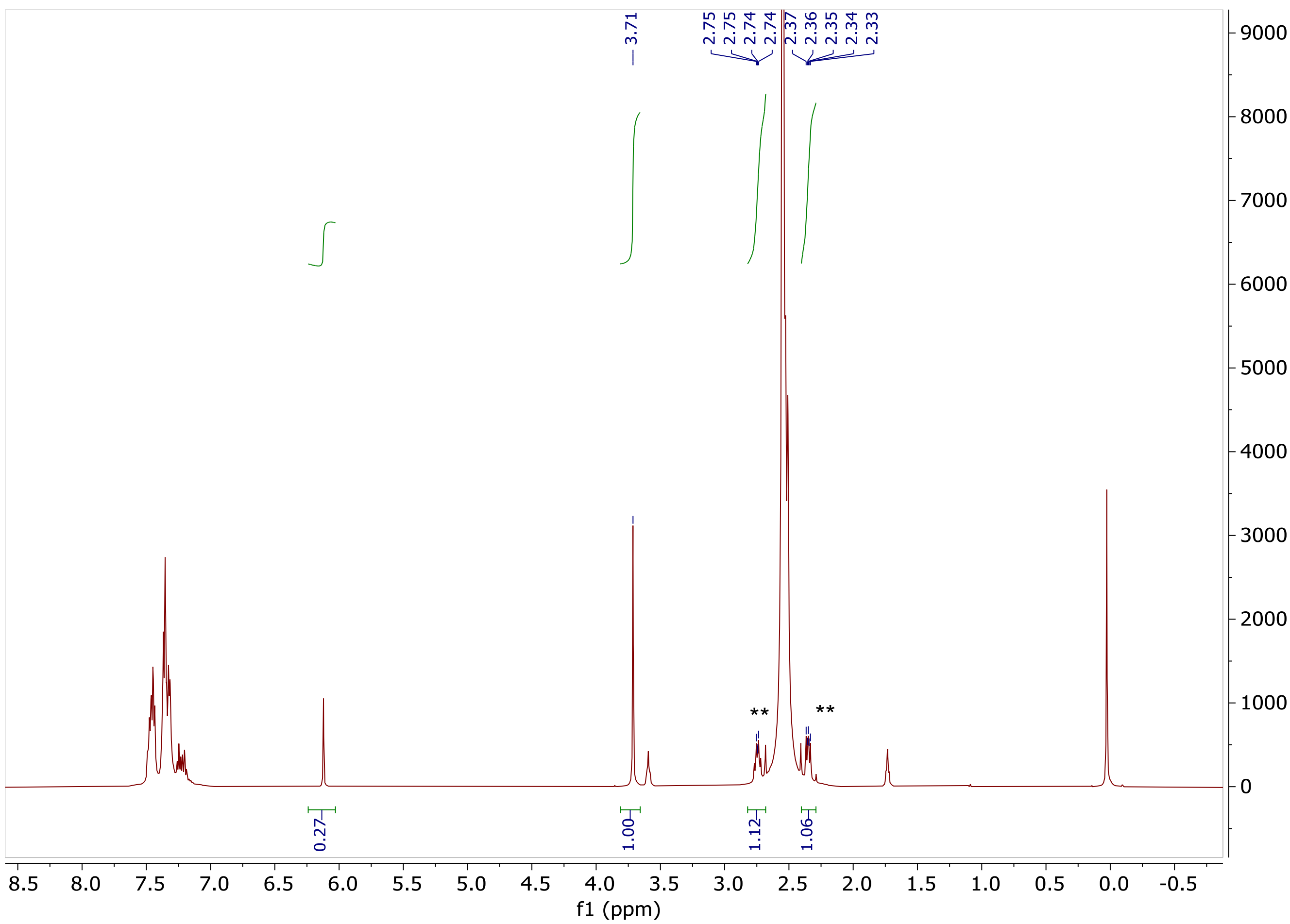

Figure S26: Hydrophosphination of 2-chloro styrene with Ph_2PH in DMSO-d_6 at ambient conditions using 5 mol% **1,** and 1,3,5 – trimethoxybenzene as internal standard – 0 h – ^1H NMR

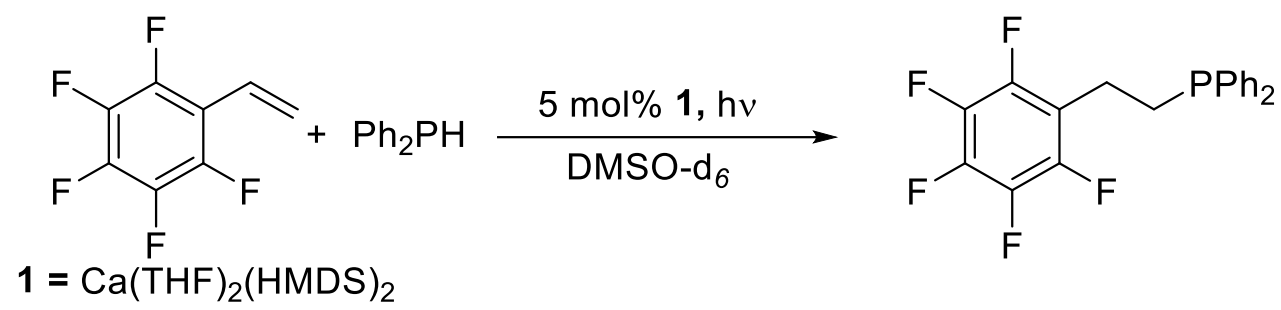

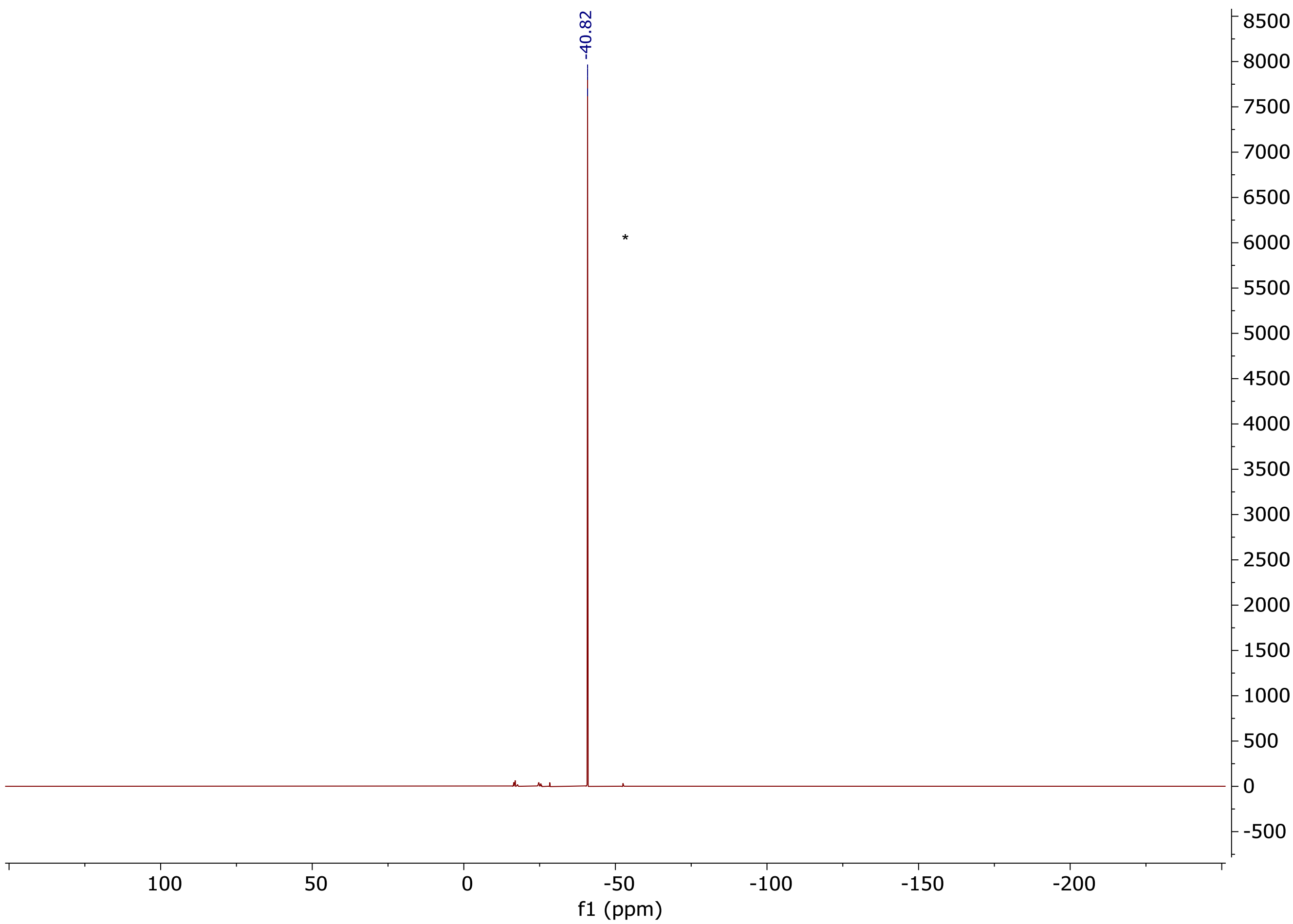

Figure S27: Hydrophosphination of 0.5 mmol 2,3,4,5,6 - pentafluorostyrene with equimolar of Ph_2PH in DMSO-d_6 using 5 mol% **1** under 360 nm UV light – 0 h – $^{31}P\{^1H\}$ NMR

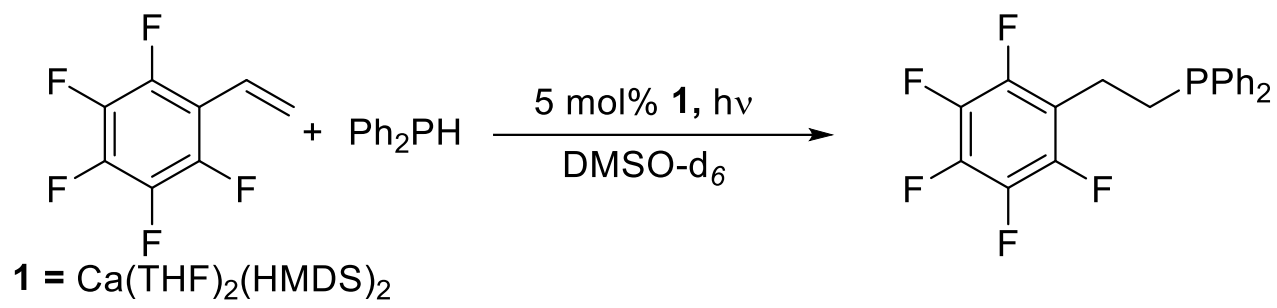

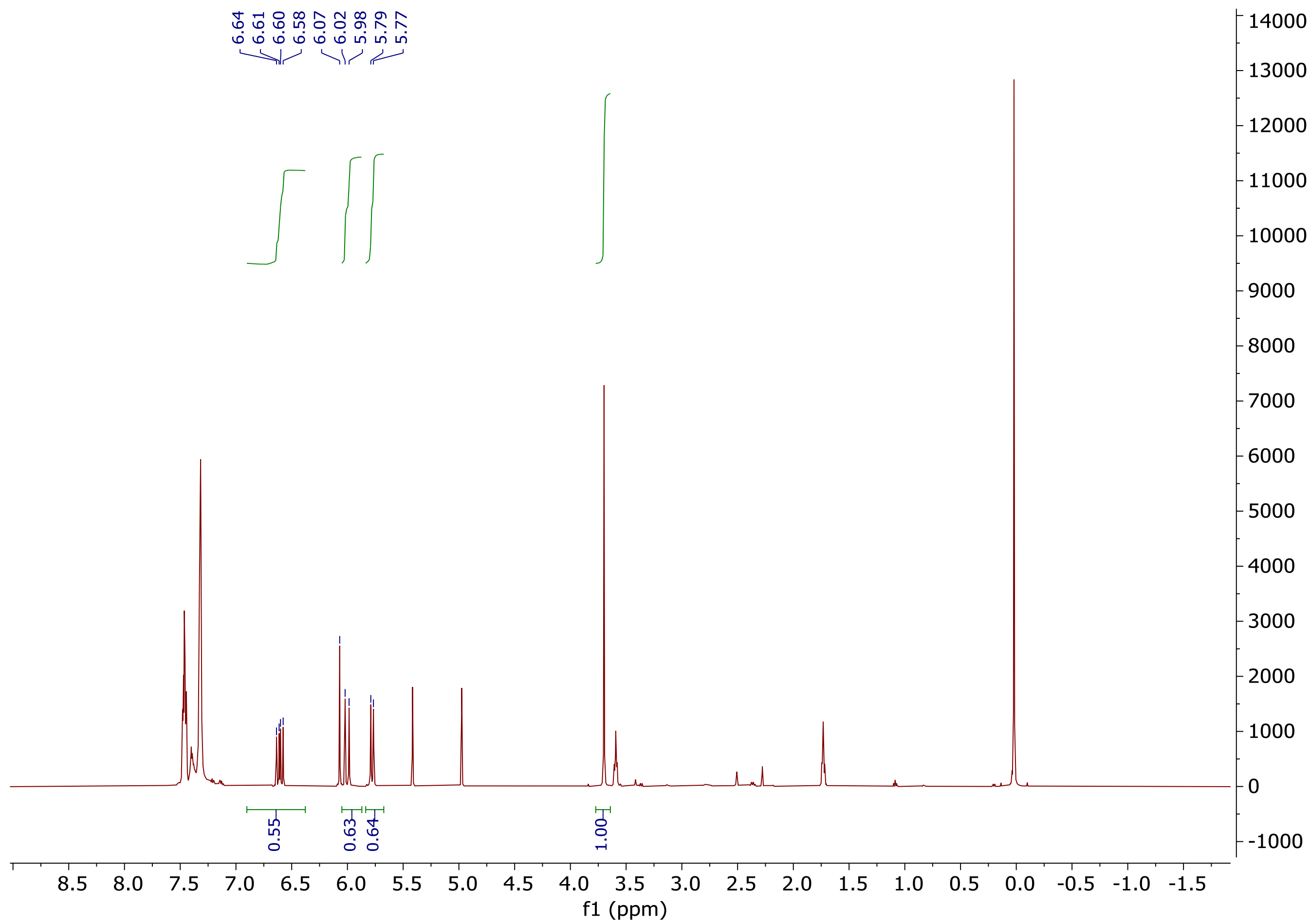

Figure S28: Hydrophosphination of 0.5 mmol 2,3,4,5,6 - pentafluorostyrene with equimolar of Ph_2PH in DMSO-d_6 using 5 mol% **1** under 360 nm UV light – 0 h – ^1H NMR

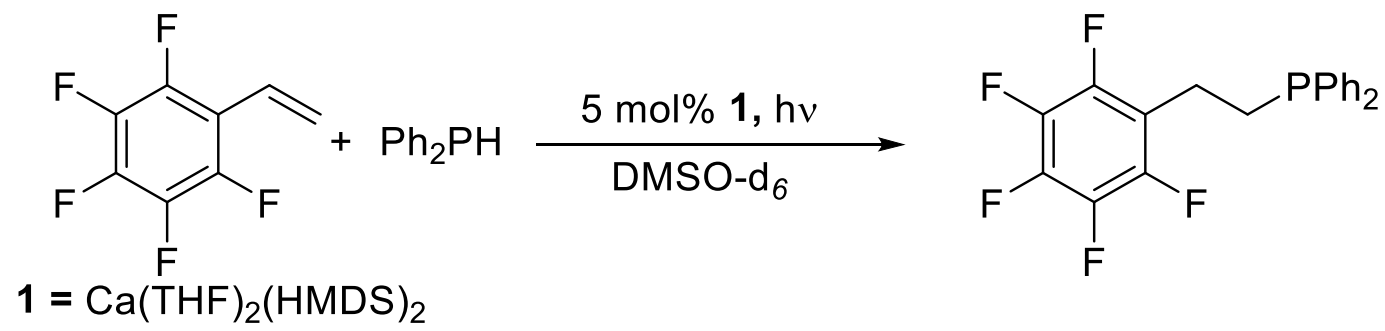

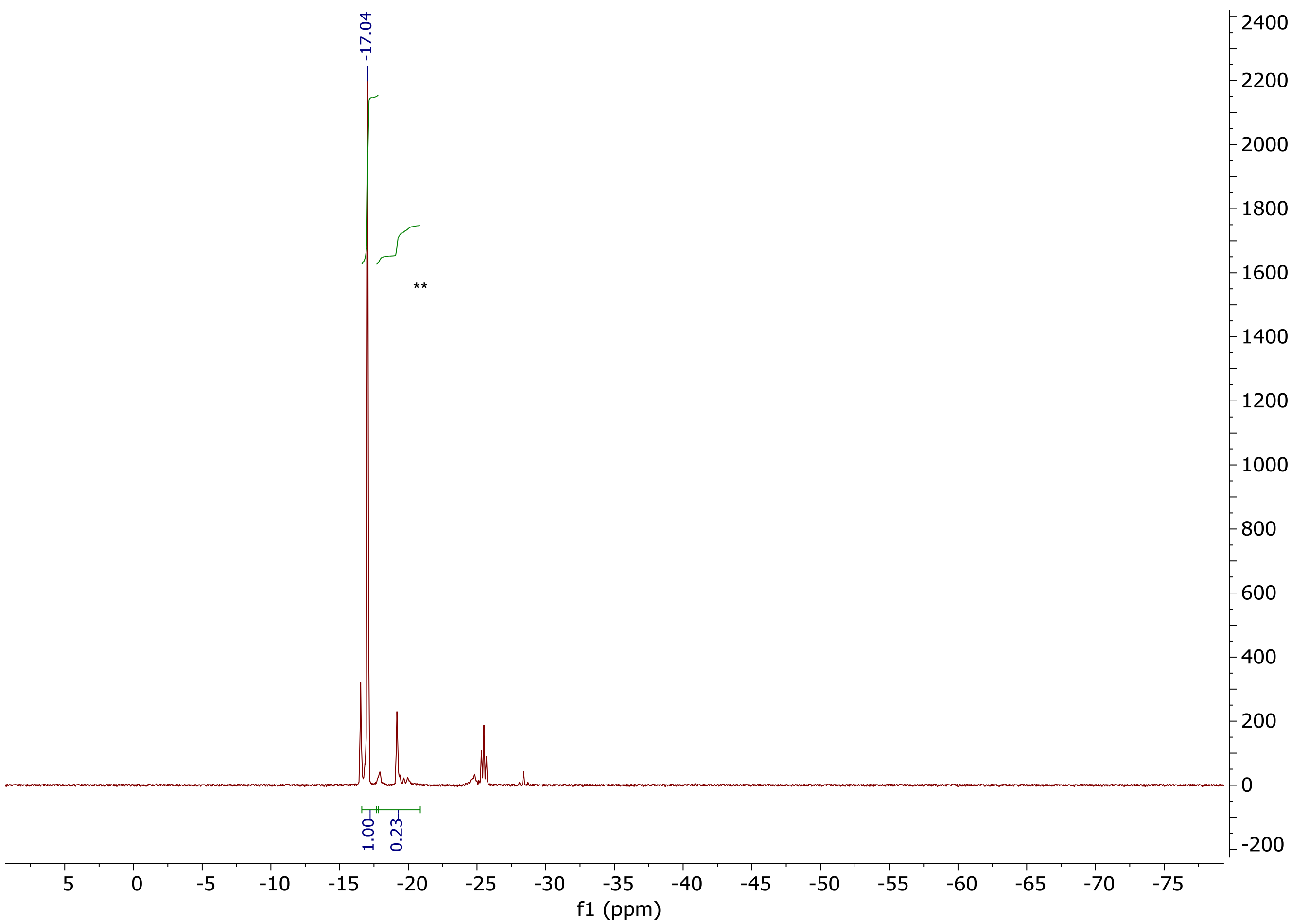

Figure S29: Hydrophosphination of 0.5 mmol 2,3,4,5,6 - pentafluorostyrene with equimolar of Ph_2PH in DMSO-d_6 using 5 mol% **1** under 360 nm UV light – 4 h – $^{31}P\{^1H\}$ NMR

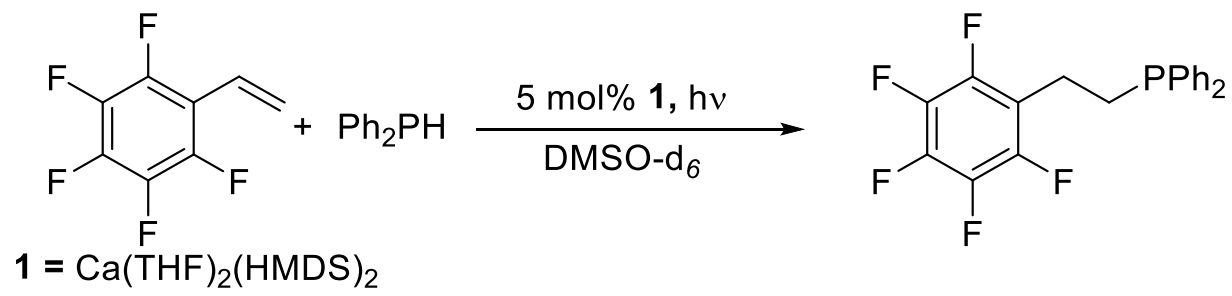

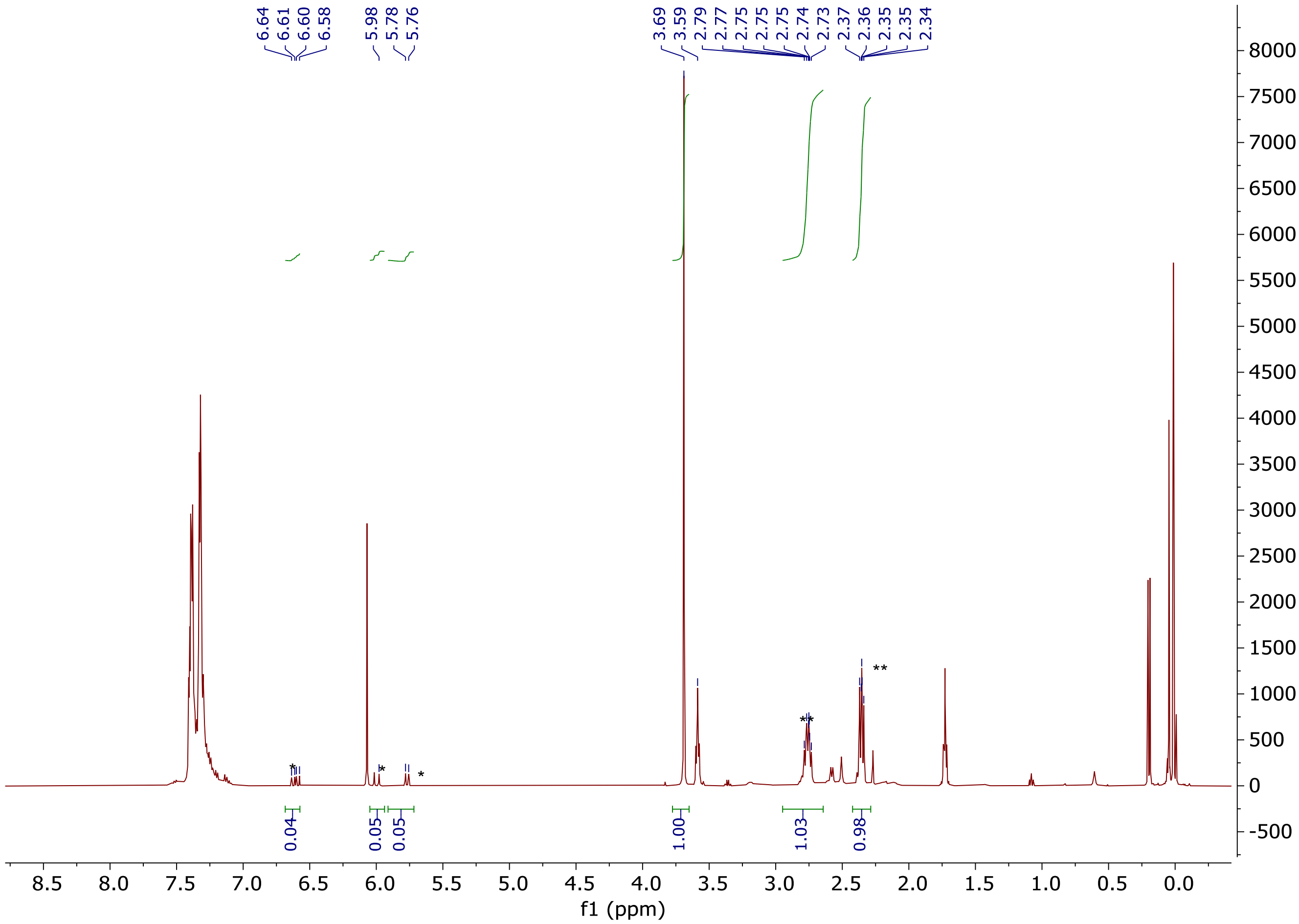

Figure S30: Hydrophosphination of 0.5 mmol 2,3,4,5,6 - pentafluorostyrene with equimolar of Ph_2PH in DMSO-d_6 using 5 mol% **1** under 360 nm UV light – 4 h – ^1H NMR

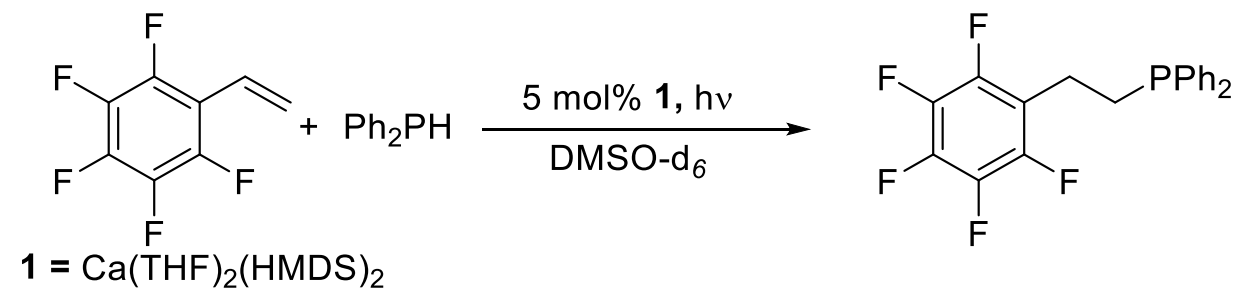

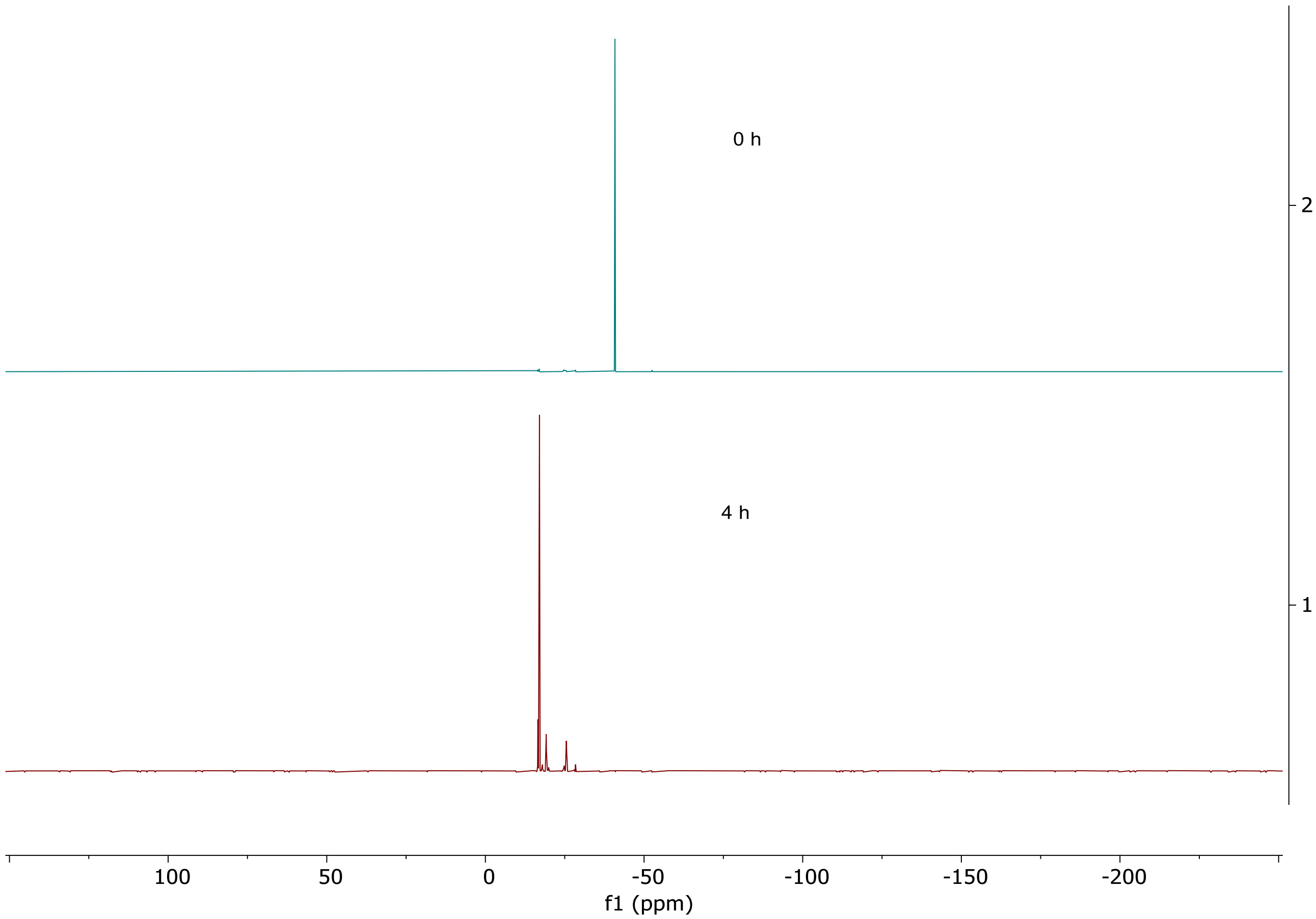

Figure S31: Hydrophosphination of 0.5 mmol 2,3,4,5,6 - pentafluorostyrene with equimolar of Ph_2PH in DMSO-d_6 using 5 mol% **1** under 360 nm UV light – stacked $^{31}P\{^1H\}$ NMR

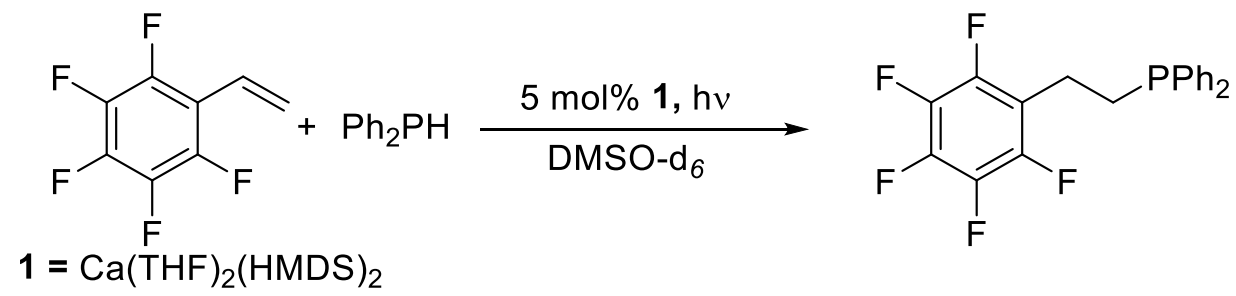

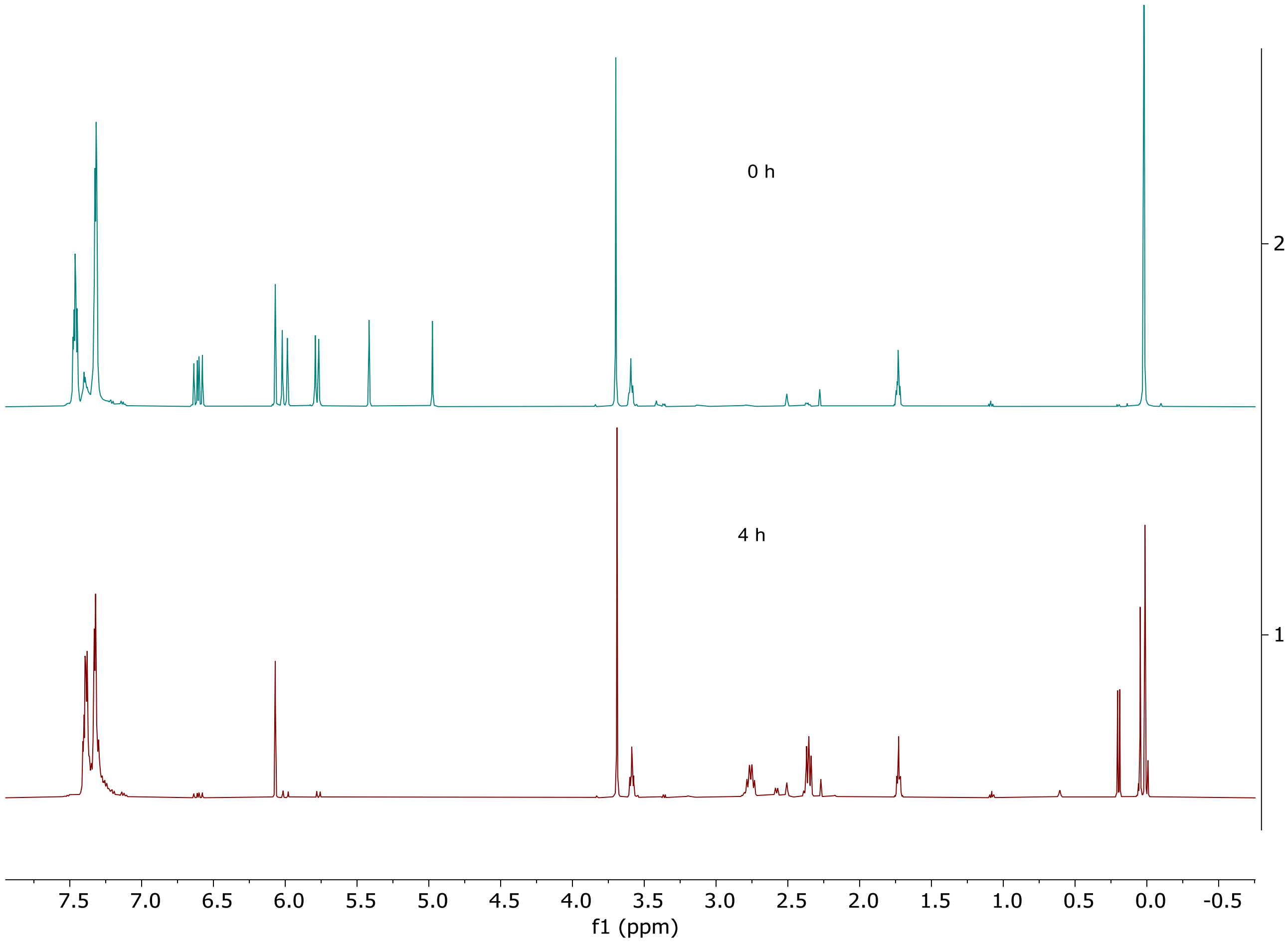

Figure S32: Hydrophosphination of 0.5 mmol 2,3,4,5,6 - pentafluorostyrene with equimolar of Ph_2PH in DMSO-d_6 using 5 mol% **1** under 360 nm UV light – stacked ^1H NMR

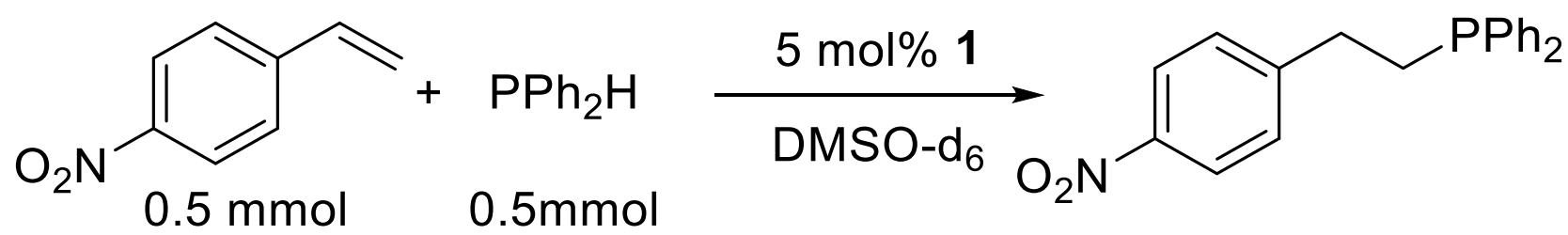

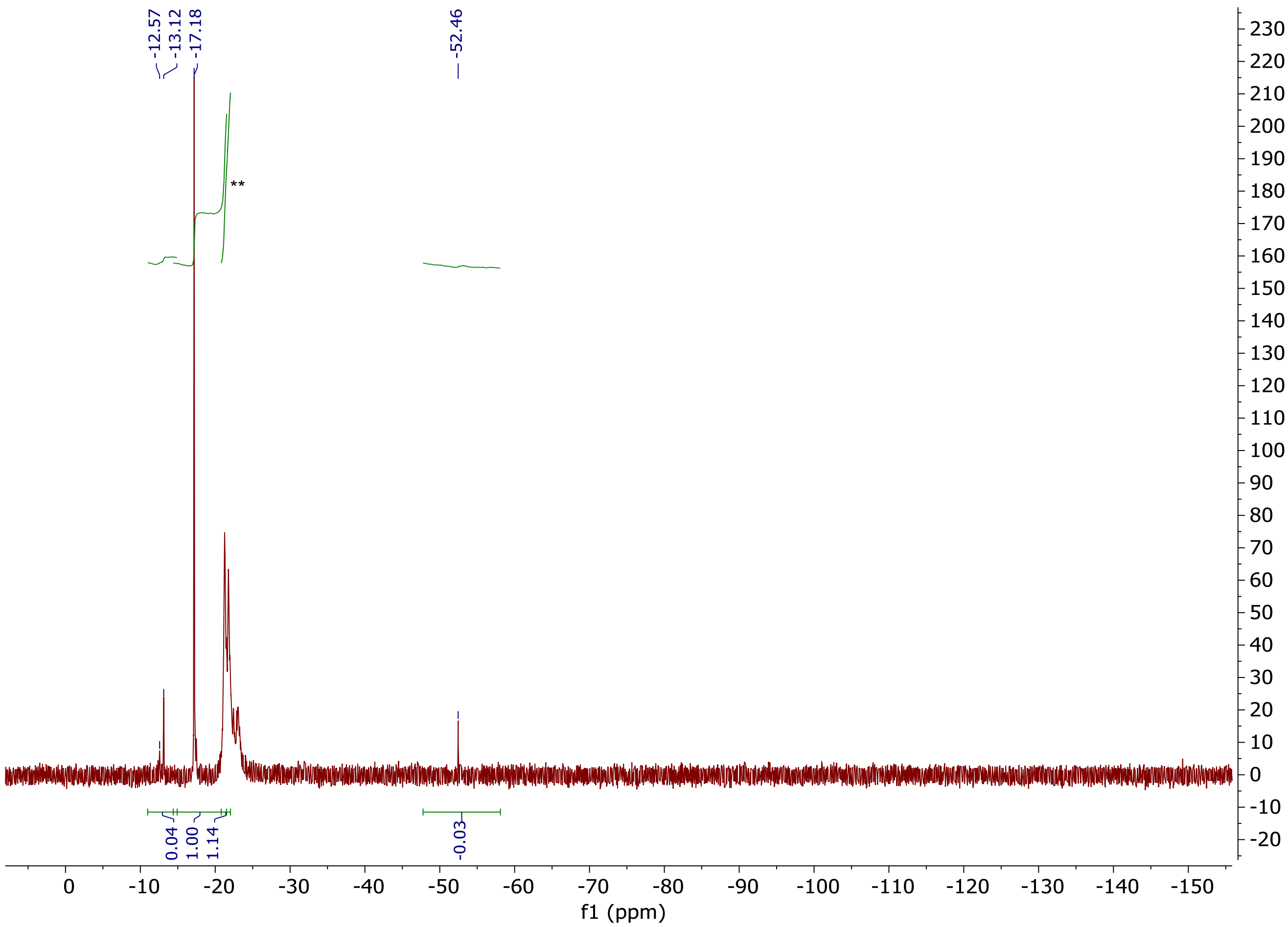

Figure S33: Hydrophosphination of 0.5 mmol 4-nitrostyrene with equimolar of Ph_2PH in DMSO-d_6 using 5 mol% **1** under 360 nm UV light –0 h - $^{31}P\{^1H\}$ NMR

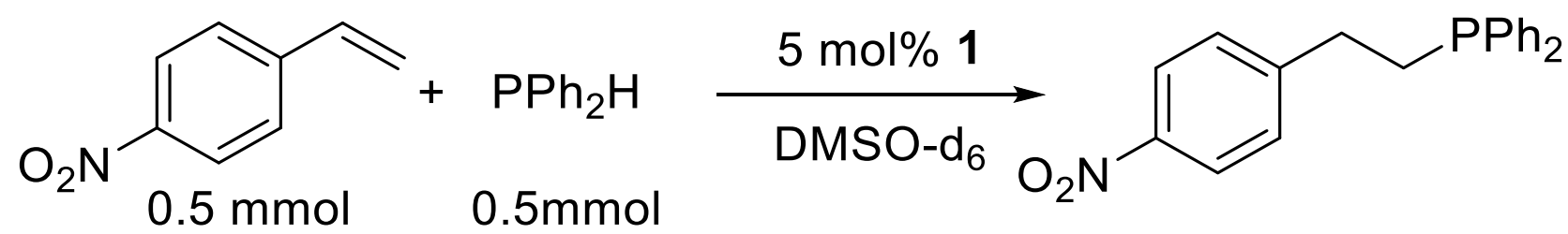

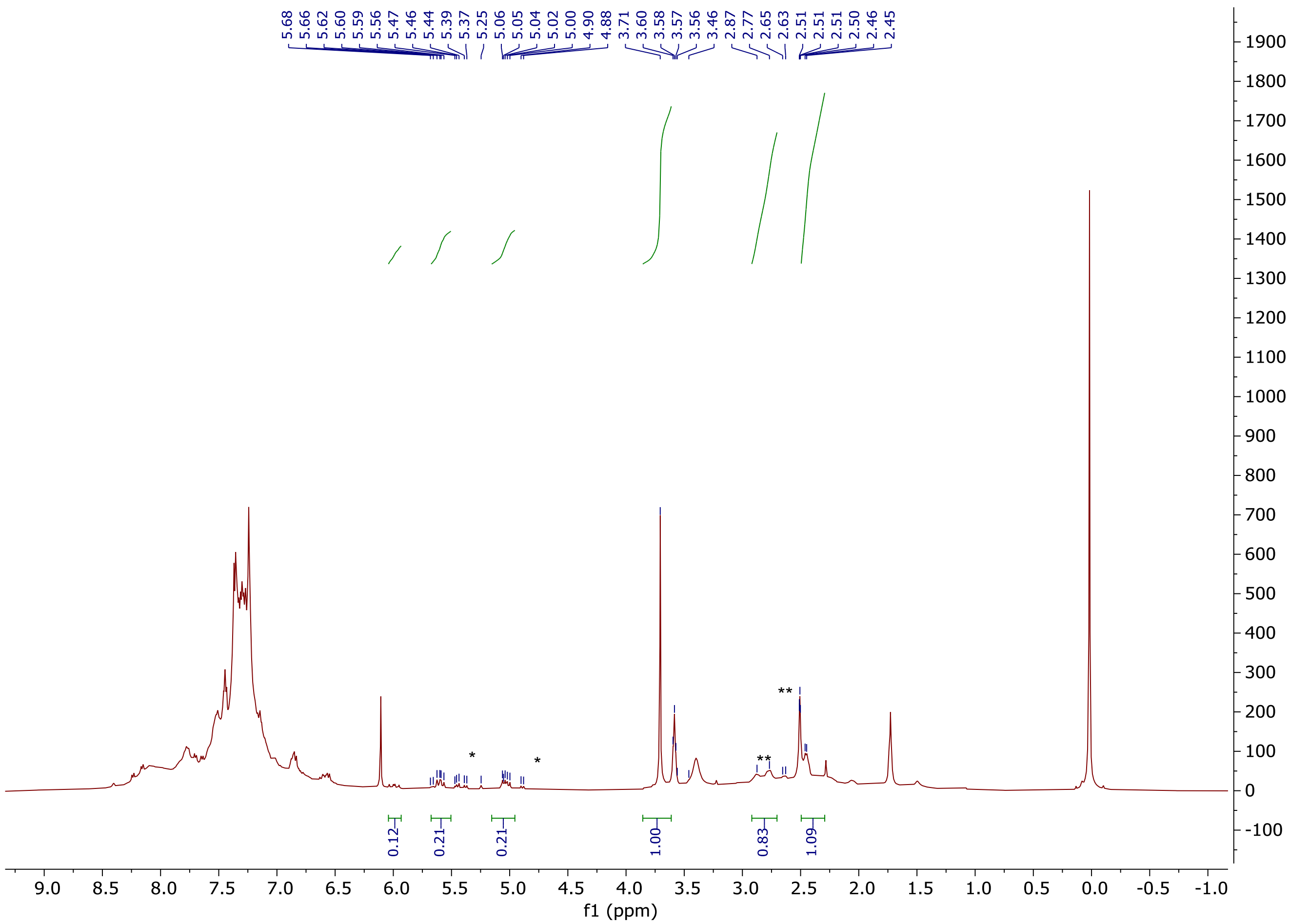

Figure S34: Hydrophosphination of 0.5 mmol 4-nitrostyrene with equimolar of Ph_2PH in DMSO-d_6 using 5 mol% **1** under 360 nm UV light – 0 h - ^{1}H NMR

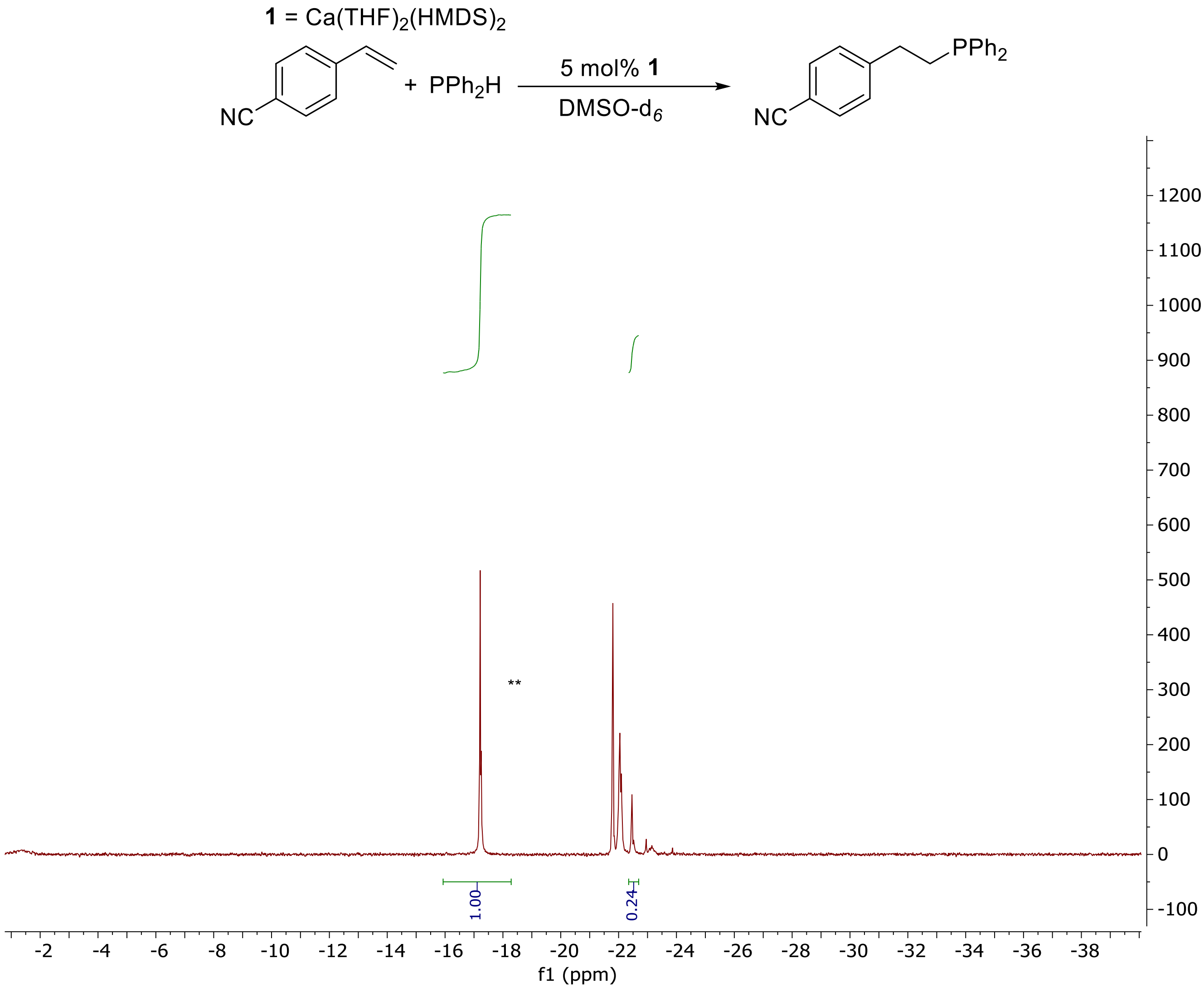

Figure S35: Hydrophosphination of 0.5 mmol 4-cyanostyrene with equimolar Ph_2PH in DMSO-d_6 using 5 mol% **1** under 360 nm UV light – 0 h - $^{31}P\{^1H\}$ NMR

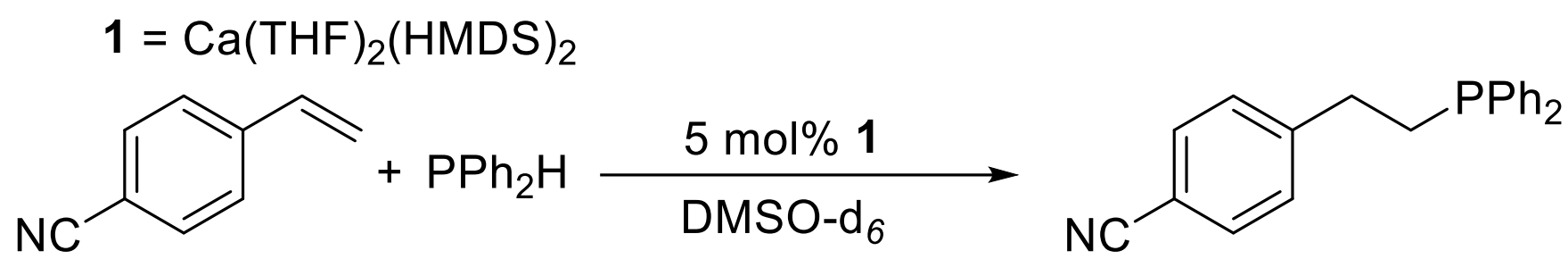

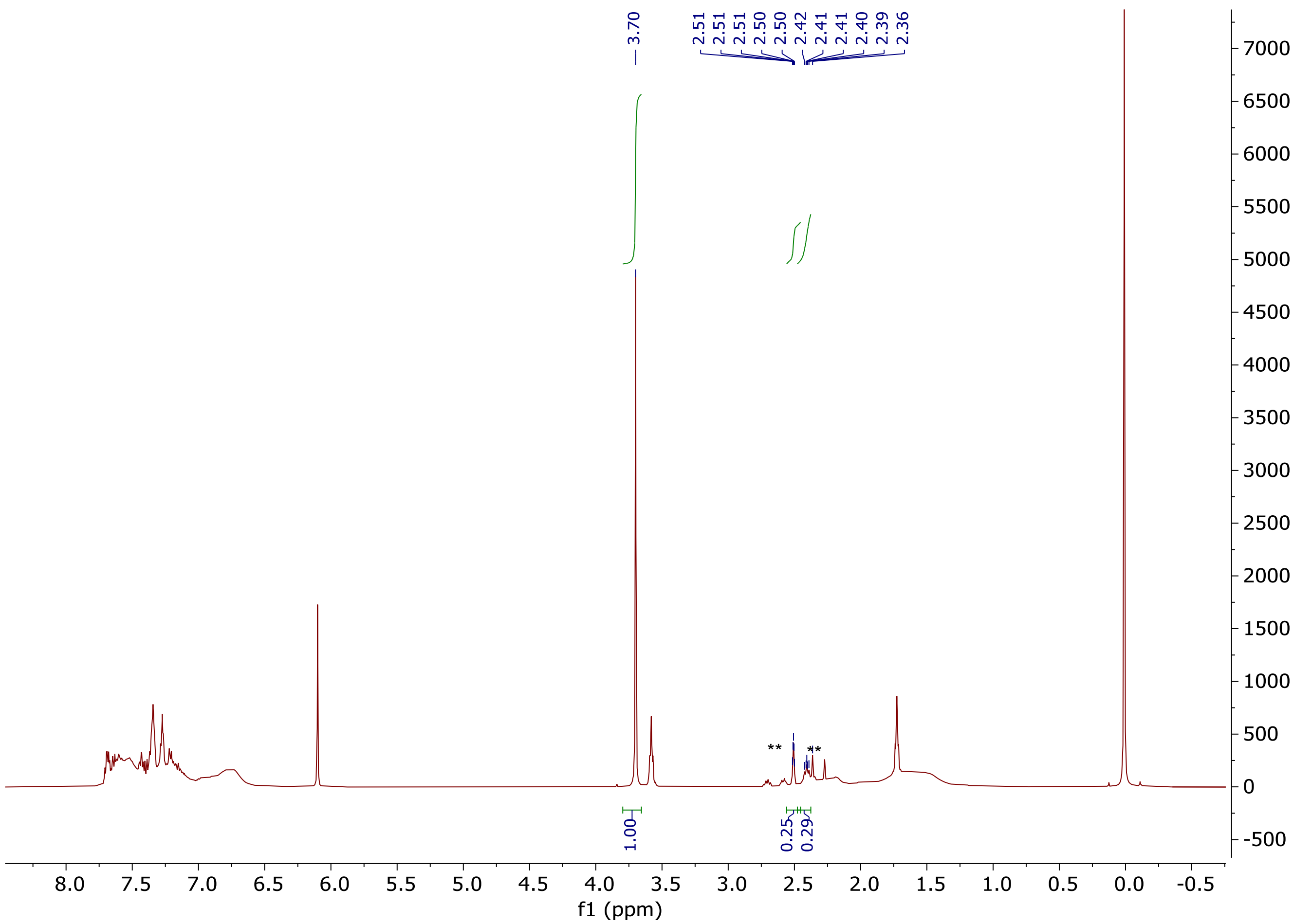

Figure S36: Hydrophosphination of 0.5 mmol 4-cyanostyrene with equimolar of Ph_2PH in DMSO-d_6 using 5 mol% **1** under 360 nm UV light – 0 h - ^1H NMR

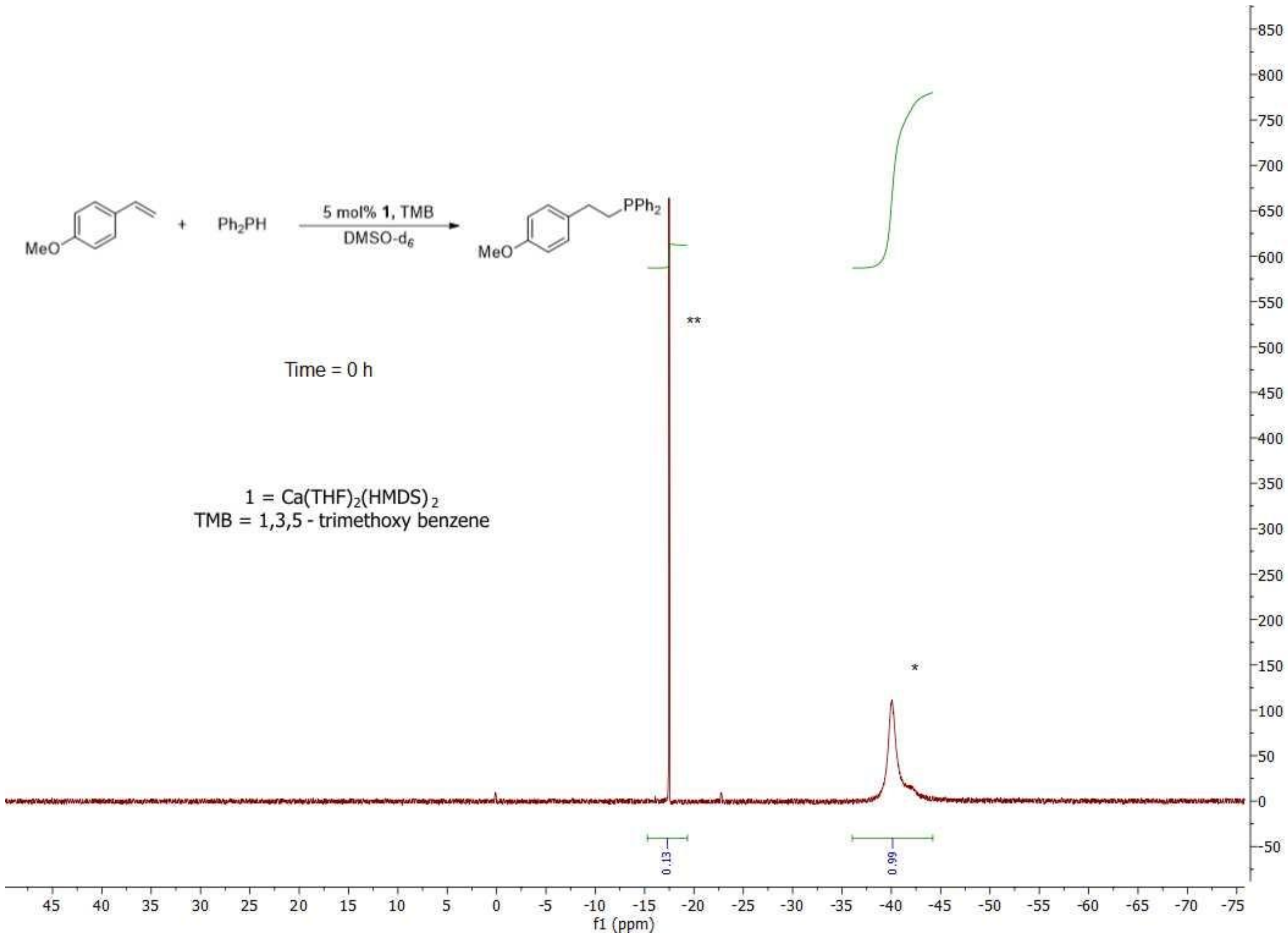

Figure S37: Hydrophosphination of 0.5 mmol 4-methoxy styrene with equimolar Ph_2PH in DMSO-d_6 using 5 mol% **1** under ambient condition- 0 h – $^{31}P\{^1H\}$ NMR

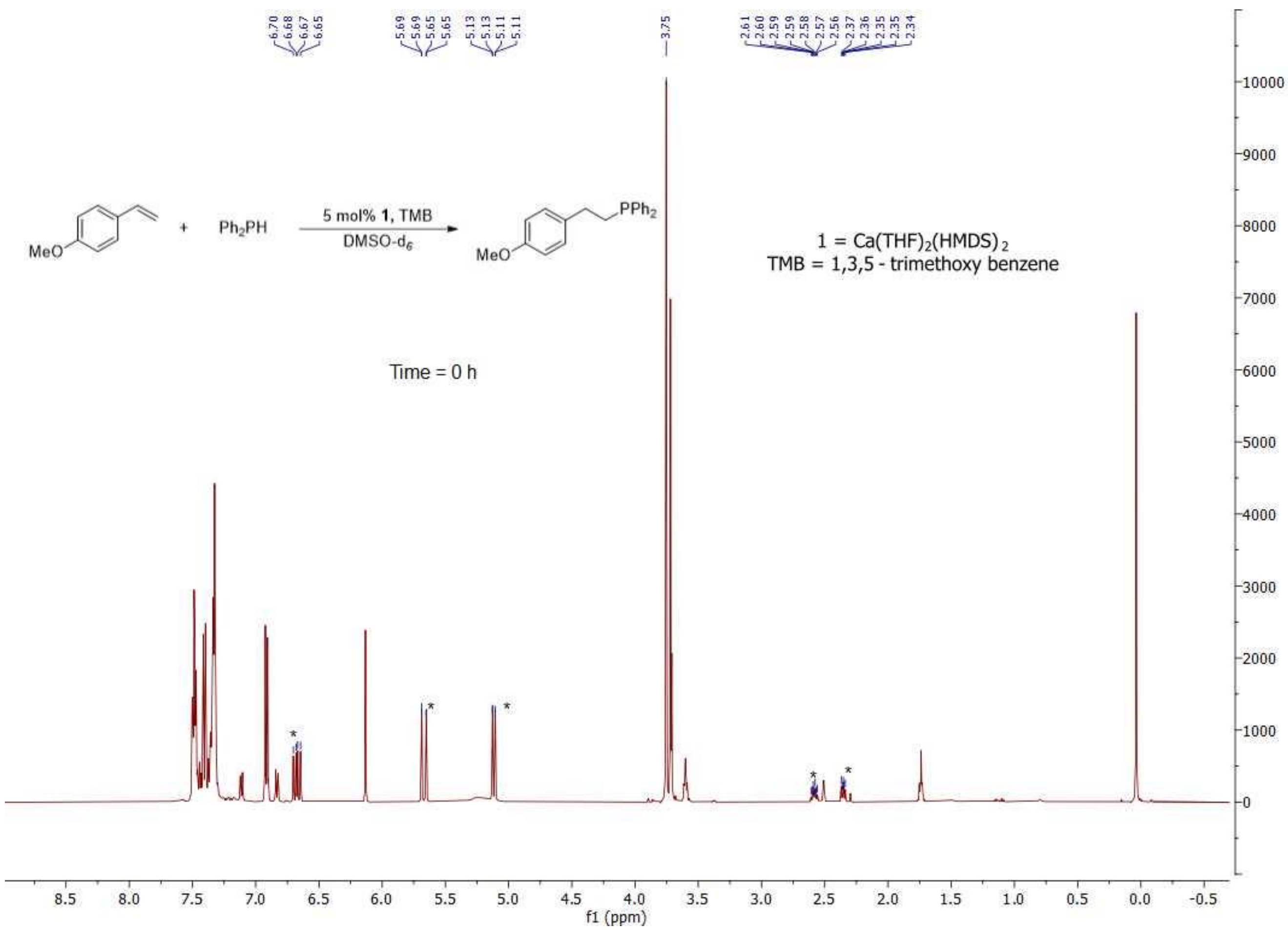

Figure S38: Hydrophosphination of 0.5 mmol 4-methoxy styrene with equimolar of Ph_2PH in DMSO-d_6 using 5 mol% **1** under ambient condition - 0 h – ^{1}H NMR

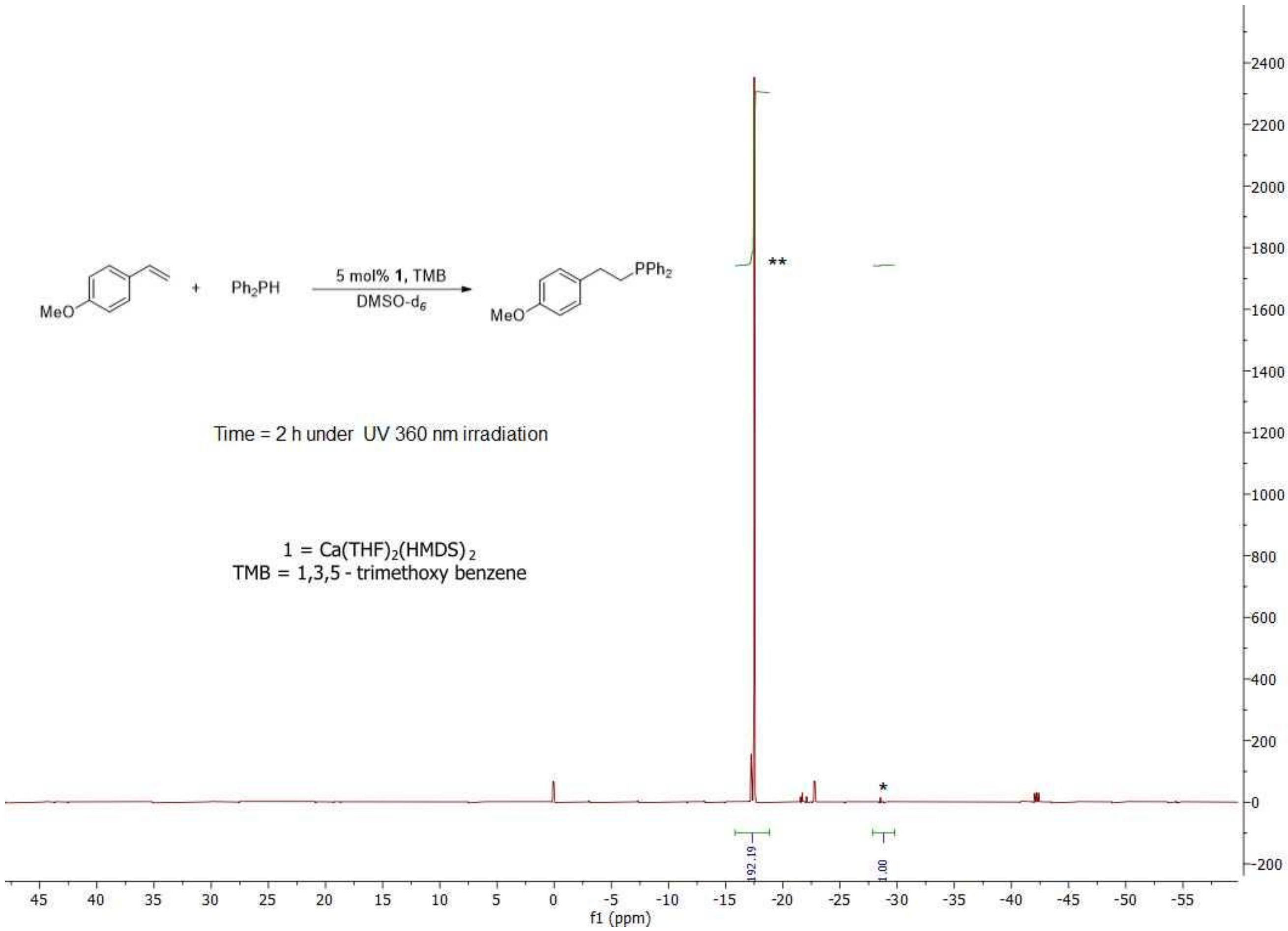

Figure S39: Hydrophosphination of 0.5 mmol 4-methoxy styrene with equimolar of Ph_2PH in DMSO-d_6 using 5 mol% **1** under 360 nm UV light - 2 h – $^{31}P\{^1H\}$ NMR

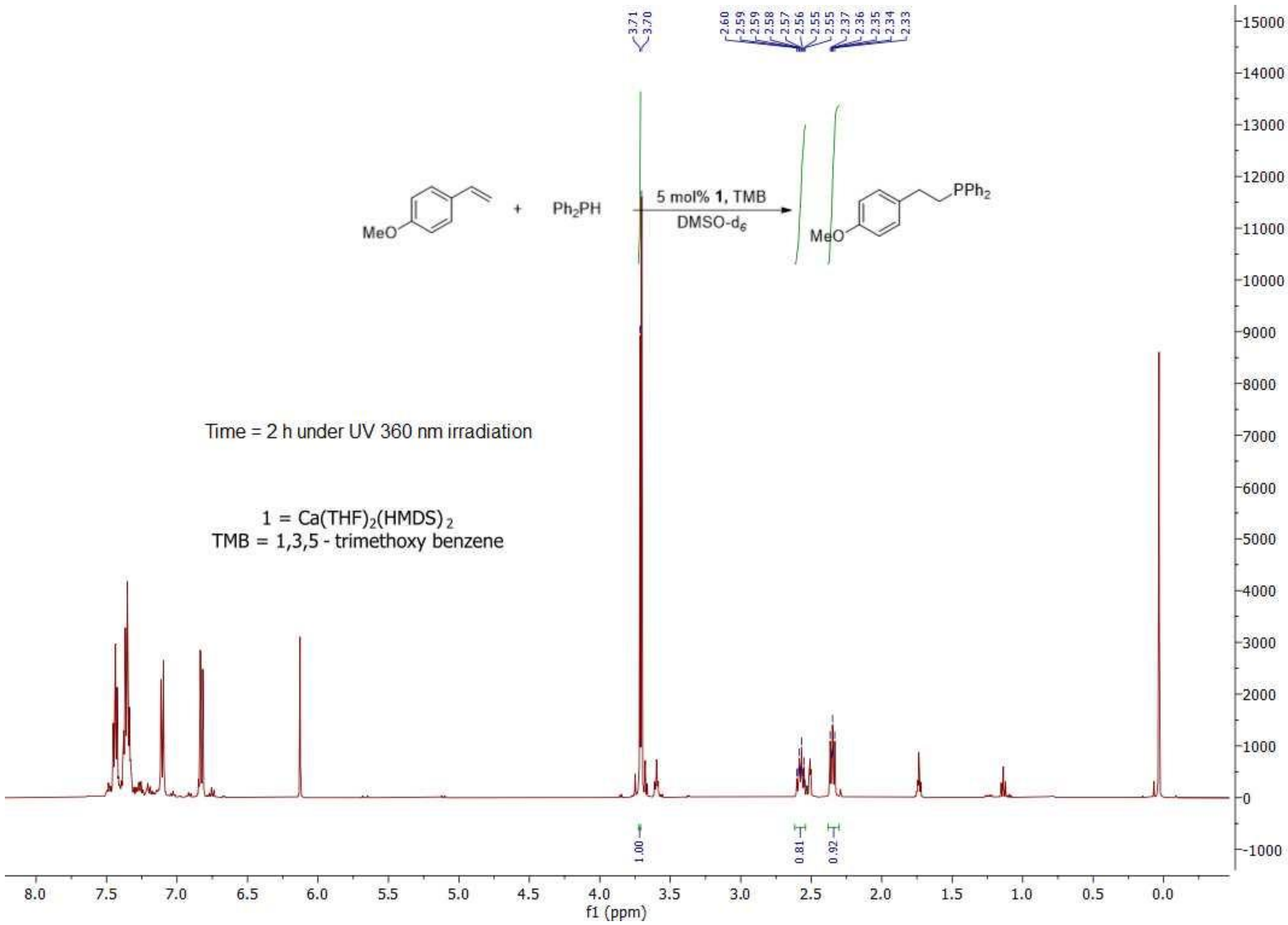

Figure S40: Hydrophosphination of 0.5 mmol 4-methoxy styrene with equimolar of Ph_2PH in DMSO-d_6 using 5 mol% **1** under 360 nm UV light - 2 h – ^{1}H NMR

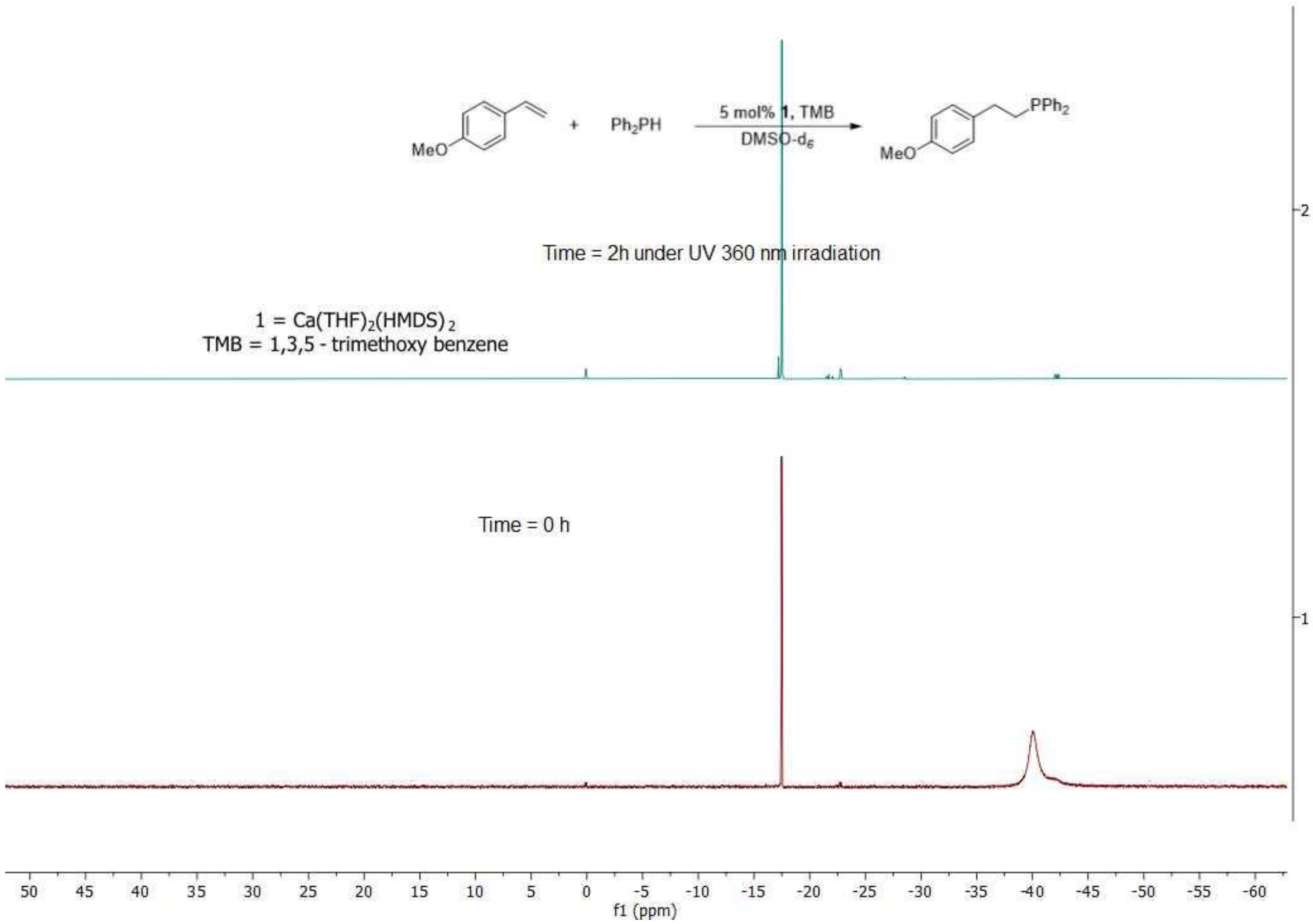

Figure S41: Hydrophosphination of 0.5 mmol 4-methoxy styrene with equimolar of Ph_2PH in DMSO-d_6 using 5 mol% **1** under 360 nm UV light - 2 h – stacked $^{31}P\{^1H\}$ NMR

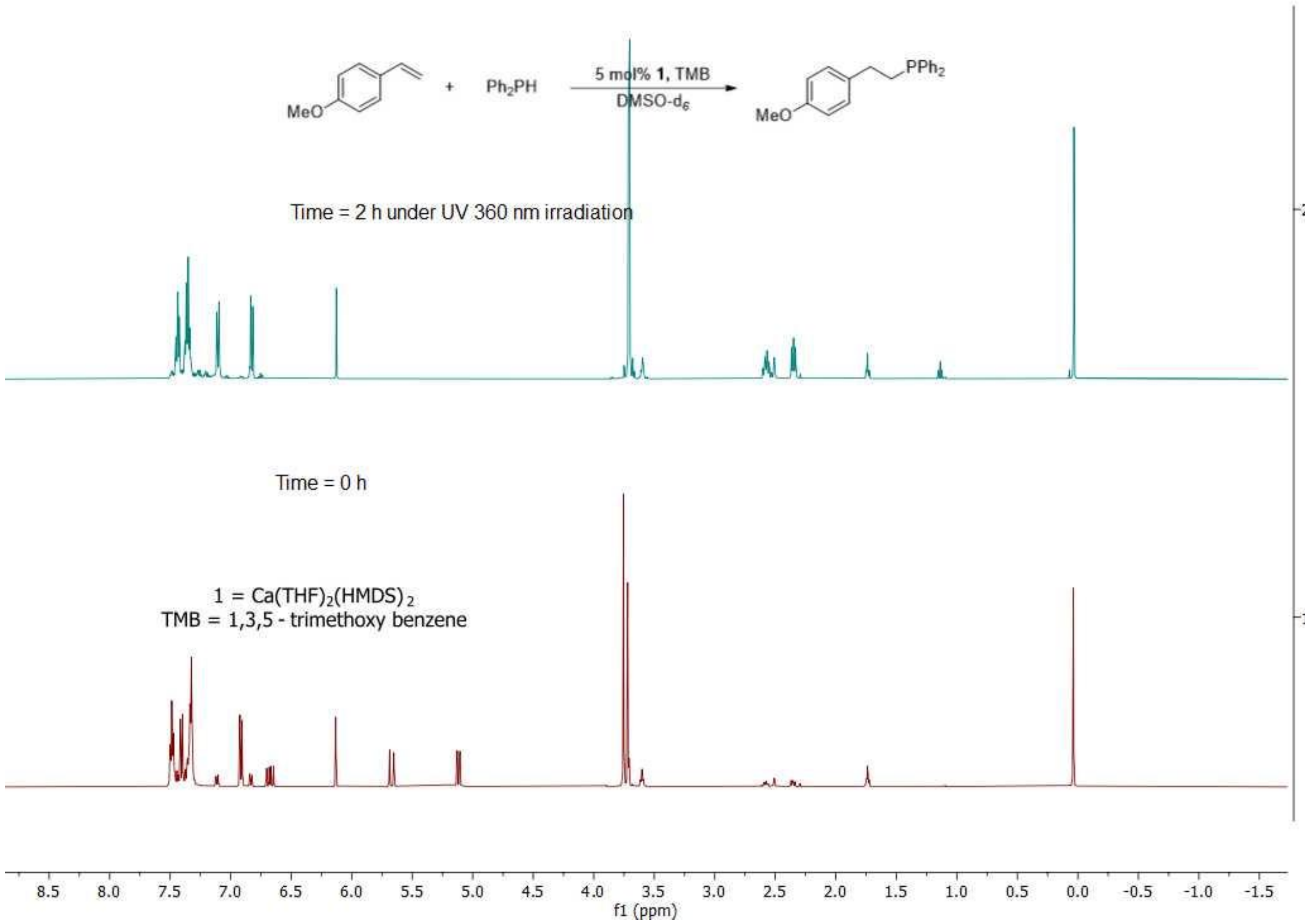

Figure S42: Hydrophosphination of 0.5 mmol 4-methoxy styrene with equimolar of Ph_2PH in DMSO-d_6 using 5 mol% **1** under 360 nm UV light – stacked {^{1}H} NMR

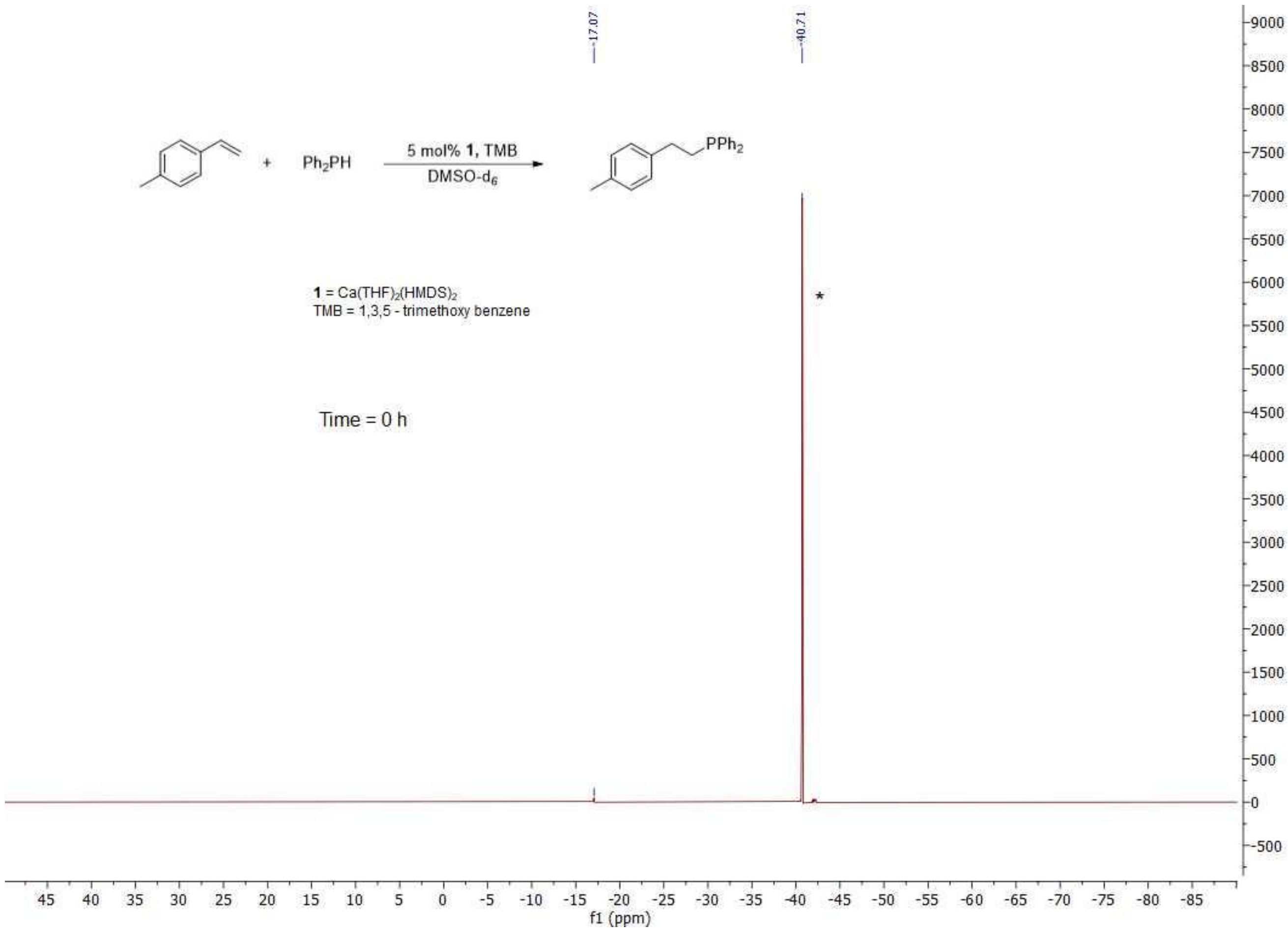

Figure S43: Hydrophosphination of 0.5 mmol 4-methyl styrene with equimolar of Ph_2PH in DMSO-d_6 using 5 mol% **1** under ambient condition - 0 h – $^{31}P\{^1H\}$ NMR

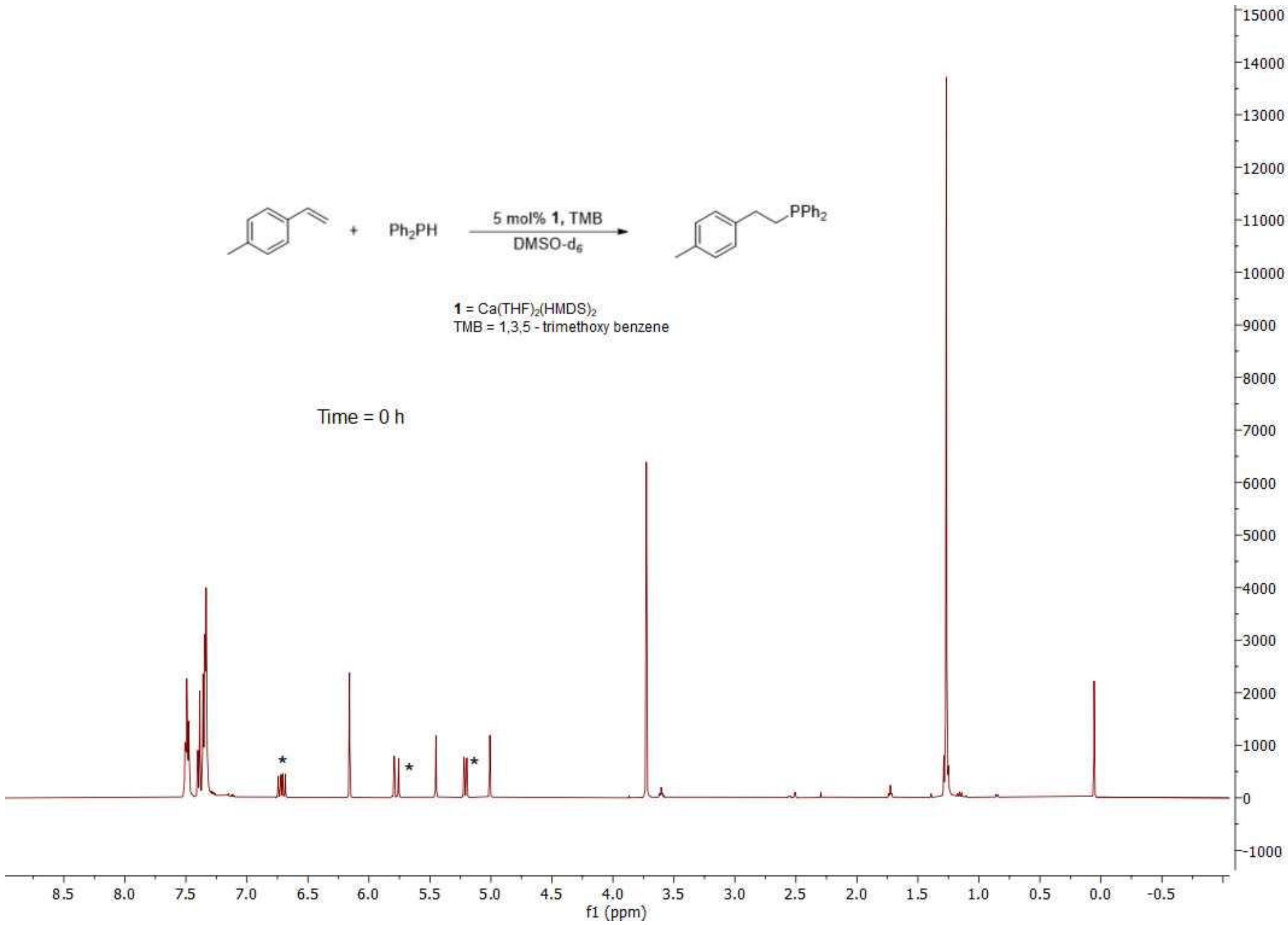

Figure S44: Hydrophosphination of 0.5 mmol 4-methyl styrene with equimolar of Ph_2PH in DMSO-d_6 using 5 mol% **1** under ambient condition - 0 h – ^{1}H NMR

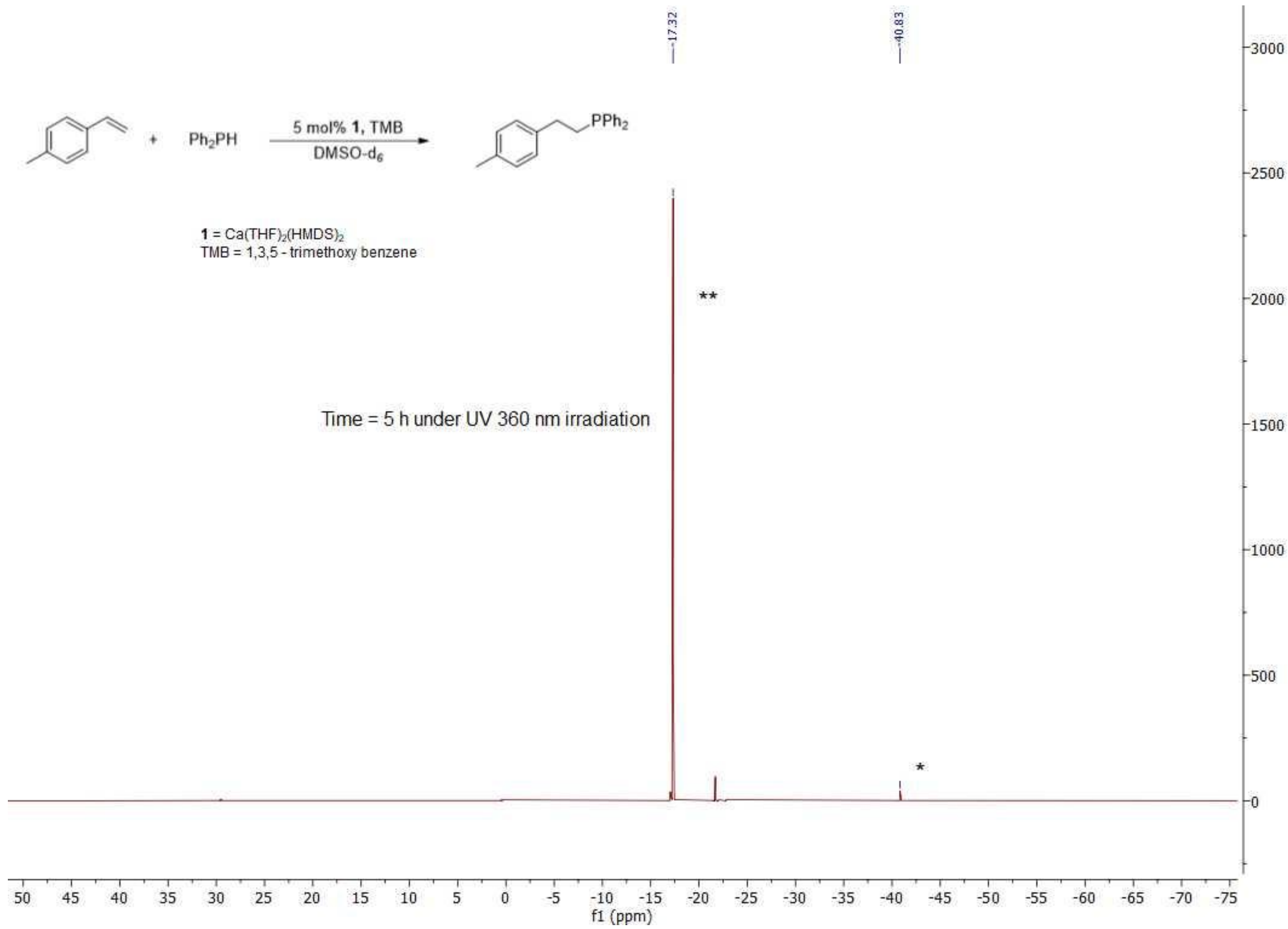

Figure S45: Hydrophosphination of 0.5 mmol 4-methyl styrene with equimolar of Ph_2PH in DMSO-d_6 using 5 mol% **1** under 360 nm UV light - 5 h – $^{31}P\{^1H\}$ NMR

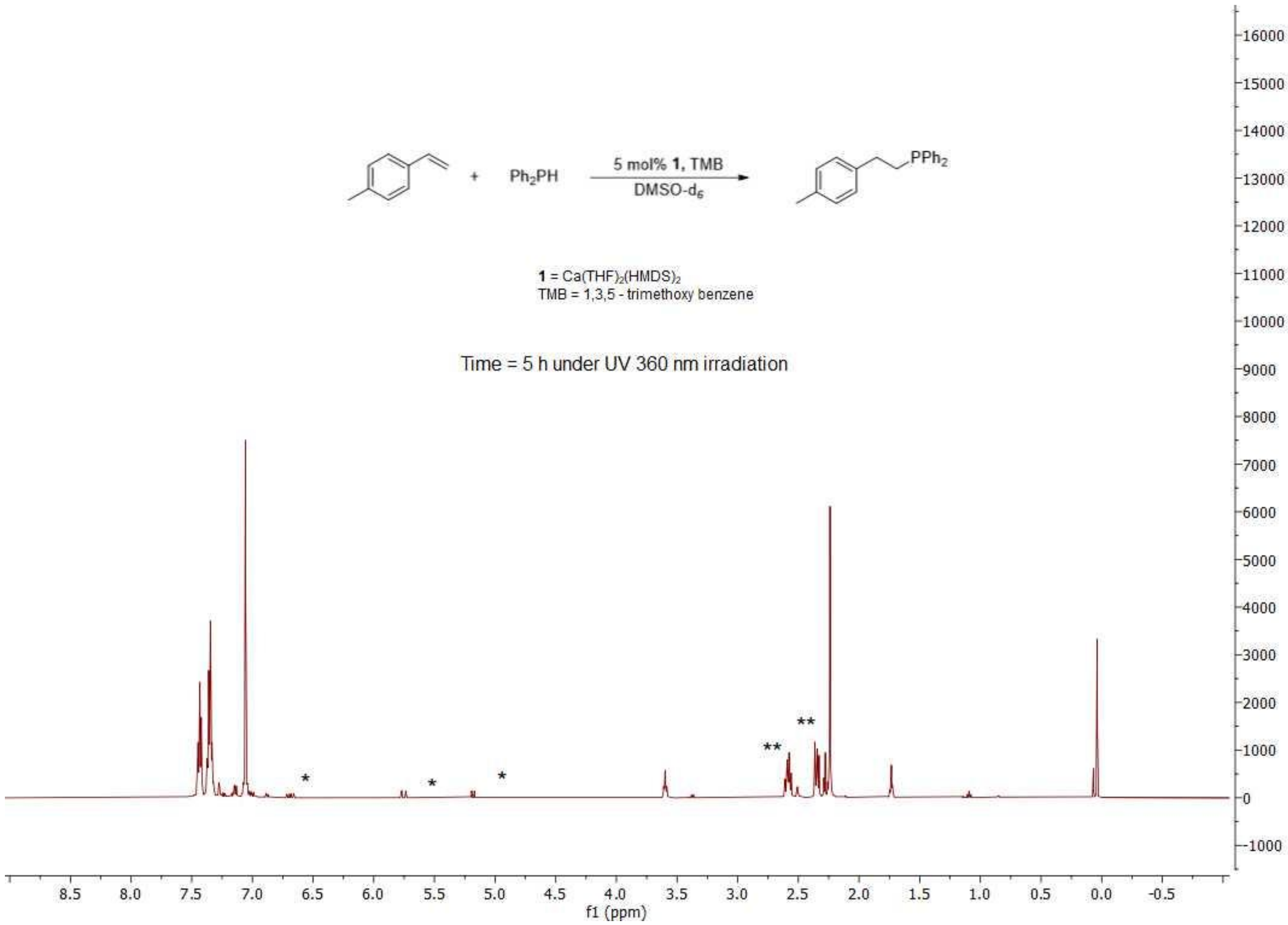

Figure S46: Hydrophosphination of 0.5 mmol 4-methyl styrene with equimolar of Ph_2PH in DMSO-d_6 using 5 mol% **1** under 360 nm UV light - 5 h – ^{1}H NMR

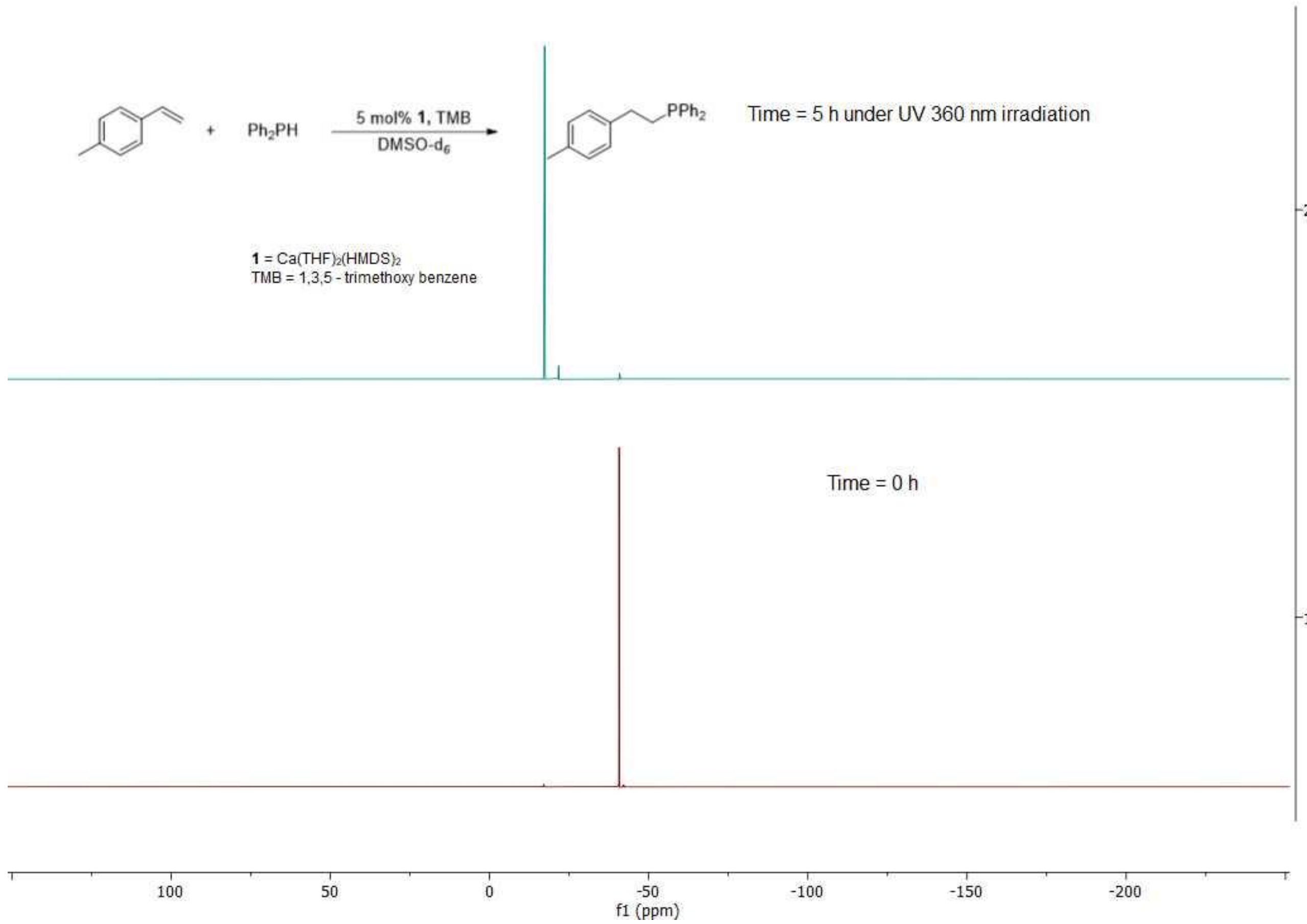

Figure S47: Hydrophosphination of 0.5 mmol 4-methyl styrene with equimolar of Ph_2PH in DMSO-d_6 using 5 mol% **1** under 360 nm UV light - stacked $^{31}P\{^1H\}$ NMR

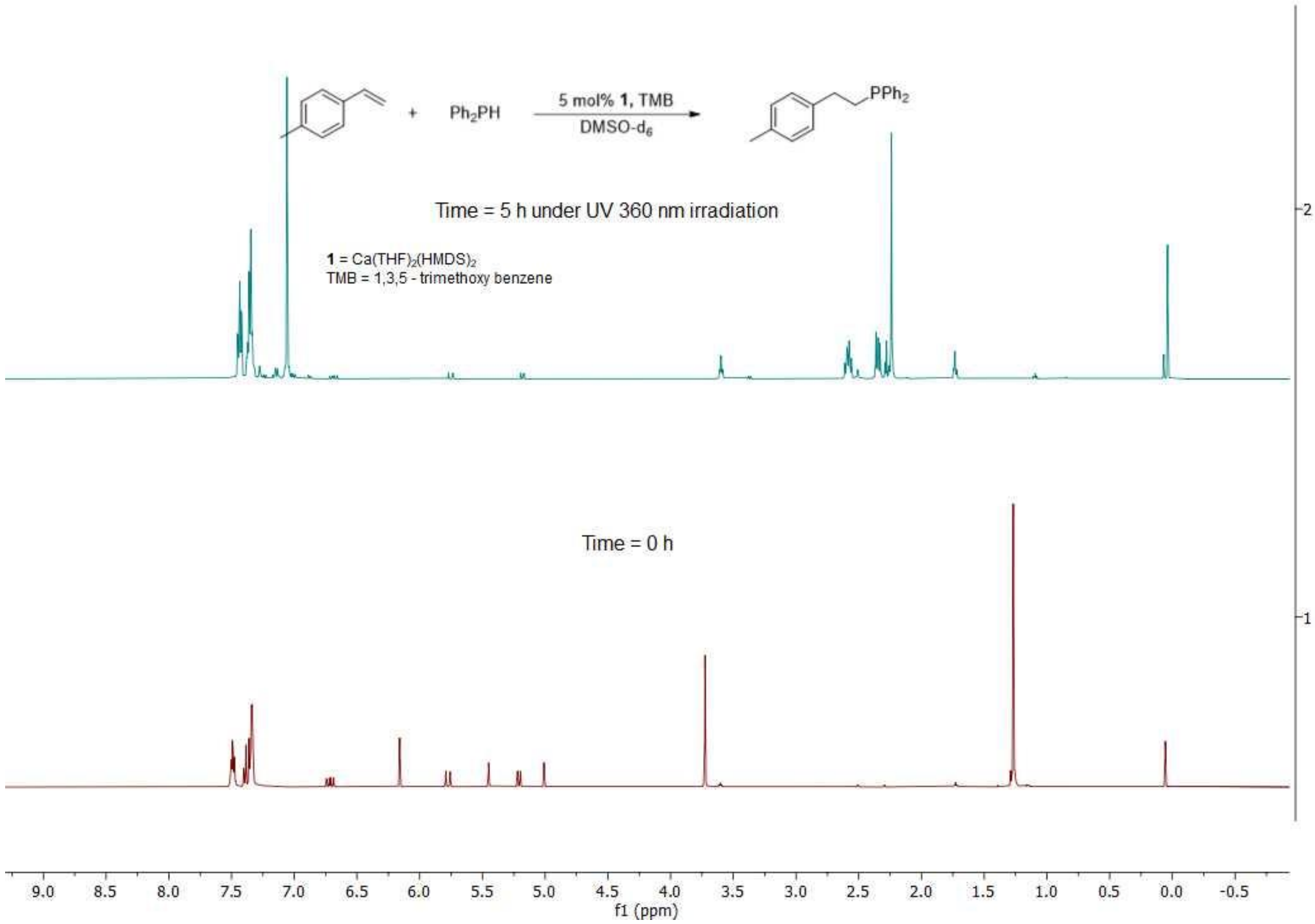

Figure S48: Hydrophosphination of 0.5 mmol 4-methyl styrene with equimolar of Ph_2PH in DMSO-d_6 using 5 mol% **1** under 360 nm UV light, stacked –^{1}H NMR

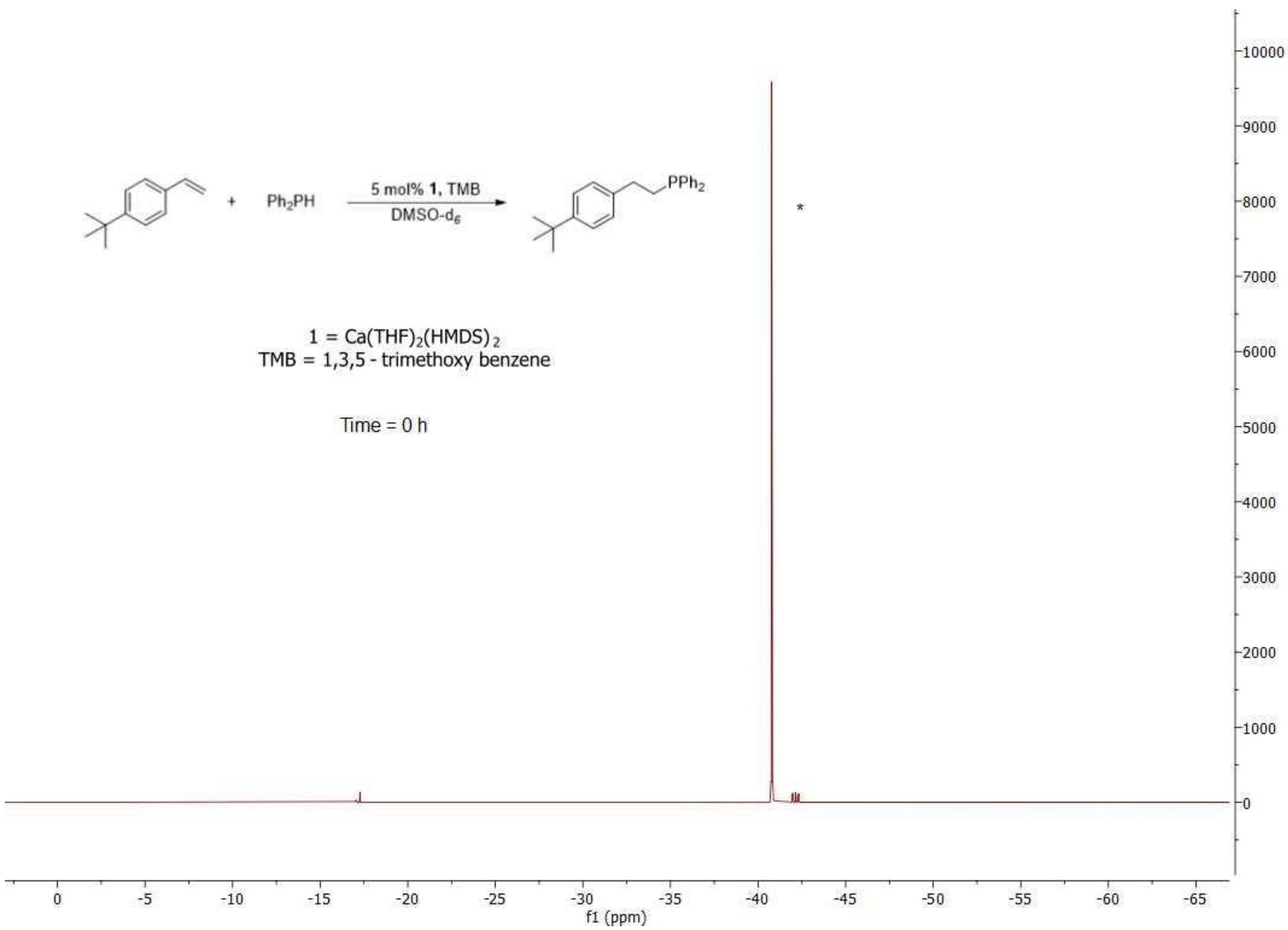

Figure S49: Hydrophosphination of 0.5 mmol 4-tertiarybutyl styrene with equimolar of Ph_2PH in DMSO-d_6 using 5 mol% **1** under ambient condition – 0 h –$^{31}P\{^1H\}$ NMR

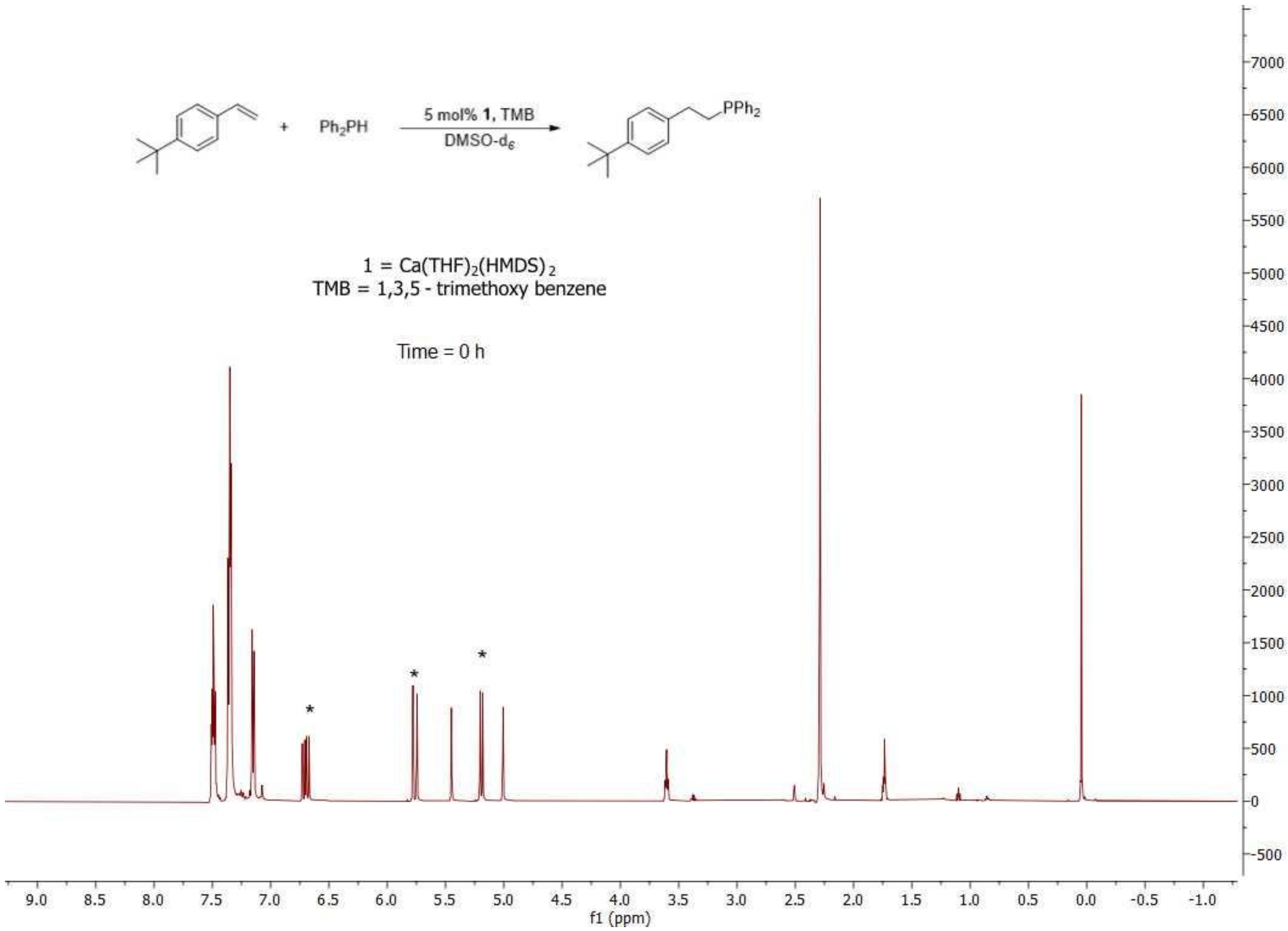

Figure S50: Hydrophosphination of 0.5 mmol 4-tertiarybutyl styrene with equimolar of Ph_2PH in DMSO-d_6 using 5 mol% **1** under ambient condition- 0 h–^{1}H NMR

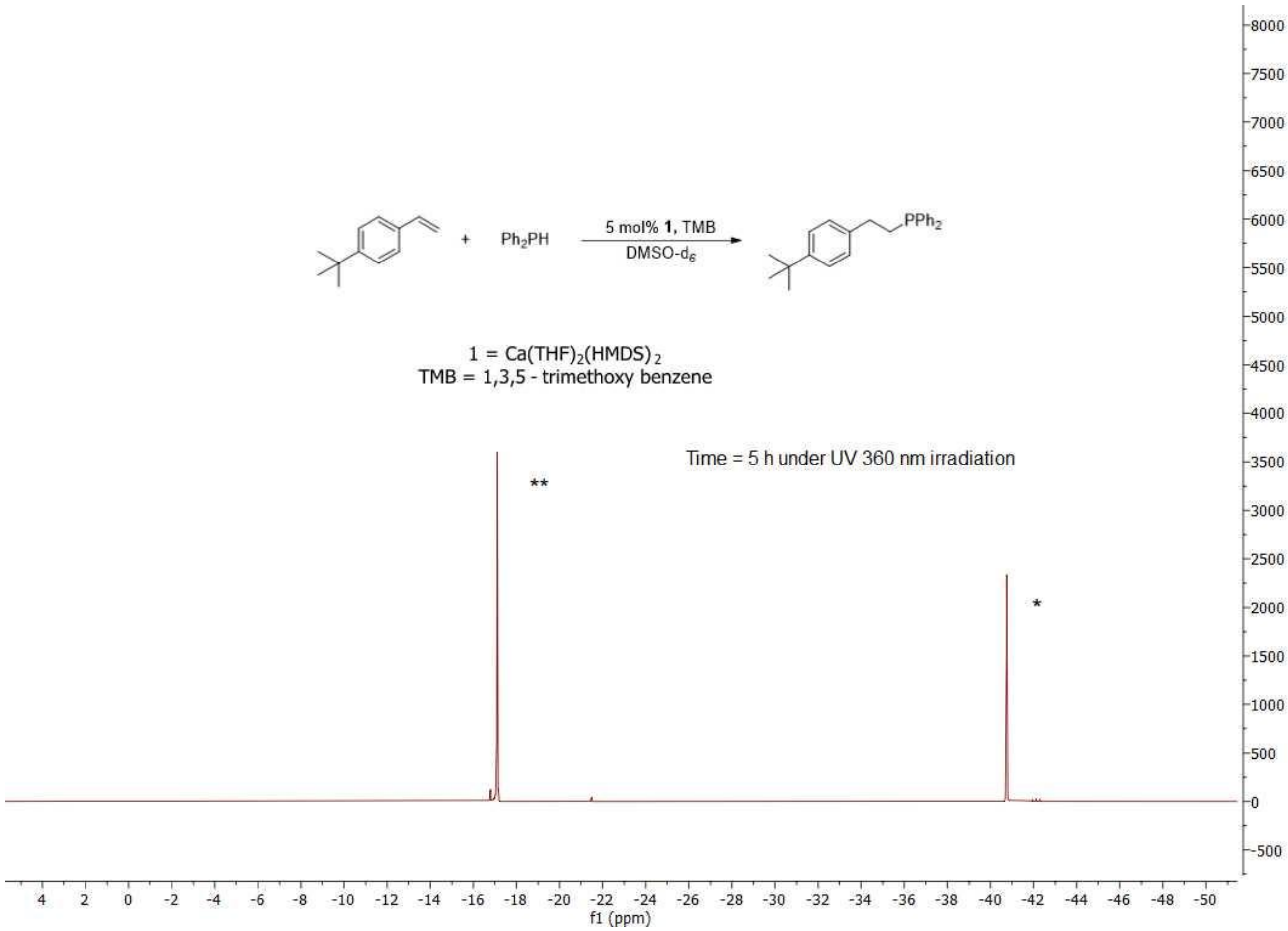

Figure S51: Hydrophosphination of 0.5 mmol 4-tertiarybutyl styrene with equimolar of Ph_2PH in DMSO-d_6 using 5 mol% **1** under 360 nm UV light - 5 h– $^{31}P\{^1H\}$ NMR

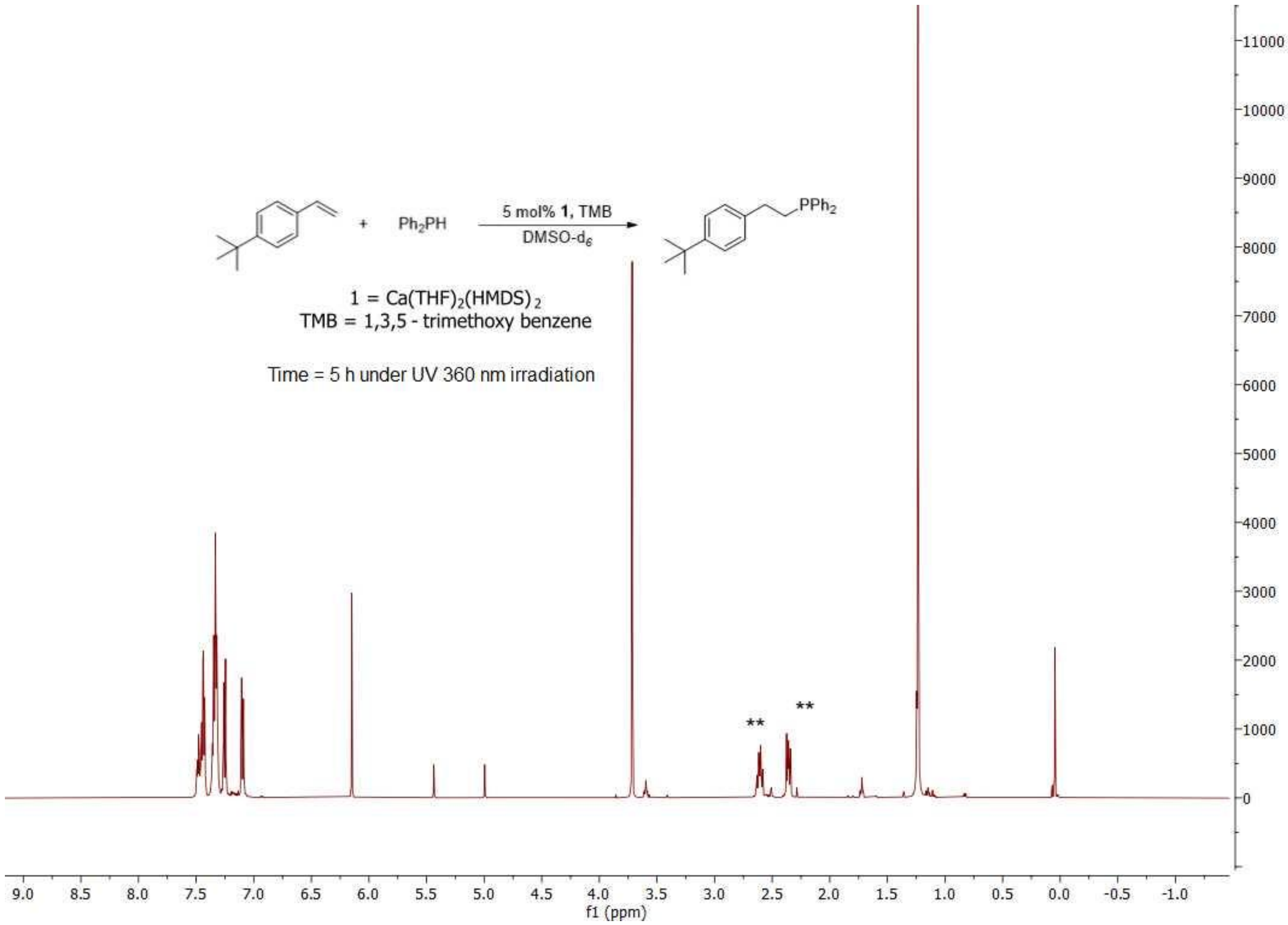

Figure S52: Hydrophosphination of 0.5 mmol 4-tertiarybutyl styrene with equimolar of Ph_2PH in DMSO-d_6 using 5 mol% **1** under 360 nm UV light - 5 h–^{1}H NMR

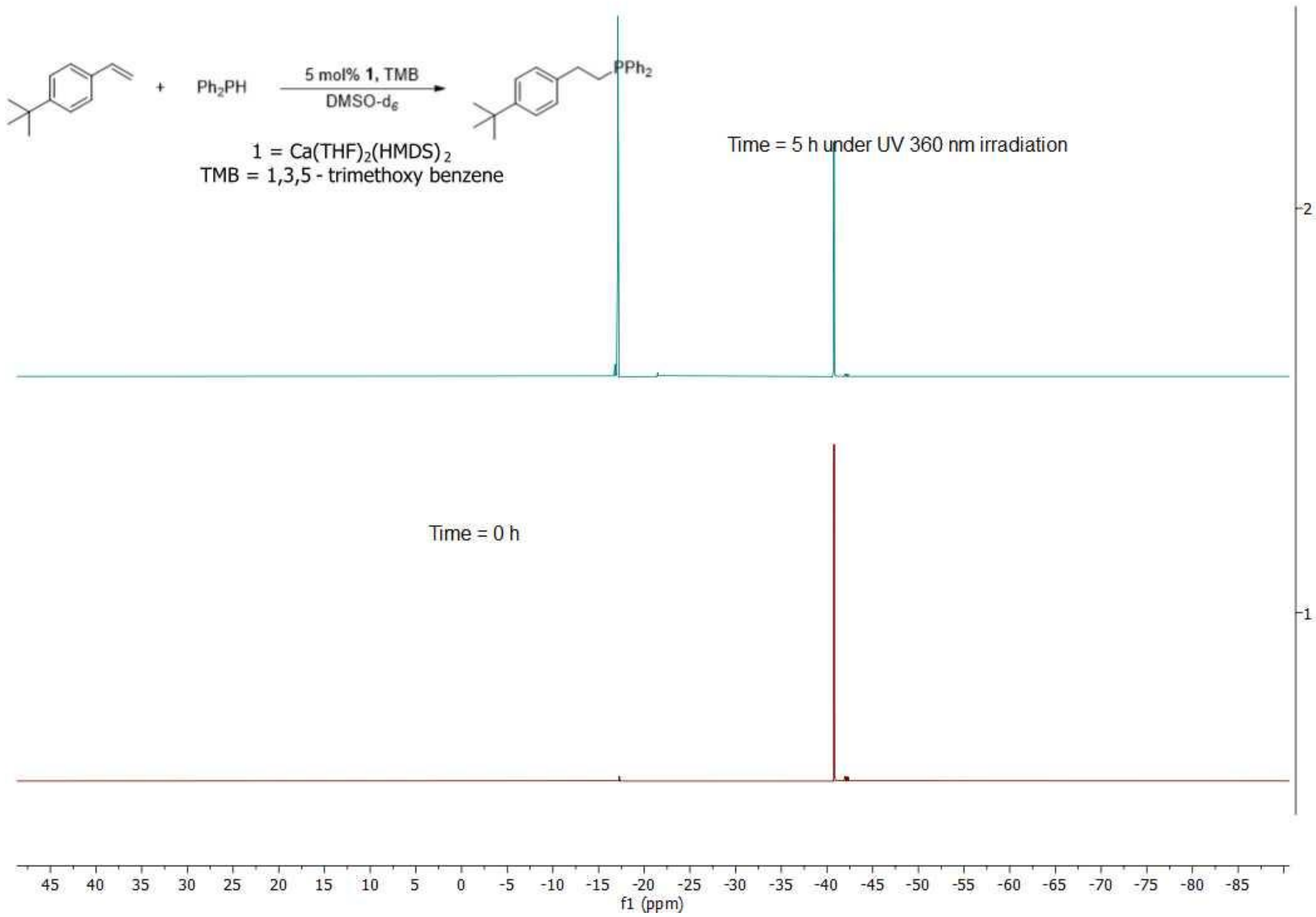

Figure S53: Hydrophosphination of 0.5 mmol 4-tertiarybutyl styrene with equimolar of Ph_2PH in DMSO-d_6 using 5 mol% **1** under 360 nm UV light - stacked $^{31}P\{^1H\}$ NMR

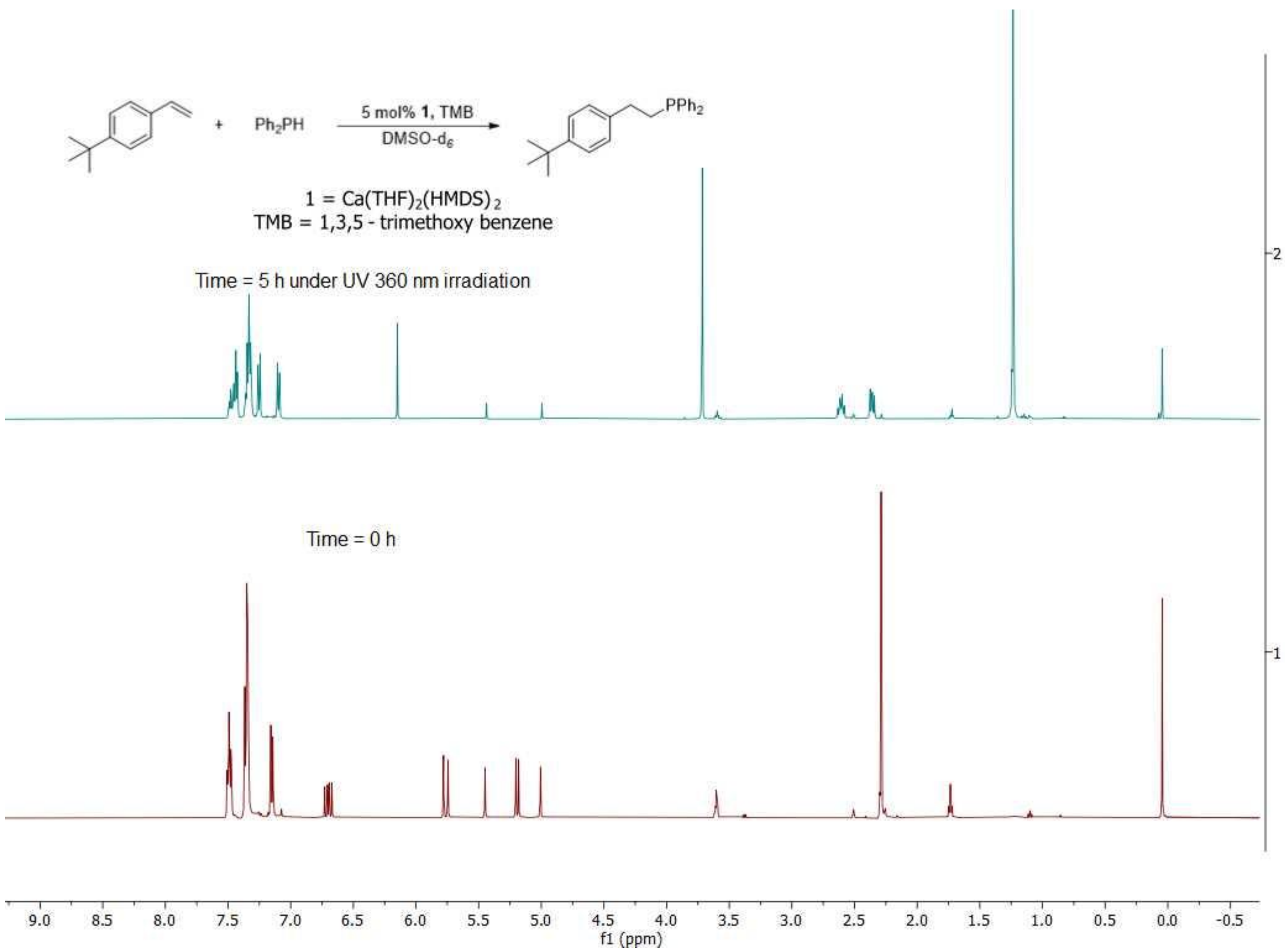

Figure S54: Hydrophosphination of 0.5 mmol 4-tertiarybutyl styrene with equimolar of Ph_2PH in DMSO-d_6 using 5 mol% **1** under 360 nm UV light - stacked ^{1}H NMR

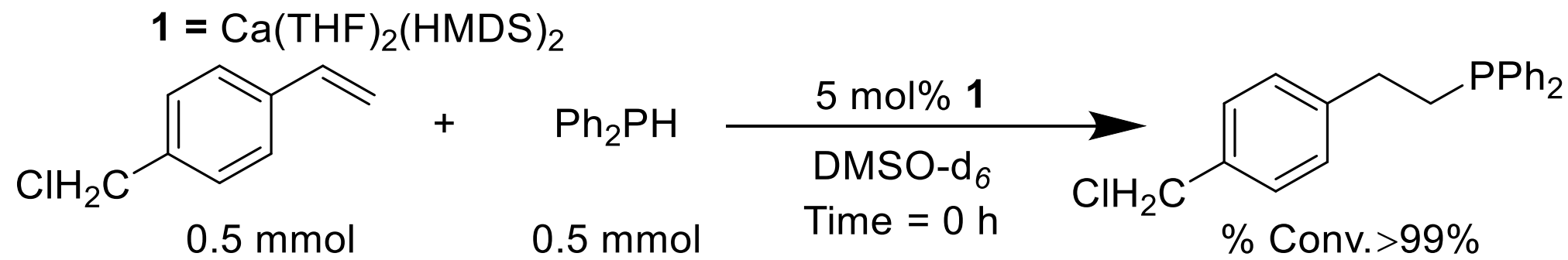

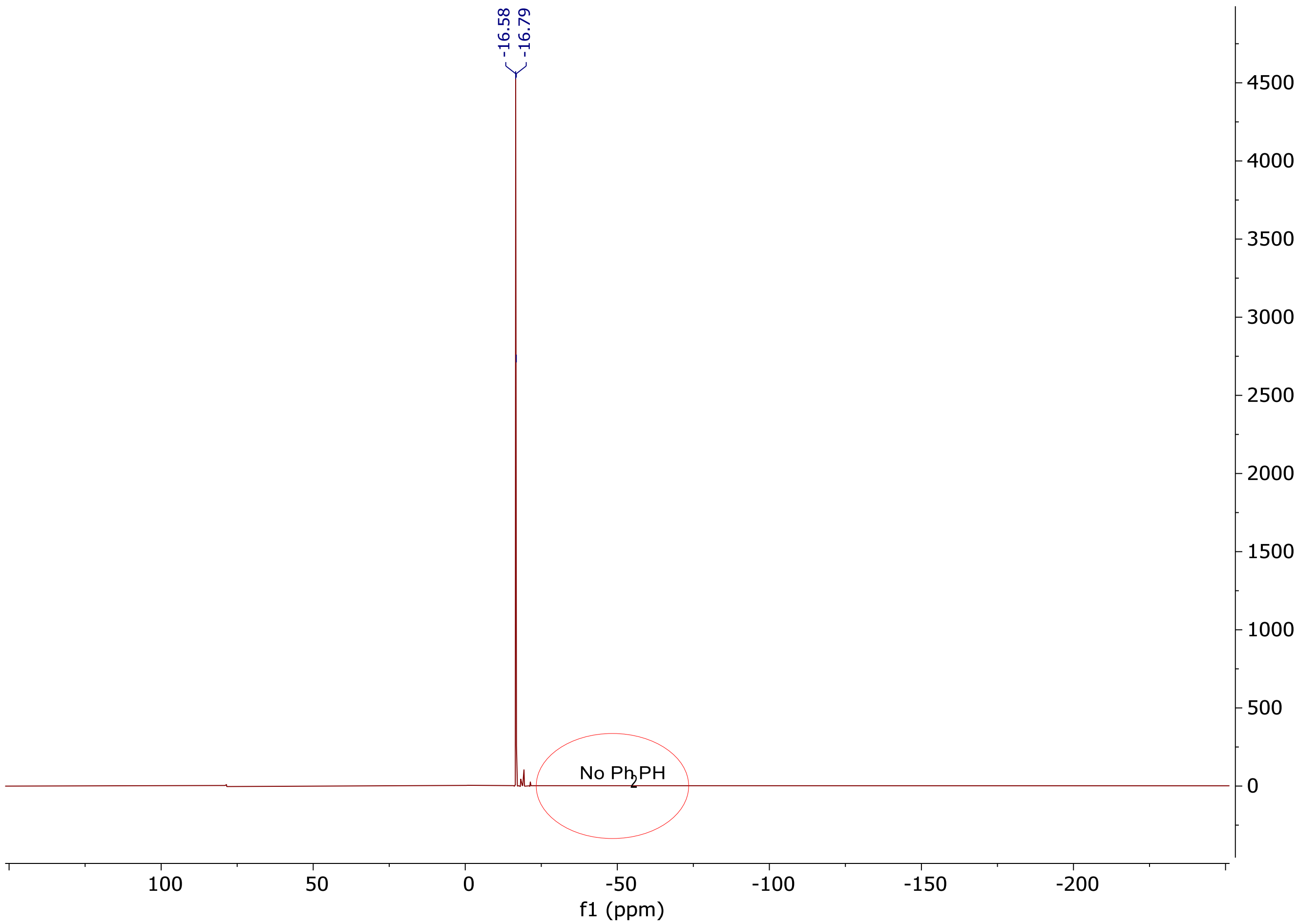

Figure S55: Hydrophosphination of 4-chloromethyl styrene with Ph_2PH in DMSO-d_6 at ambient conditions using 5 mol% **1,** and 1,3,5 – trimethoxybenzene as internal standard – 0 h – $^{31}P\{^1H\}$ NMR

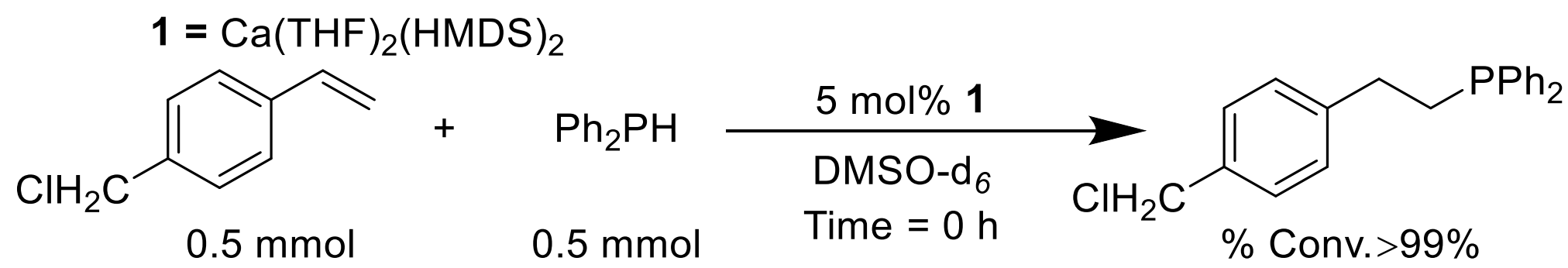

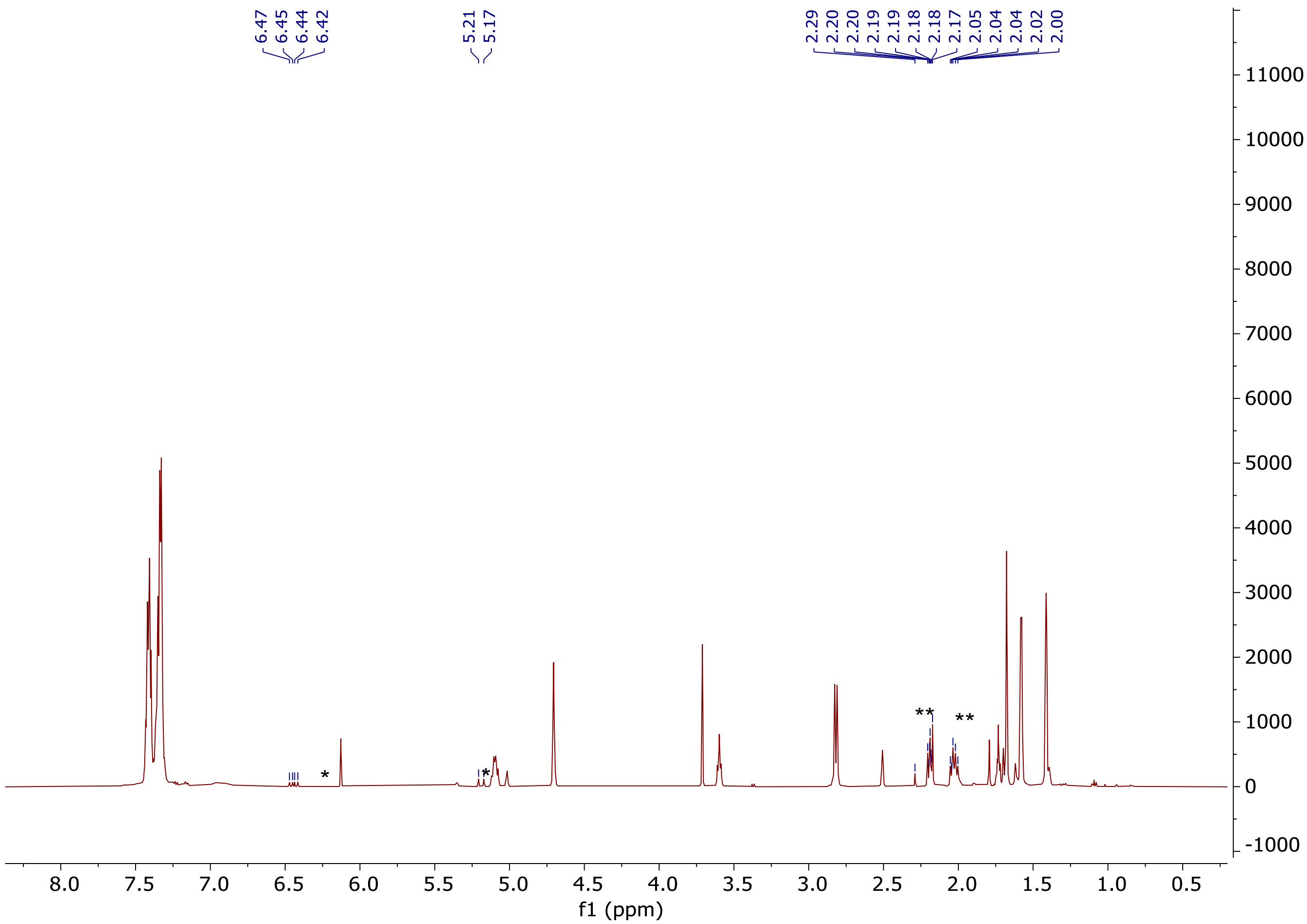

Figure S56: Hydrophosphination of 4-chloromethyl styrene with Ph_2PH in DMSO-d_6 at ambient conditions using 5 mol% **1,** and 1,3,5 – trimethoxybenzene as internal standard – 0 h – ^{1}H NMR

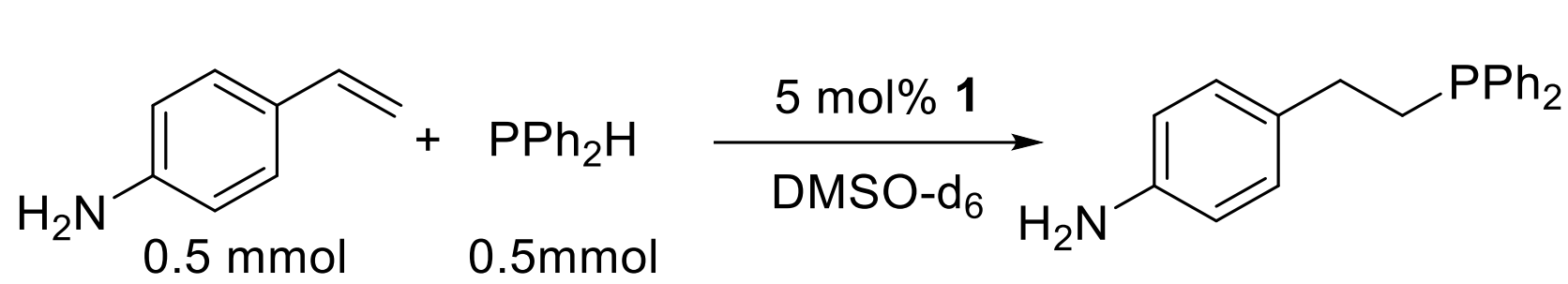

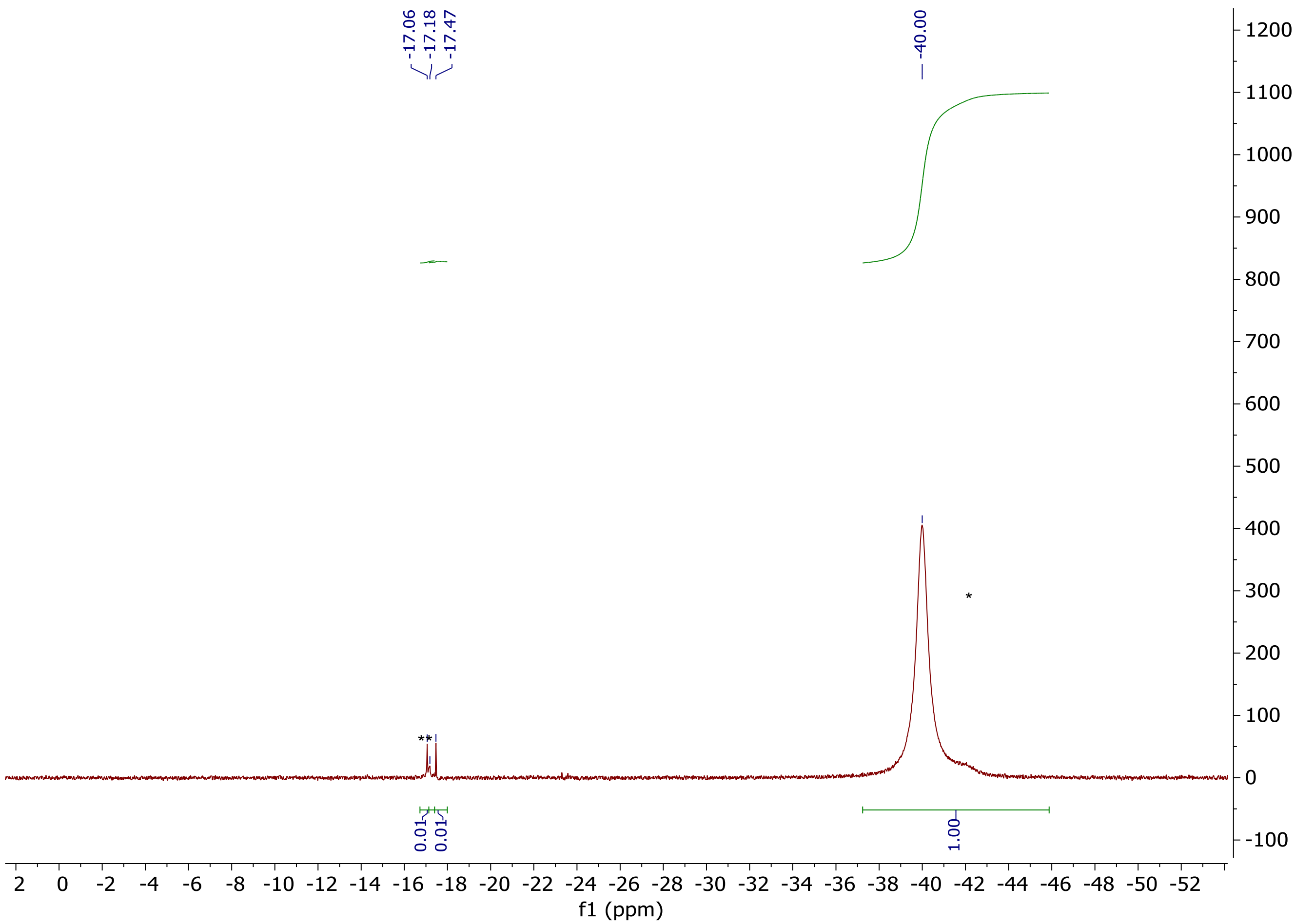

Figure S57: Hydrophosphination of 0.5 mmol 4-aminostyrene with equimolar of Ph_2PH in DMSO-d_6 using 5 mol% **1** under 360 nm UV light - 0 h – $^{31}P\{^1H\}$ NMR

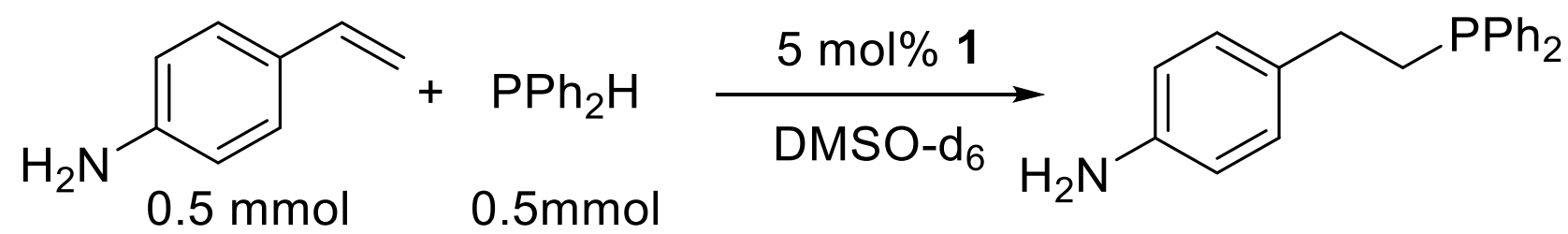

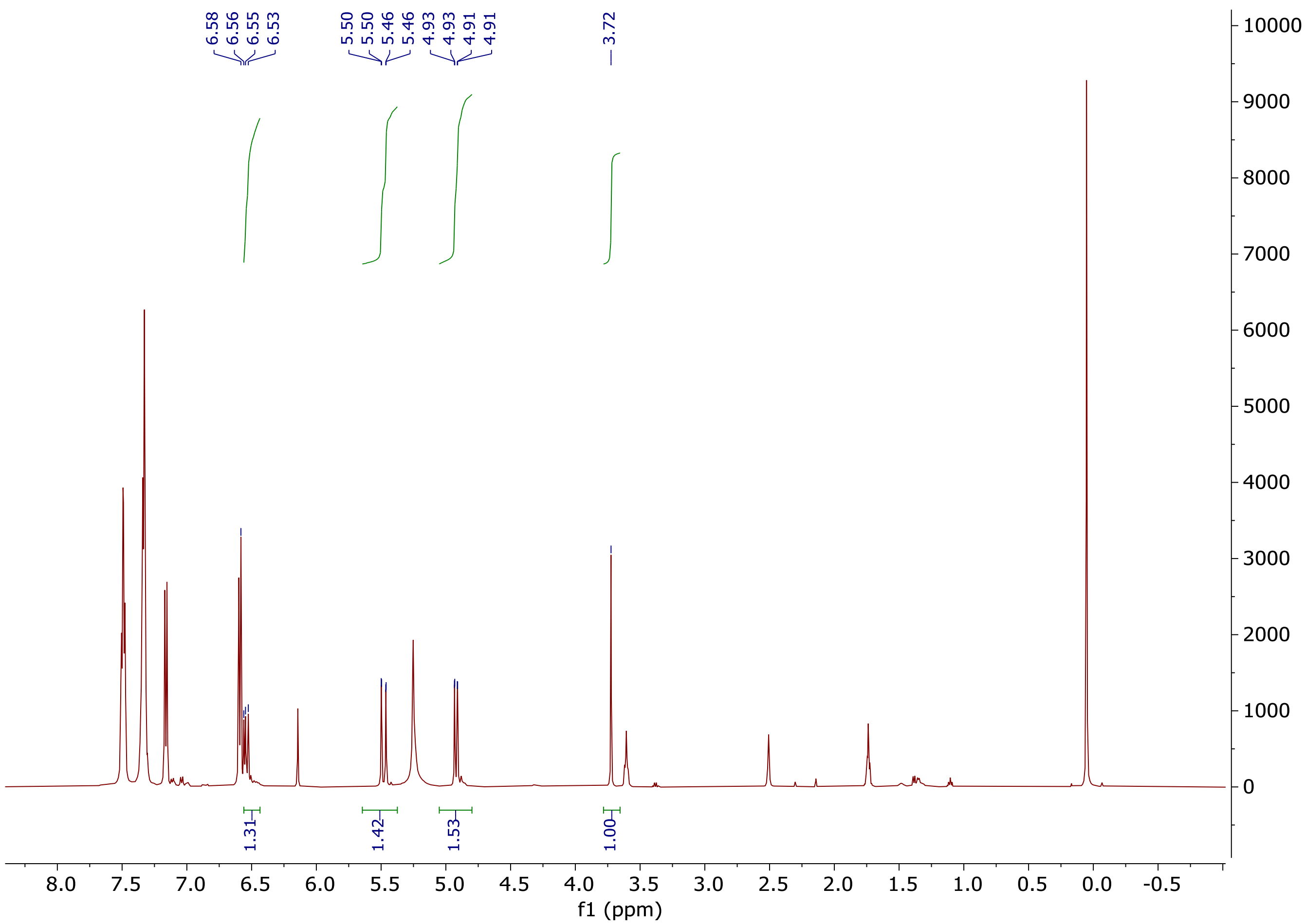

Figure S58: Hydrophosphination of 0.5 mmol 4-aminostyrene with equimolar of Ph_2PH in DMSO-d_6 using 5 mol% **1** under 360 nm UV light – 0 h - ^{1}H NMR

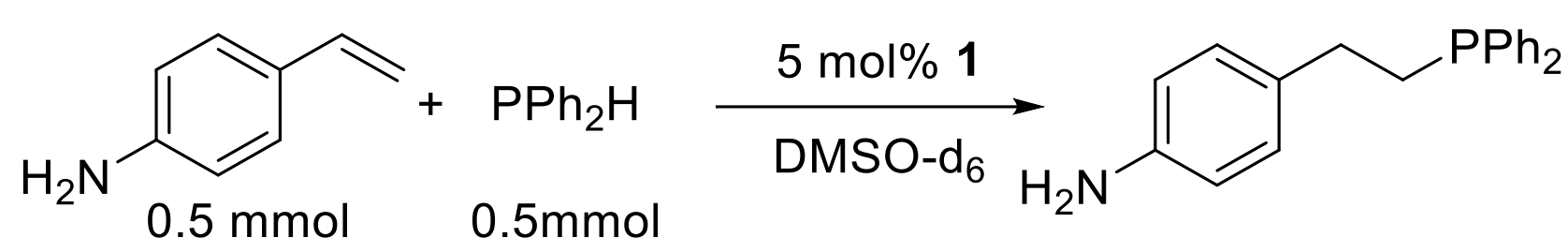

4h under UV 360 nm irradiation

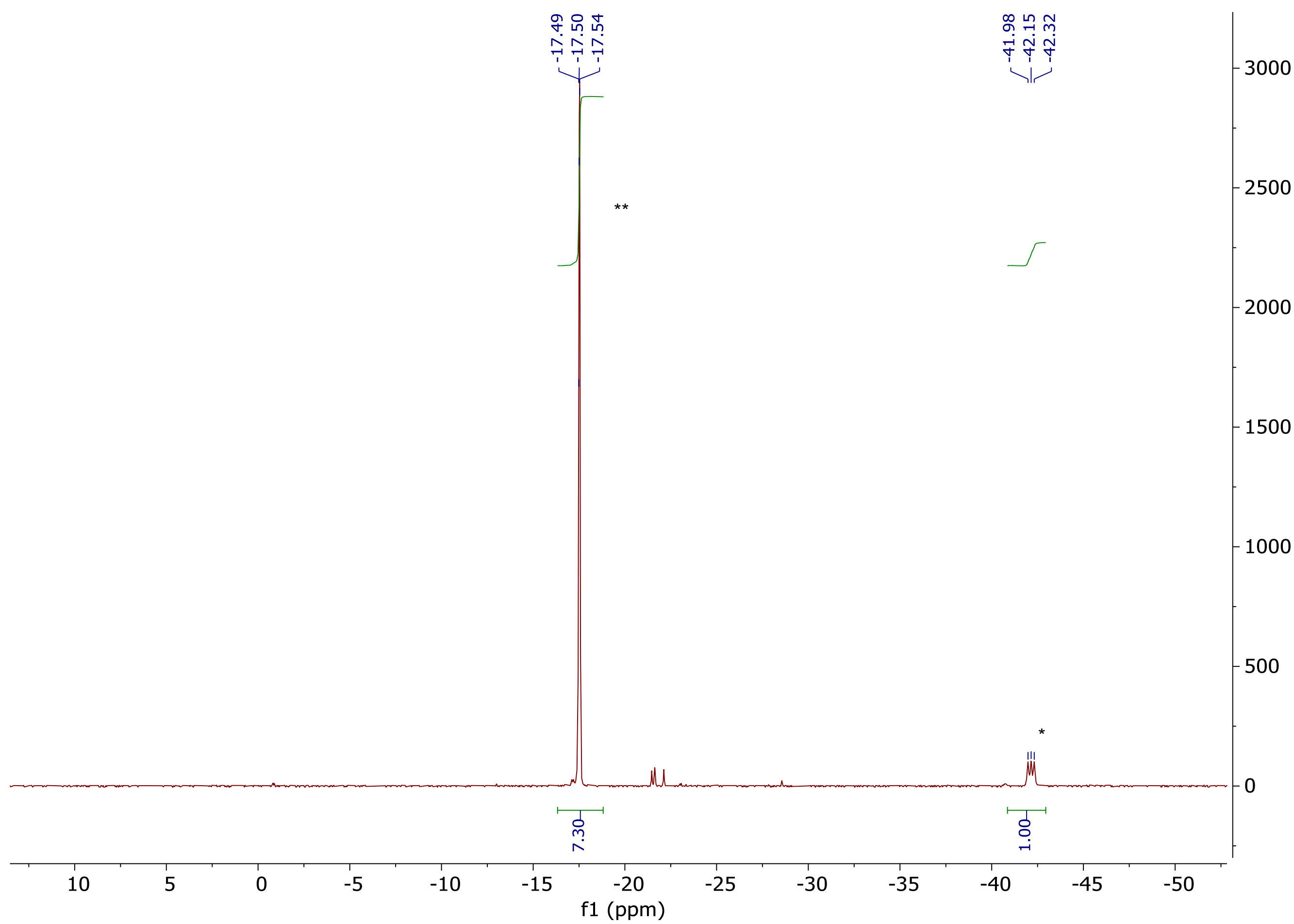

Figure S59: Hydrophosphination of 0.5 mmol 4-aminostyrene with equimolar of Ph_2PH in DMSO-d_6 using 5 mol% **1** under 360 nm UV light – 4 h – $^{31}P\{^1H\}$ NMR

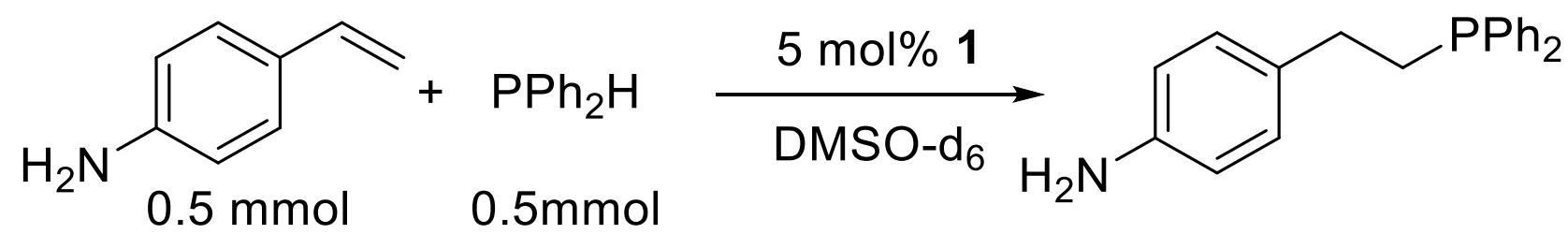

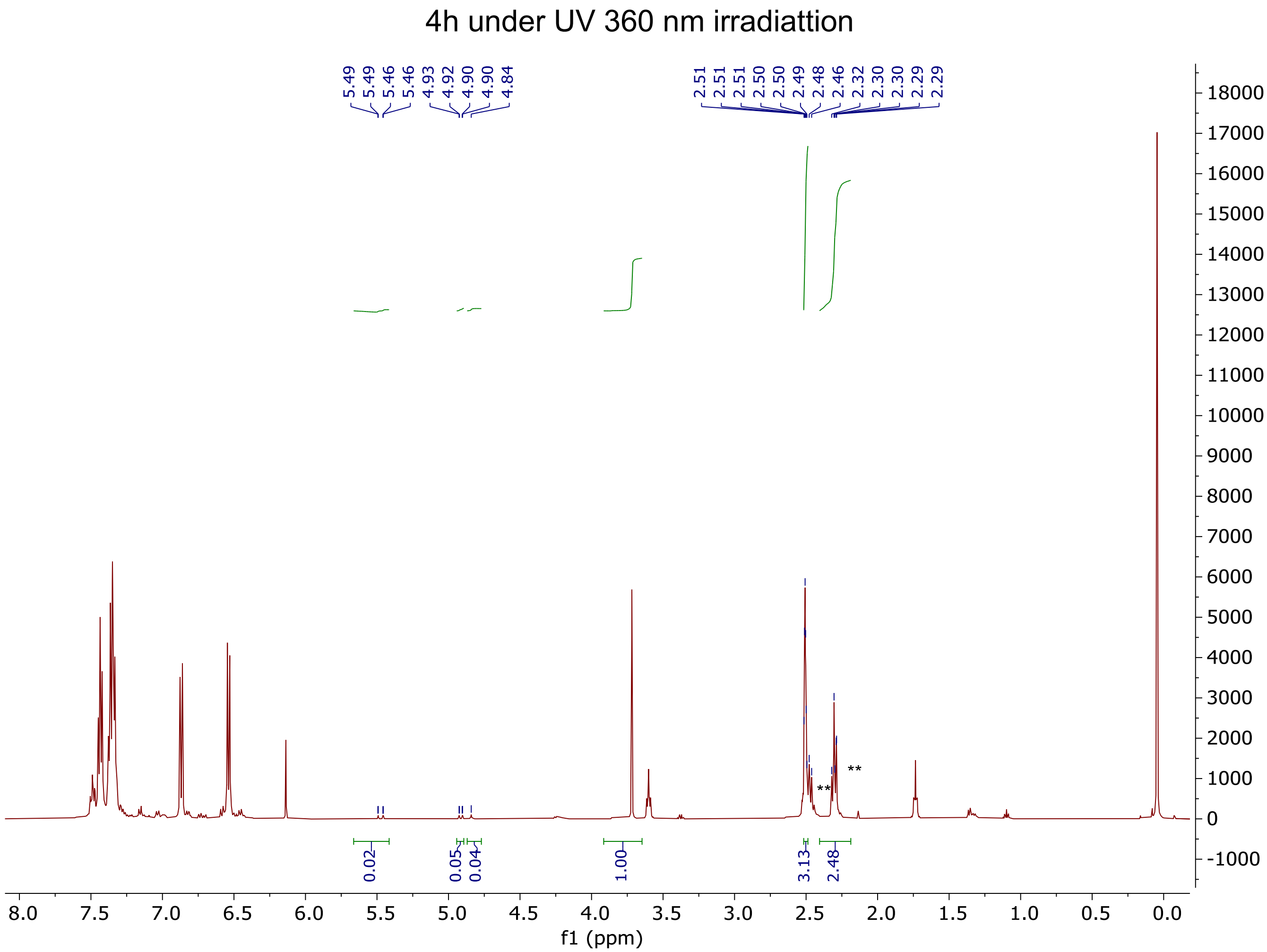

Figure S60: Hydrophosphination of 0.5 mmol 4-aminostyrene with equimolar of Ph_2PH in DMSO-d_6 using 5 mol% **1** under 360 nm UV light – 4 h -^{1}H NMR

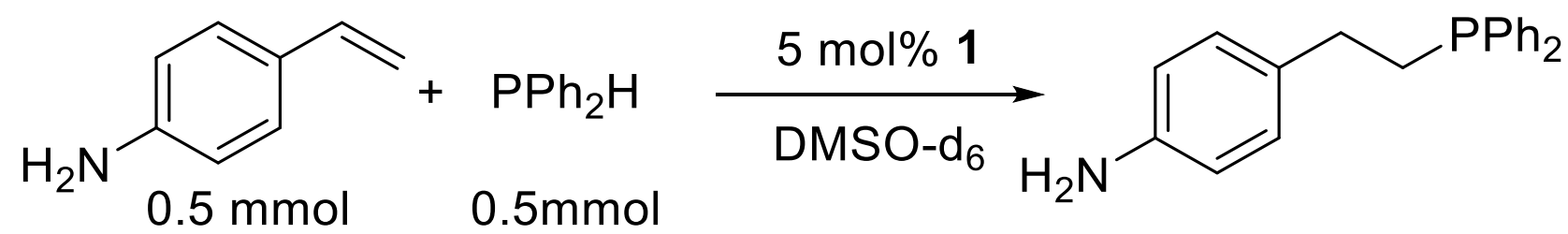

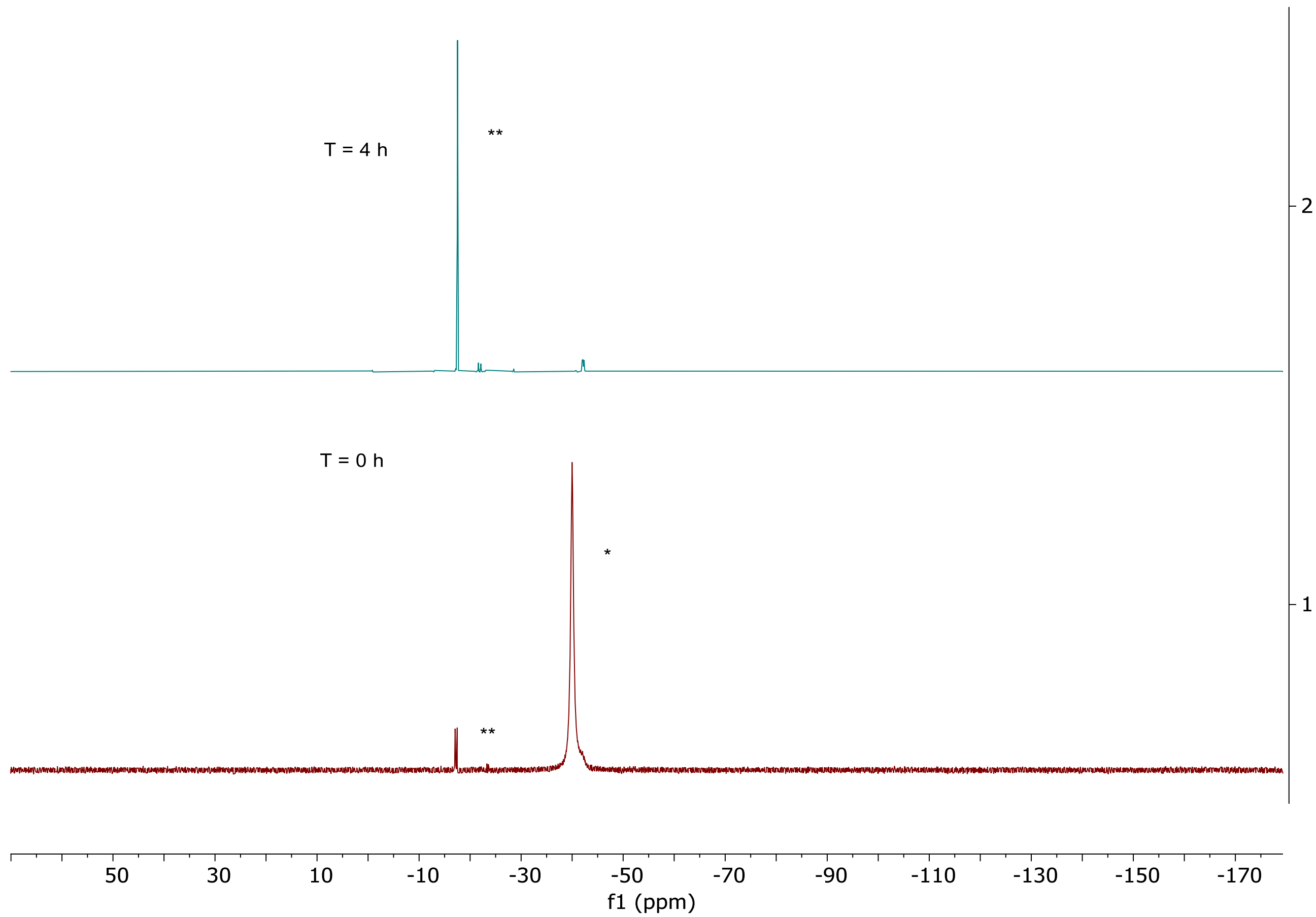

Figure S61: Hydrophosphination of 0.5 mmol 4-aminostyrene with equimolar of Ph_2PH in DMSO-d_6 using 5 mol% **1** under 360 nm UV light – stacked $^{31}P\{^1H\}$ NMR

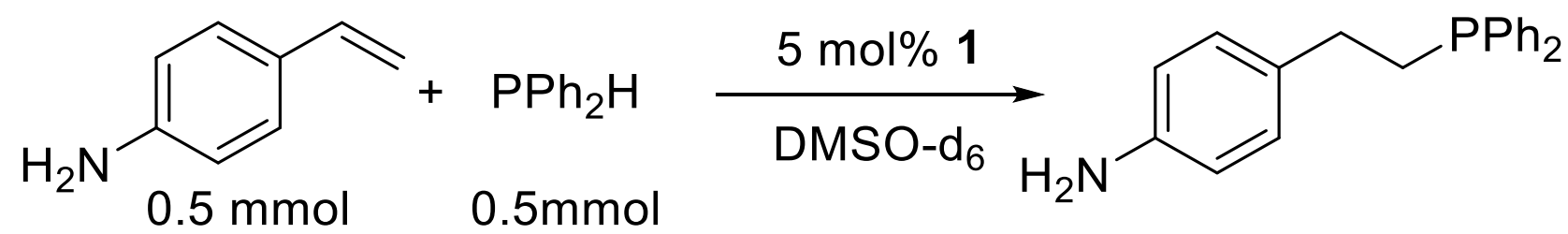

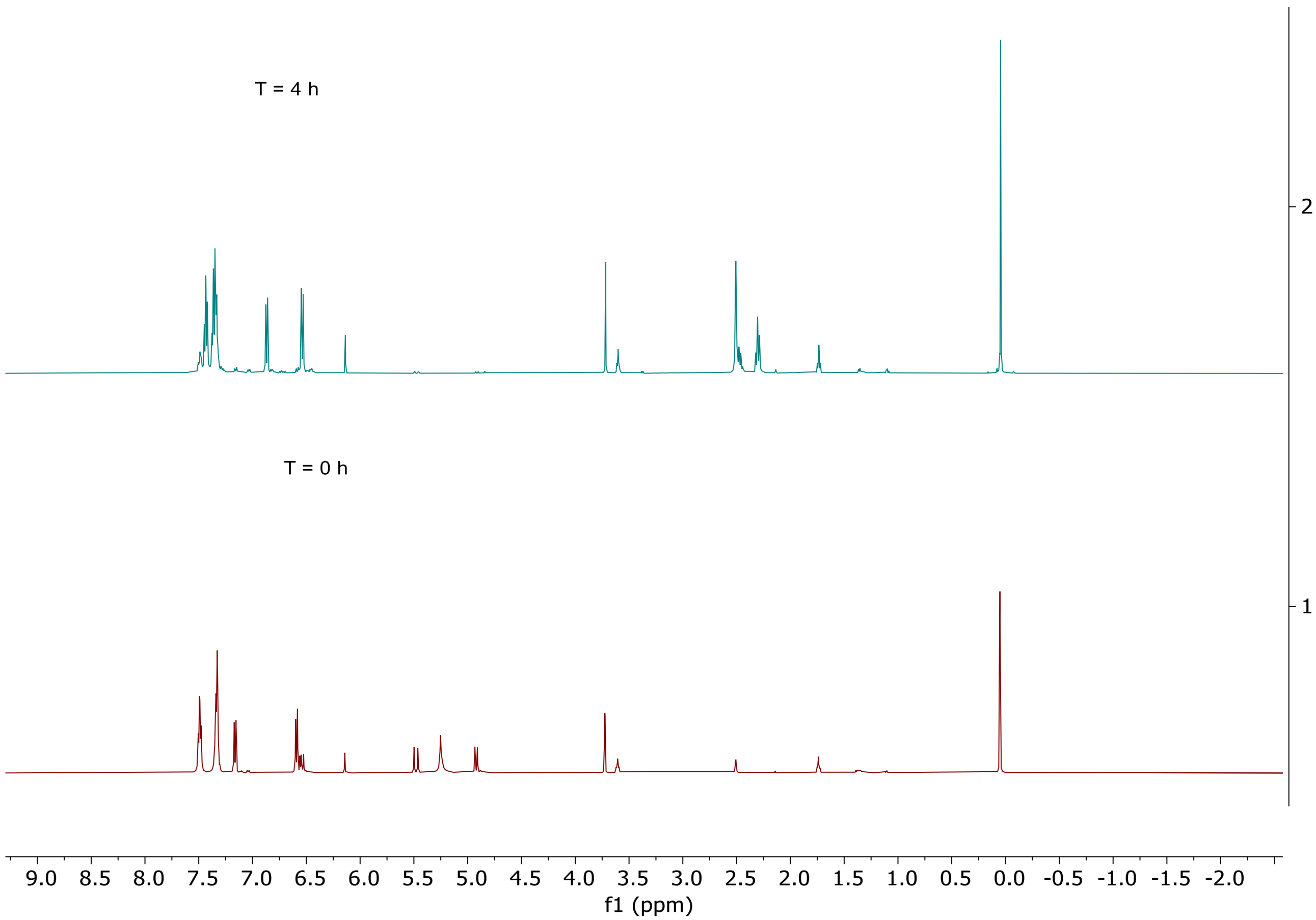

Figure S62: Hydrophosphination of 0.5 mmol 4-amionstyrene with equimolar of Ph_2PH in DMSO-d_6 using 5 mol% **1** under 360 nm UV light – stacked ^1H NMR

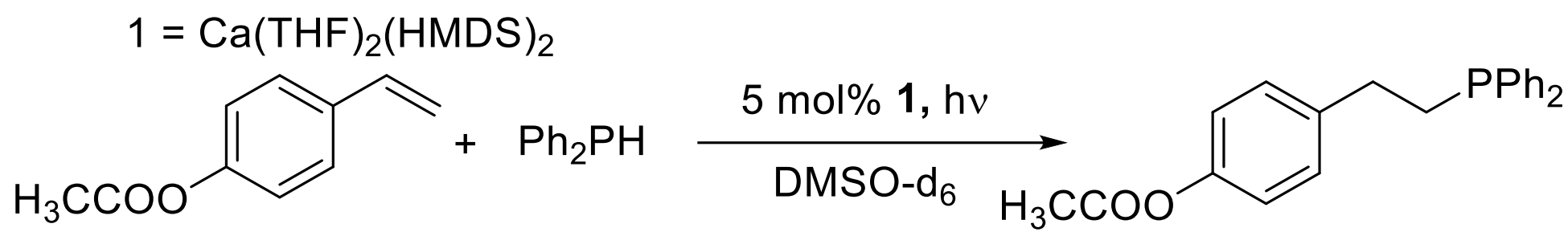

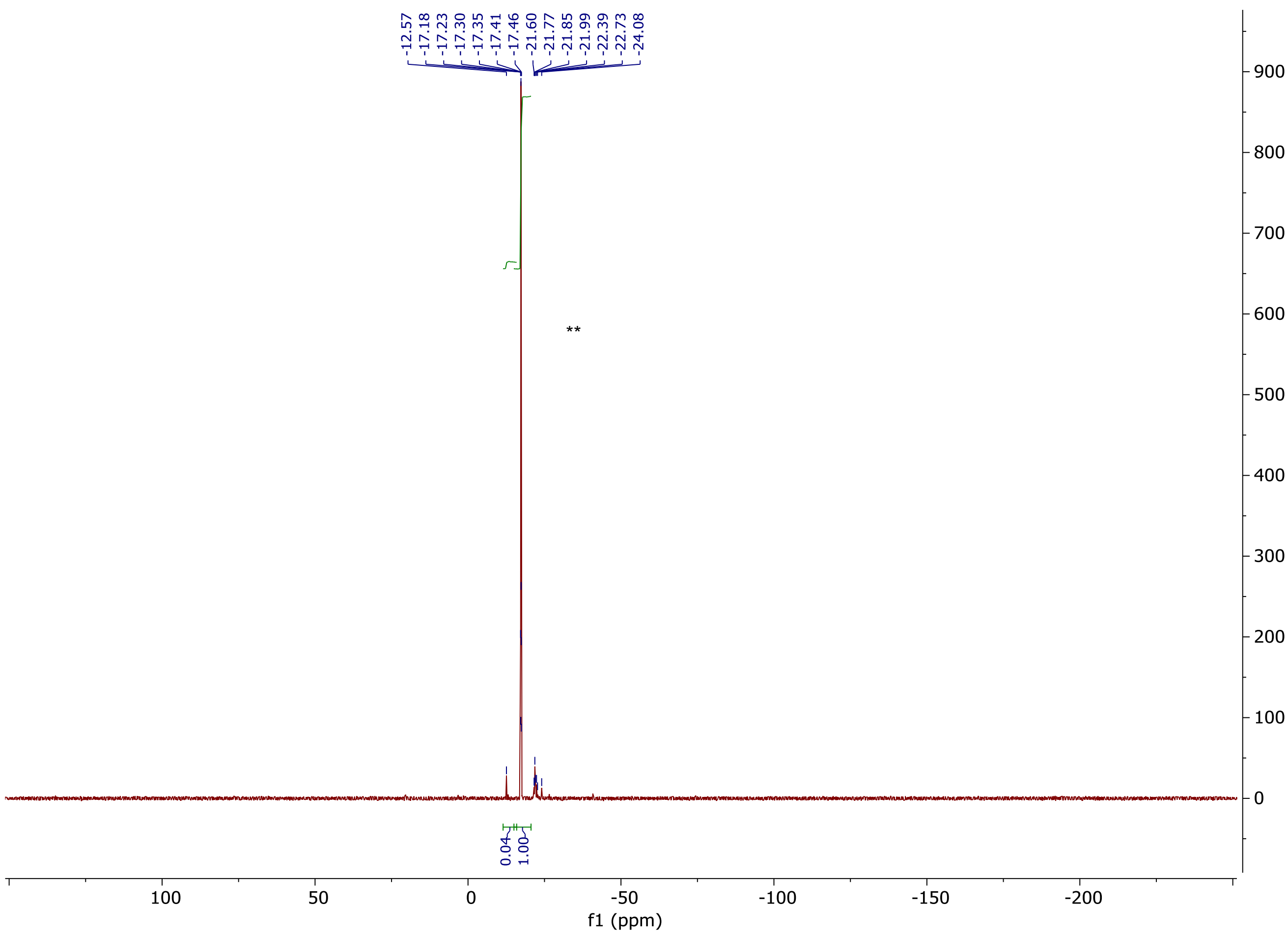

Figure S63: Hydrophosphination of 0.5 mmol 4 - acetyloxy styrene with equimolar of Ph_2PH in DMSO-d_6 using 5 mol% **1** under UV 360 nm irradiation – 18 h – $^{31}P\{^1H\}$ NMR

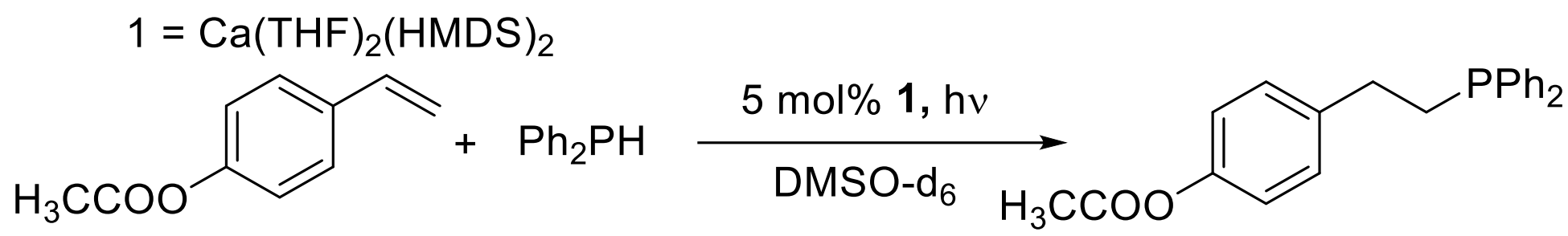

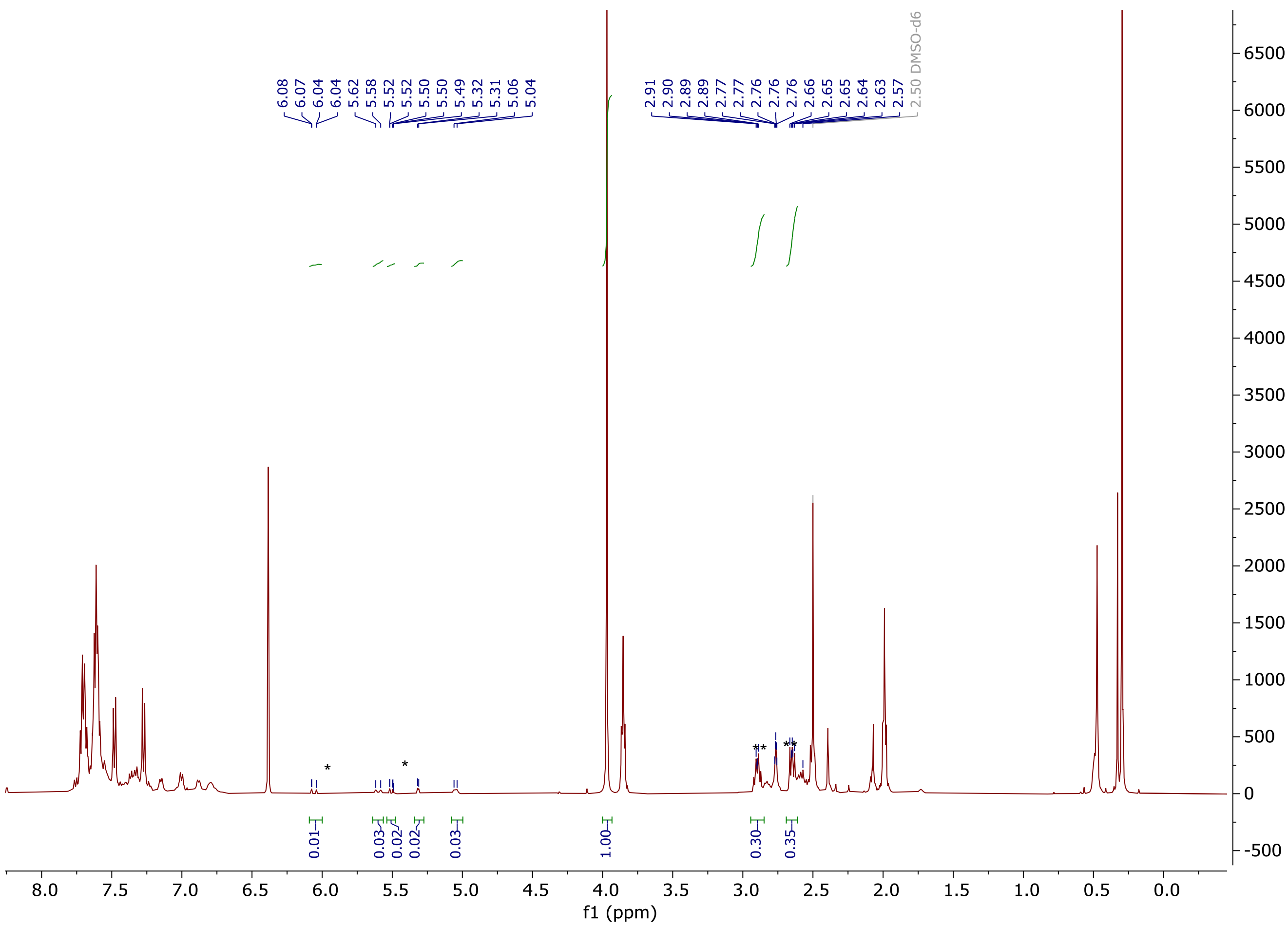

Figure S64: Hydrophosphination of 0.5 mmol 4 - acetyloxy styrene with equimolar of Ph_2PH in DMSO-d_6 using 5 mol% **1** under UV 360 nm irradiation – 18 h – ^{1}H NMR

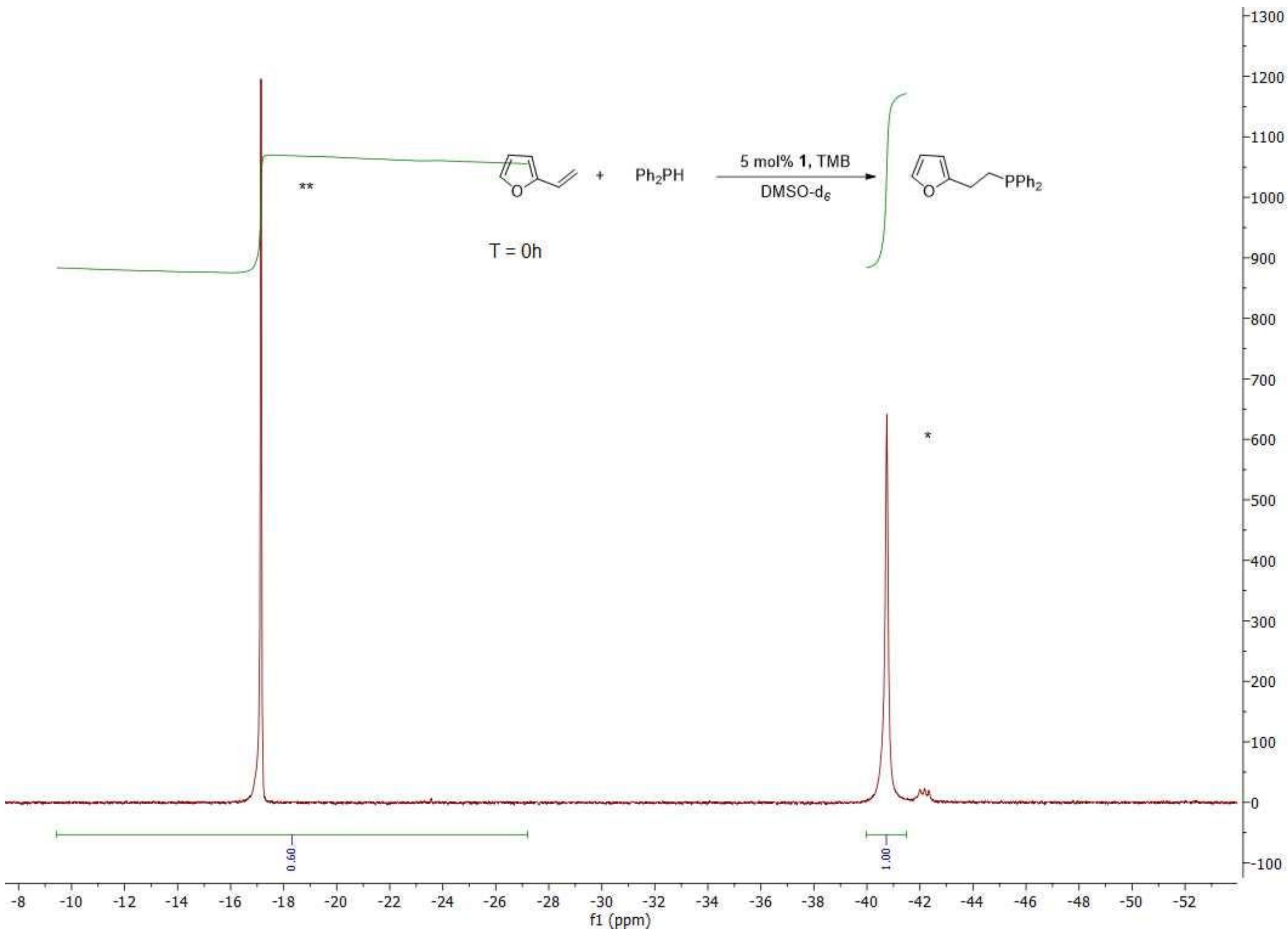

Figure S65: Hydrophosphination of 0.5 mmol 2-vinyl furan with equimolar of Ph_2PH in DMSO-d_6 using 5 mol% **1** under 360 nm UV light – 0 h – $^{31}P\{^1H\}$ NMR

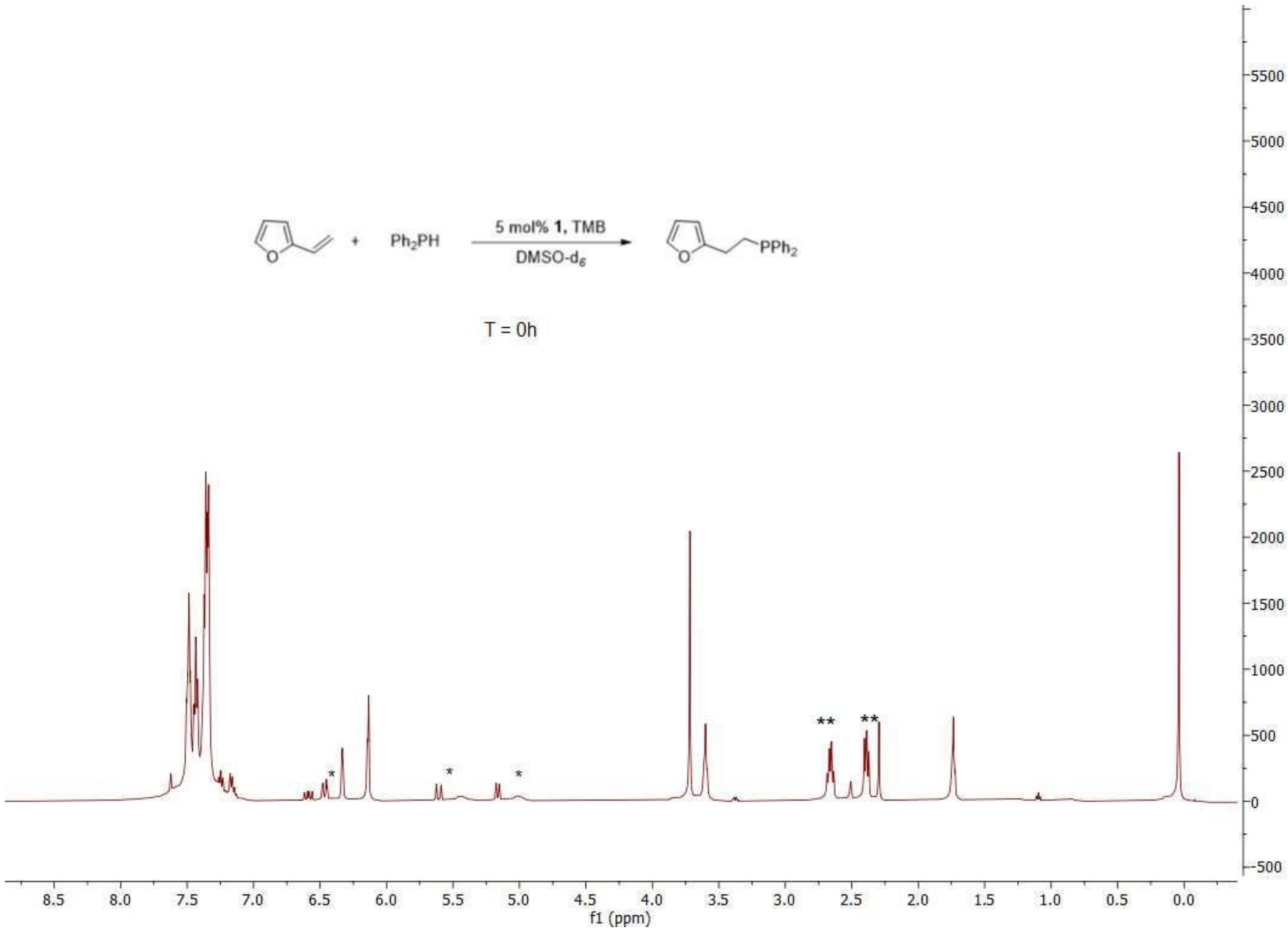

Figure S66: Hydrophosphination of 0.5 mmol 2-vinyl furan with equimolar of Ph_2PH in DMSO-d_6 using 5 mol% **1** under 360 nm UV light – 0 h - ^{1}H NMR

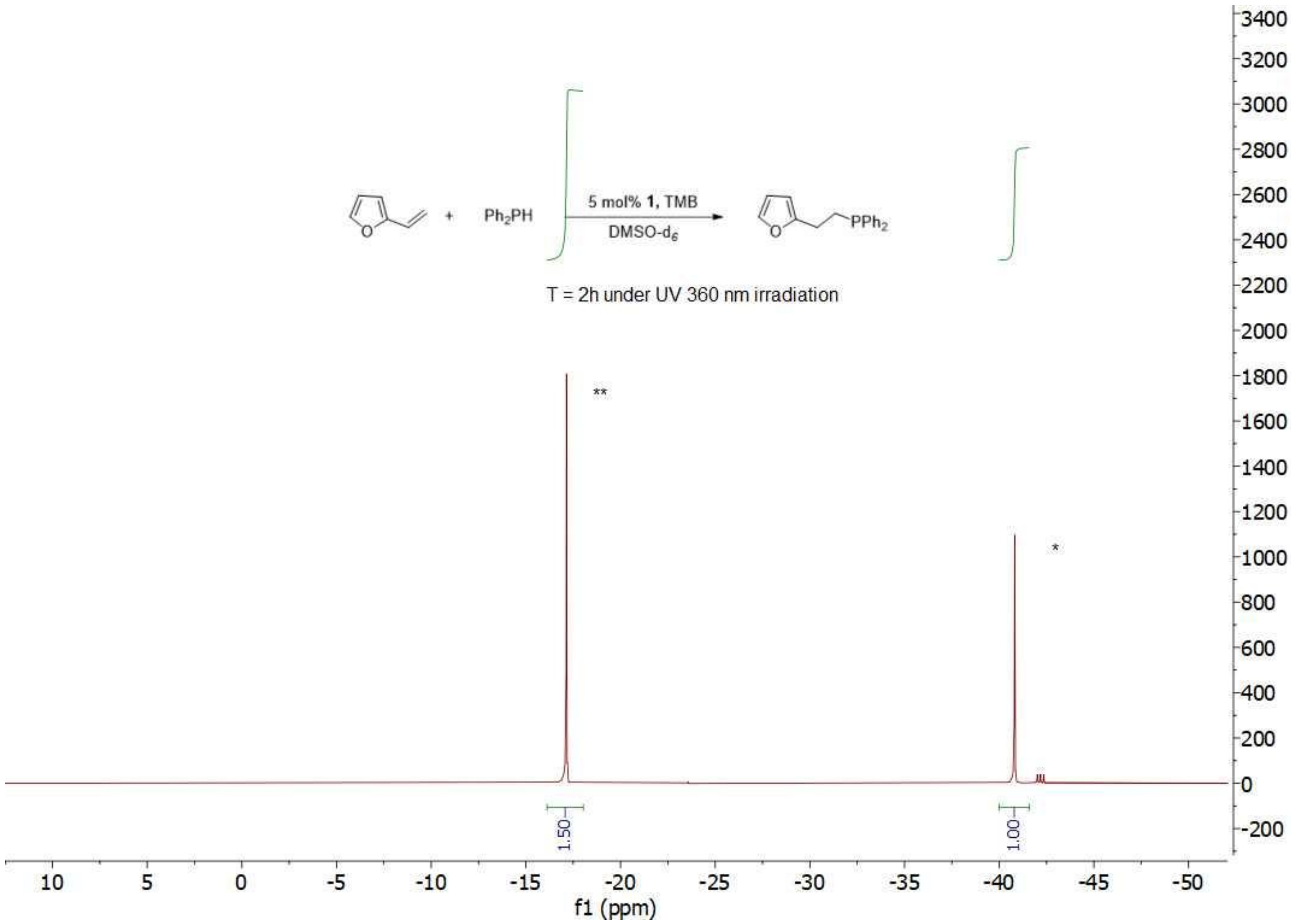

Figure S67: Hydrophosphination of 0.5 mmol 2-vinyl furan with equimolar of Ph_2PH in DMSO-d_6 using 5 mol% **1** under 360 nm UV light – 2 h – $^{31}P\{^1H\}$ NMR

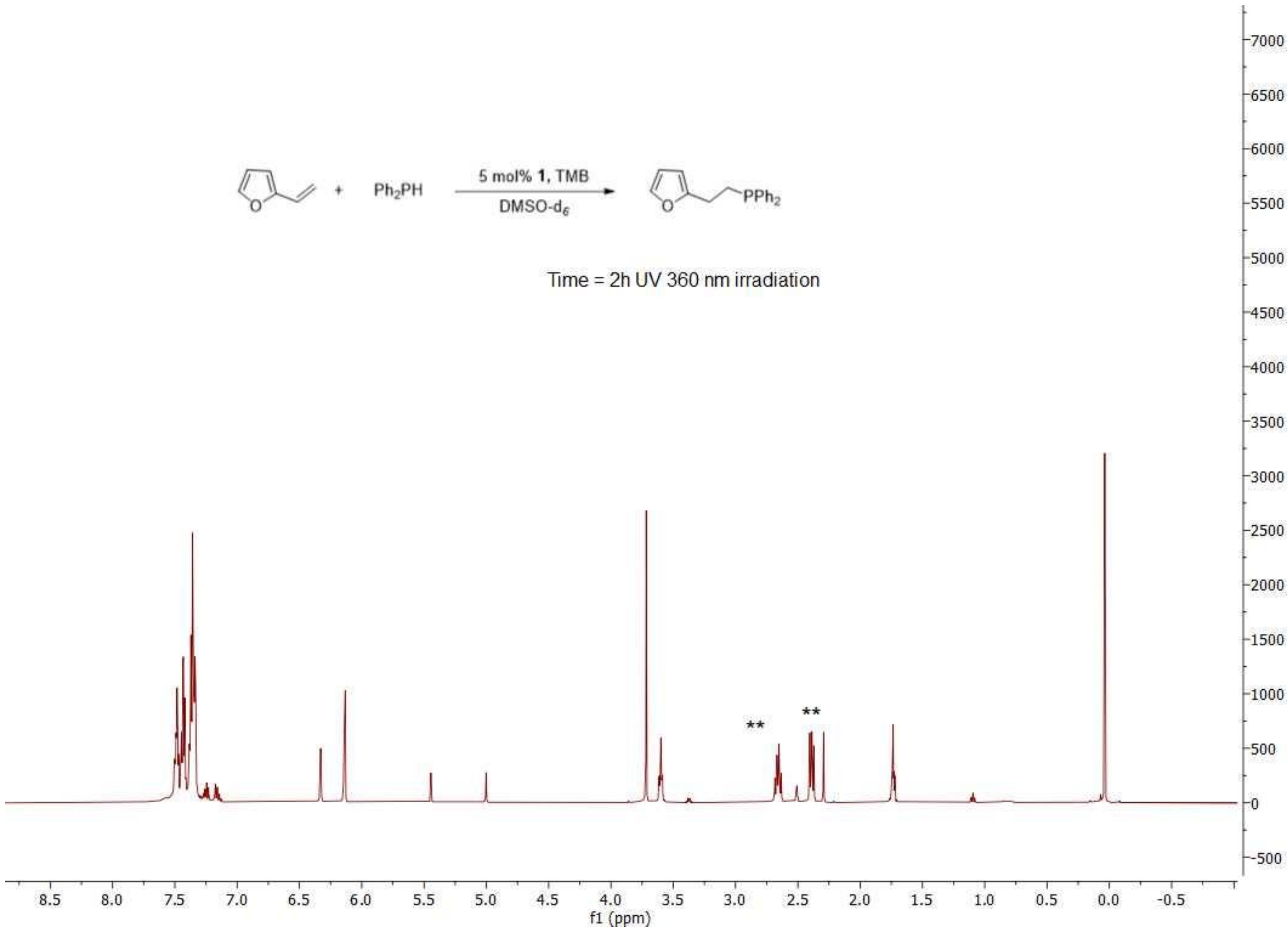

Figure S68: Hydrophosphination of 0.5 mmol 2-vinyl furan with equimolar Ph_2PH in DMSO-d_6 using 5 mol% **1** under 360 nm UV light – 2 h - ^{1}H NMR

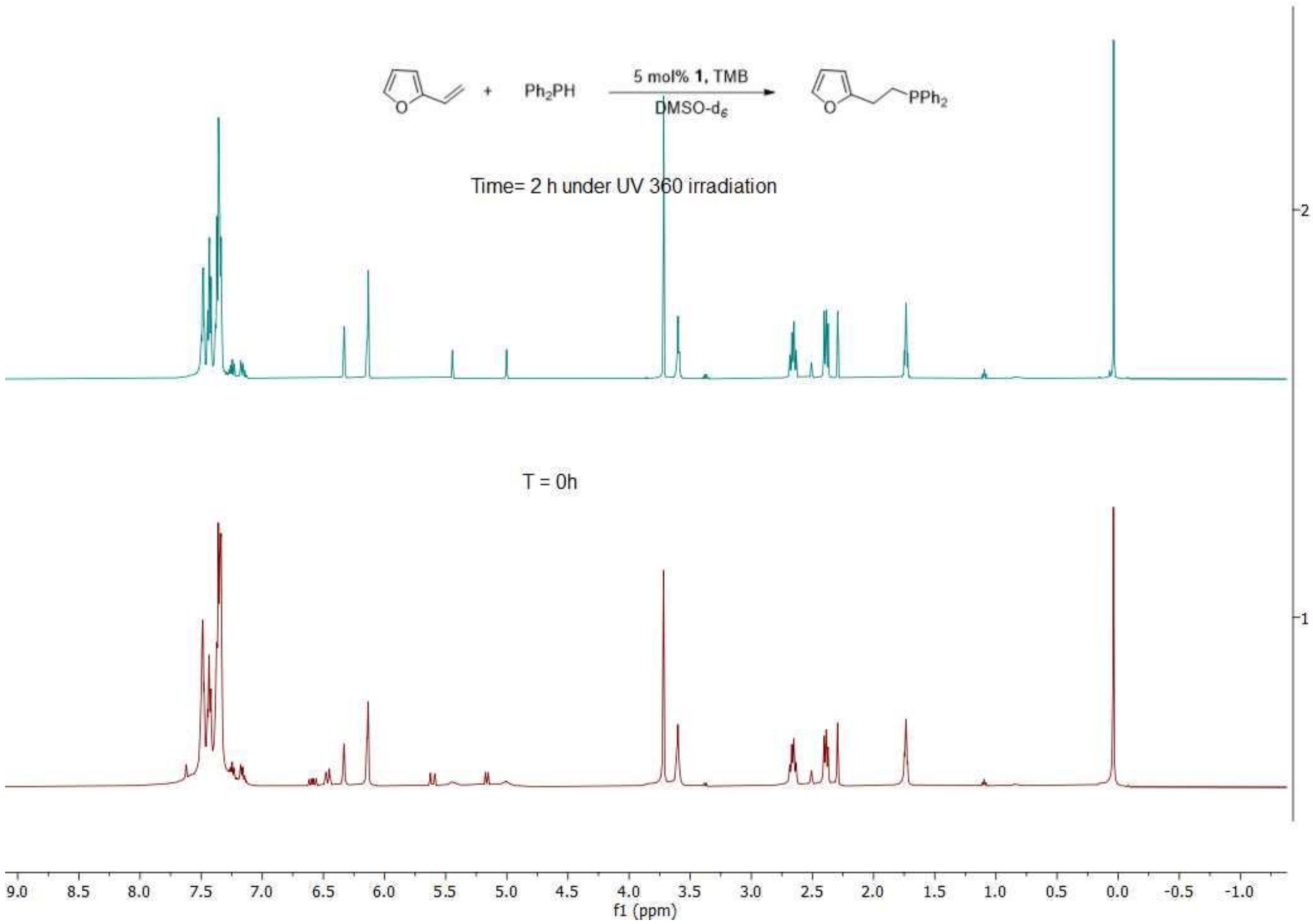

Figure S69: Hydrophosphination of 0.5 mmol 2-vinyl furan with equimolar Ph_2PH in DMSO-d_6 using 5 mol% **1** under 360 nm UV light - stacked - ^{1}H NMR

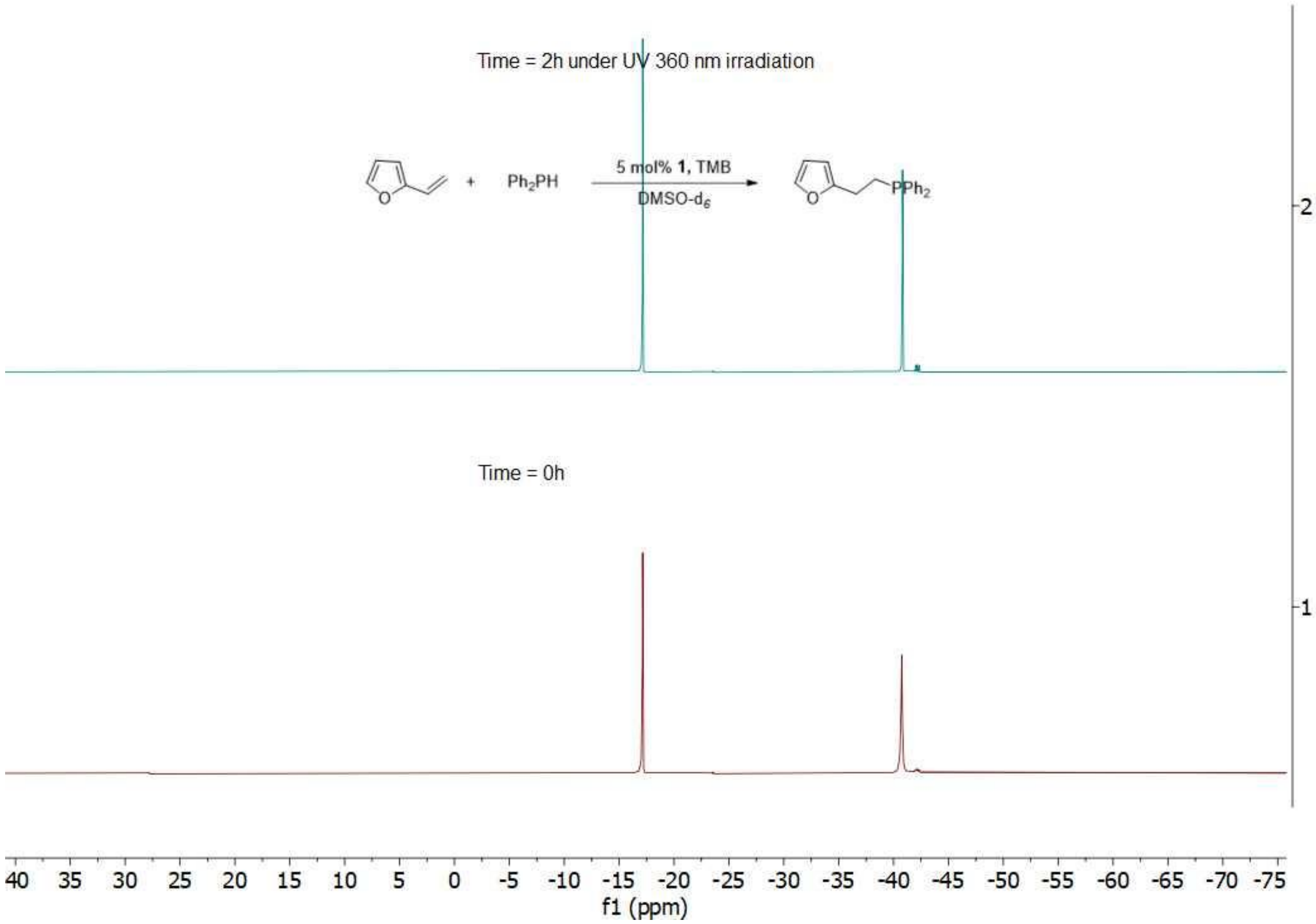

Figure S70: Hydrophosphination of 0.5 mmol 2-vinyl furan with equimolar Ph_2PH in DMSO-d_6 using 5 mol% **1** under 360 nm UV light - stacked ^1H NMR

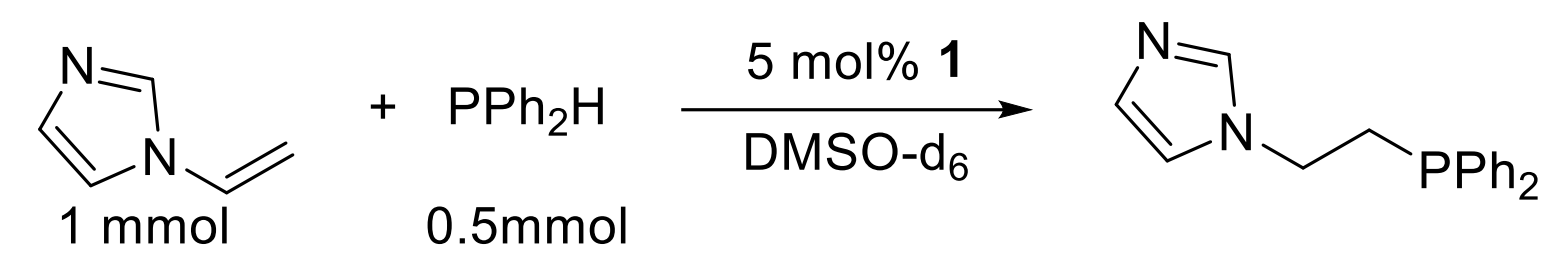

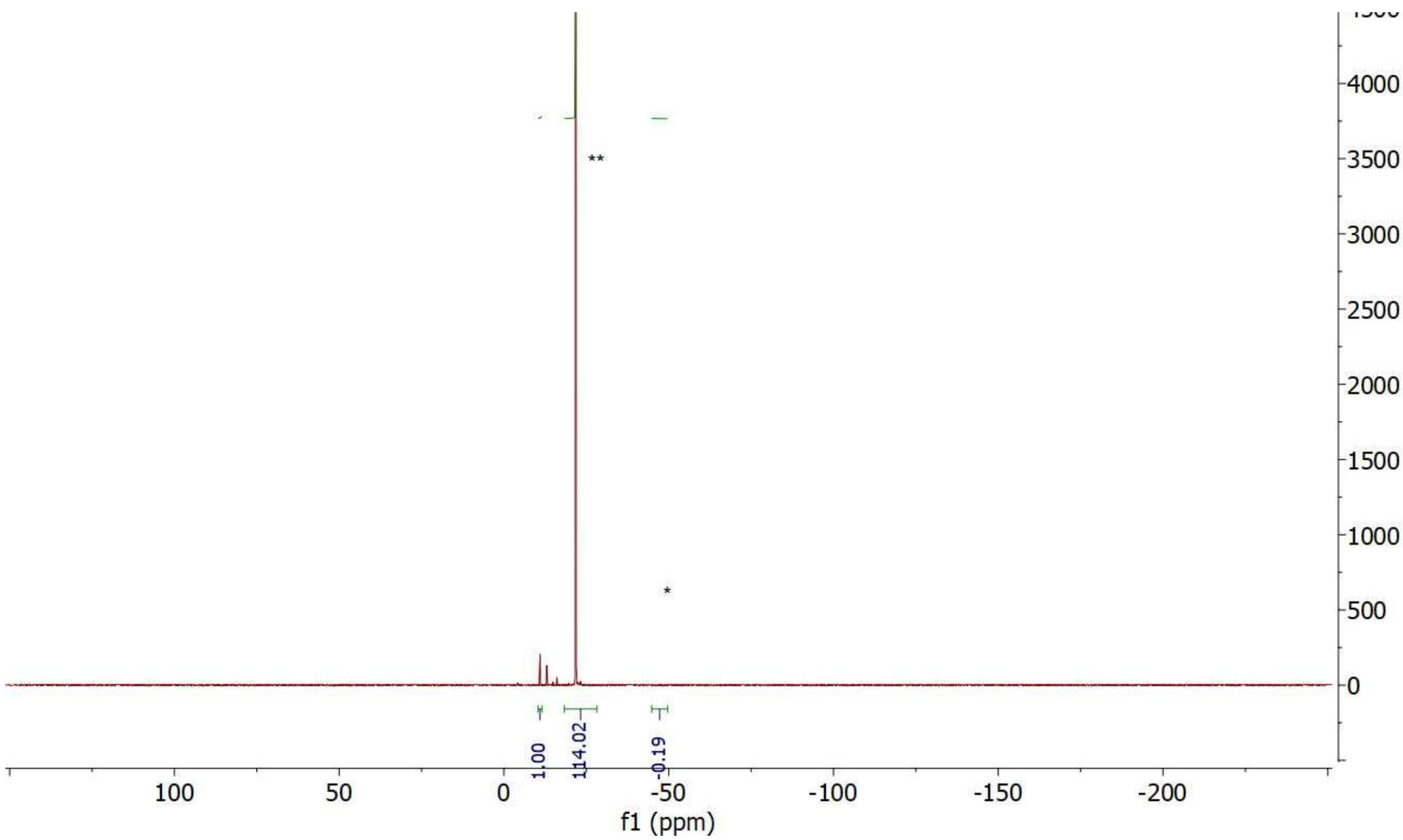

Figure S71: Hydrophosphination of 0.5 mmol 1-vinylimidazole with equimolar of Ph_2PH in DMSO-d_6 using 5 mol% **1** – 0 h – $^{31}P\{^1H\}$ NMR

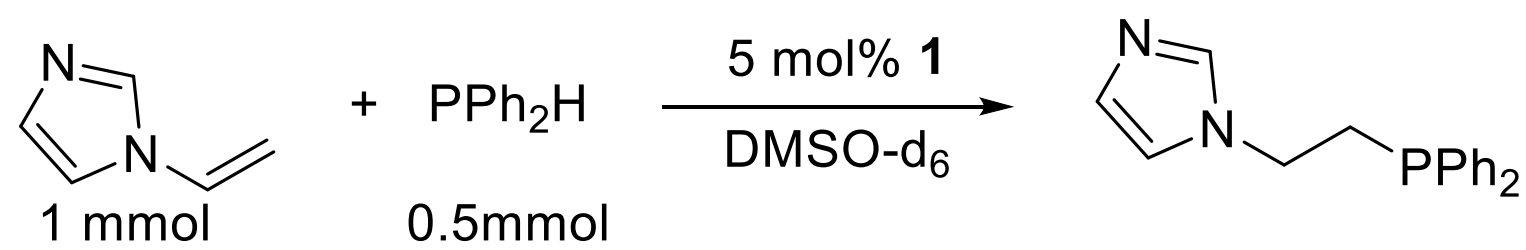

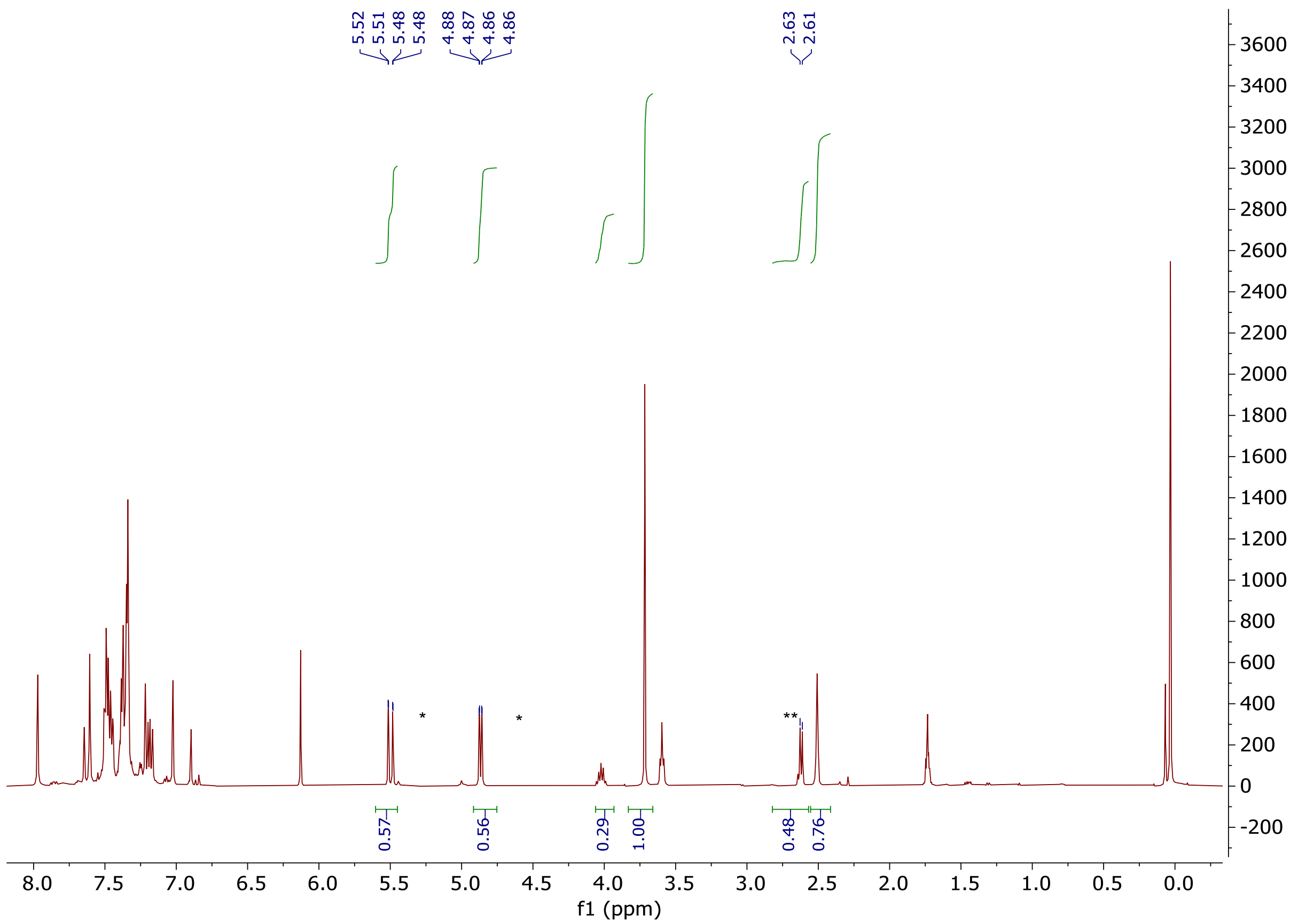

Figure S72: Hydrophosphination of 0.5 mmol 1-vinylimidazole with equimolar of Ph_2PH in DMSO-d_6 using 5 mol% **1** – 0 h - ^1H NMR

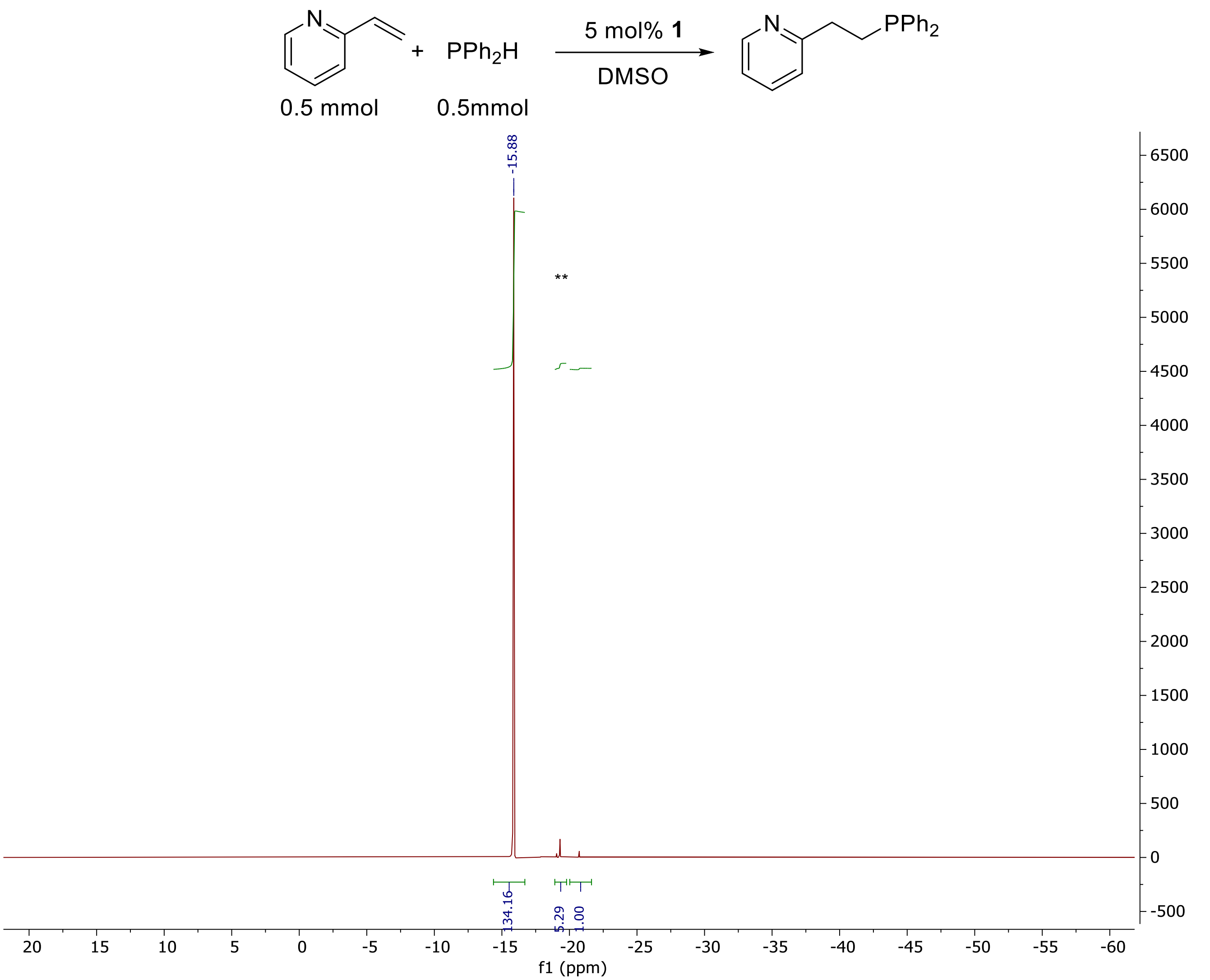

Figure S73: Hydrophosphination of 0.5 mmol 2-vinyl pyridine with equimolar of Ph_2PH in DMSO-d_6 using 5 mol% **1** under ambient conditions – 0 h - $^{31}P\{^1H\}$ NMR

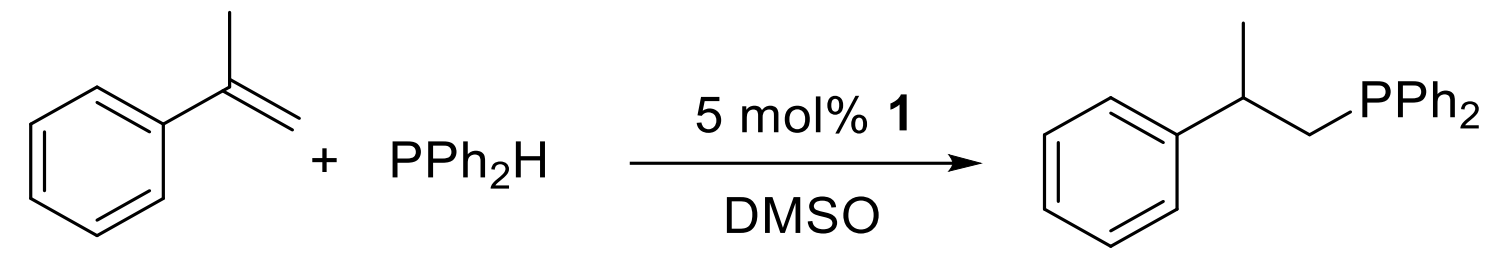

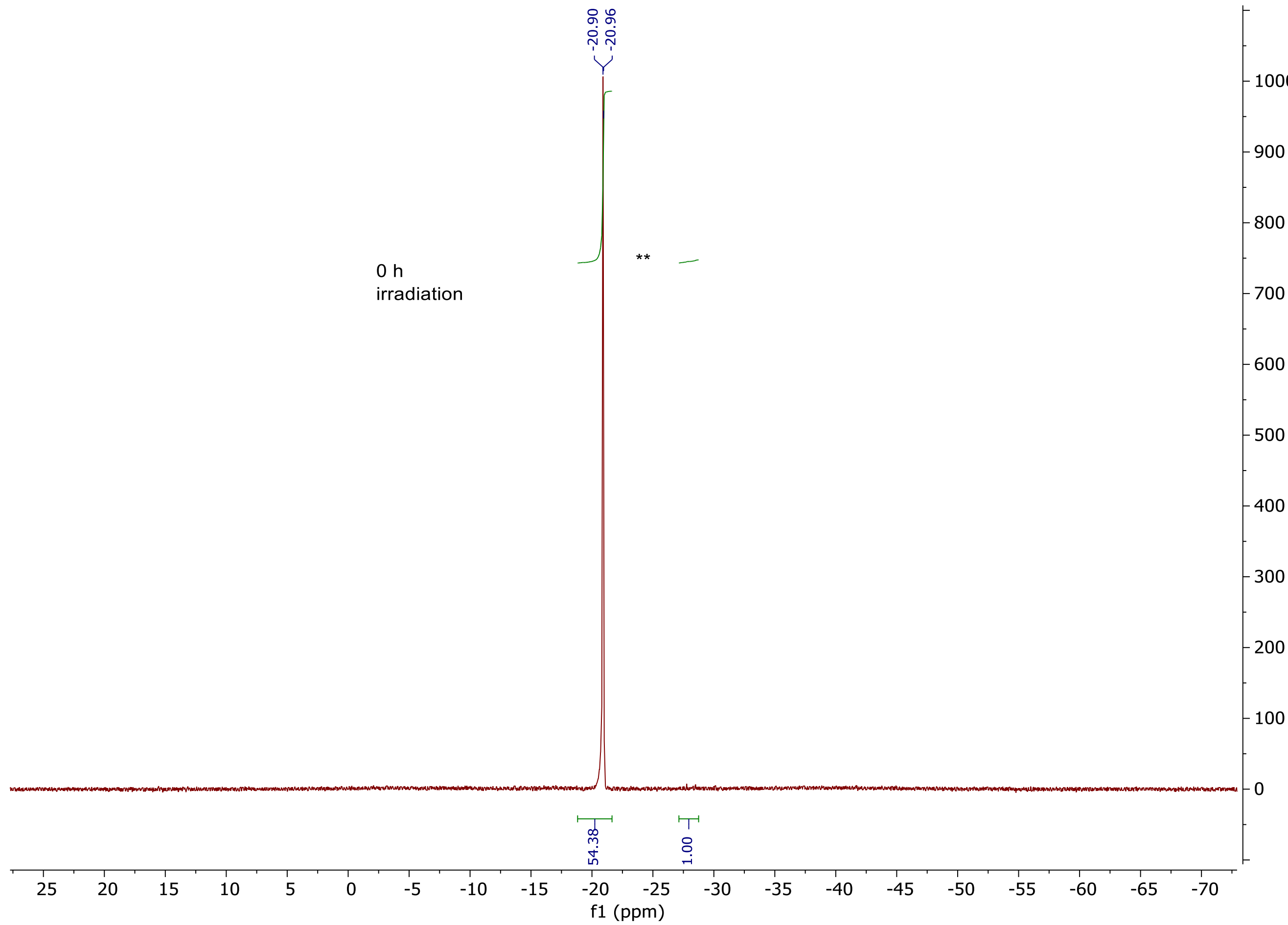

Figure S74: Hydrophosphination of 0.5 mmol α-methyl styrene with equimolar of Ph_2PH in DMSO-d_6 using 5 mol% **1** under- 0 h – $^{31}P\{^1H\}$ NMR

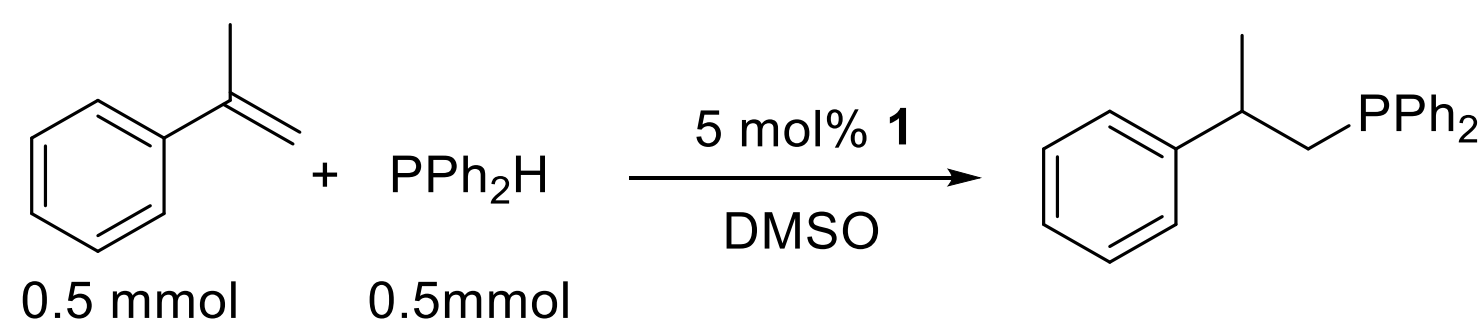

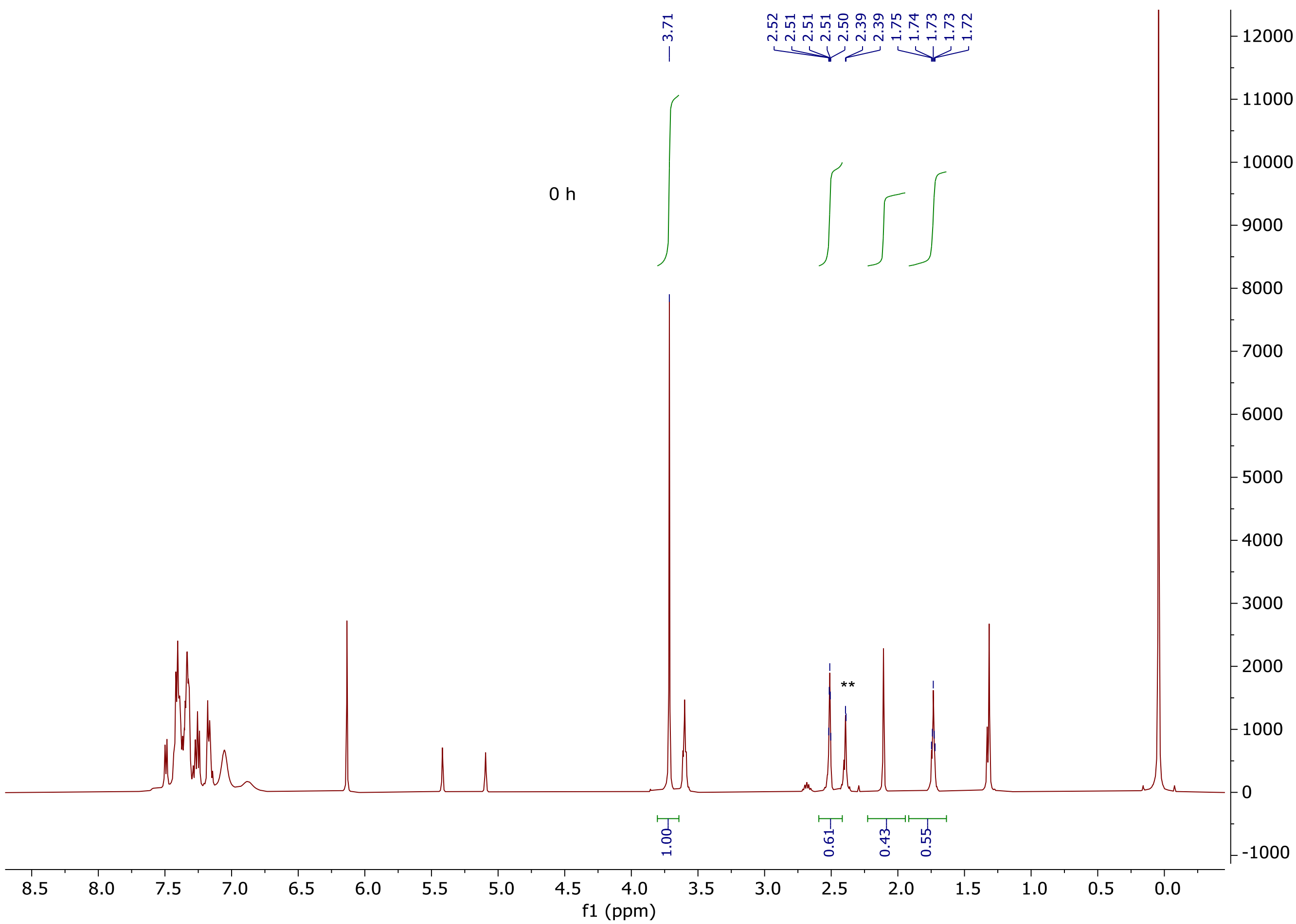

Figure S75: Hydrophosphination of 0.5 mmol α-methyl styrene with equimolar of Ph_2PH in DMSO-d_6 using 5 mol% **1** under - 0 h– ^{1}H NMR

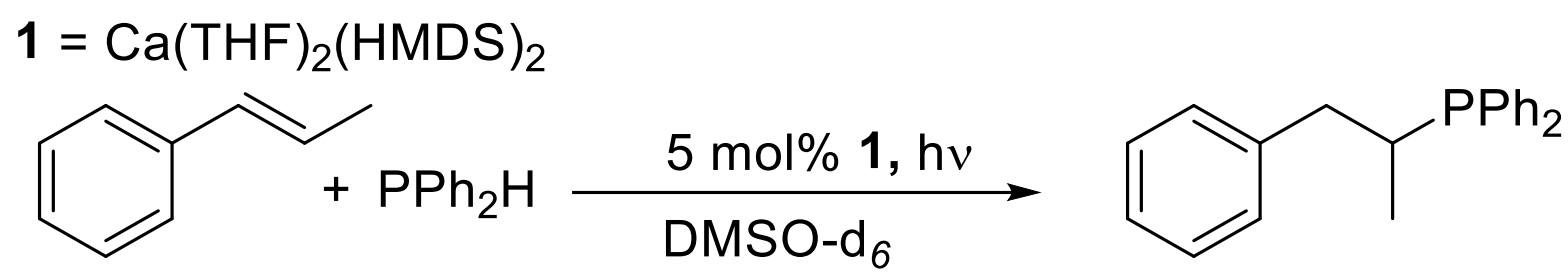

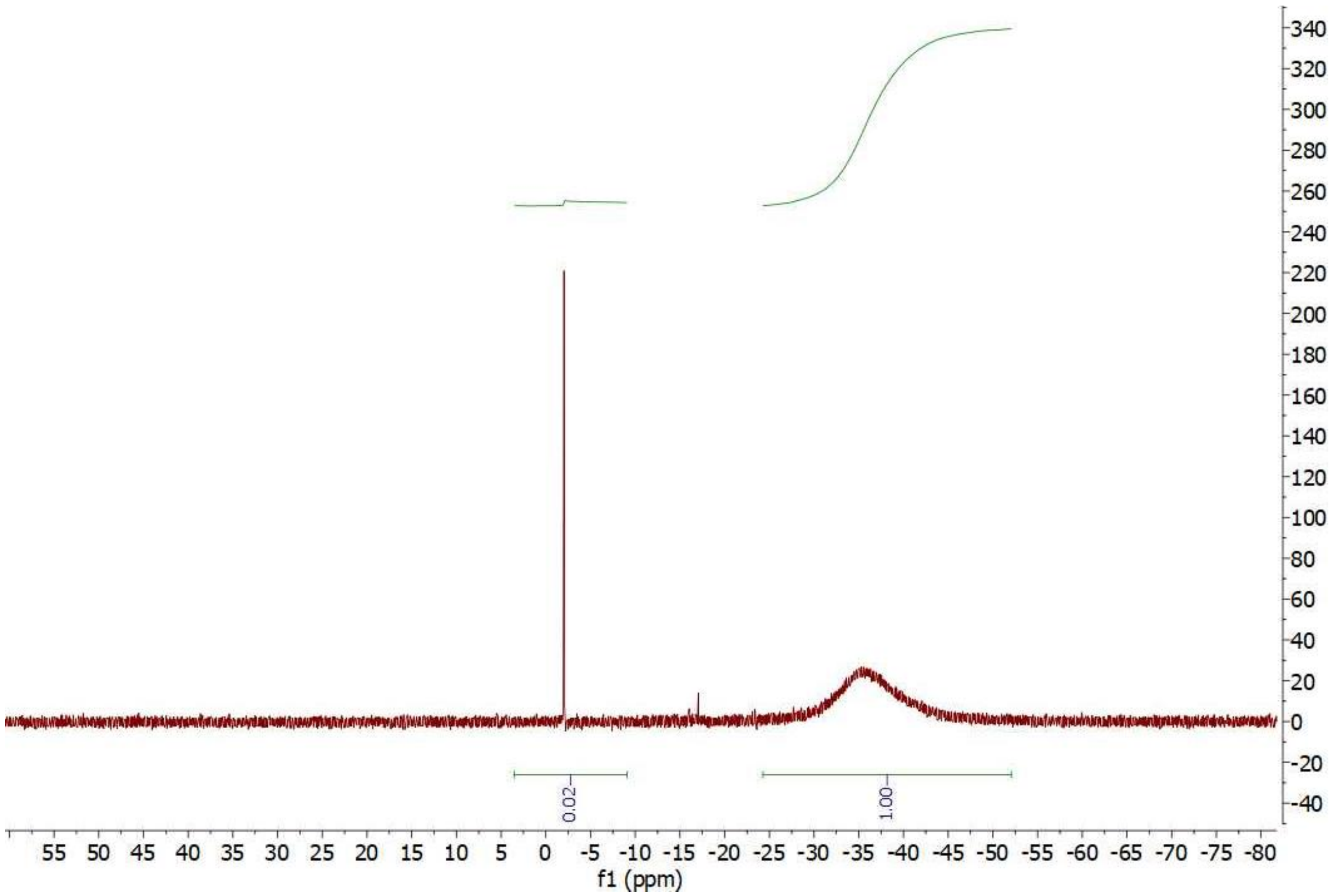

Figure S76 : Hydrophosphination of 0.5 mmol β-methyl styrene with equimolar of Ph_2PH in DMSO-d_6 using 5 mol% **1** under UV 360 nm irradiation - 0 h– $^{31}P\{^1H\}$ NMR

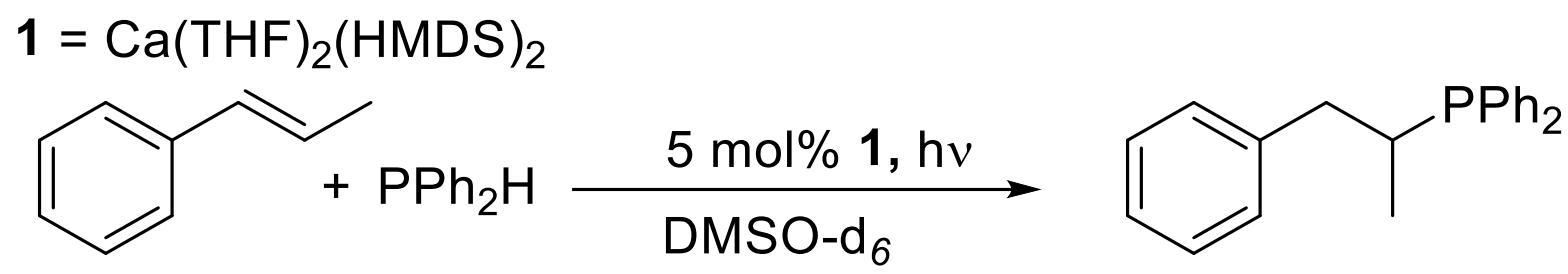

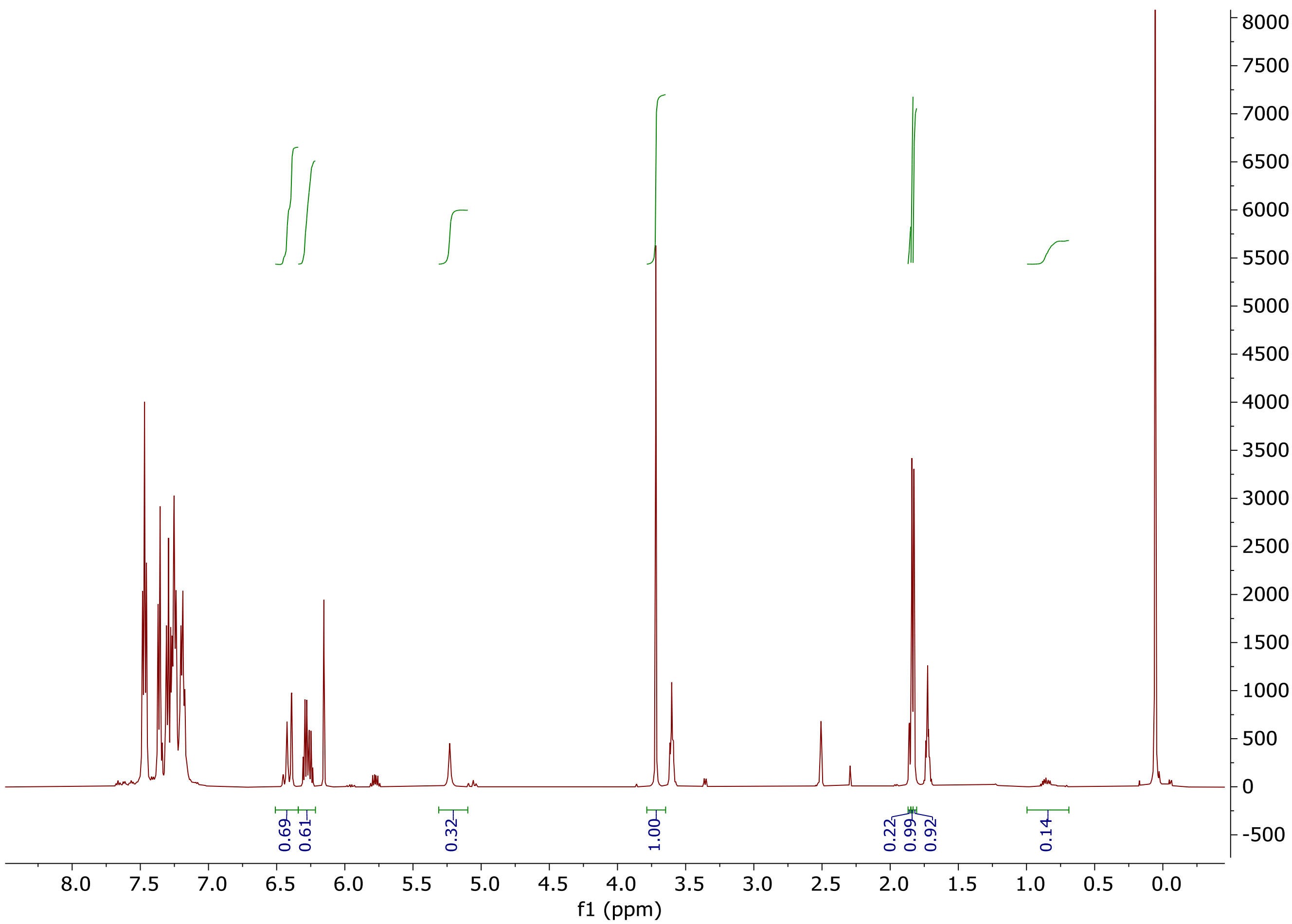

Figure S77: Hydrophosphination of 0.5 mmol β-methyl styrene with equimolar of Ph_2PH in DMSO-d_6 using 5 mol% **1** under UV 360 nm irradiation - 0 h– ^1H NMR

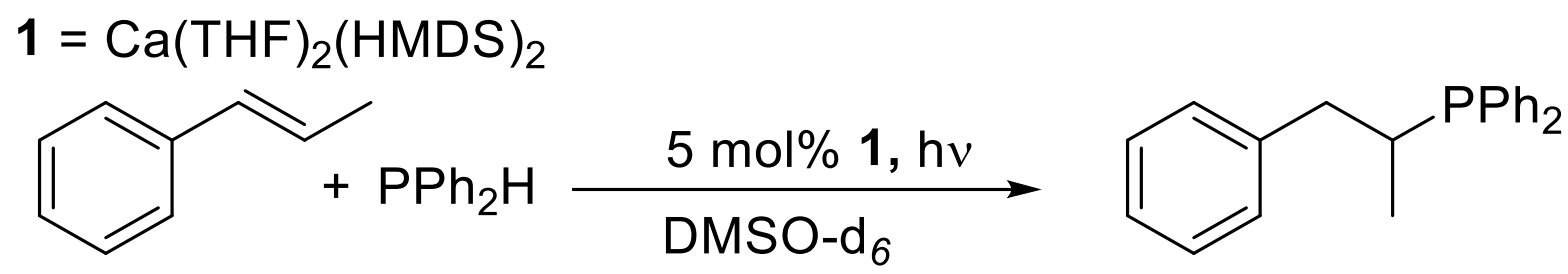

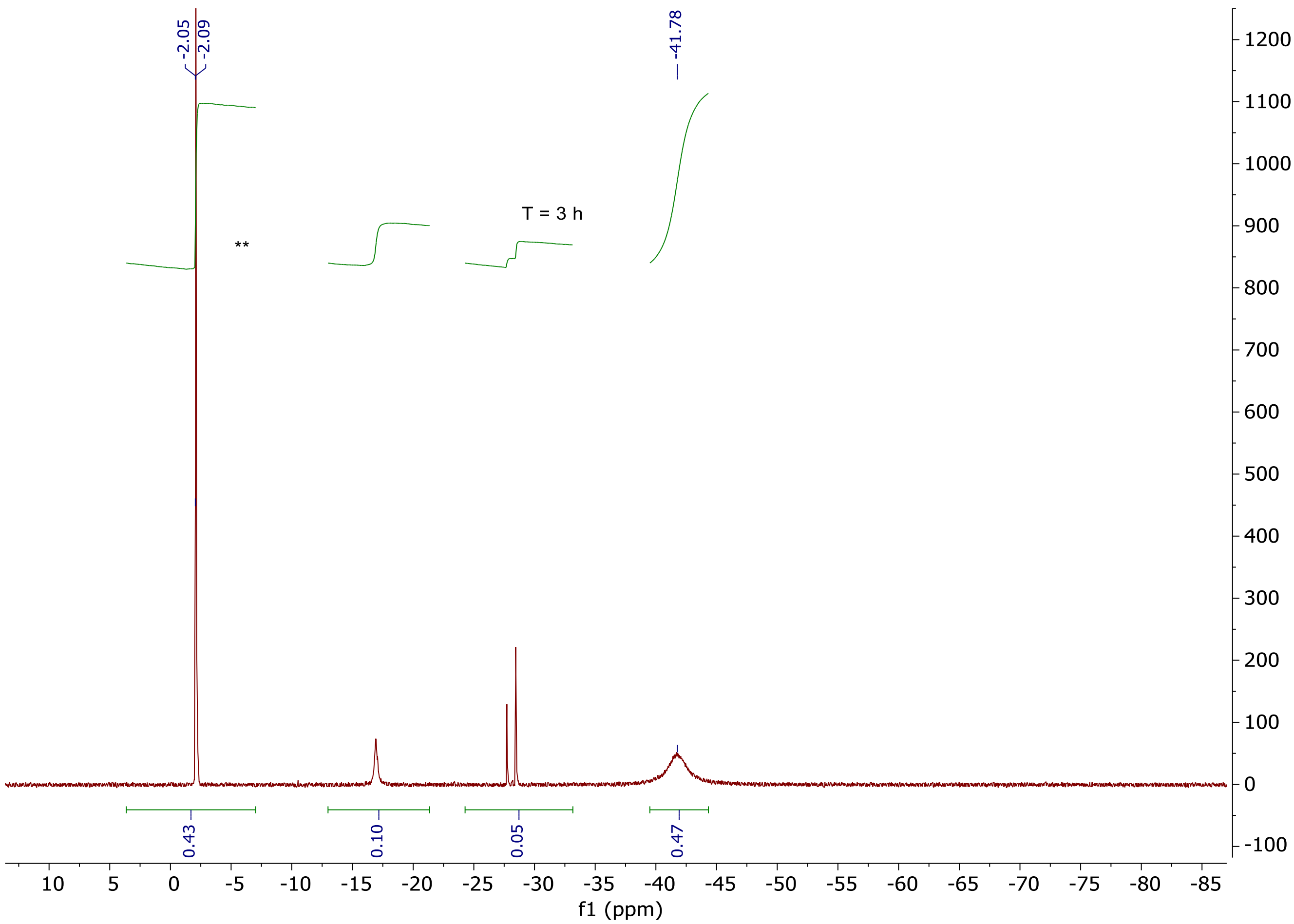

Figure S78: Hydrophosphination of 0.5 mmol β-methyl styrene with equimolar of Ph_2PH in DMSO-d_6 using 5 mol% **1** under UV 360 nm irradiation - 3 h– $^{31}P\{^{1}H\}$ NMR

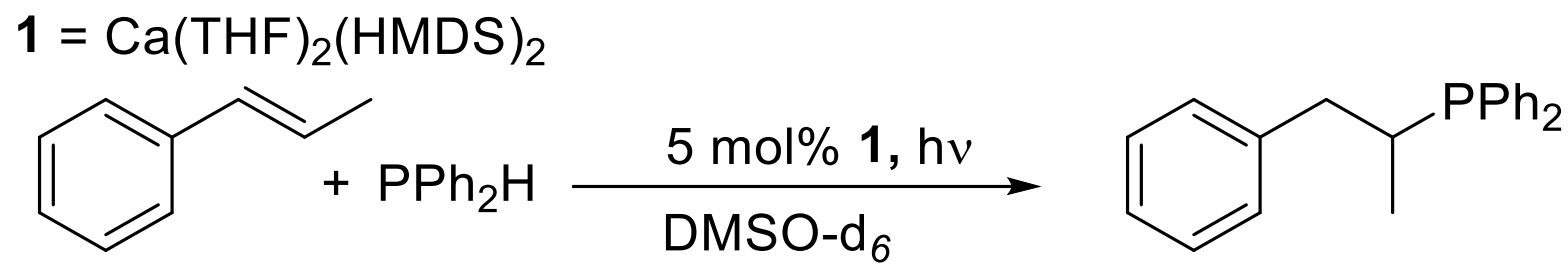

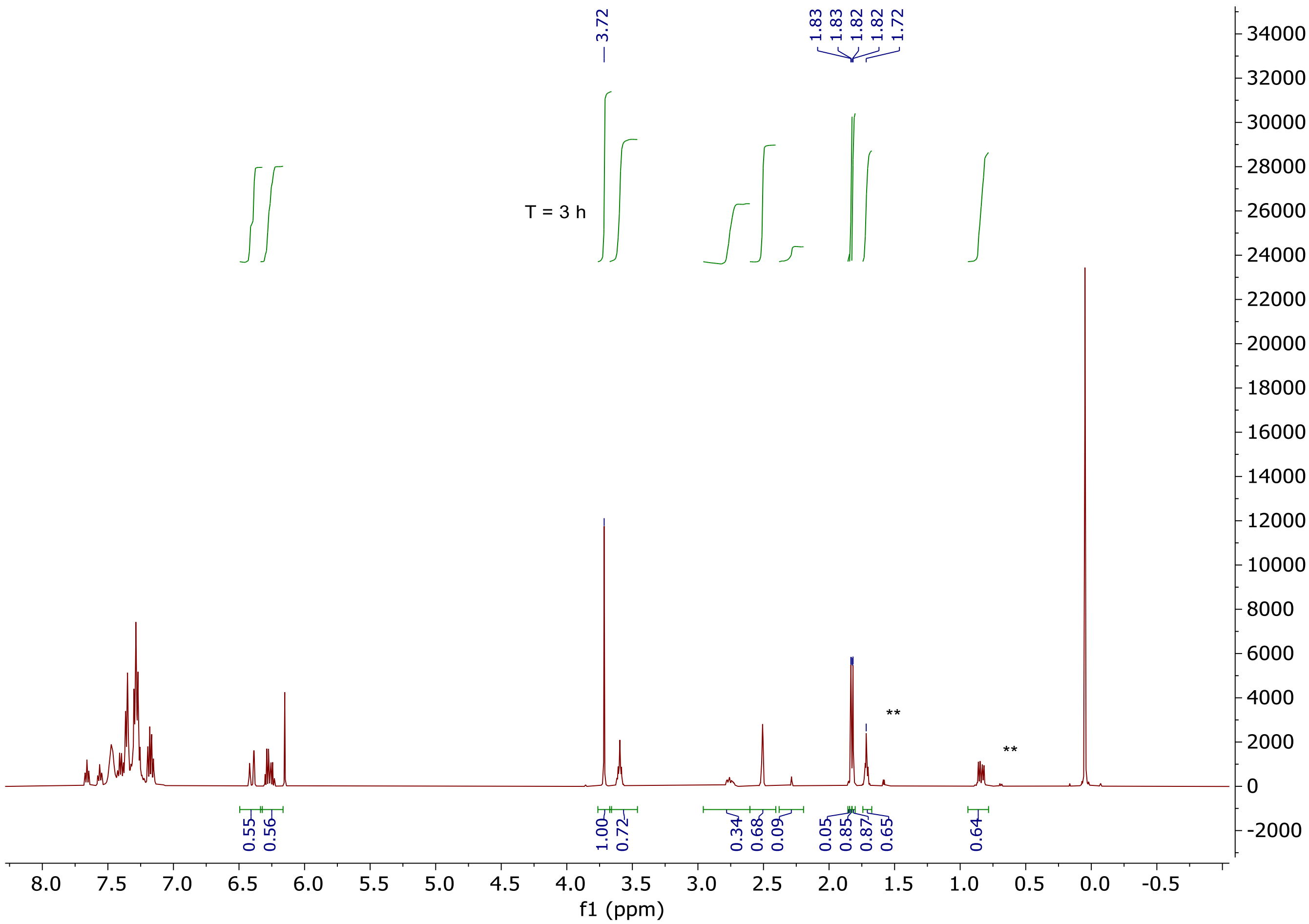

Figure S79:
Hydrophosphination of 0.5 mmol β-methyl styrene with equimolar of Ph_2PH in DMSO-d_6 using 5 mol% **1** under UV 360 nm irradiation - 3 h – $^{31}P\{^1H\}$ NMR

1 = $Ca(THF)_2(HMDS)_2$

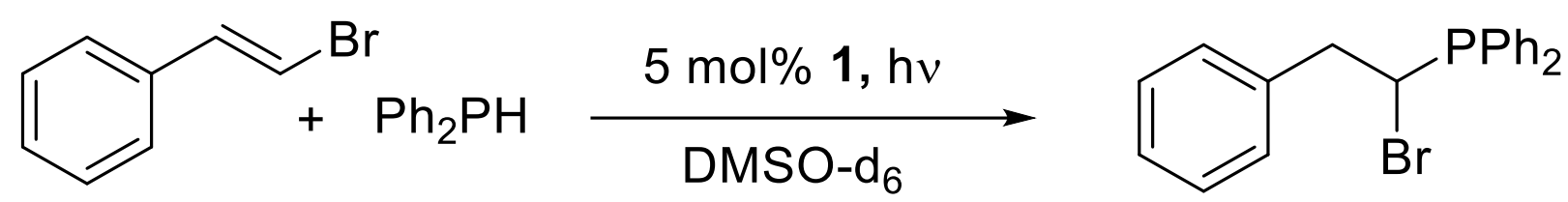

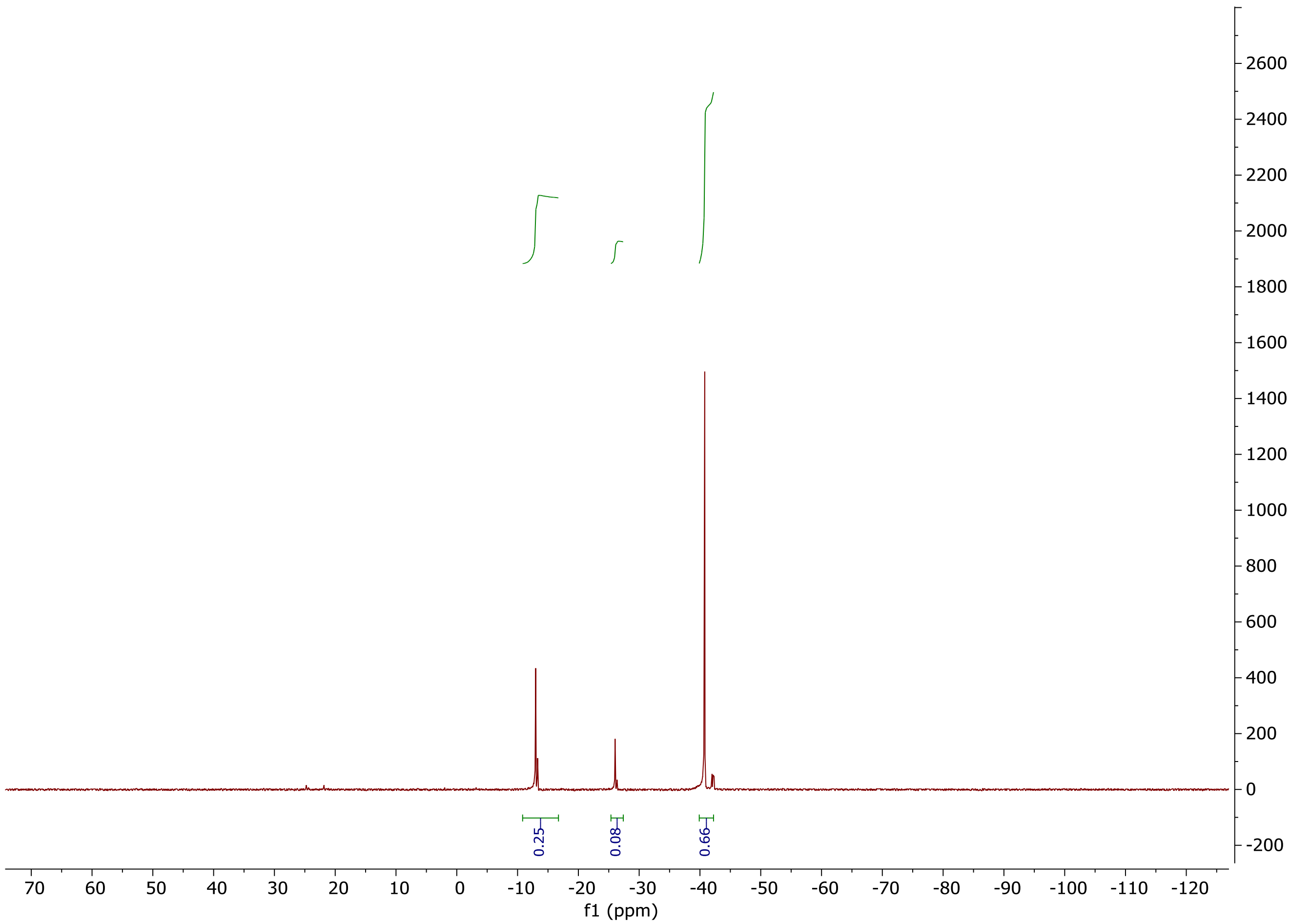

Figure S80: Hydrophosphination of 0.5 mmol β – bromo styrene with equimolar of Ph_2PH in DMSO-d_6 using 5 mol% **1** under UV 360 nm irradiation – 20 h – $^{31}P\{^1H\}$ NMR

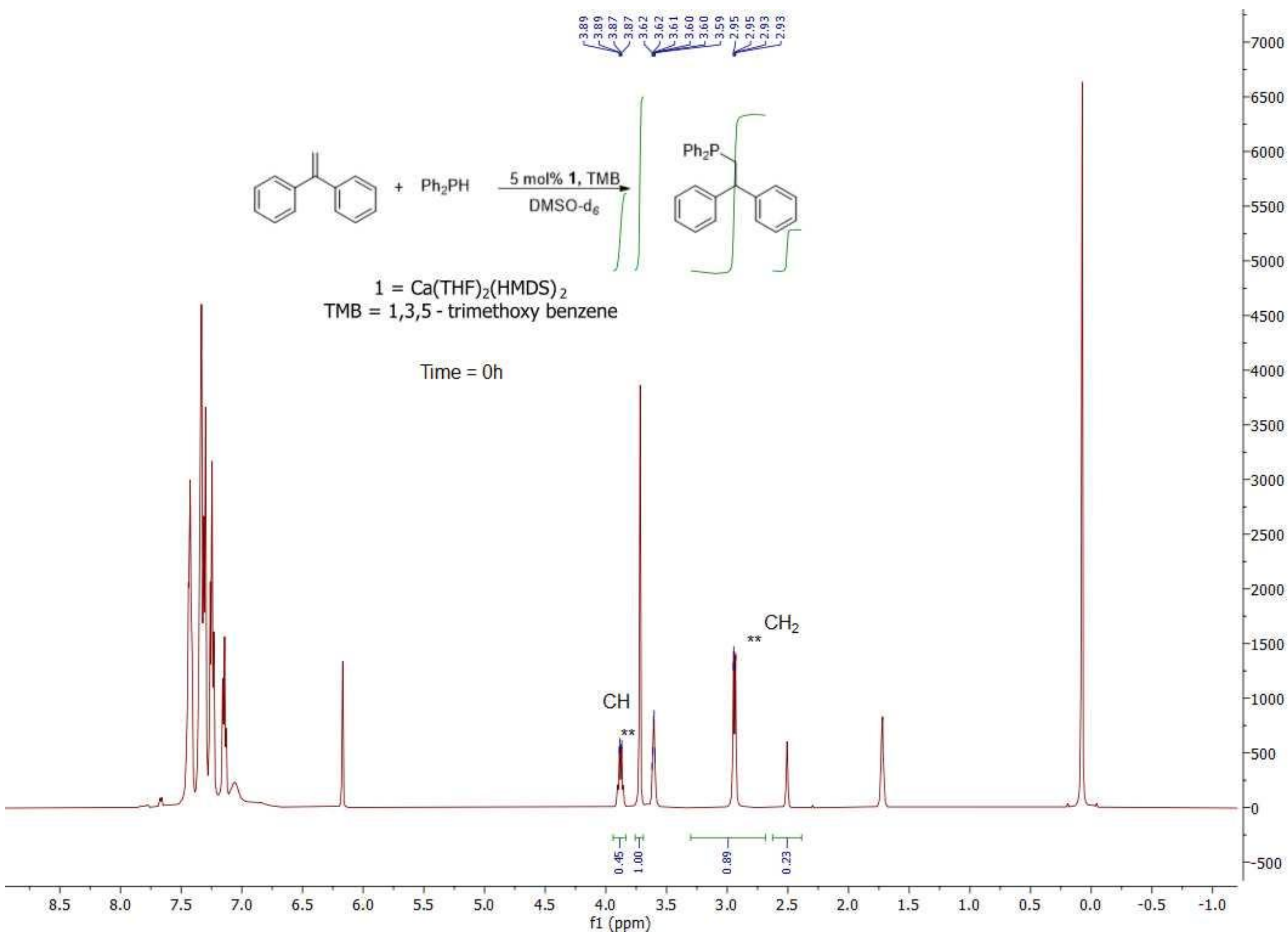

Figure S81: Hydrophosphination of 1,1-diphenyl ethene with equimolar Ph_2PH in DMSO-d_6 using 5 mol% **1** under ambient condition- 0 h – ^{1}H NMR

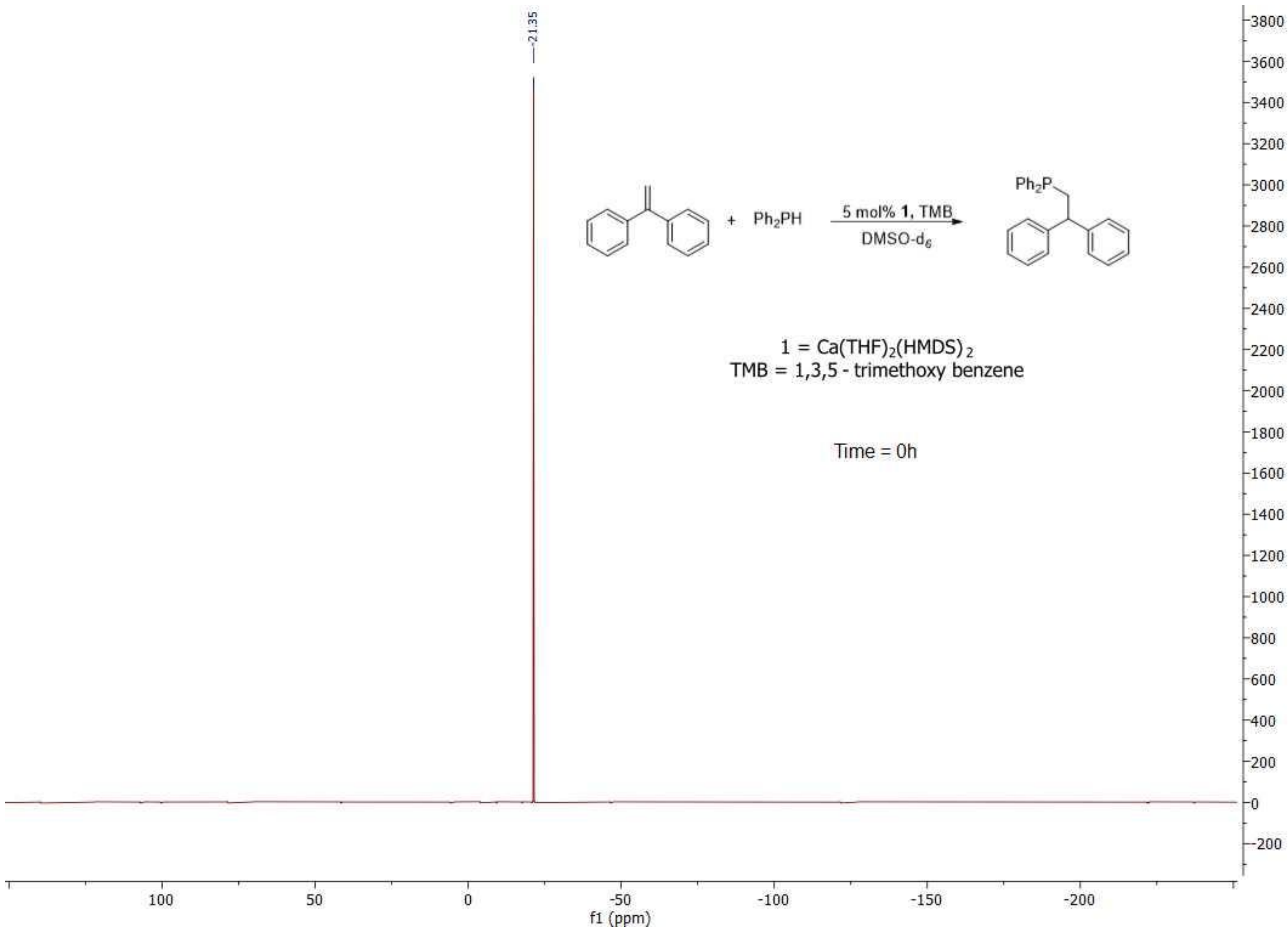

Figure S82: Hydrophosphination of 1,1-diphenyl ethene with equimolar of Ph_2PH in DMSO-d_6 using 5 mol% **1** under ambient condition- 0 h – $^{31}P\{^1H\}$ NMR

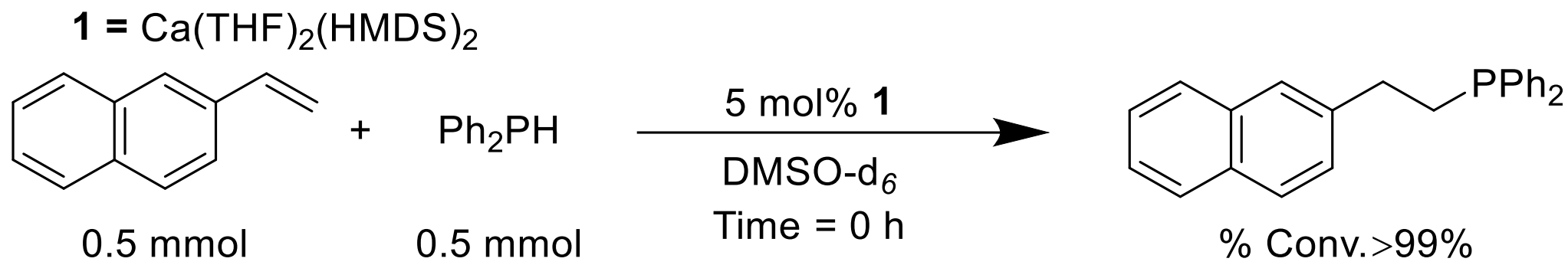

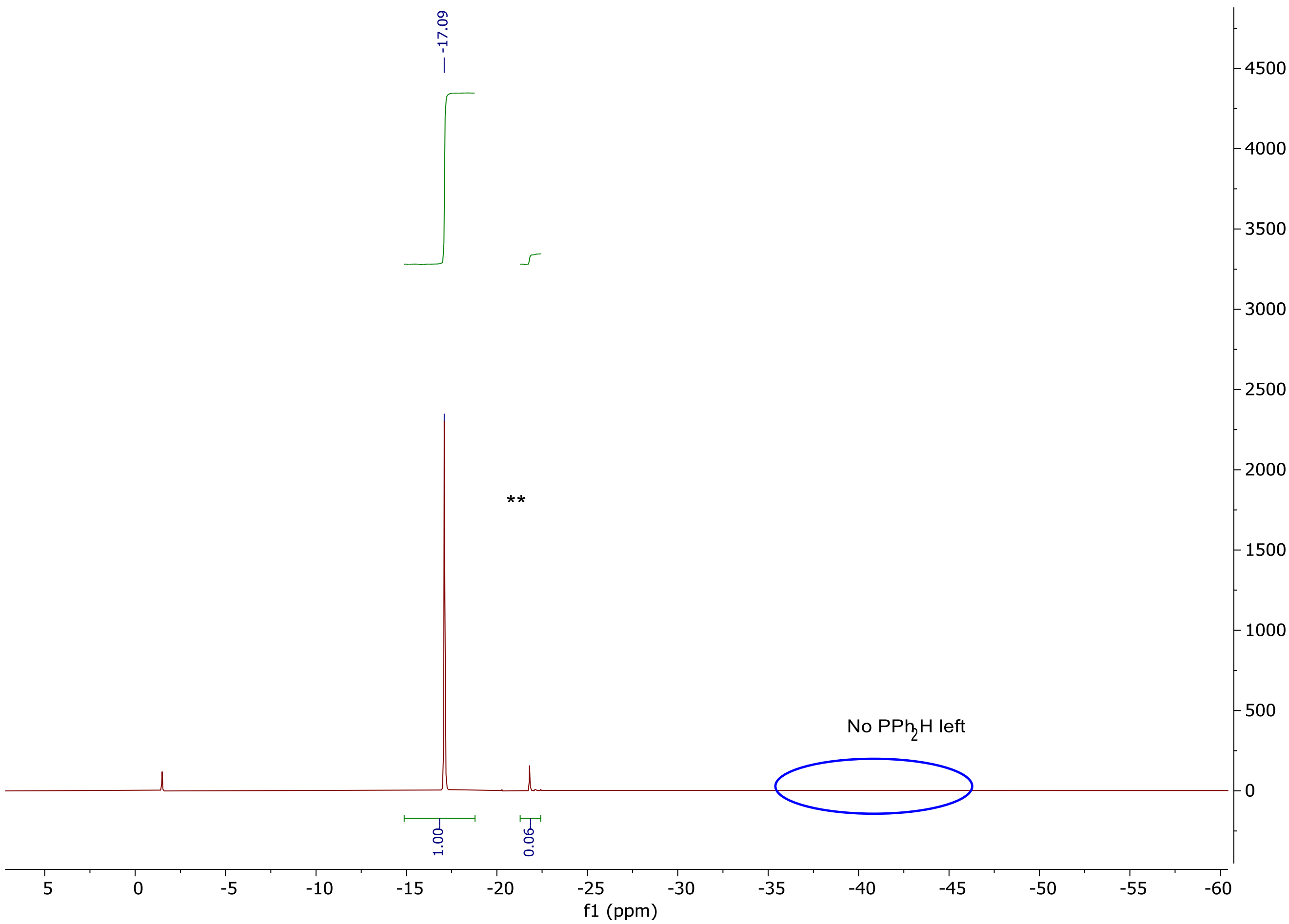

Figure S83: Hydrophosphination of 2-vinyl naphthalene with Ph_2PH in DMSO-d_6 using 5 mol% **1,** and 1,3,5 – trimethoxybenzene as internal standard under ambient condition - 0 h – $^{31}P\{^1H\}$ NMR

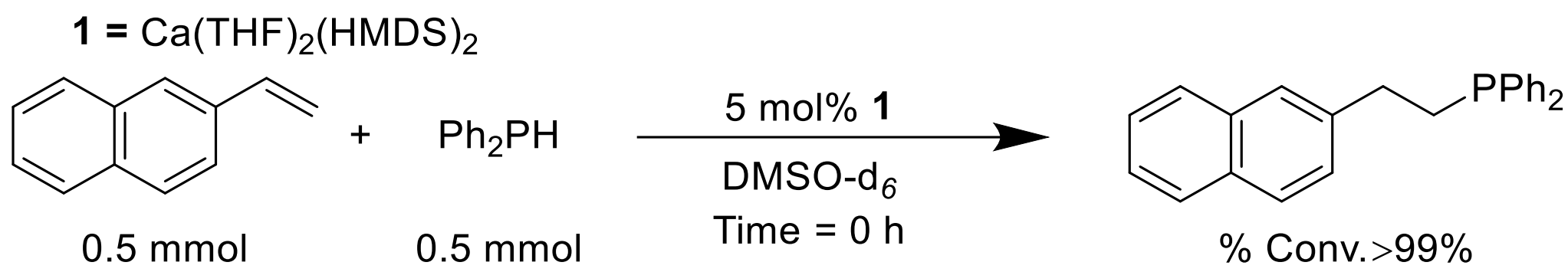

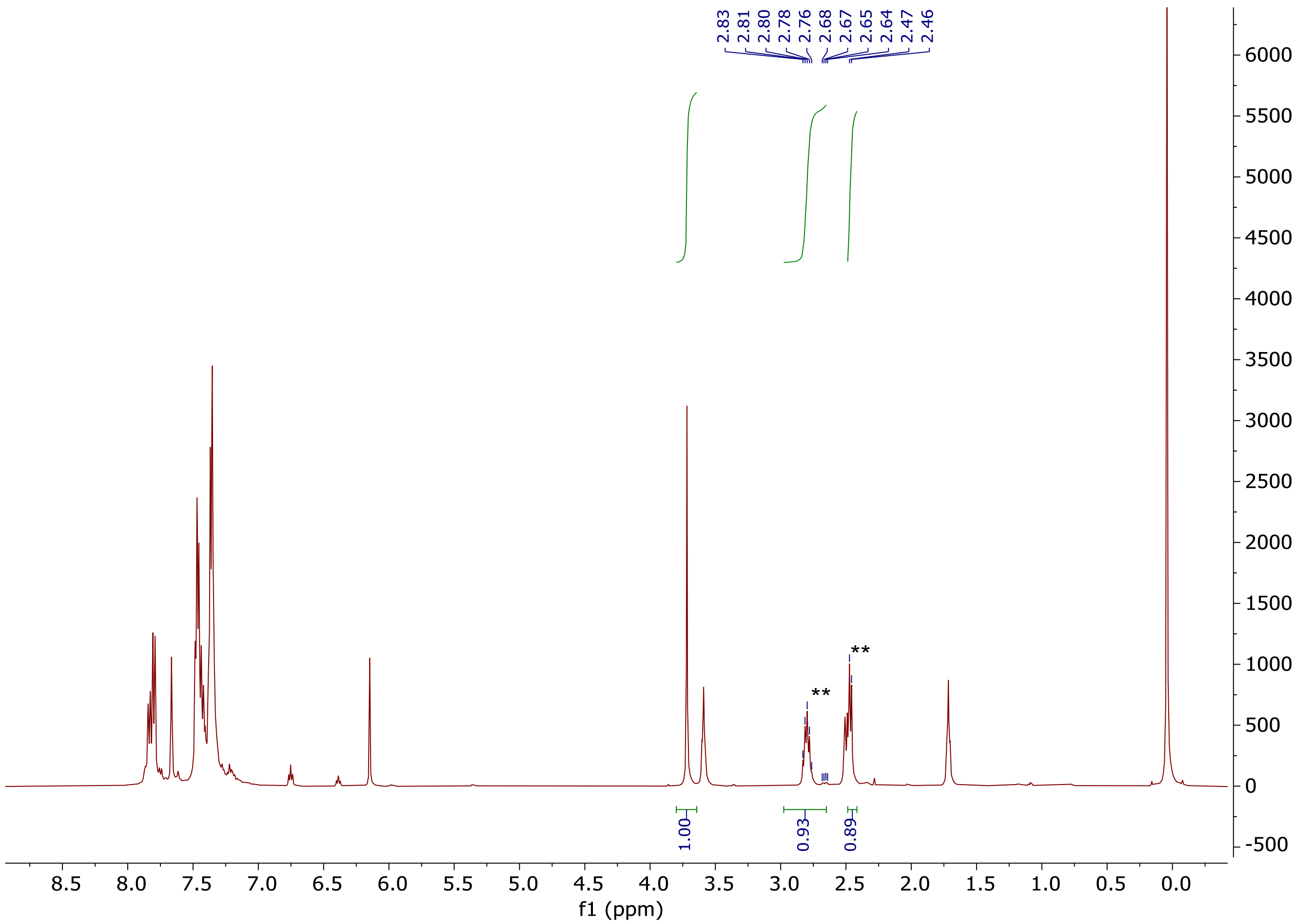

Figure S84: Hydrophosphination of 2-vinyl naphthalene with Ph_2PH in DMSO-d_6 using 5 mol% **1,** and 1,3,5 – trimethoxybenzene as internal standard under ambient condition- 0 h – ^{1}H NMR

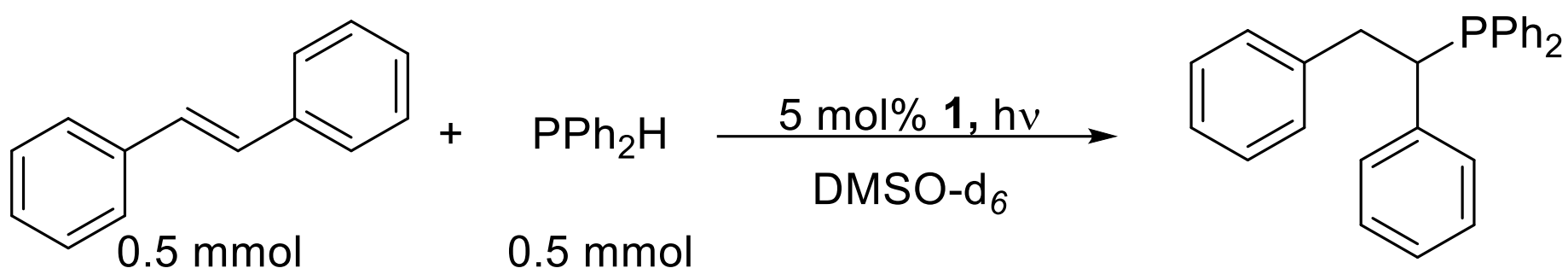

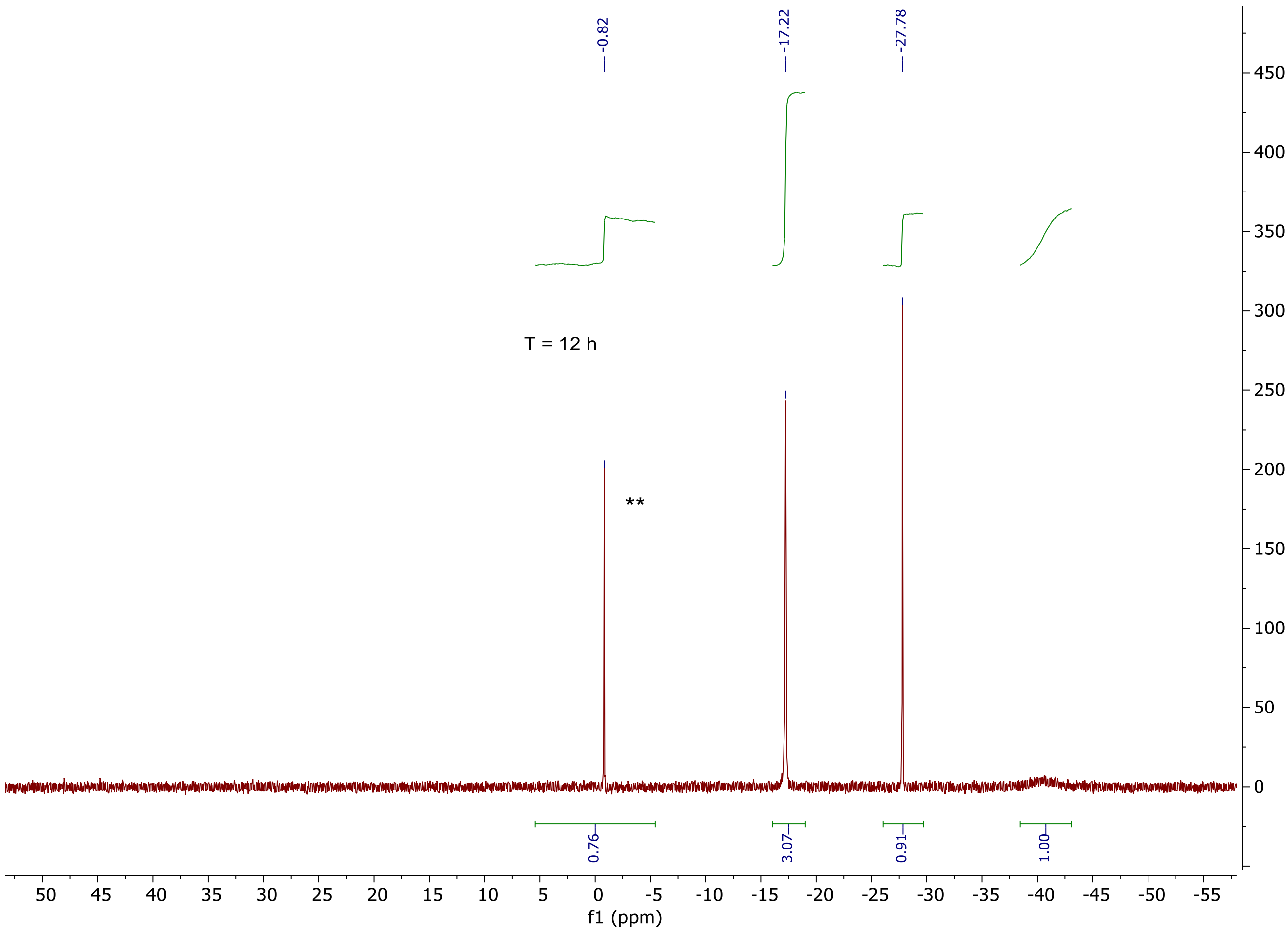

Figure S85: Hydrophosphination of 0.5 mmol trans stilbene with equimolar of Ph_2PH in DMSO-d_6 using 5 mol% **1** under UV 360 nm irradiation – 12 h – $^{31}P\{^1H\}$ NMR

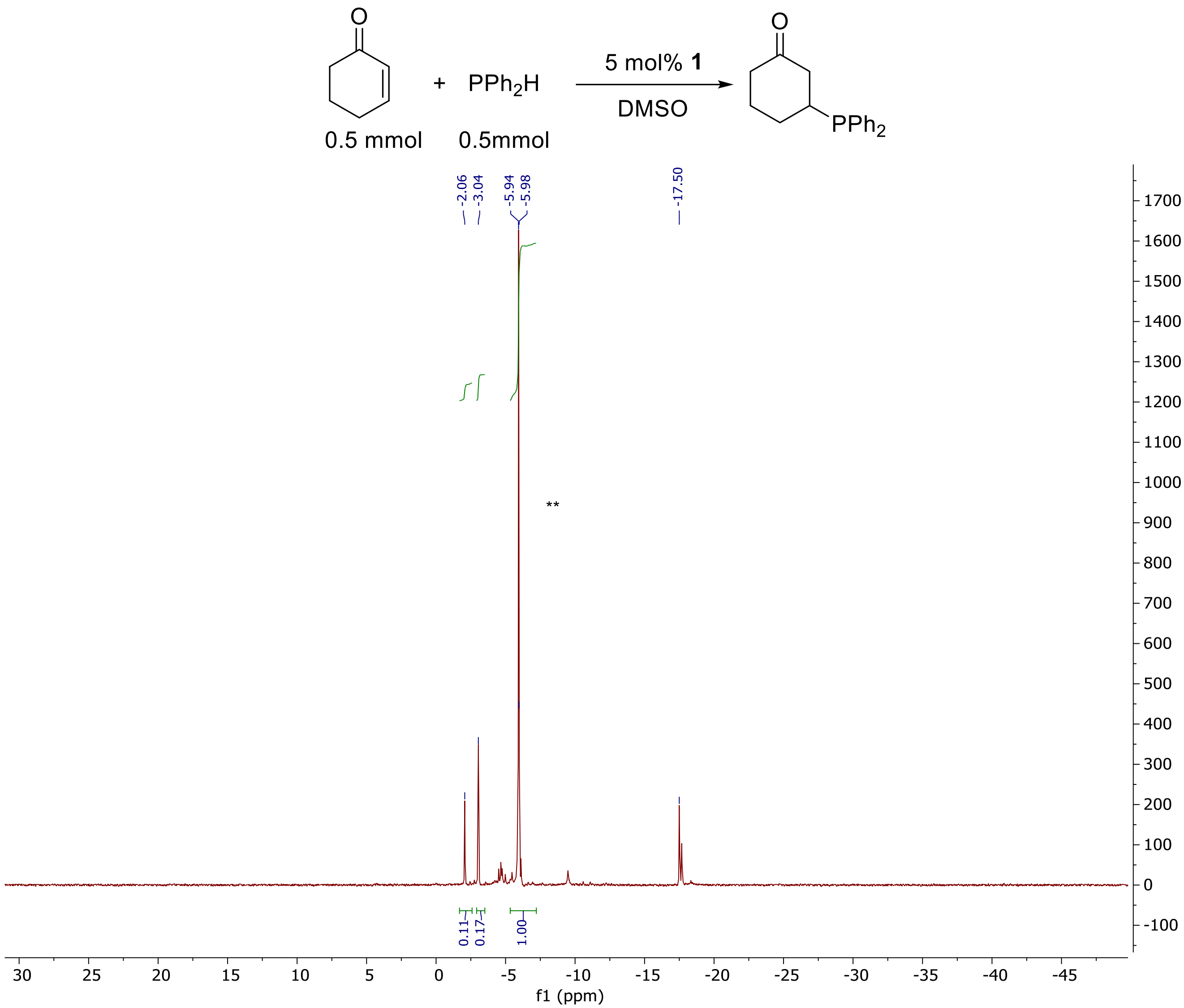

Figure S86: Hydrophosphination of 0.5 mmol cyclohexenone with equimolar of Ph_2PH in DMSO-d_6 using 5 mol% **1** under– 0 h – $^{31}P\{^1H\}$ NMR

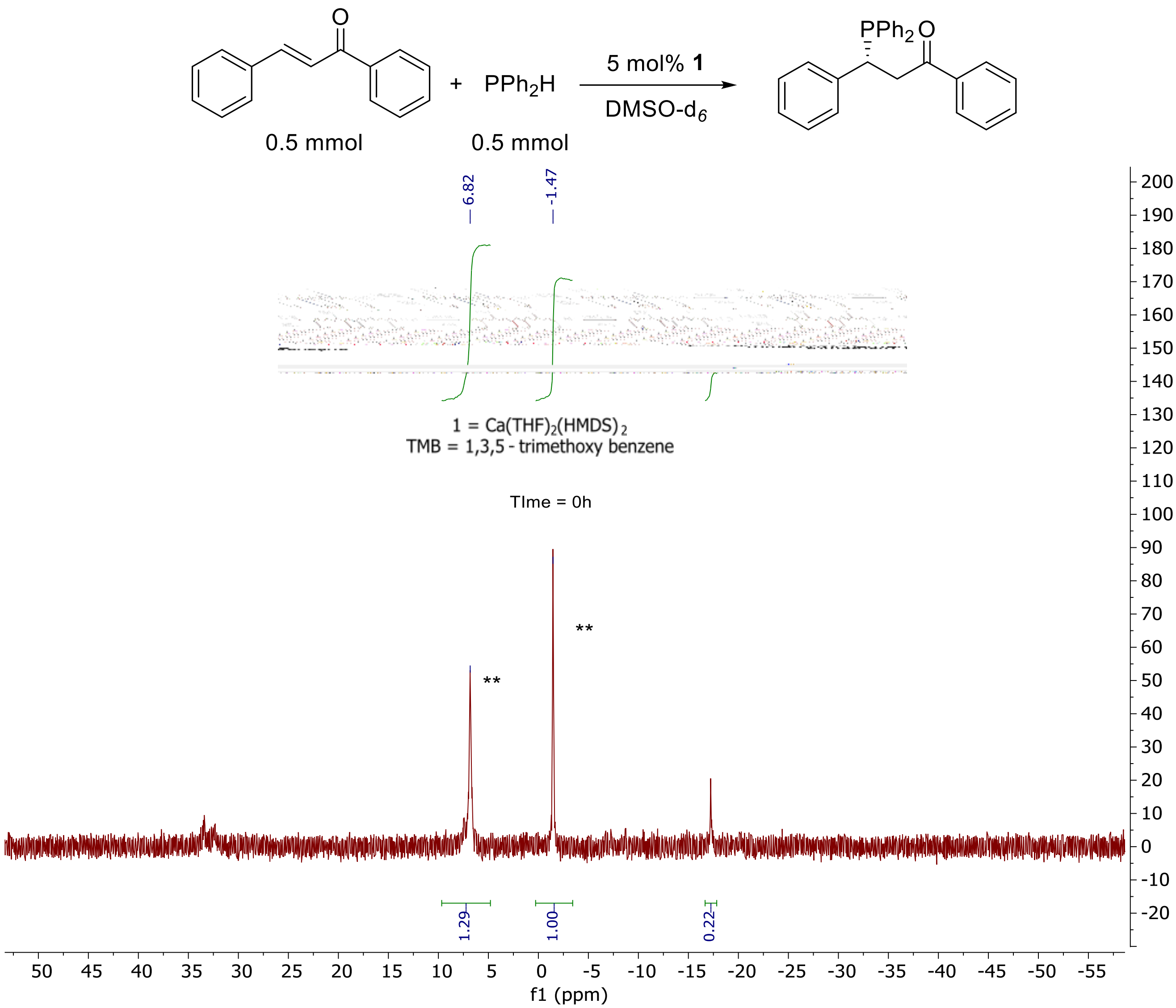

Figure S87: Hydrophosphination of 0.5 mmol trans-chalcone with equimolar of Ph_2PH in DMSO-d_6 using 5 mol% **1** under– 0 h – $^{31}P\{^1H\}$ NMR

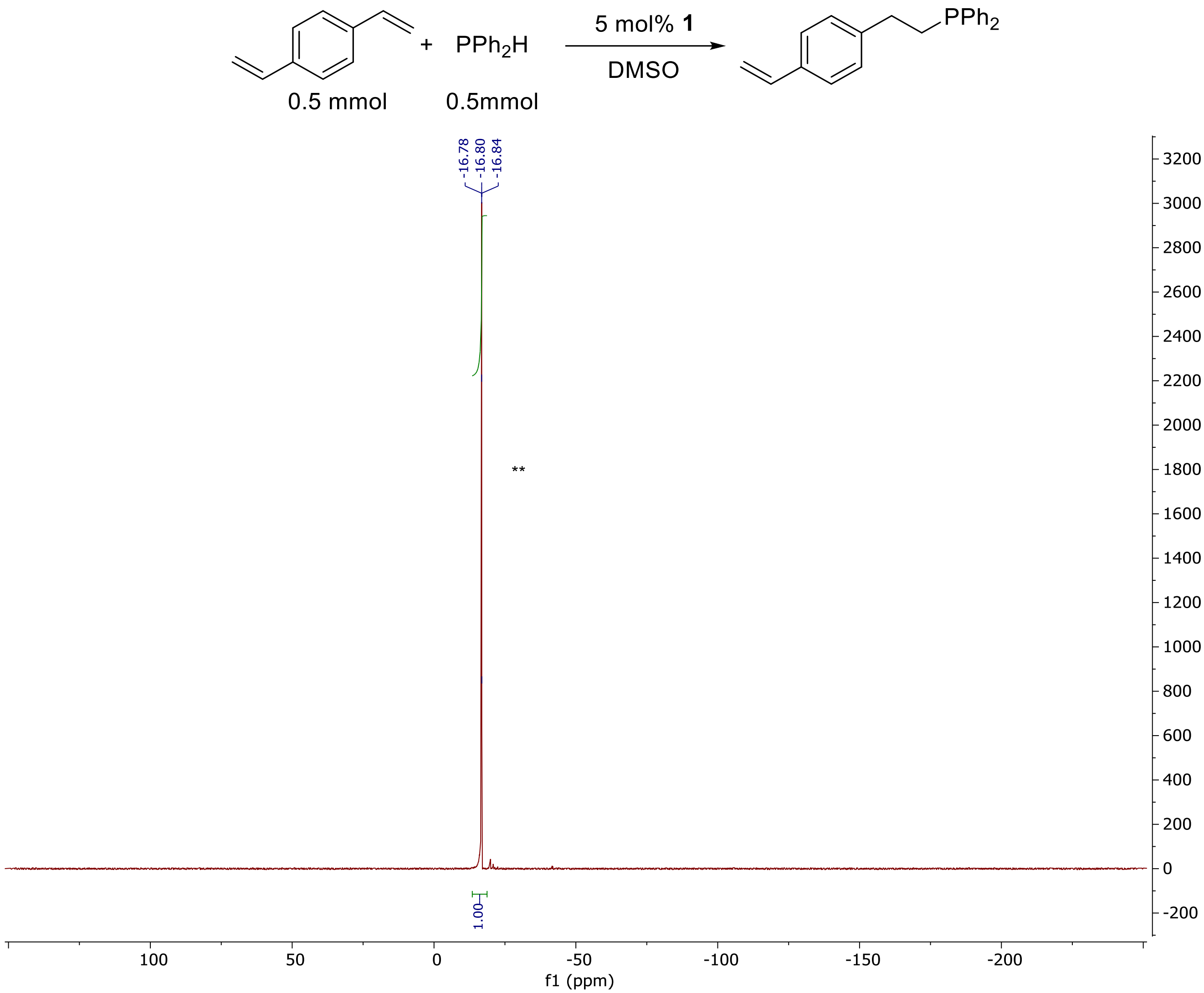

Figure S88: Hydrophosphination of 0.5 mmol 4-vinyl styrene with equimolar of Ph_2PH in DMSO-d_6 using 5 mol% **1** - 0 h – $^{31}P\{^1H\}$ NMR

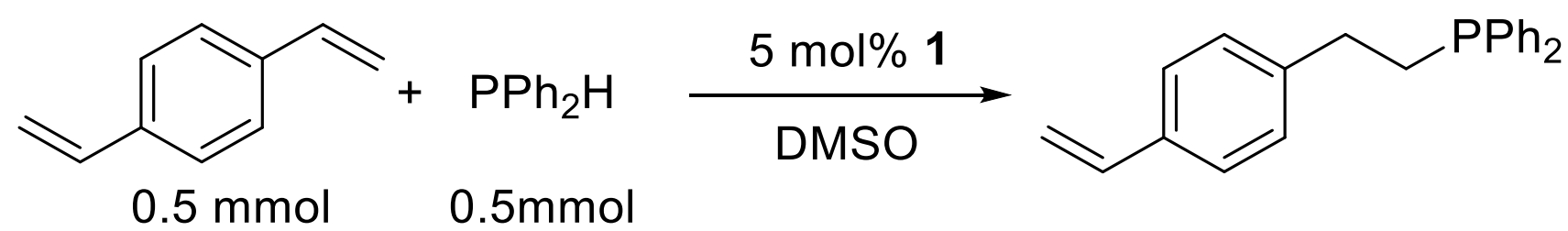

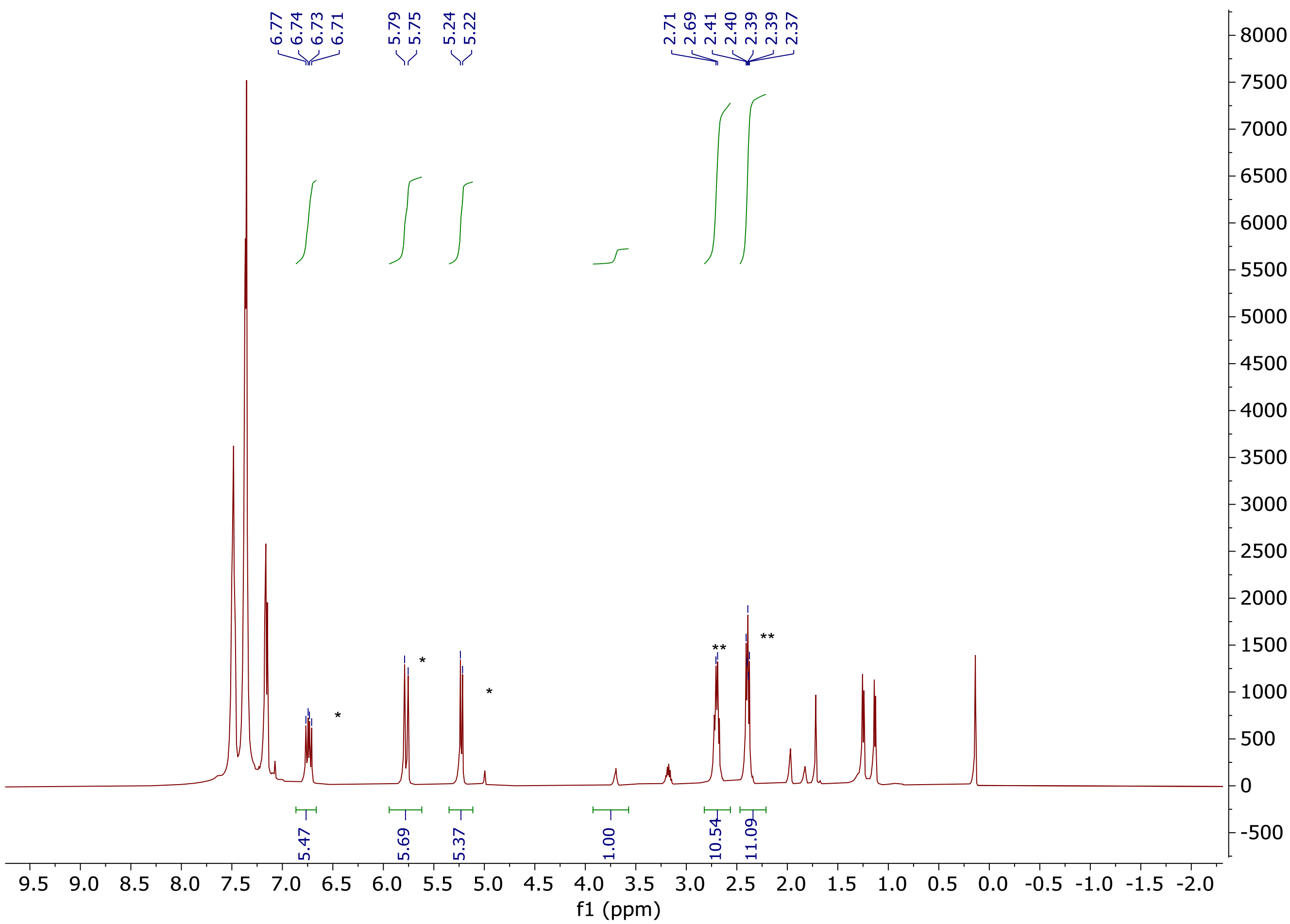

Figure S89: Hydrophosphination of 0.5 mmol 4-vinyl styrene with equimolar of Ph_2PH in DMSO-d_6 using 5 mol% **1** – 0 h – ^{1}H NMR

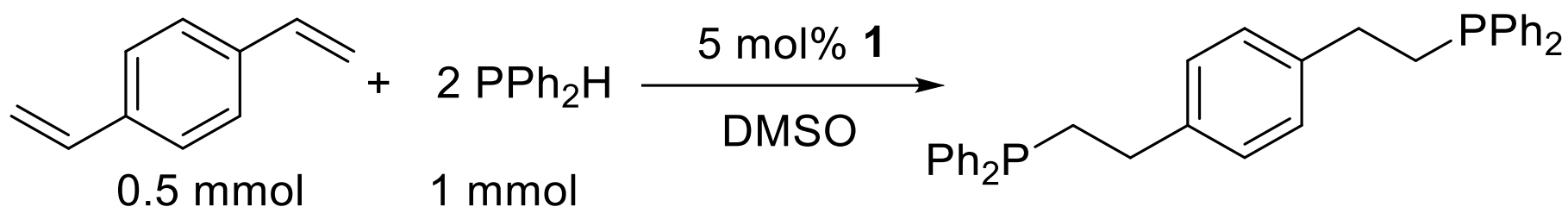

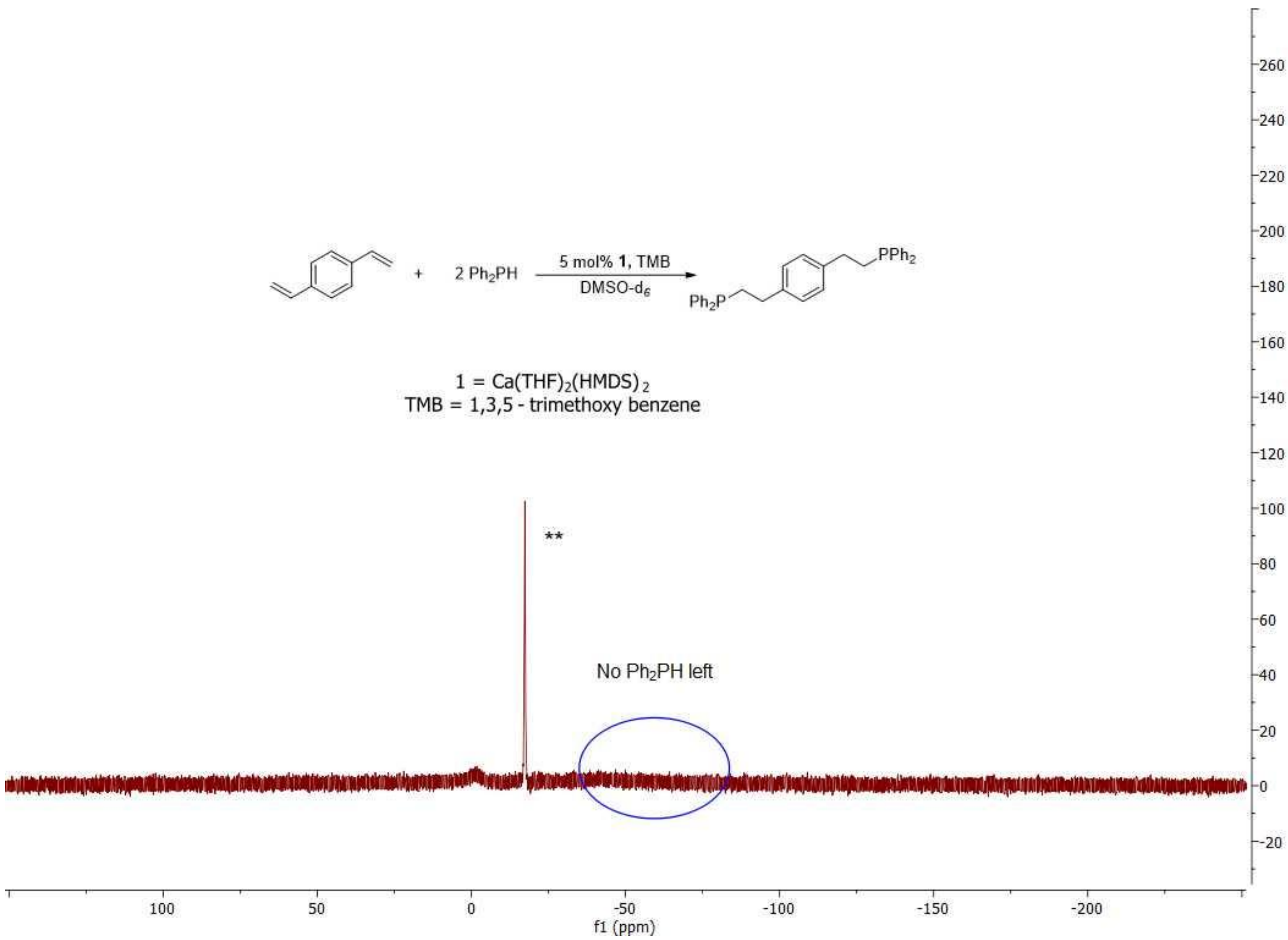

Figure S90: Hydrophosphination of 0.5 mmol 4-vinyl styrene with equimolar of Ph_2PH in DMSO-d_6 using 5 mol% **1** – 0 h – $^{31}P\{^1H\}$ NMR

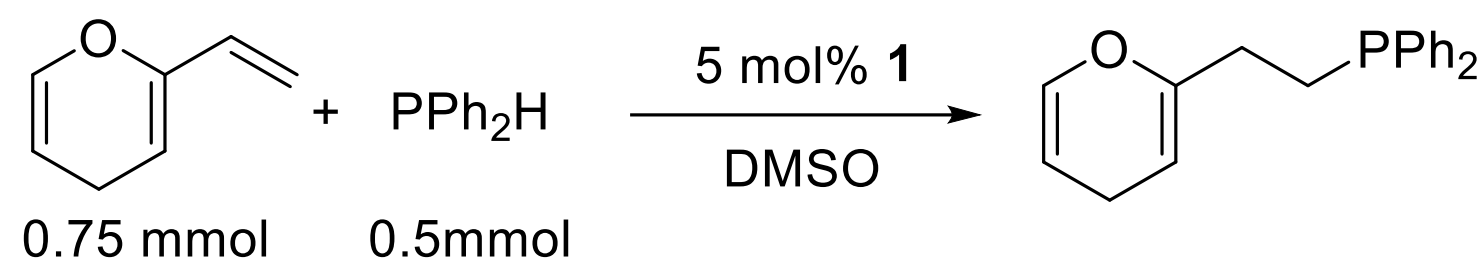

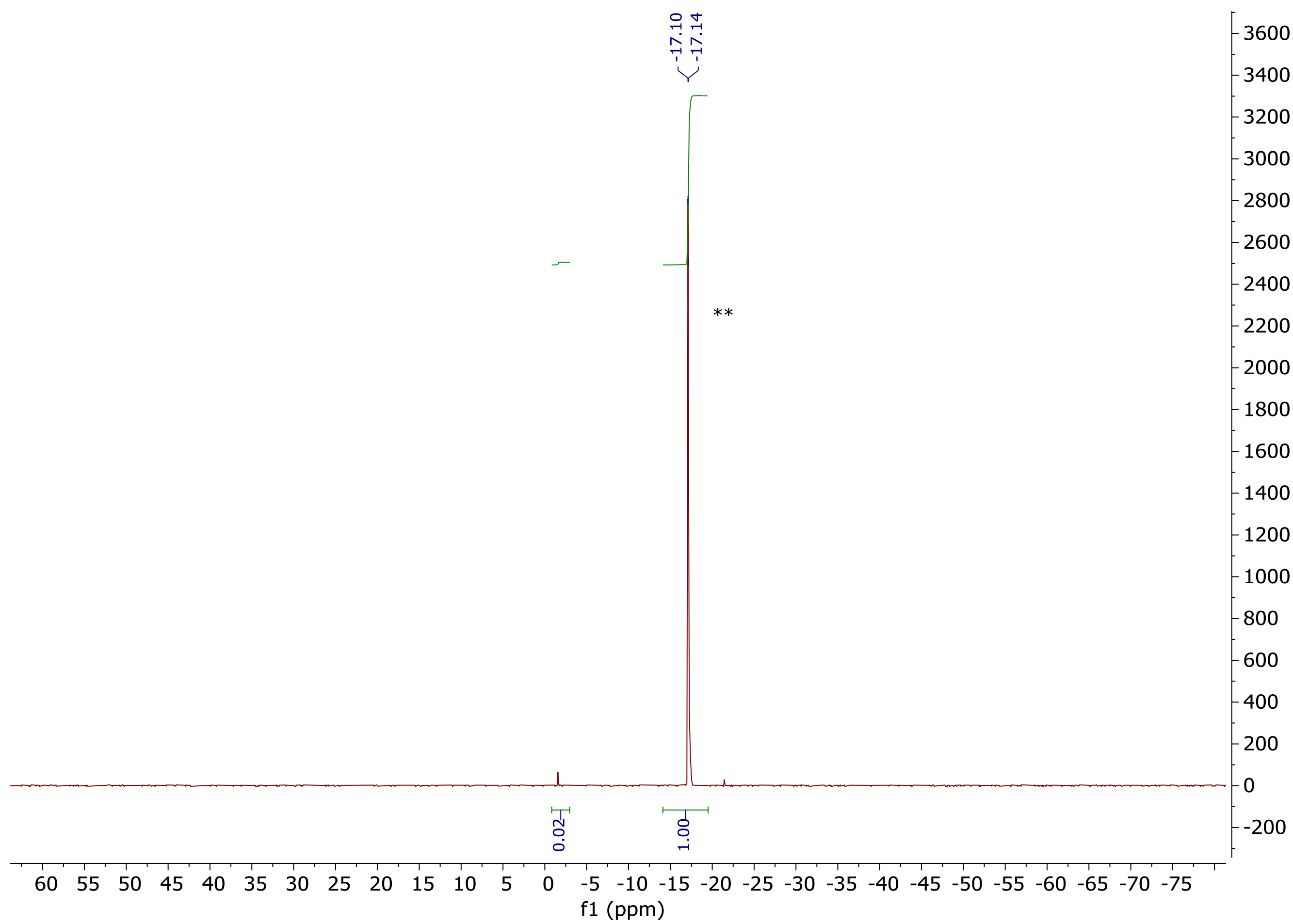

Figure S91: Hydrophosphination of 0.5 mmol 2-vinyl-4H-pyran with equimolar of Ph_2PH in DMSO-d_6 using 5 mol% **1** – 0 h – $^{31}P\{^1H\}$ NMR

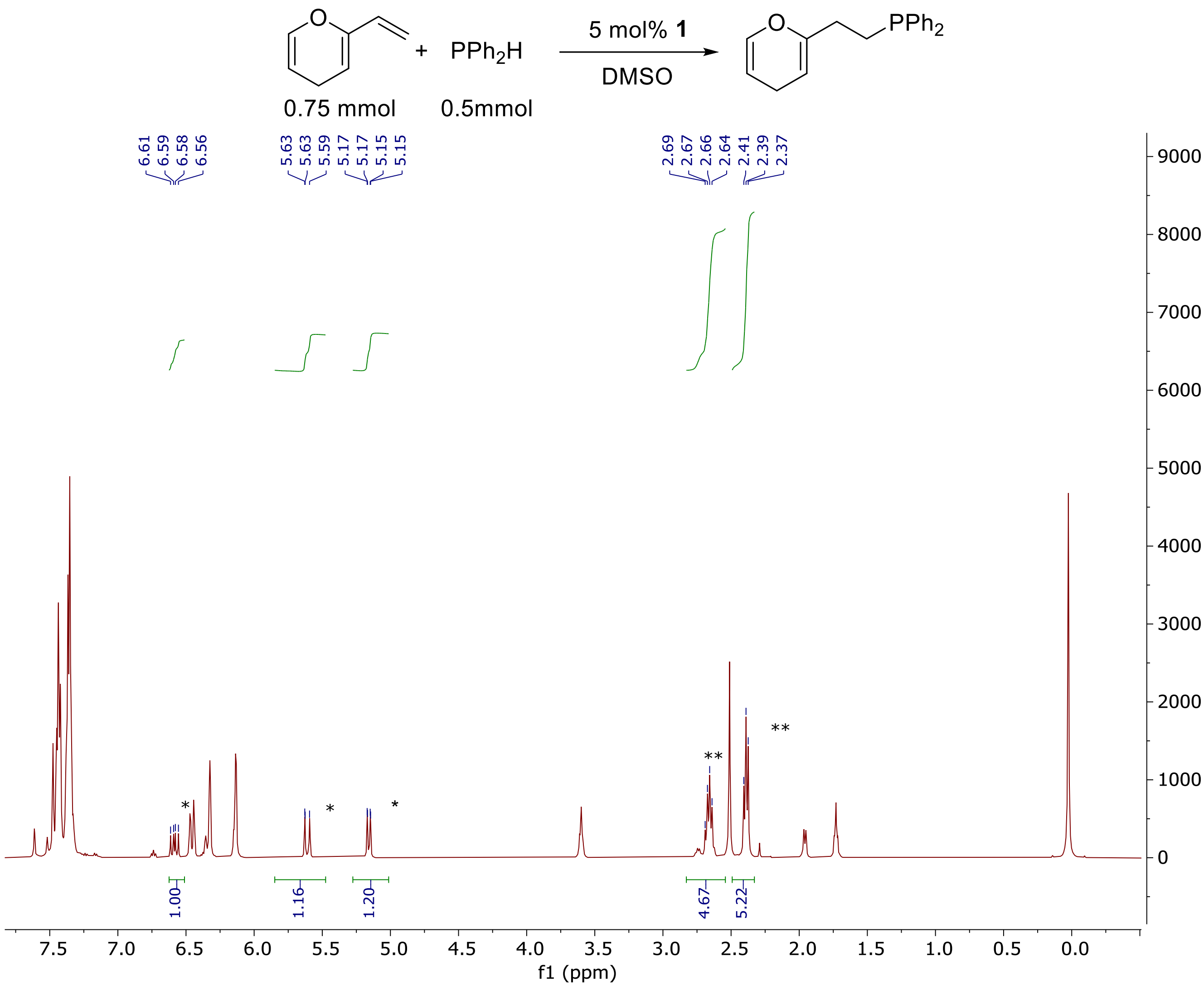

Figure S92: Hydrophosphination of 0.5 mmol 2-vinyl-4H-pyran with equimolar of Ph_2PH in DMSO-d_6 using 5 mol% **1** – 0 h – ^{1}H NMR

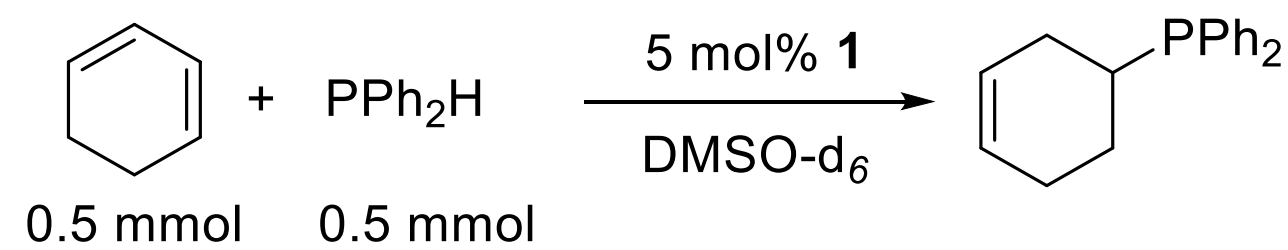

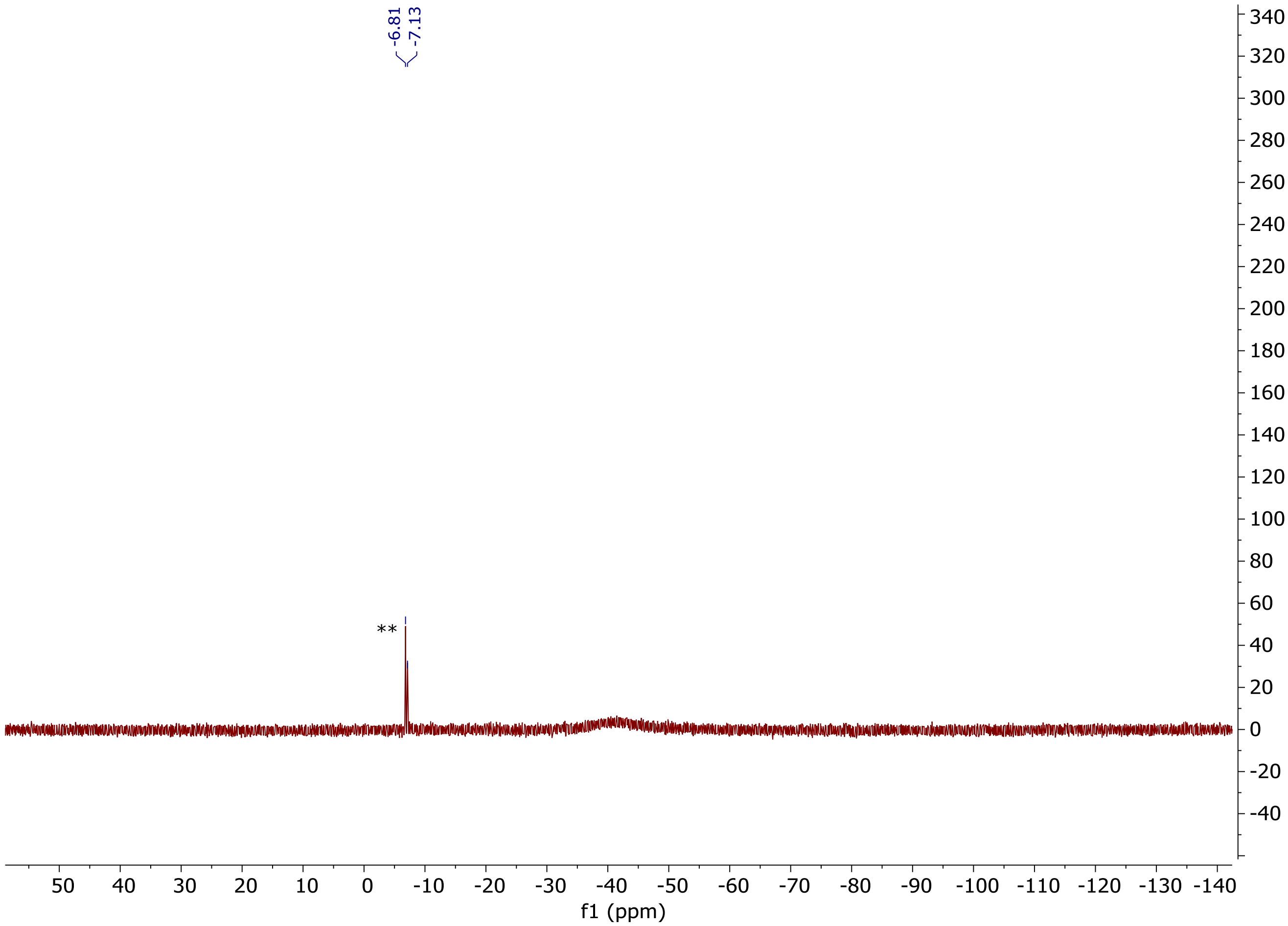

Figure S93: Hydrophosphination of 0.5 mmol 1,3 - cyclohexadiene with equimolar of Ph_2PH in DMSO-d_6 using 5 mol% **1** – 0 h – $^{31}P\{^1H\}$ NMR

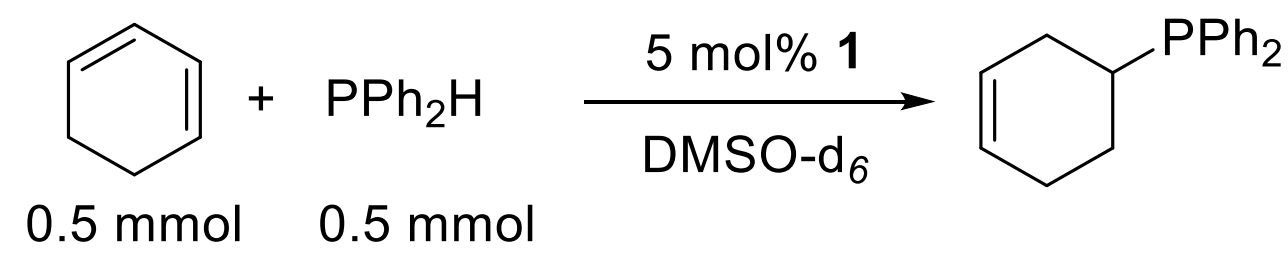

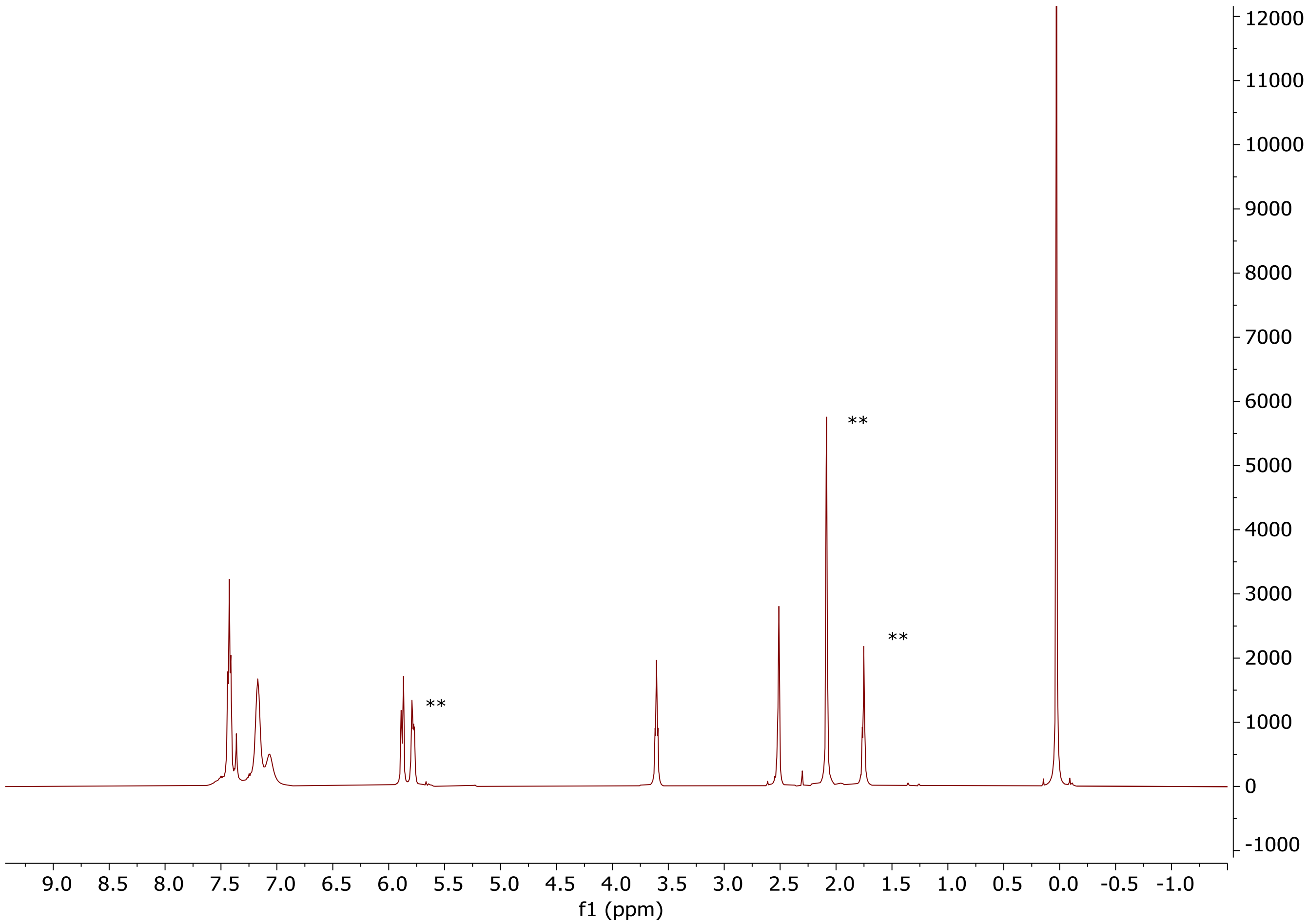

Figure S94: Hydrophosphination of 0.5 mmol 1,3 - cyclohexadiene with equimolar of Ph_2PH in DMSO-d_6 using 5 mol% **1** under– 0 h – ^{1}H NMR

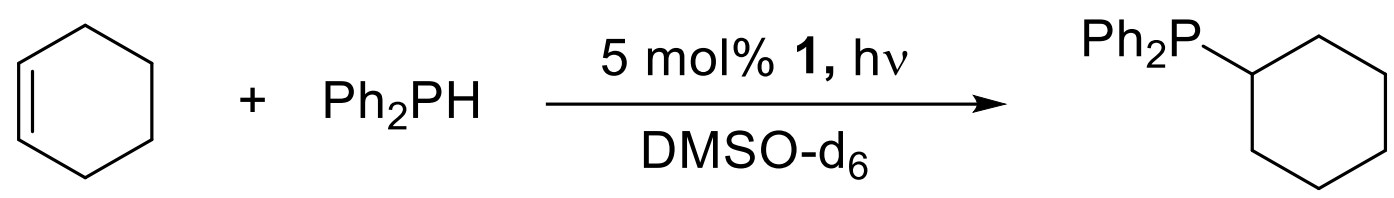

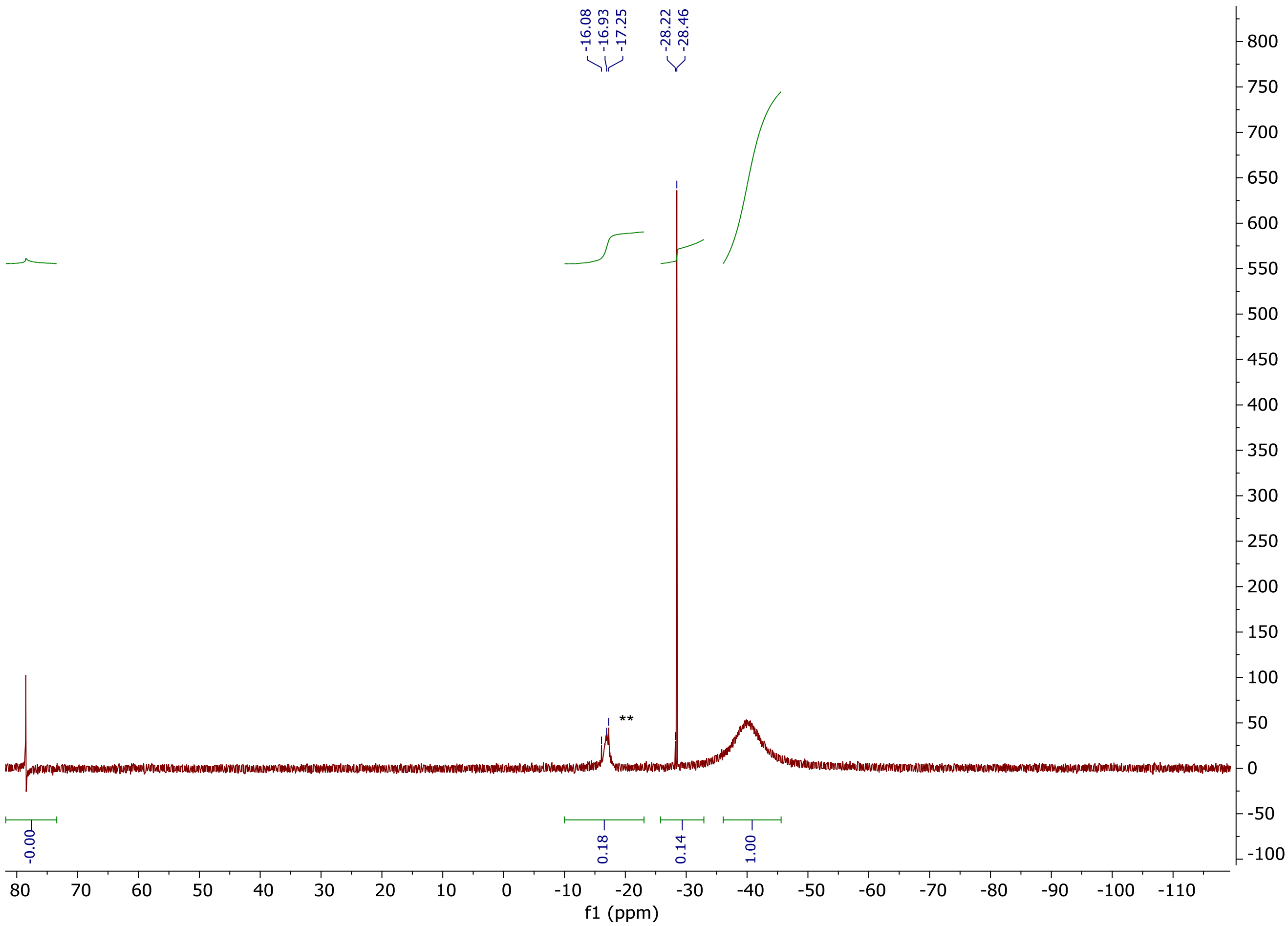

Figure S95: Hydrophosphination of 0.5 mmol cyclohexene with equimolar of Ph_2PH in DMSO-d_6 using 5 mol% **1** under UV 360 nm irradiation – 48 h – $^{31}P\{^1H\}$ NMR

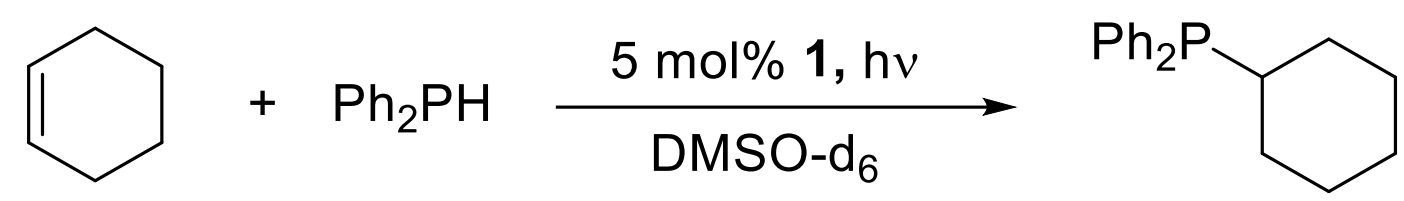

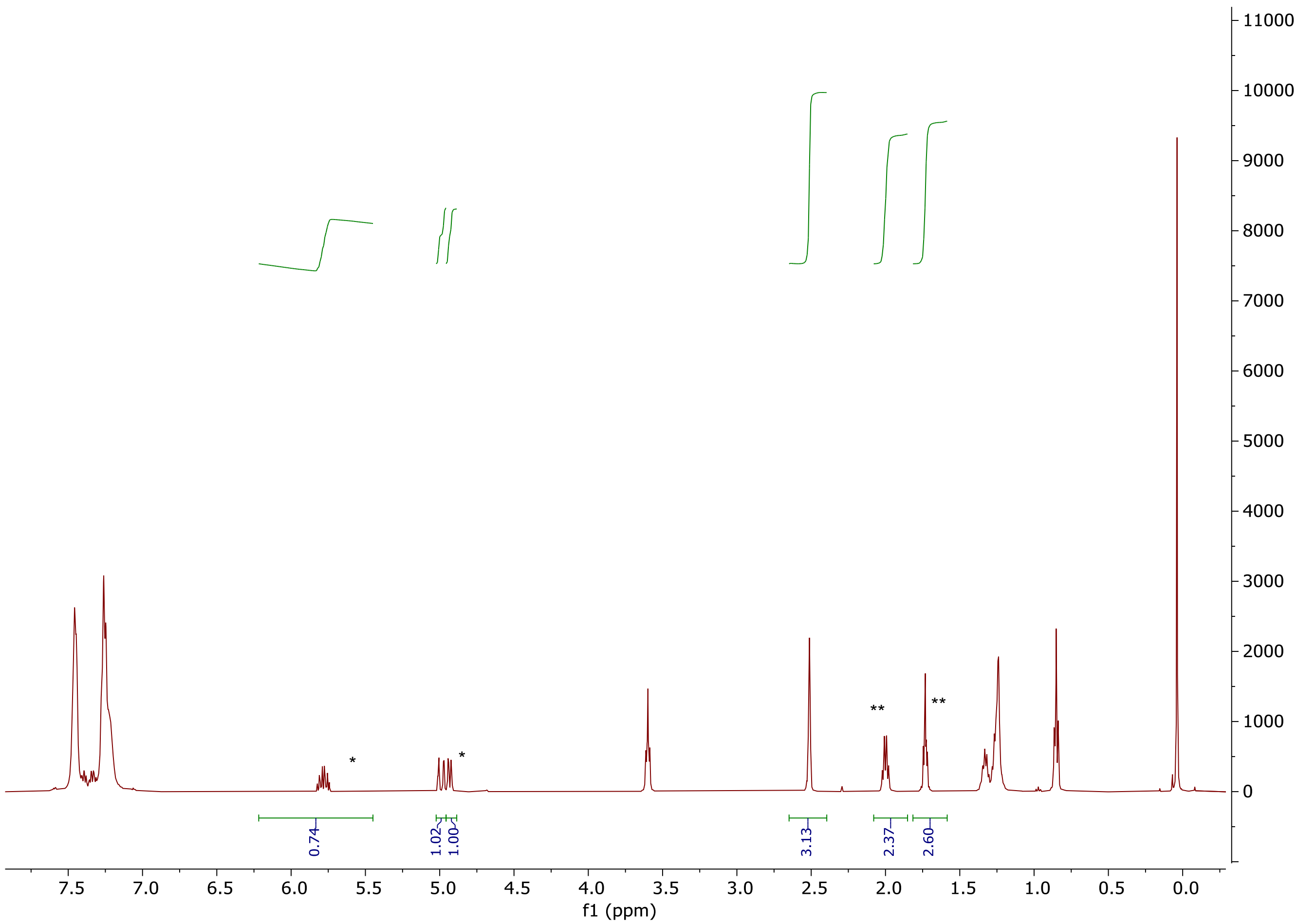

Figure S96: Hydrophosphination of 0.5 mmol cyclohexene with equimolar of Ph_2PH in DMSO-d_6 using 5 mol% **1** under UV 360 nm irradiation – 48 h – ^1H NMR

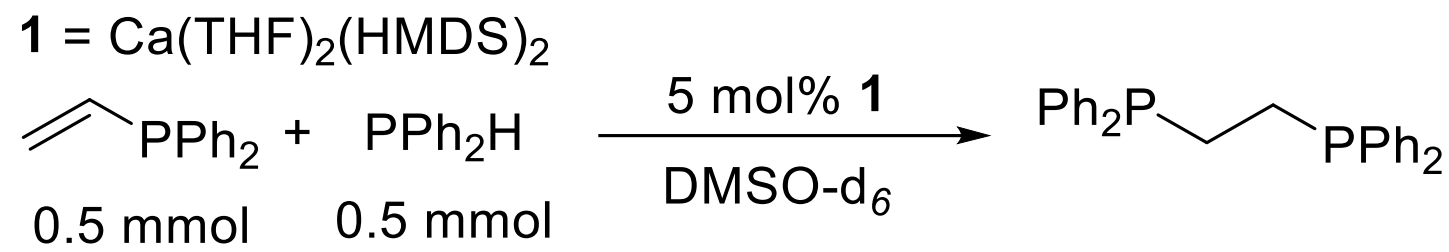

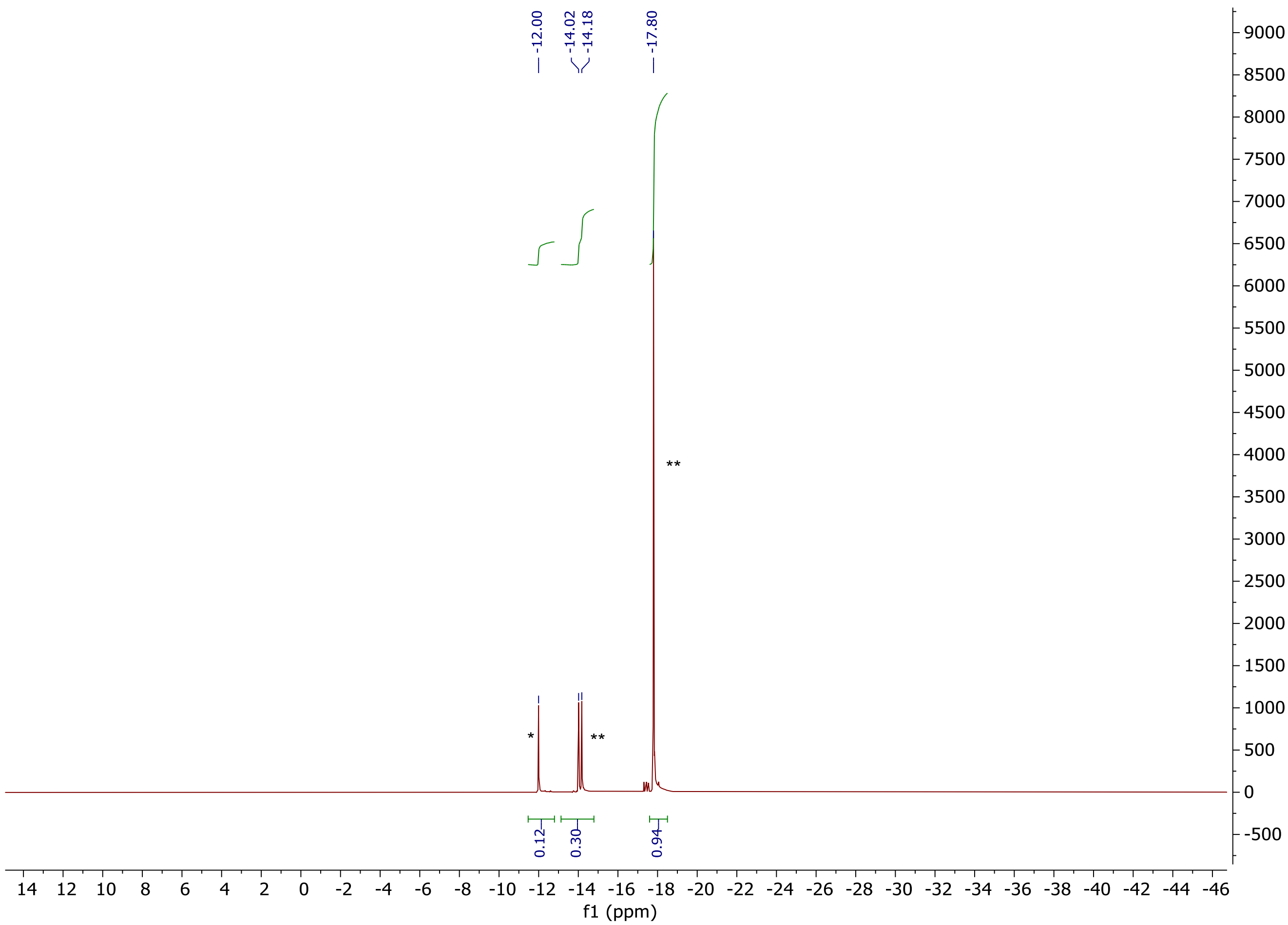

Figure S97: Hydrophosphination of 0.5 mmol vinyl diphenyl phosphine with equimolar of Ph_2PH in DMSO-d_6 using 5 mol% **1** – 0 h – $^{31}P\{^1H\}$ NMR

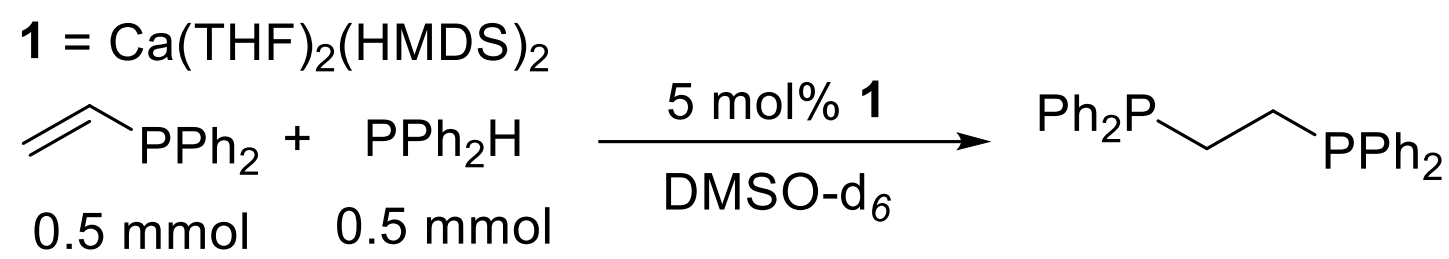

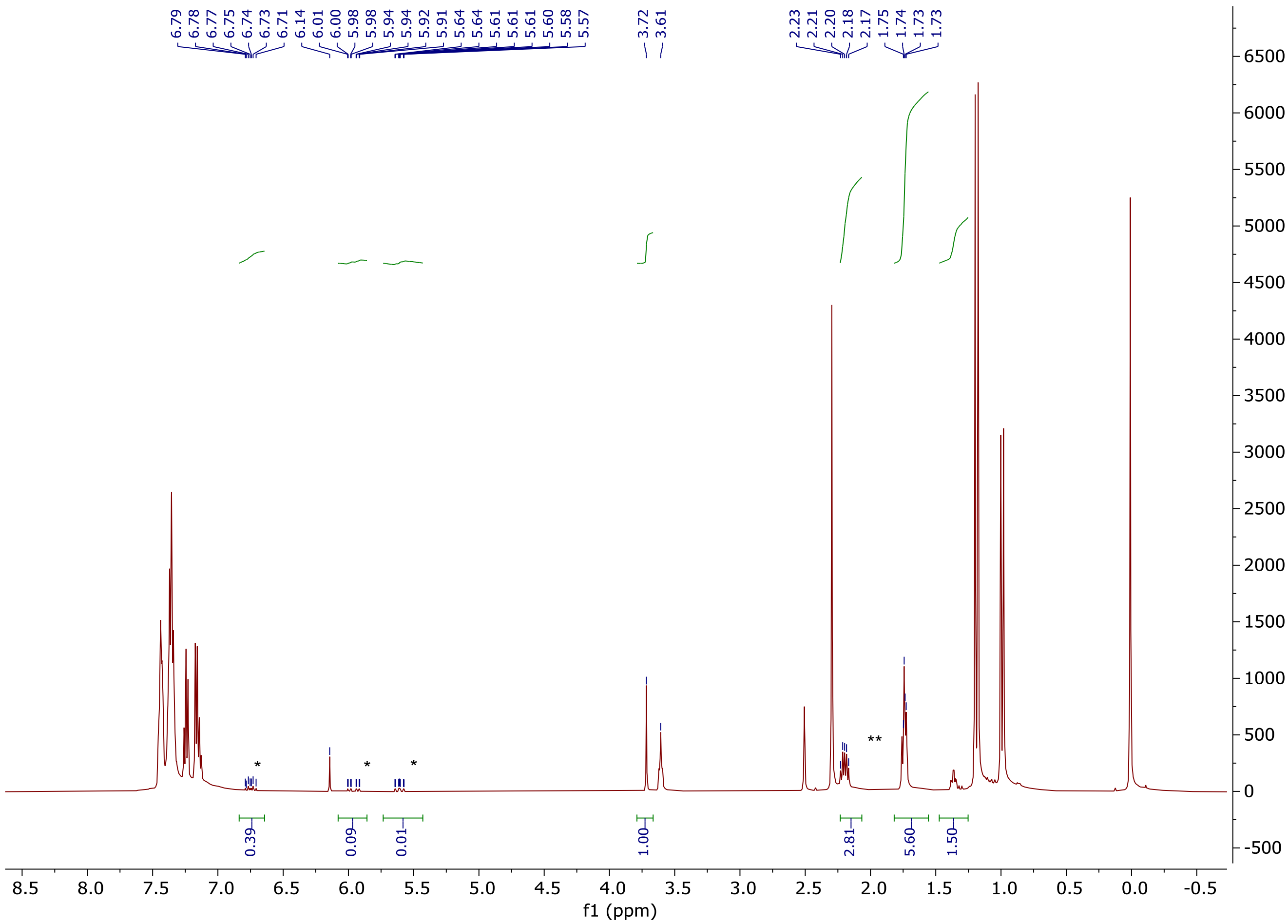

Figure S98: Hydrophosphination of 0.5 mmol vinyl diphenyl phosphine with equimolar of Ph_2PH in DMSO-d_6 using 5 mol% **1** – 0 h – ^1H NMR

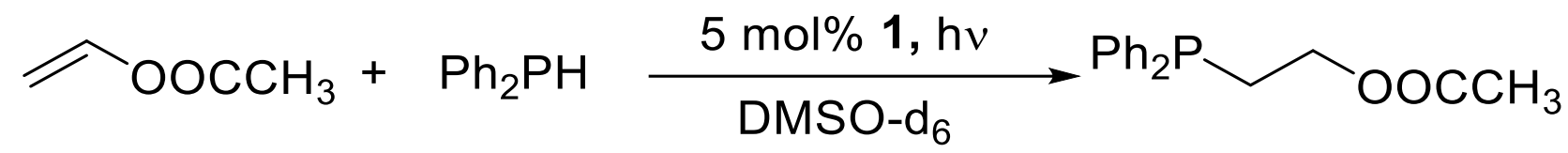

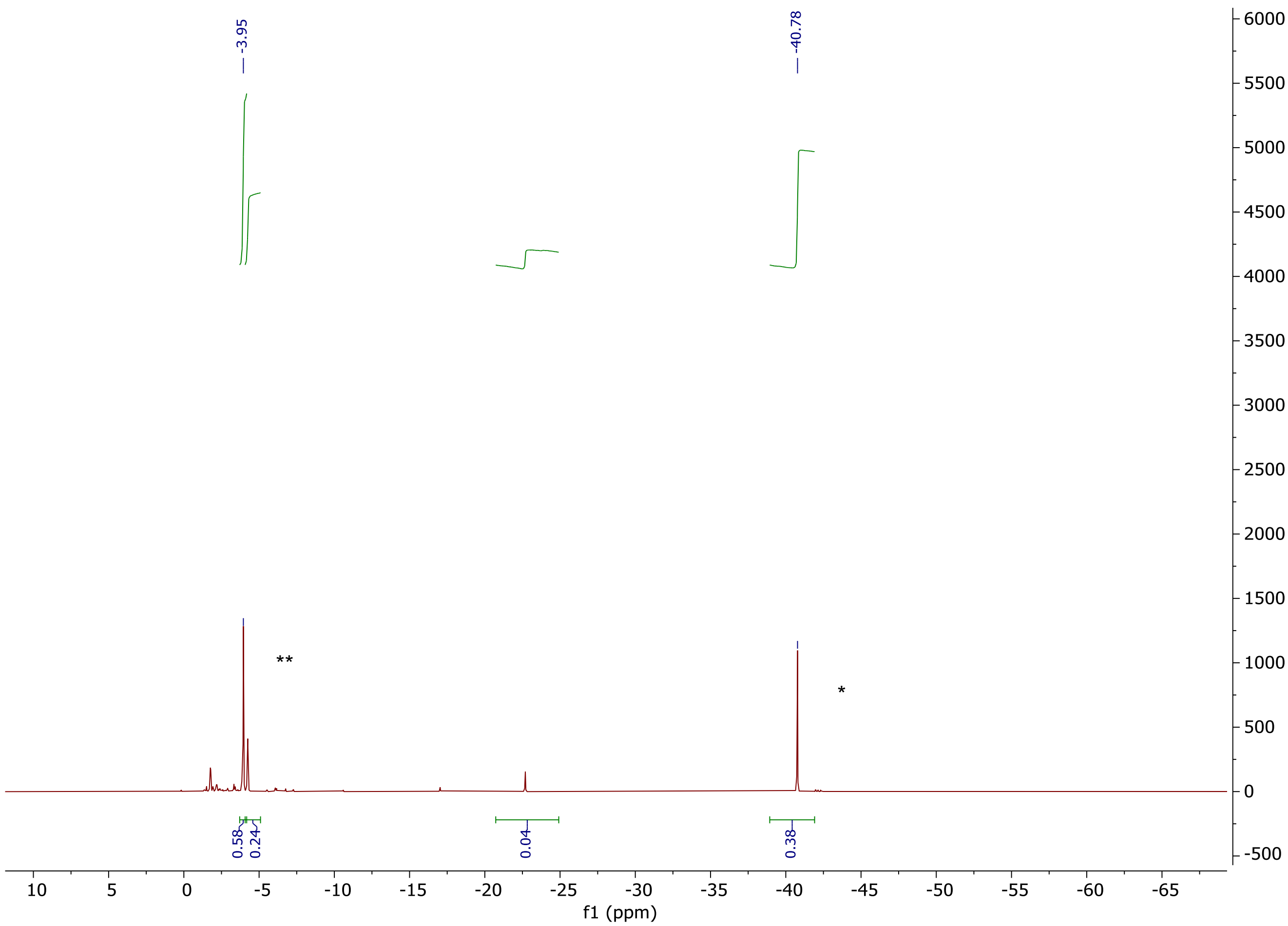

Figure S99: Hydrophosphination of 0.5 mmol vinyl acetate with equimolar of Ph_2PH in DMSO-d_6 using 5 mol% **1** under UV 360 nm irradiation – 48 h – $^{31}P\{^1H\}$ NMR

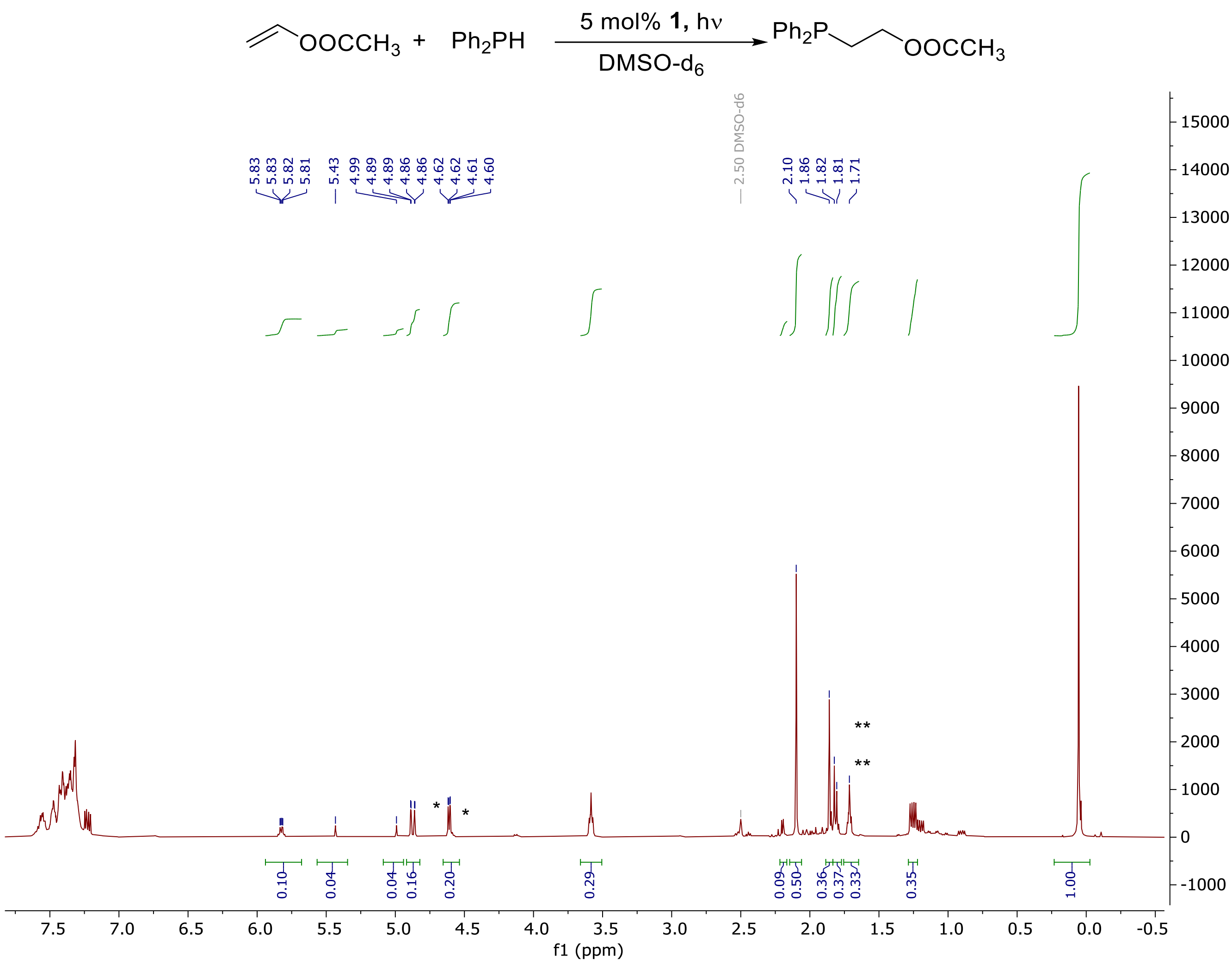

Figure S100: Hydrophosphination of 0.5 mmol vinyl acetate with equimolar of Ph_2PH in DMSO-d_6 using 5 mol% **1** under UV 360 nm irradiation – 48 h – ^1H NMR

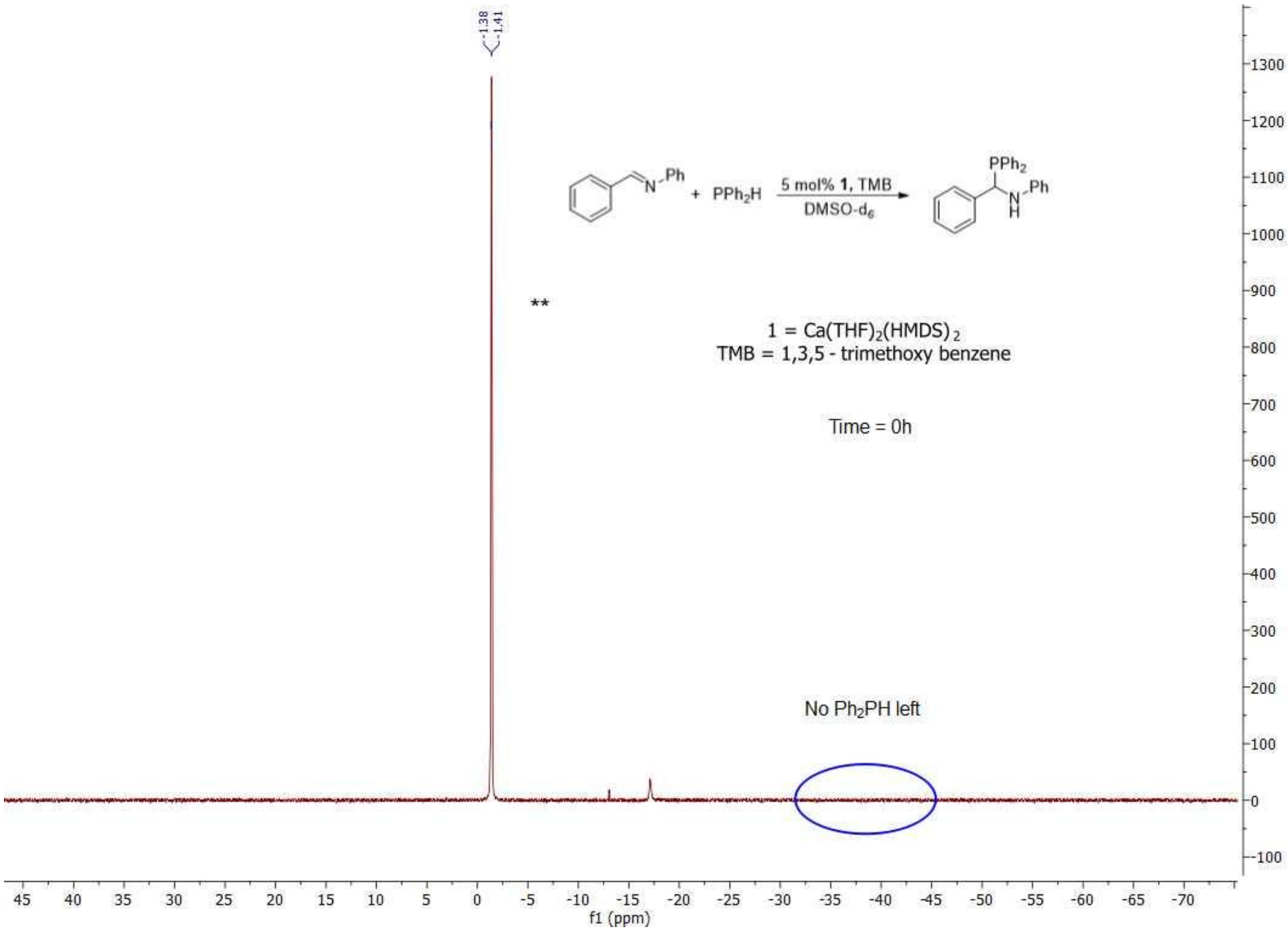

Figure S101: Hydrophosphination of 0.5 mmol N-benzylidine aniline with equimolar of Ph_2PH in DMSO-d_6 using 5 mol% **1** – 0 h – $^{31}P\{^{1}H\}$ NMR

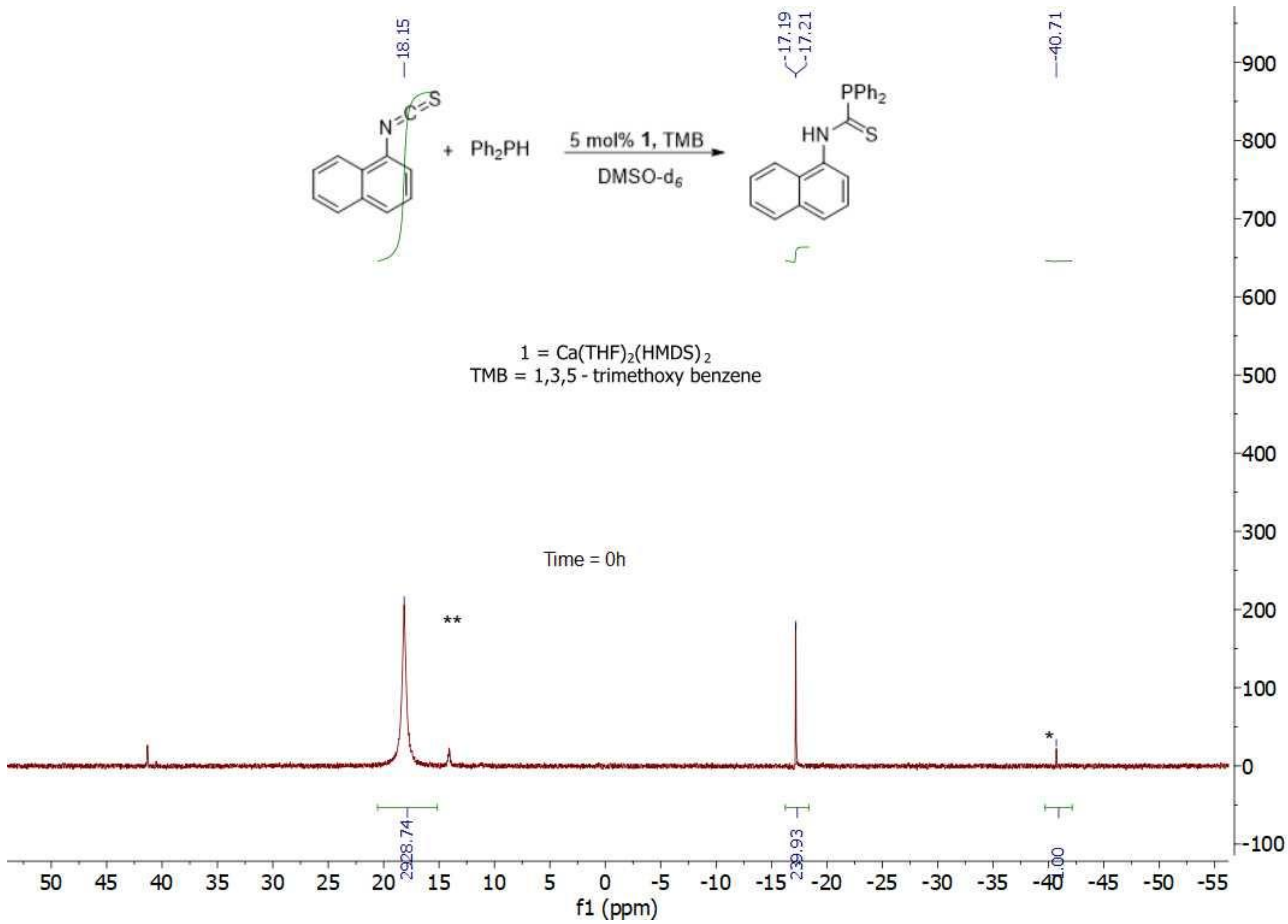

Figure S102: Hydrophosphination of 0.5 mmol 1-naphthylisothiocyanate with equimolar of Ph_2PH in DMSO-d_6 using 5 mol% **1** 0 h – $^{31}P\{^1H\}$ NMR

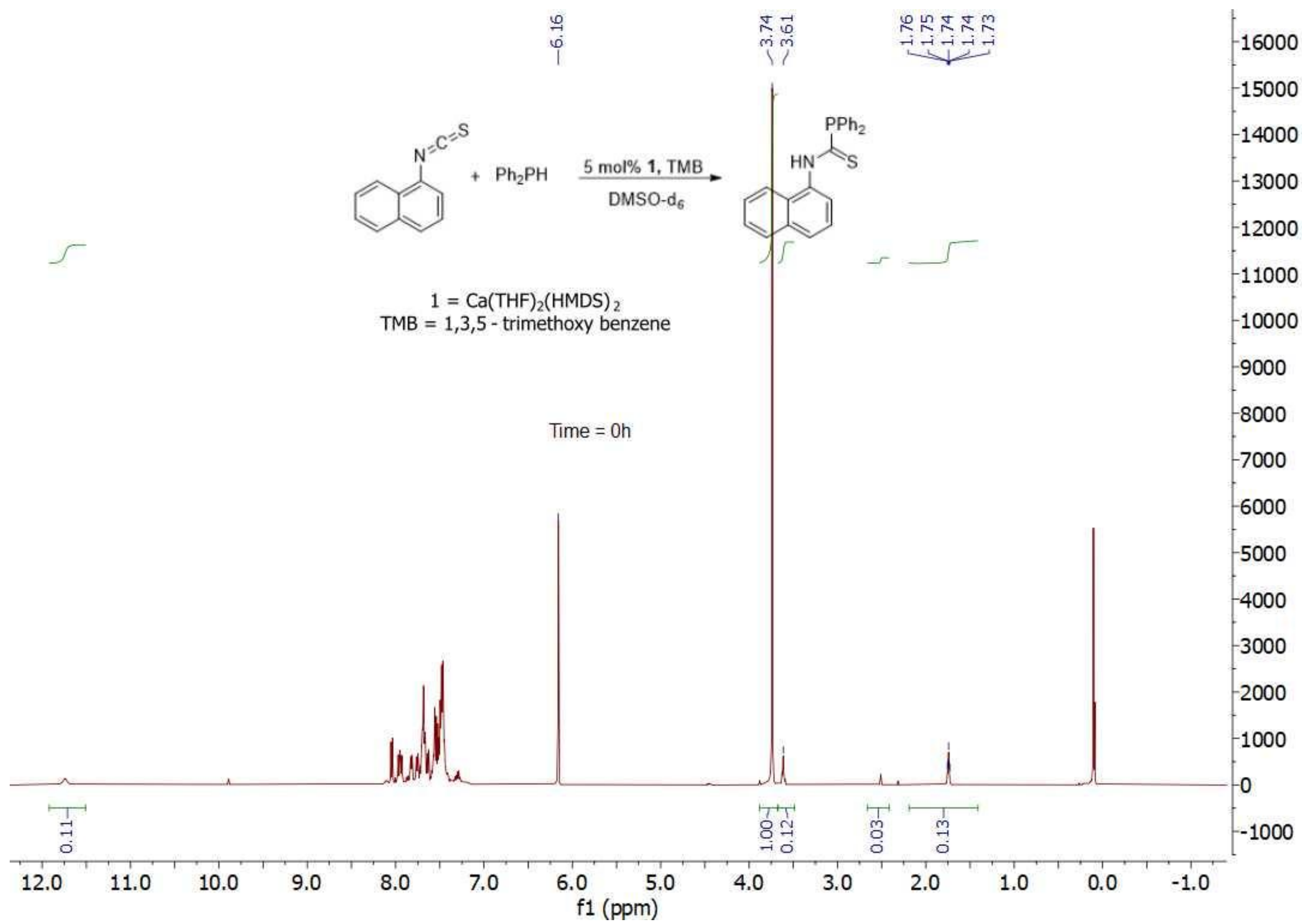

Figure S103: Hydrophosphination of 0.5 mmol 1-naphthylisothiocyanate with equimolar of Ph_2PH in DMSO-d_6 using 5 mol% **1** – 0 h – ^1H NMR

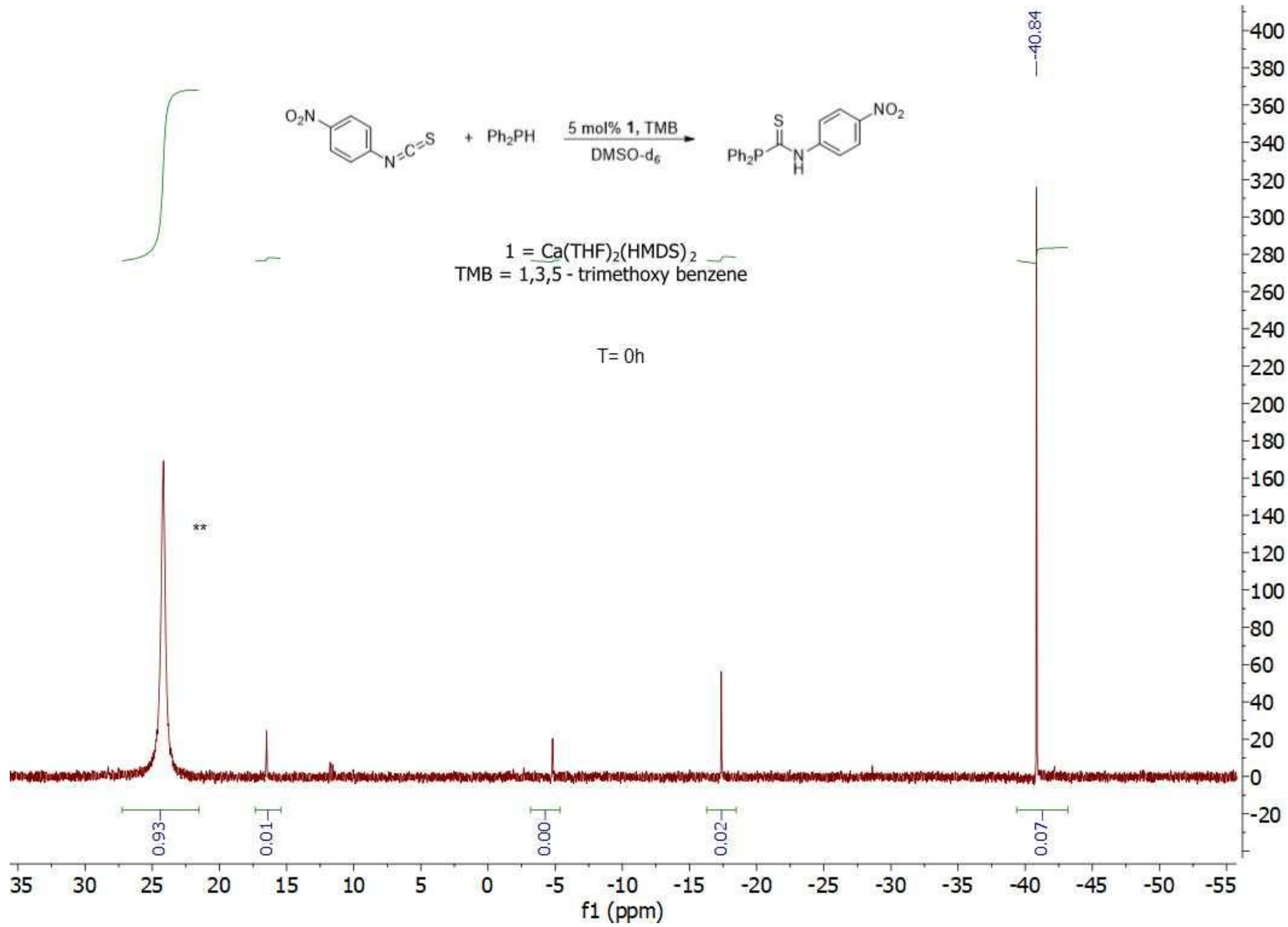

Figure S104: Hydrophosphination of 0.5 mmol 4-nitrophenylisothiocyanate with equimolar of Ph_2PH in DMSO-d_6 using 5 mol% **1** – 0 h – $^{31}P\{^1H\}$ NMR

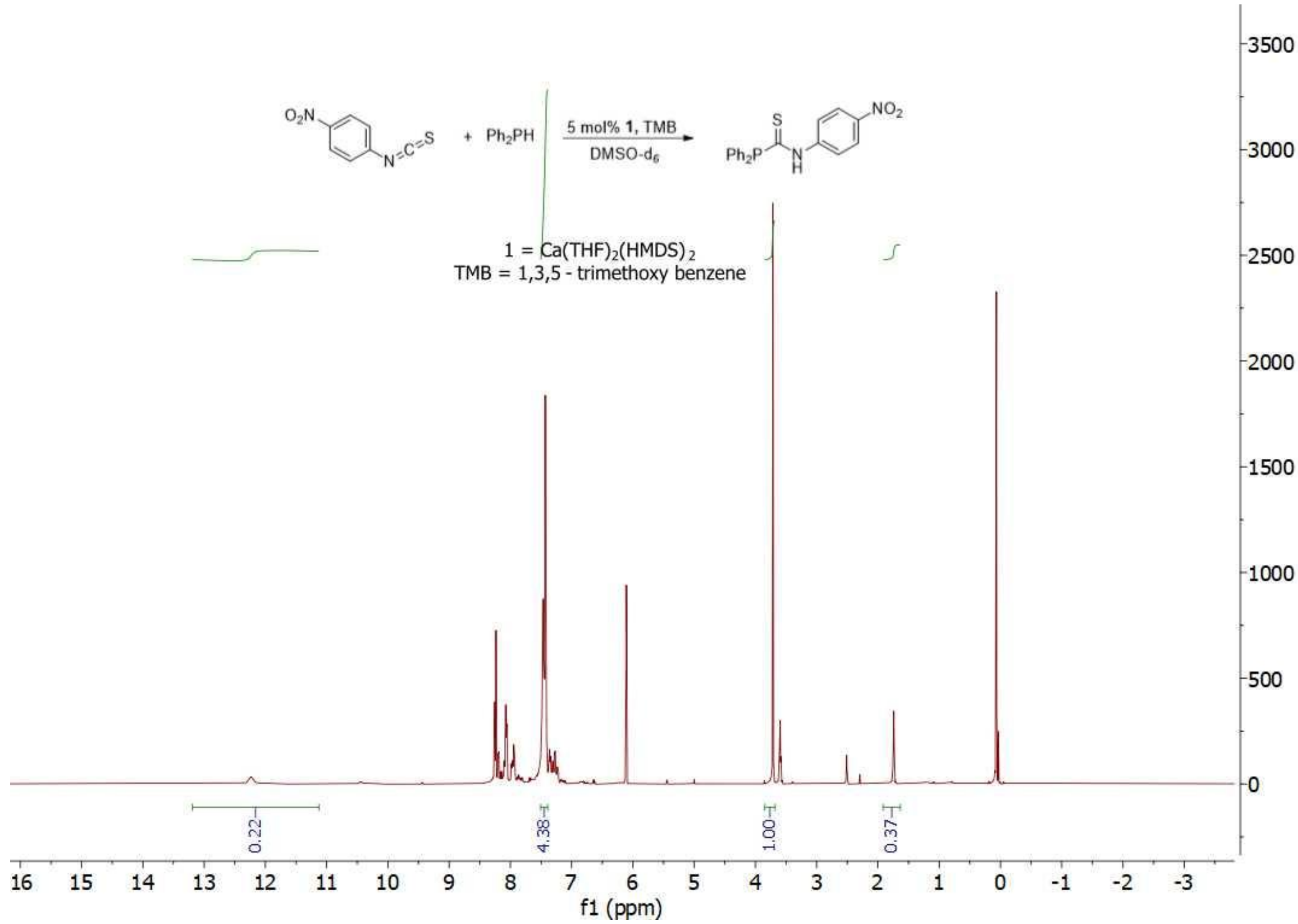

Figure S105: Hydrophosphination of 0.5 mmol 4-nitrophenylisothiocyanate with equimolar of Ph_2PH in DMSO-d_6 using 5 mol% **1** under UV360 nm irradiation – 0 h – ^{1}H NMR

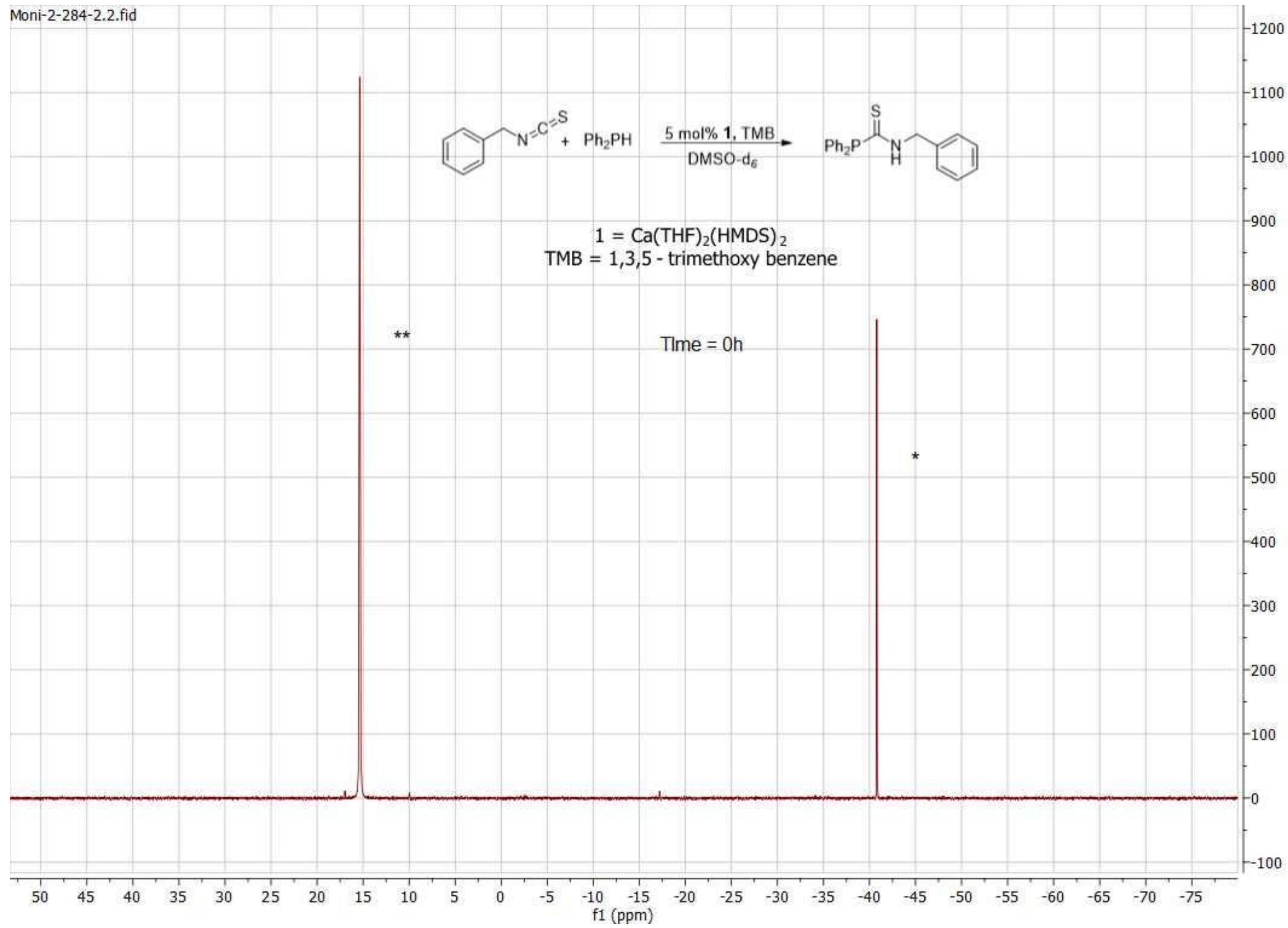

Figure S106: Hydrophosphination of 0.5 mmol benzylisothiocyanate with equimolar of Ph_2PH in DMSO-d_6 using 5 mol% **1** under UV 360 nm irradiation – 0 h – $^{31}P\{^1H\}$ NMR

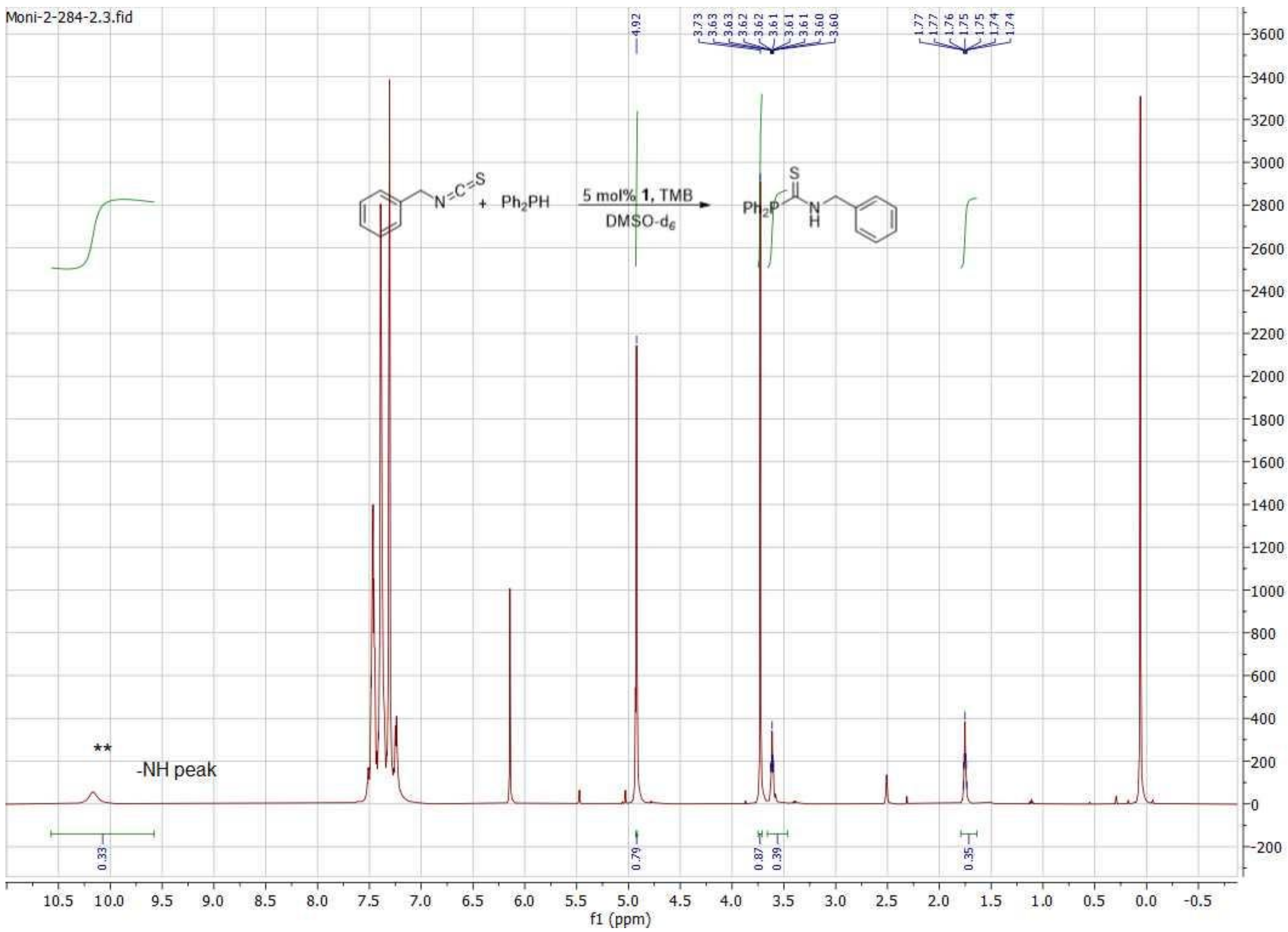

Figure S107: Hydrophosphination of 0.5 mmol benzylisothiocyanate with equimolar of Ph_2PH in DMSO-d_6 using 5 mol% **1** under UV 360 nm irradiation – 0 h – ^{1}H NMR

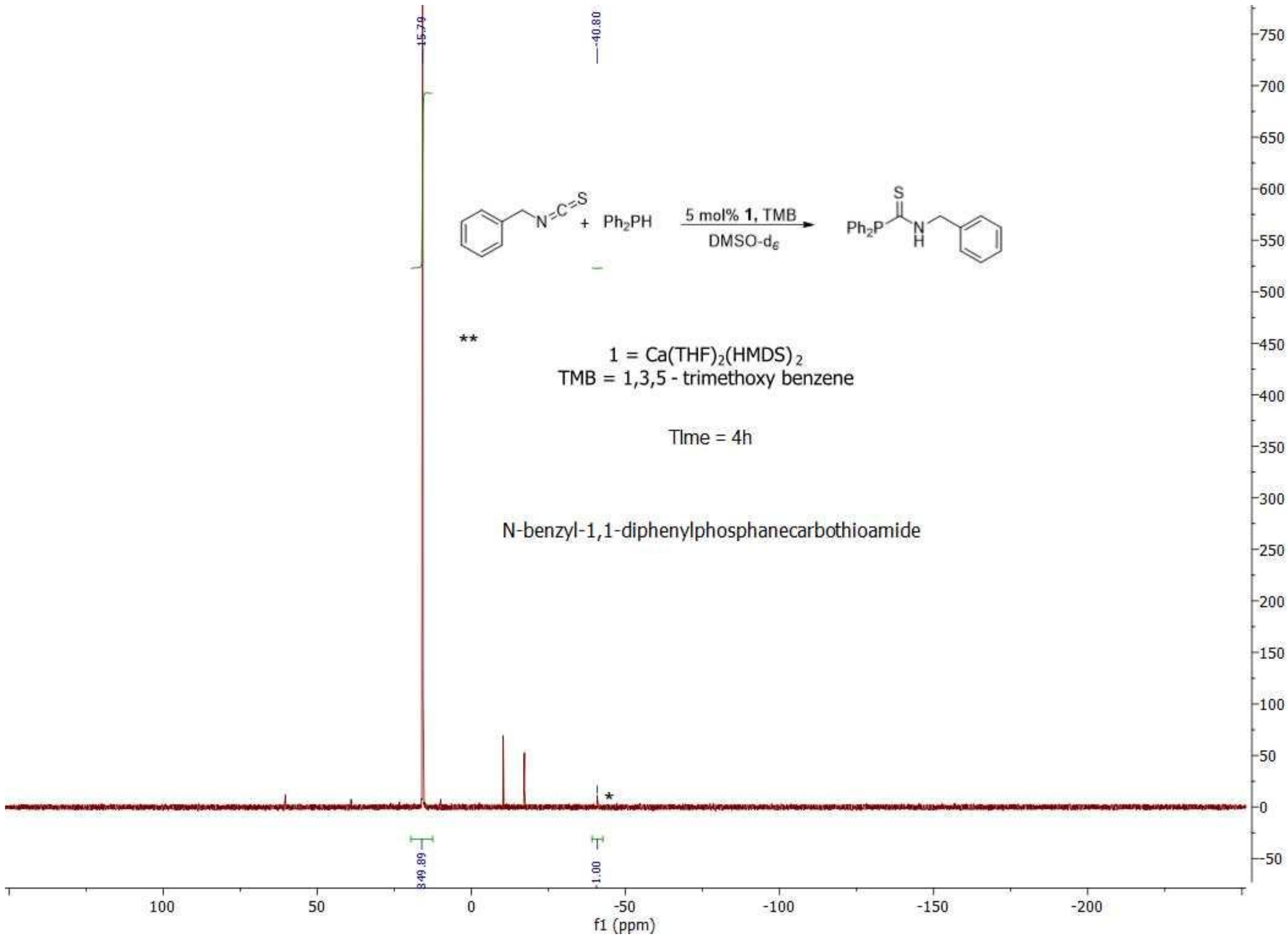

Figure S108: Hydrophosphination of 0.5 mmol benzylisothiocyanate with equimolar of Ph_2PH in DMSO-d_6 using 5 mol% **1** under UV 360 nm irradiation – 4 h – $^{31}P\{^1H\}$ NMR

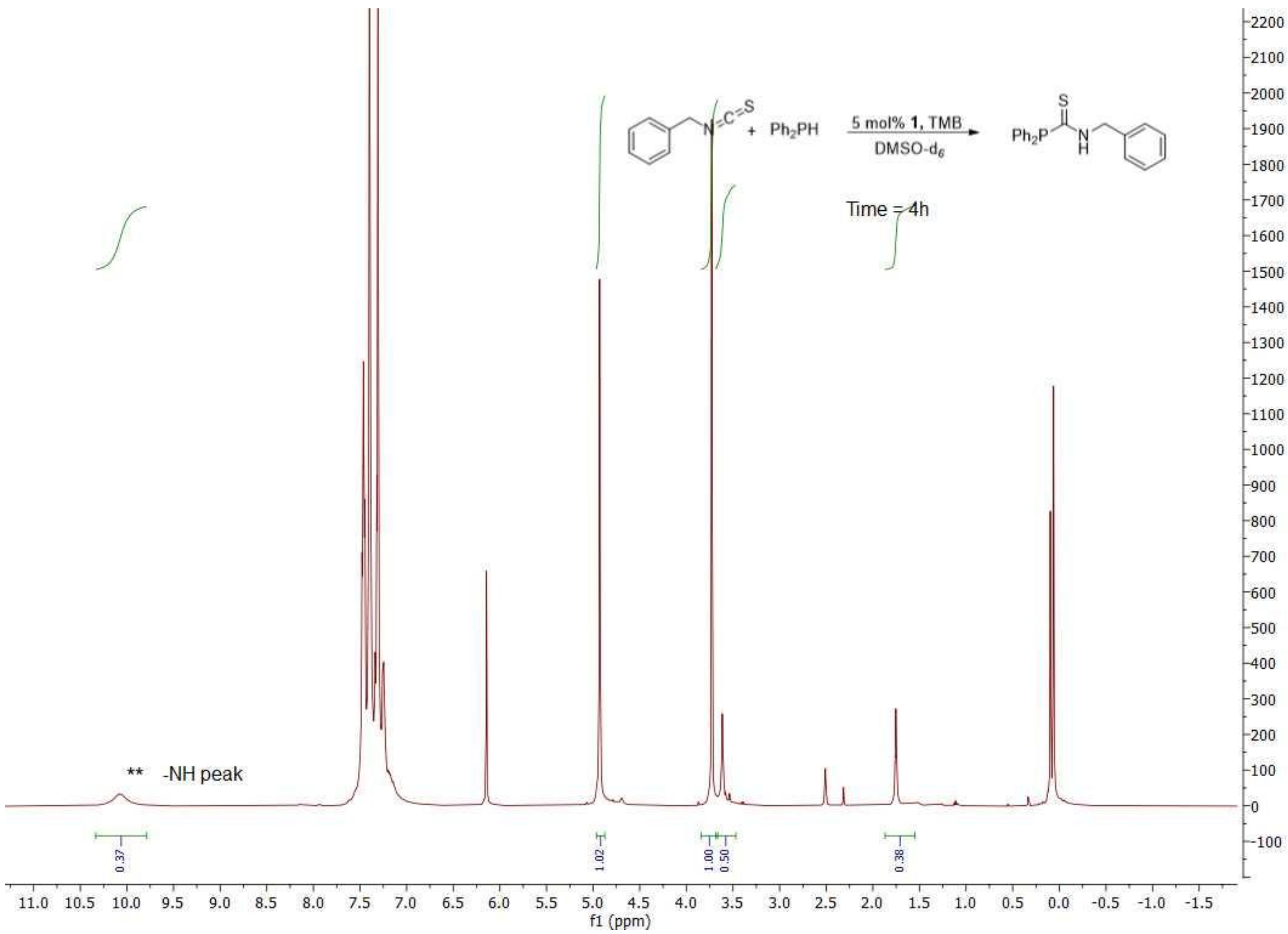

Figure S109: Hydrophosphination of 0.5 mmol benzylisothiocyanate with equimolar of Ph_2PH in DMSO-d_6 using 5 mol% **1** under UV 360 nm irradiation – 4 h – ^1H NMR

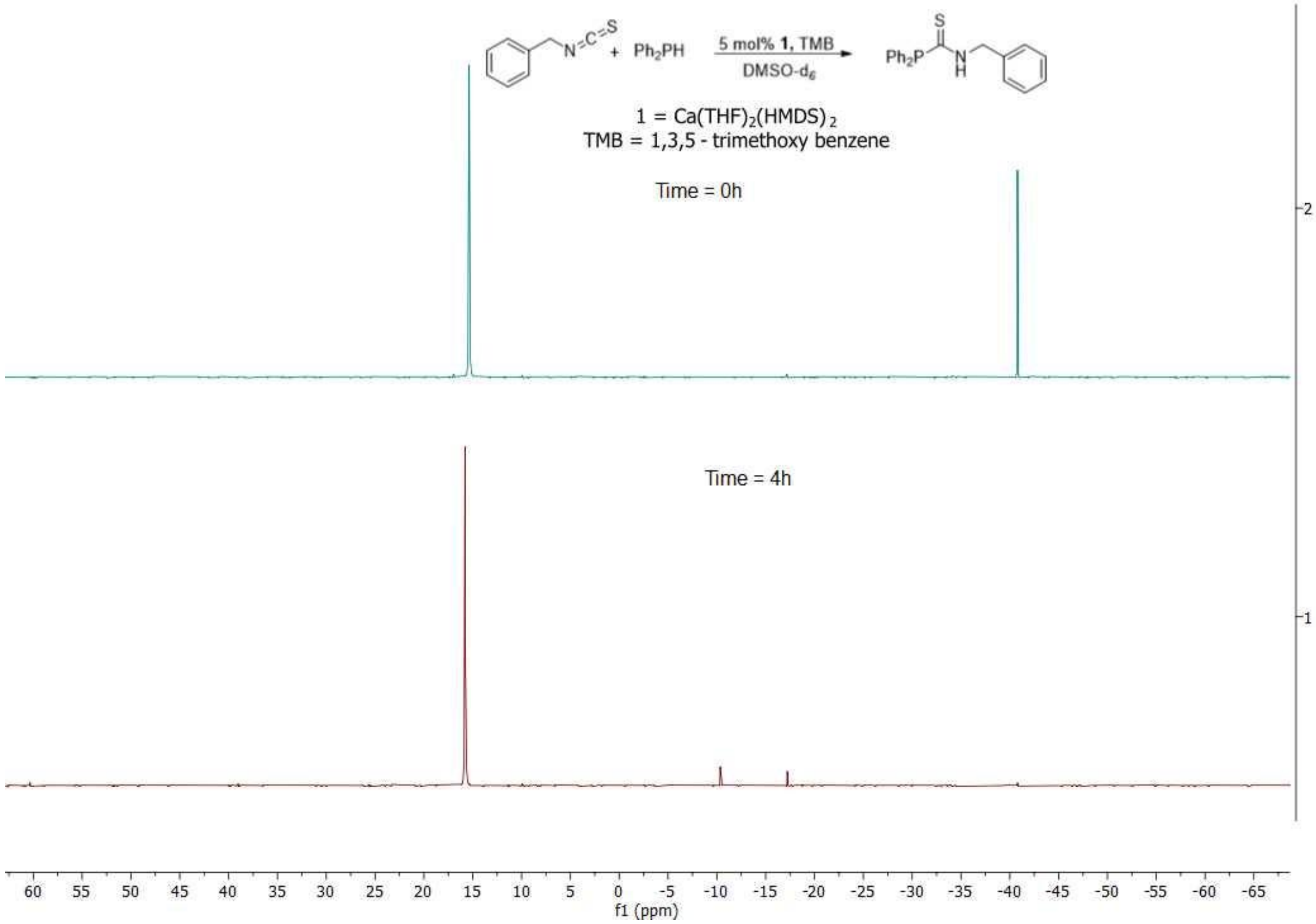

Figure S110: Hydrophosphination of 0.5 mmol benzylisothiocyanate with equimolar of Ph_2PH in DMSO-d_6 using 5 mol% **1** under UV 360 nm irradiation – stacked – $^{31}P\{^1H\}$ NMR

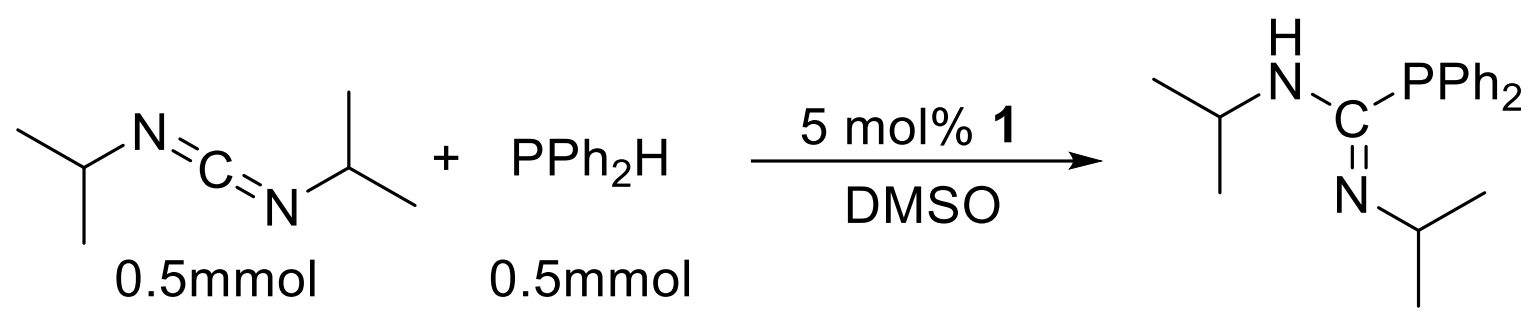

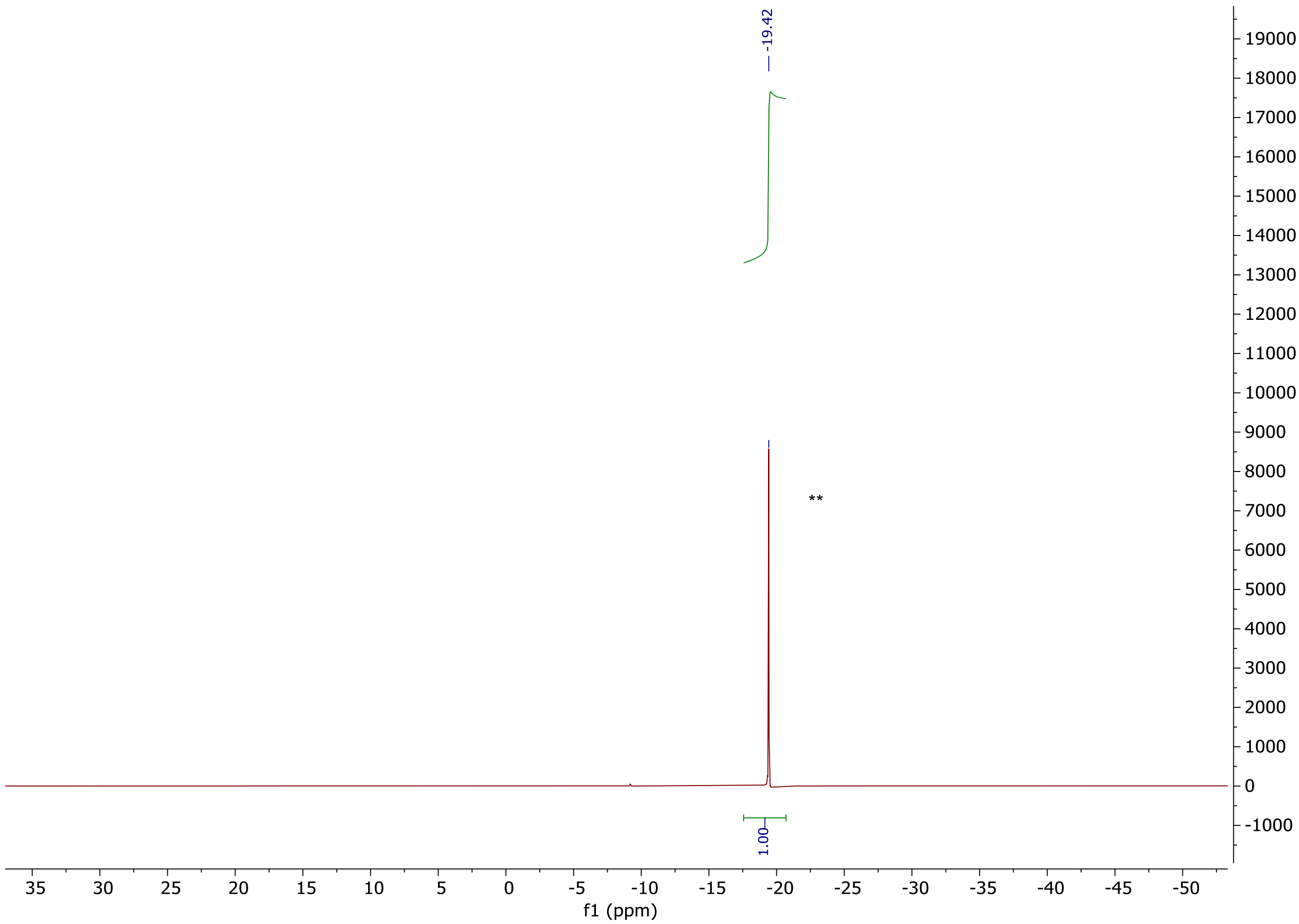

Figure S111: Hydrophosphination of 0.5 mmol N,N' - diisopropylcarbodiimide with equimolar of Ph_2PH in DMSO-d_6 using 5 mol% **1**– 0 h – $^{31}P\{^1H\}$ NMR

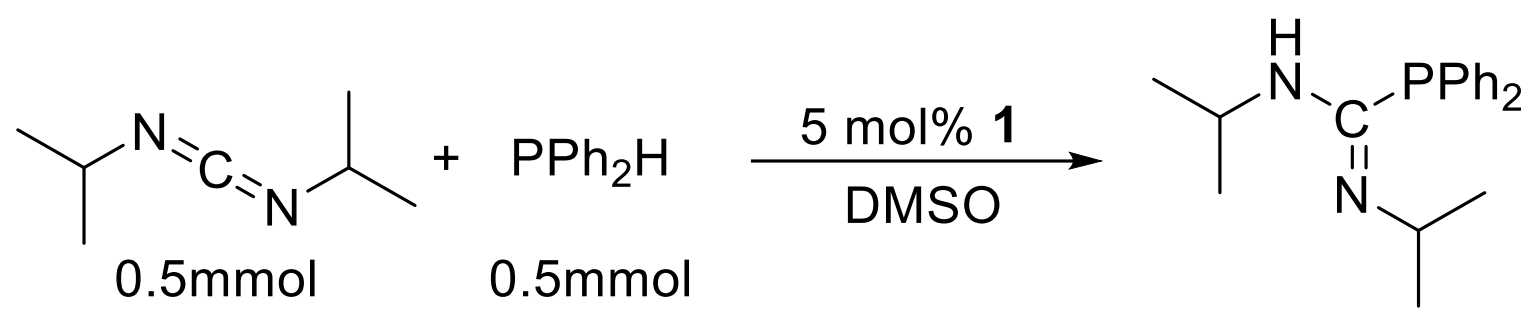

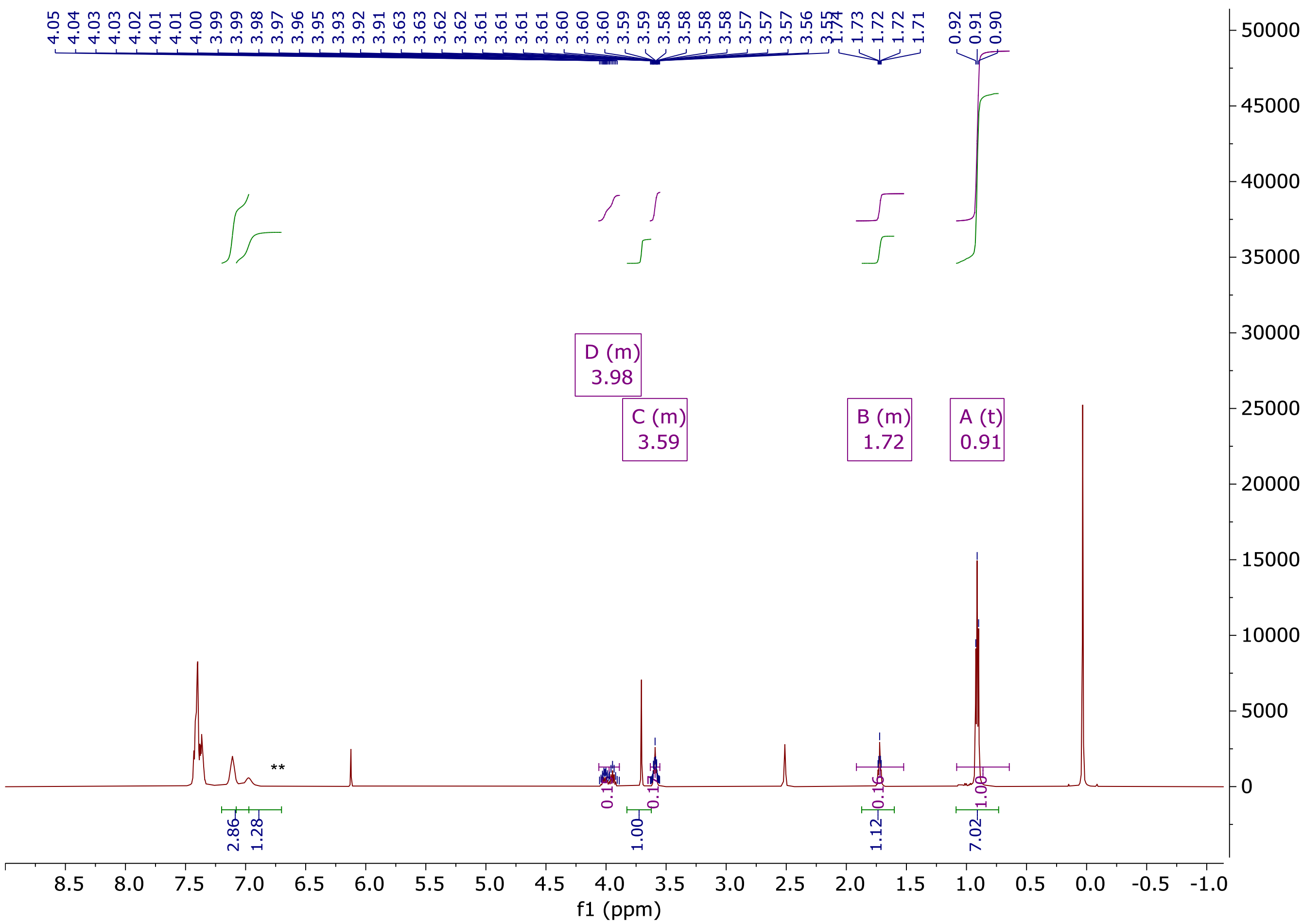

Figure S112: Hydrophosphination of 0.5 mmol N,N' - diisopropylcarbodiimide with equimolar of Ph_2PH in DMSO-d_6 using 5 mol% **1** – 0 h – ^1H NMR

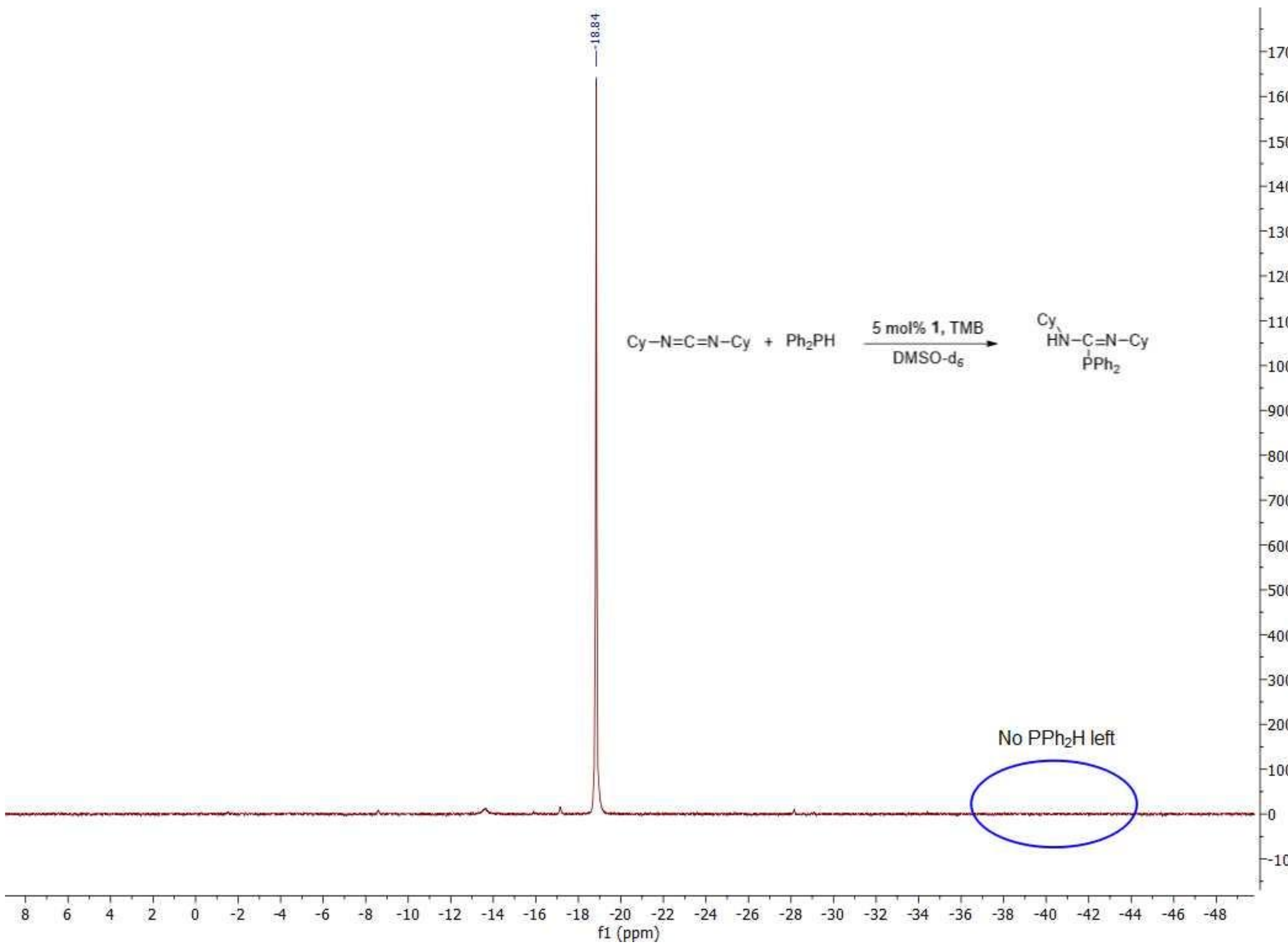

Figure S113: Hydrophosphination of 0.5 mmol with equimolar of Ph_2PH in N,N' -dicyclohexylcarbodiimide DMSO-d_6 using 5 mol% **1** – 0 h – $^{31}P\{^1H\}$ NMR.

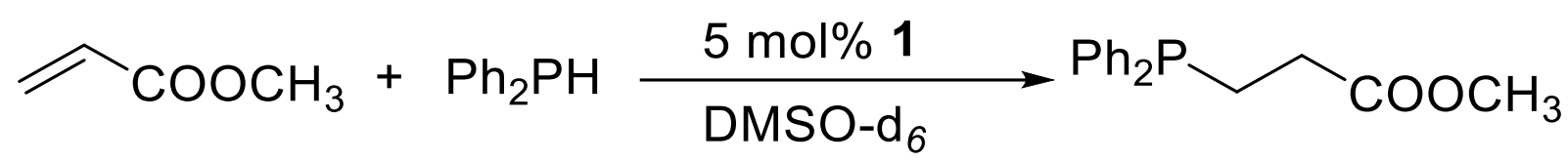

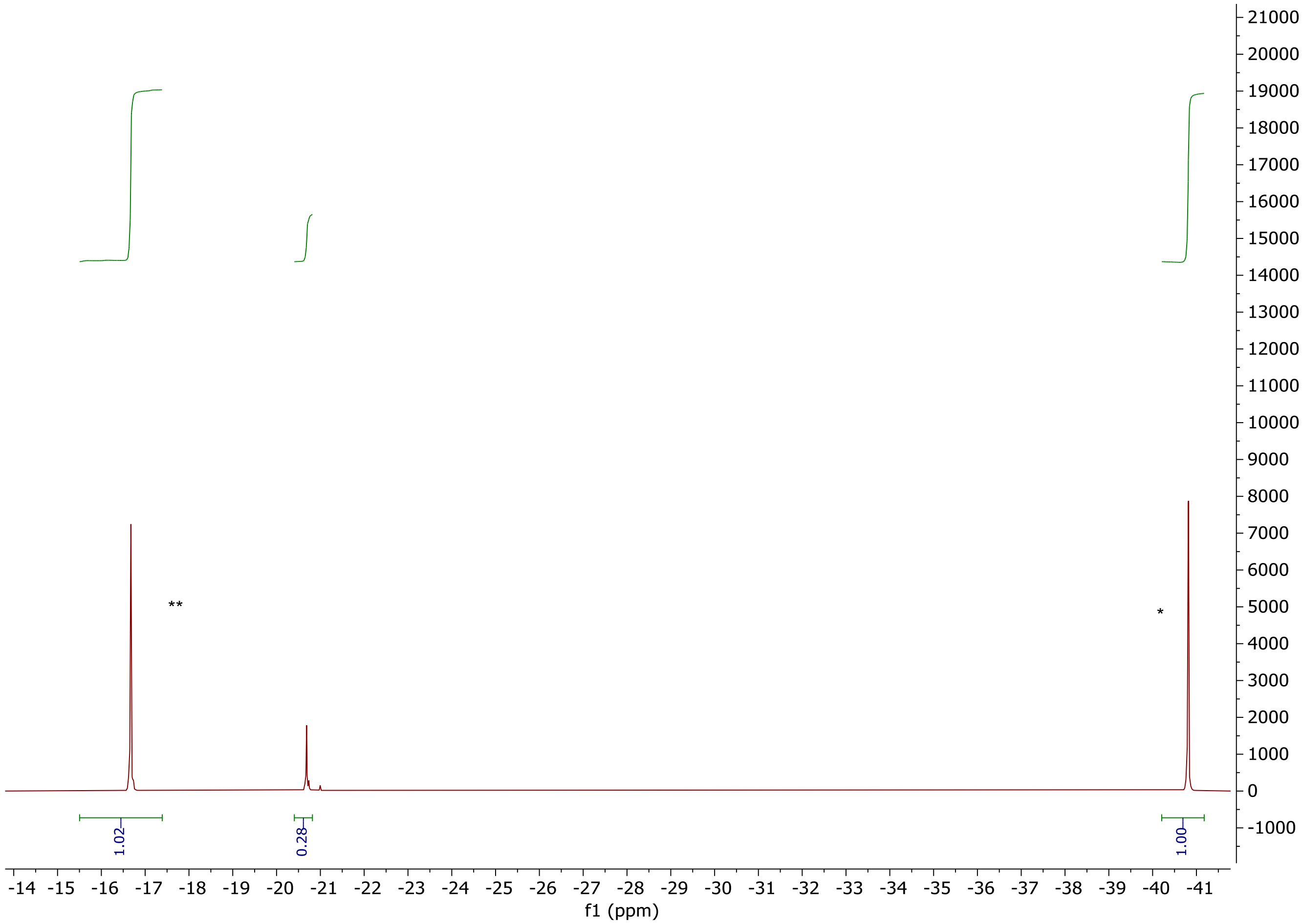

Figure S114: Hydrophosphination of 0.5 mmol methyl acrylate with equimolar of Ph_2PH in DMSO-d_6 using 5 mol% **1** – 0 h – $^{31}P\{^1H\}$ NMR.

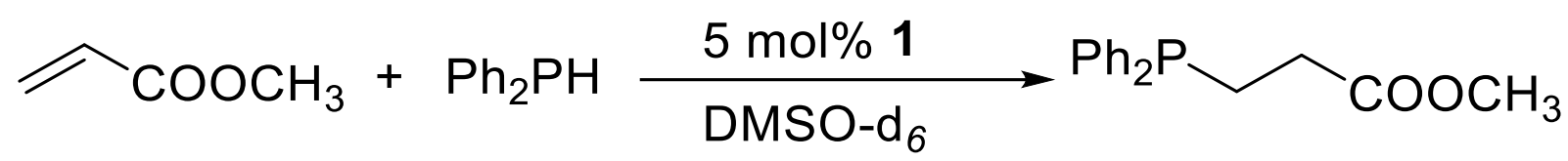

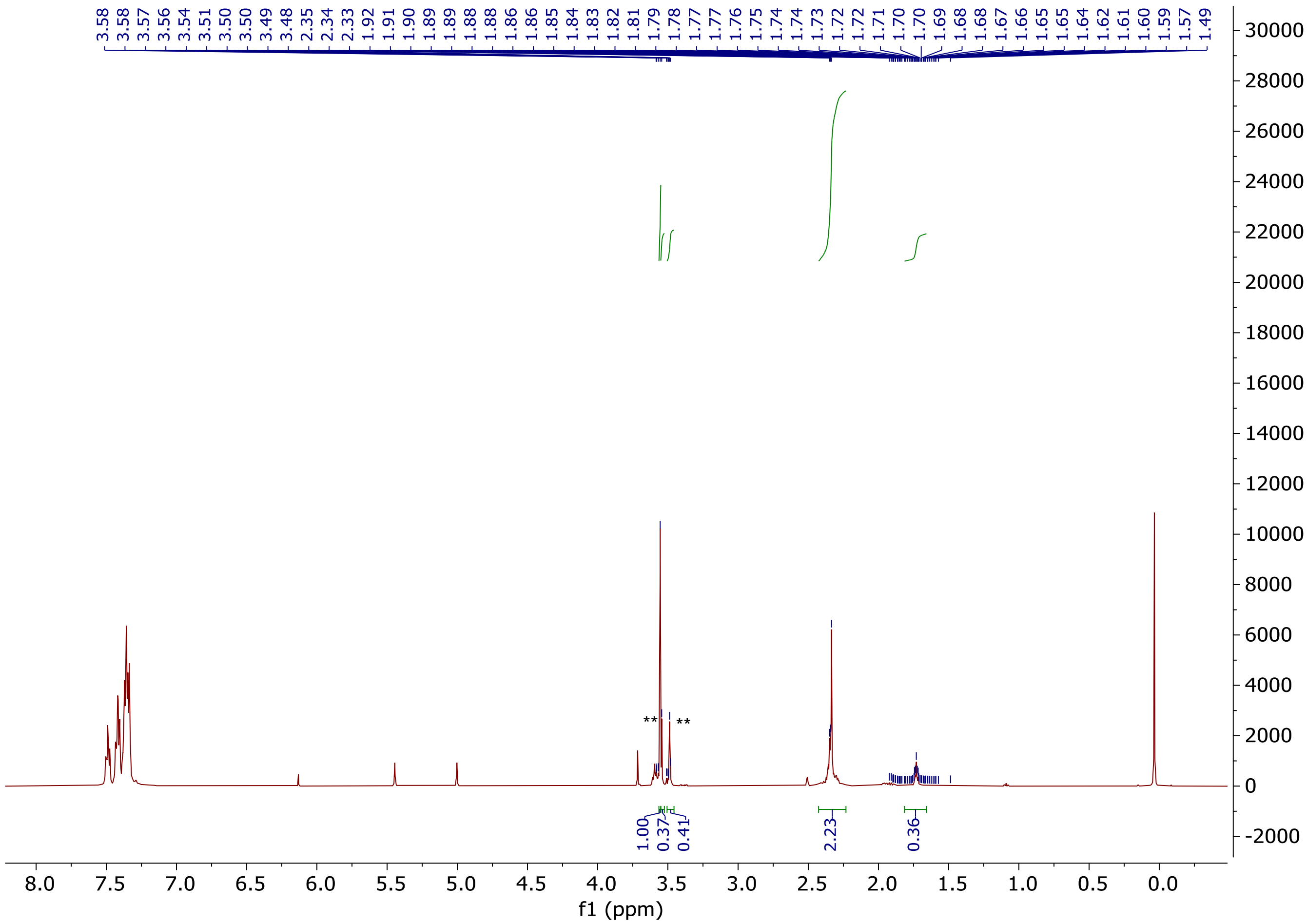

Figure S115: Hydrophosphination of 0.5 mmol methyl acrylate with equimolar of Ph_2PH in DMSO-d_6 using 5 mol% **1** – 0 h – ^1H NMR.

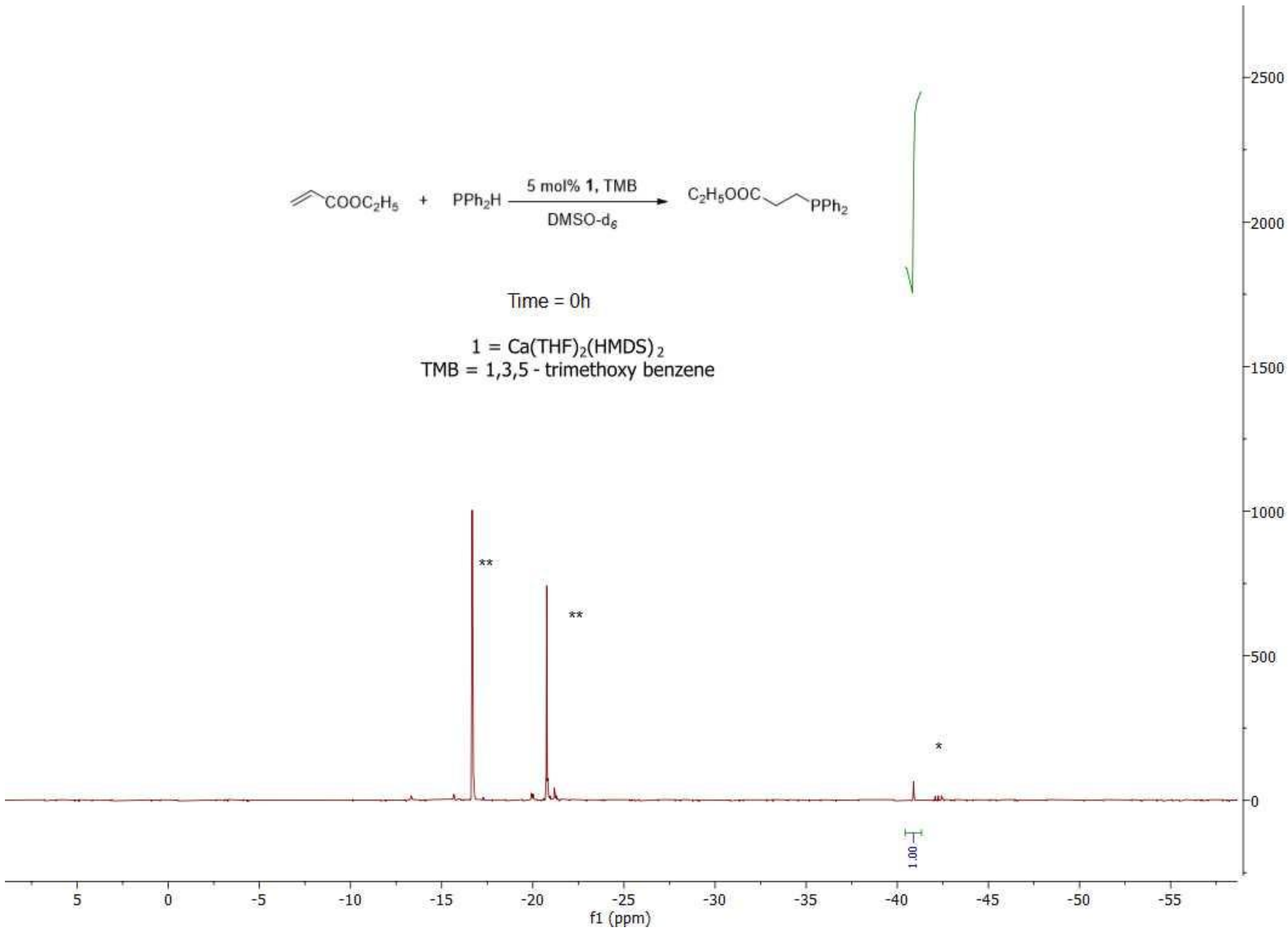

Figure S116: Hydrophosphination of 0.5 mmol ethyl acrylate with equimolar of Ph_2PH in DMSO-d_6 using 5 mol% **1** – 0 h – $^{31}P\{^1H\}$ NMR.

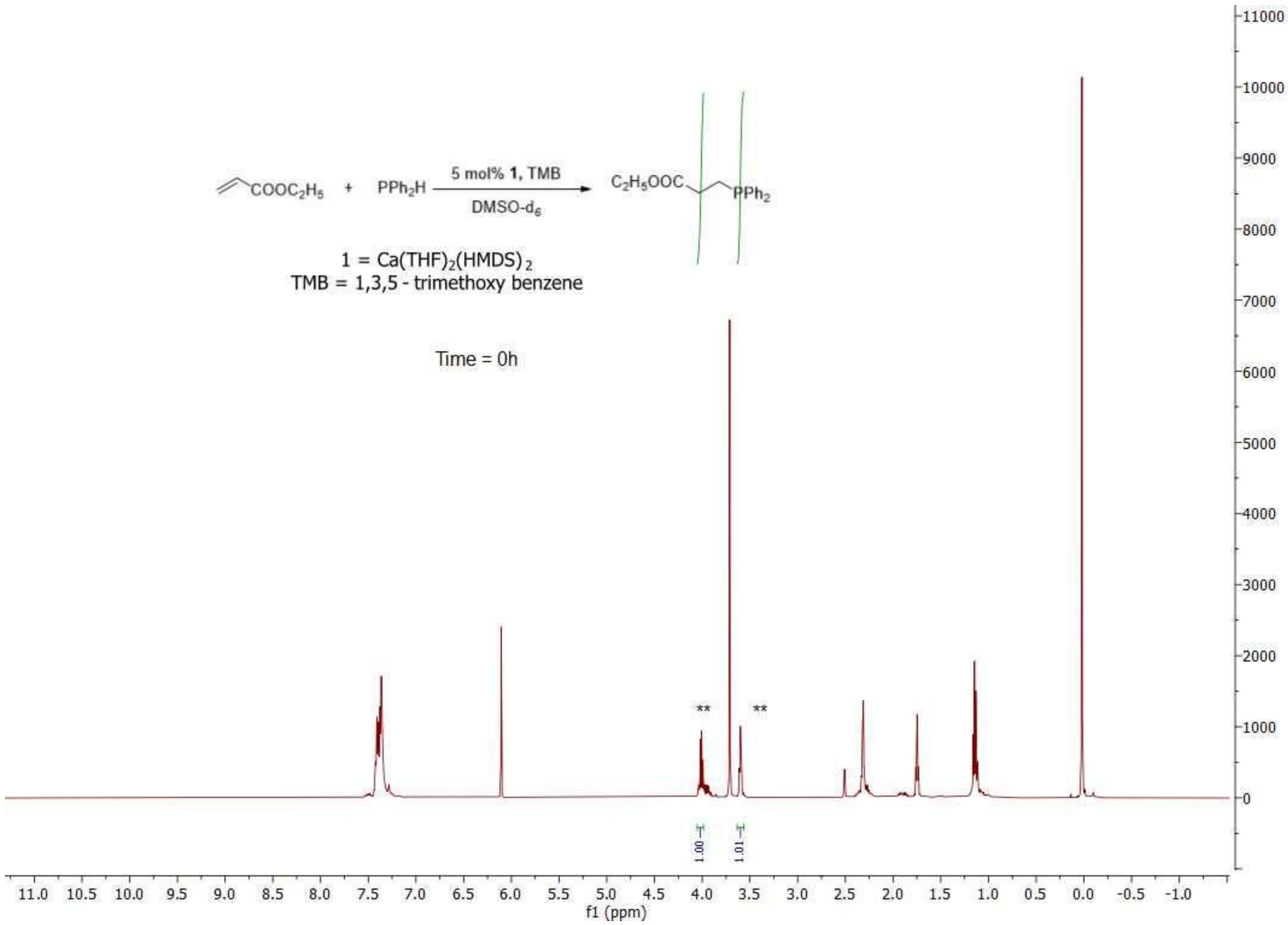

Figure S117: Hydrophosphination of 0.5 mmol ethyl acrylate with equimolar of Ph_2PH in DMSO-d_6 using 5 mol% **1** – 0 h – ^{1}H NMR.

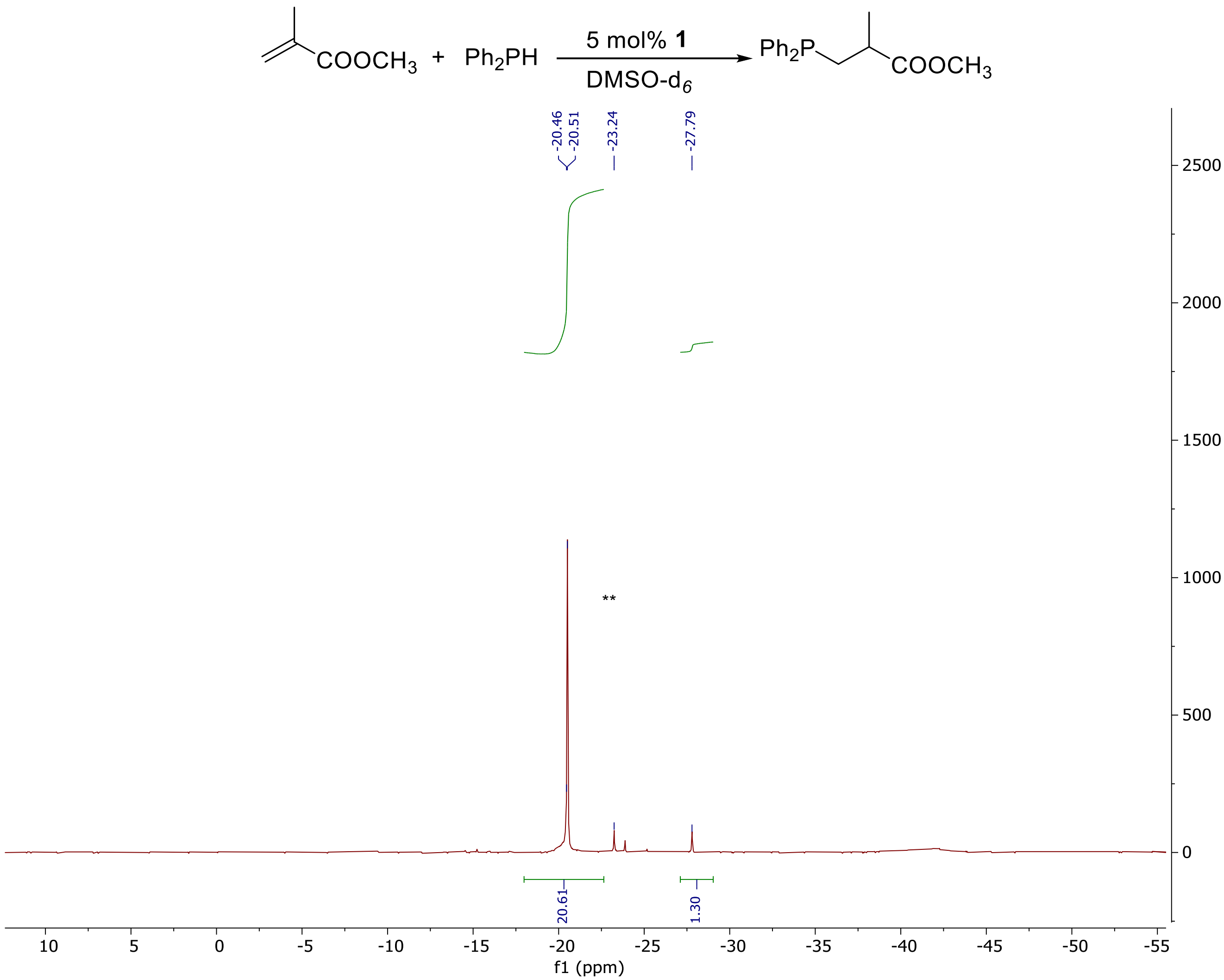

Figure S118: Hydrophosphination of 0.5 mmol methylmethacrylate with equimolar of Ph_2PH in DMSO-d_6 using 5 mol% **1** – 0 h –$^{31}P\{^1H\}$ NMR.

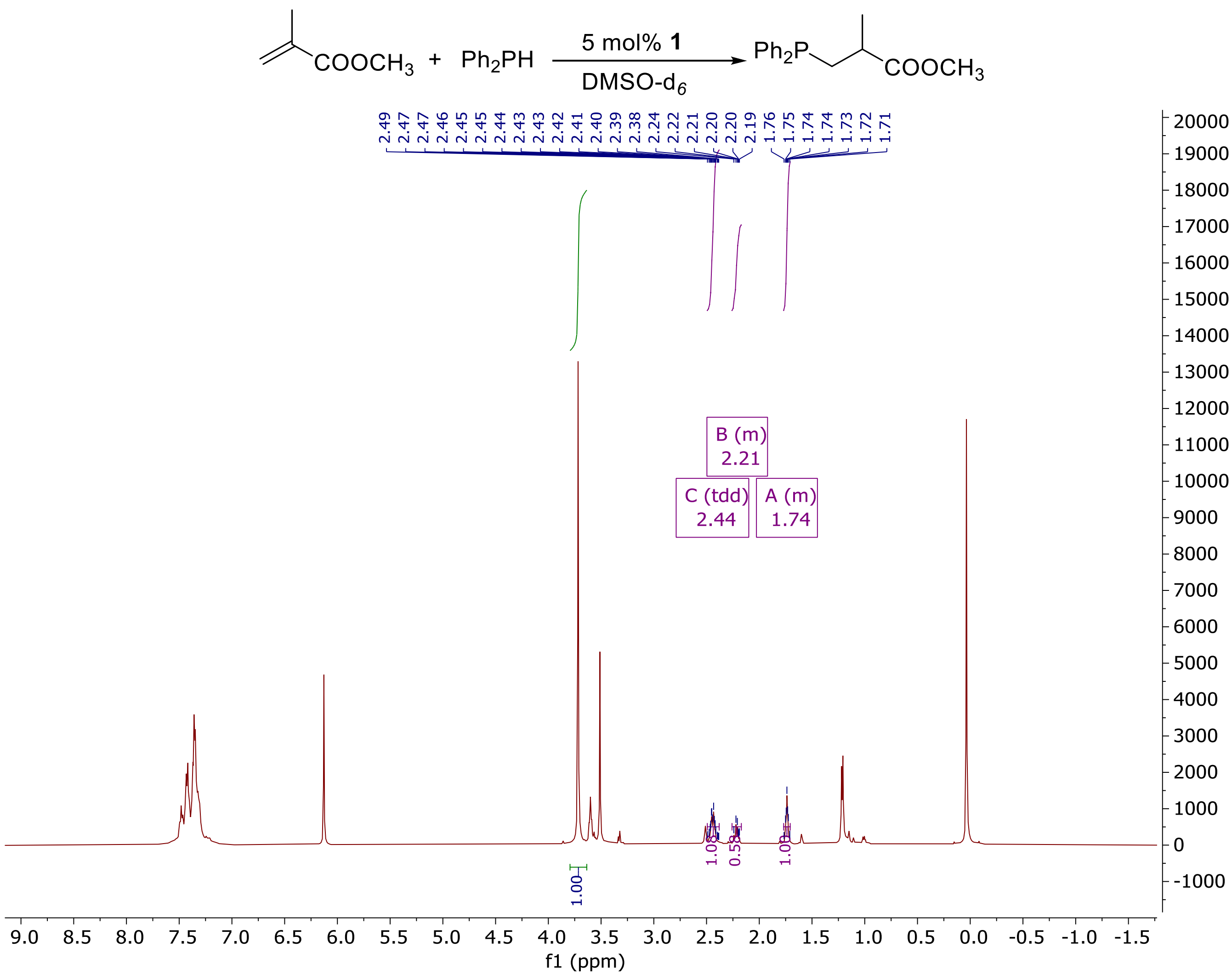

Figure S119: Hydrophosphination of 0.5 mmol methylmethacrylate with equimolar of Ph_2PH in $DMSO\text{-}d_6$ using 5 mol% **1** – 0 h –^1H NMR.

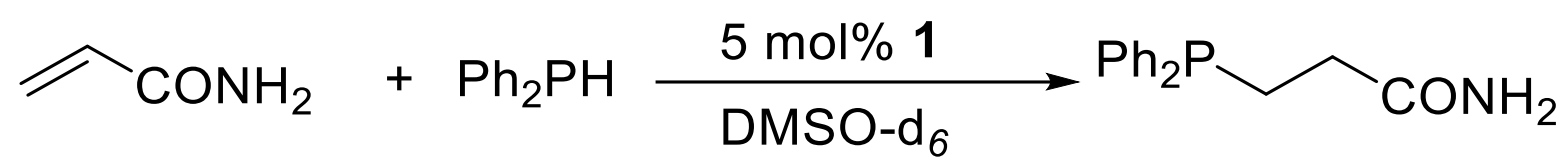

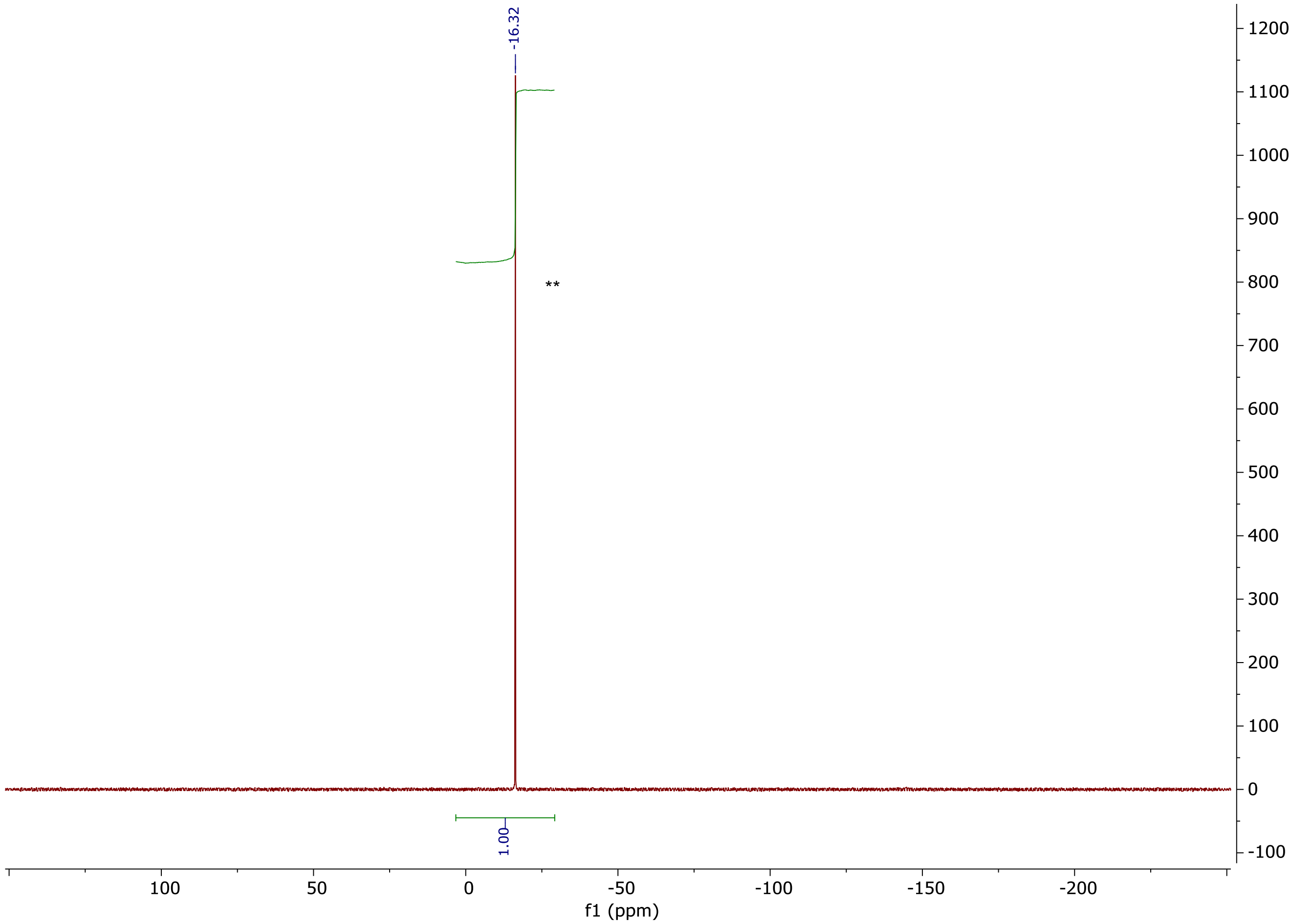

Figure S120: Hydrophosphination of 0.5 mmol acrylamide with equimolar of Ph_2PH in DMSO-d_6 using 5 mol% **1** – 0 h –$^{31}P\{^1H\}$ NMR.

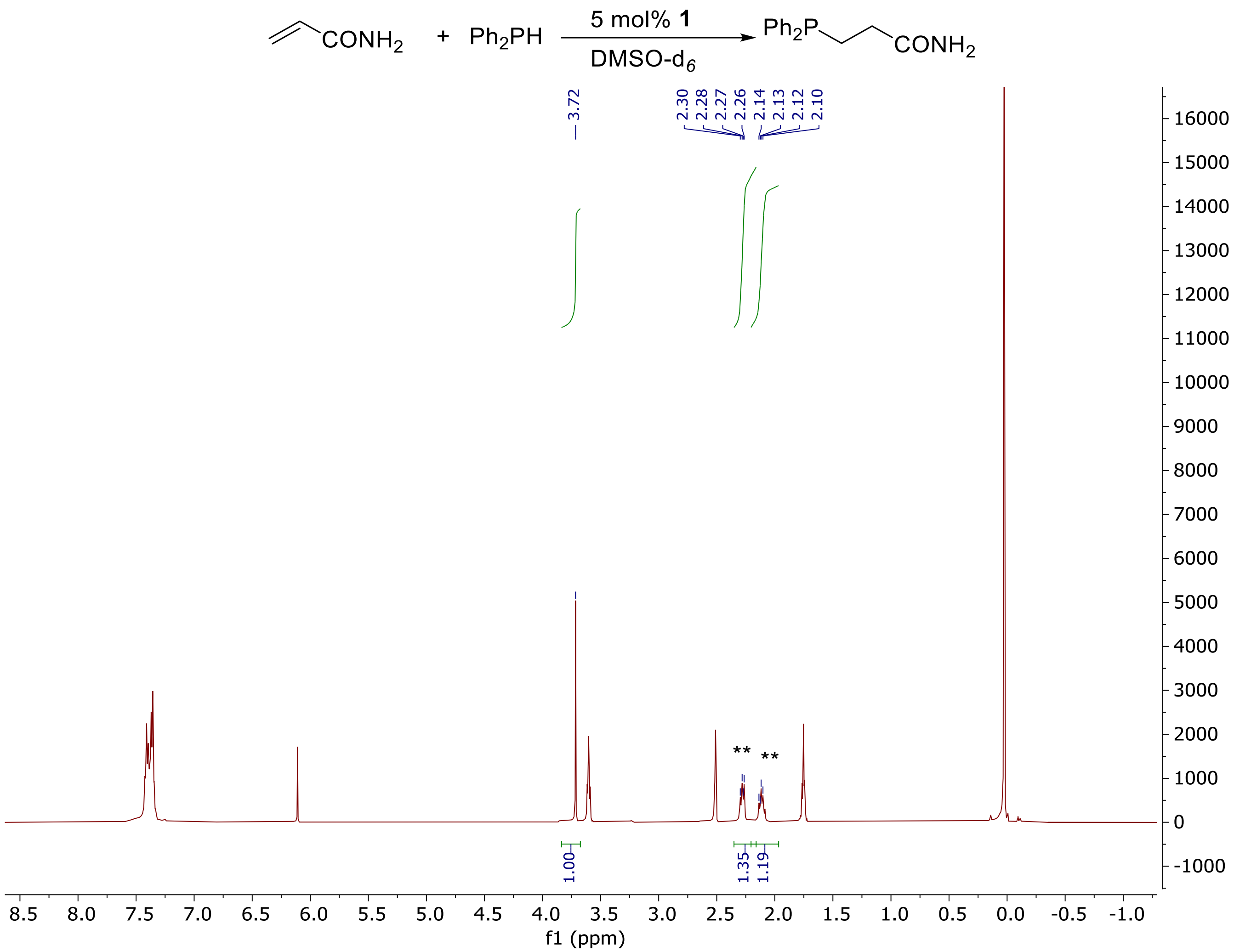

Figure S121: Hydrophosphination of 0.5 mmol acrylamide with equimolar of Ph_2PH in DMSO-d_6 using 5 mol% **1** – 0 h –^{1}H NMR.

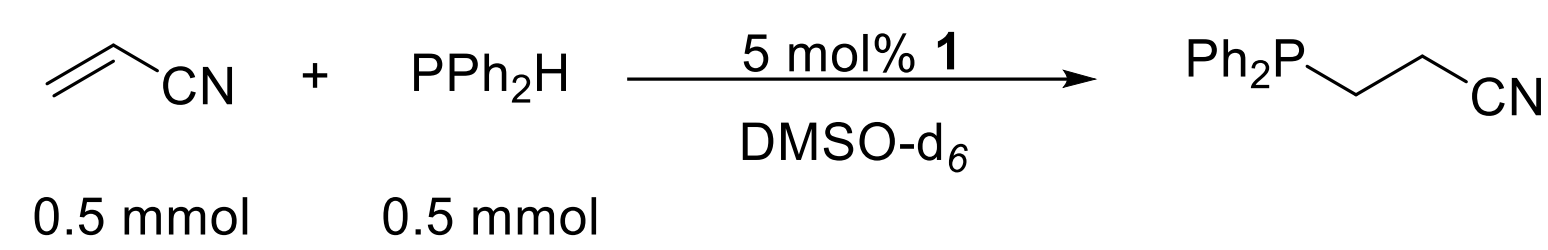

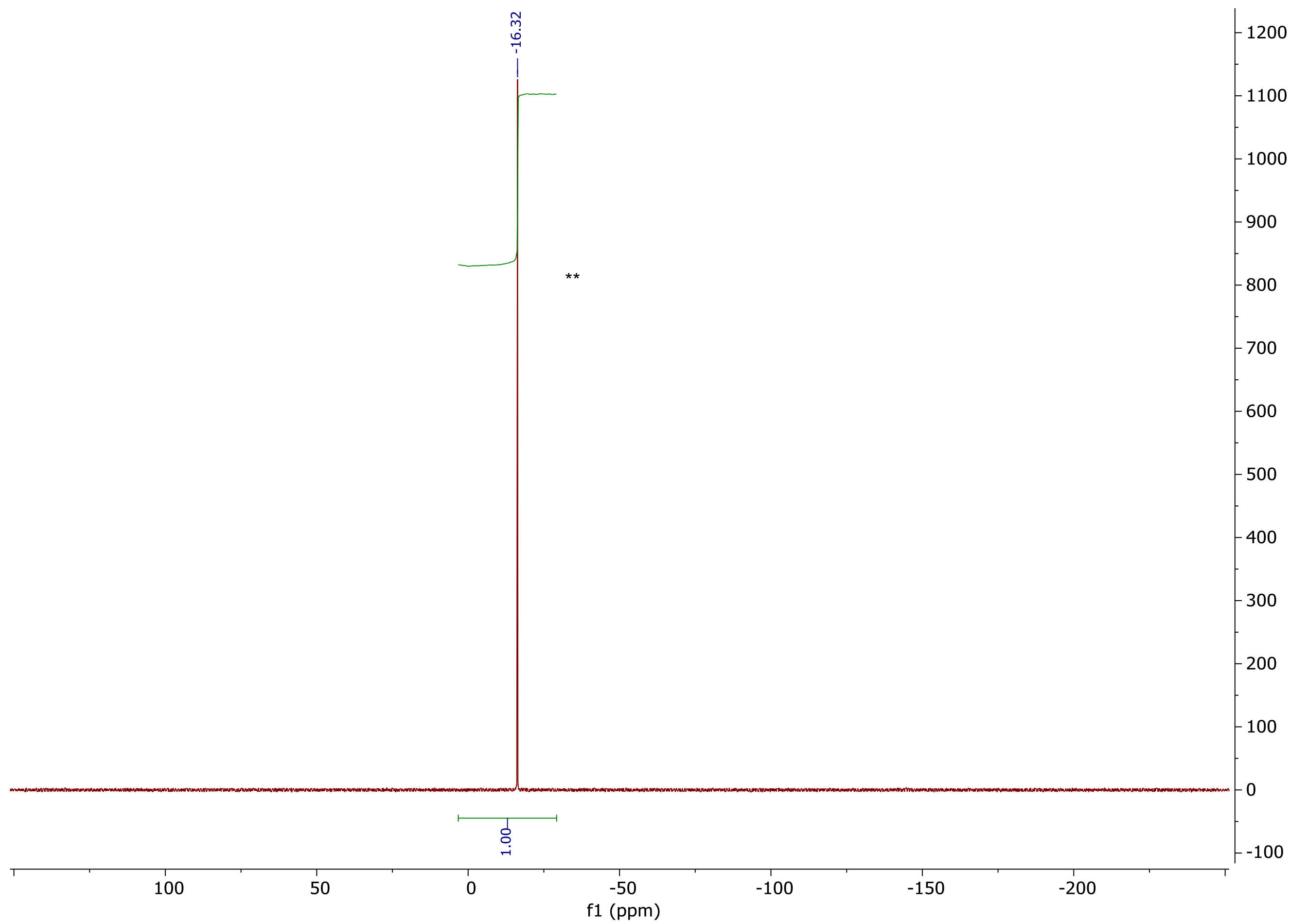

Figure S122: Hydrophosphination of 0.5 mmol acrylonitrile with equimolar of Ph_2PH in DMSO-d_6 using 5 mol% **1** – 0 h – $^{31}P\{^1H\}$ NMR.

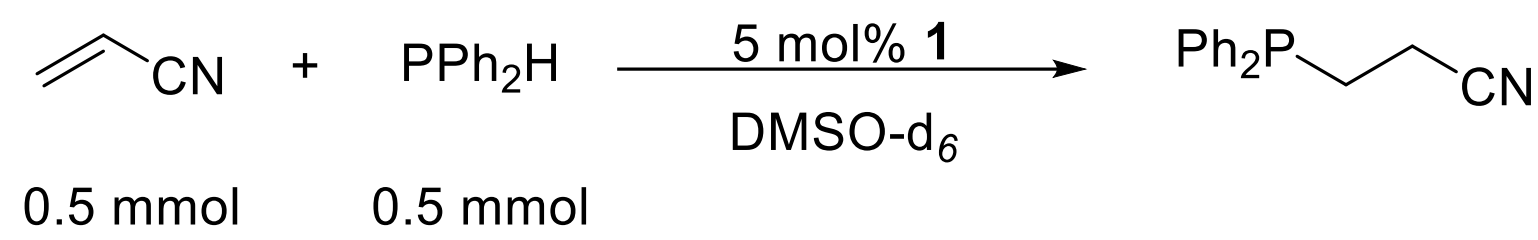

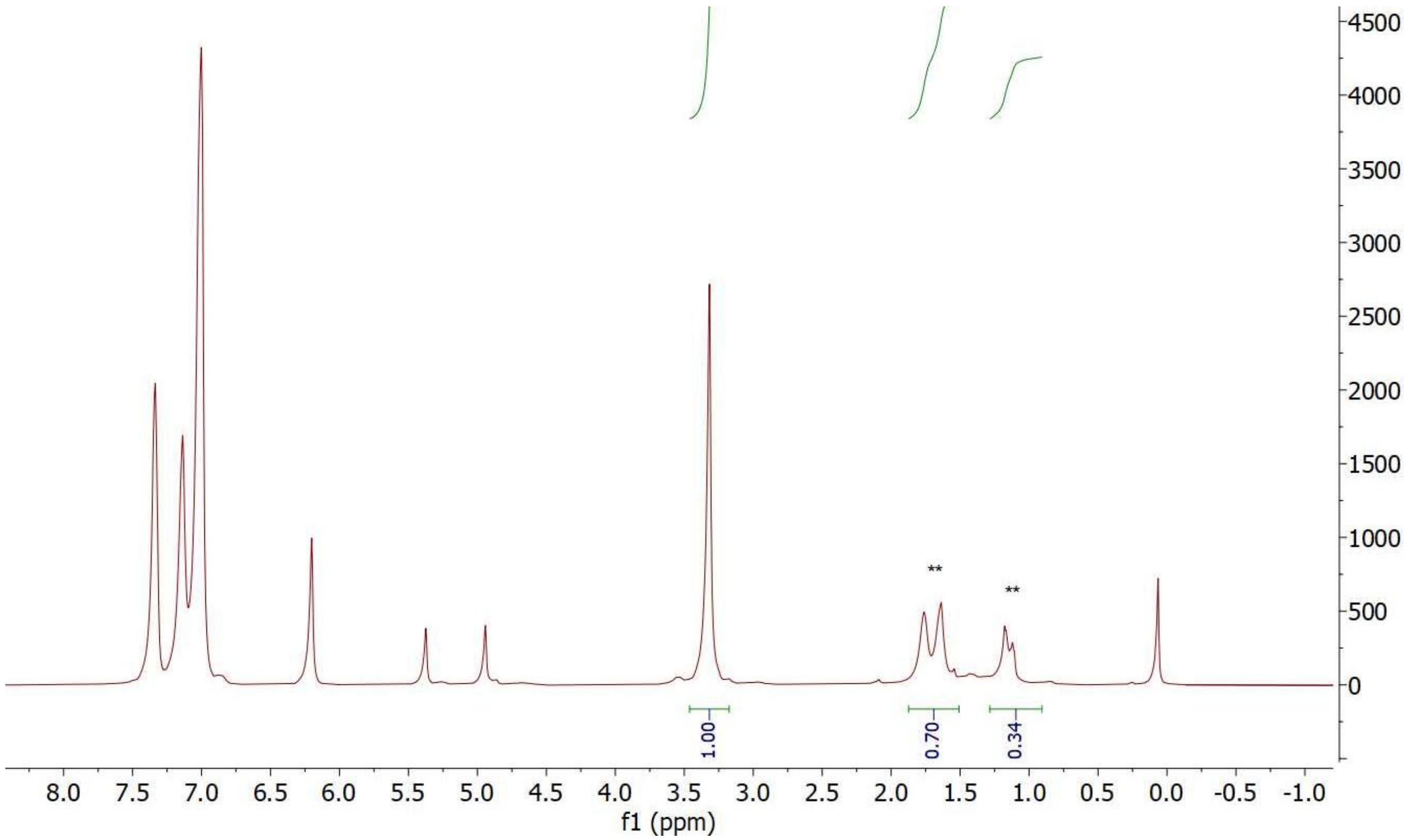

Figure S123: Hydrophosphination of 0.5 mmol acrylonitrile with equimolar of Ph_2PH in DMSO-d_6 using 5 mol% **1** – 0 h –^{1}H NMR.

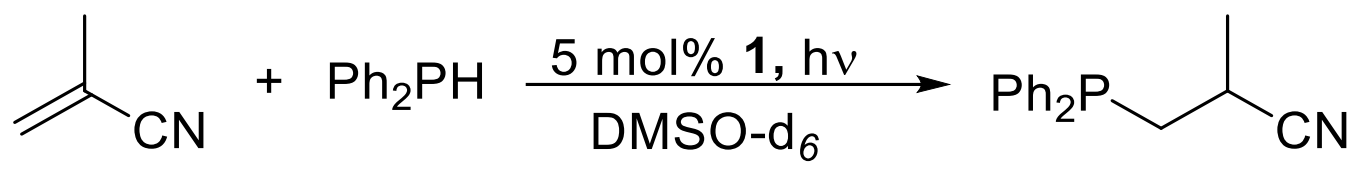

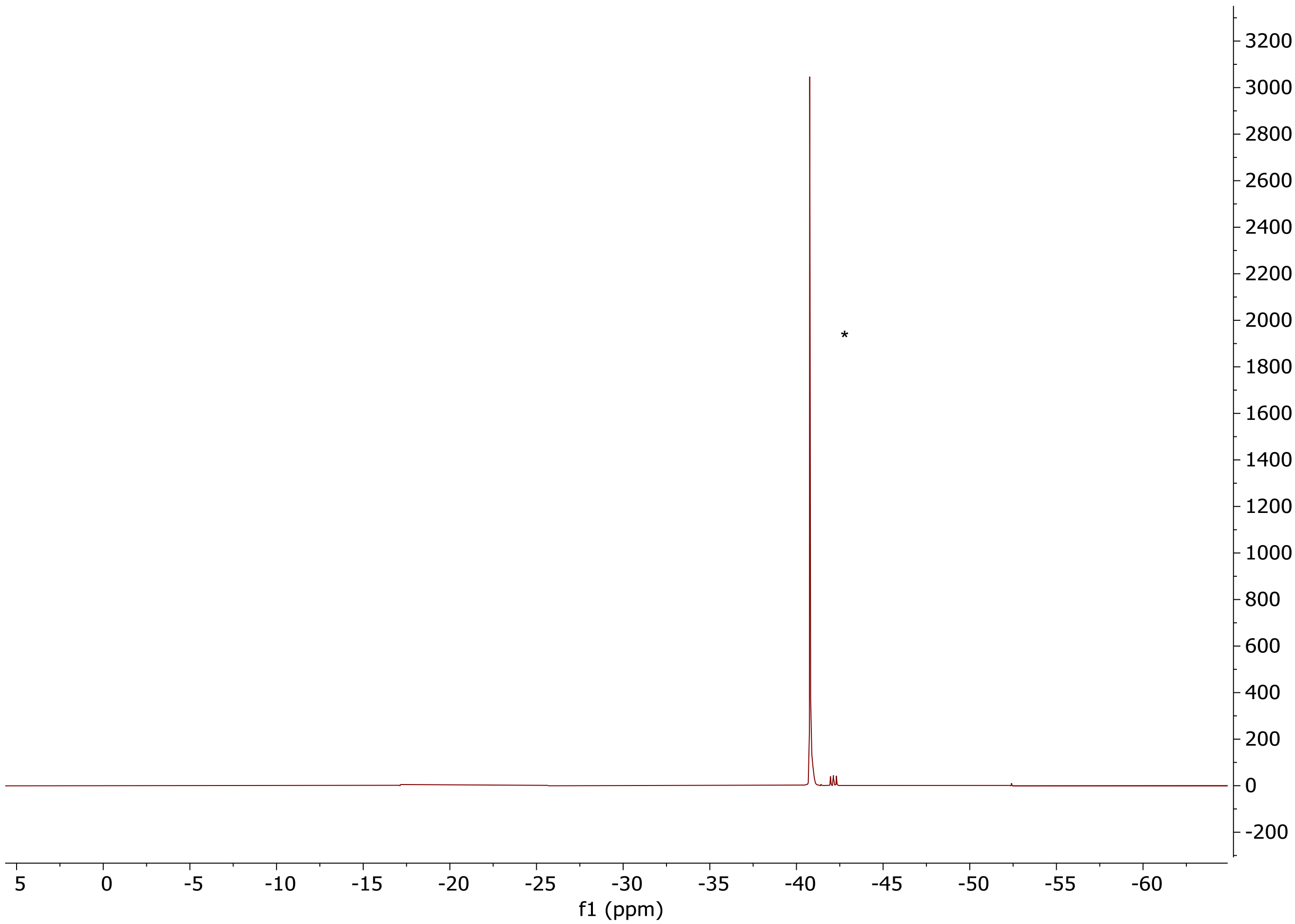

Figure S124: Hydrophosphination of 0.5 mmol methacrylonitrile with equimolar of Ph_2PH in DMSO-d_6 using 5 mol% **1** – 24 h – $^{31}P\{^1H\}$ NMR.

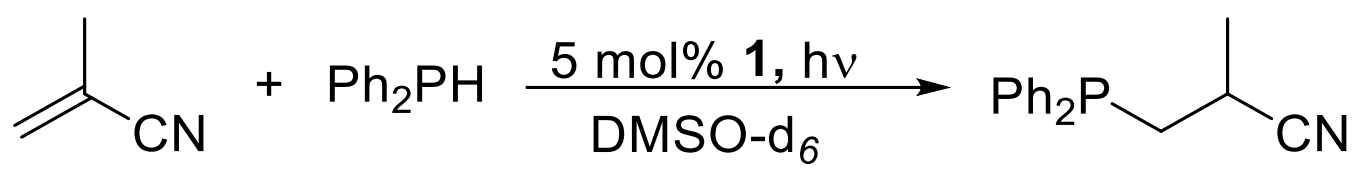

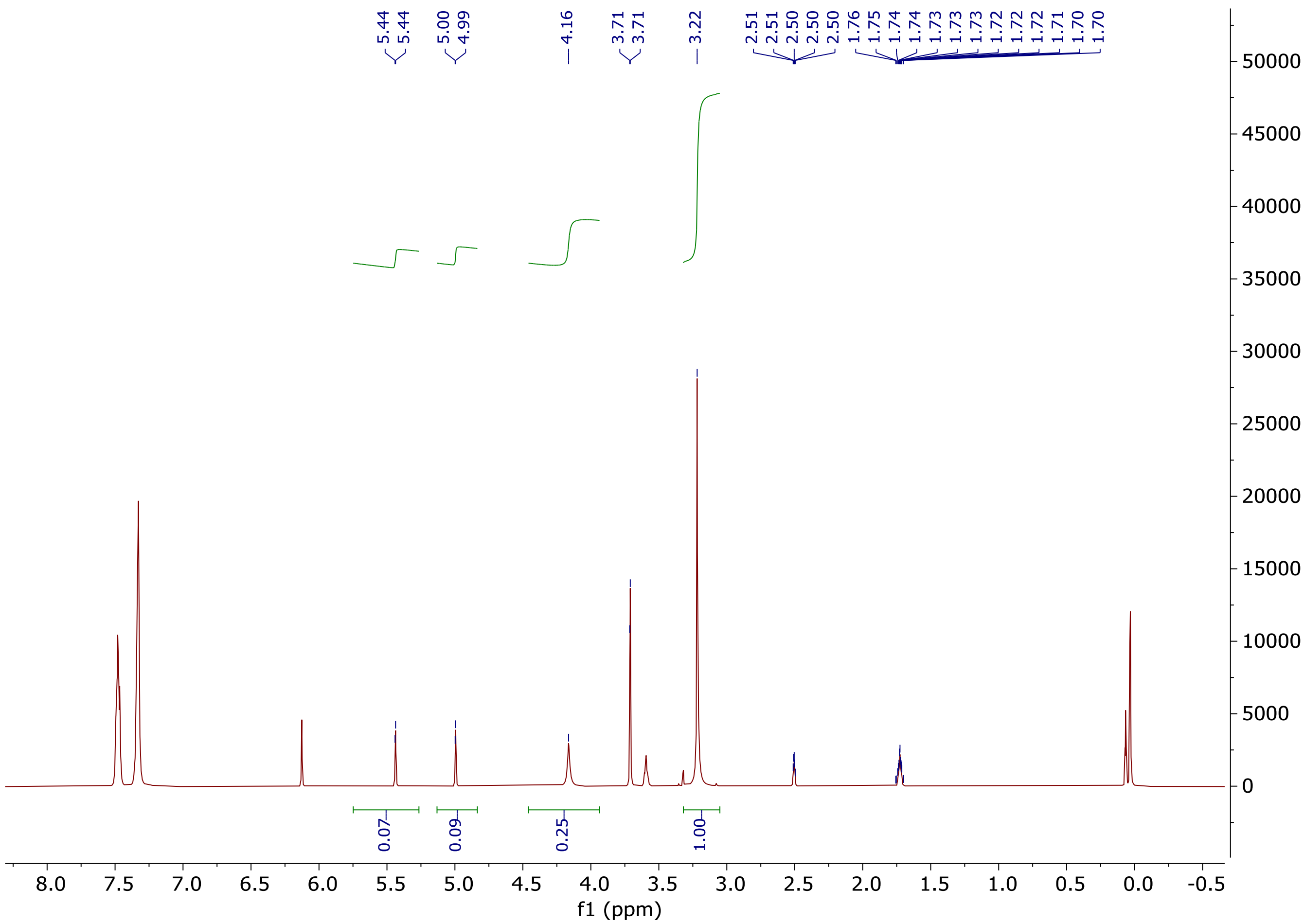

Figure S125: Hydrophosphination of 0.5 mmol methacrylonitrile with equimolar of Ph_2PH in DMSO-d_6 using 5 mol% **1** – 24 h –^{1}H NMR.

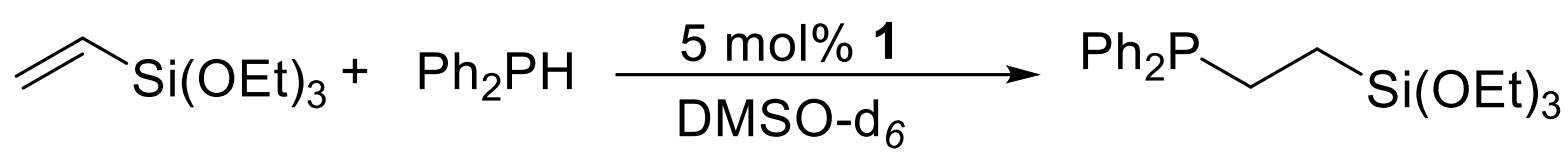

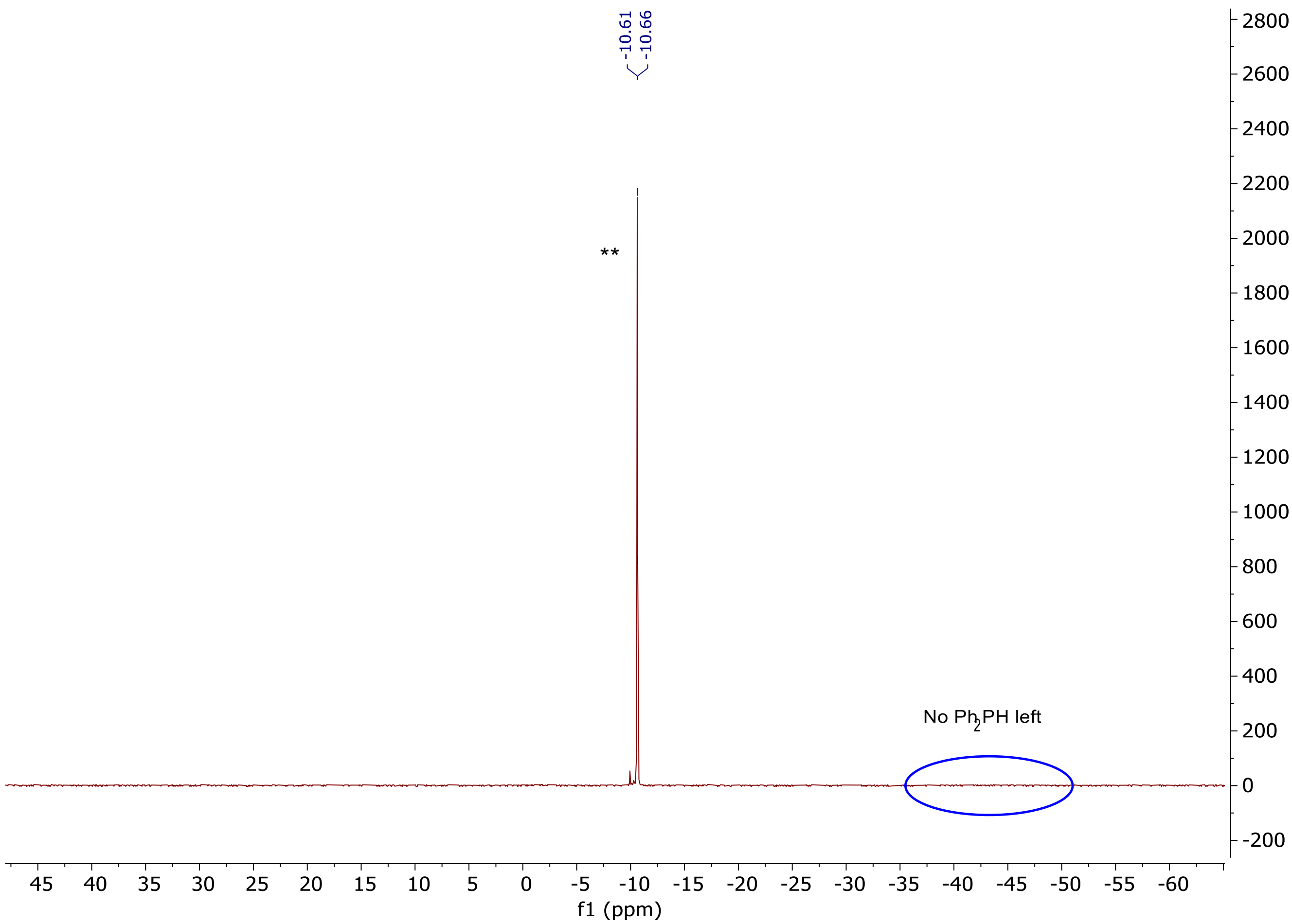

Figure S126: Hydrophosphination of 0.5 mmol vinyl triethoxysilane with equimolar of Ph_2PH in DMSO-d_6 using 5 mol% **1** – 0 h – $^{31}P\{^1H\}$ NMR.

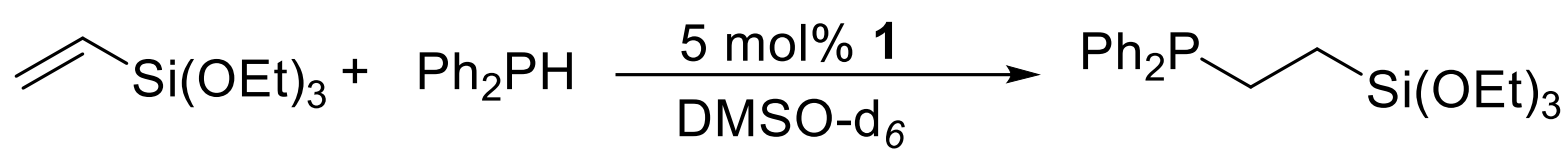

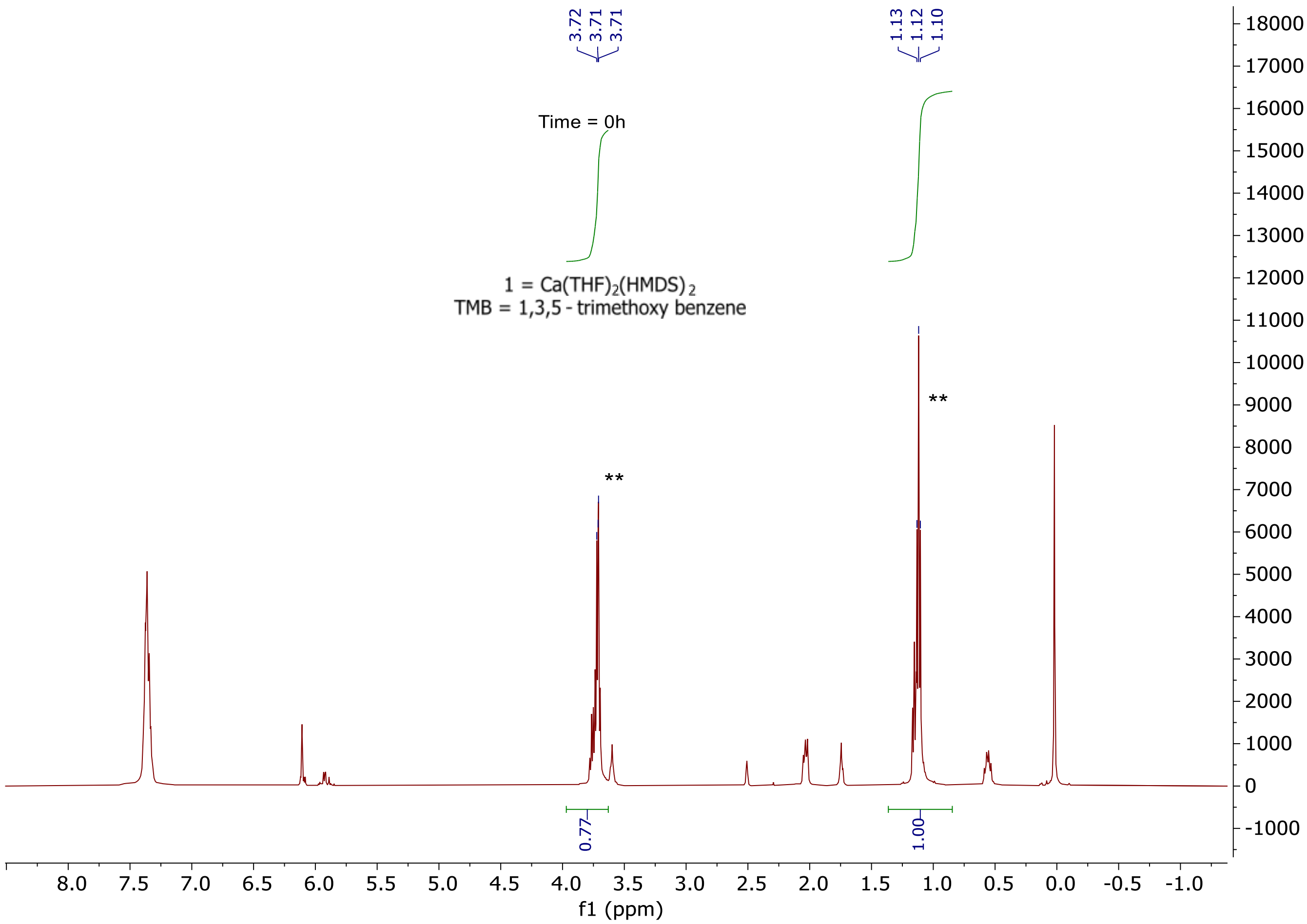

Figure S127: Hydrophosphination of 0.5 mmol vinyl triethoxysilane with equimolar of Ph_2PH in DMSO-d_6 using 5 mol% **1** – 0 h –^{1}H NMR.

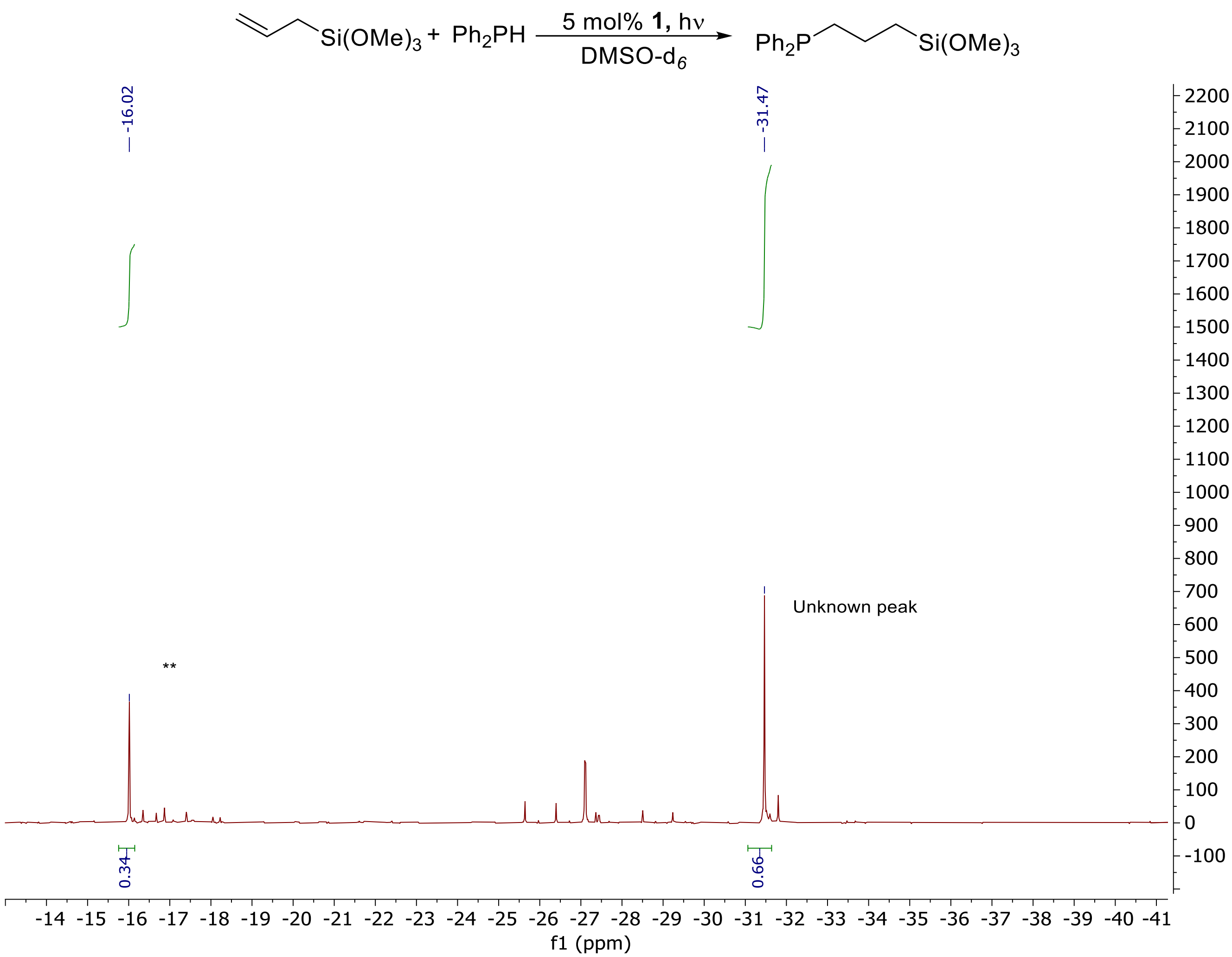

Figure S128: Hydrophosphination of 0.5 mmol allyl trimethoxysilane with equimolar of Ph_2PH in DMSO-d_6 using 5 mol% **1** under UV 360 nm irradiation – 24 h –$^{31}P\{^1H\}$ NMR.

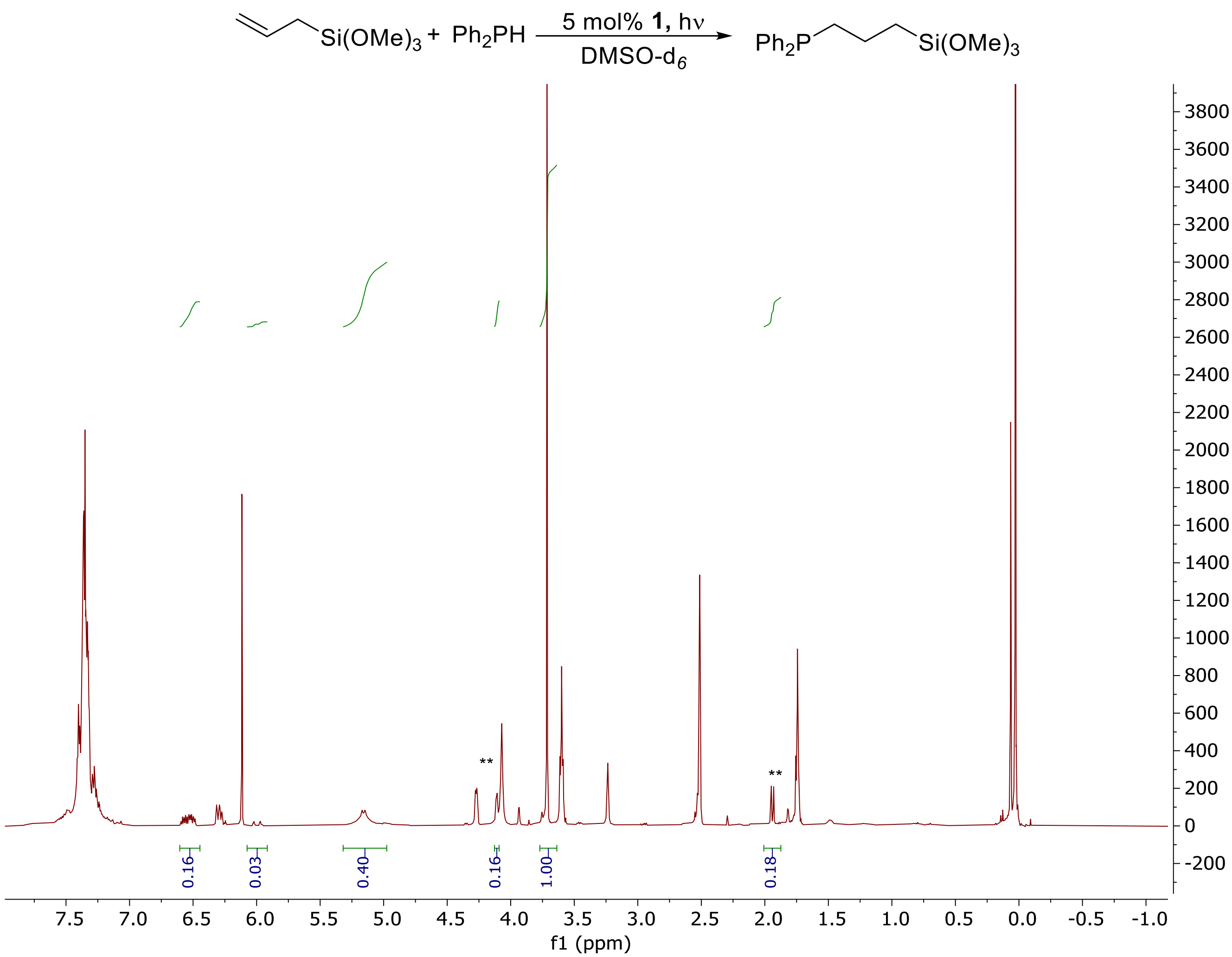

Figure S129: Hydrophosphination of 0.5 mmol allyl trimethoxy silane with equimolar of Ph_2PH in DMSO-d_6 using 5 mol% **1** under UV 360 nm irradiation – 24 h –^{1}H NMR.

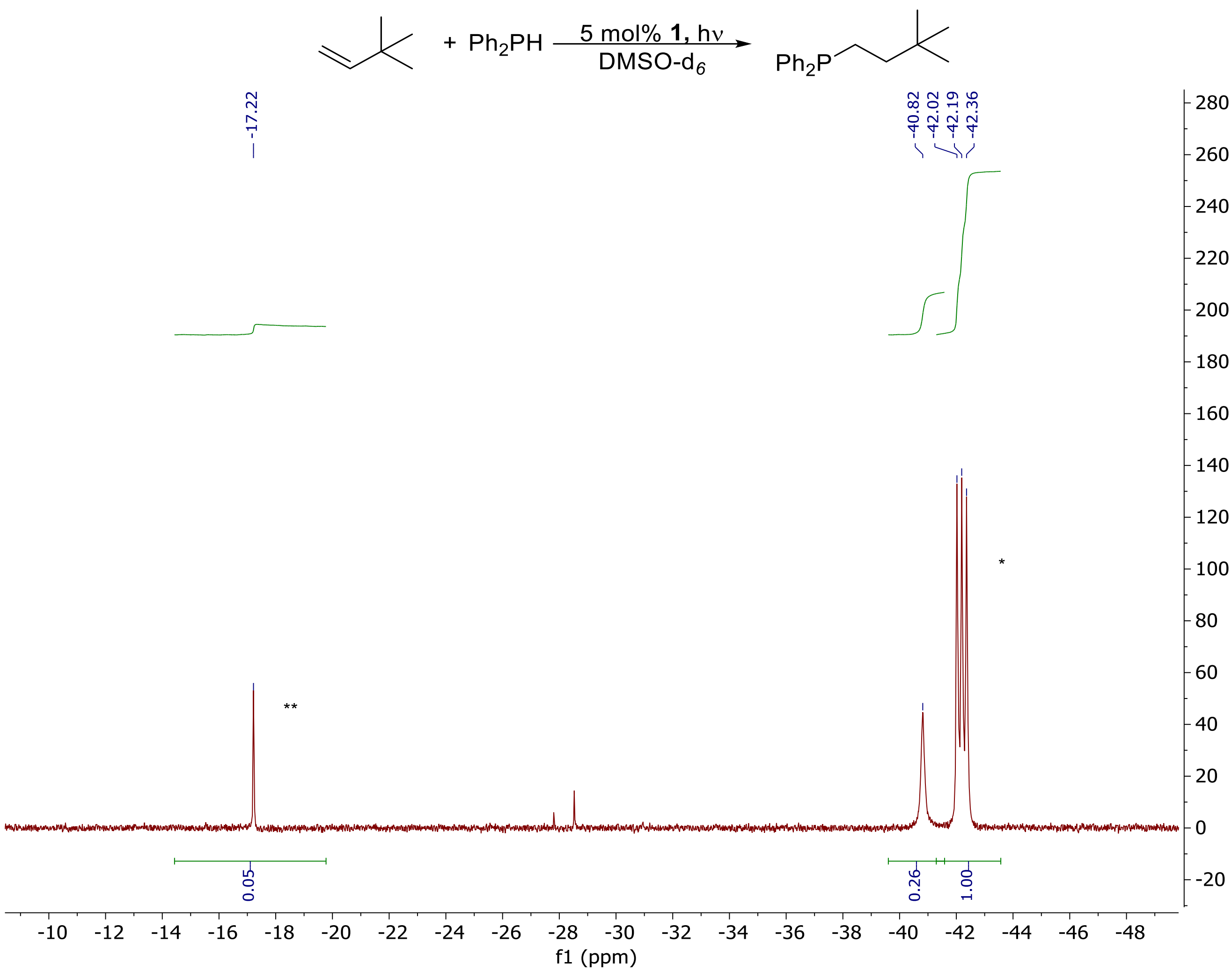

Figure S130: Hydrophosphination of 0.5 mmol tert-butyl ethylene with equimolar of Ph_2PH in DMSO-d_6 using 5 mol% **1** under UV 360 nm irradiation - 24 h – $^{31}P\{^1H\}$ NMR.

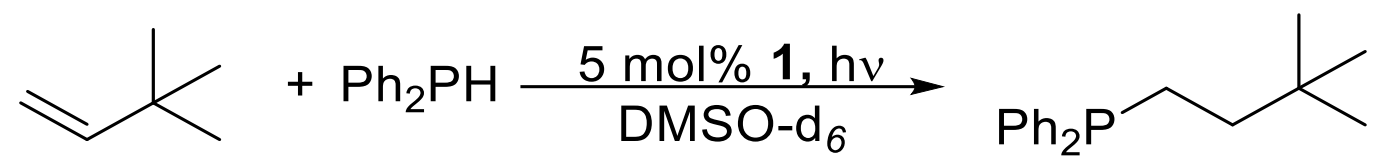

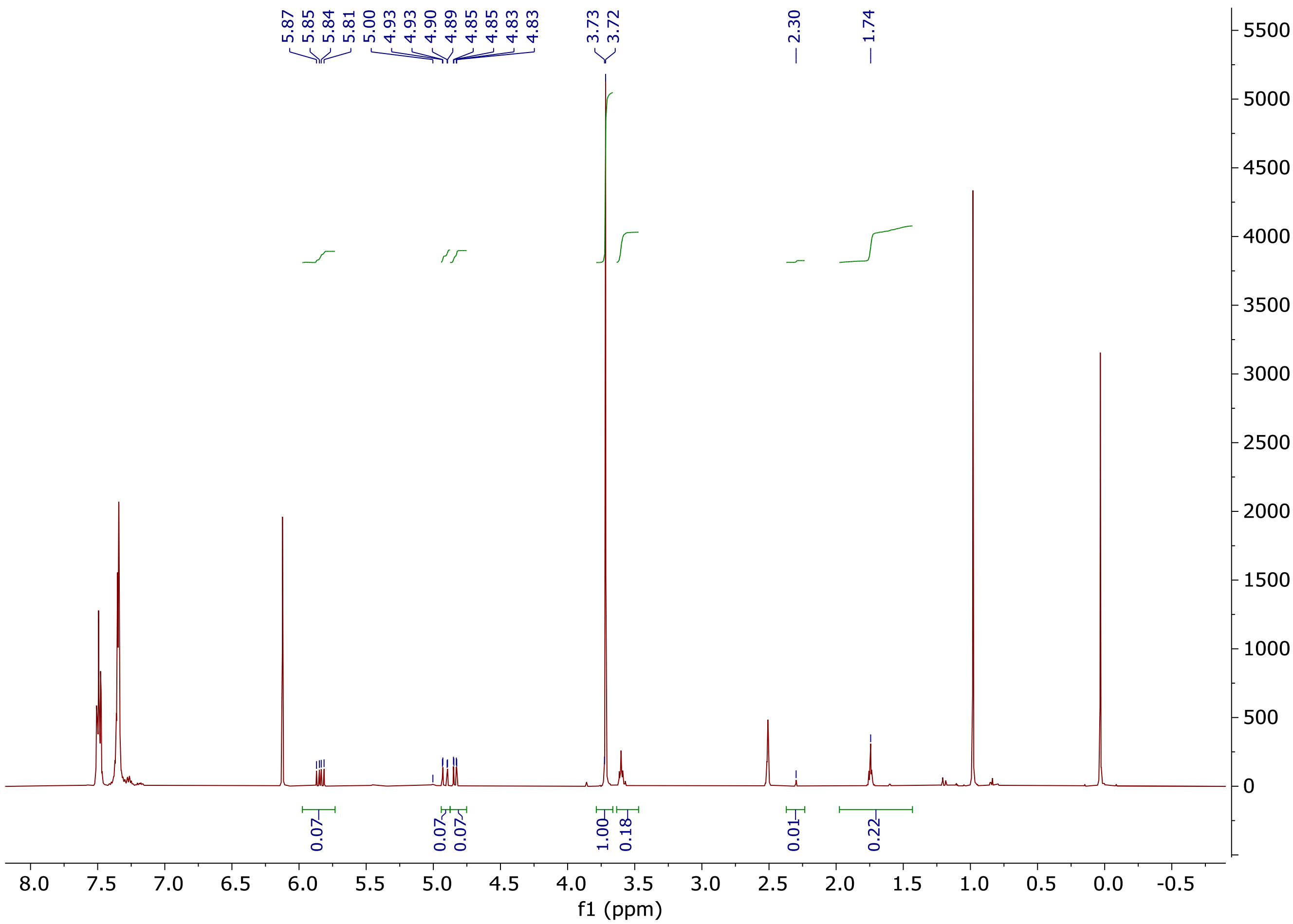

Figure S131: Hydrophosphination of 0.5 mmol tert-butyl ethylene with equimolar of Ph_2PH in DMSO-d_6 using 5 mol% **1** under UV 360 nm irradiation – 24 h –^{1}H NMR.

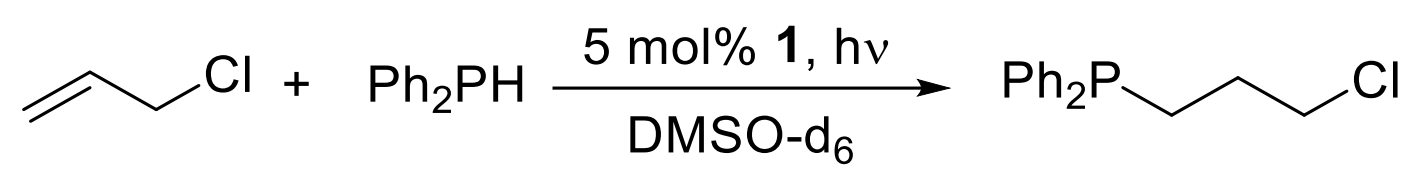

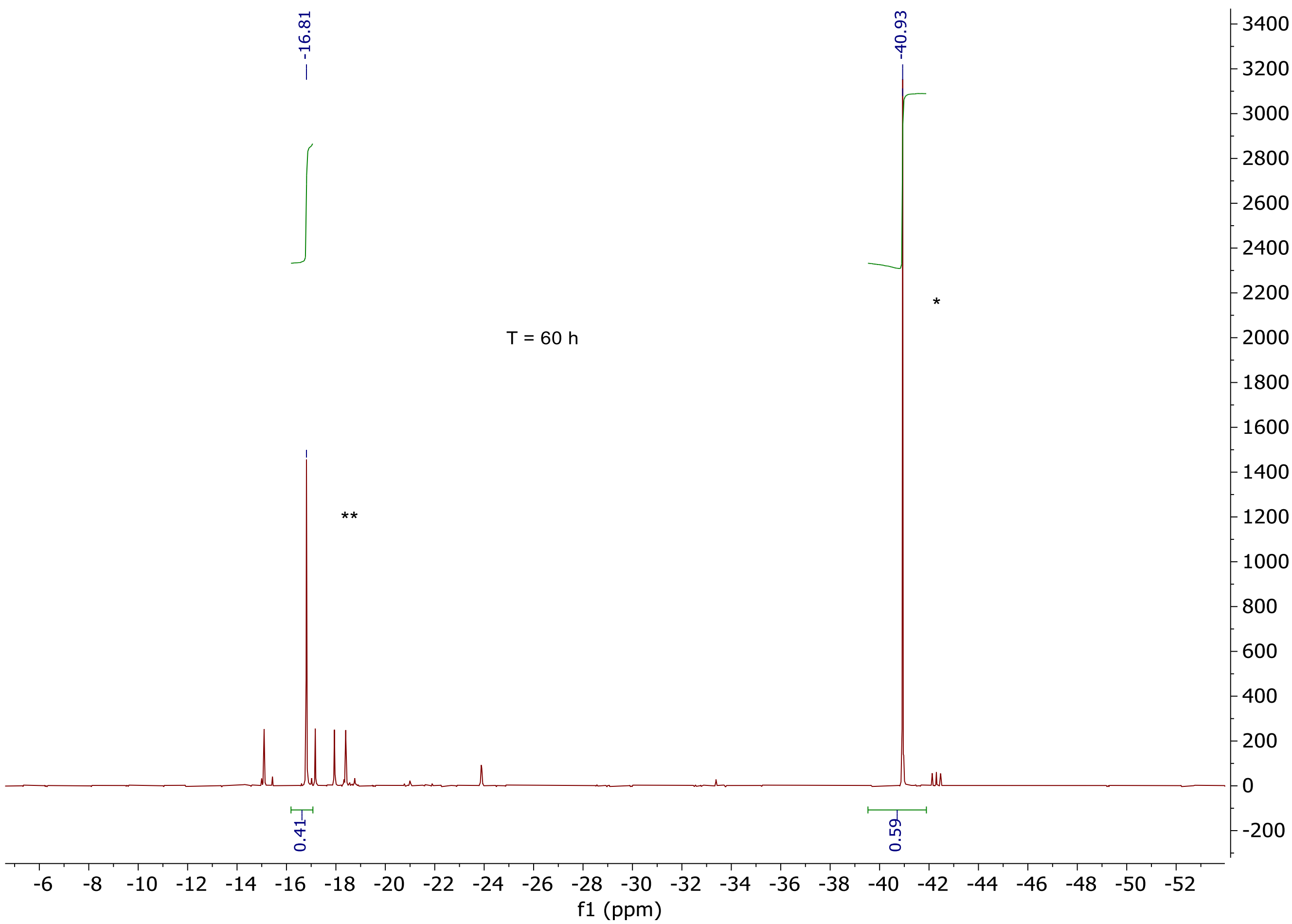

Figure S132: Hydrophosphination of 0.5 mmol allyl chloride with equimolar of Ph_2PH in DMSO-d_6 using 5 mol% **1** under UV 360 nm irradiation – 60 h – $^{31}P\{^1H\}$ NMR.

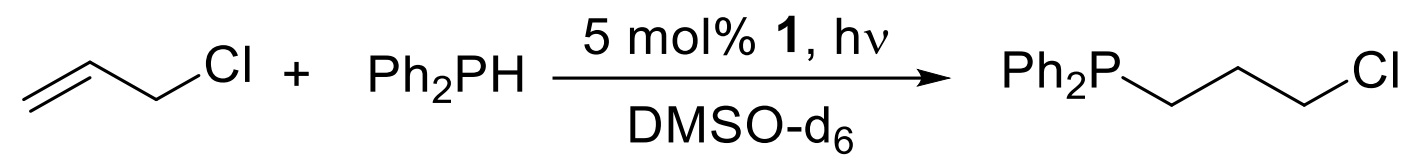

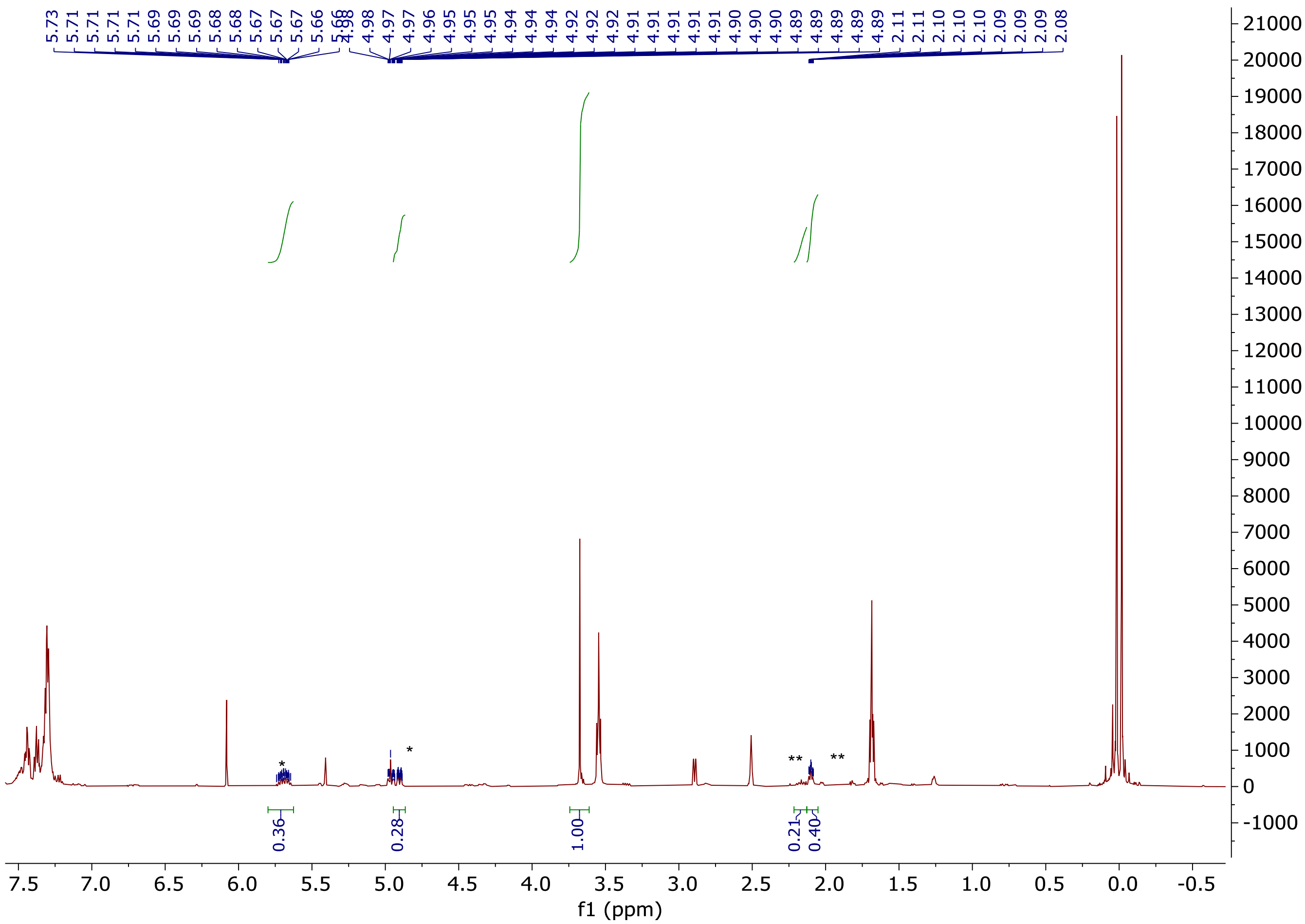

Figure S133: Hydrophosphination of 0.5 mmol allyl chloride with equimolar of Ph_2PH in DMSO-d_6 using 5 mol% **1** under UV 360 nm irradiation – 60 h –^{1}H NMR.

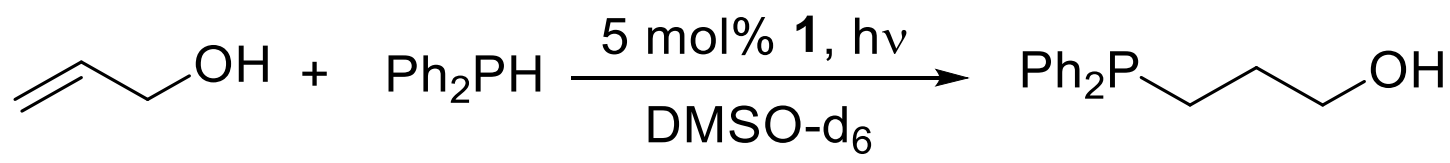

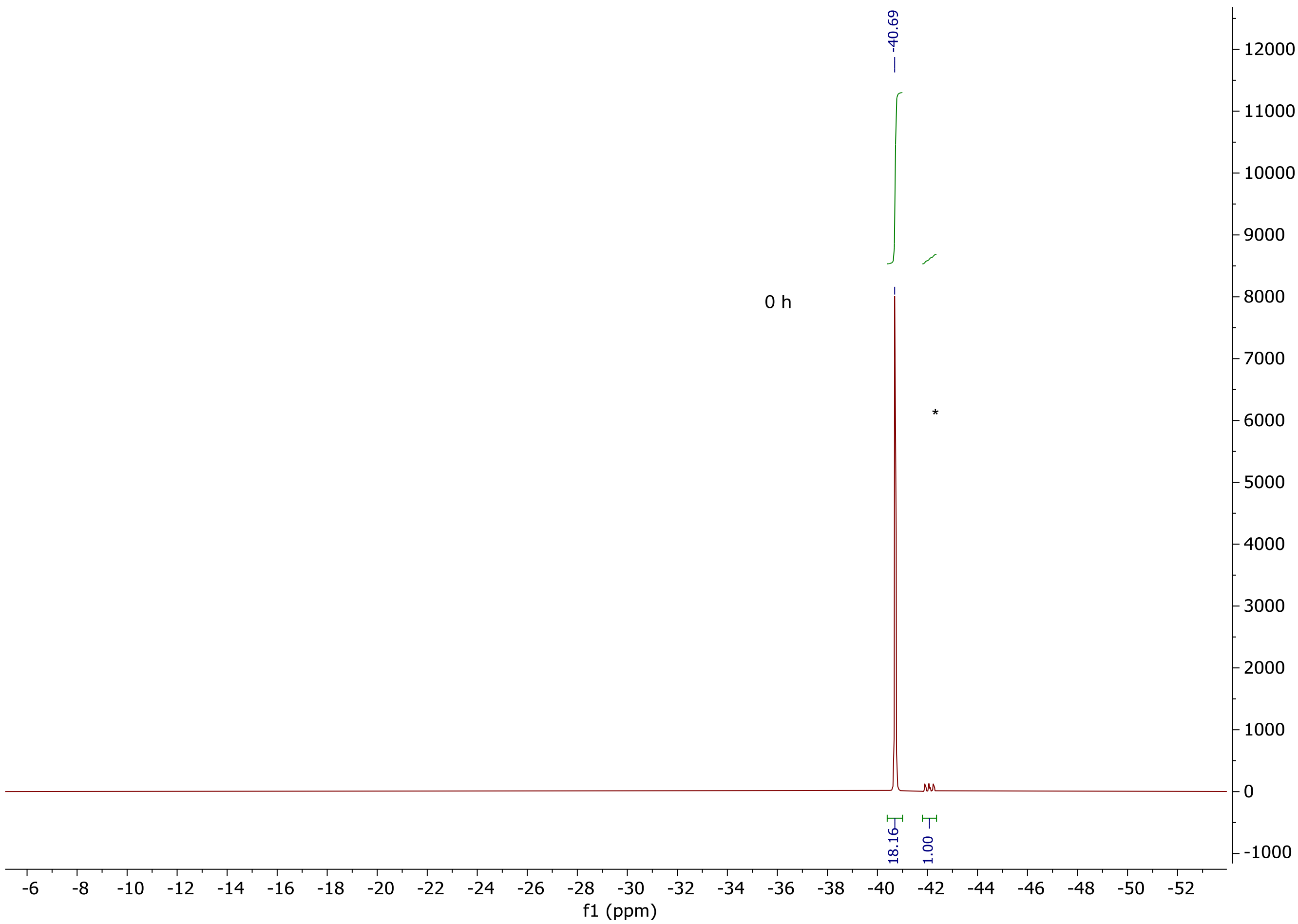

Figure S134: Hydrophosphination of 0.5 mmol allyl alcohol with equimolar of Ph_2PH in DMSO-d_6 using 5 mol% **1** under UV 360 nm irradiation – 0 h –$^{31}P\{^1H\}$ NMR.

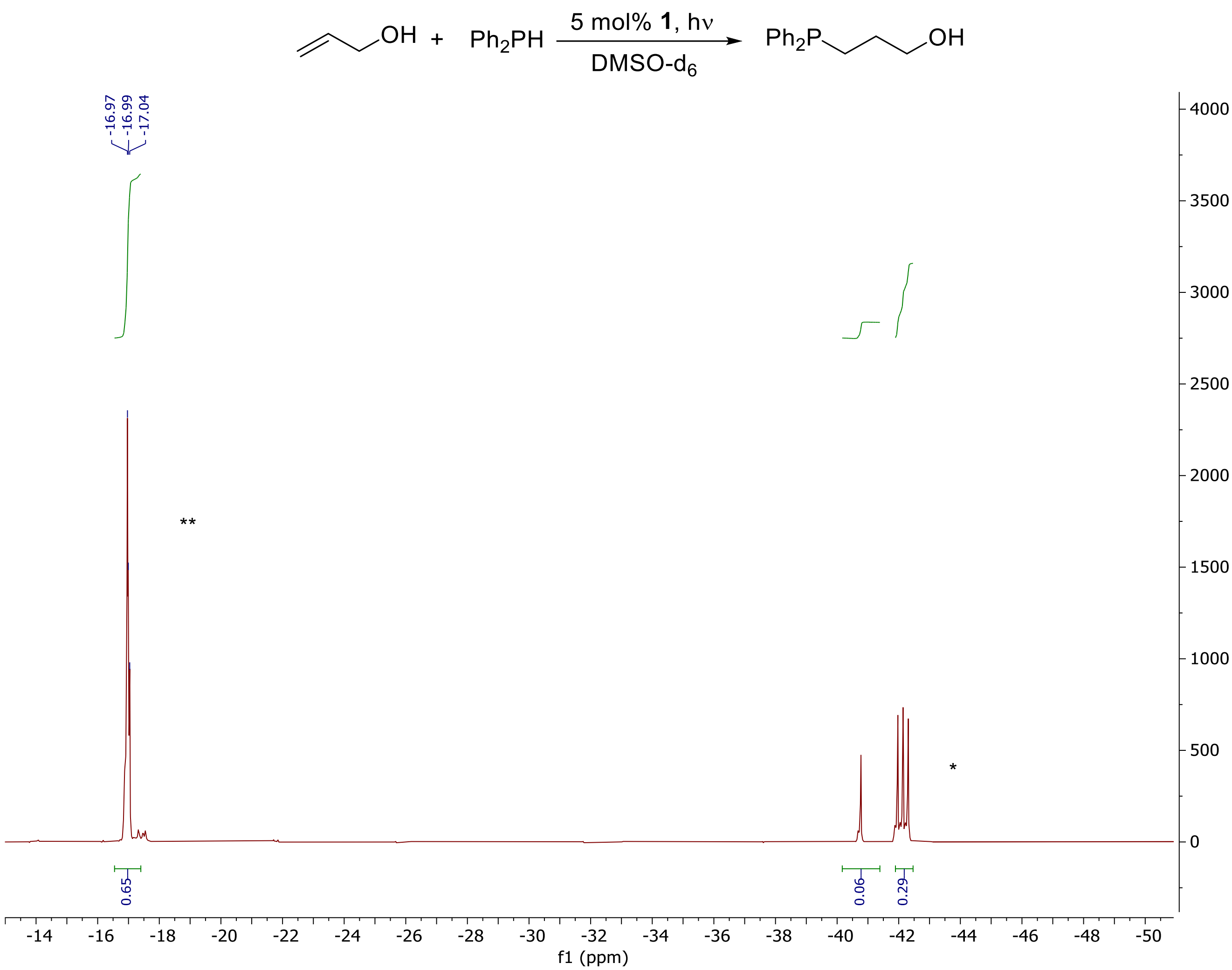

Figure S135: Hydrophosphination of 0.5 mmol allyl alcohol with equimolar of Ph_2PH in DMSO-d_6 using 5 mol% **1** under UV 360 nm irradiation – 72 h –$^{31}P\{^1H\}$ NMR.

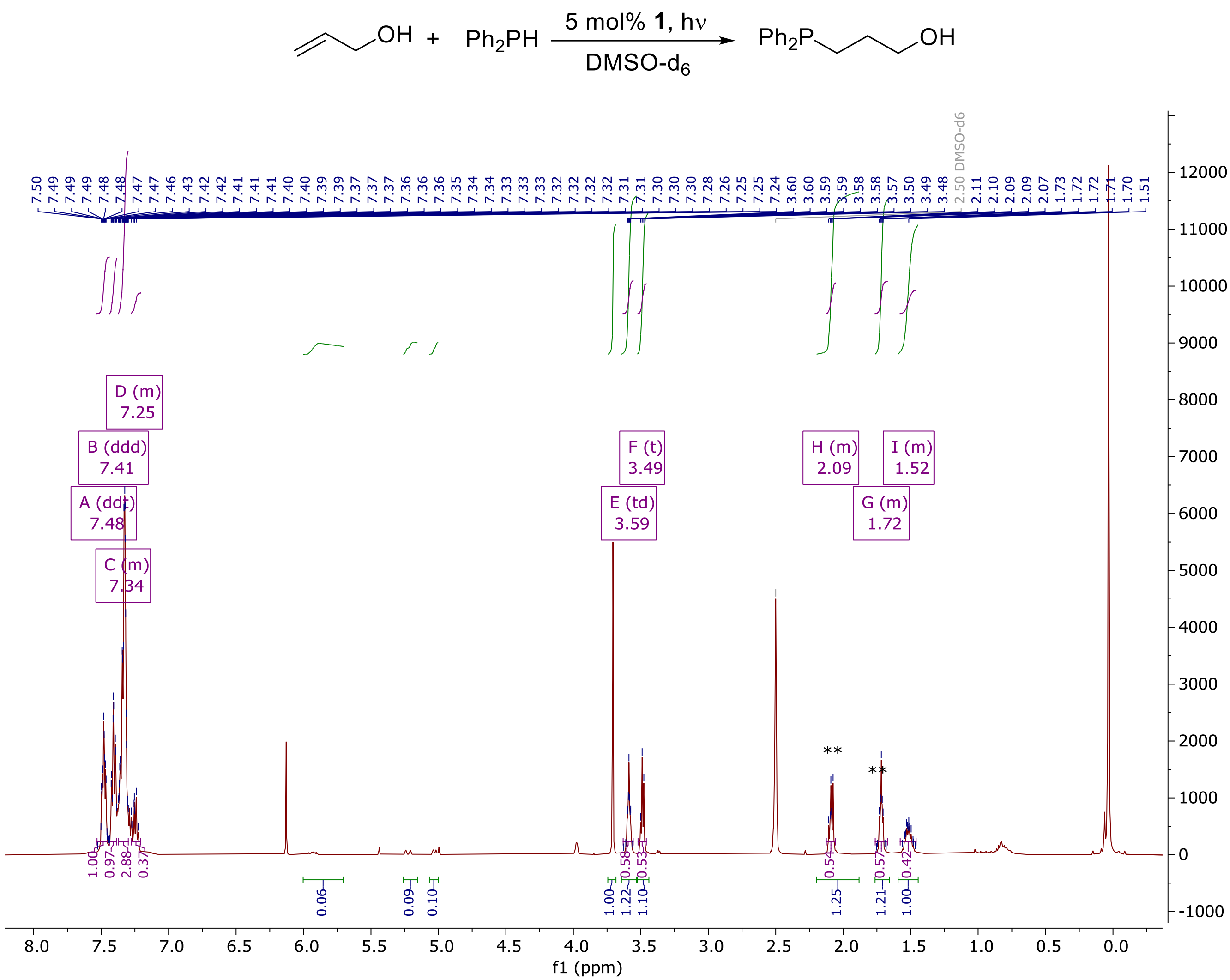

Figure S136: Hydrophosphination of 0.5 mmol allyl alcohol with equimolar of Ph_2PH in DMSO-d_6 using 5 mol% **1** under UV 360 nm irradiation – 72 h –^{1}H NMR

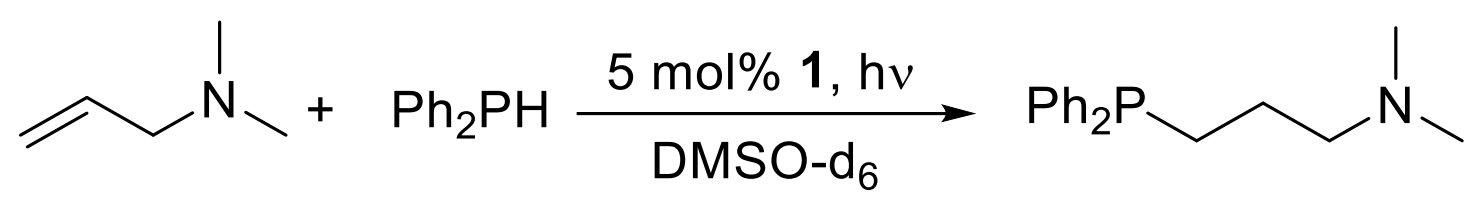

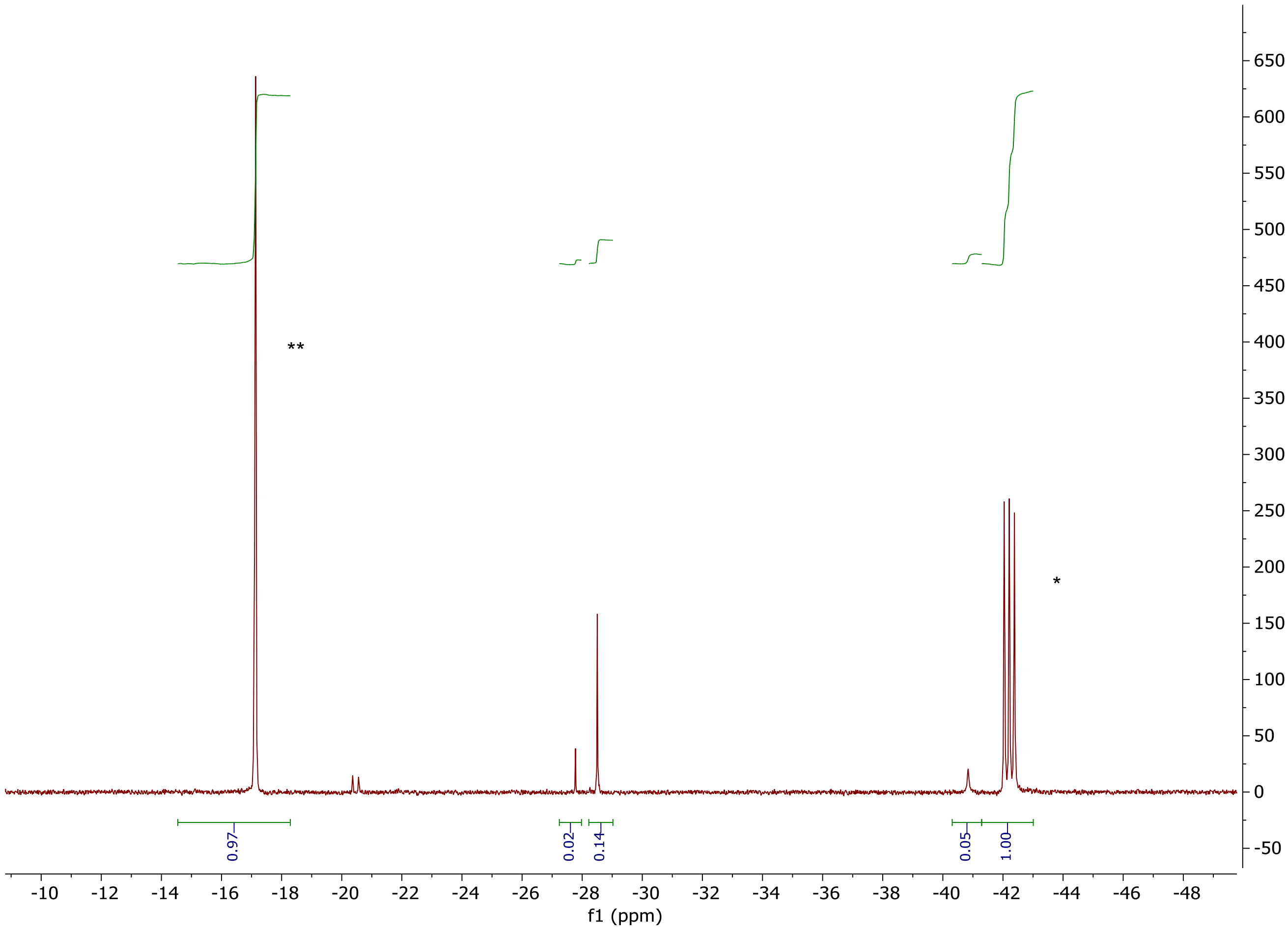

Figure S137: Hydrophosphination of 0.5 mmol N,N-dimethyl-2-propene-1-amine with equimolar of Ph_2PH in DMSO-d_6 using 5 mol% **1** under UV 360 nm irradiation – 40h – $^{31}P\{^1H\}$ NMR

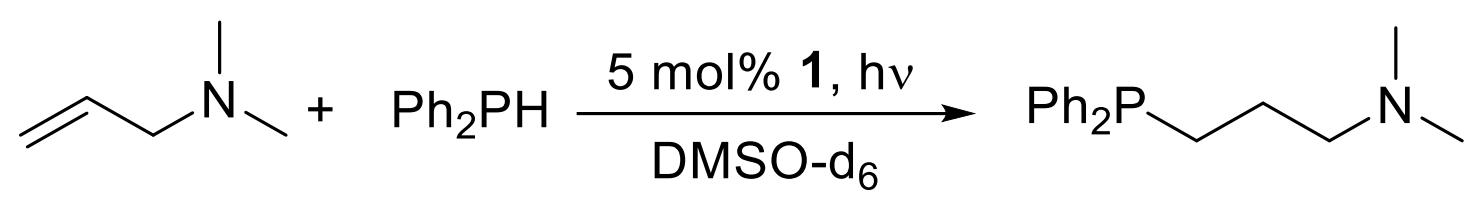

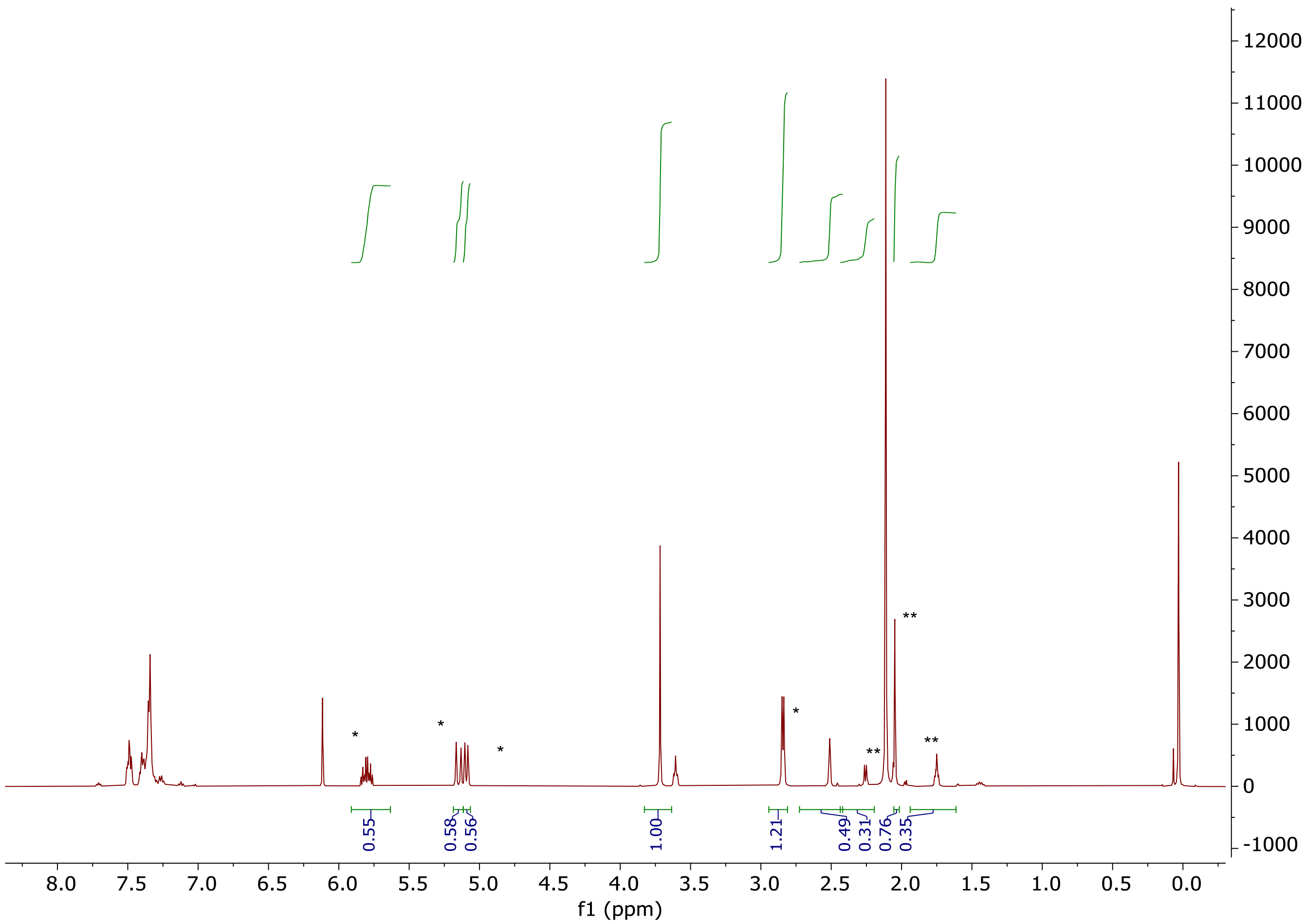

Figure S138: Hydrophosphination of 0.5 mmol N,N-dimethyl-2-propene-1-amine with equimolar of Ph_2PH in DMSO-d_6 using 5 mol% **1** under UV 360 nm irradiation – 40h –^{1}H NMR

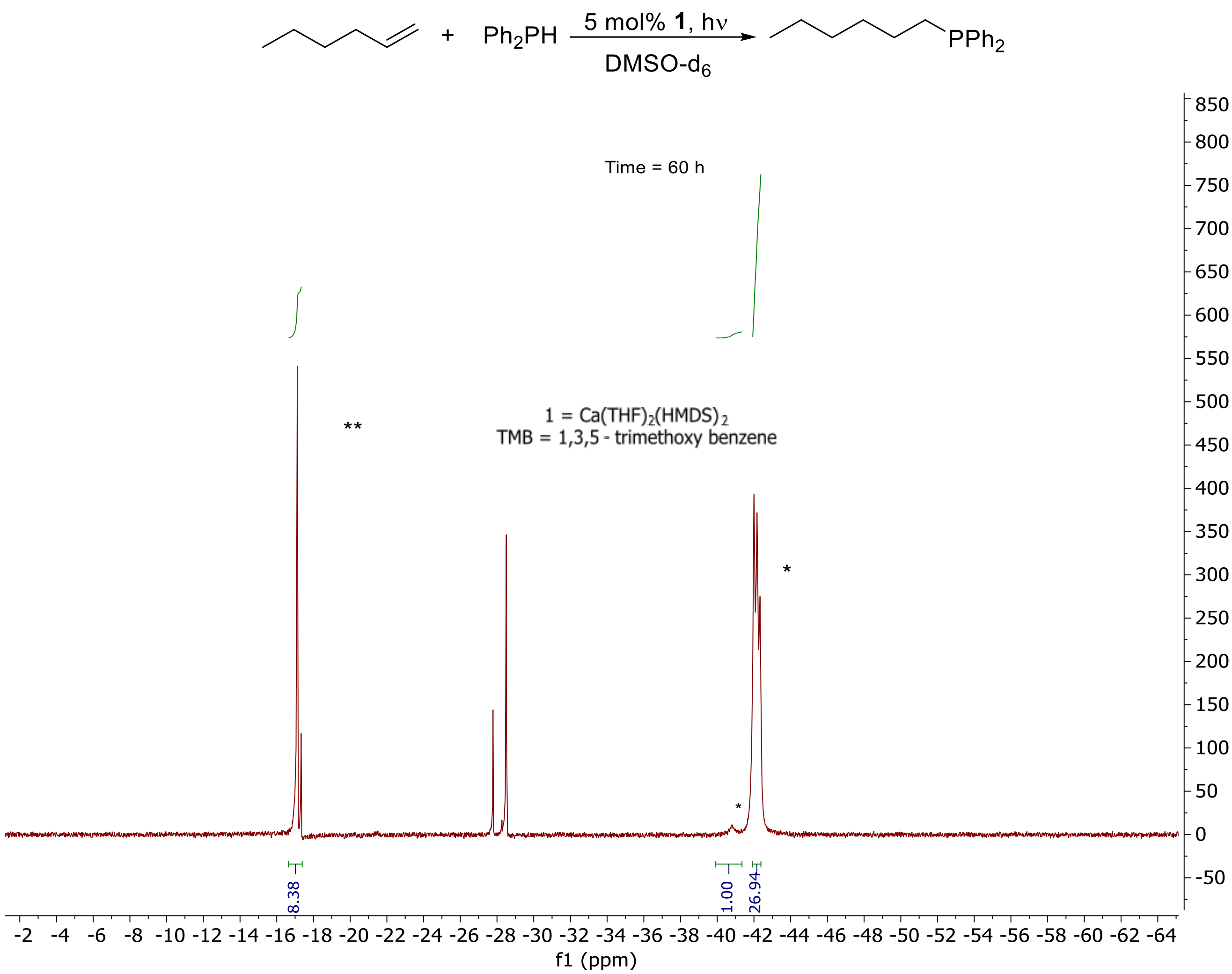

Figure S139: Hydrophosphination of 0.5 mmol 1-hexene with equimolar of Ph_2PH in DMSO-d_6 using 5 mol% **1** under UV 360 nm irradiation – 60h –$^{31}P\{^1H\}$ NMR

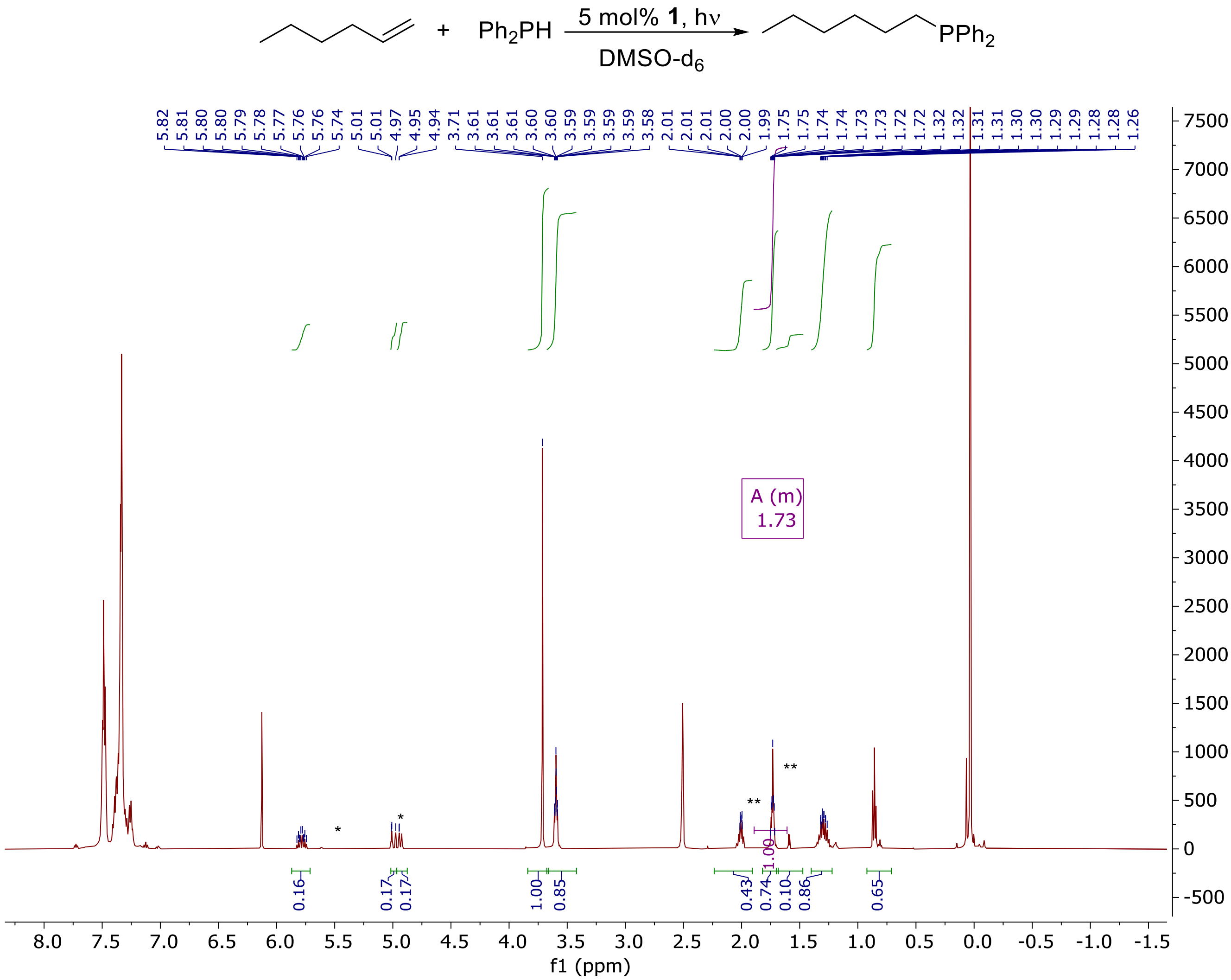

Figure S140: Hydrophosphination of 0.5 mmol 1-hexene with equimolar of Ph_2PH in DMSO-d_6 using 5 mol% **1** under UV 360 nm irradiation – 60h –^{1}H NMR

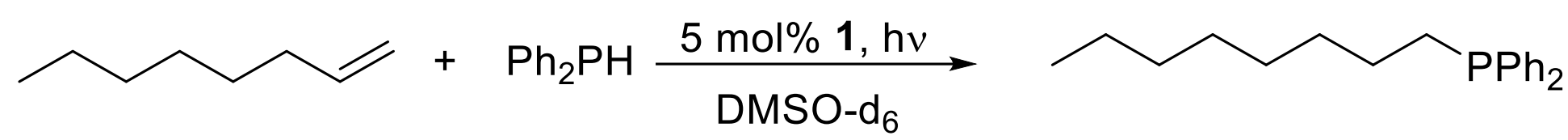

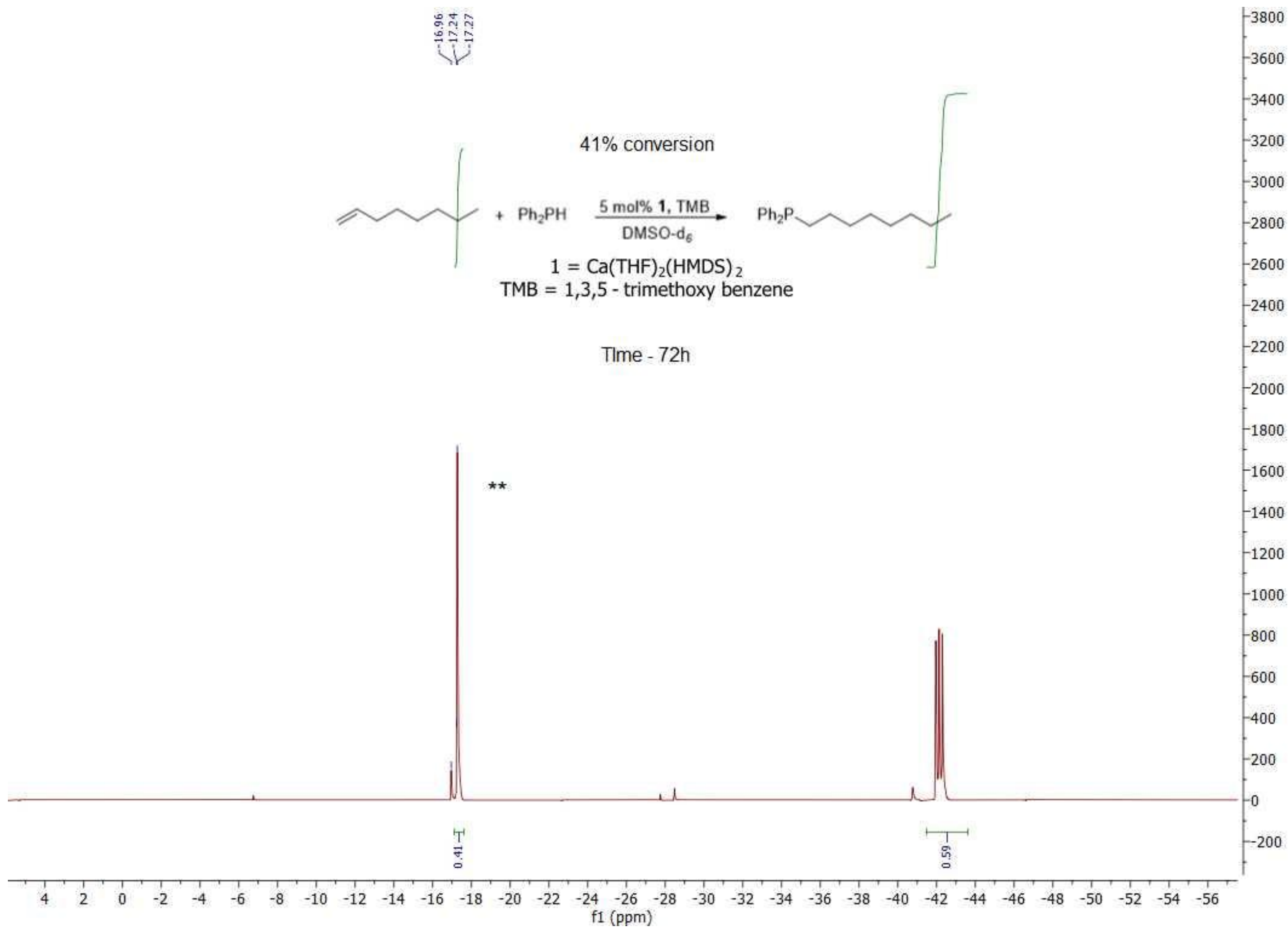

Figure S141: Hydrophosphination of 0.5 mmol 1-octene with equimolar of Ph_2PH in DMSO-d_6 using 5 mol% **1** under UV 360 nm irradiation – 72h –^{1}H NMR

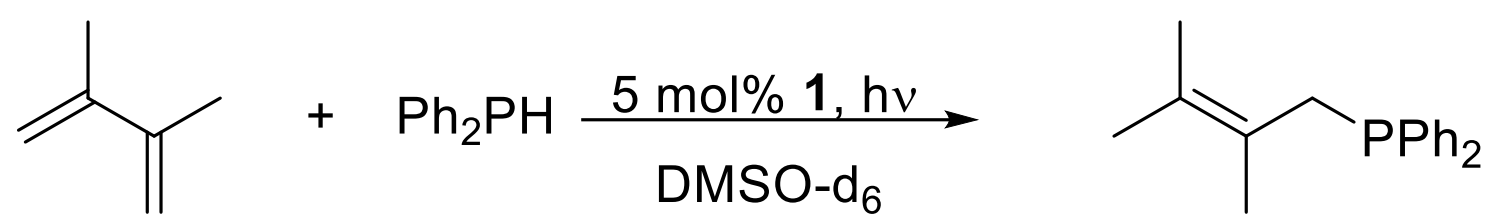

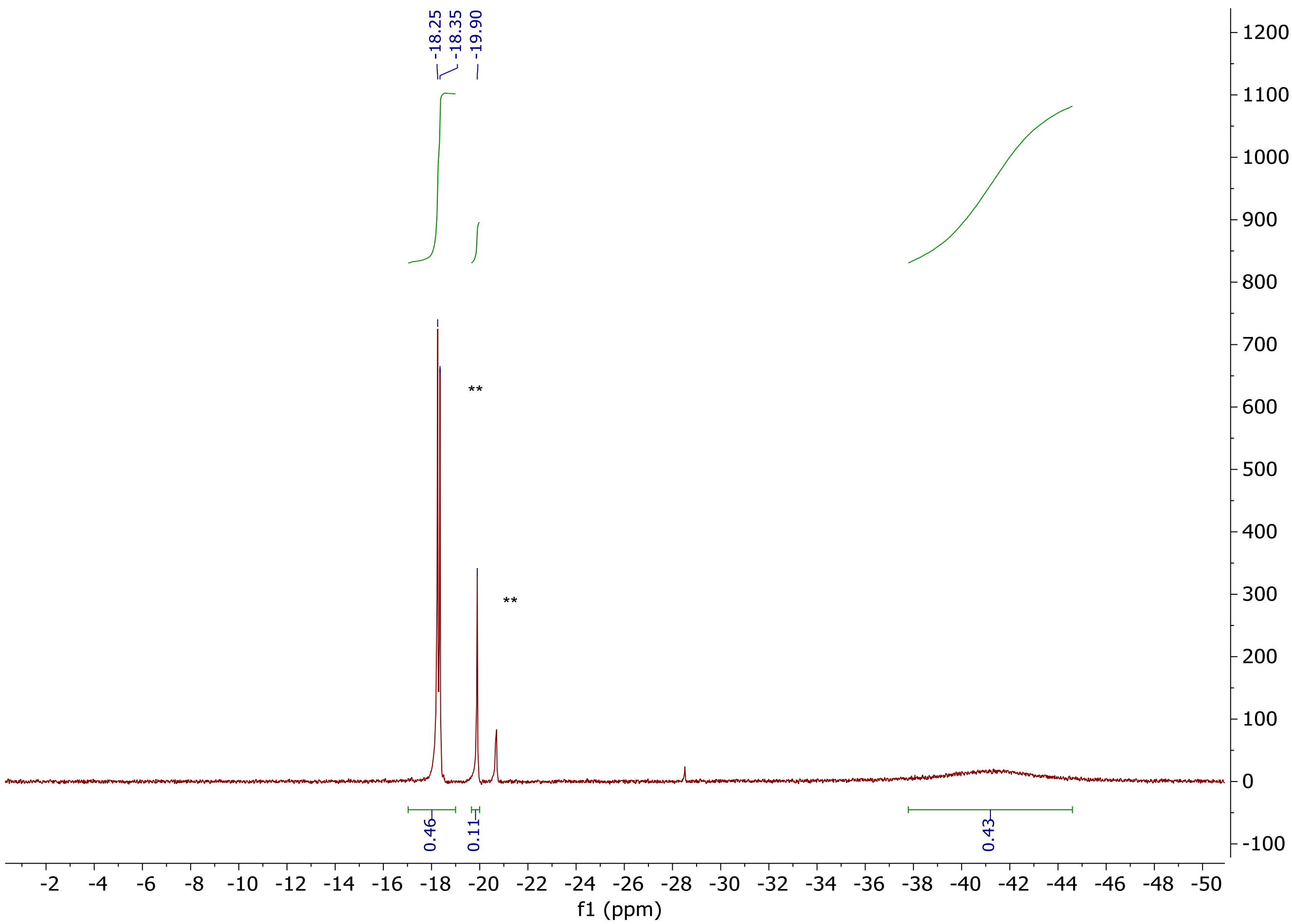

Figure S142: Hydrophosphination of 0.5 mmol 2,3-dimethyl-1,3-butadiene with equimolar of Ph_2PH in DMSO-d_6 using 5 mol% **1** under UV 360 nm irradiation – 24h –$^{31}P\{^1H\}$ NMR

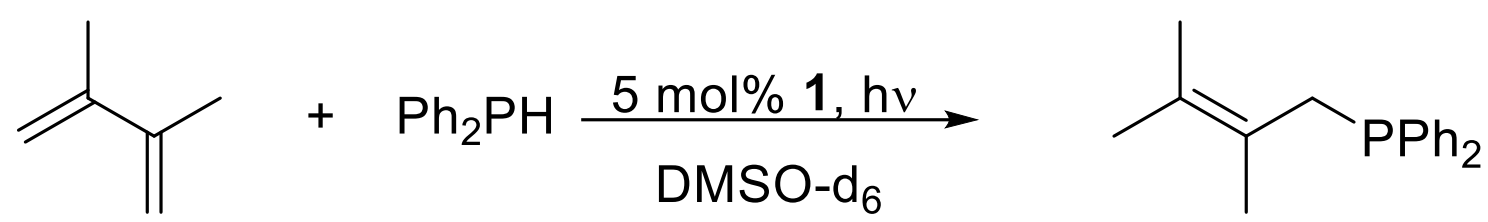

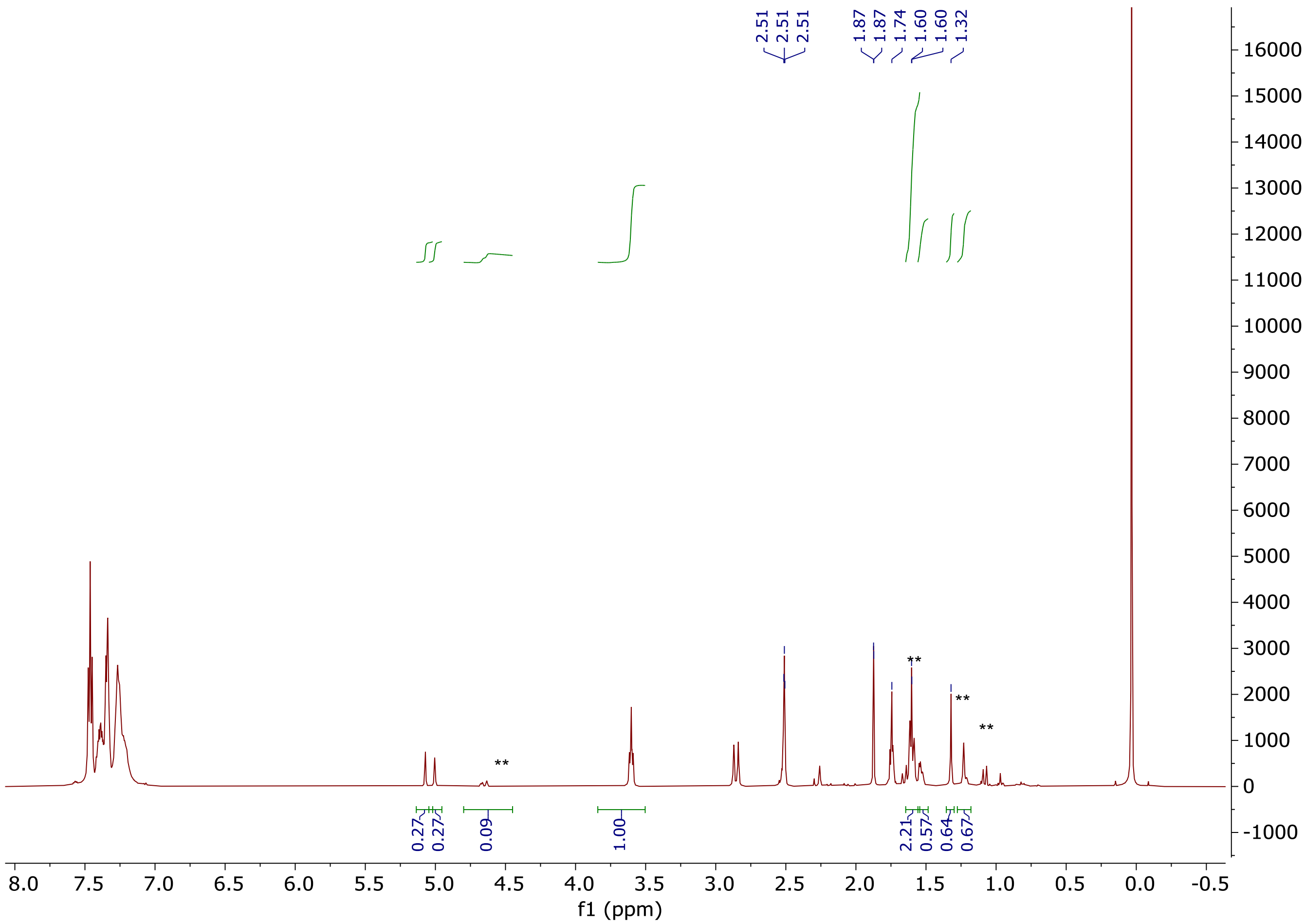

Figure S143: Hydrophosphination of 0.5 mmol 2,3-dimethyl-1,3-butadiene with equimolar of Ph_2PH in DMSO-d_6 using 5 mol% **1** under UV 360 nm irradiation – 24h –^{1}H NMR

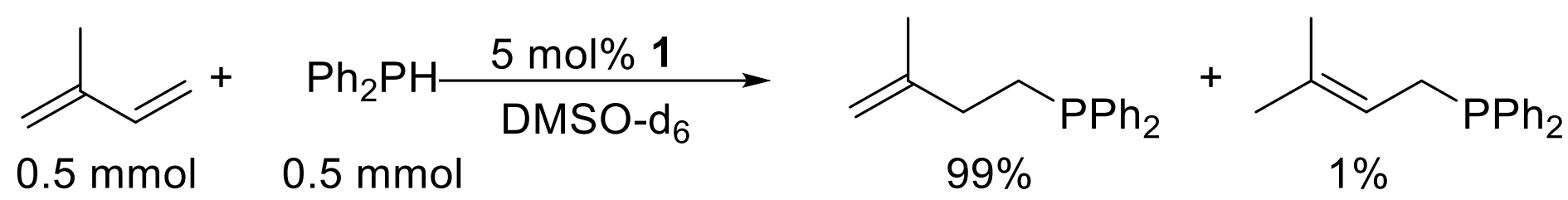

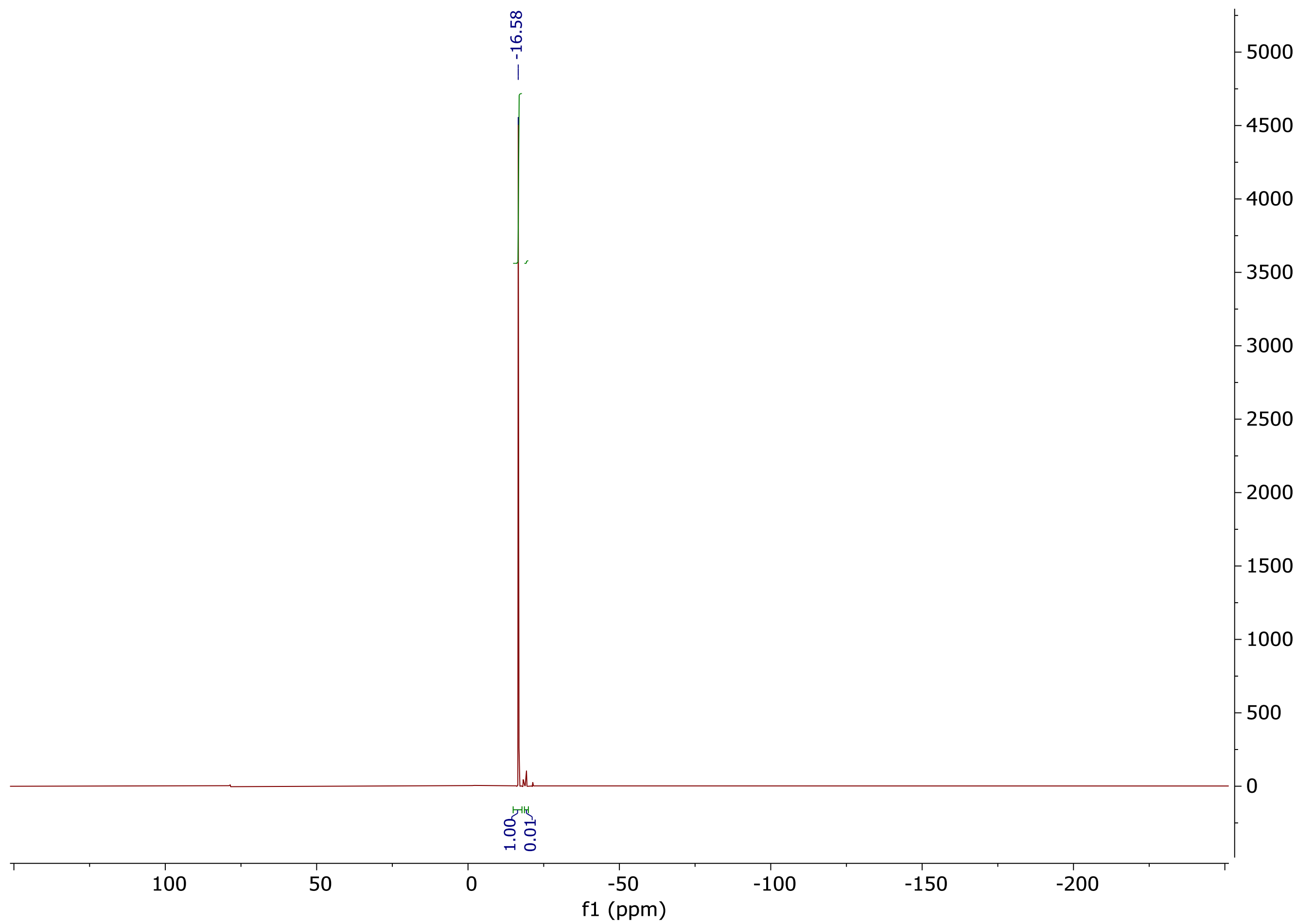

Figure S144: Hydrophosphination of 0.5 mmol isoprene with equimolar of Ph_2PH in DMSO-d_6 using 5 mol% **1** – 0 h –^{1}H NMR

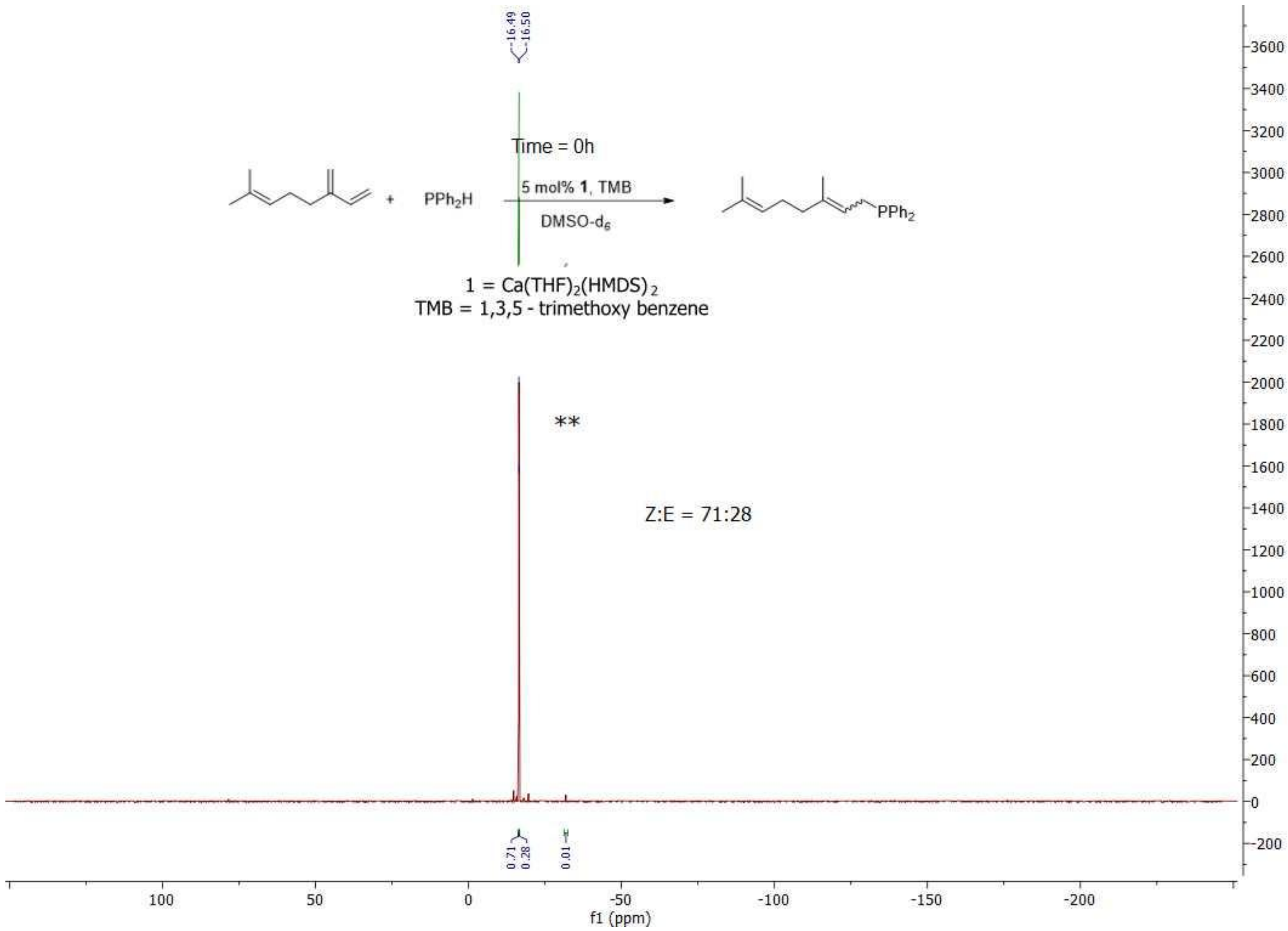

Figure S145: Hydrophosphination of 0.5 mmol myrcene with equimolar of Ph_2PH in DMSO-d_6 using 5 mol% **1** – 0 h –^{1}H NMR

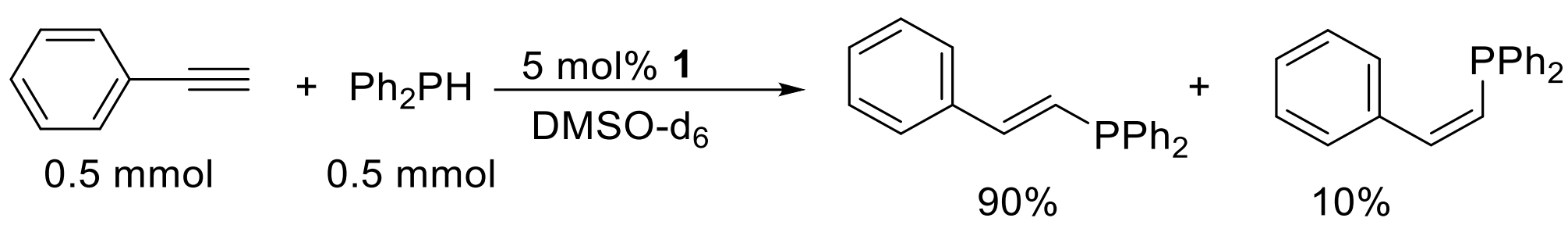

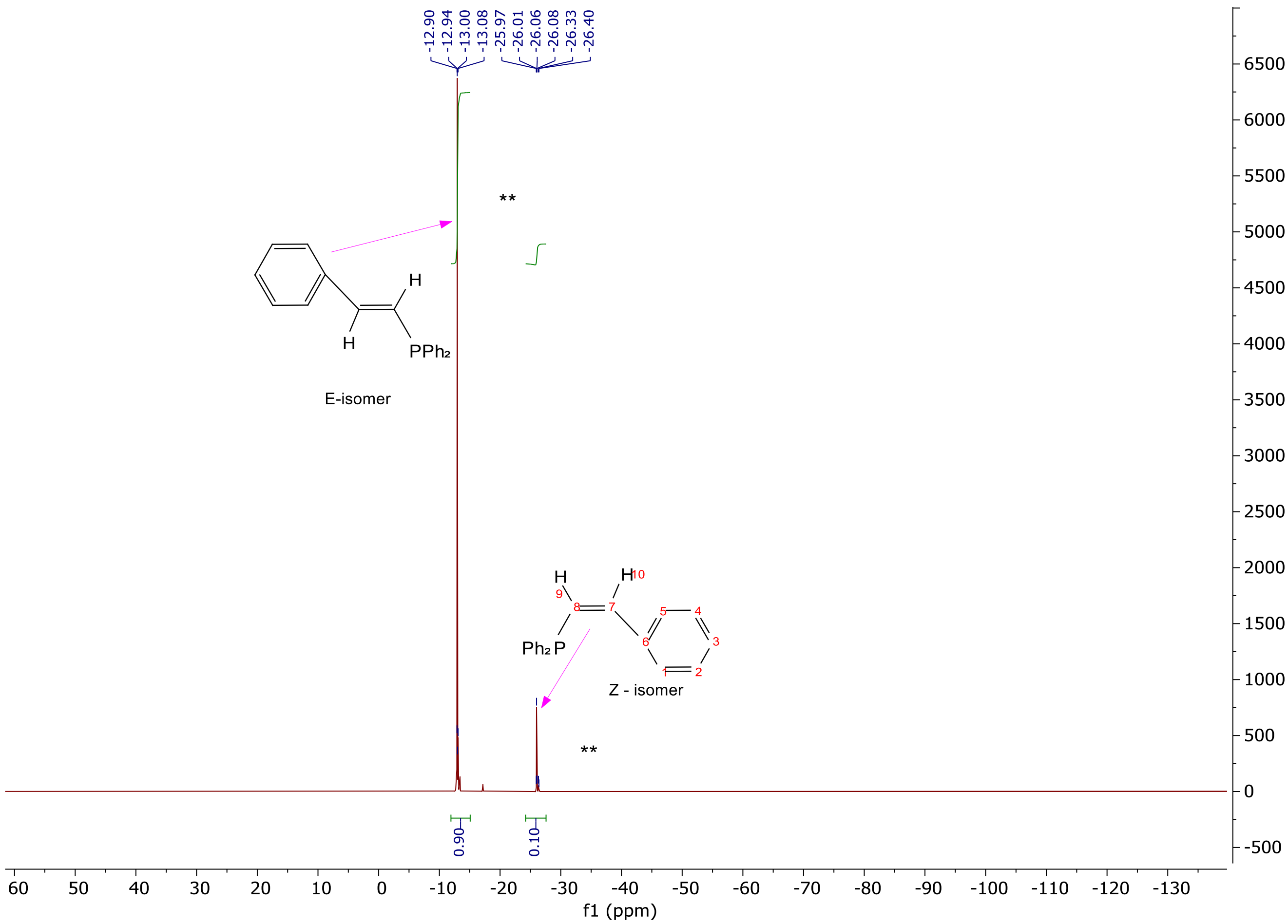

Figure S146: Hydrophosphination of 0.5 mmol phenyl acetylene with equimolar of Ph_2PH in DMSO-d_6 using 5 mol% **1** – 0 h –$^{31}P\{^1H\}$ NMR

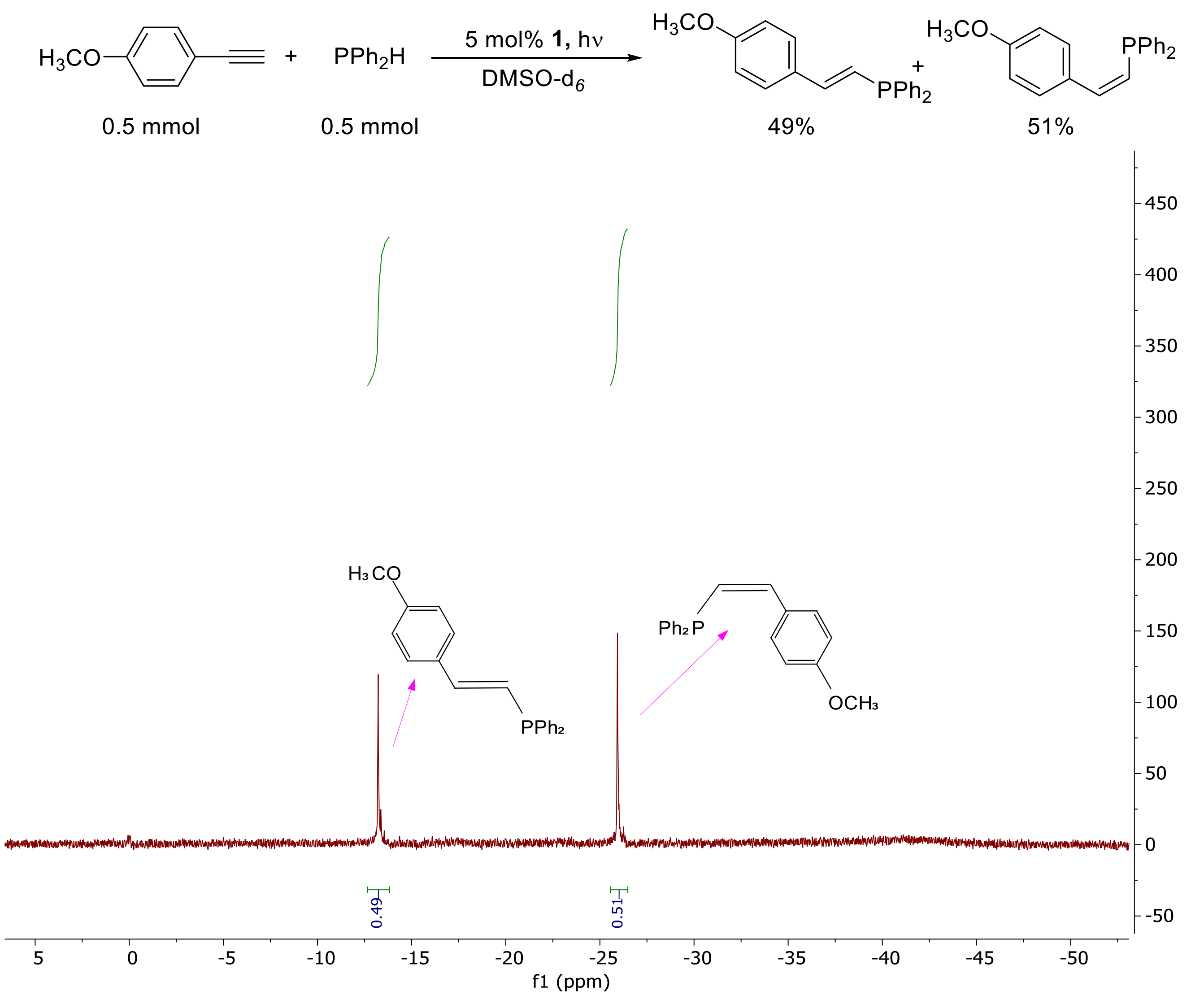

Figure S147: Hydrophosphination of 0.5 mmol 4-ethynyl anisole with equimolar of Ph_2PH in DMSO-d_6 using 5 mol% **1** under UV 360 nm irradiation – 2 h –$^{31}P\{^1H\}$ NMR

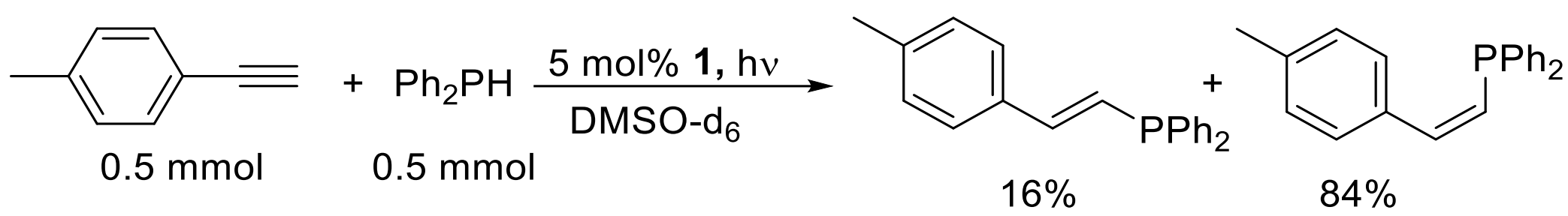

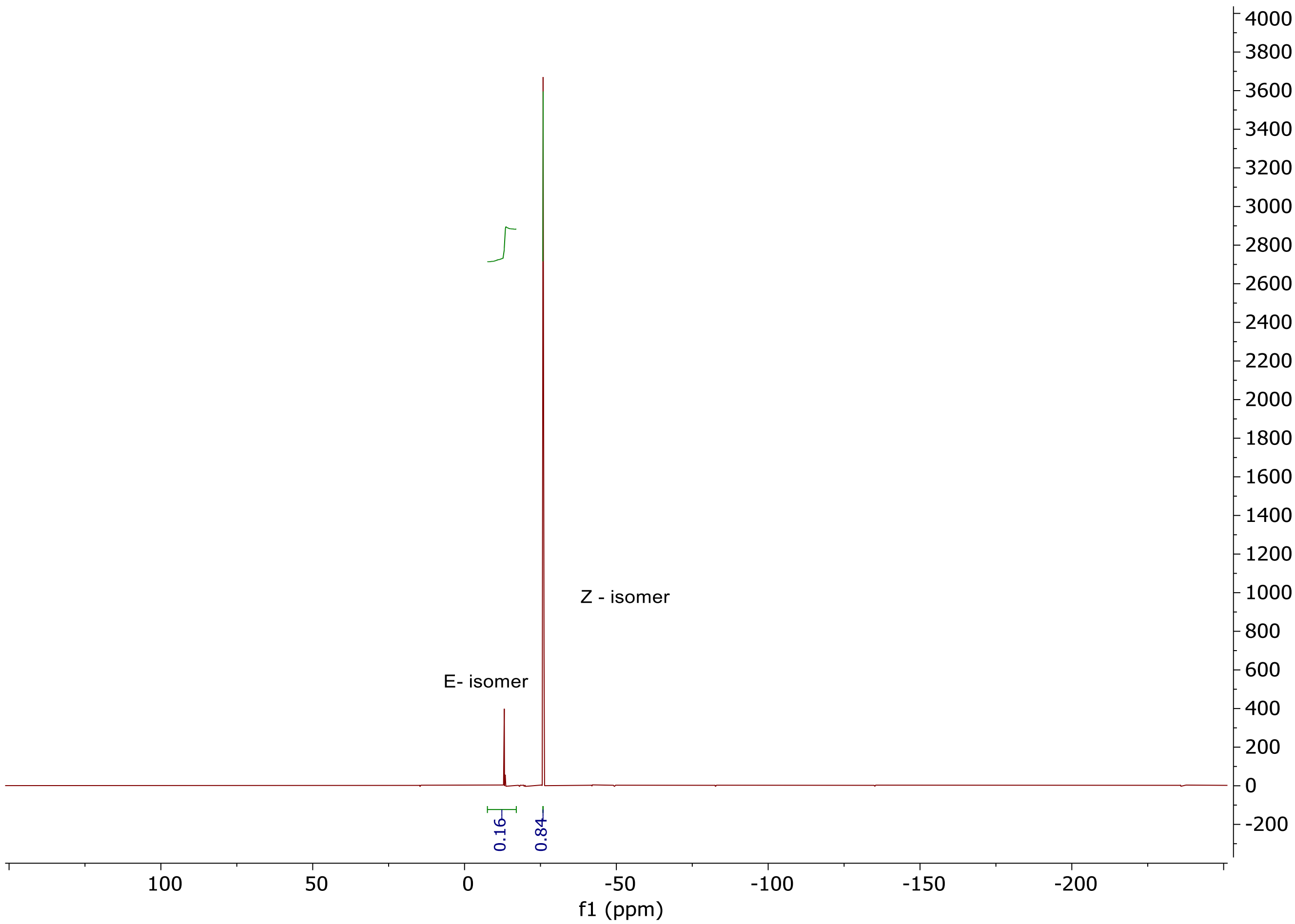

Figure S148: Hydrophosphination of 0.5 mmol 4-ethynyl toluene with equimolar of Ph_2PH in DMSO-d_6 using 5 mol% **1** – 1 h –$^{31}P\{^1H\}$ NMR.

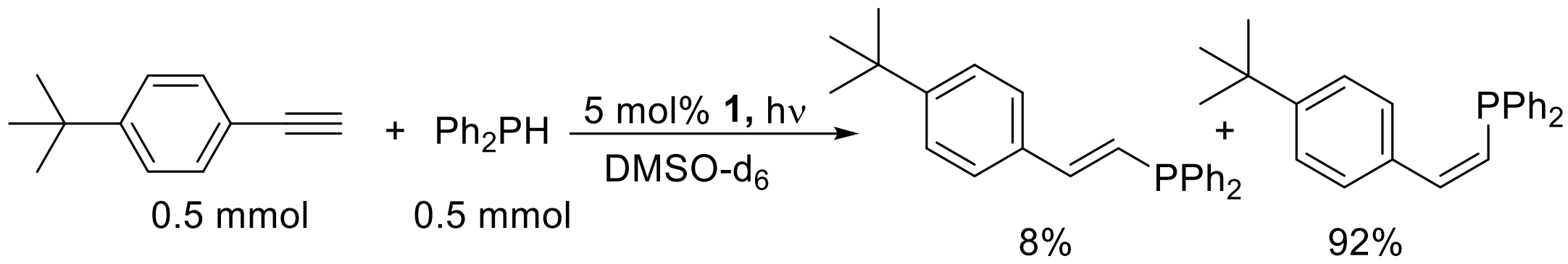

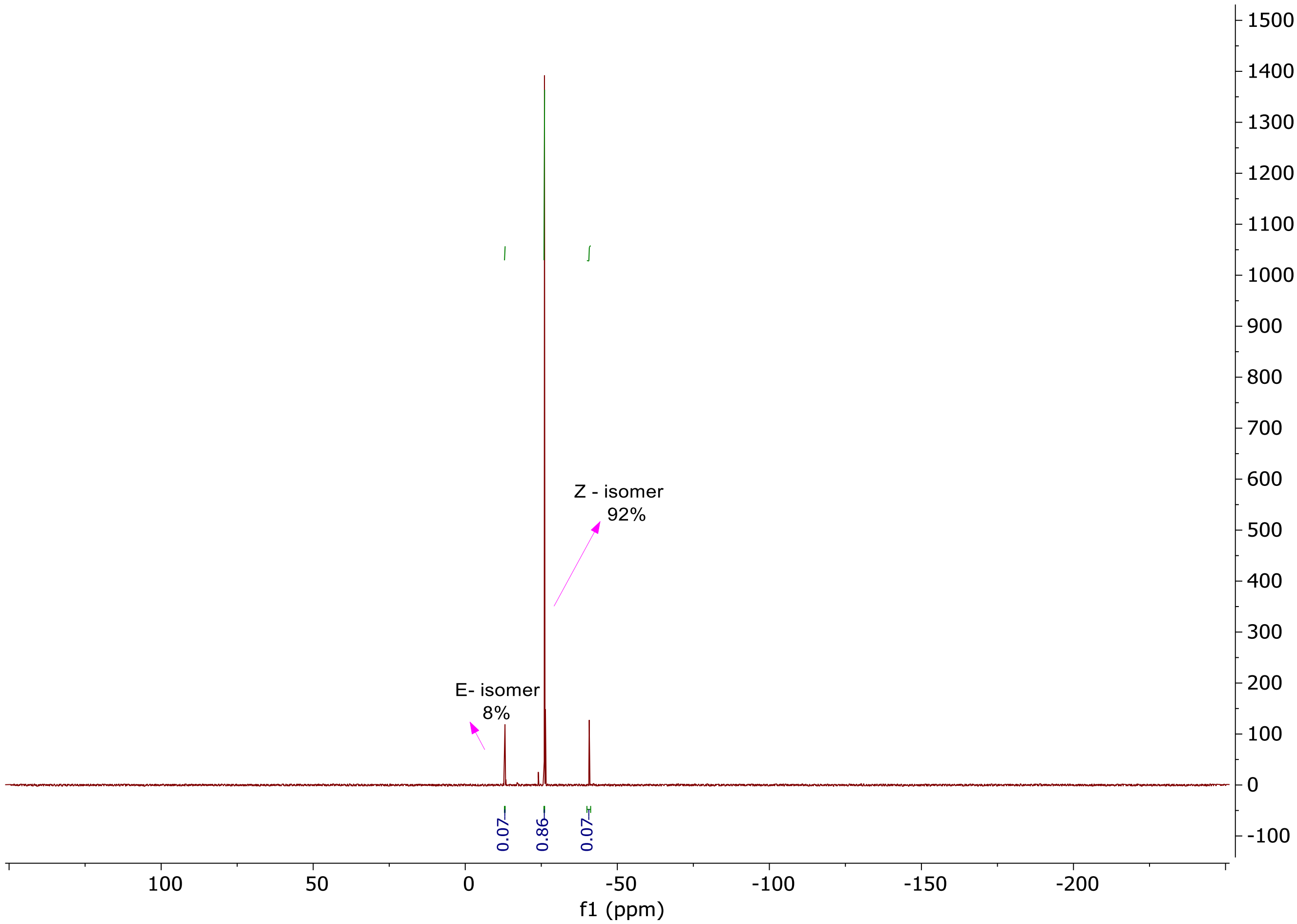

Figure S149: Hydrophosphination of 0.5 mmol 4-ethynyl tert-butyl benzene with equimolar Ph_2PH in DMSO-d_6 using 5 mol% **1** under UV 360 nm irradiation – 5 h – $^{31}P\{^1H\}$ NMR

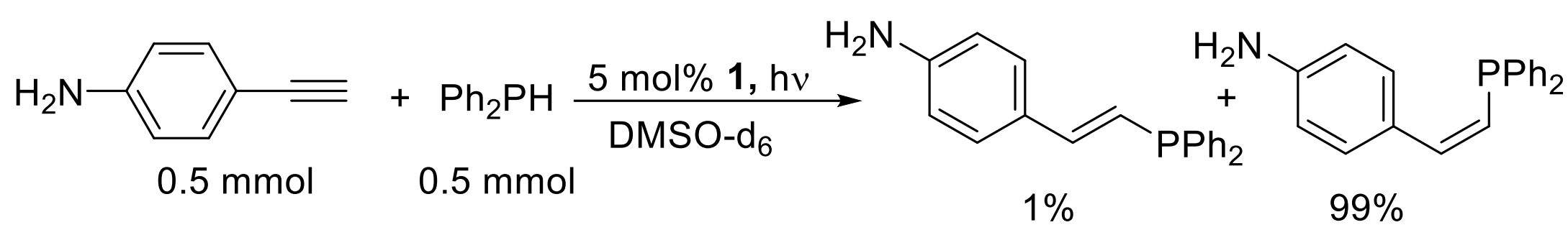

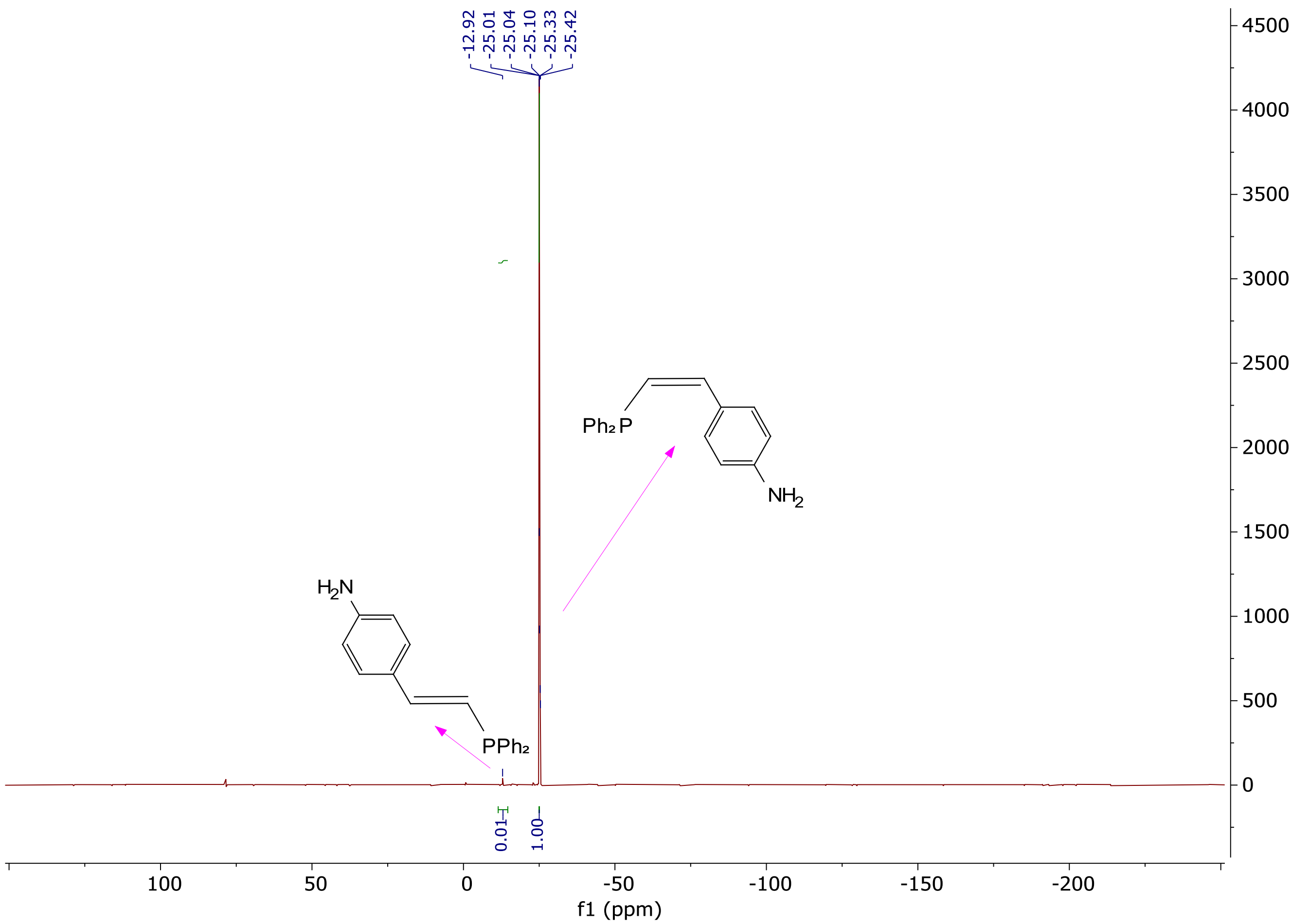

Figure S150: Hydrophosphination of 0.5 mmol 4-ethynyl aniline with equimolar of Ph_2PH in DMSO-d_6 using 5 mol% **1** – 0 h – $^{31}P\{^1H\}$ NMR

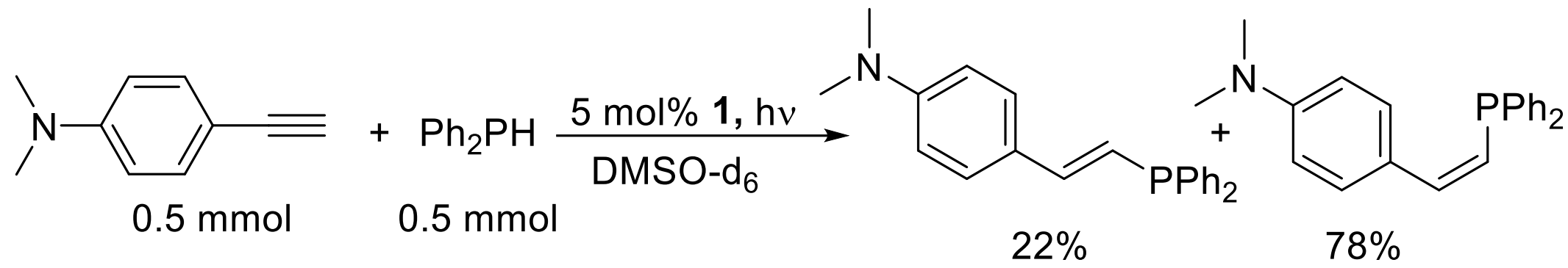

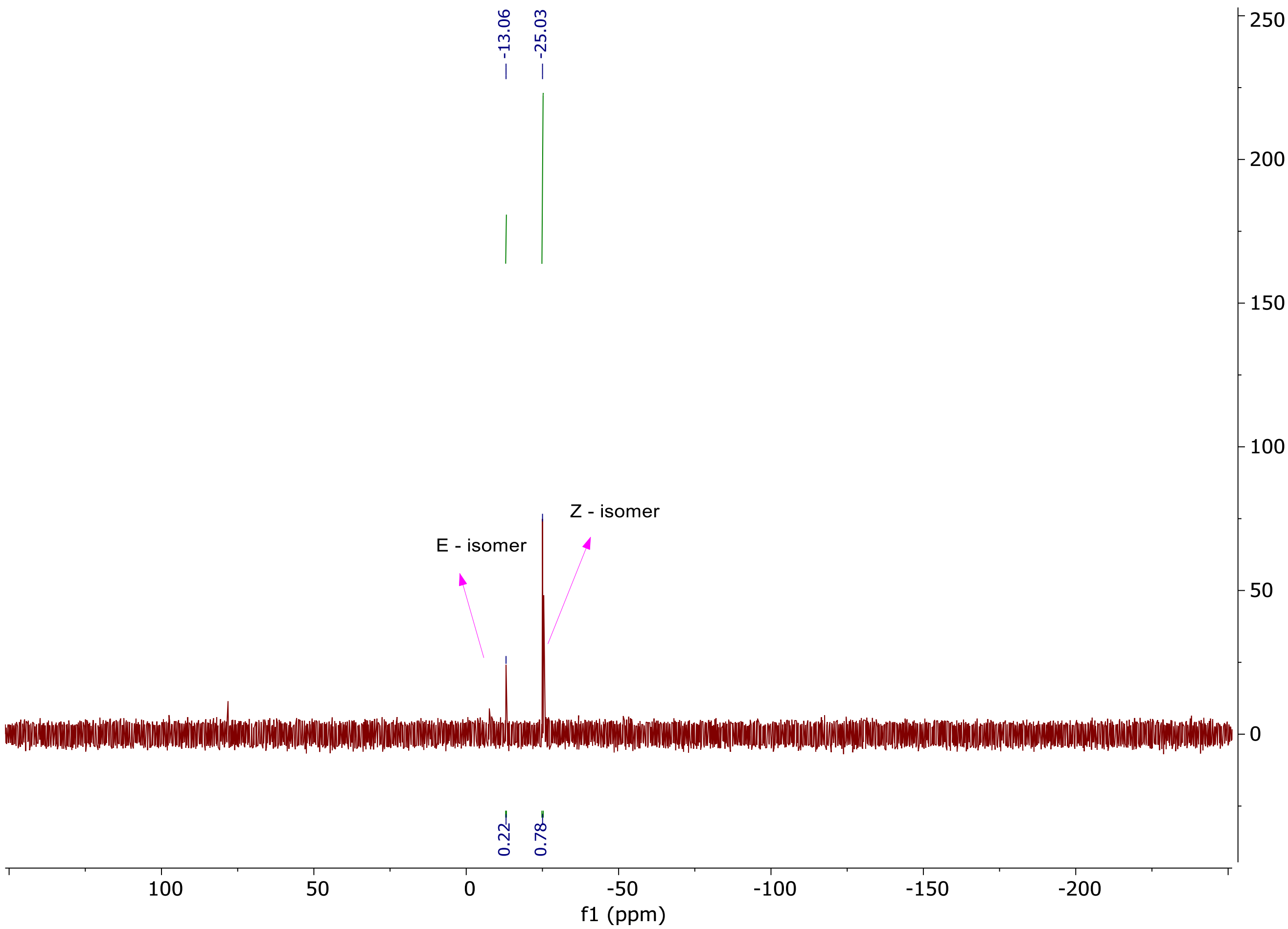

Figure S151: Hydrophosphination of 0.5 mmol 4-ethynyl – N,N – dimethyl aniline with equimolar of Ph_2PH in DMSO-d_6 using 5 mol% **1** – 0 h – $^{31}P\{^1H\}$ NMR

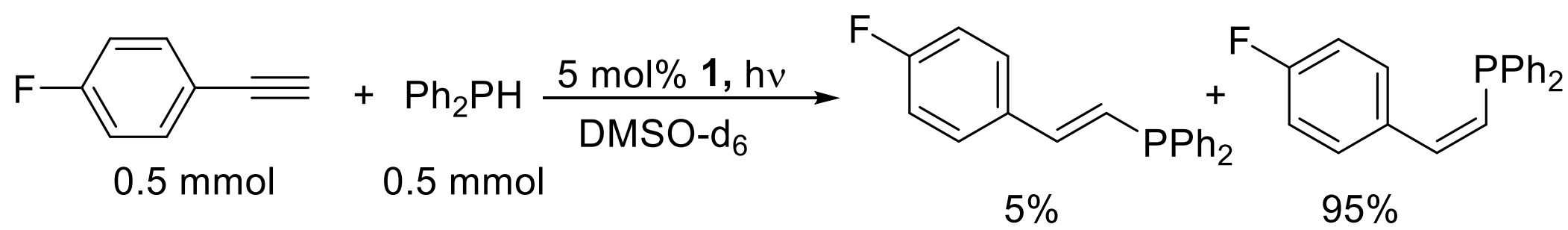

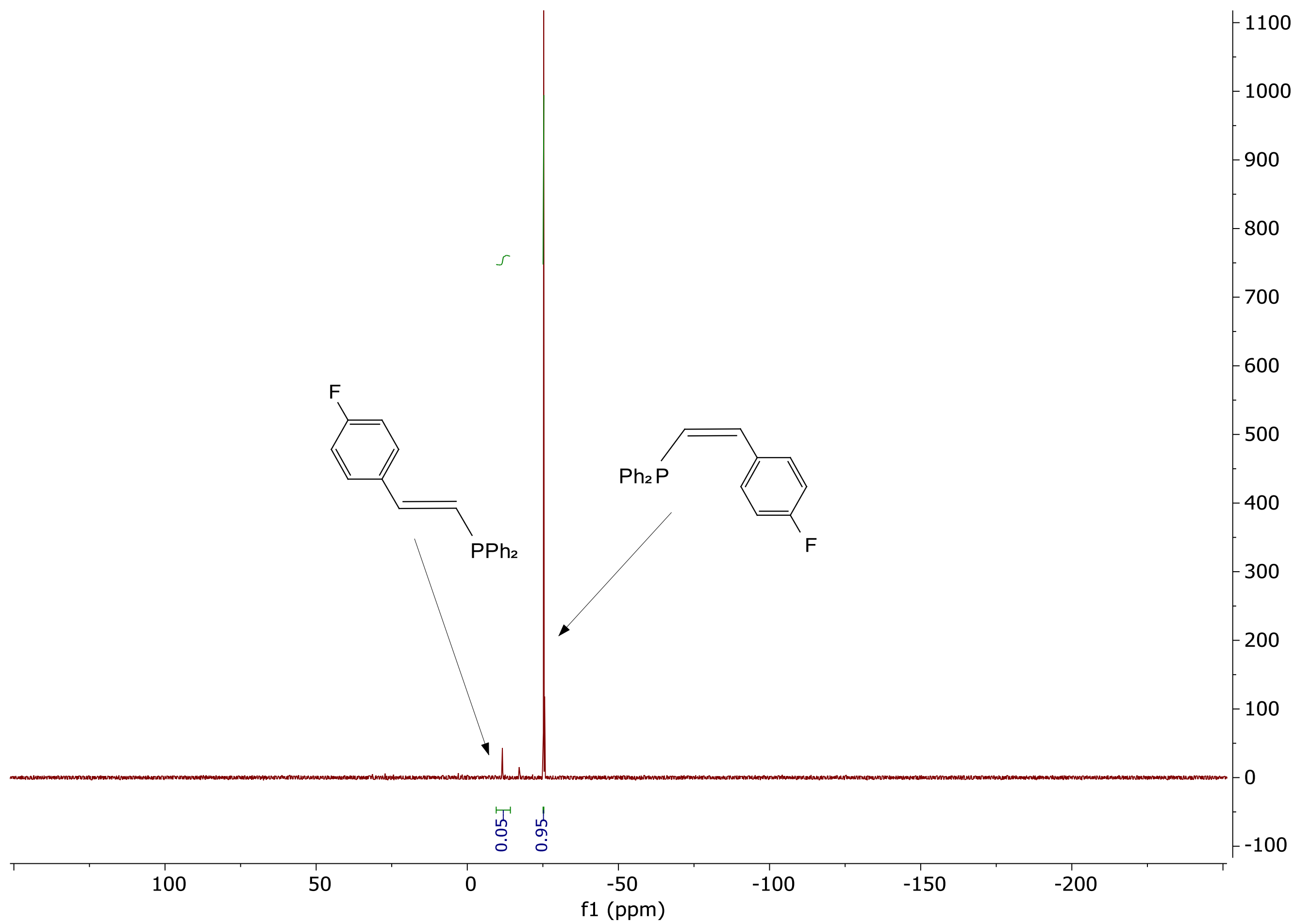

Figure S152: Hydrophosphination of 0.5 mmol 4-ethynyl-fluorobenzene with equimolar of Ph_2PH in DMSO-d_6 using 5 mol% **1** – 0 h – $^{31}P\{^1H\}$ NMR

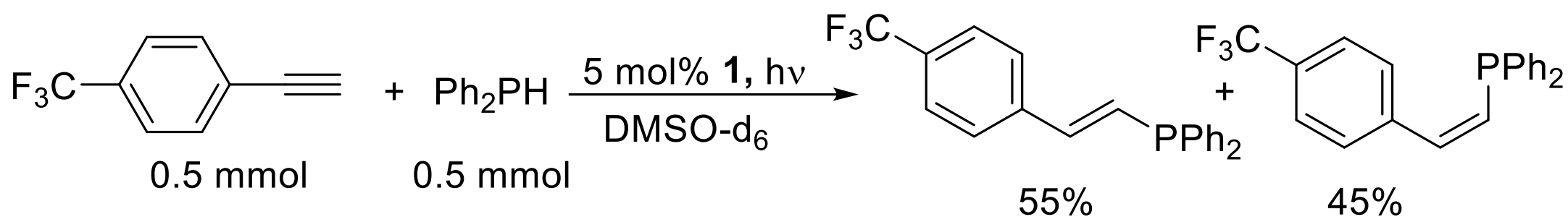

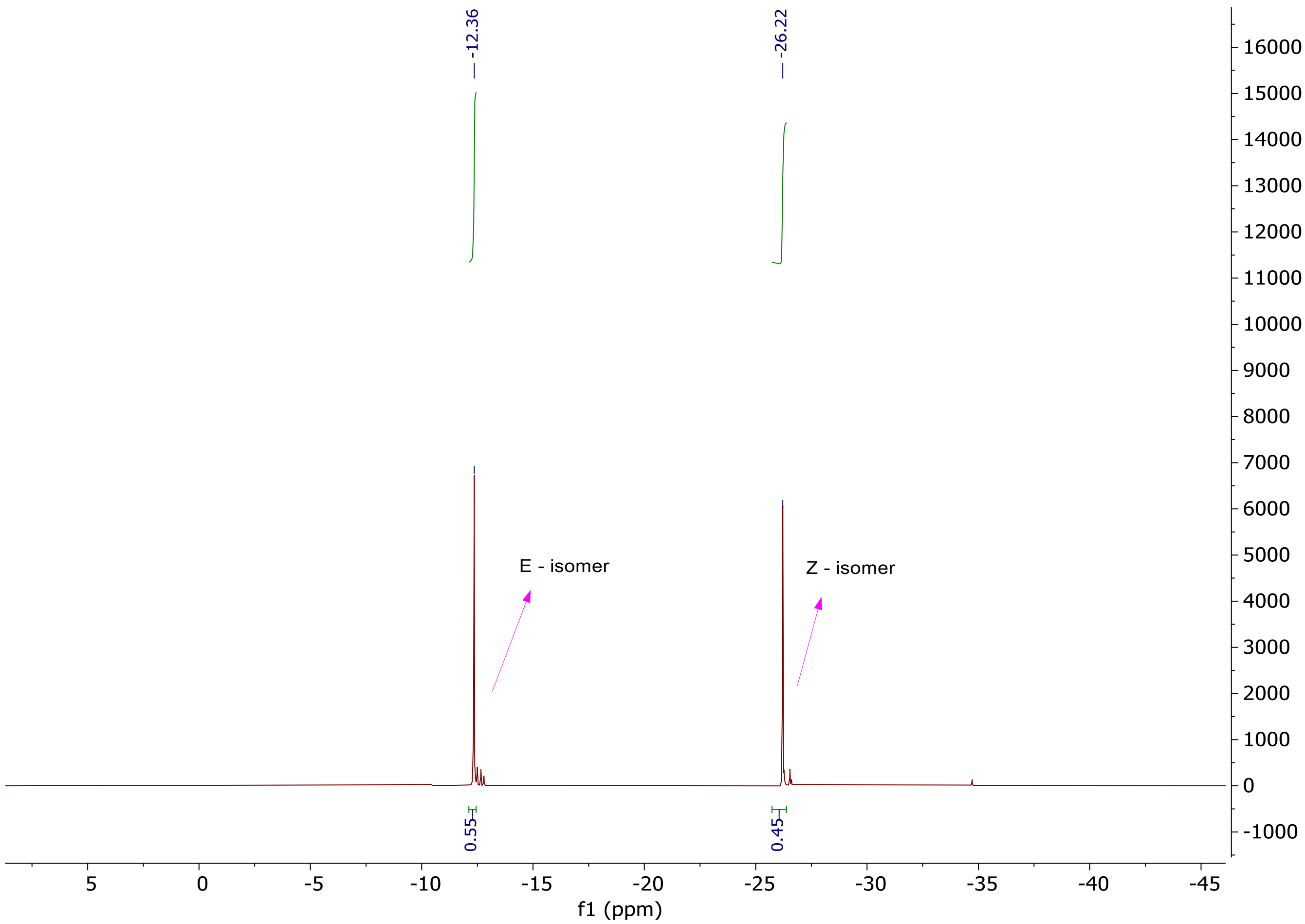

Figure S153: Hydrophosphination of 0.5 mmol 4-ethynyl-trifluoromethyl benzene with equimolar of Ph_2PH in DMSO-d_6 using 5 mol% **1** – 0 h –$^{31}P\{^1H\}$ NMR

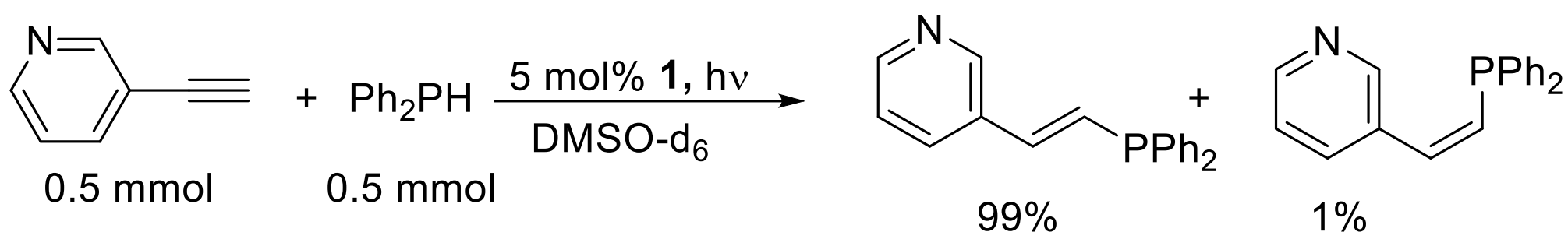

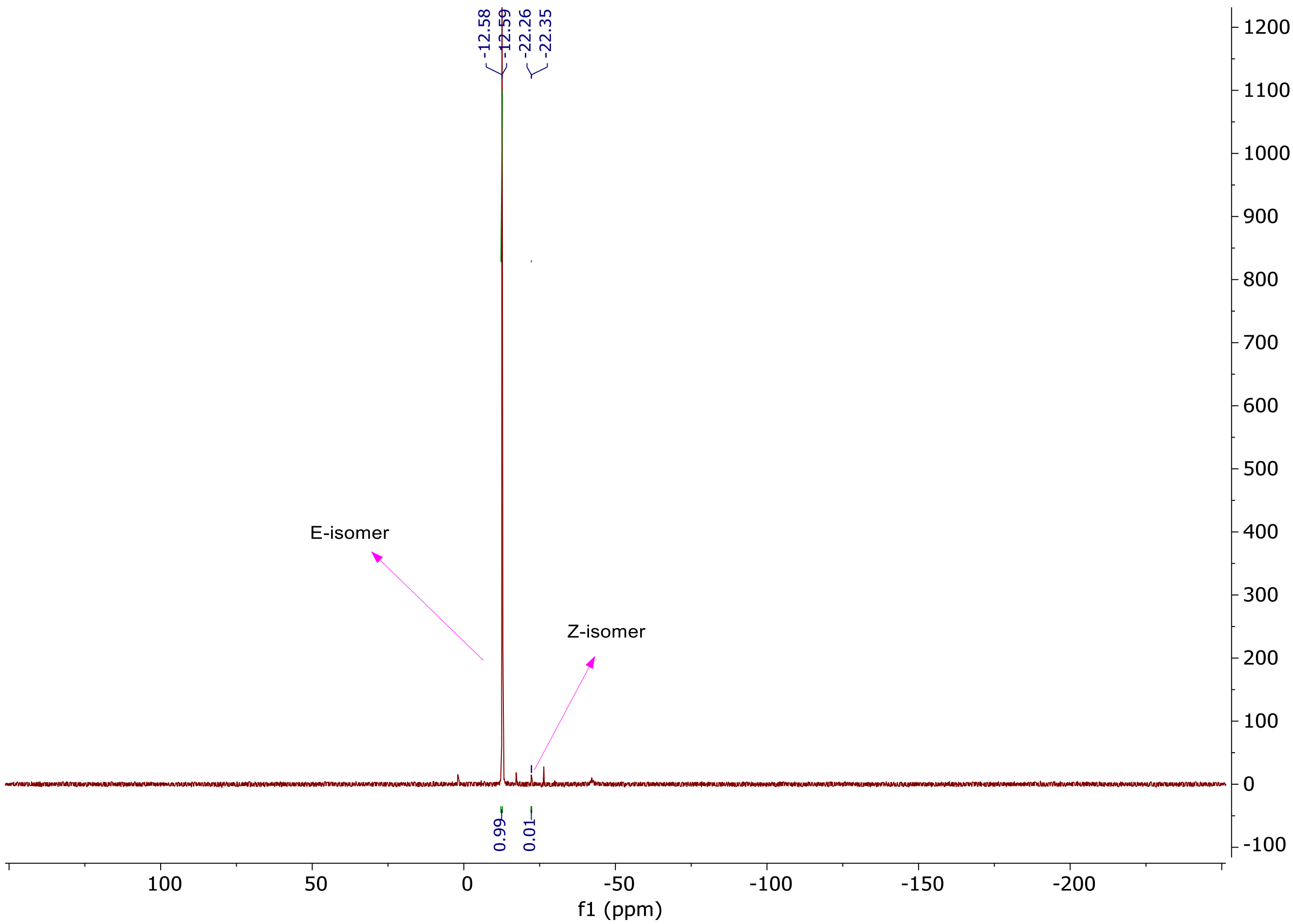

Figure S154: Hydrophosphination of 0.5 mmol 3-ethynyl pyridine with equimolar of Ph_2PH in DMSO-d_6 using 5 mol% **1** – 0 h –$^{31}P\{^1H\}$ NMR

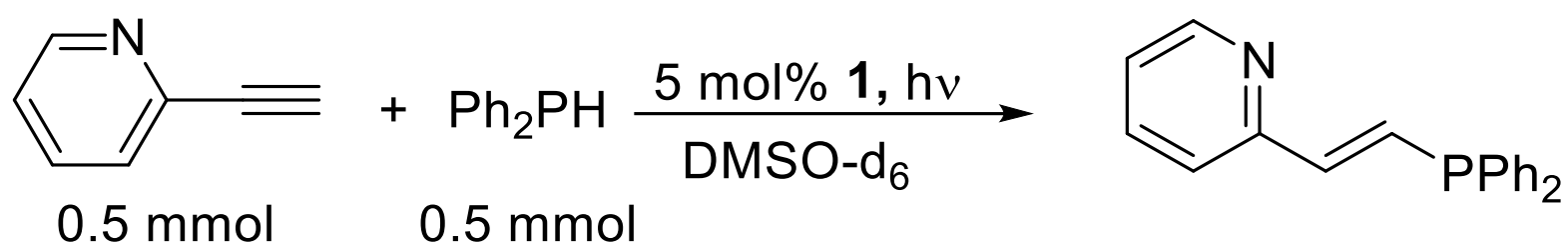

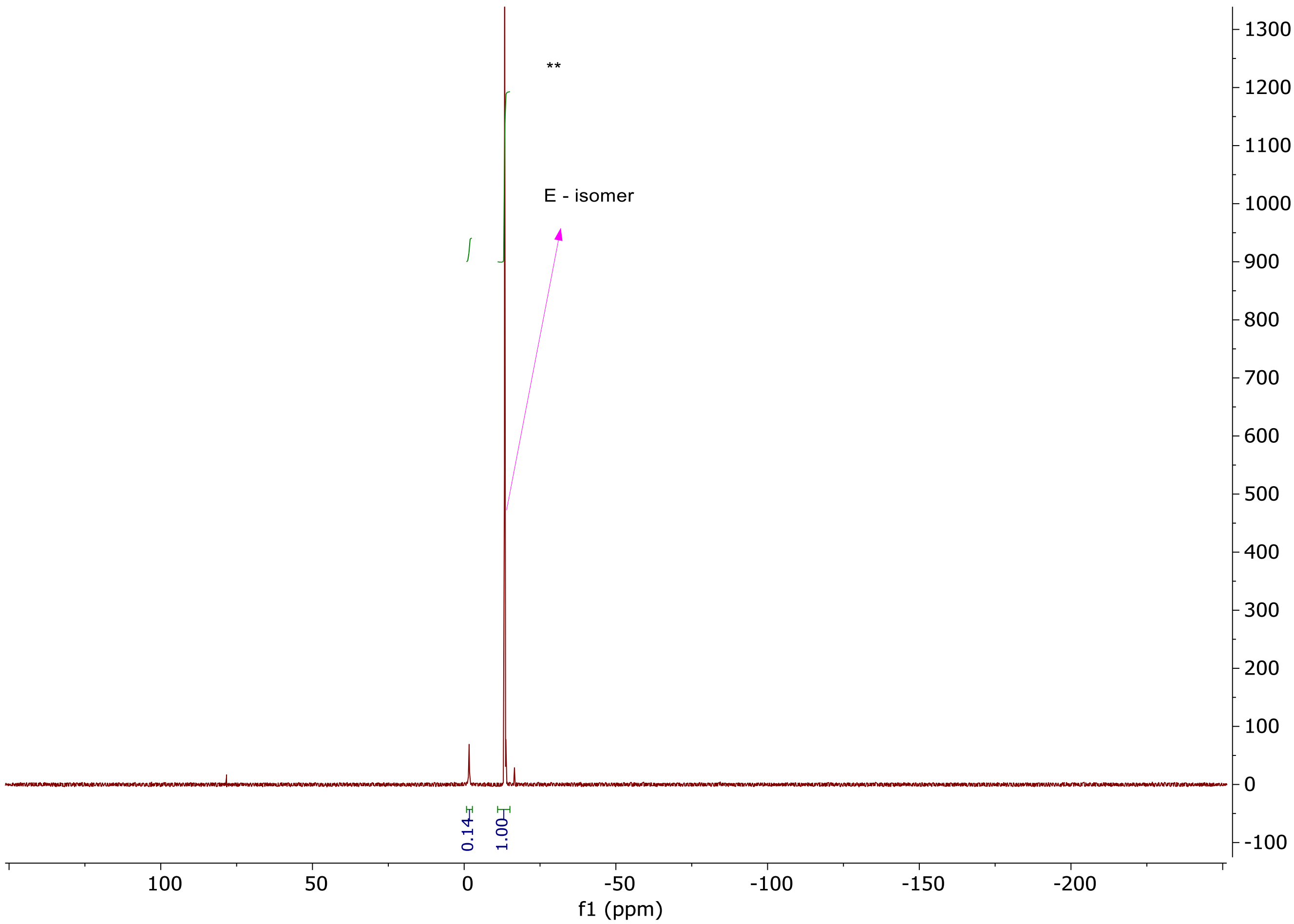

Figure S155: Hydrophosphination of 0.5 mmol 2-ethynyl pyridine with equimolar of Ph_2PH in DMSO-d_6 using 5 mol% **1** – 0 h – $^{31}P\{^1H\}$ NMR

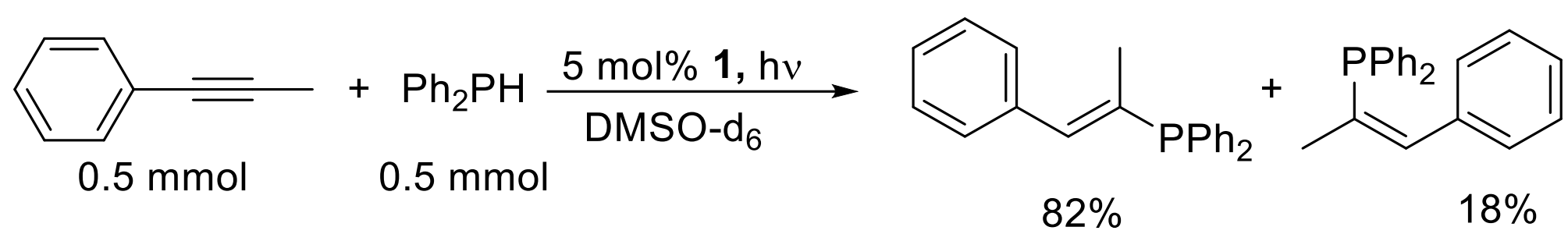

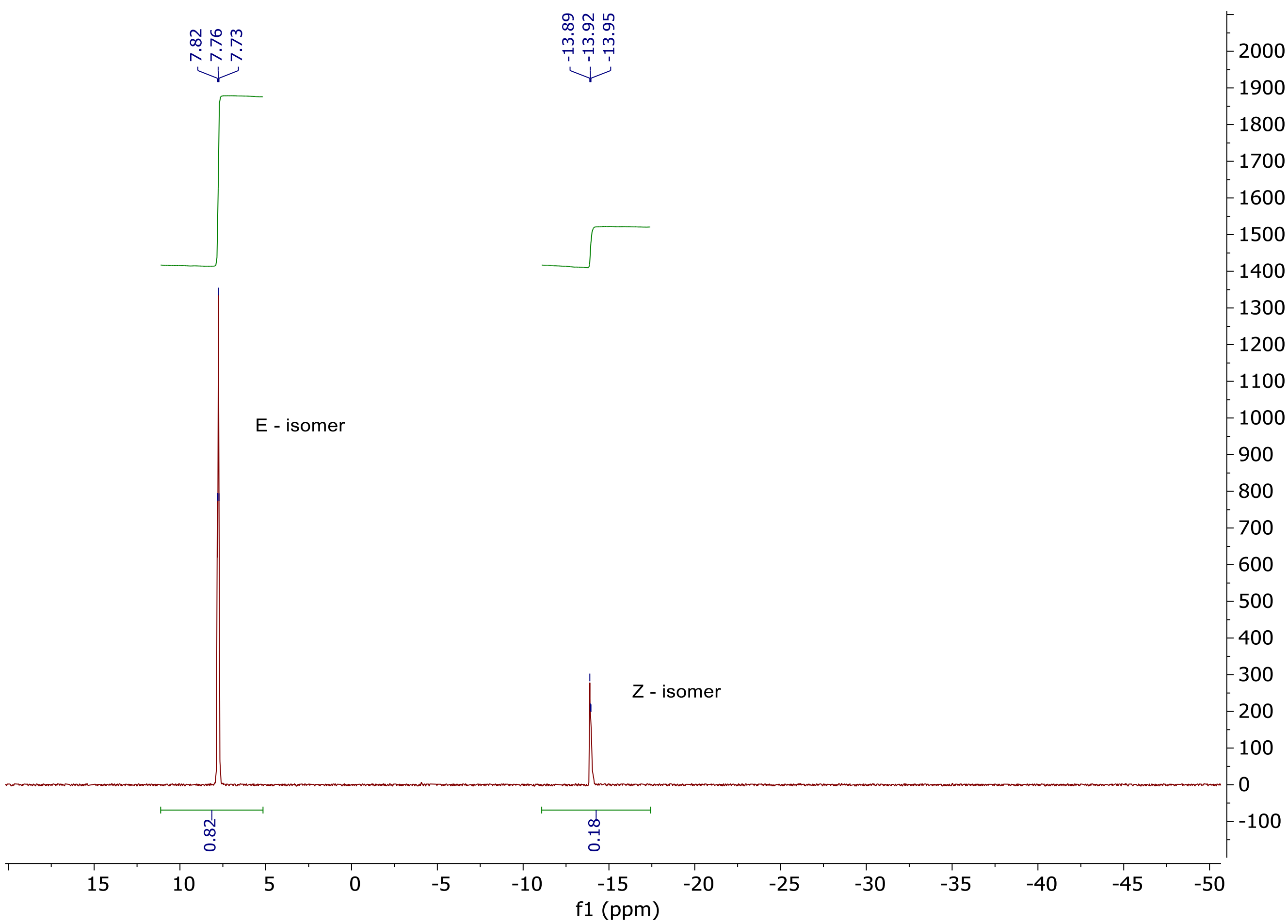

Figure S156: Hydrophosphination of 0.5 mmol 1-phenyl-1-propyne with equimolar of Ph_2PH in DMSO-d_6 using 5 mol% **1** – 0 h – $^{31}P\{^1H\}$ NMR

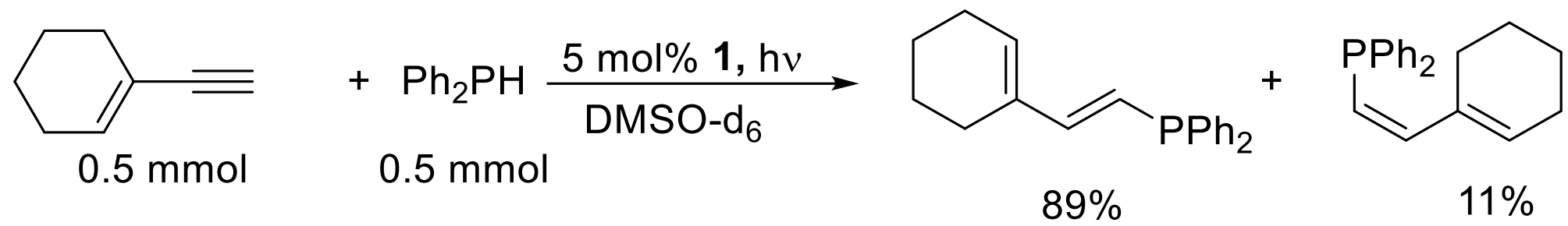

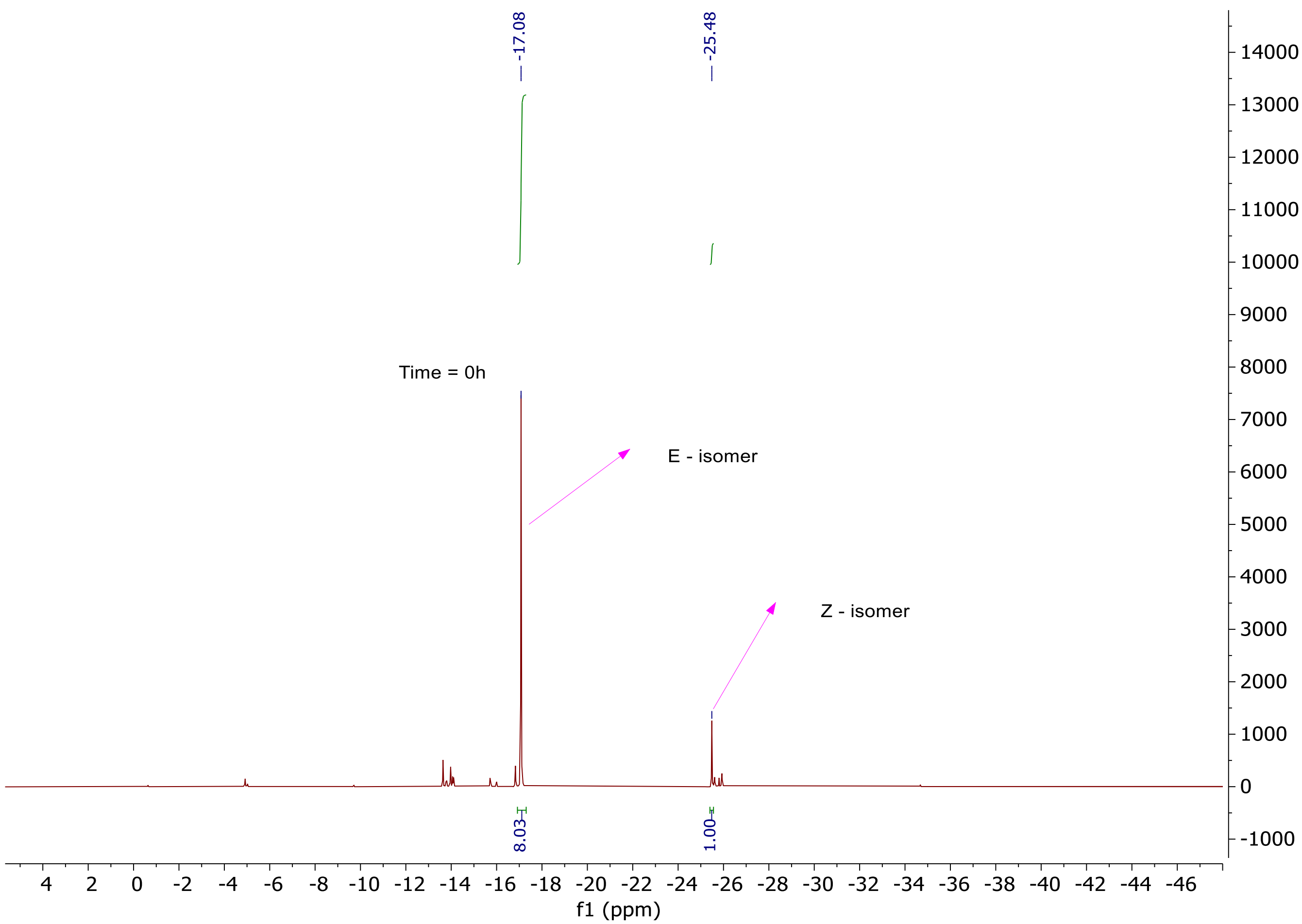

Figure S157: Hydrophosphination of 0.5 mmol 1-ethynyl cyclohexene with equimolar of Ph_2PH in DMSO-d_6 using 5 mol% **1** – 0 h – $^{31}P\{^1H\}$ NMR

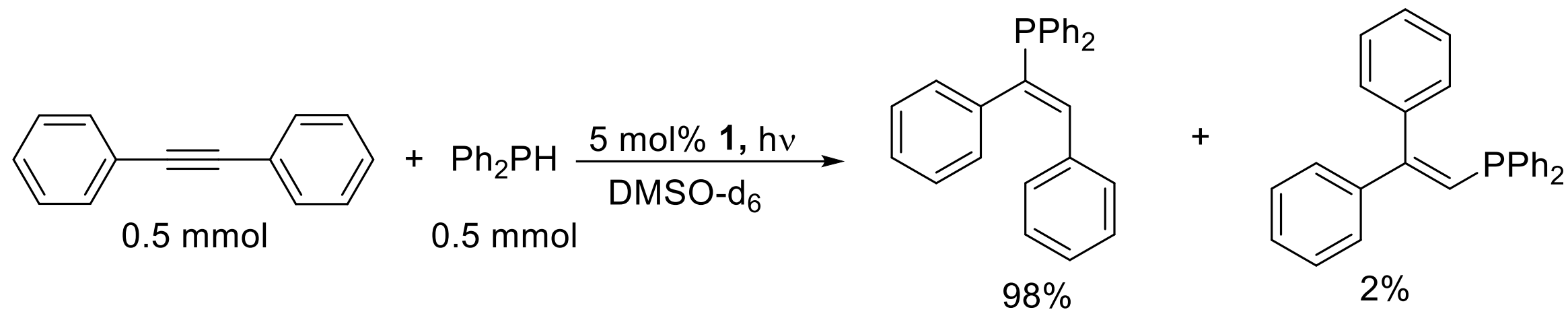

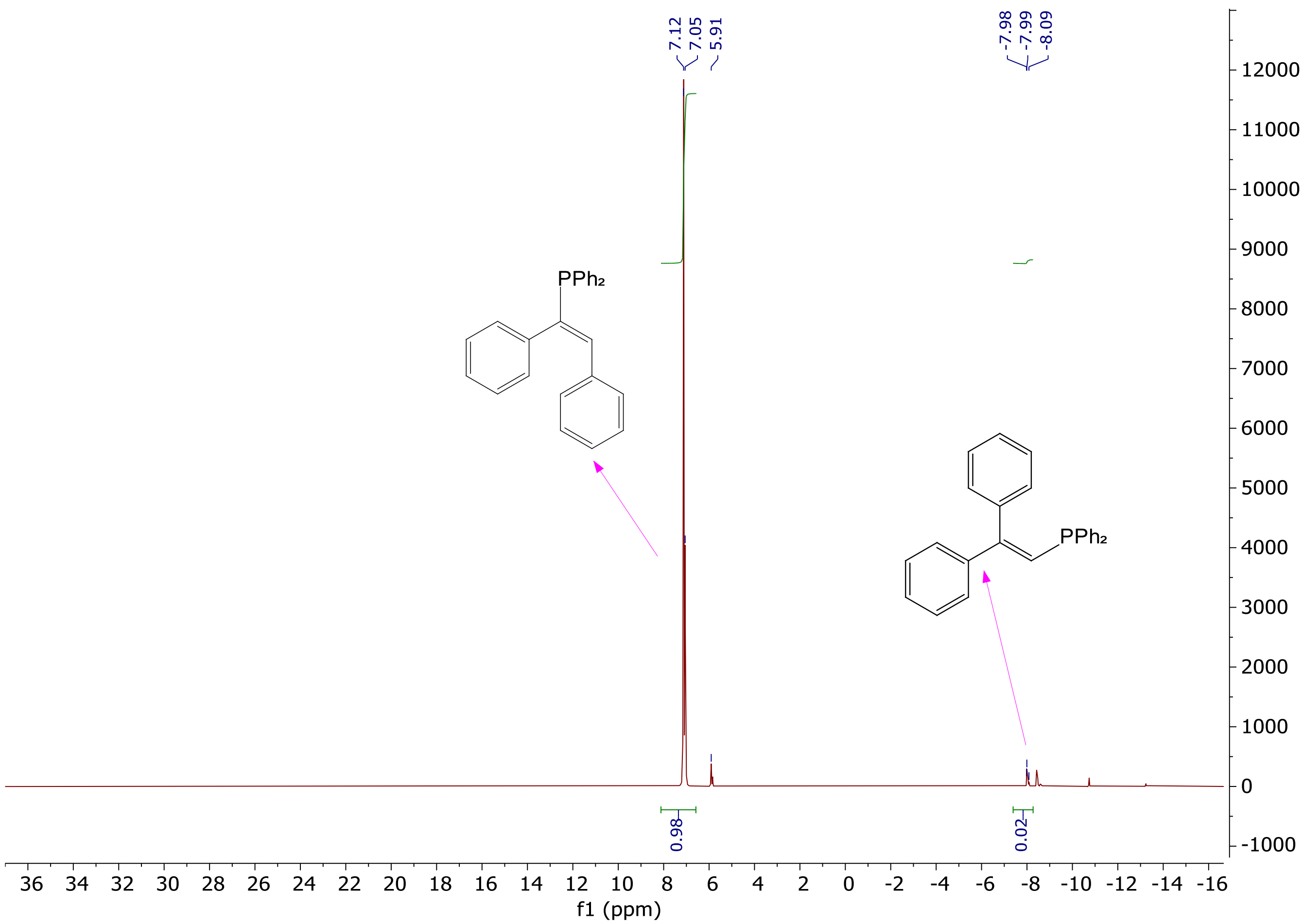

Figure S158: Hydrophosphination of 0.5 mmol diphenyl acetylene with equimolar of Ph_2PH in DMSO-d_6 using 5 mol% **1** – 0 h – $^{31}P\{^1H\}$ NMR

UV 360 nm irradiation, 24 h

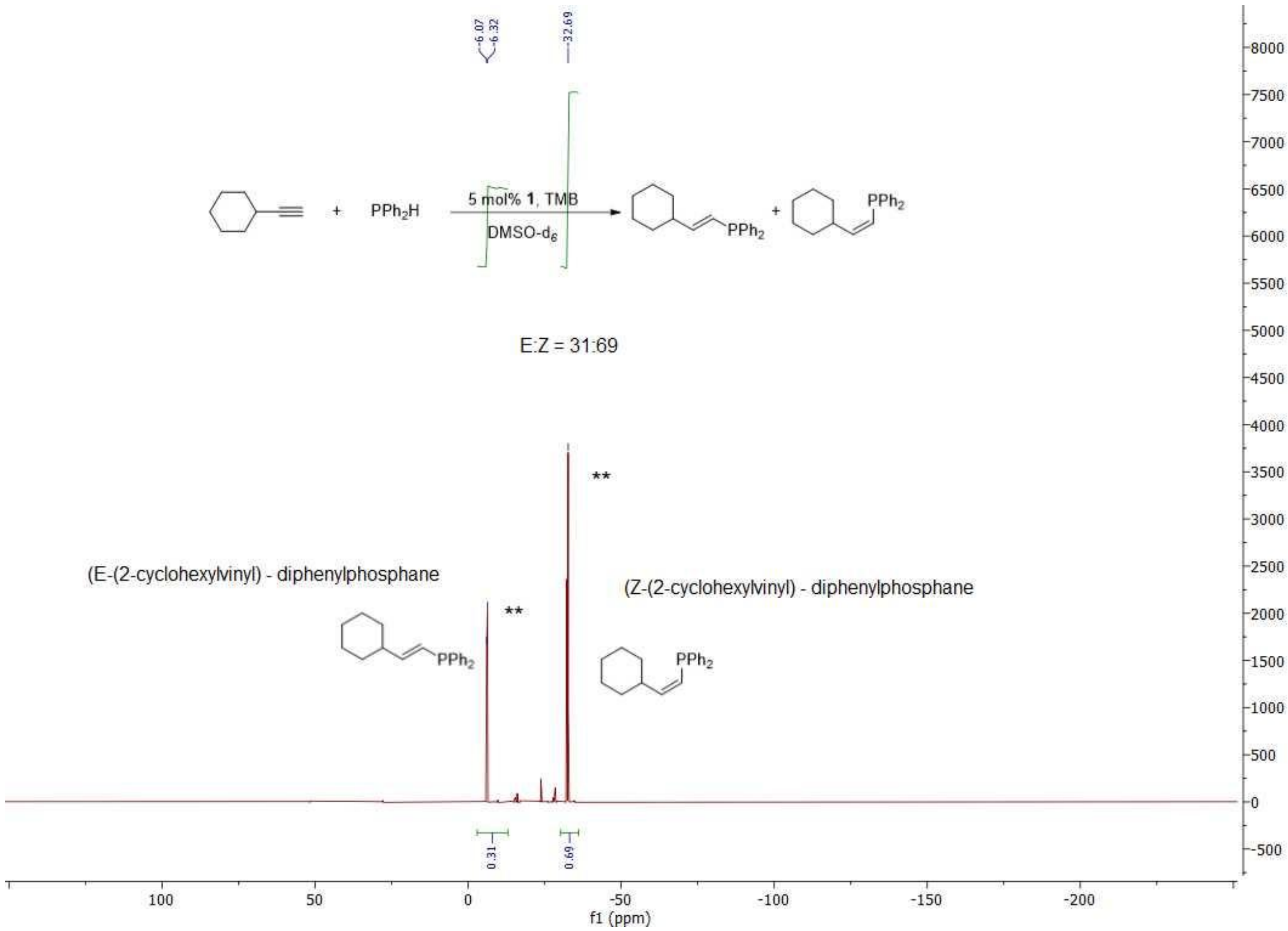

Figure S159: Hydrophosphination of 0.5 mmol ethynyl cyclohexane with equimolar of Ph_2PH in DMSO-d_6 using 5 mol% **1** under UV 360 nm light – 24 h –$^{31}P\{^1H\}$ NMR

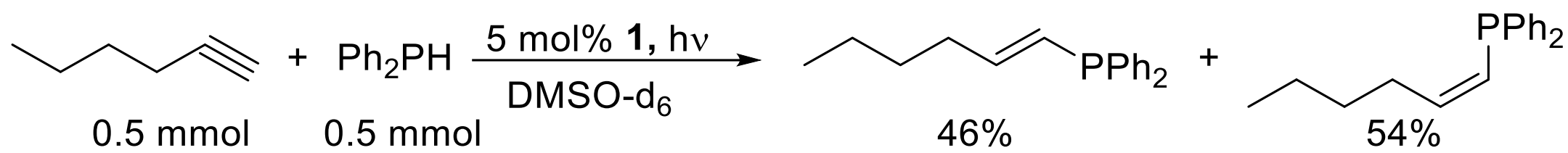

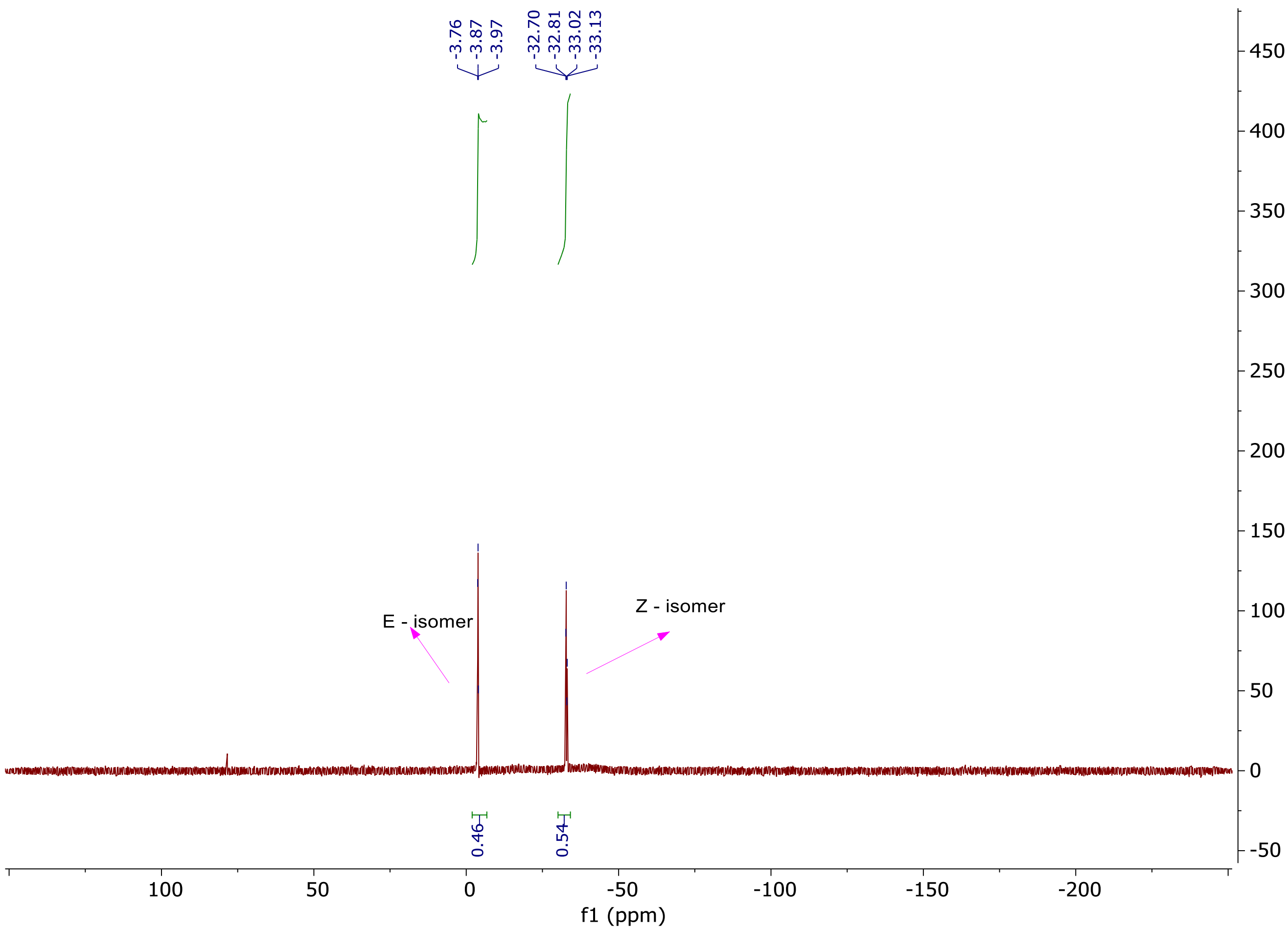

Figure S160: Hydrophosphination of 0.5 mmol 1-hexyne with equimolar of Ph_2PH in DMSO-d_6 using 5 mol% **1** – 0 h –$^{31}P\{^1H\}$ NMR

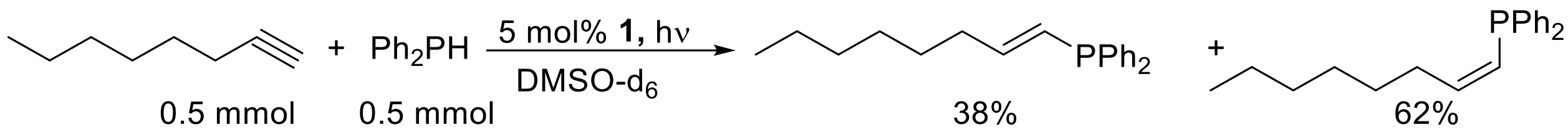

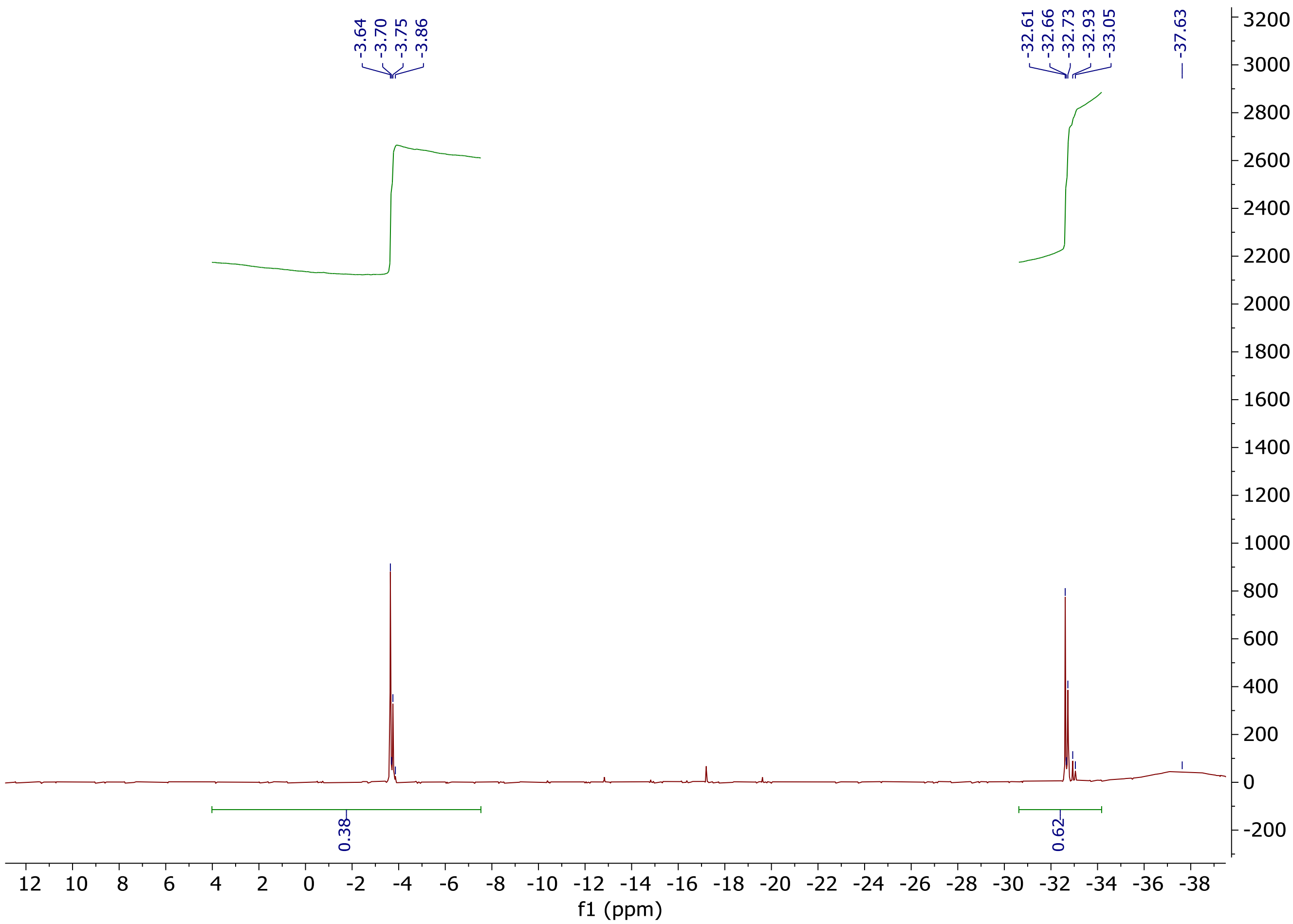

Figure S161: Hydrophosphination of 0.5 mmol 1-octyne with equimolar of Ph_2PH in DMSO-d_6 using 5 mol% **1** under UV 360 nm irradiation – 4 h – $^{31}P\{^1H\}$ NMR

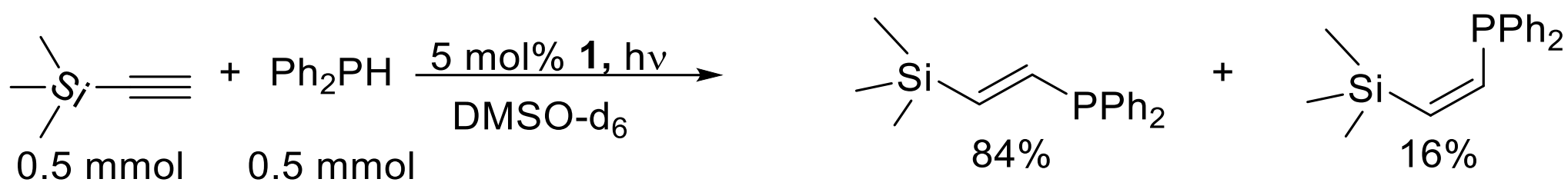

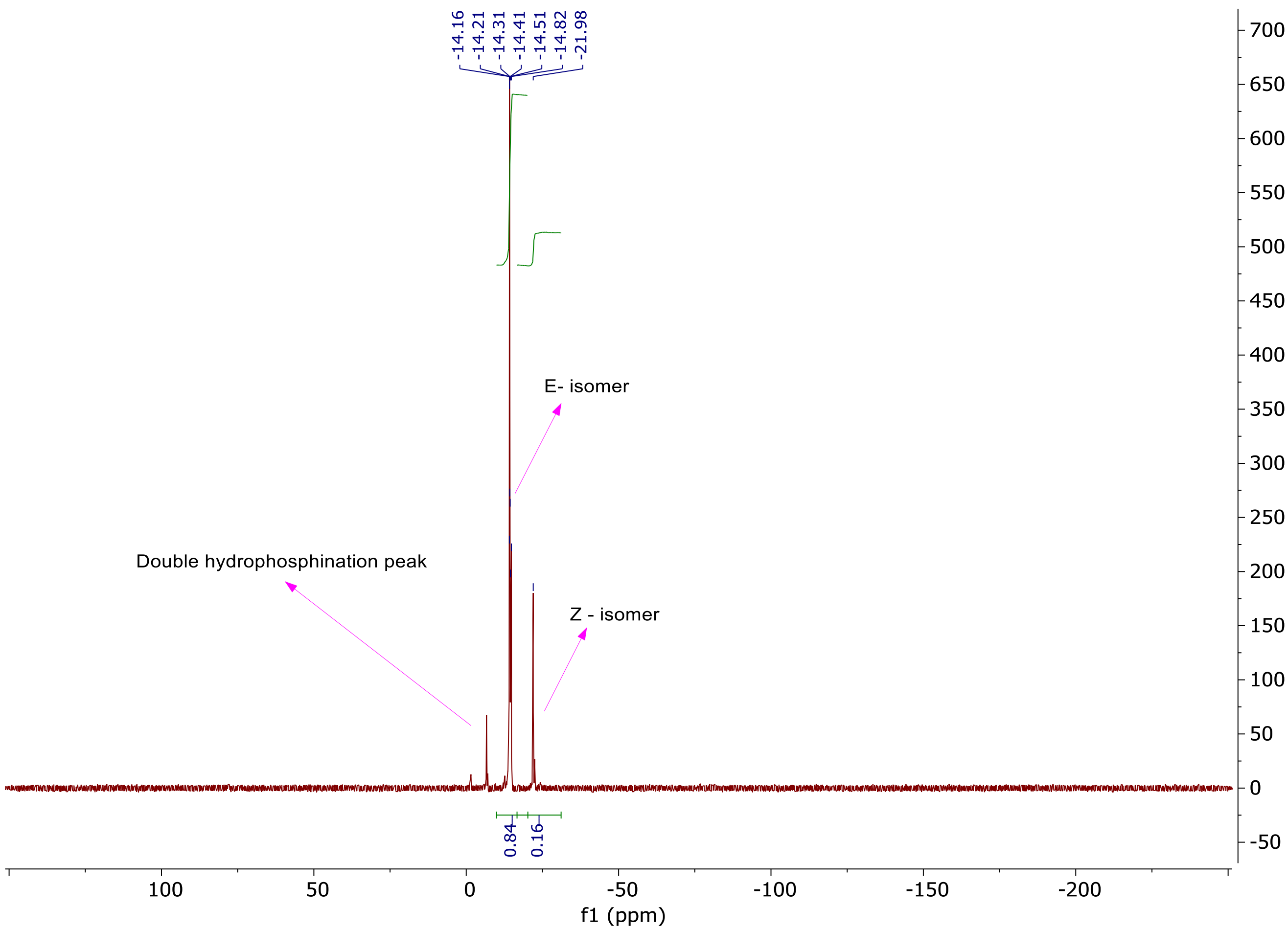

Figure S162: Hydrophosphination of 0.5 mmol trimethyl silyl acetylene with equimolar of Ph_2PH in DMSO-d_6 using 5 mol% **1** – 0 h –$^{31}P\{^1H\}$ NMR

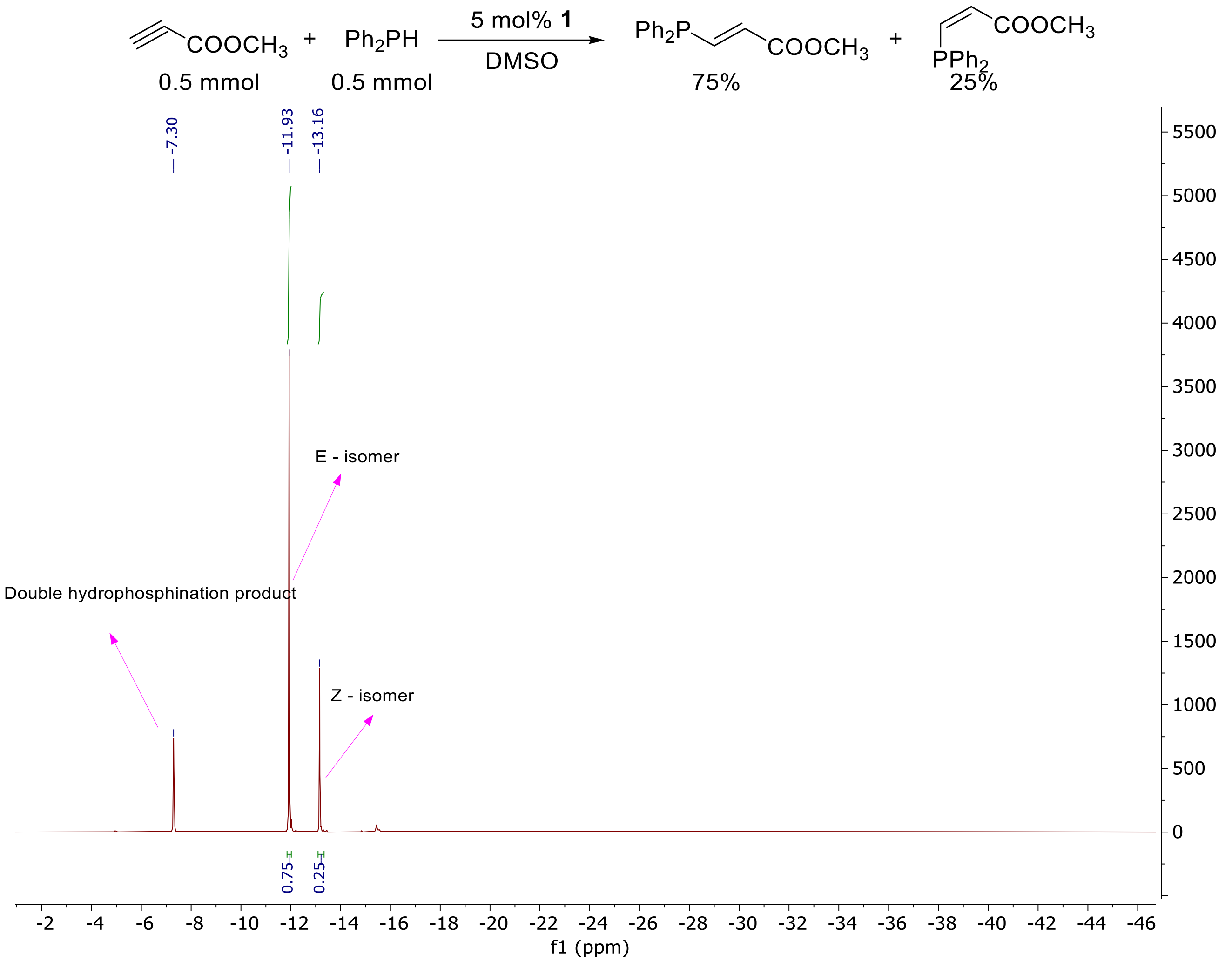

Figure S163: Hydrophosphination of 0.5 mmol methyl propiolate with equimolar of Ph_2PH in DMSO-d_6 using 5 mol% **1** – 0 h –$^{31}P\{^1H\}$ NMR

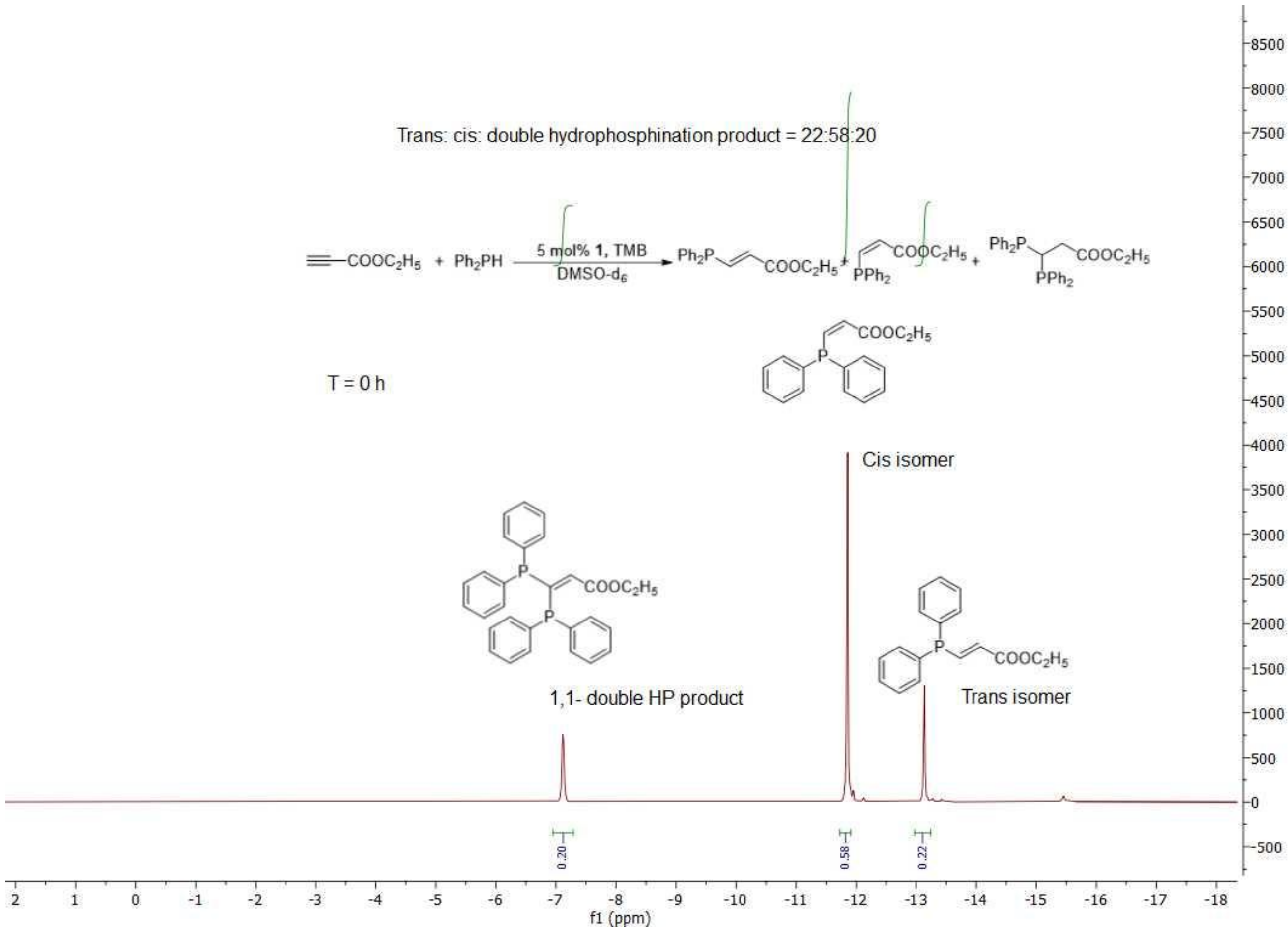

Figure S164: Hydrophosphination of 0.5 mmol ethyl propiolate with equimolar of Ph_2PH in DMSO-d_6 using 5 mol% **1** – 0 h –$^{31}P\{^1H\}$ NMR

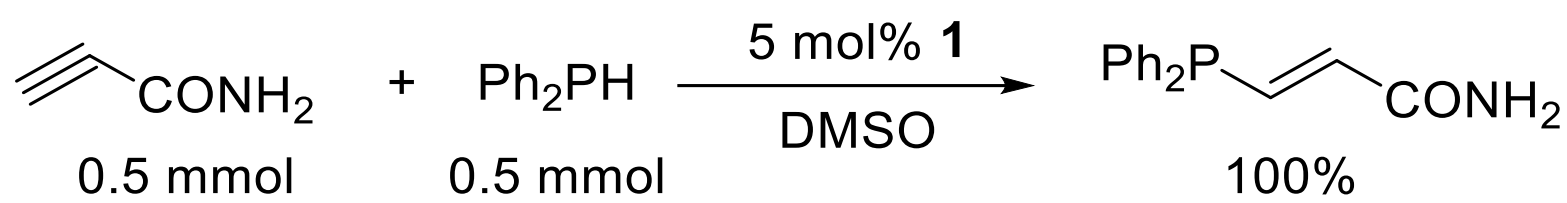

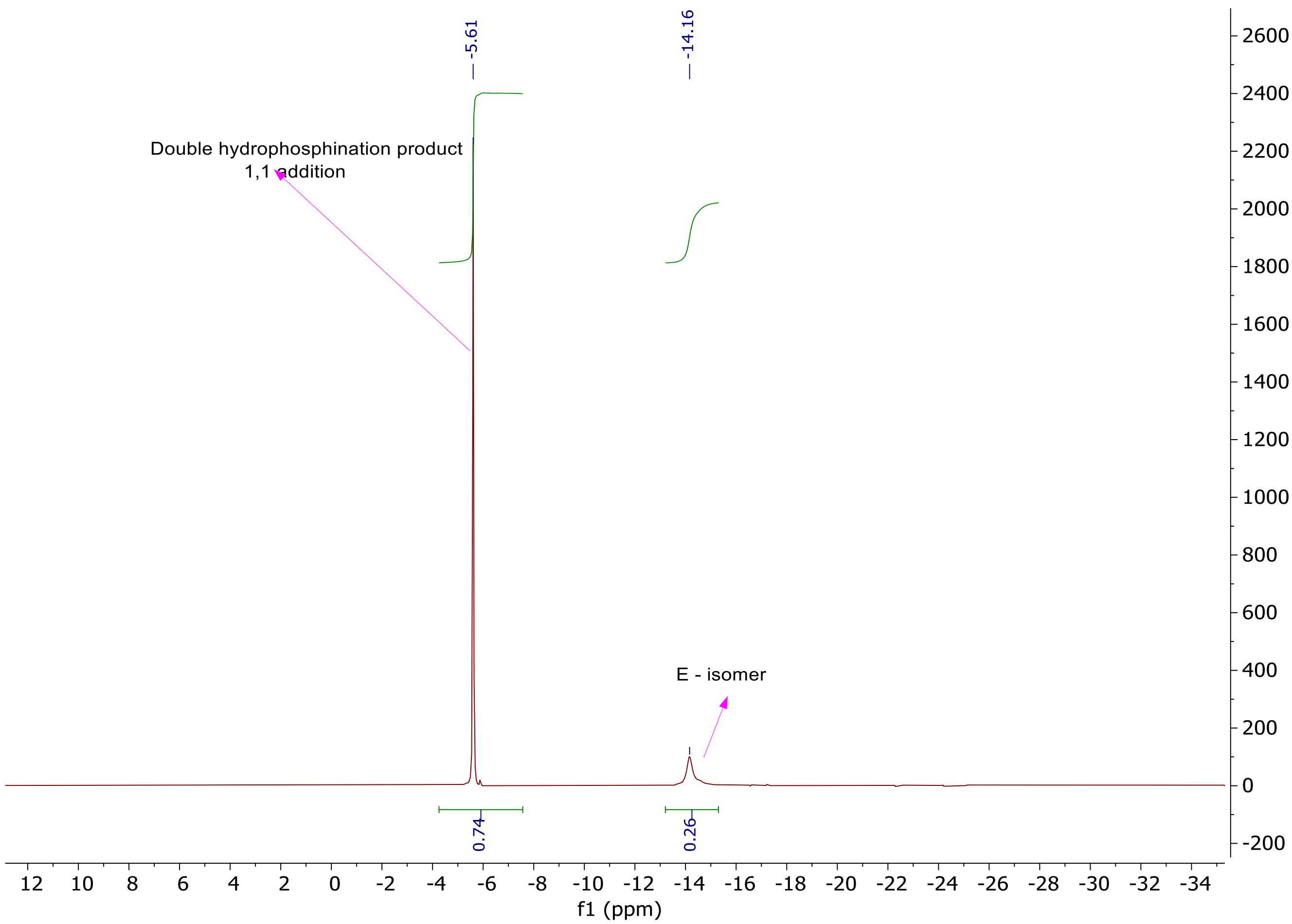

Figure S165: Hydrophosphination of 0.5 mmol propiolamide with equimolar of Ph_2PH in DMSO-d_6 using 5 mol% **1** – 0 h –$^{31}P\{^1H\}$ NMR

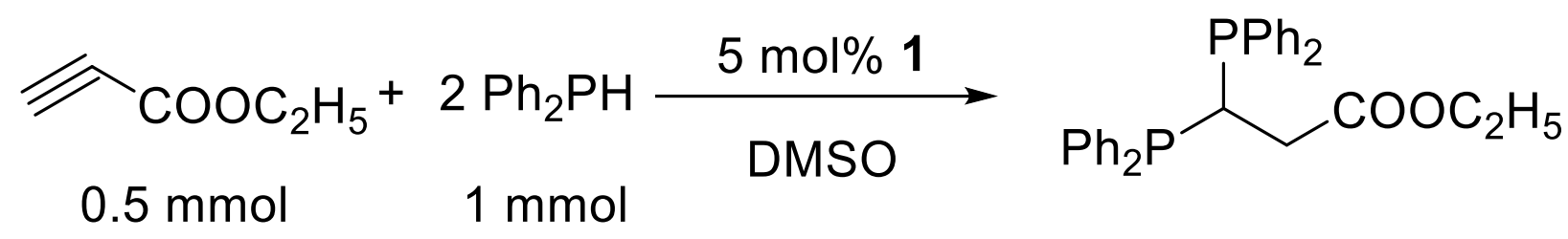

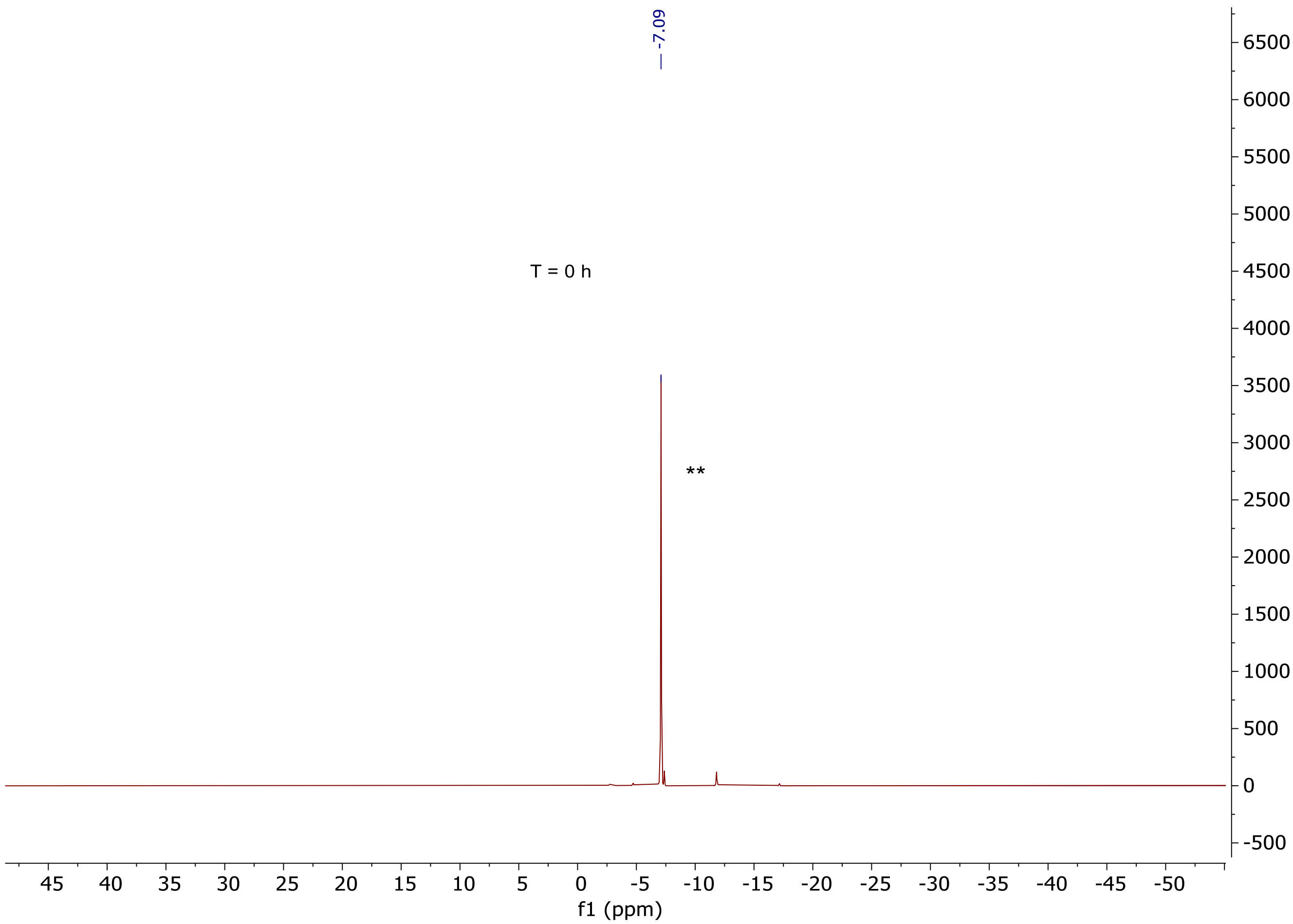

Figure S166: Double hydrophosphination of 0.5 mmol ethyl propiolate with 2 equivalents of Ph_2PH in DMSO-d_6 using 5 mol% **1** – 0 h – $^{31}P\{^1H\}$ NMR (1,1 – addition product)

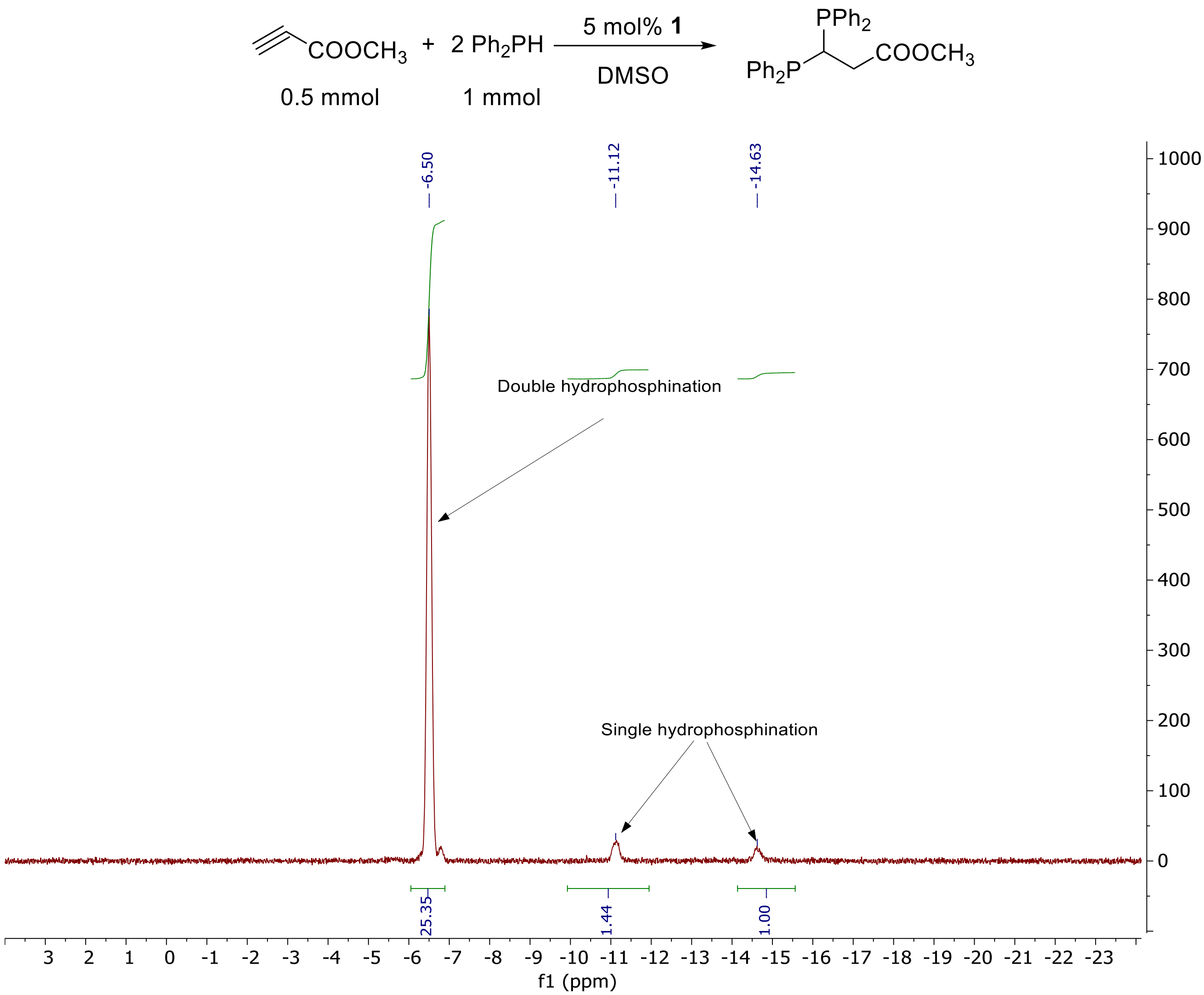

Figure S167: Double hydrophosphination of 0.5 mmol methyl propiolate with 2 equivalents of Ph_2PH in DMSO-d_6 using 5 mol% **1** – 0 h –$^{31}P\{^1H\}$ NMR (1,1 – addition product)

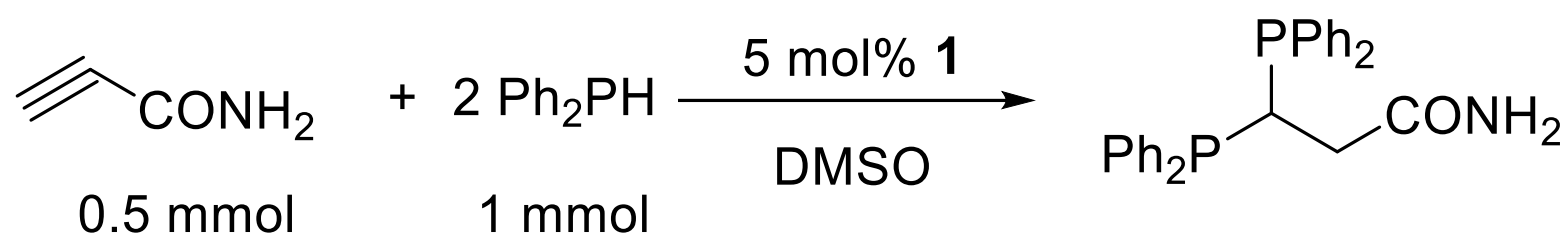

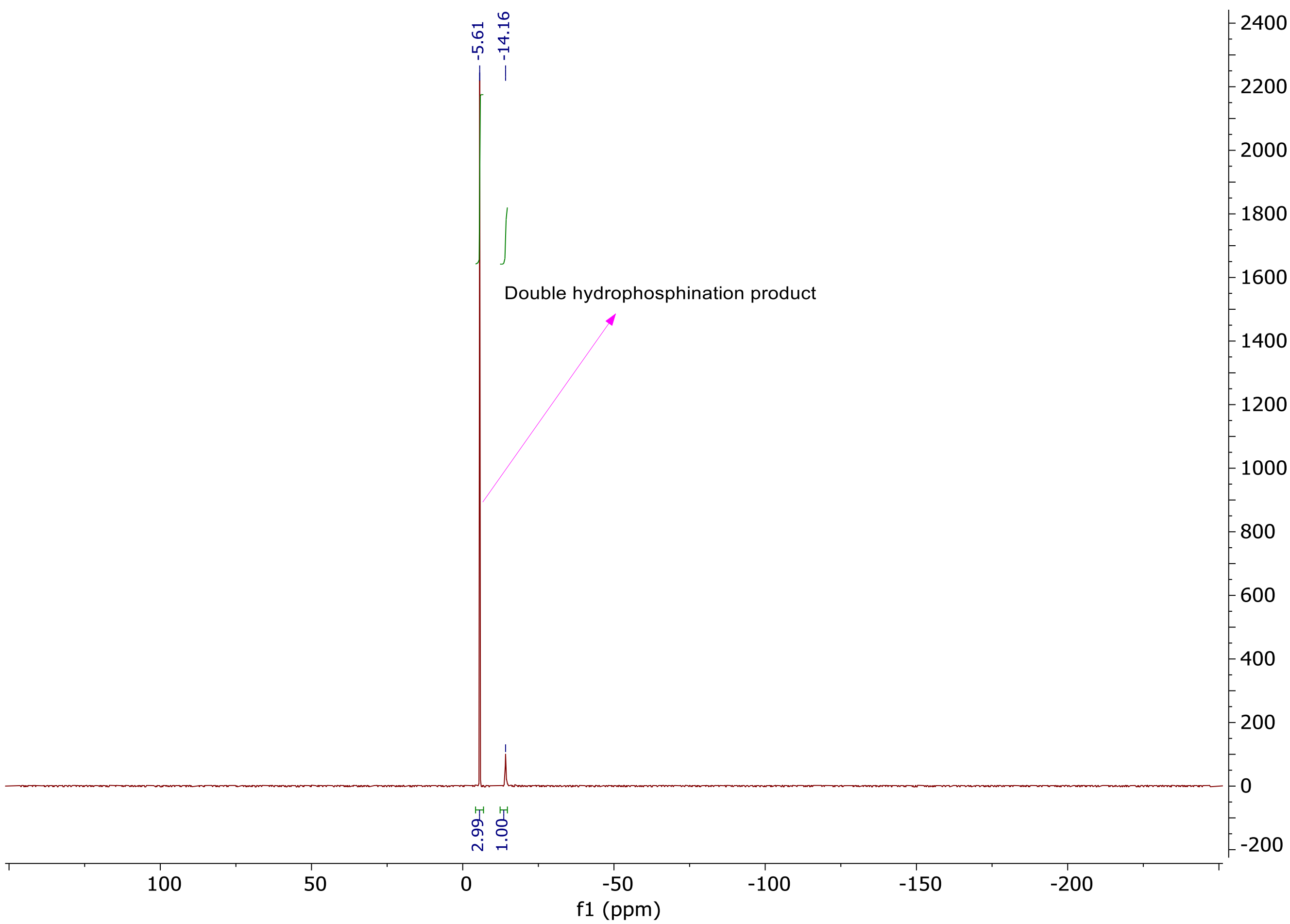

Figure S168: Double hydrophosphination of 0.5 mmol propiolamide with 2 equivalents of Ph_2PH in DMSO-d_6 using 5 mol% **1** – 0 h –$^{31}P\{^1H\}$ NMR (1,1 – addition product)

Hydrophosphination of unsaturated compounds with phenyl phosphine using 5 mol% of **1** in DMSO-d_6

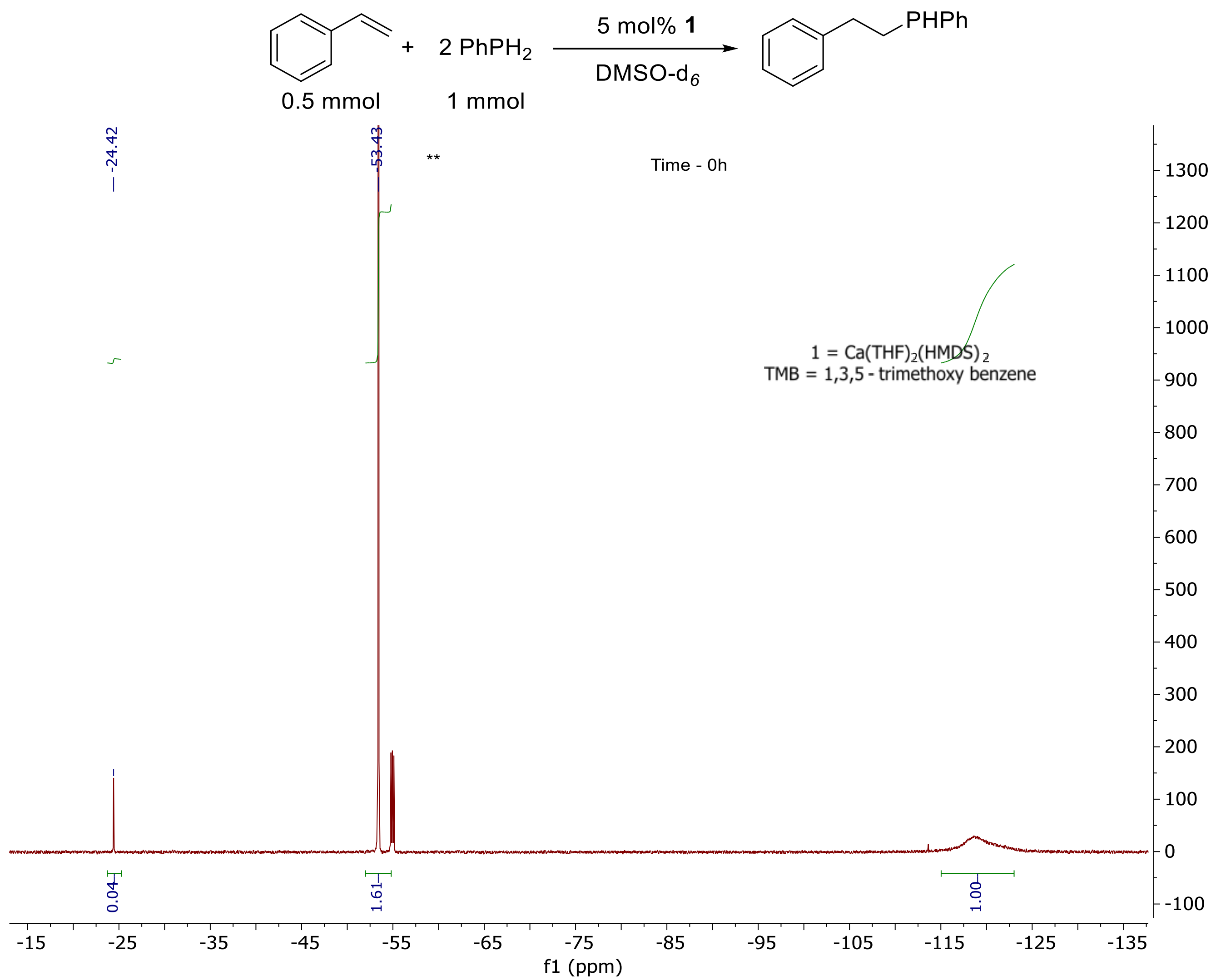

Figure S169: Hydrophosphination of 0.5 mmol styrene with 2 equivalents of $PhPH_2$ in DMSO-d_6 using 5 mol% **1** – 0 h – $^{31}P\{^1H\}$ NMR.

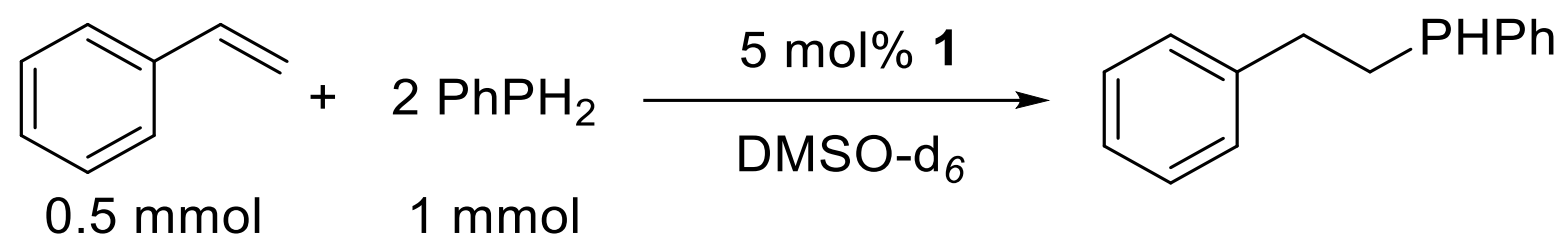

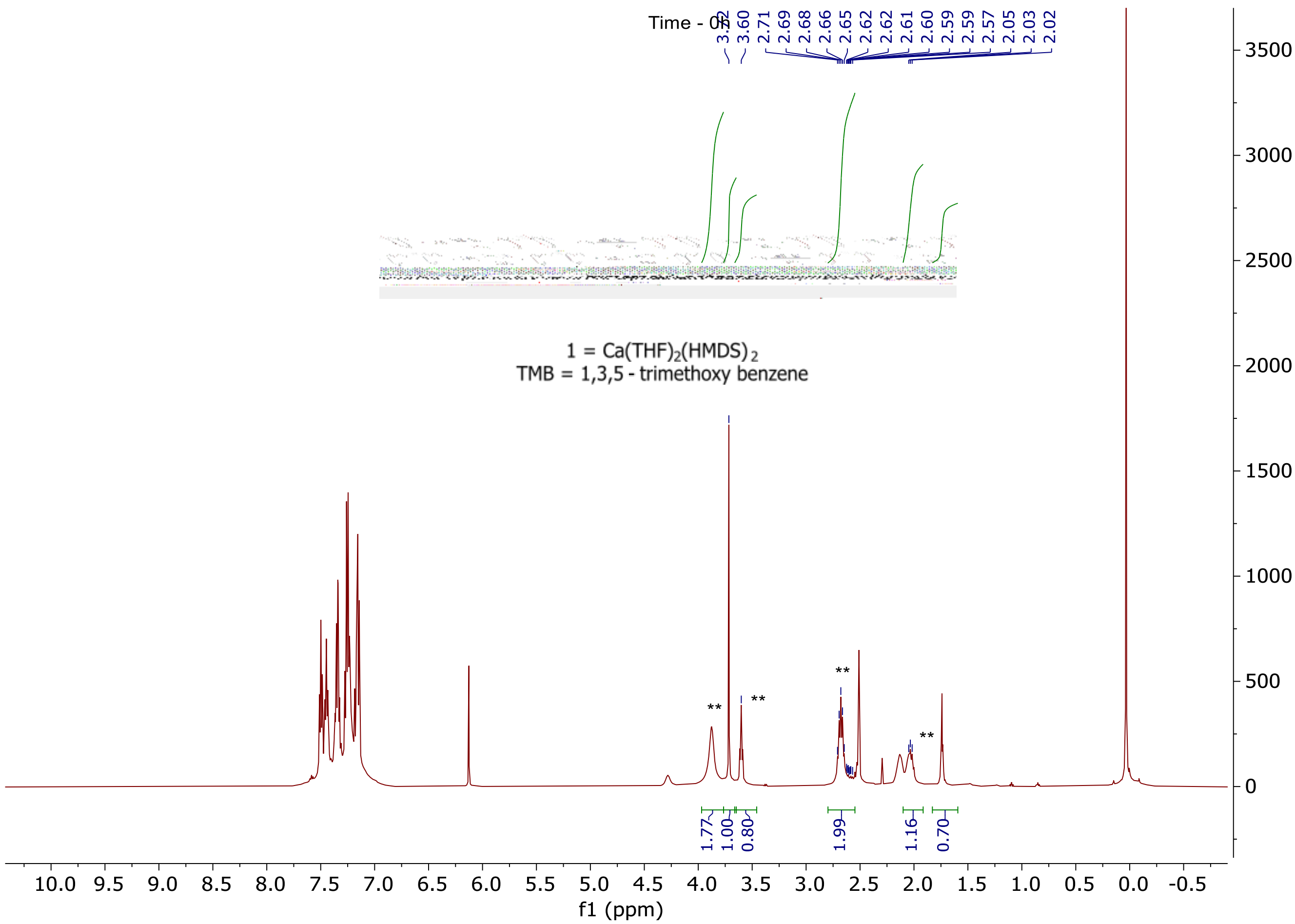

Figure S170: Hydrophosphination of 0.5 mmol styrene with 2 equivalents of $PhPH_2$ in DMSO-d_6 using 5 mol% **1** – 0 h –^{1}H NMR.

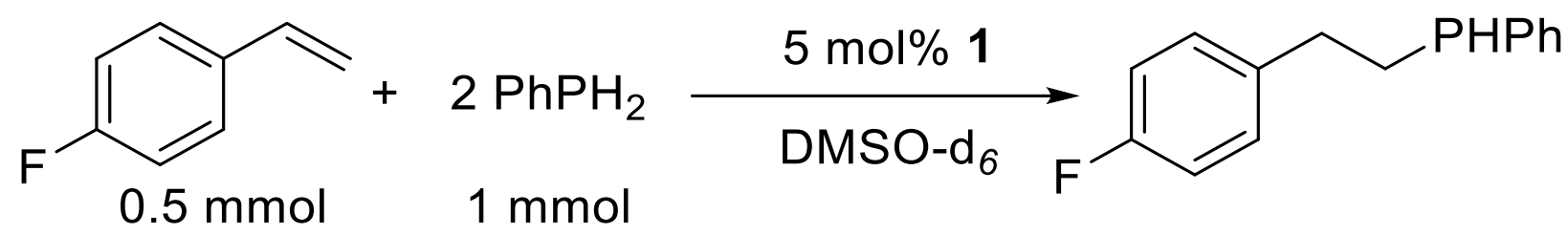

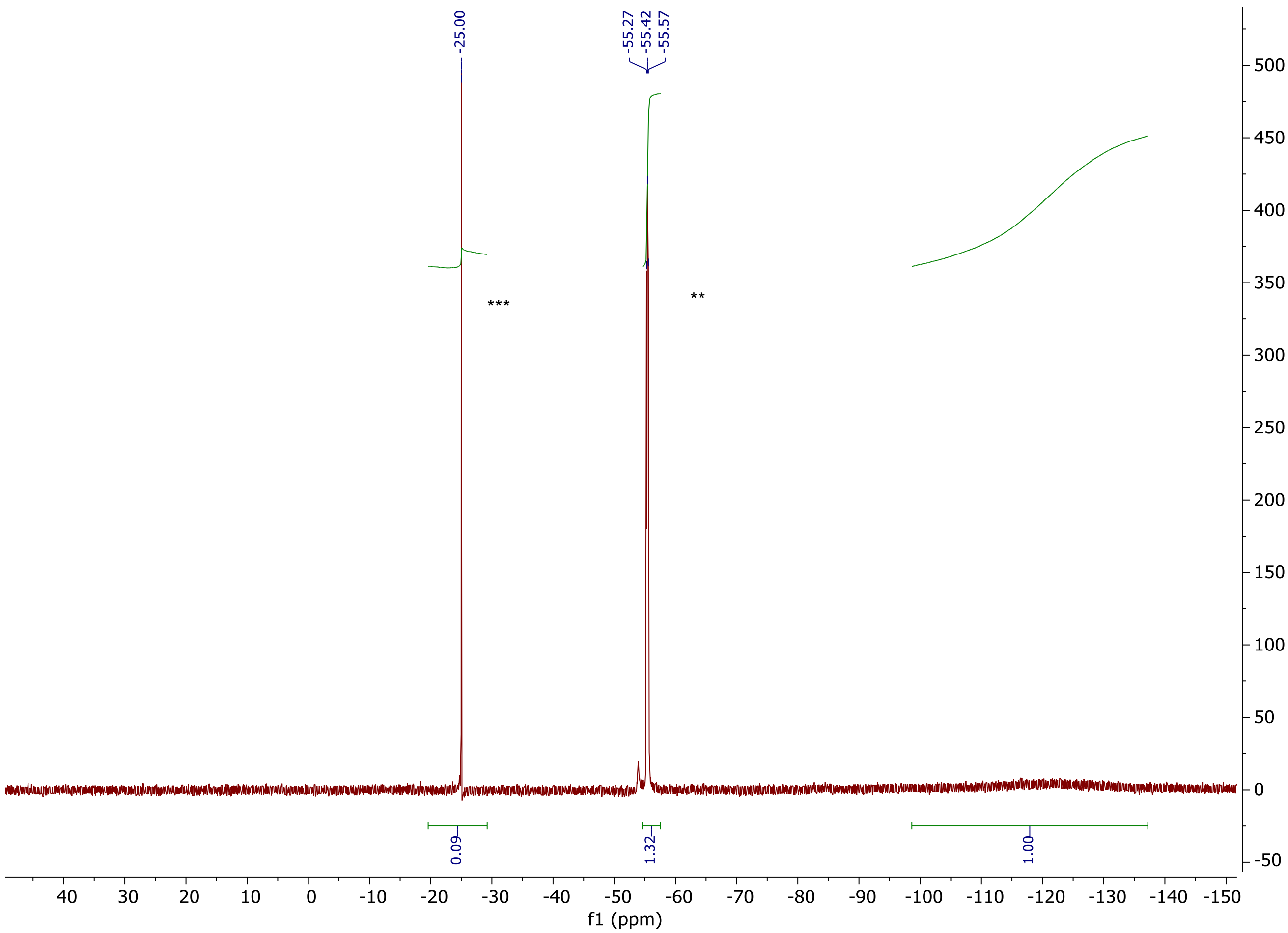

Figure S171: Hydrophosphination of 0.5 mmol 4-fluorostyrene with 2 equivalents of $PhPH_2$ in DMSO-d_6 using 5 mol% **1** – 0 h – $^{31}P\{^1H\}$ NMR.

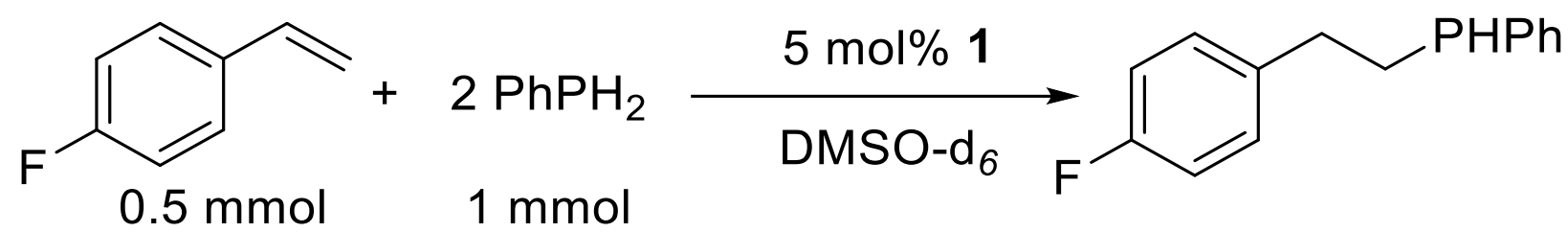

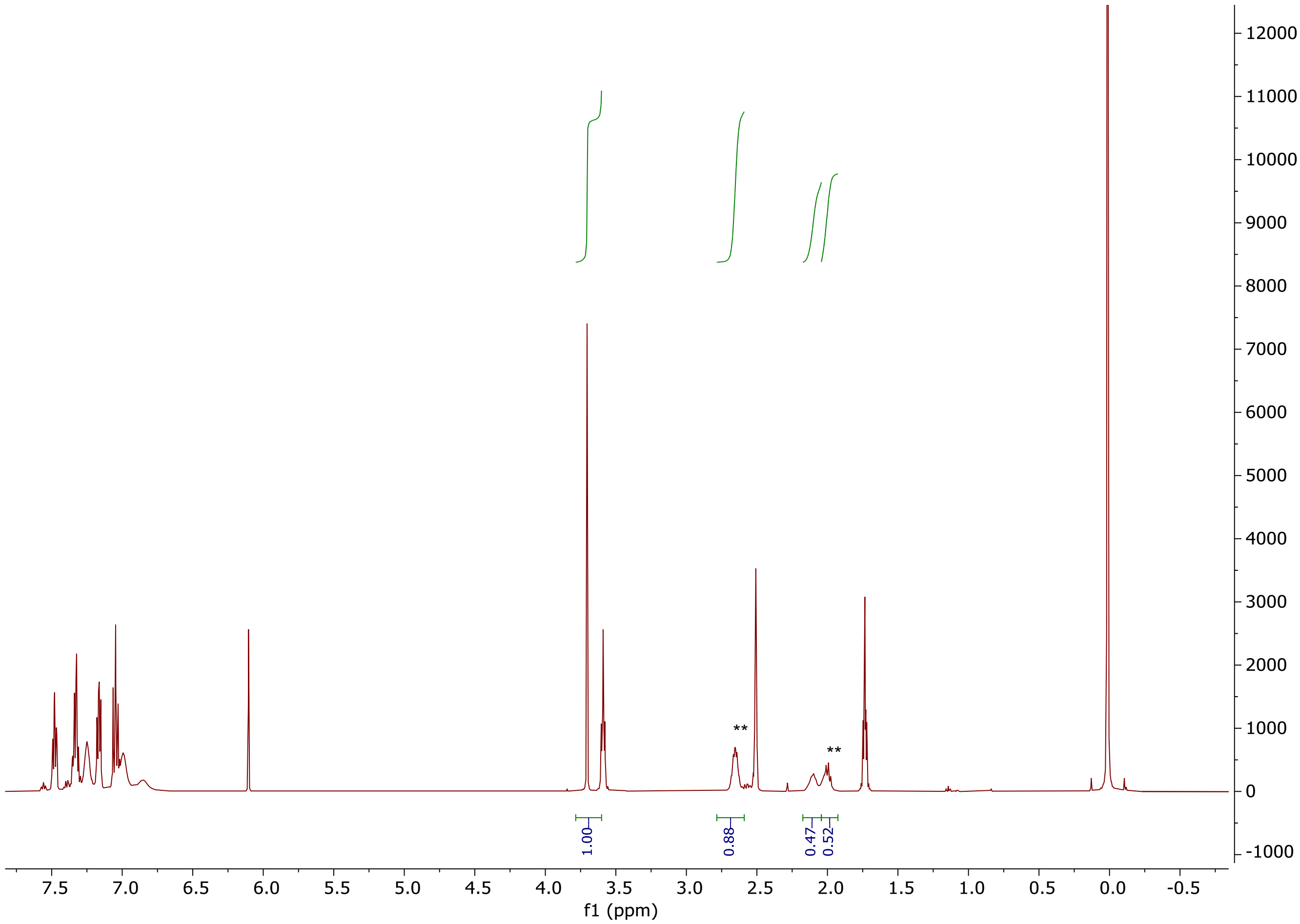

Figure S172: Hydrophosphination of 0.5 mmol 4-fluorostyrene with 2 equivalents of $PhPH_2$ in DMSO-d_6 using 5 mol% **1** – 0 h – ^{1}H NMR.

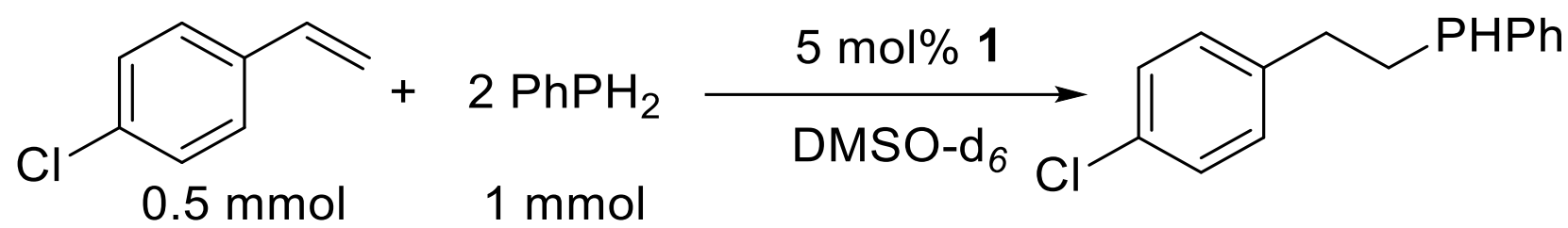

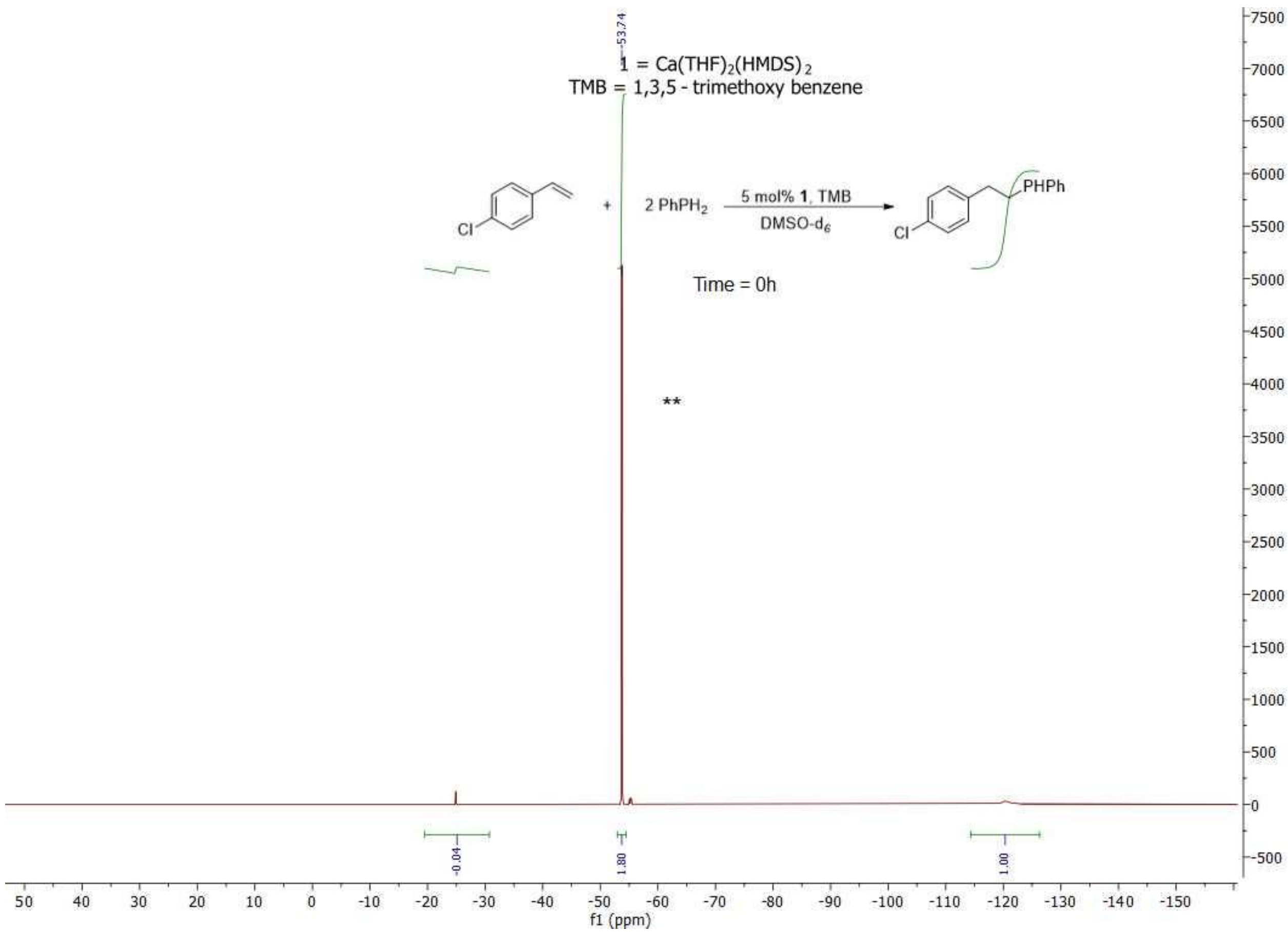

Figure S173: Hydrophosphination of 0.5 mmol 4-chlorostyrene with 2 equivalents of $PhPH_2$ in DMSO-d_6 using 5 mol% **1** – 0 h – $^{31}P\{^1H\}$ NMR.

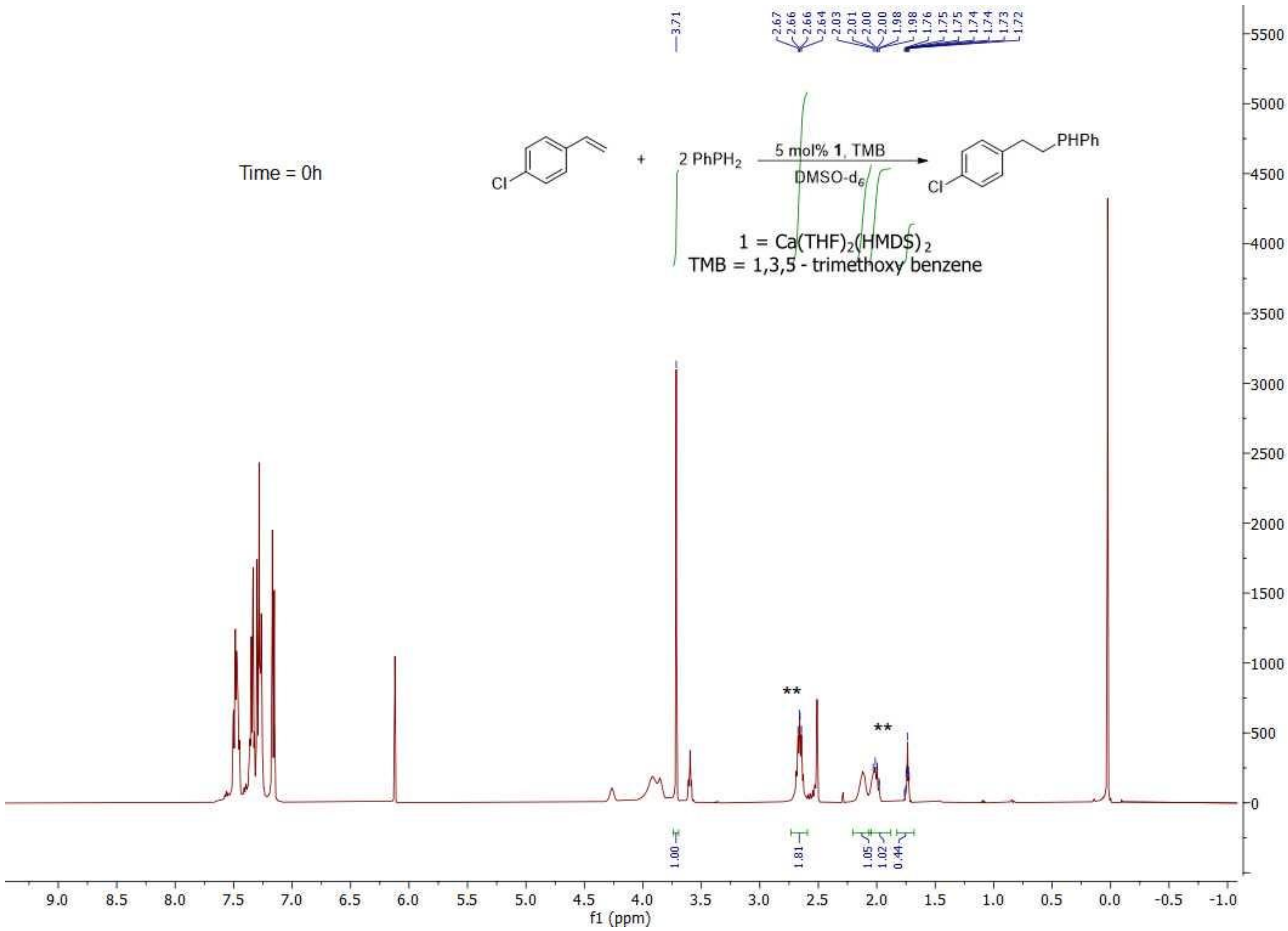

Figure S174: Hydrophosphination of 0.5 mmol 4-chlorostyrene with 2 equivalents of $PhPH_2$ in DMSO-d_6 using 5 mol% **1** – 0 h – ^{1}H NMR.

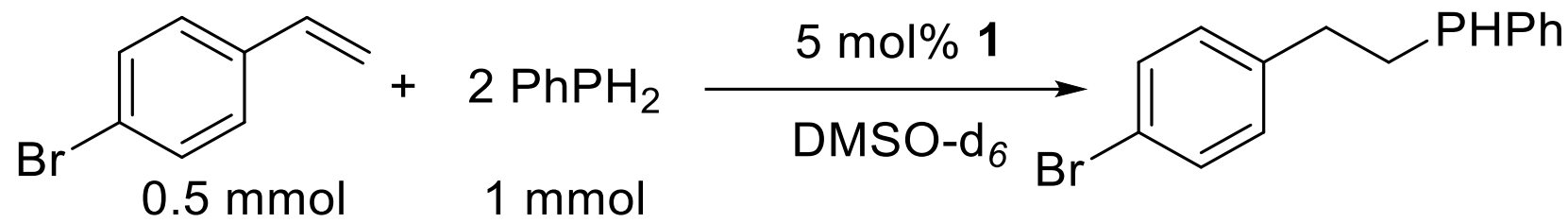

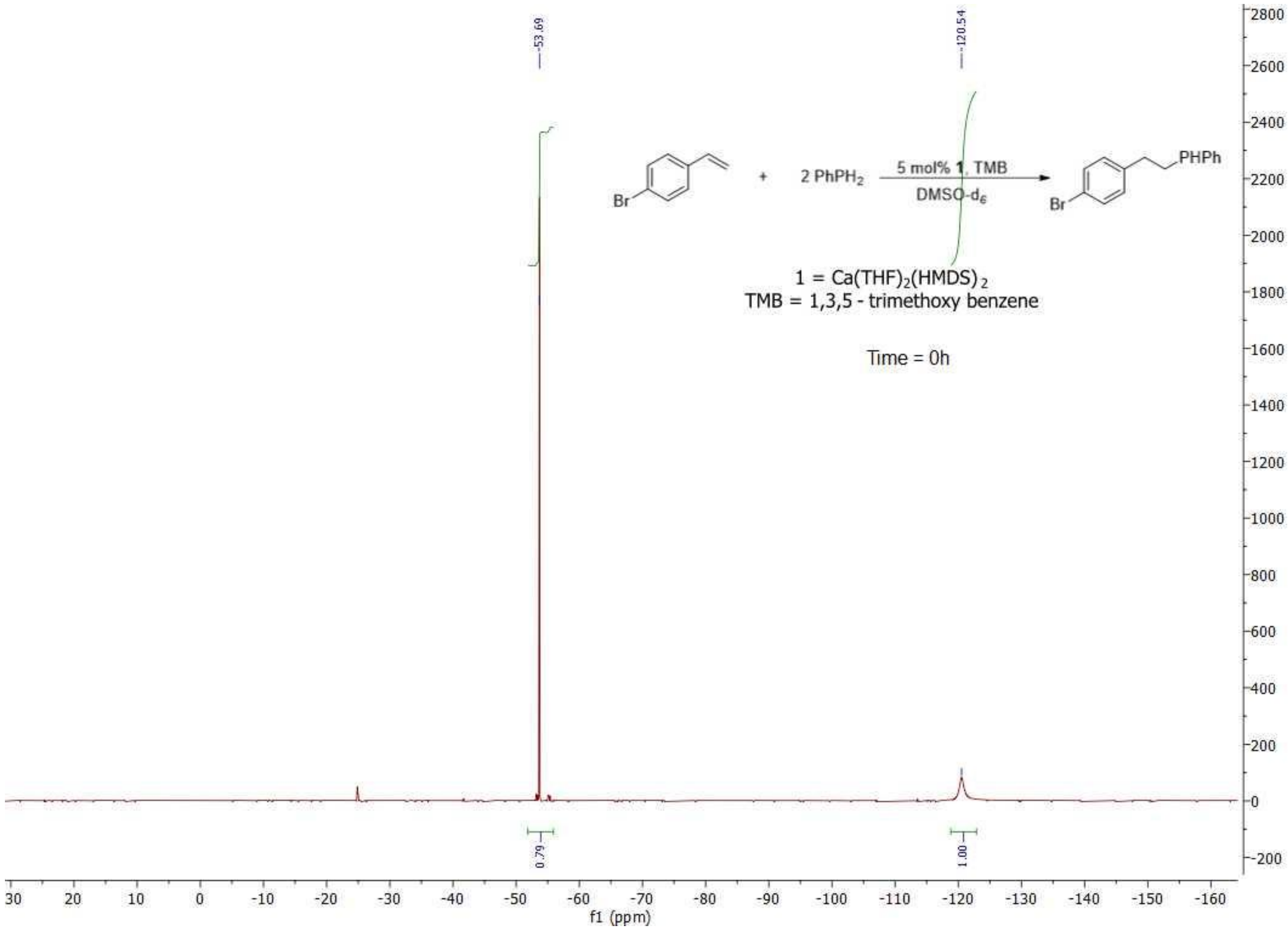

Figure S175: Hydrophosphination of 0.5 mmol 4-bromostyrene with 2 equivalents of $PhPH_2$ in DMSO-d_6 using 5 mol% **1** – 0 h – $^{31}P\{^1H\}$ NMR.

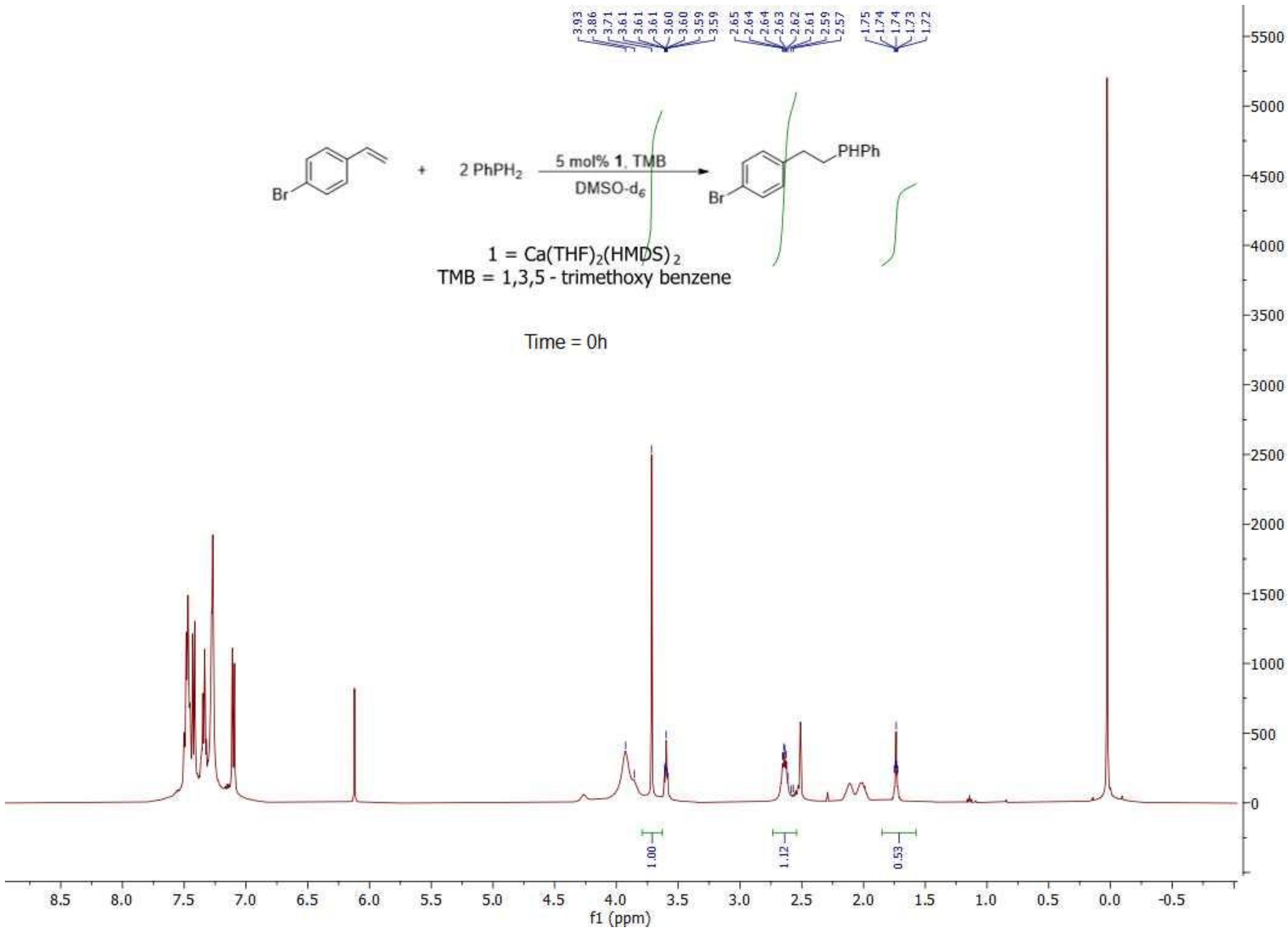

Figure S176: Hydrophosphination of 0.5 mmol 4-bromostyrene with 2 equivalents of $PhPH_2$ in DMSO-d_6 using 5 mol% **1** – 0 h – ^{1}H NMR.

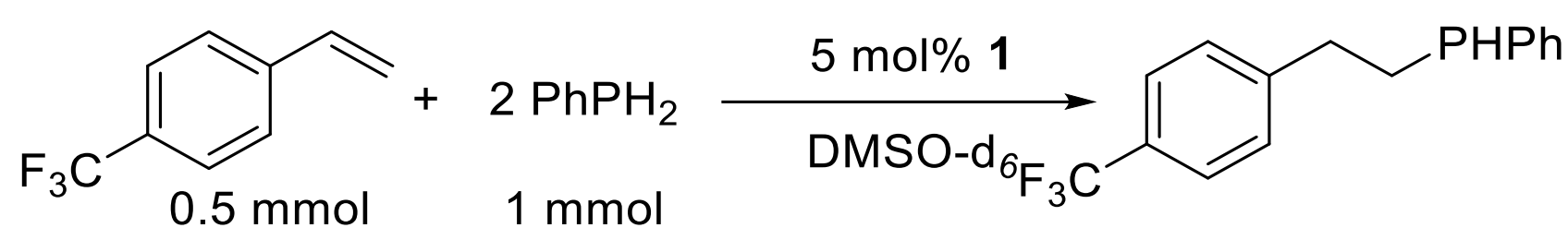

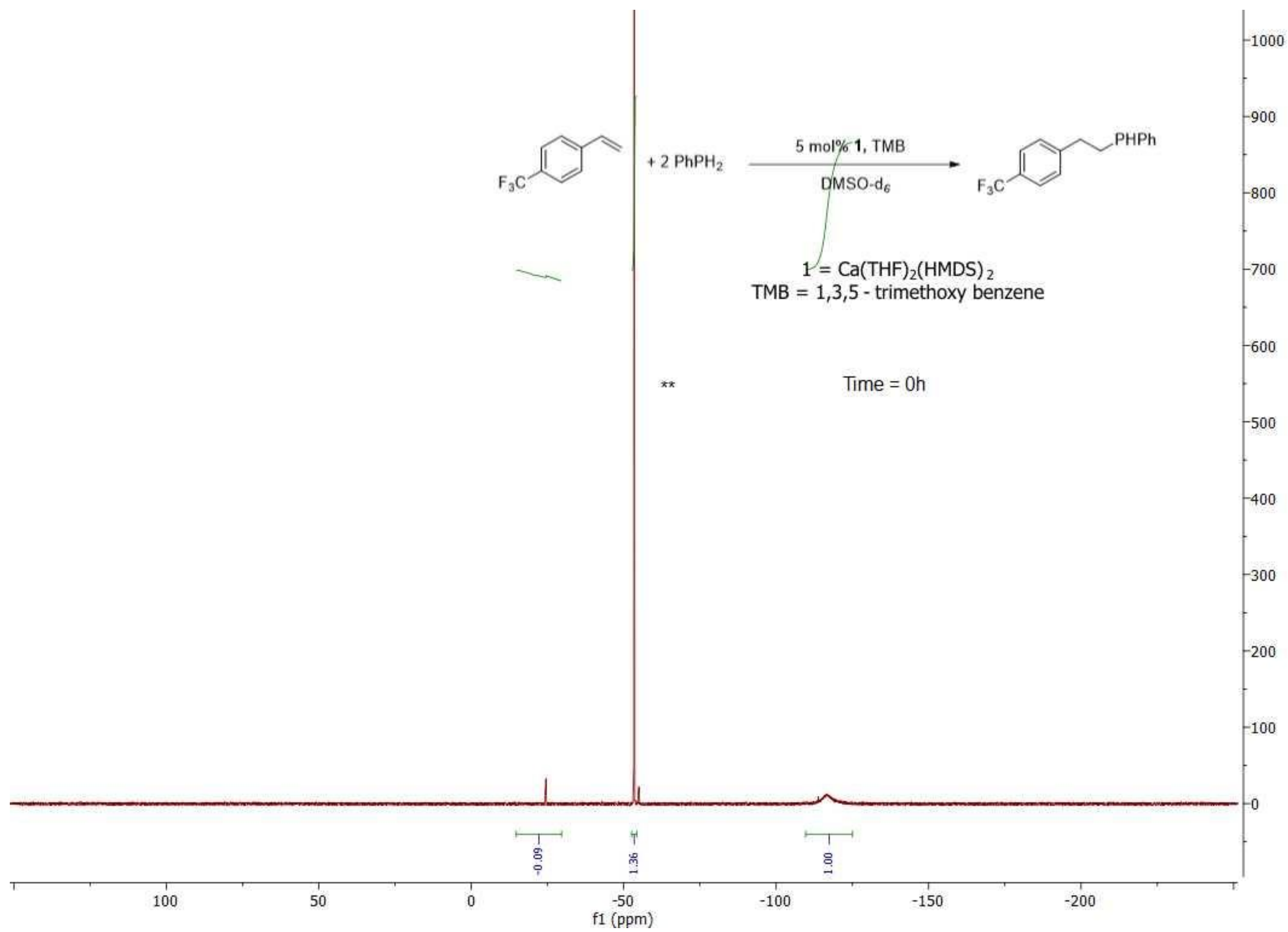

Figure S177: Hydrophosphination of 0.5 mmol 4-trifluoromethylstyrene with 2 equivalents of $PhPH_2$ in DMSO-d_6 using 5 mol% **1** – 0 h – $^{31}P\{^1H\}$ NMR.

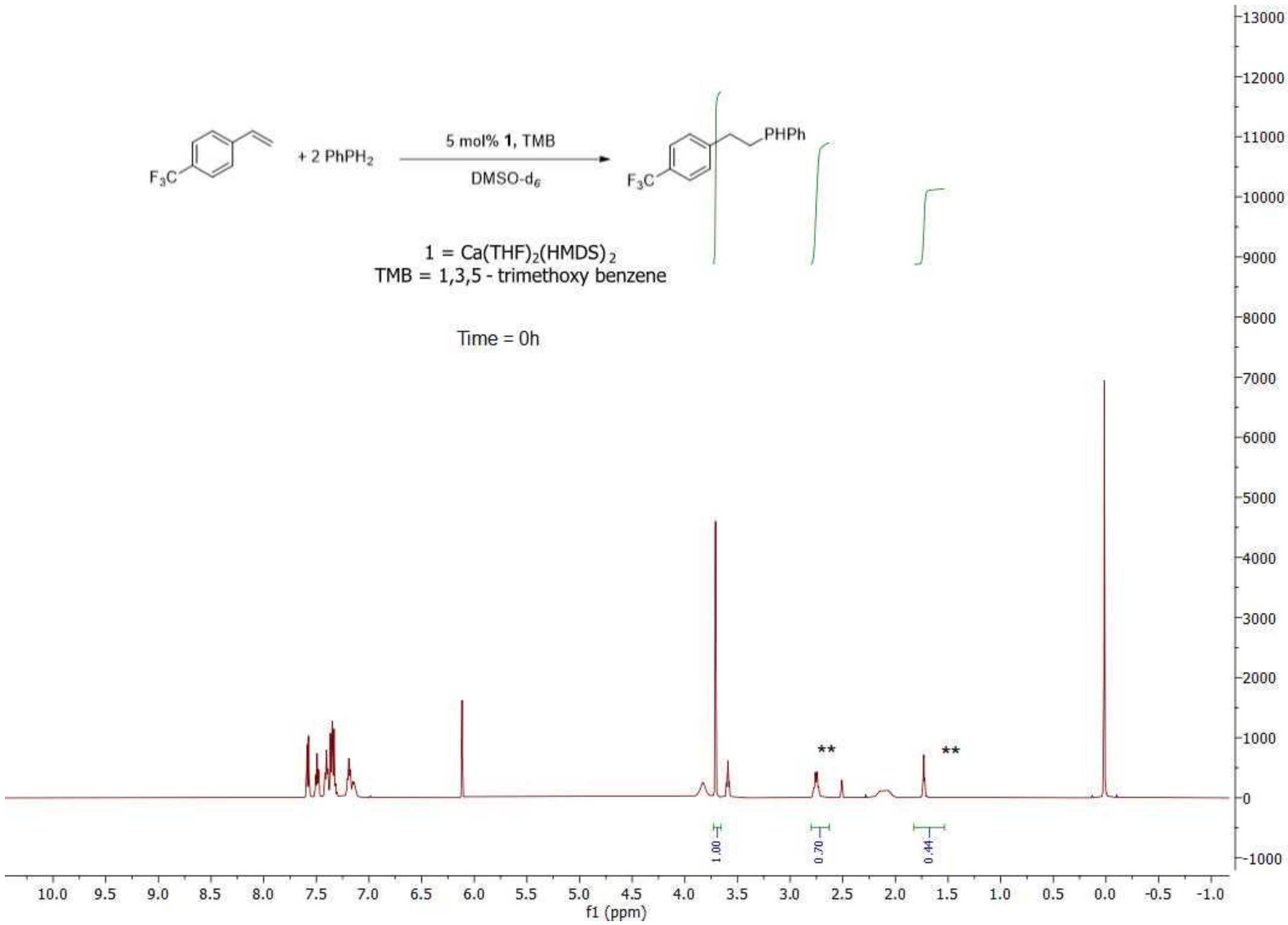

Figure S178: Hydrophosphination of 0.5 mmol 4-trifluoromethylstyrene with 2 equivalents of $PhPH_2$ in DMSO-d_6 using 5 mol% **1** – 0 h – ^{1}H NMR.

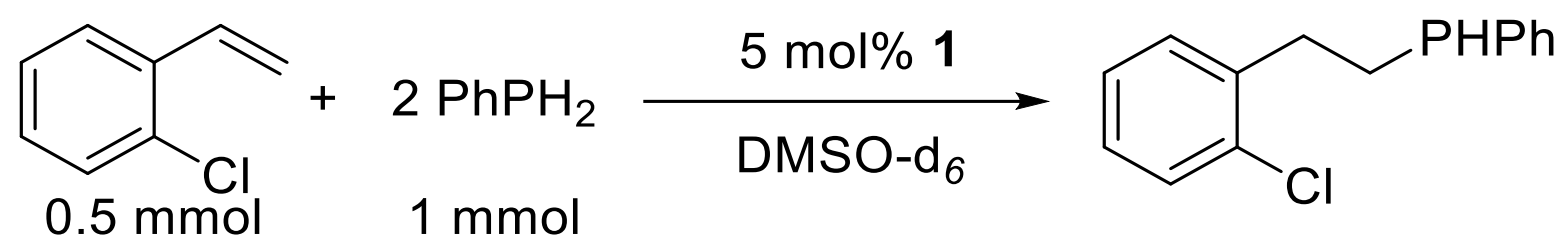

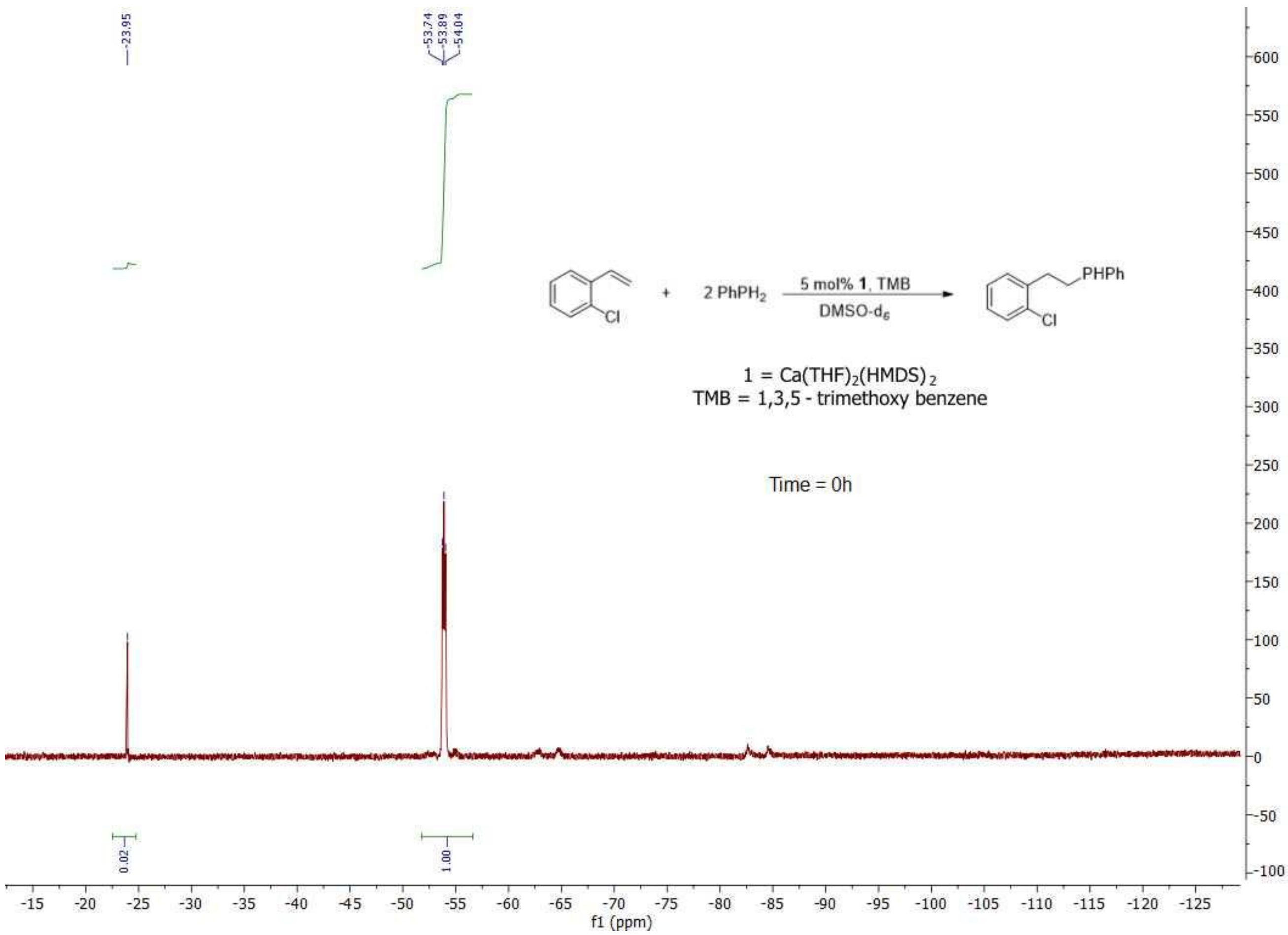

Figure S179: Hydrophosphination of 0.5 mmol 2-chlorostyrene with 2 equivalents of $PhPH_2$ in DMSO-d_6 using 5 mol% **1** – 0 h – $^{31}P\{^1H\}$ NMR.

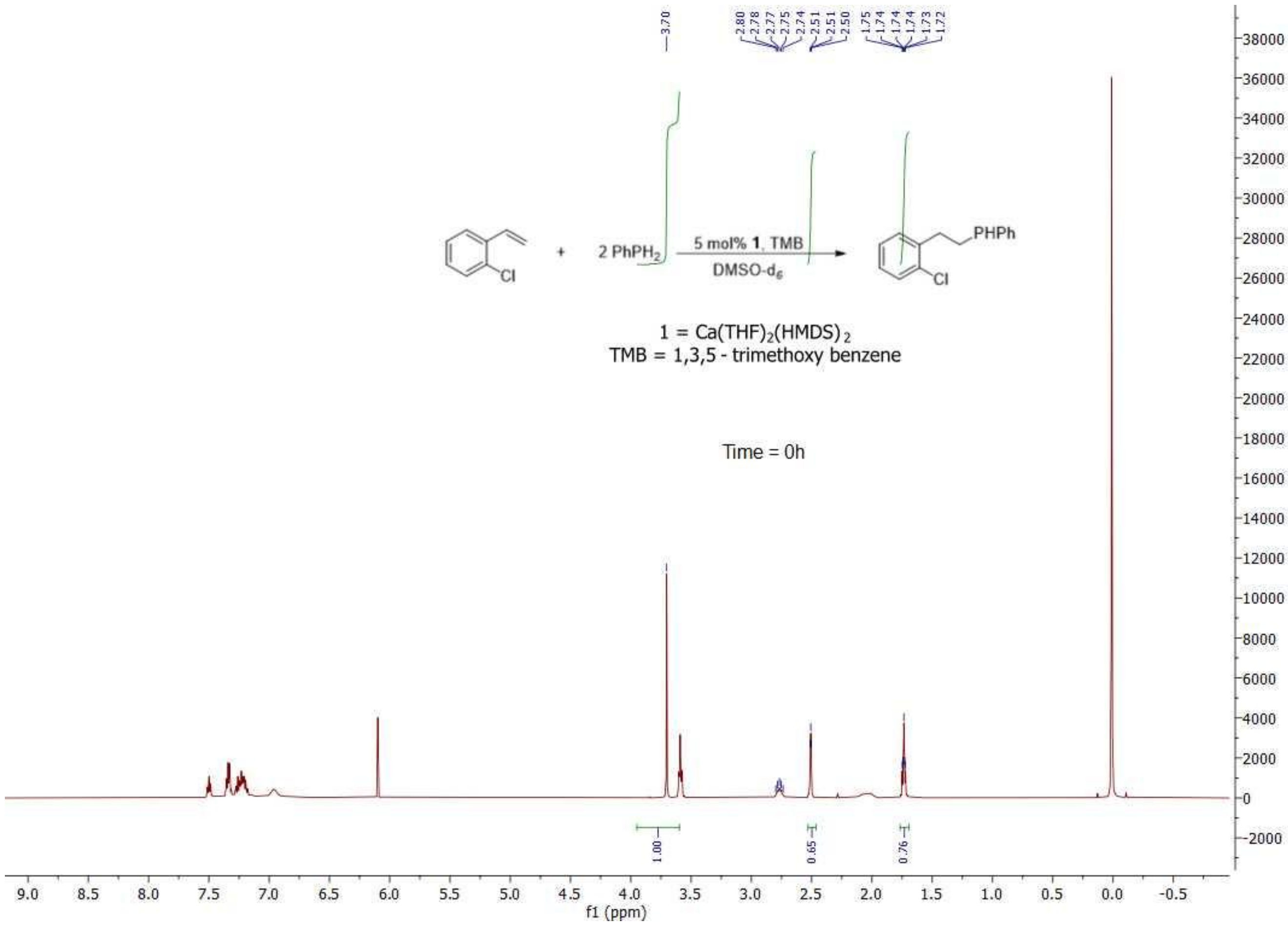

Figure S180: Hydrophosphination of 0.5 mmol 2-chlorostyrene with 2 equivalents of $PhPH_2$ in DMSO-d_6 using 5 mol% **1** – 0 h – ^{1}H NMR.

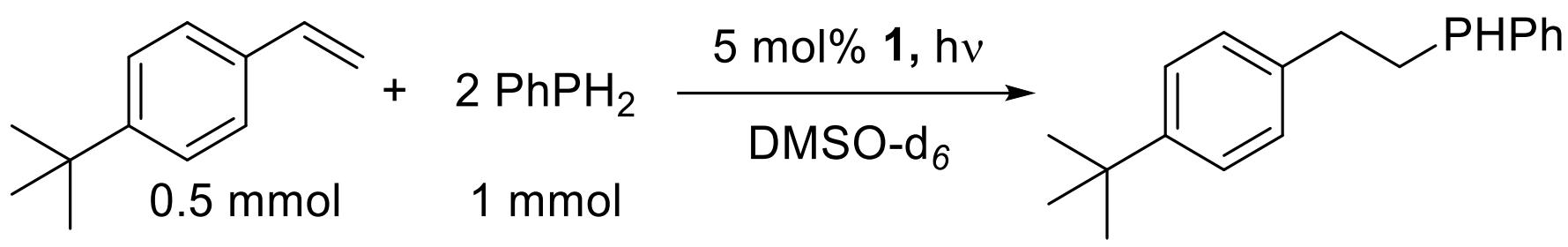

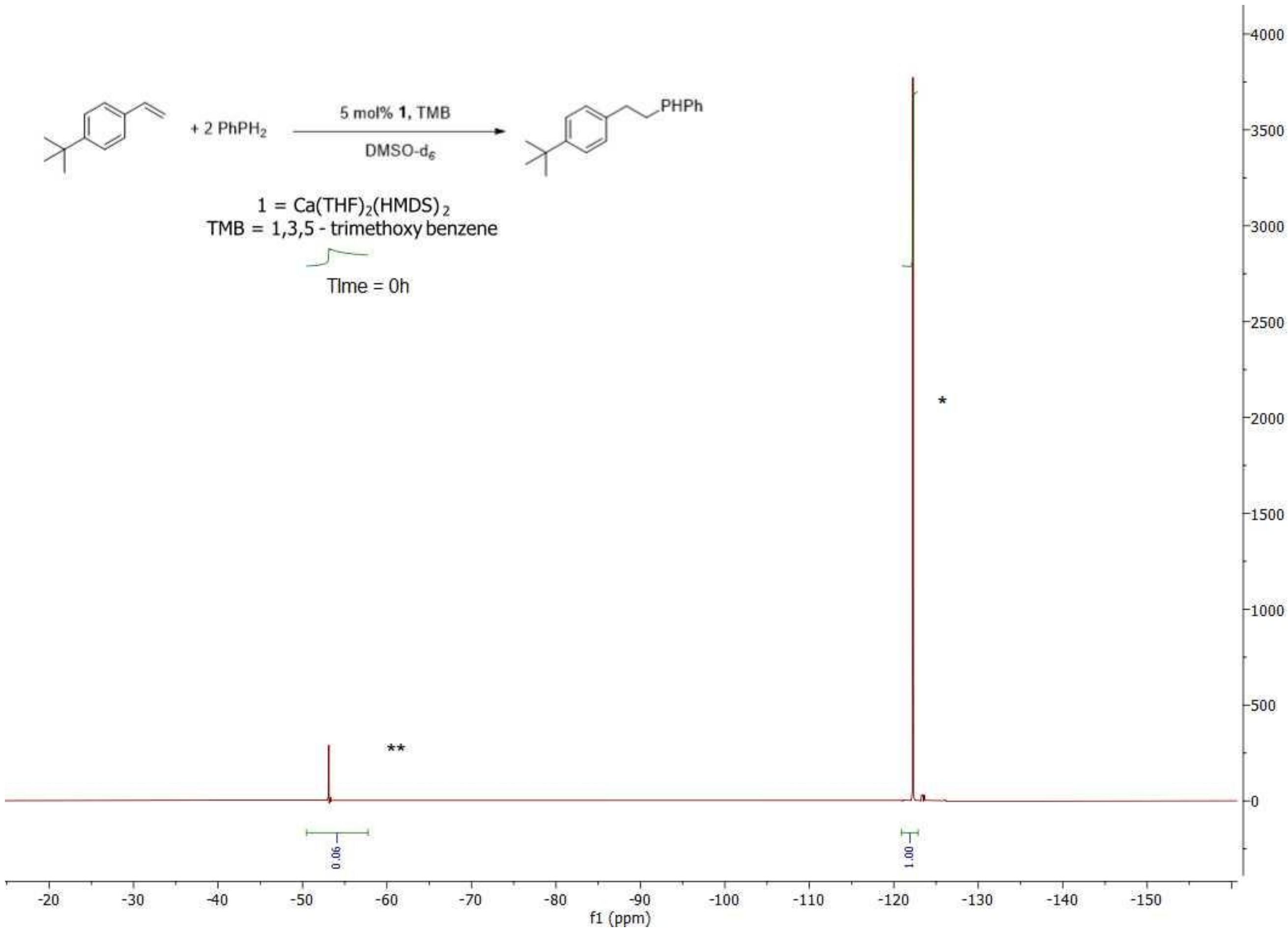

Figure S181: Hydrophosphination of 0.5 mmol 4-tertiarybutyl styrene with 2 equivalents of $PhPH_2$ in DMSO-d_6 using 5 mol% **1** under UV 360 nm irradiation – 0 h – $^{31}P\{^1H\}$ NMR.

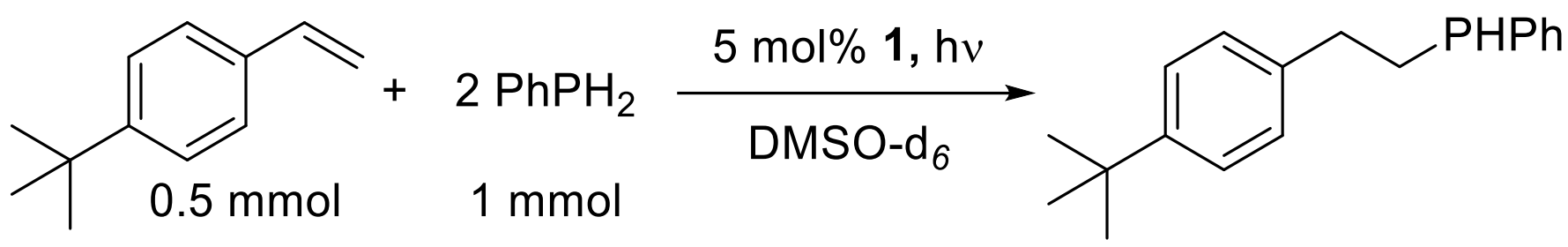

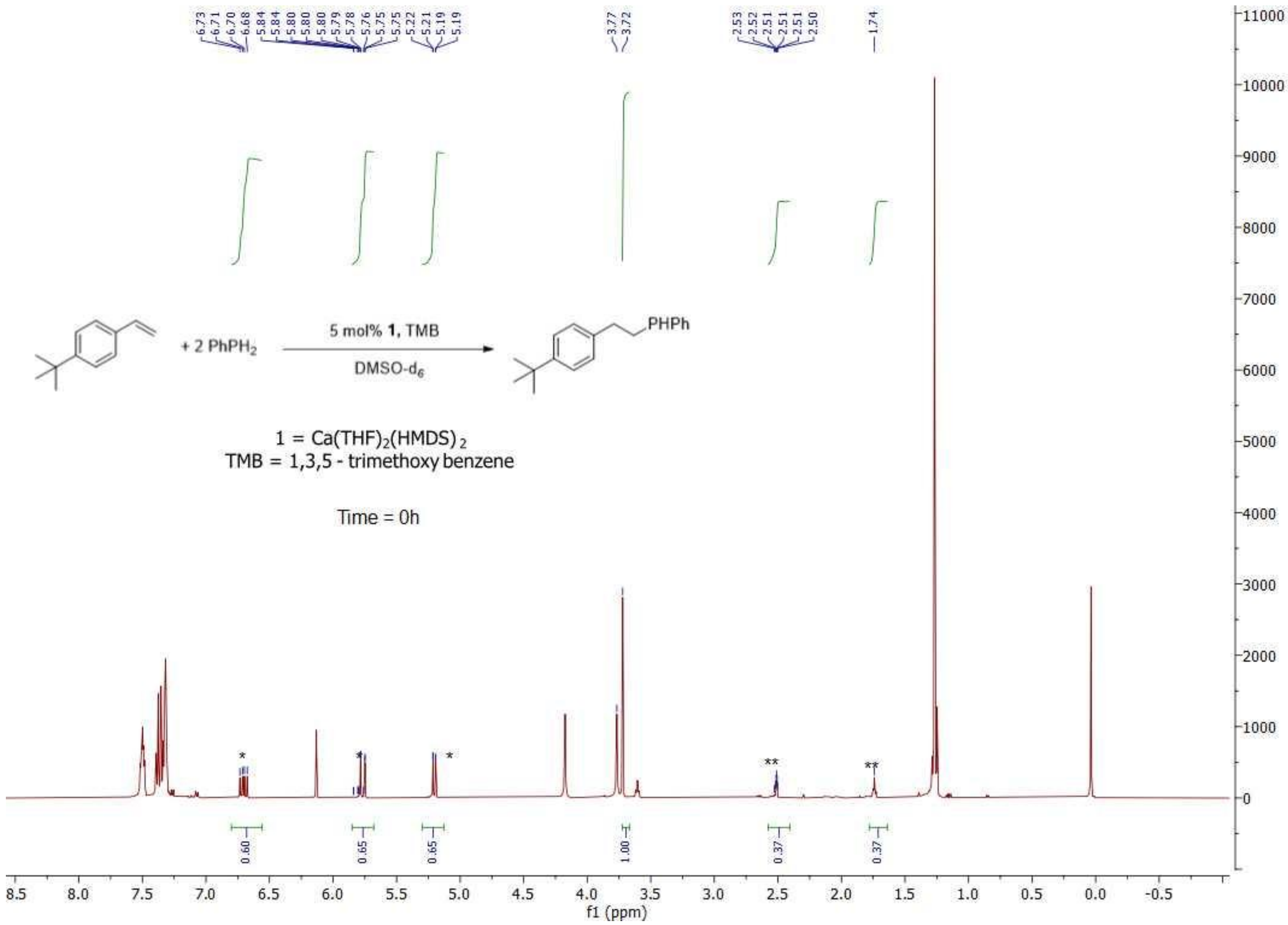

Figure S182: Hydrophosphination of 0.5 mmol 4-tertiarybutyl styrene with 2 equivalents of $PhPH_2$ in DMSO-d_6 using 5 mol% **1** under UV 360 nm irradiation – 0 h – ^{1}H NMR.

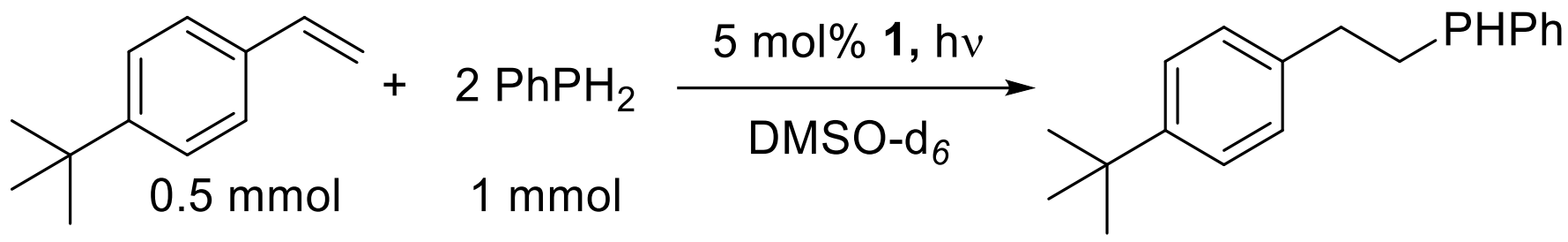

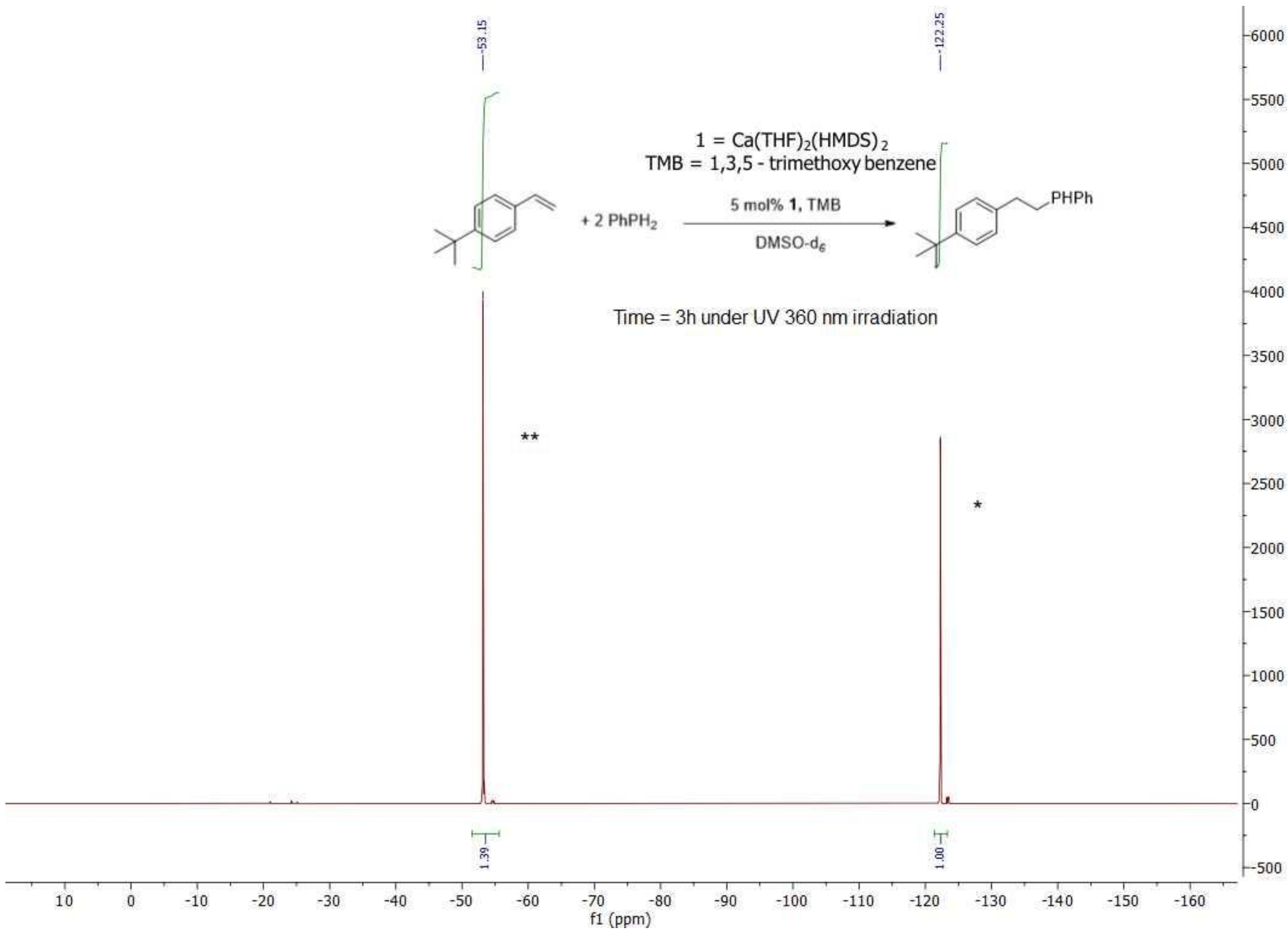

Figure S183: Hydrophosphination of 0.5 mmol 4-tertiarybutyl styrene with 2 equivalents of $PhPH_2$ in DMSO-d_6 using 5 mol% **1** under UV 360 nm irradiation – 3 h – $^{31}P\{^1H\}$ NMR.

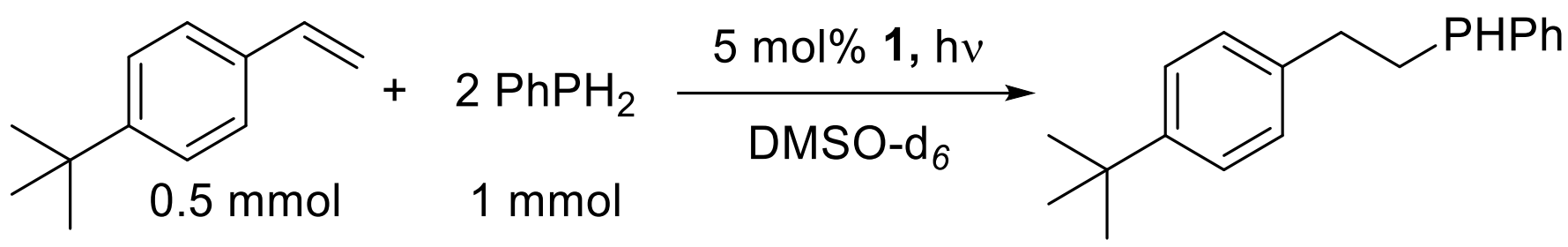

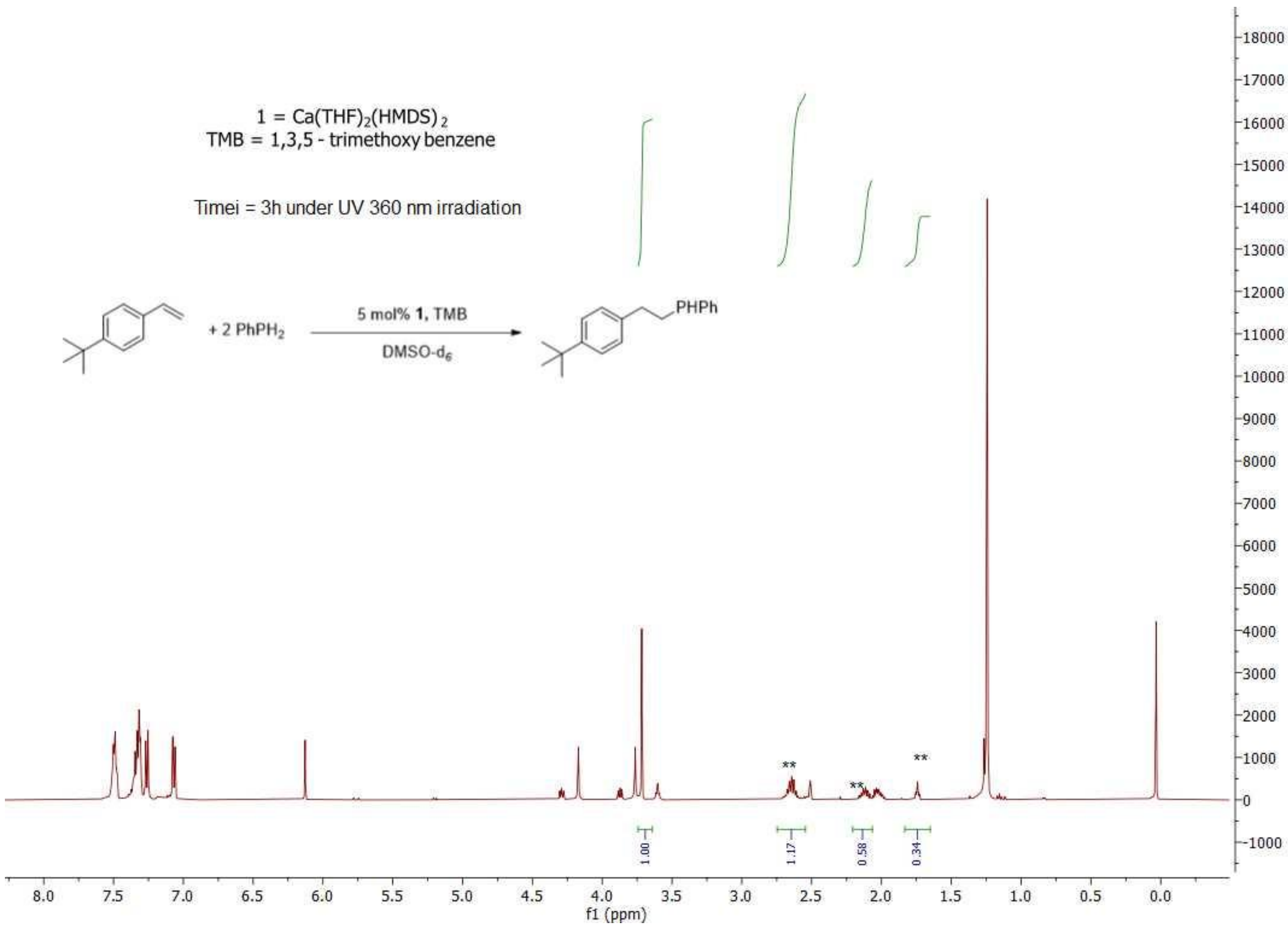

Figure S184: Hydrophosphination of 0.5 mmol 4-tertiarybutyl styrene with 2 equivalents of $PhPH_2$ in DMSO-d_6 using 5 mol% **1** under UV 360 nm irradiation – 3 h – ^{1}H NMR.

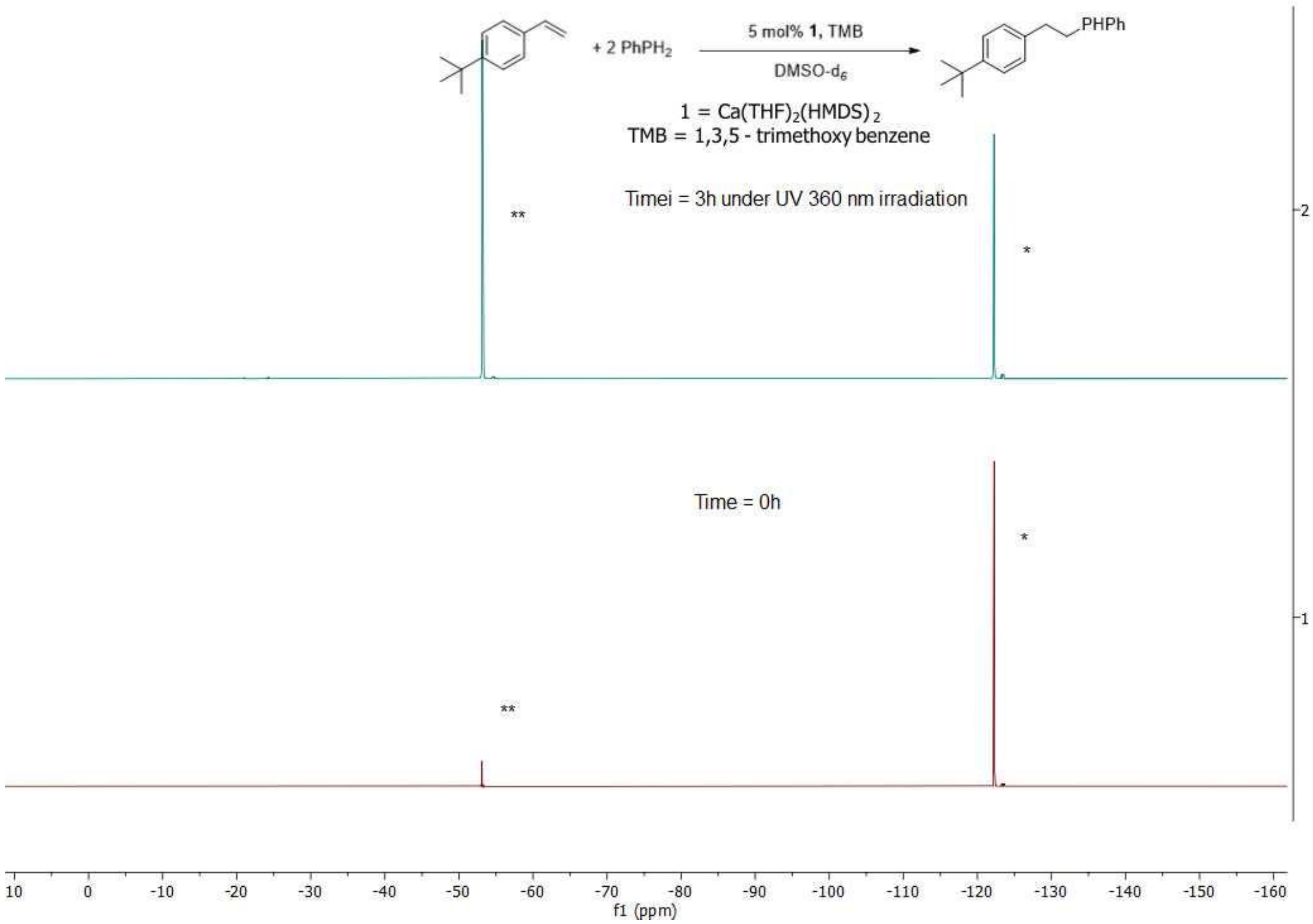

Figure S185: Hydrophosphination of 0.5 mmol 4-tertiarybutyl styrene with 2 equivalents of $PhPH_2$ in DMSO-d_6 using 5 mol% **1** under UV 360 nm irradiation - stacked $^{31}P\{^1H\}$ NMR.

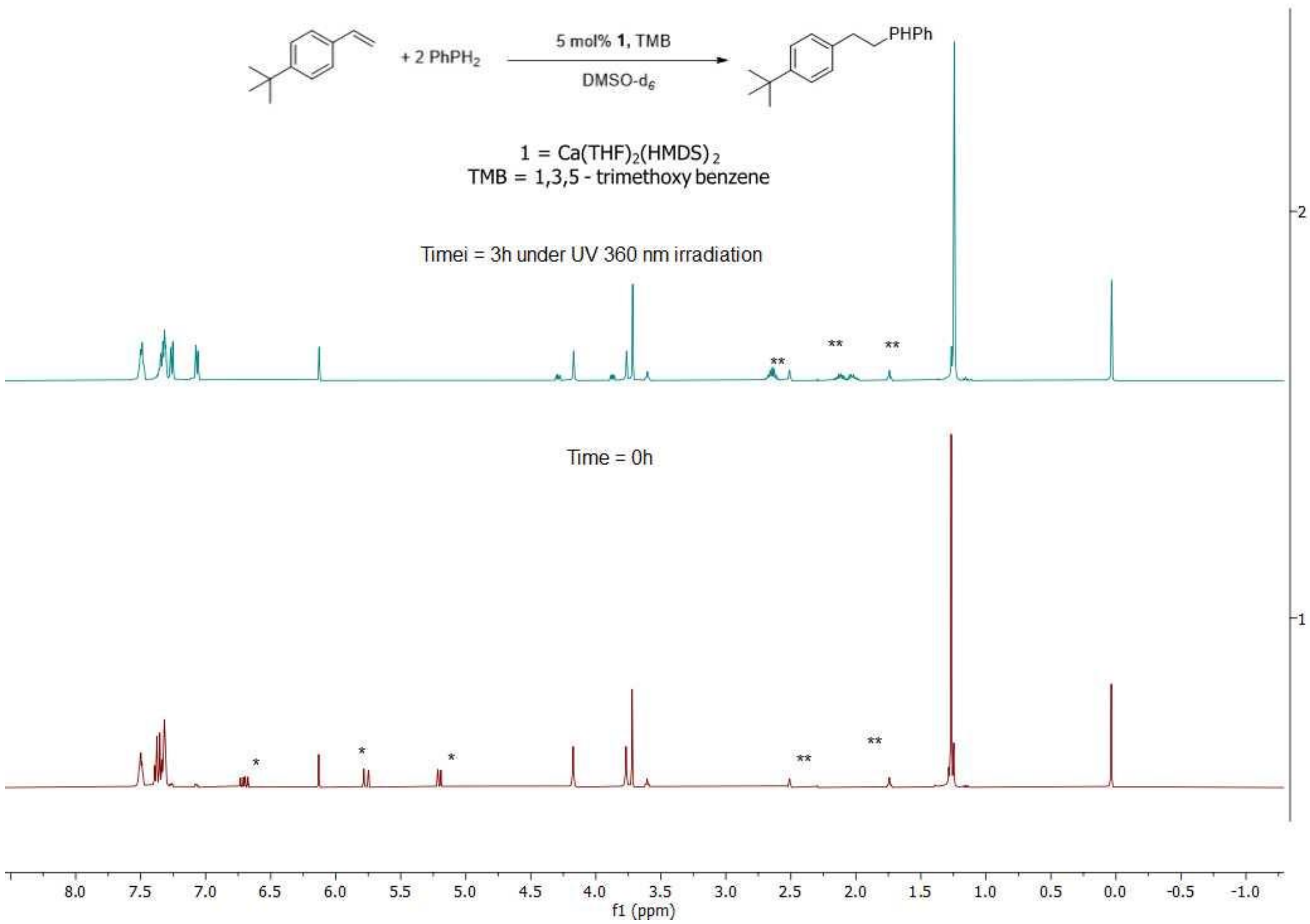

Figure S186: Hydrophosphination of 0.5 mmol 4-tertiarybutyl styrene with 2 equivalents of $PhPH_2$ in DMSO-d_6 using 5 mol% **1** under UV 360 nm irradiation – stacked ^{1}H NMR.

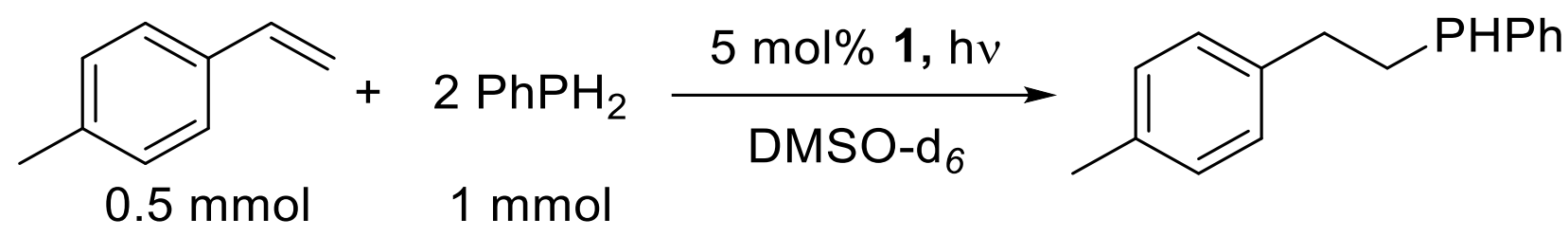

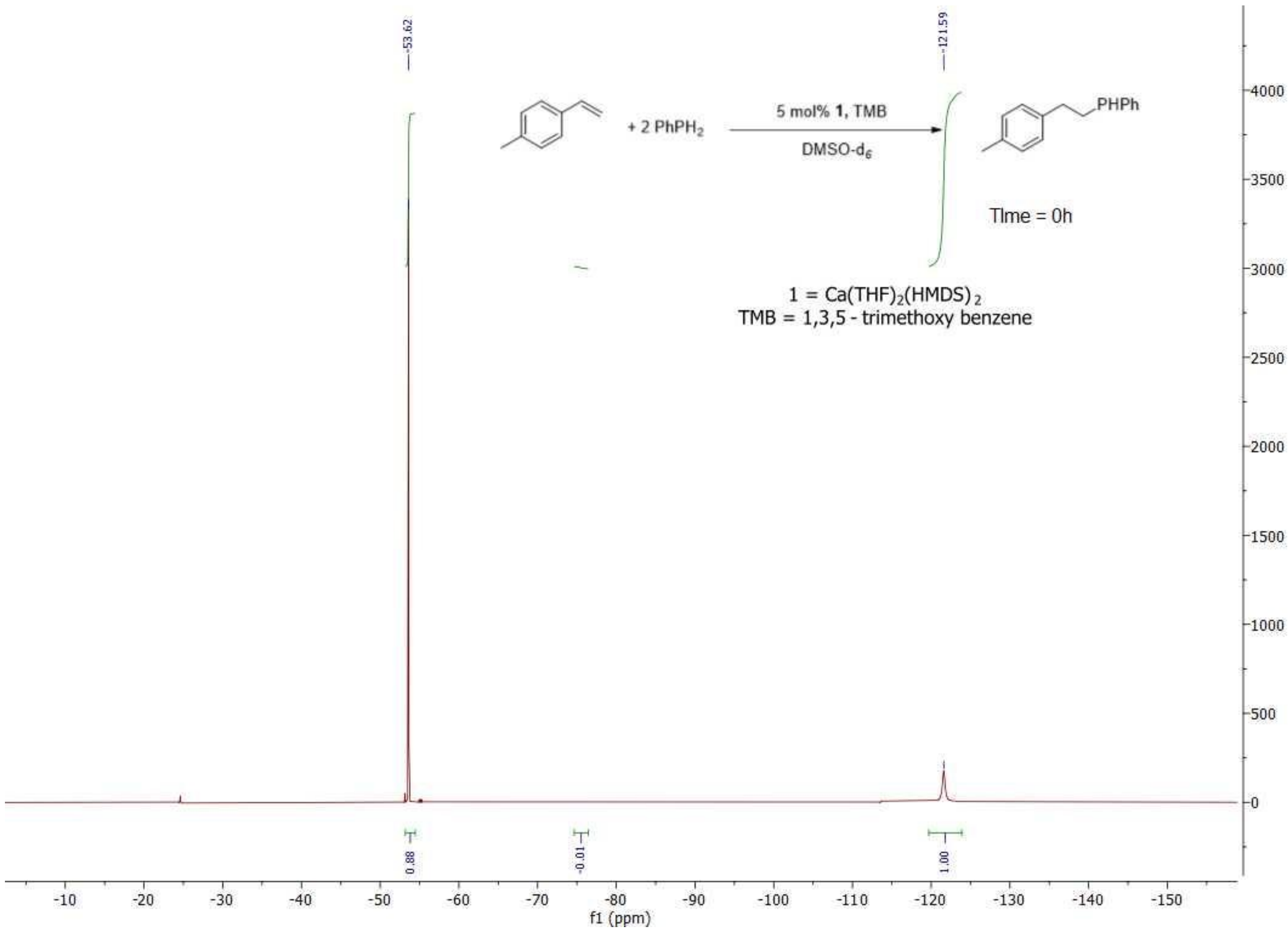

Figure S187: Hydrophosphination of 0.5 mmol 4-methyl styrene with 2 equivalents of $PhPH_2$ in DMSO-d_6 using 5 mol% **1** under UV 360 nm irradiation -0 h– $^{31}P\{^1H\}$ NMR.

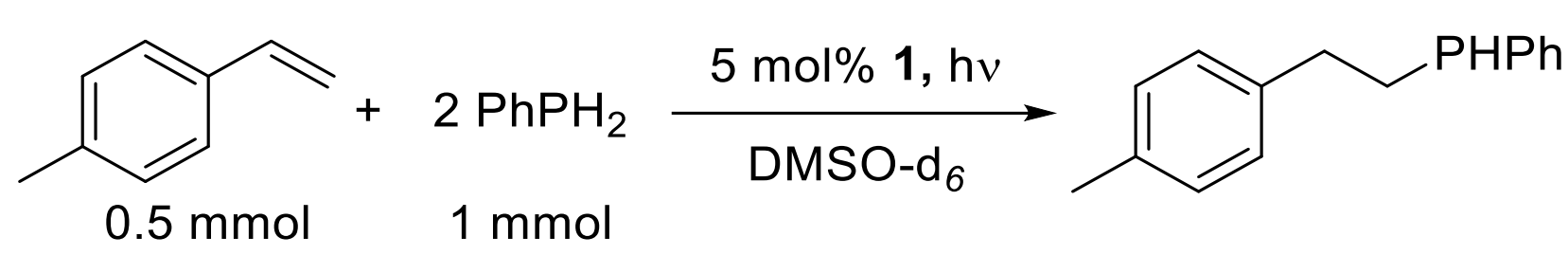

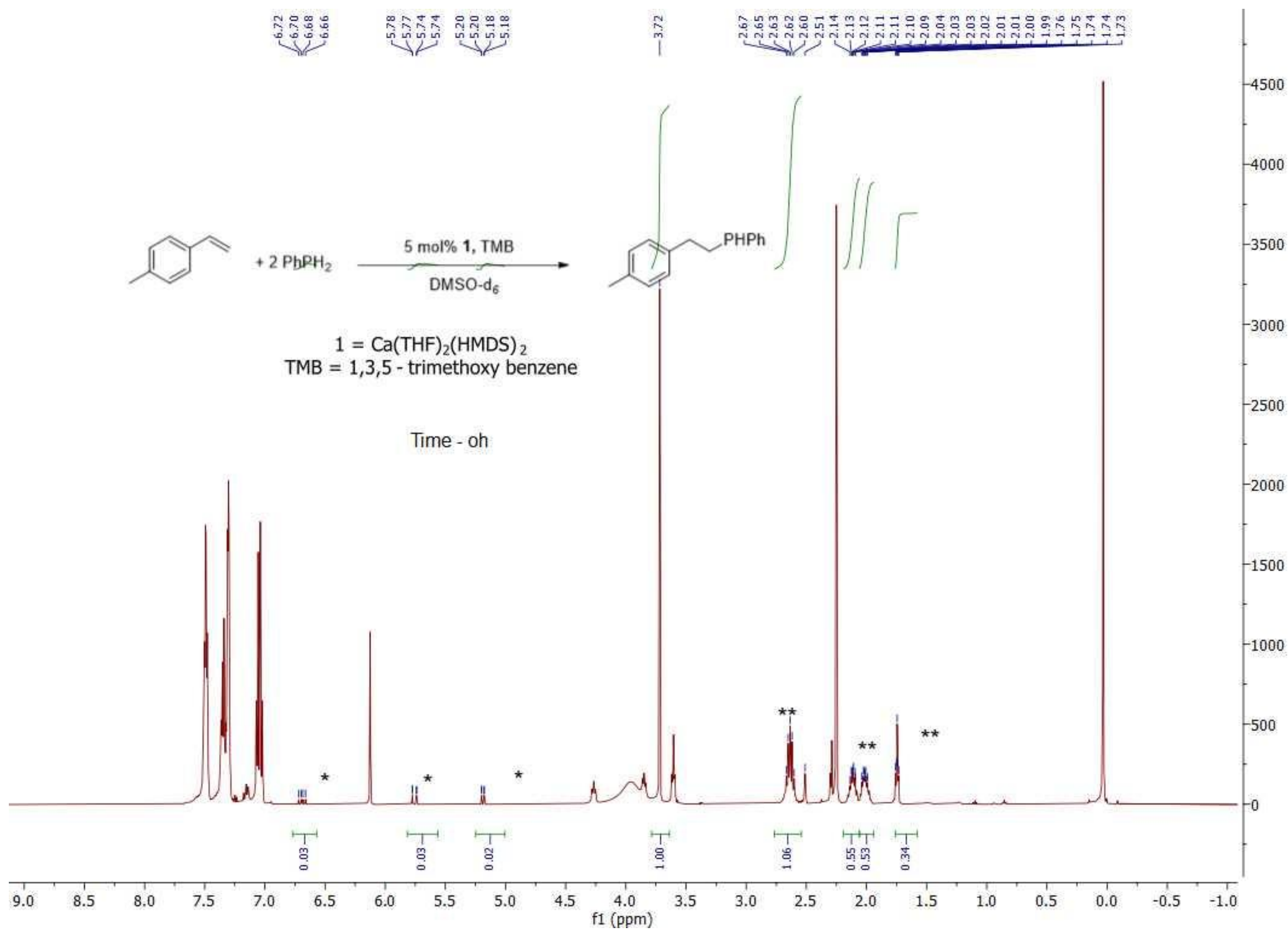

Figure S188: Hydrophosphination of 0.5 mmol 4-methyl styrene with 2 equivalents of $PhPH_2$ in DMSO-d_6 using 5 mol% **1** under UV 360 nm irradiation - 0 h – ^{1}H NMR.

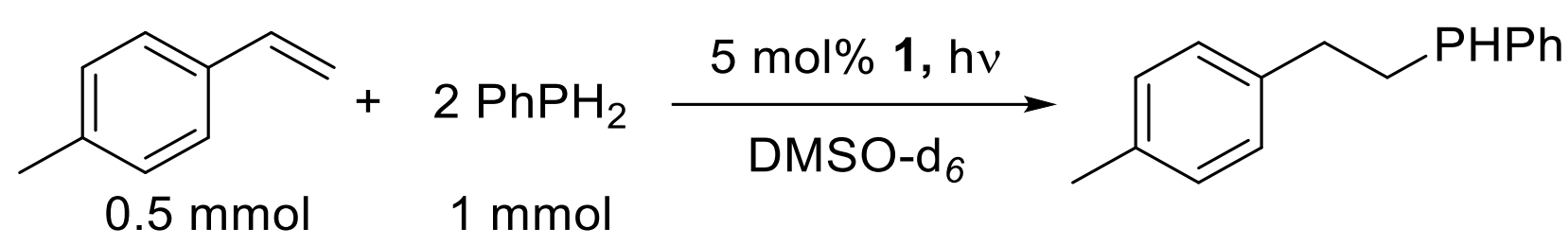

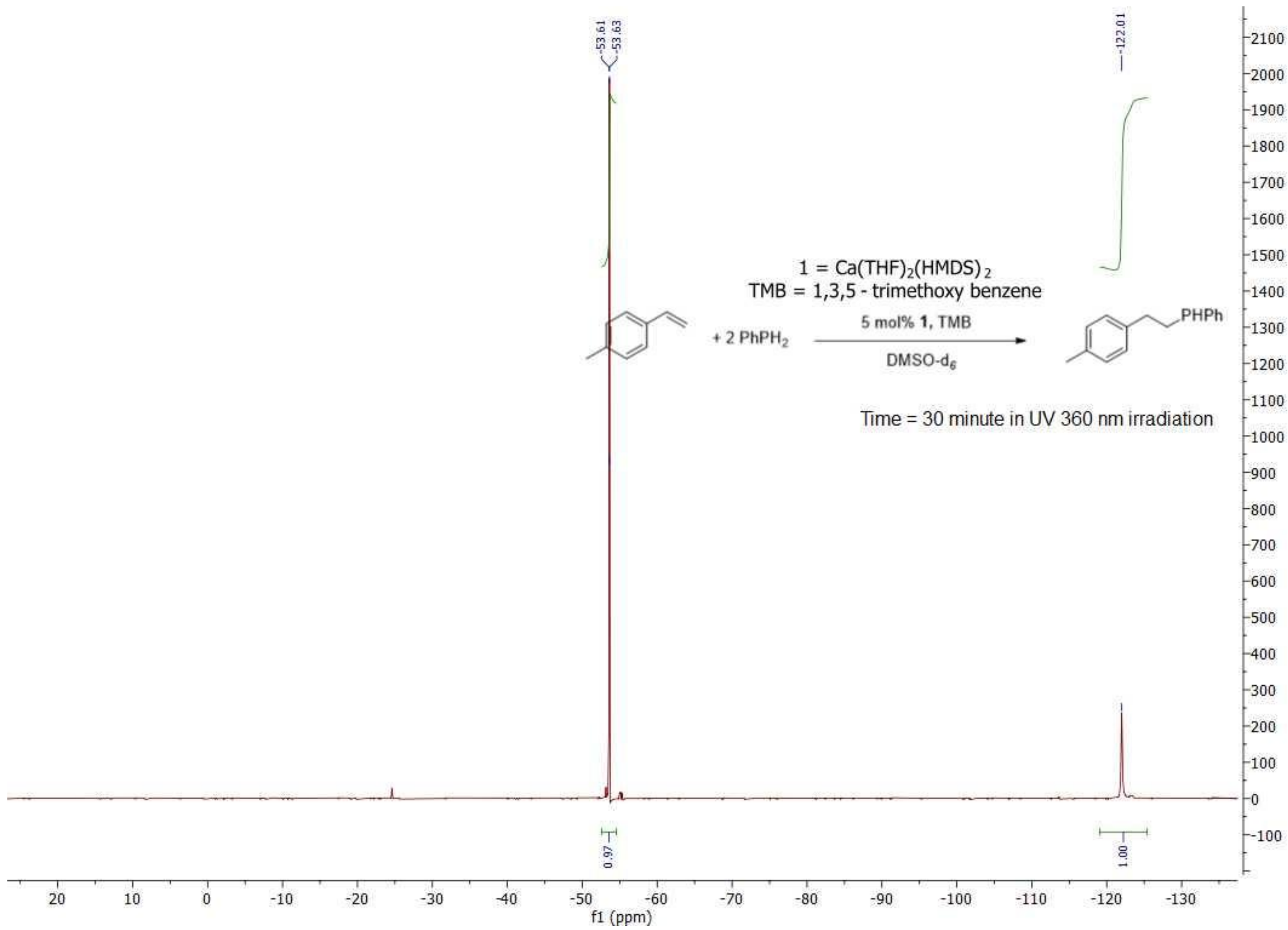

Figure S189: Hydrophosphination of 0.5 mmol 4-methyl styrene with 2 equivalents of $PhPH_2$ in DMSO-d_6 using 5 mol% **1** under UV 360 nm irradiation - 0.5 h – $^{31}P\{^1H\}$ NMR.

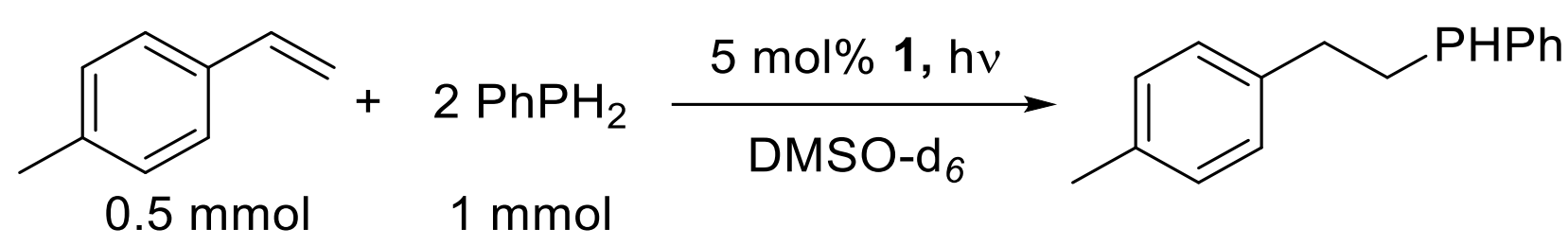

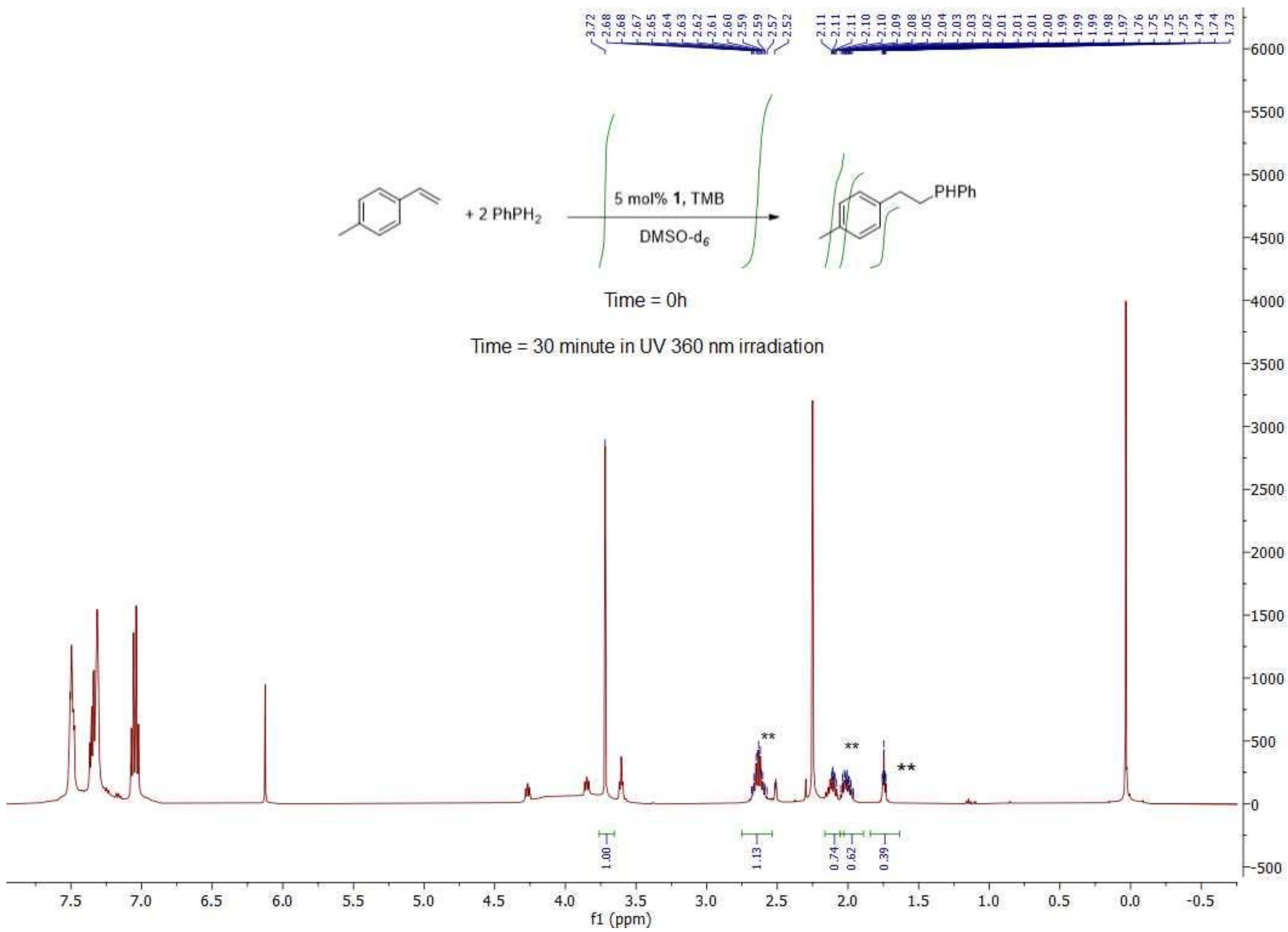

Figure S190: Hydrophosphination of 0.5 mmol 4-methyl styrene with 2 equivalents of $PhPH_2$ in DMSO-d_6 using 5 mol% **1** under UV 360 nm irradiation - 0.5 h– ^1H NMR.

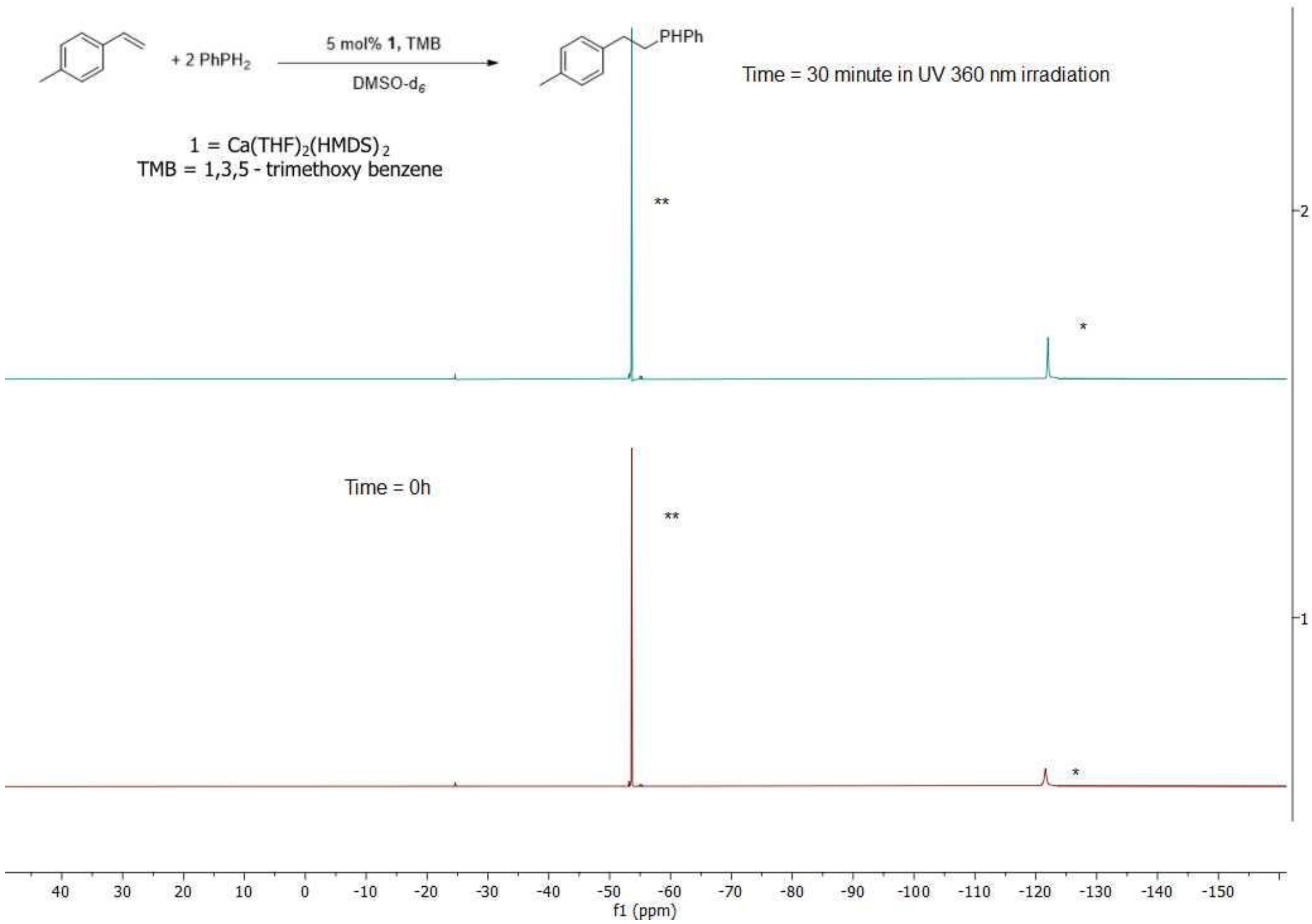

Figure S191: Hydrophosphination of 0.5 mmol 4-methyl styrene with 2 equivalents of $PhPH_2$ in DMSO-d_6 using 5 mol% **1** under UV 360 nm irradiation – stacked - $^{31}P\{^1H\}$ NMR.

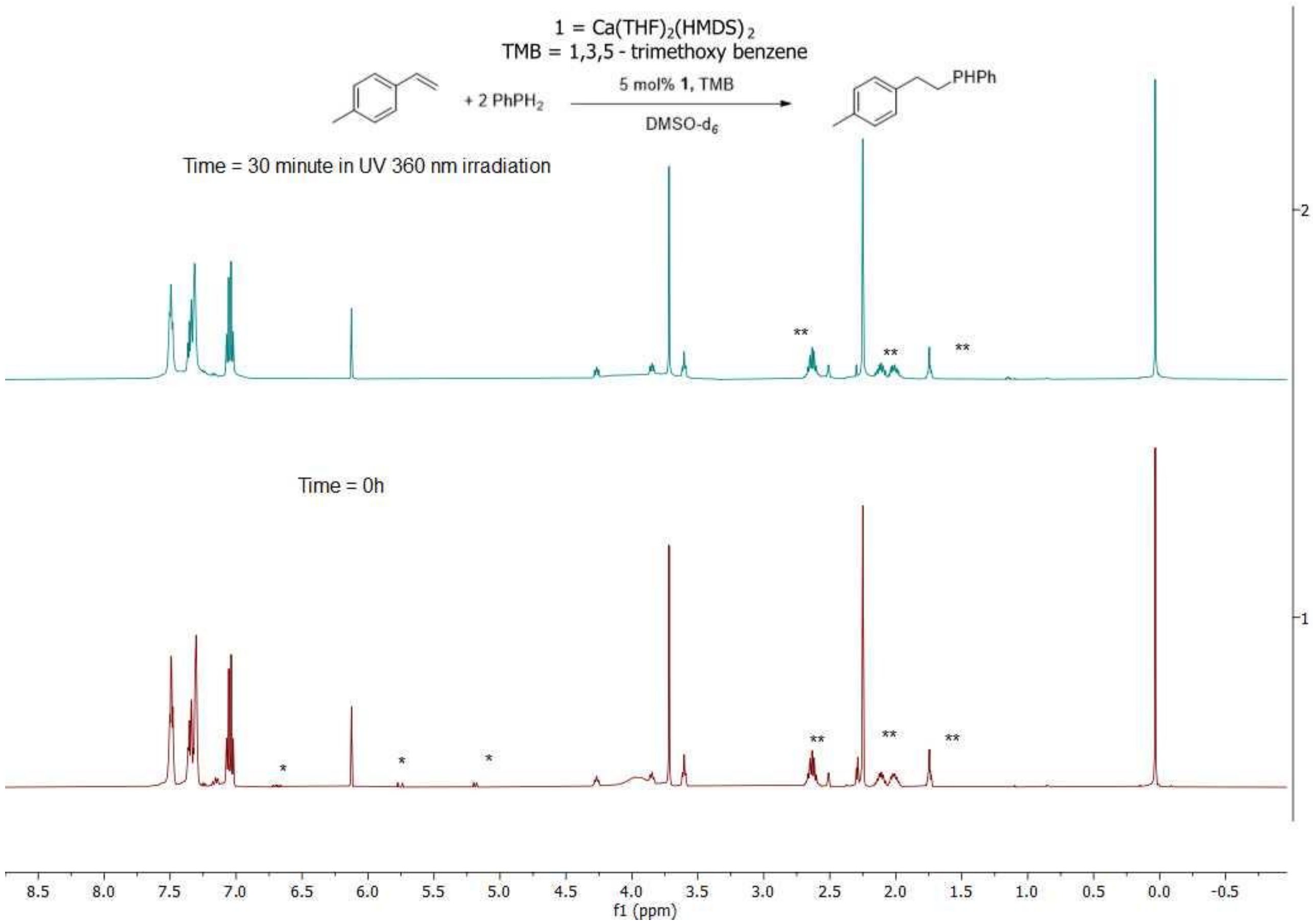

Figure S192: Hydrophosphination of 0.5 mmol 4-methyl styrene with 2 equivalents of $PhPH_2$ in DMSO-d_6 using 5 mol% **1** under UV 360 nm irradiation - stacked ^1H NMR.

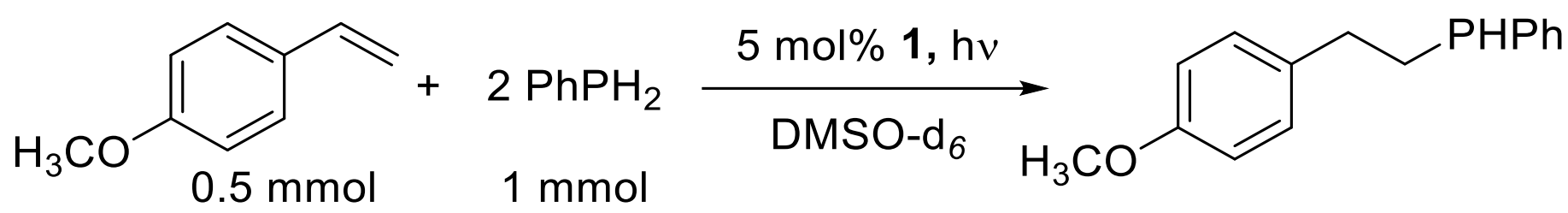

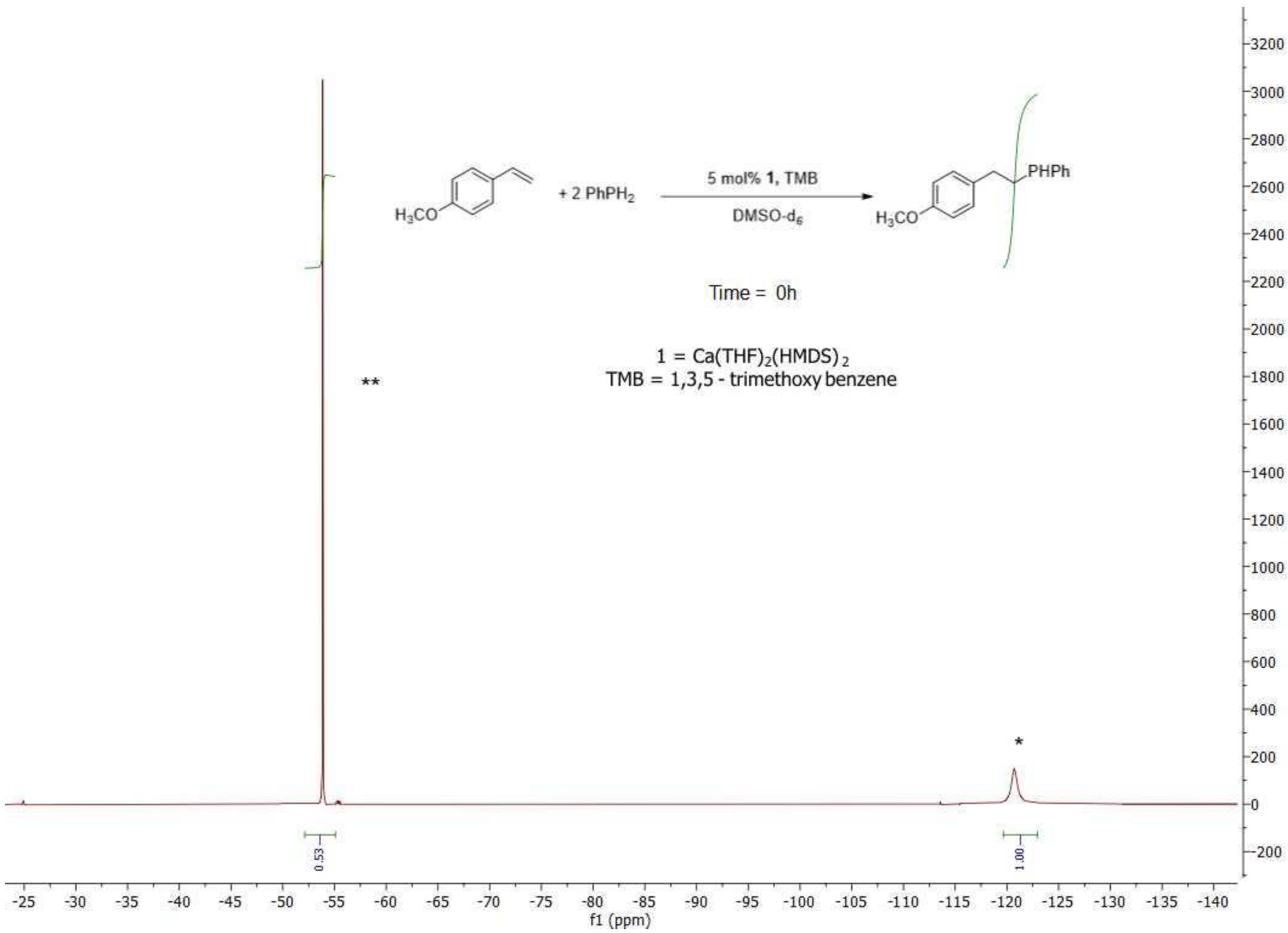

Figure S193: Hydrophosphination of 0.5 mmol 4-methoxy styrene with 2 equivalents of $PhPH_2$ in DMSO-d_6 using 5 mol% **1** under UV 360 nm irradiation – 0 h -$^{31}P\{^1H\}$ NMR.

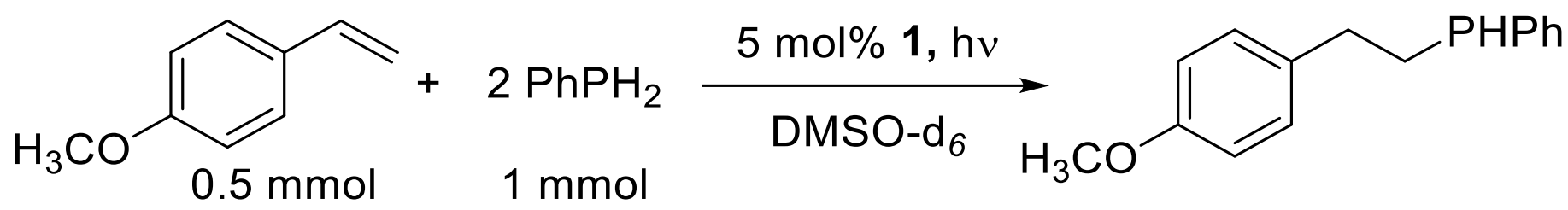

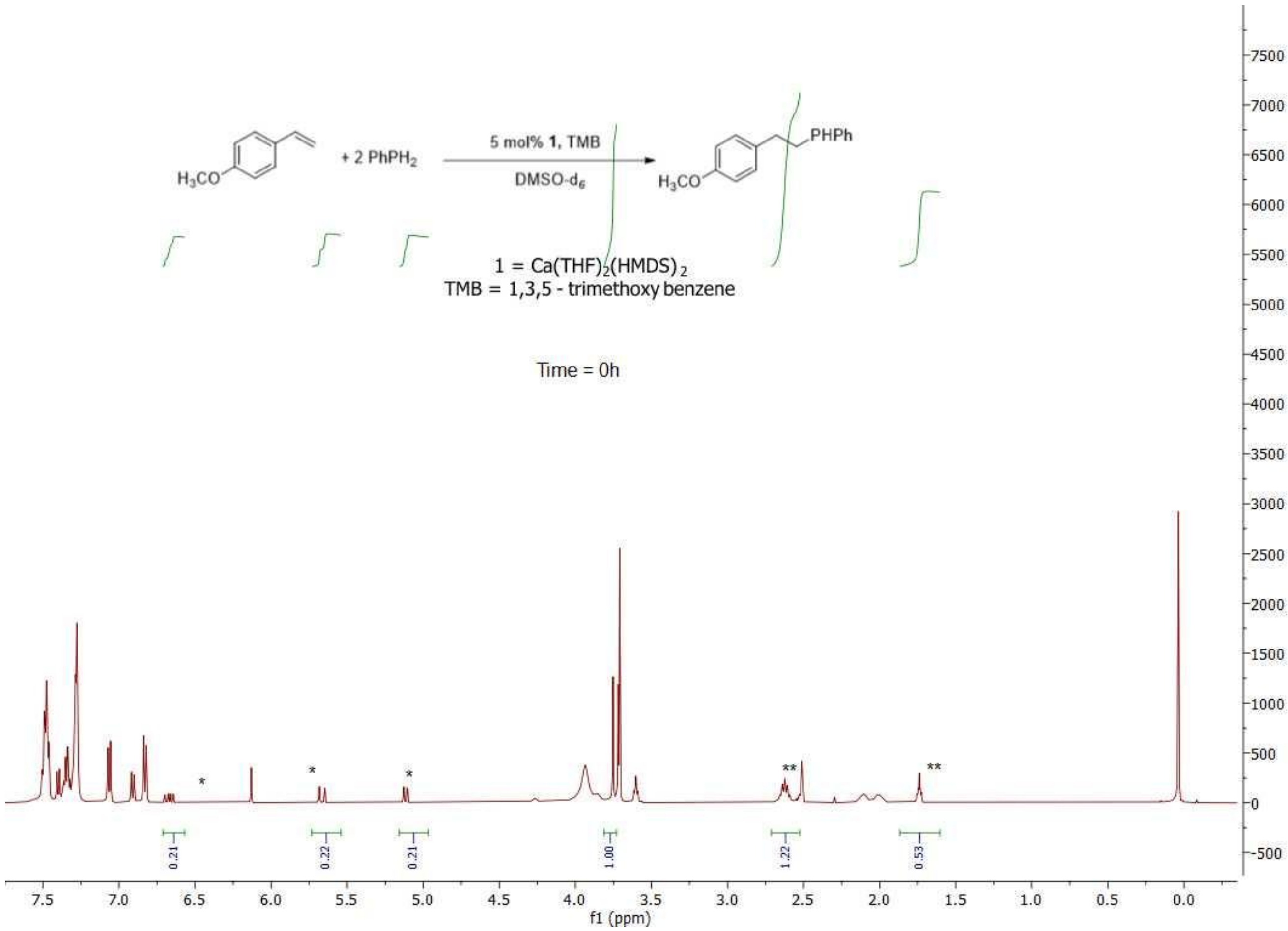

Figure S194: Hydrophosphination of 0.5 mmol 4-methoxy styrene with 2 equivalents of $PhPH_2$ in DMSO-d_6 using 5 mol% **1** under UV 360 nm irradiation – 0 h - ^1H NMR.

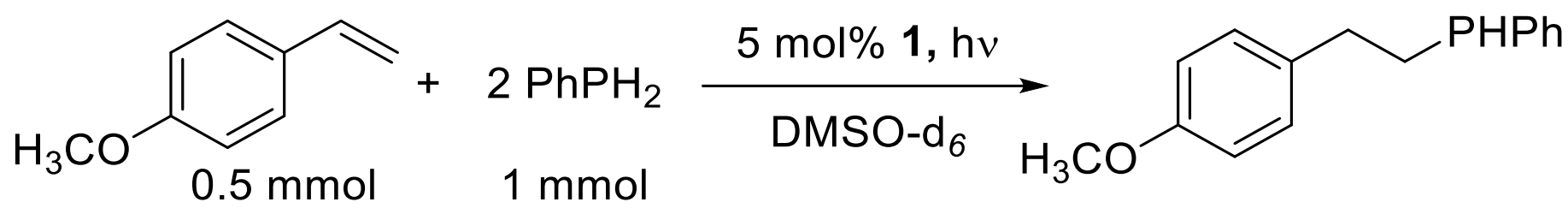

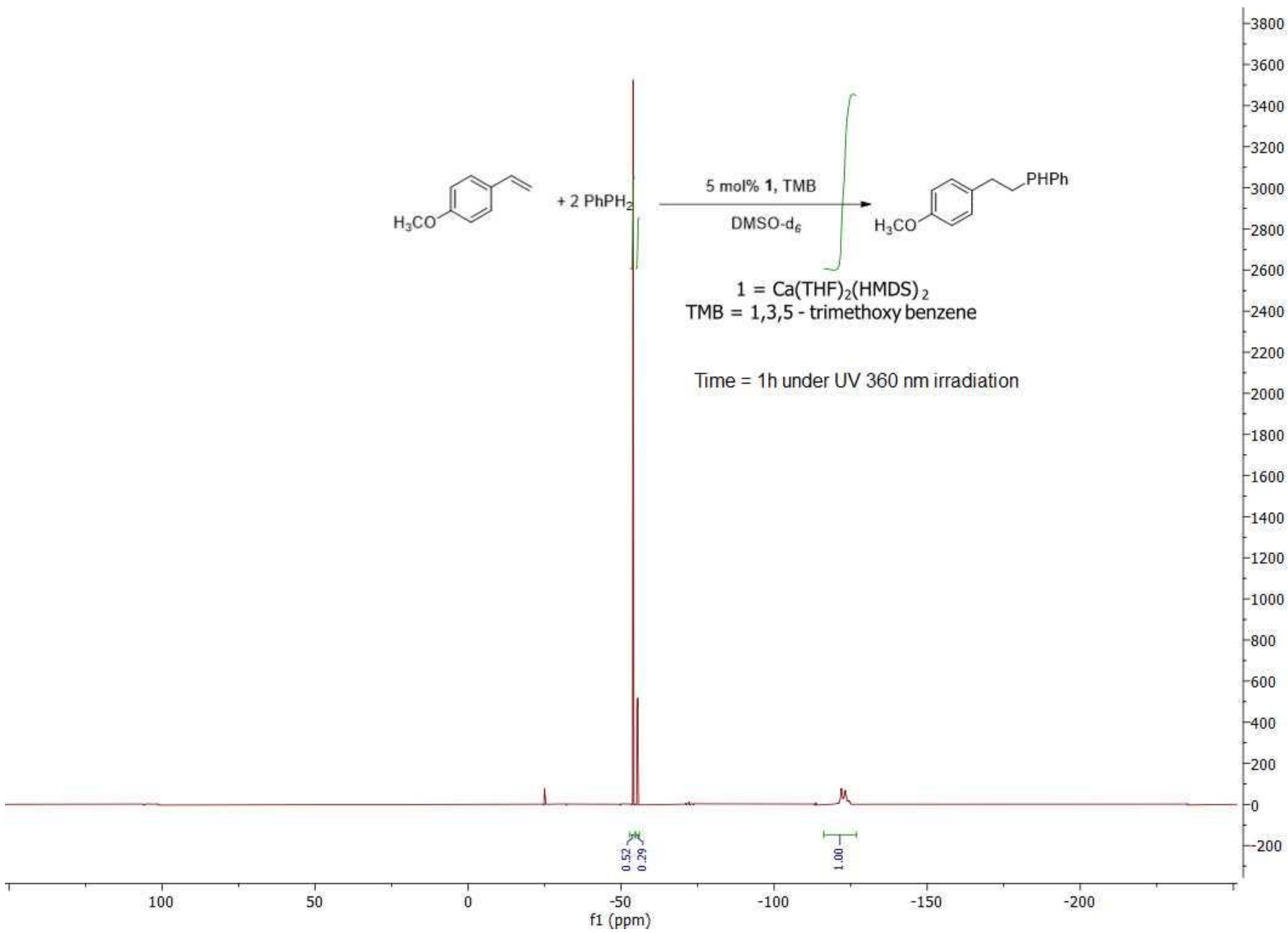

Figure S195: Hydrophosphination of 0.5 mmol 4-methoxy styrene with 2 equivalents of $PhPH_2$ in DMSO-d_6 using 5 mol% **1** under UV 360 nm irradiation – 1 h - $^{31}P\{^1H\}$ NMR.

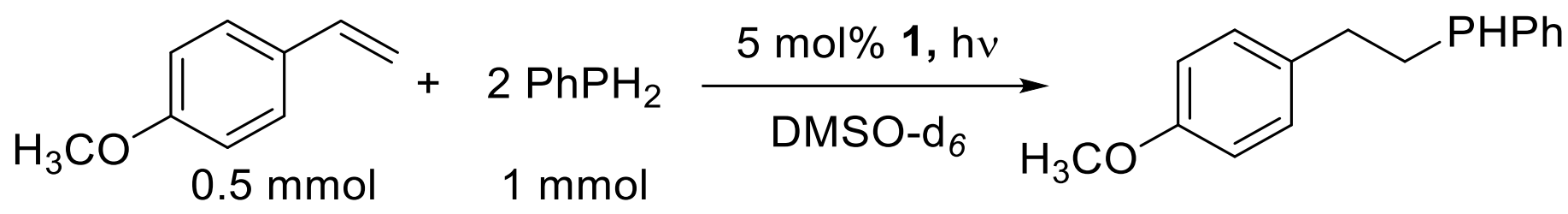

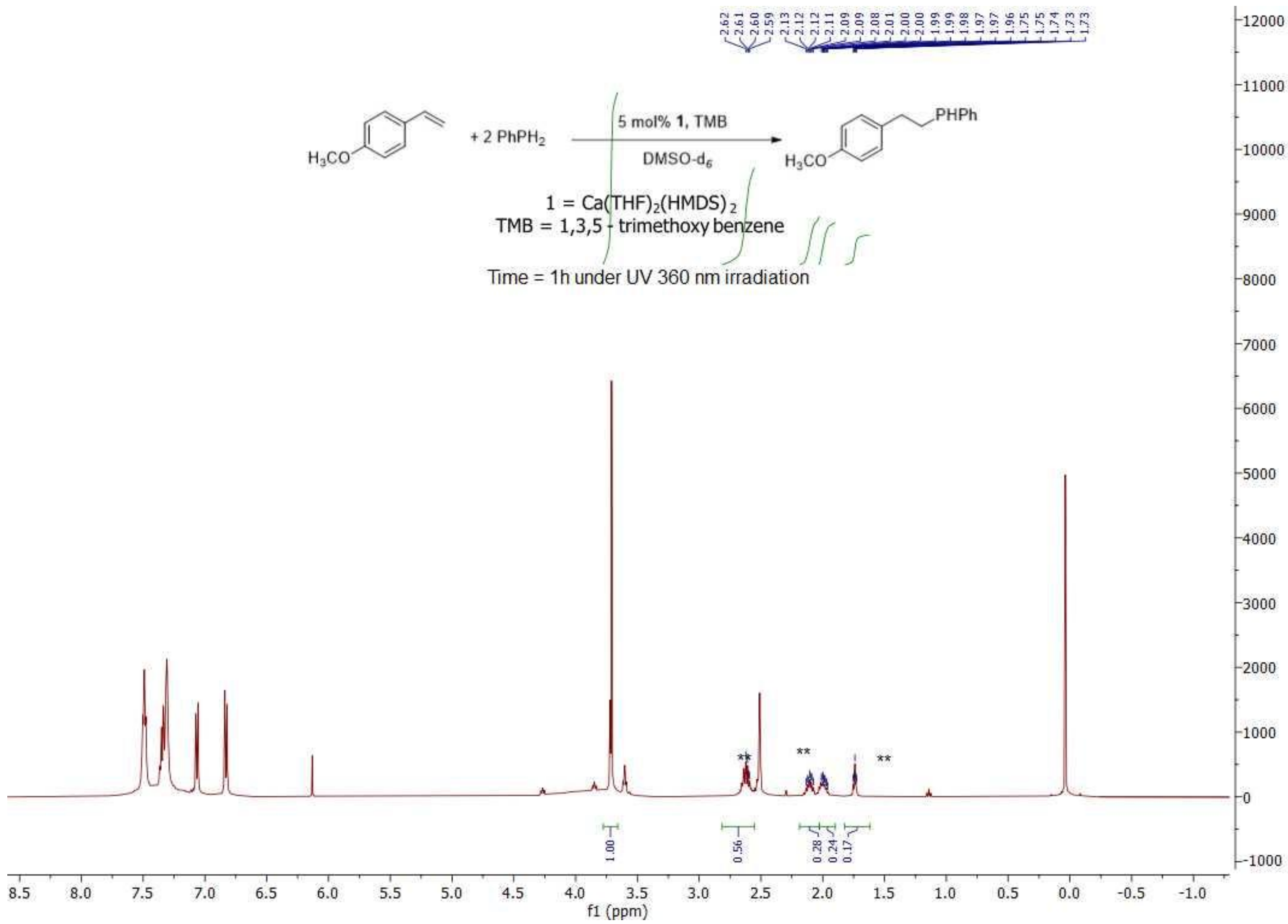

Figure S196: Hydrophosphination of 0.5 mmol 4-methoxy styrene with 2 equivalents of $PhPH_2$ in DMSO-d_6 using 5 mol% **1** under UV 360 nm irradiation – 1 h -^{1}H NMR.

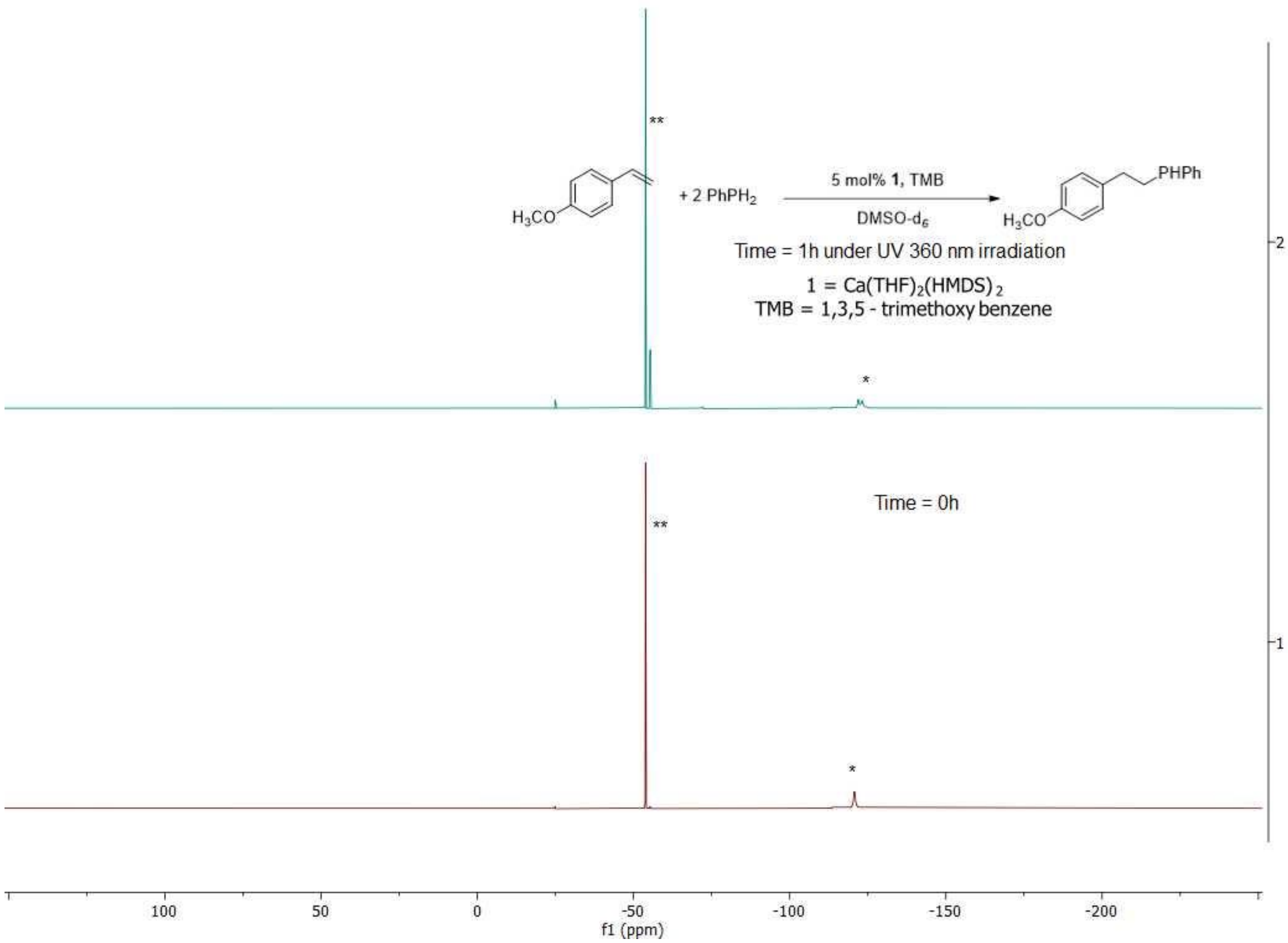

Figure S197: Hydrophosphination of 0.5 mmol 4-methoxy styrene with 2 equivalents of $PhPH_2$ in DMSO-d_6 using 5 mol% **1** under UV 360 nm irradiation stacked - $^{31}P\{^1H\}$ NMR.

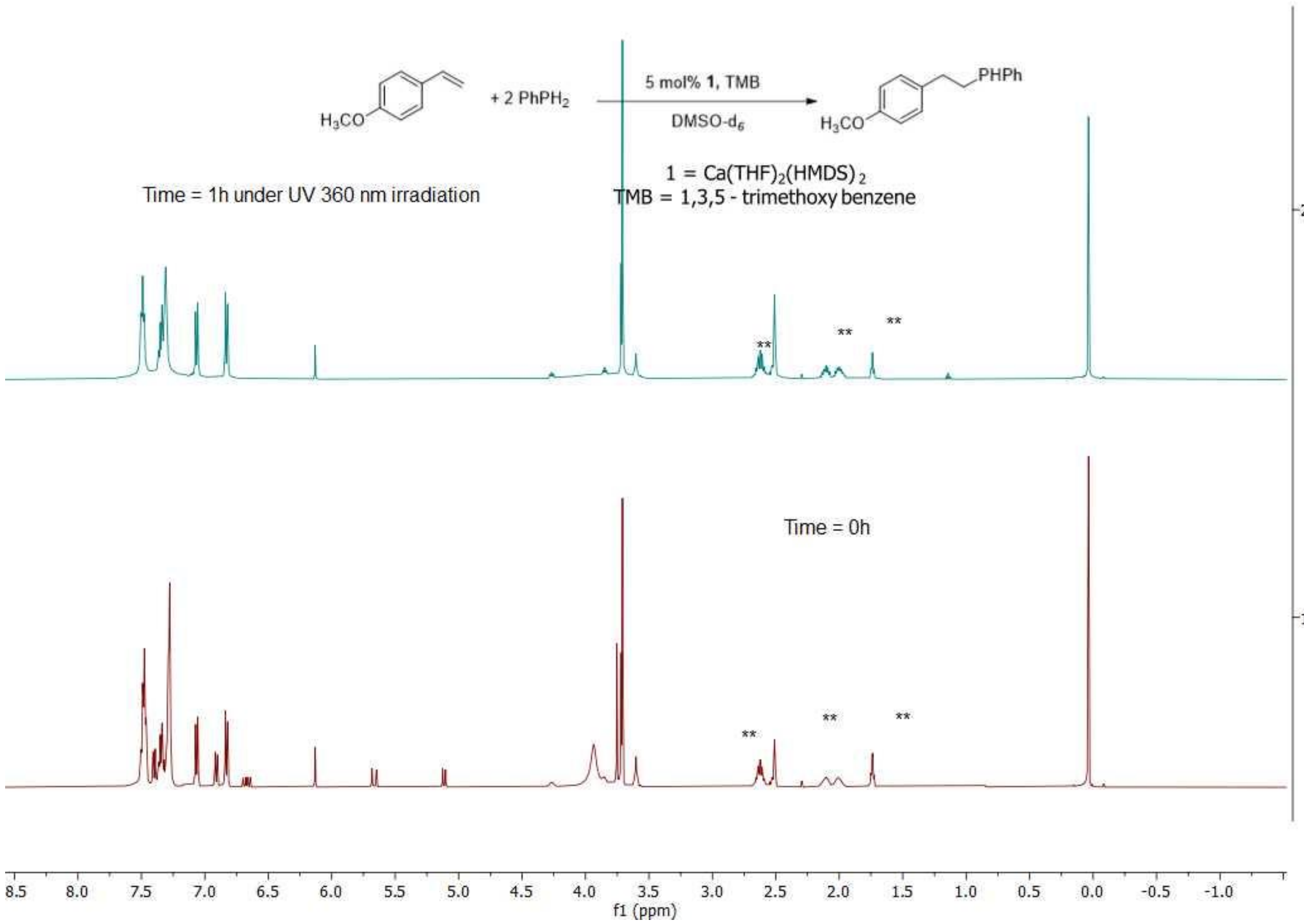

Figure S198: Hydrophosphination of 0.5 mmol 4-methoxy styrene with 2 equivalents of $PhPH_2$ in DMSO-d_6 using 5 mol% **1** under UV 360 nm irradiation – stacked ^{1}H NMR.

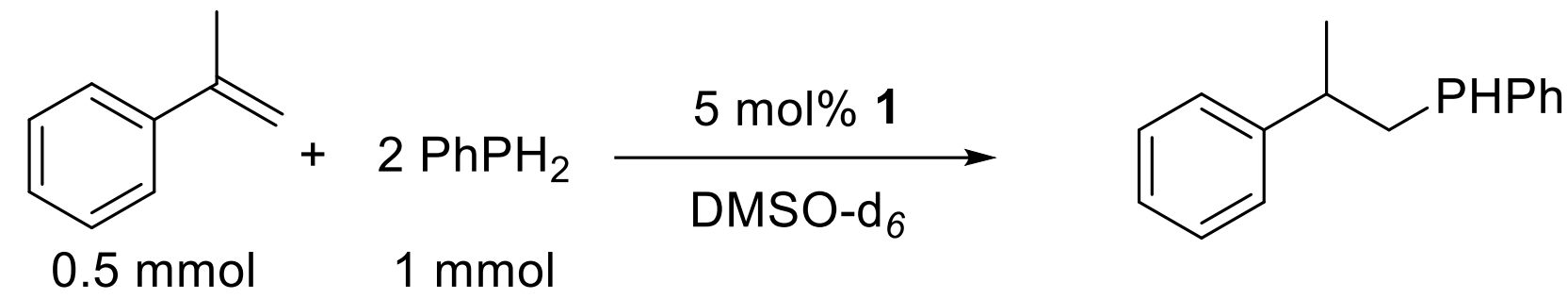

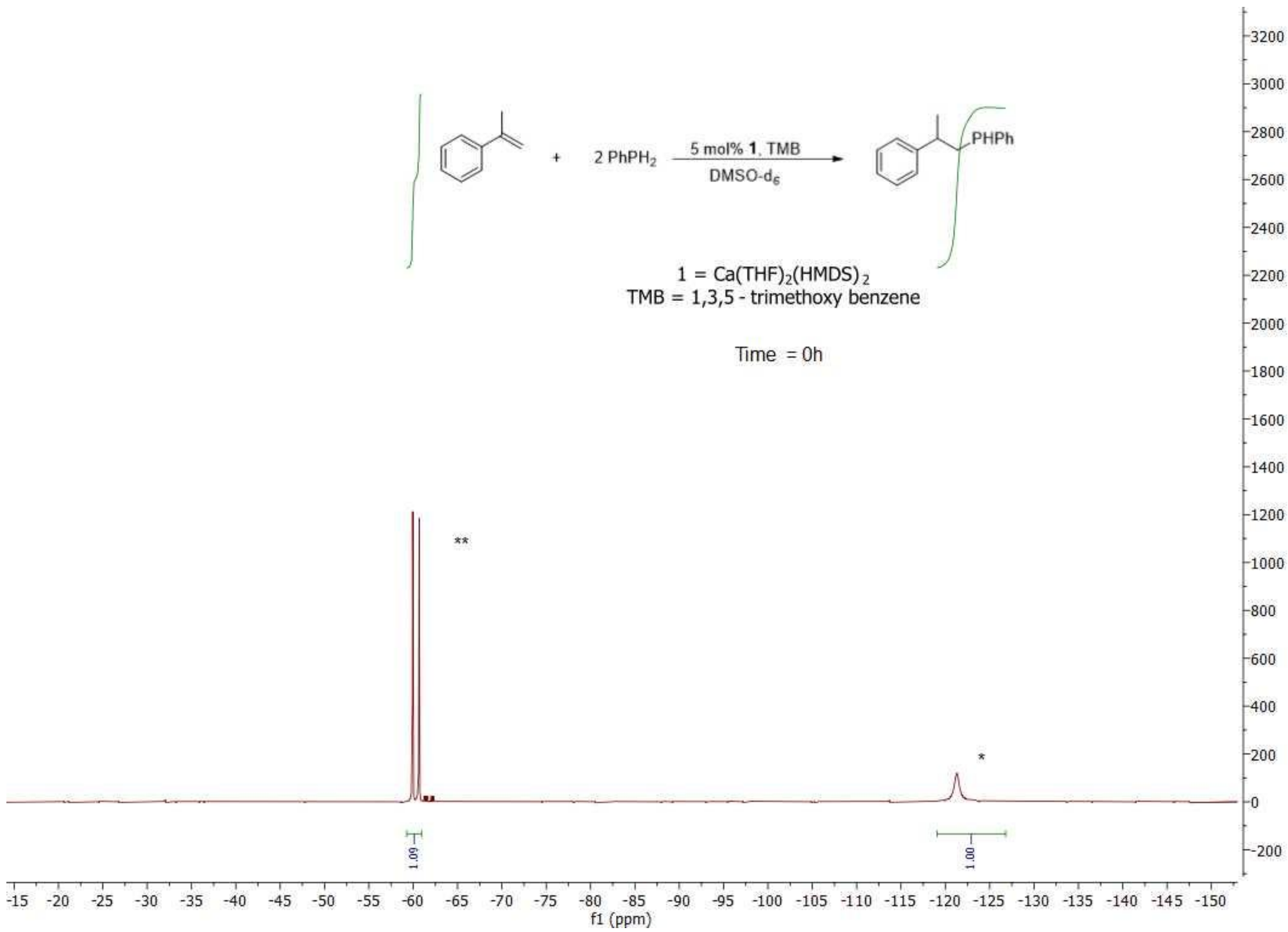

Figure S199: Hydrophosphination of 0.5 mmol α-methyl styrene with 2 equivalents of $PhPH_2$ in DMSO-d_6 using 5 mol% **1** – $^{31}P\{^1H\}$ NMR.

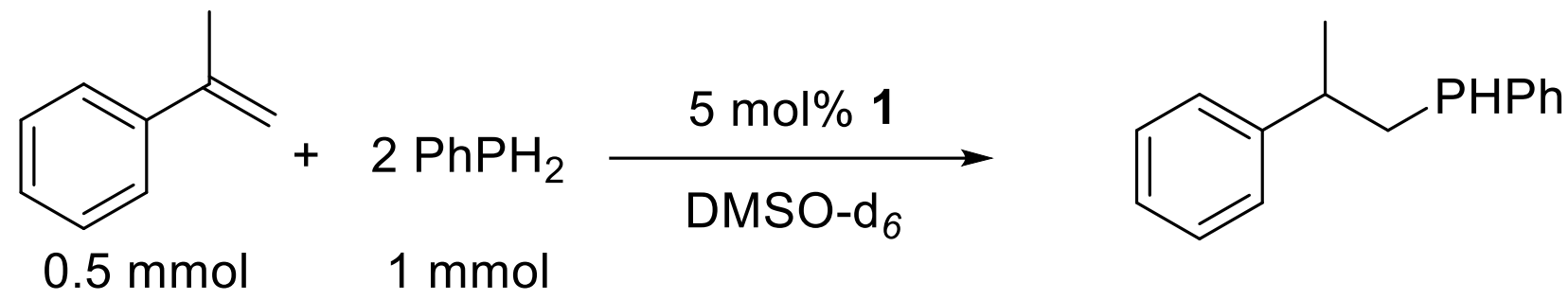

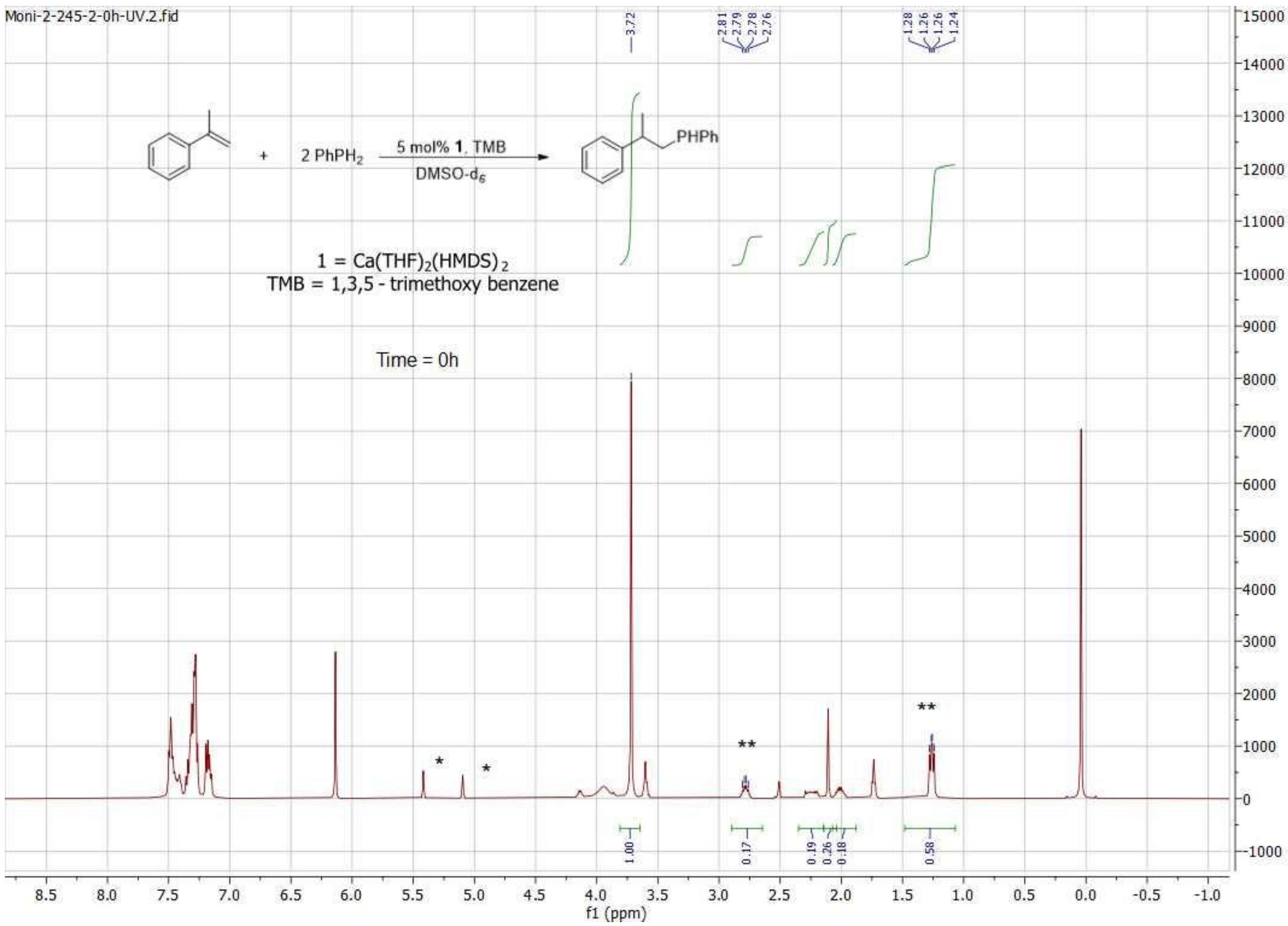

Figure S200: Hydrophosphination of 0.5 mmol α-methyl styrene with 2 equivalents of $PhPH_2$ in DMSO-d_6 using 5 mol% **1** – ^{1}H NMR.

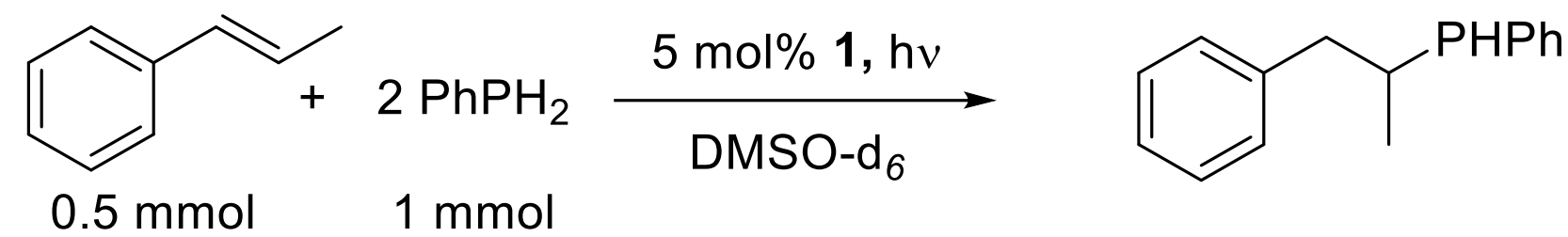

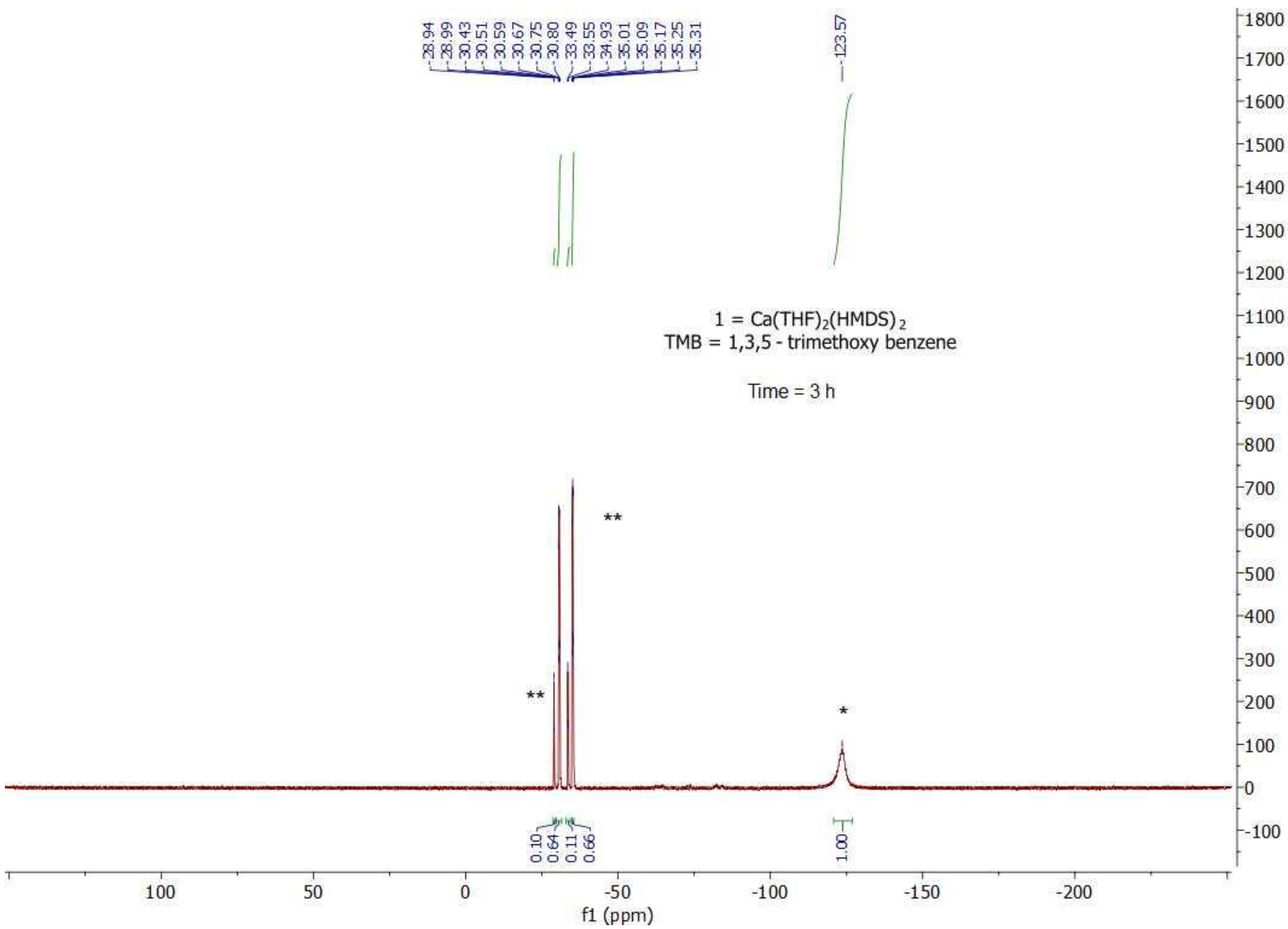

Figure S201: Hydrophosphination of 0.5 mmol β-methyl styrene with 2 equivalents of $PhPH_2$ in DMSO-d_6 using 5 mol% **1** under UV 360 nm irradiation – 3 h - $^{31}P\{^1H\}$ NMR.

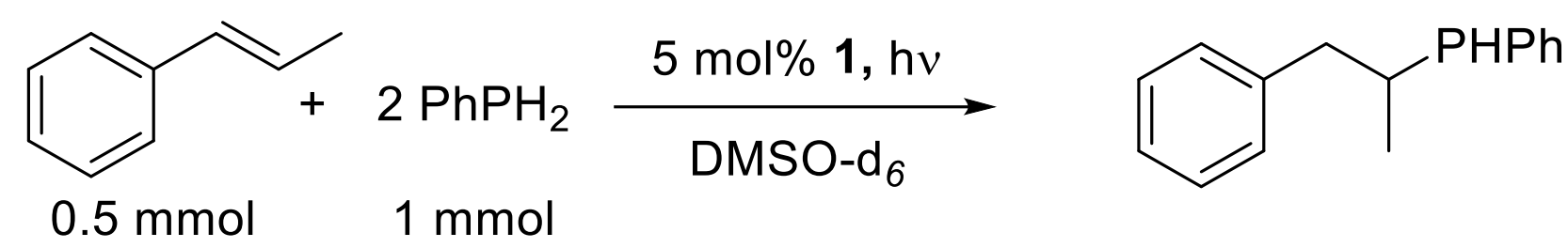

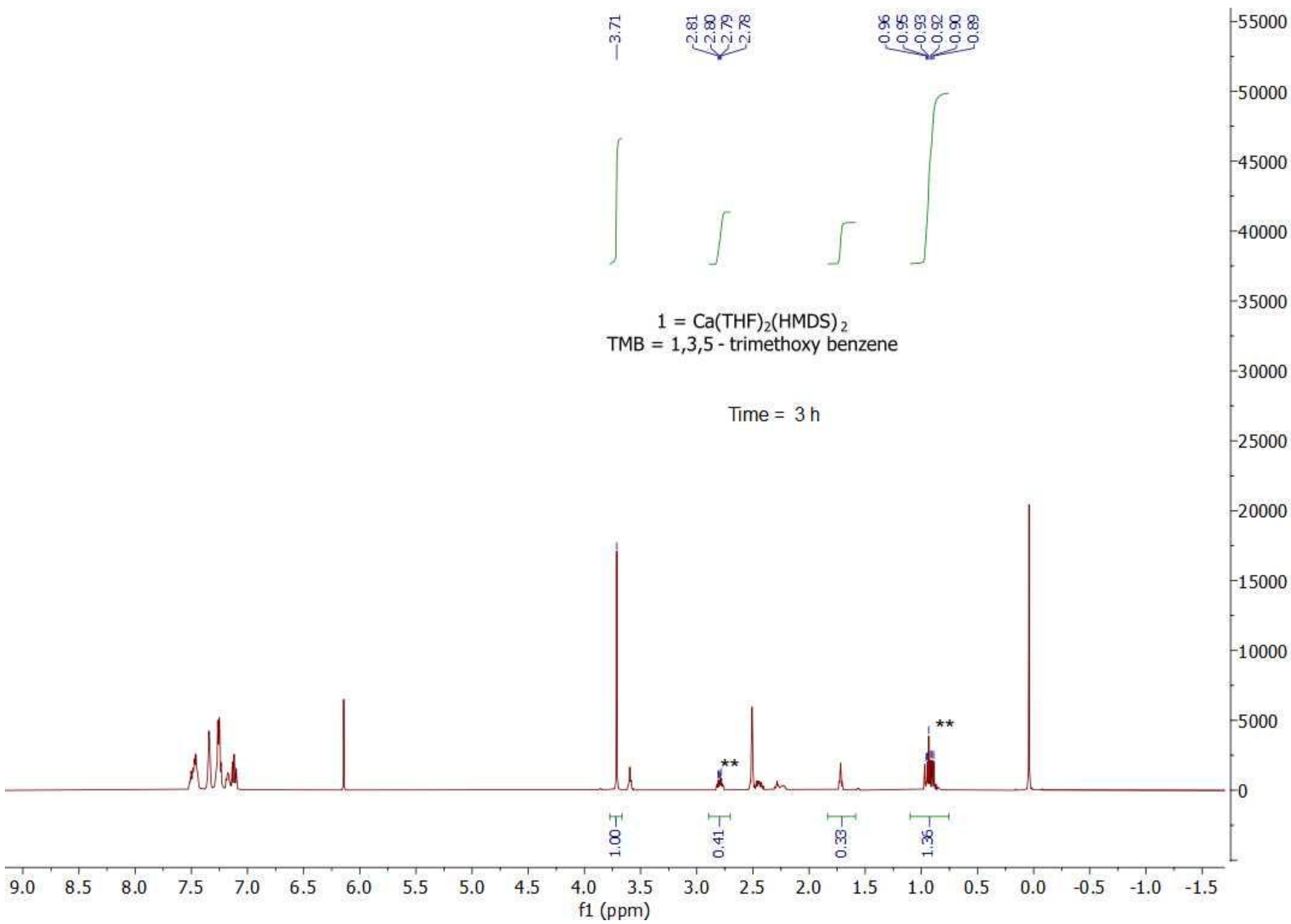

Figure S202: Hydrophosphination of 0.5 mmol β-methyl styrene with 2 equivalents of $PhPH_2$ in DMSO-d_6 using 5 mol% **1** under UV 360 nm irradiation -3 h – ^{1}H NMR.

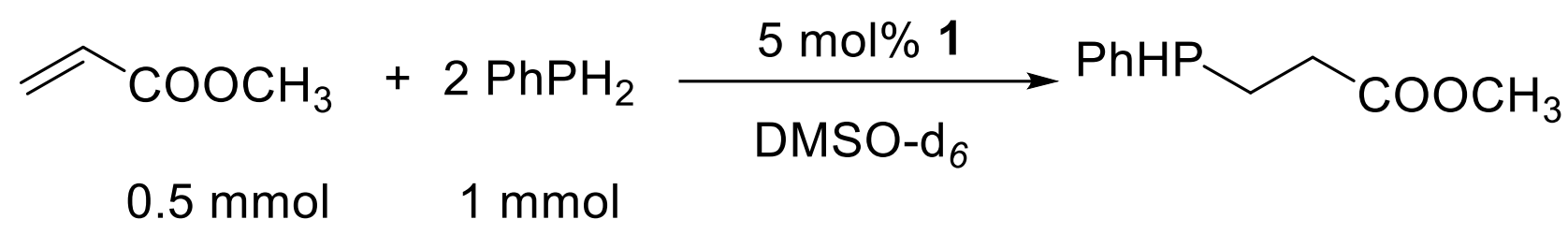

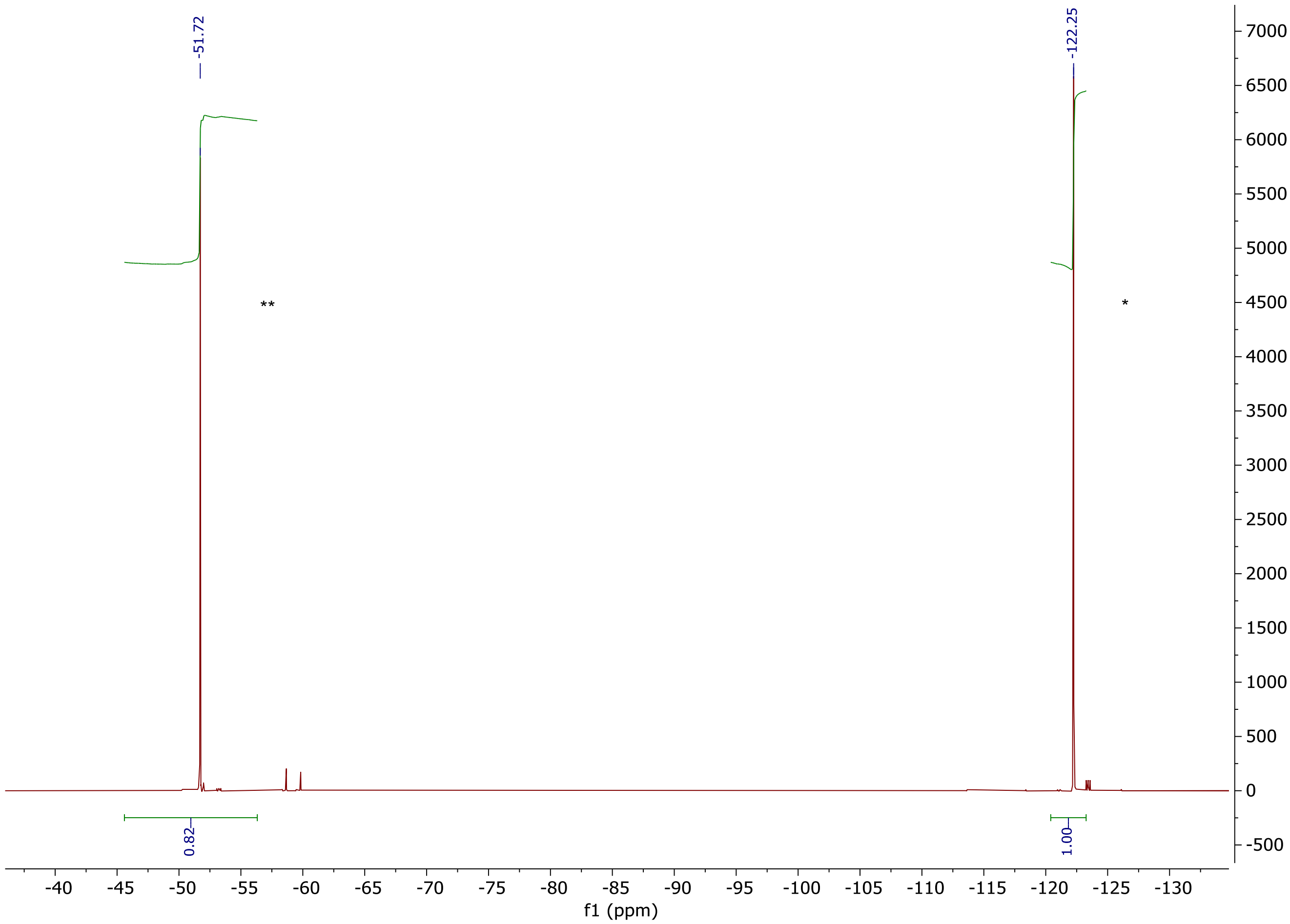

Figure S203: Hydrophosphination of 0.5 mmol methyl acrylate with 2 equivalents of $PhPH_2$ in DMSO-d_6 using 5 mol% **1** -0 h – $^{31}P\{^1H\}$ NMR.

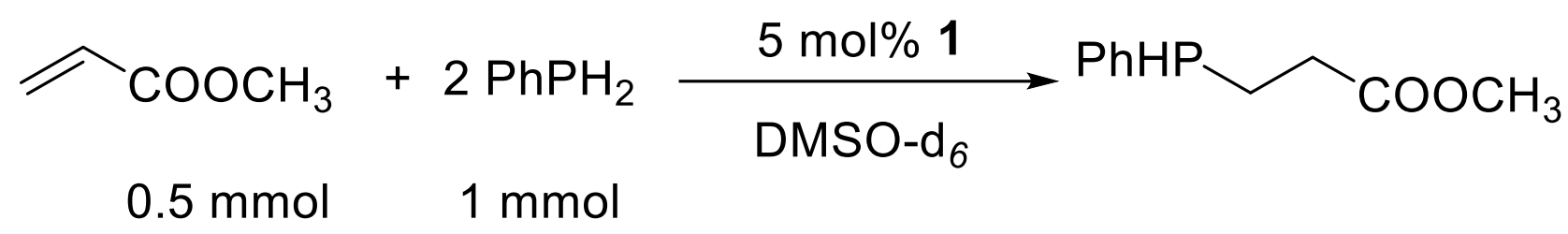

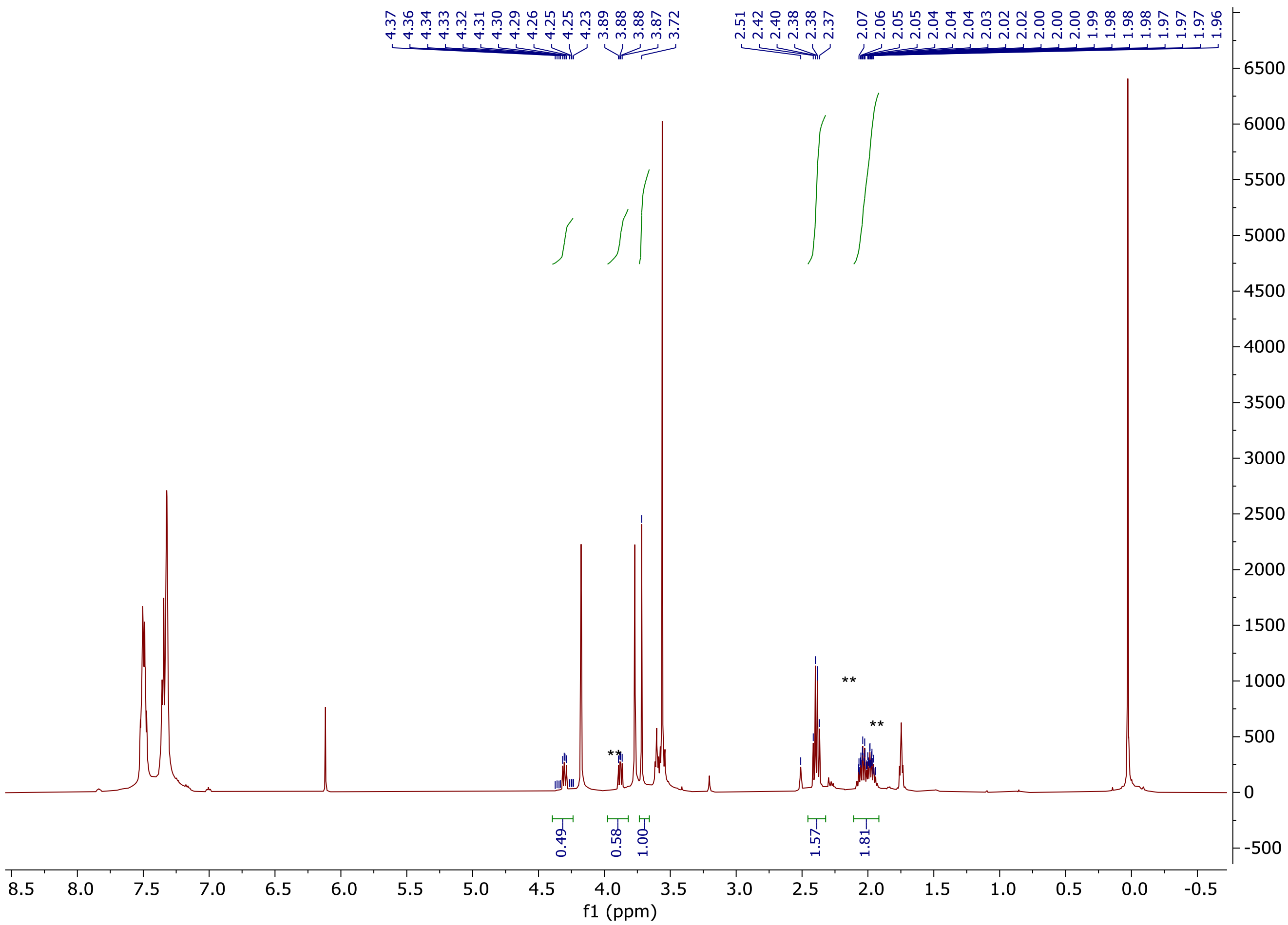

Figure S204: Hydrophosphination of 0.5 mmol methyl acrylate with 2 equivalents of $PhPH_2$ in DMSO-d_6 using 5 mol% **1** - 0 h – ^{1}H NMR.

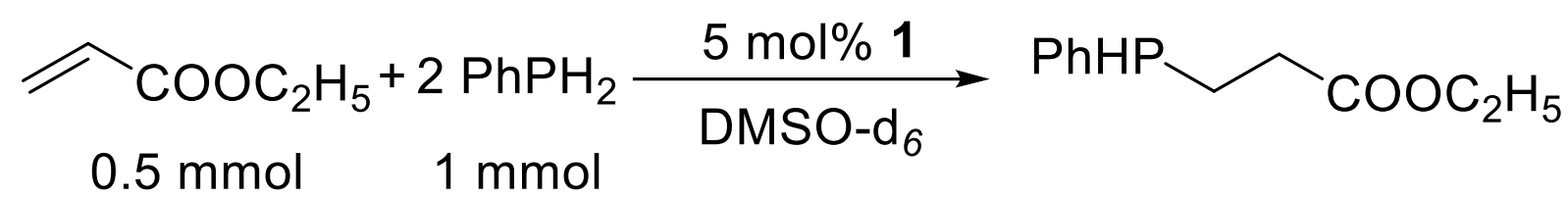

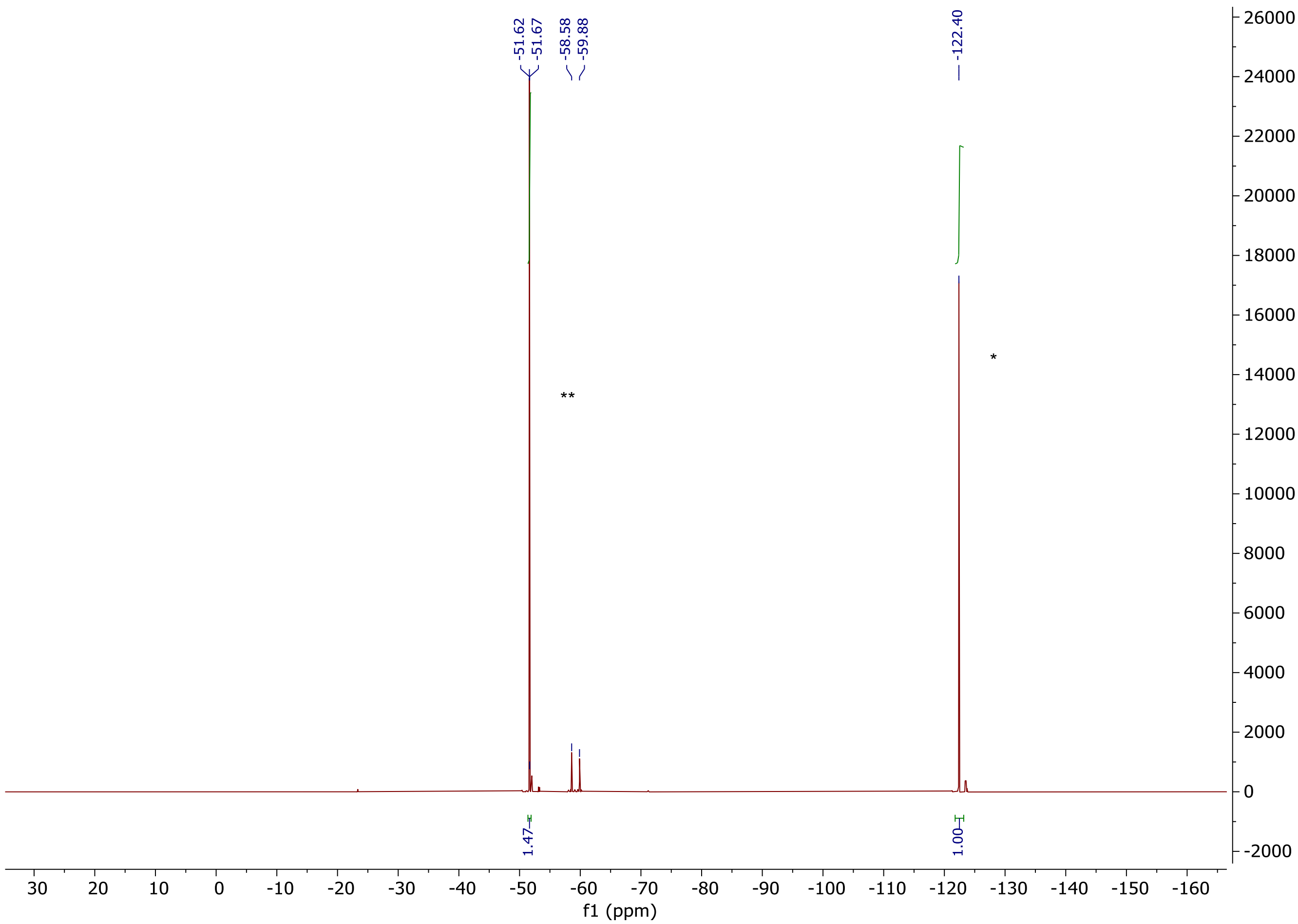

Figure S205: Hydrophosphination of 0.5 mmol ethyl acrylate with 2 equivalents of $PhPH_2$ in DMSO-d_6 using 5 mol% **1** - 0 h – $^{31}P\{^1H\}$ NMR.

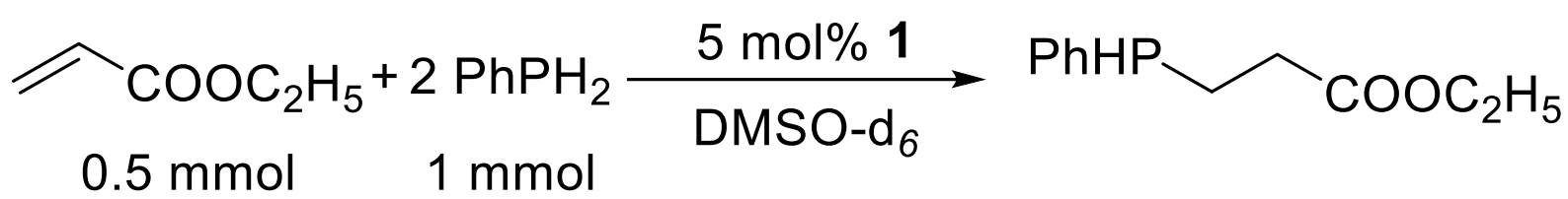

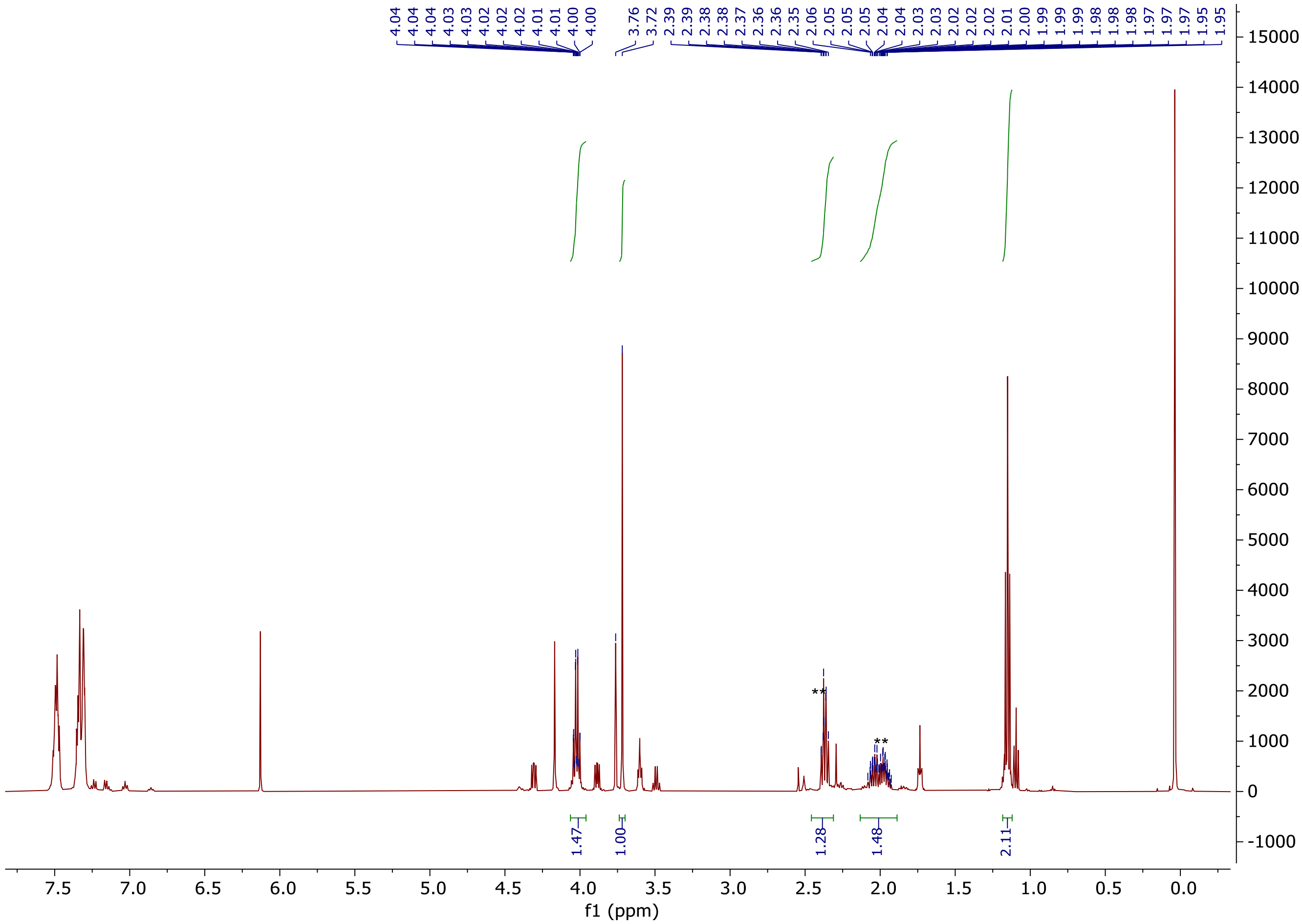

Figure S206: Hydrophosphination of 0.5 mmol ethyl acrylate with 2 equivalents of $PhPH_2$ in DMSO-d_6 using 5 mol% **1** - 0 h – ^{1}H NMR.

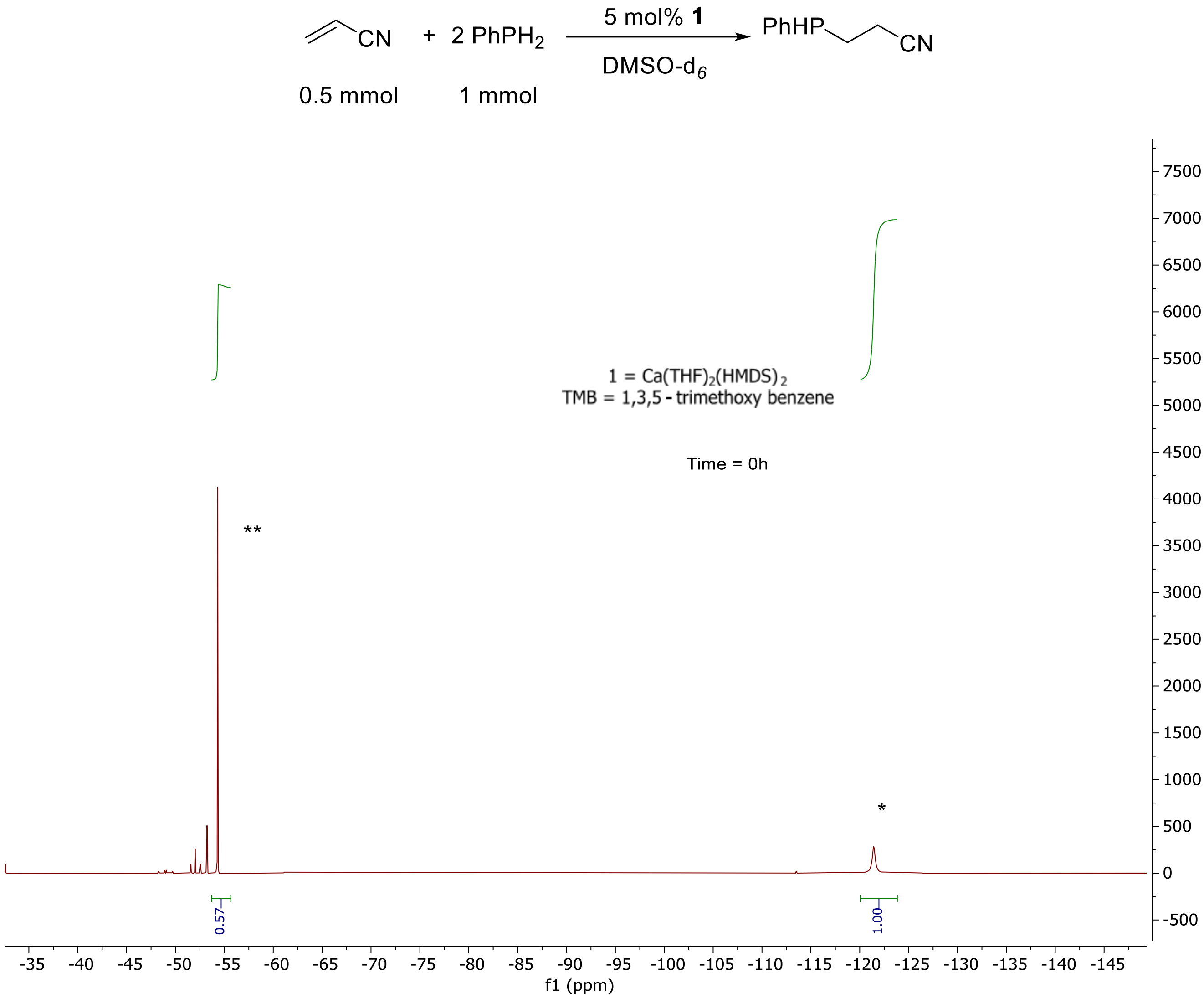

Figure S207: Hydrophosphination of 0.5 mmol acrylonitrile with 2 equivalents of $PhPH_2$ in DMSO-d_6 using 5 mol% **1** - 0 h – $^{31}P\{^1H\}$ NMR.

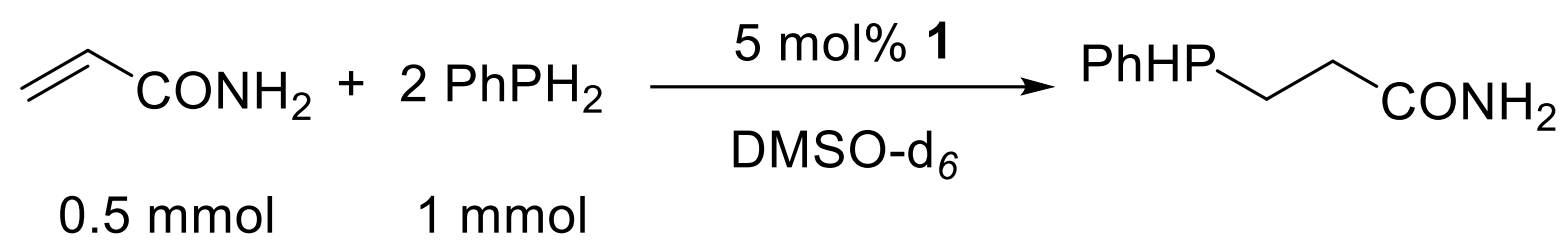

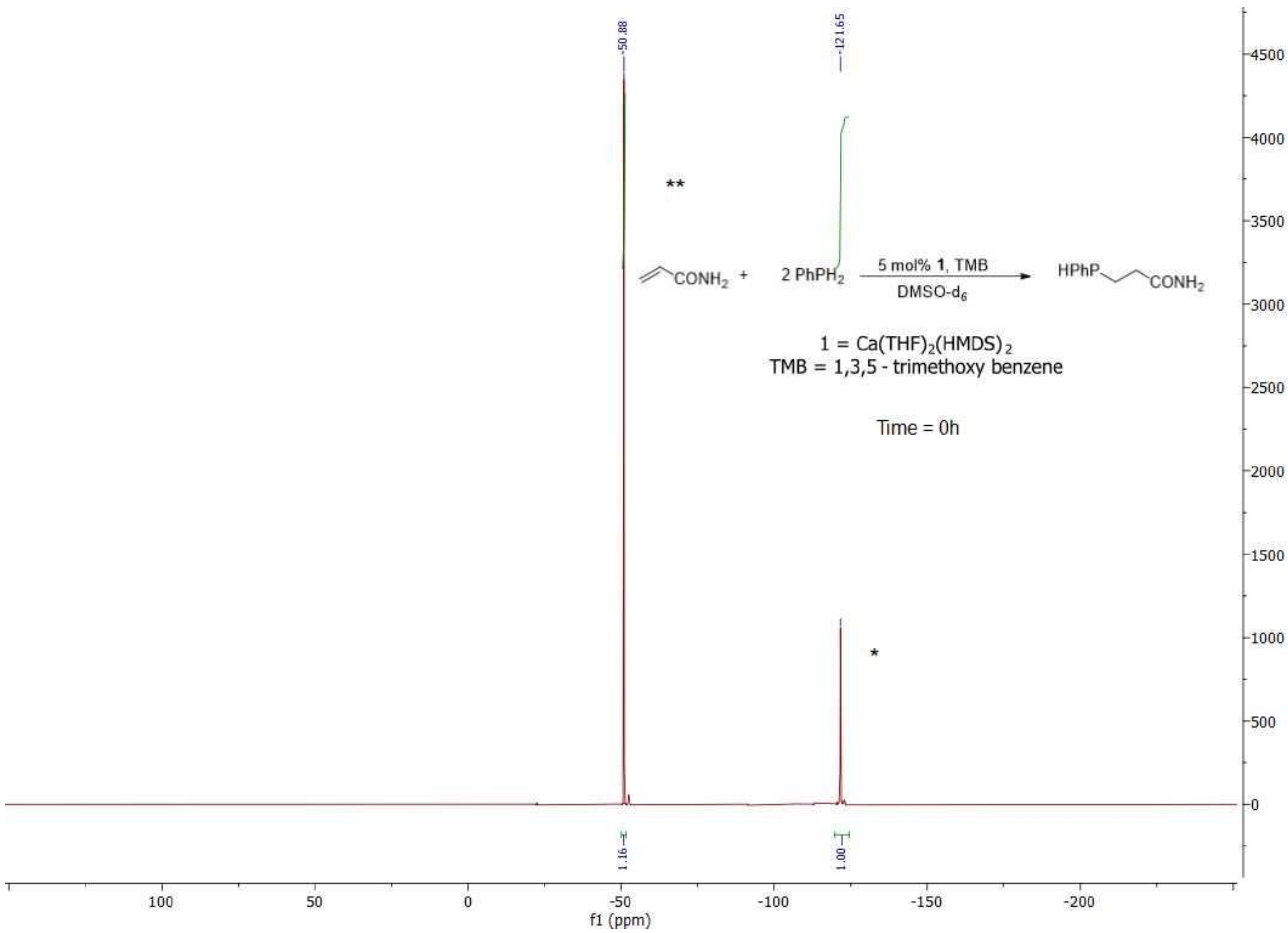

Figure S208: Hydrophosphination of 0.5 mmol acrylamide with 2 equivalents of $PhPH_2$ in DMSO-d_6 using 5 mol% **1** - 0 h – $^{31}P\{^{1}H\}$ NMR.

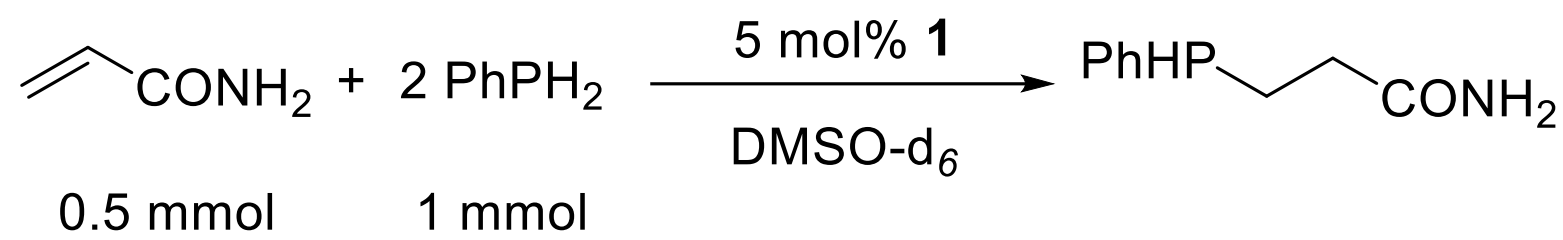

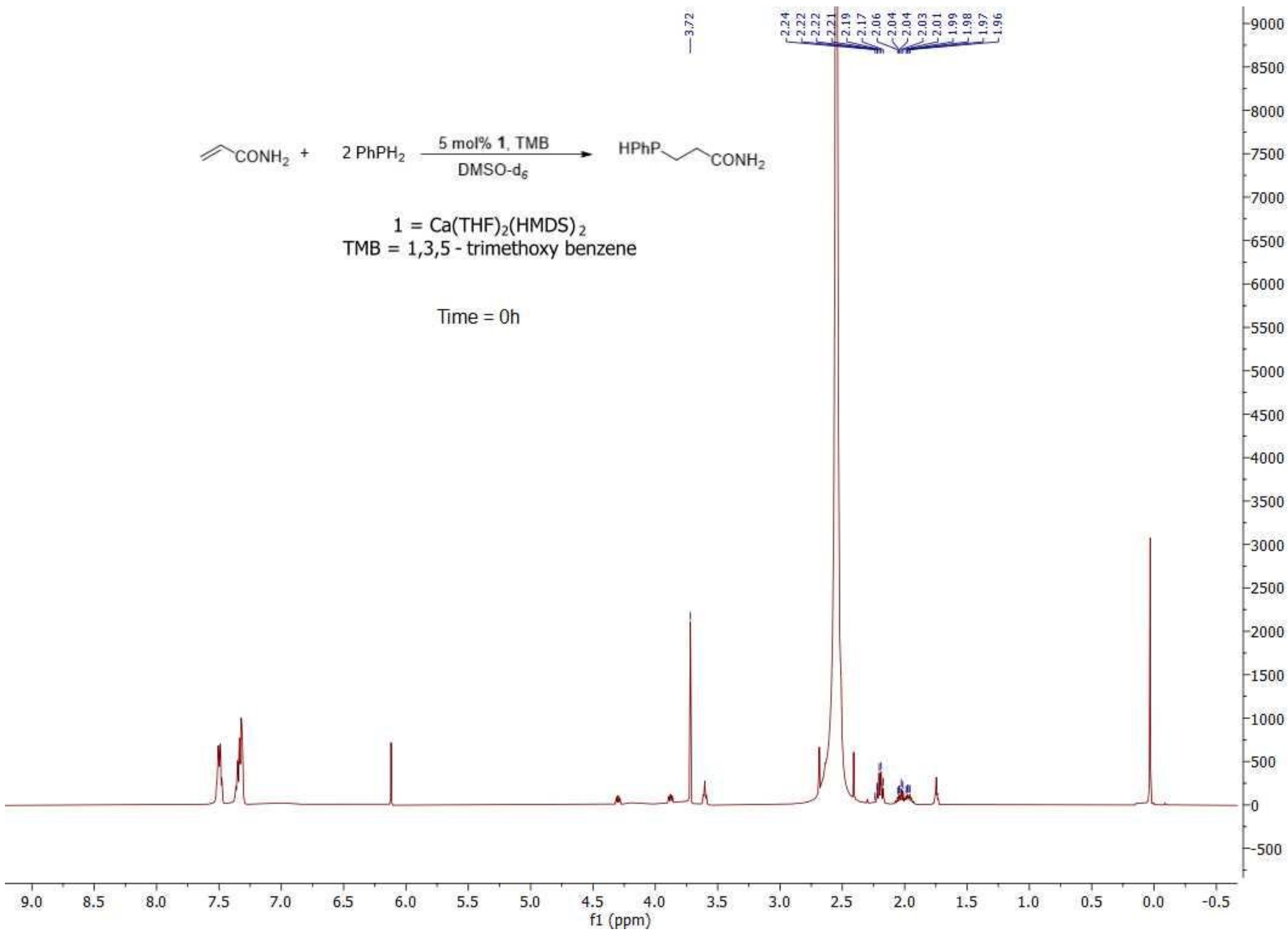

Figure S209: Hydrophosphination of 0.5 mmol acrylamide with 2 equivalents of $PhPH_2$ in DMSO-d_6 using 5 mol% **1** -0 h – ^{1}H NMR.

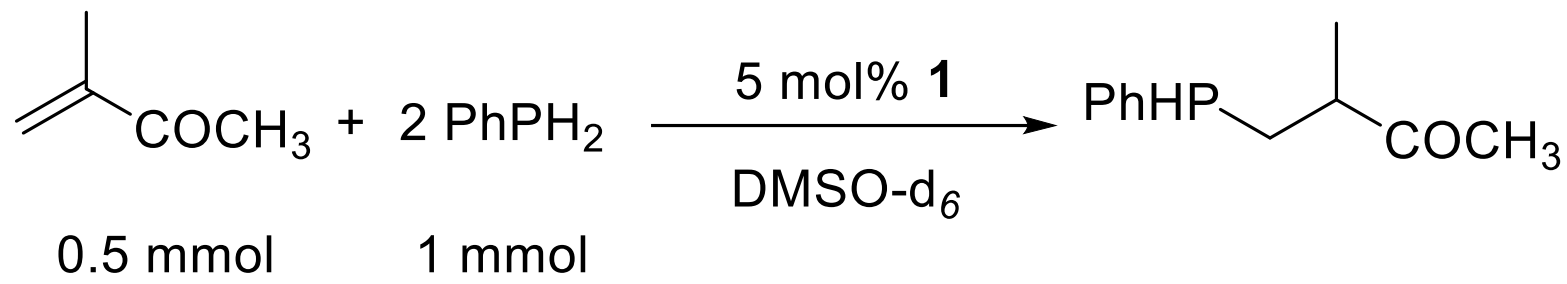

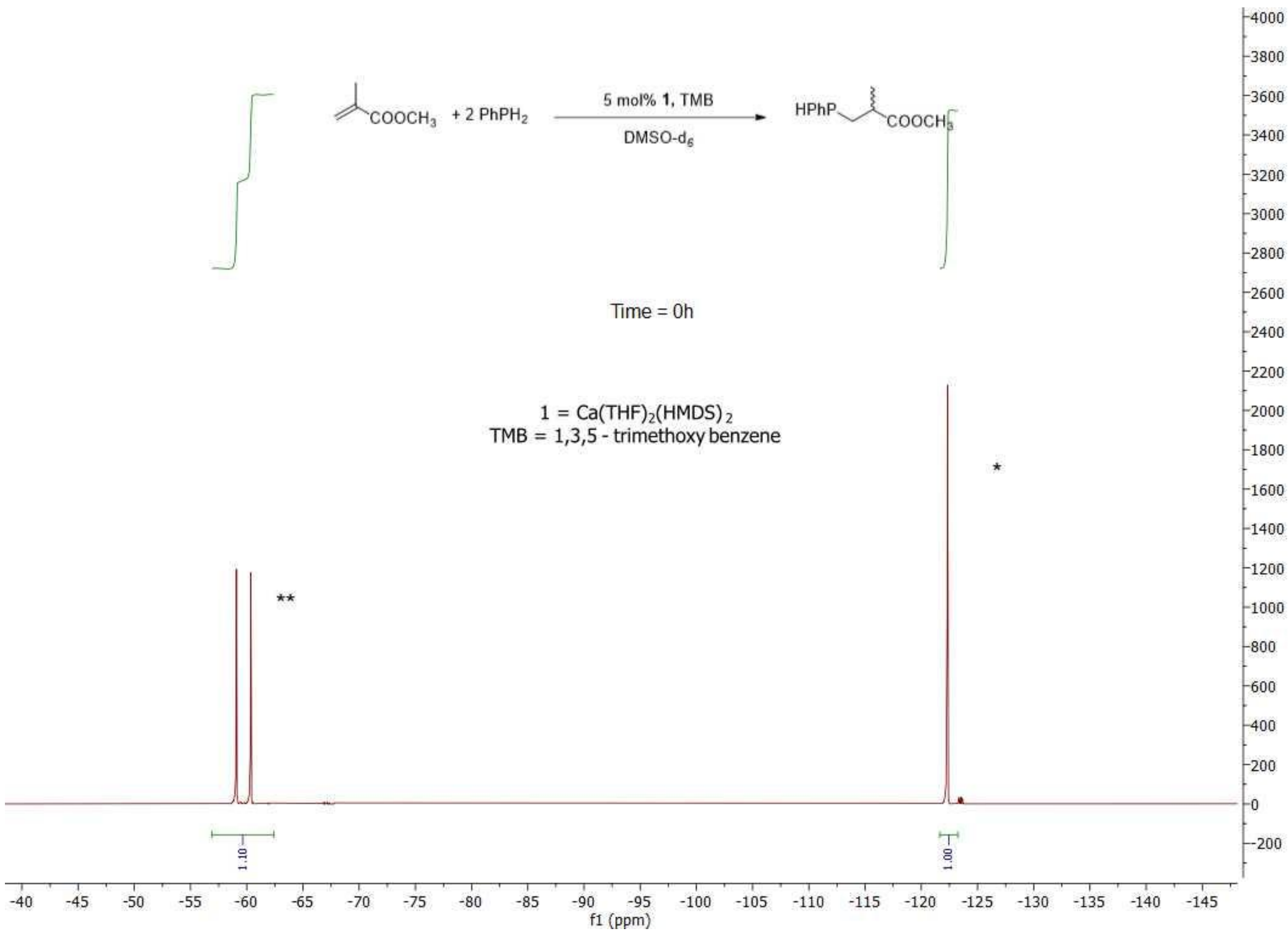

Figure S210: Hydrophosphination of 0.5 mmol methylmethacrylate with 2 equivalents of $PhPH_2$ in DMSO-d_6 using 5 mol% **1** - 0 h – $^{31}P\{^1H\}$ NMR.

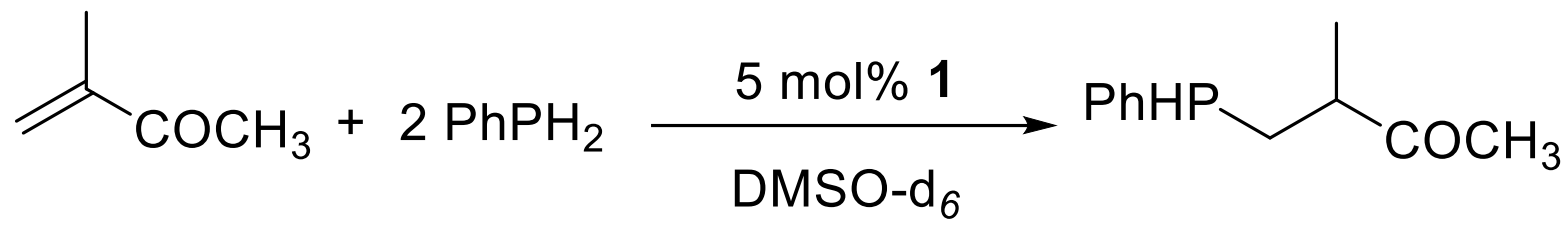

0.5 mmol 1 mmol

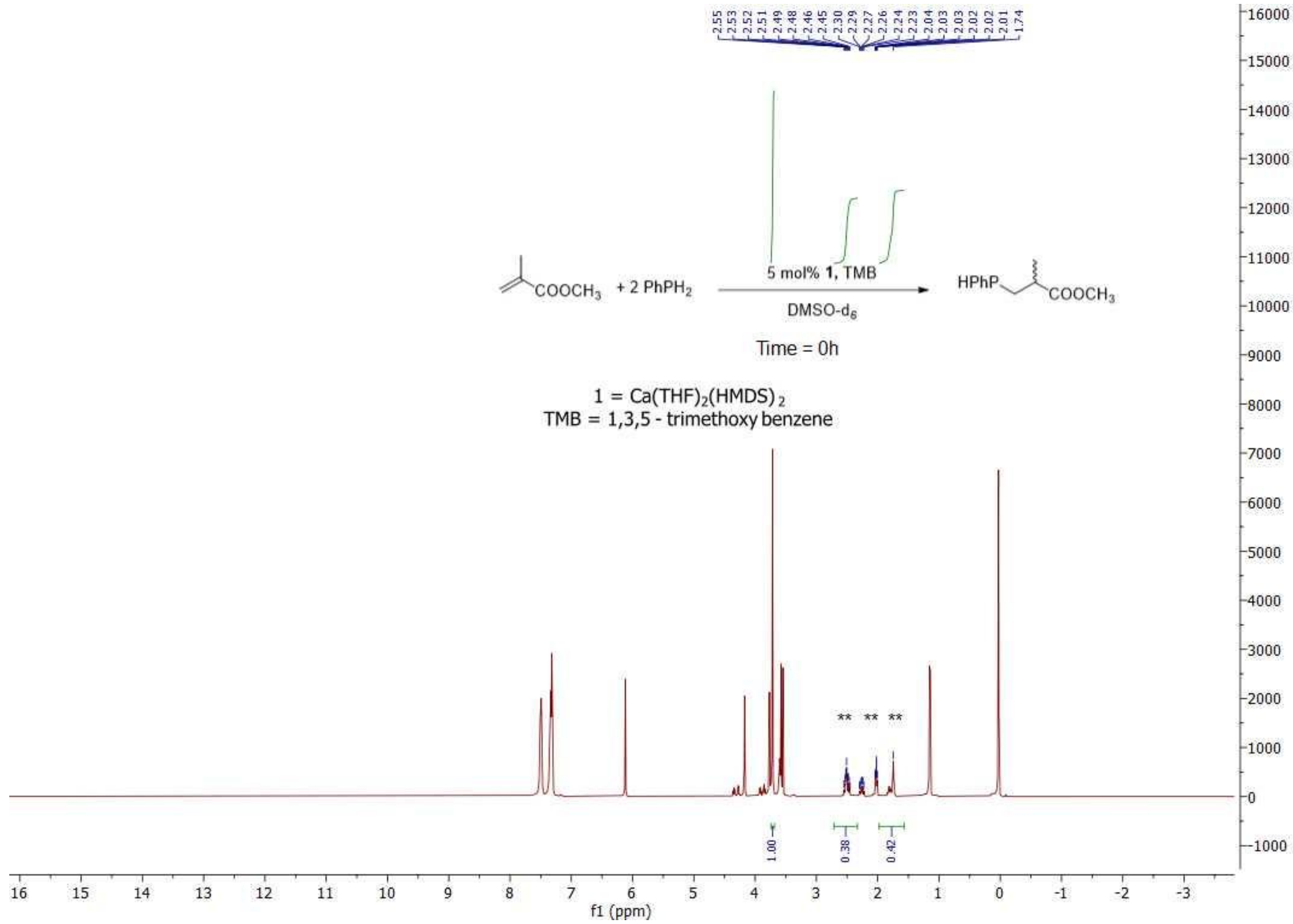

Figure 211: Hydrophosphination of 0.5 mmol methylmethacrylate with 2 equivalents of $PhPH_2$ in DMSO-d_6 using 5 mol% **1** - 0 h – ^1H NMR.

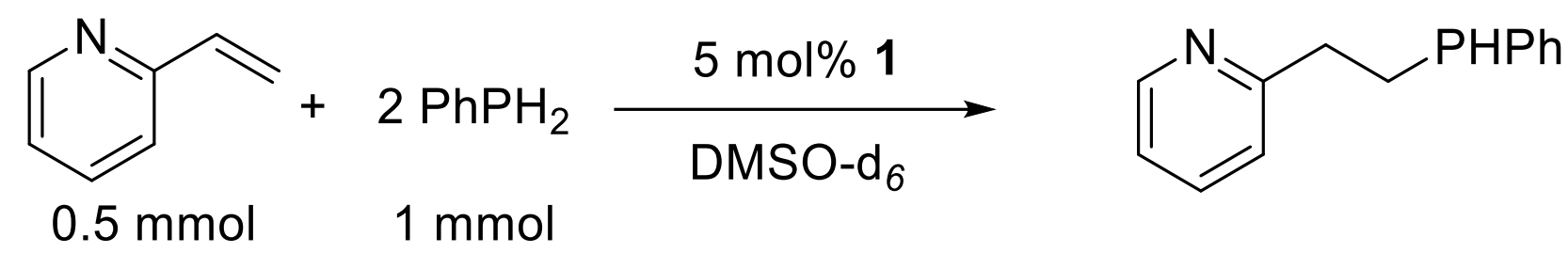

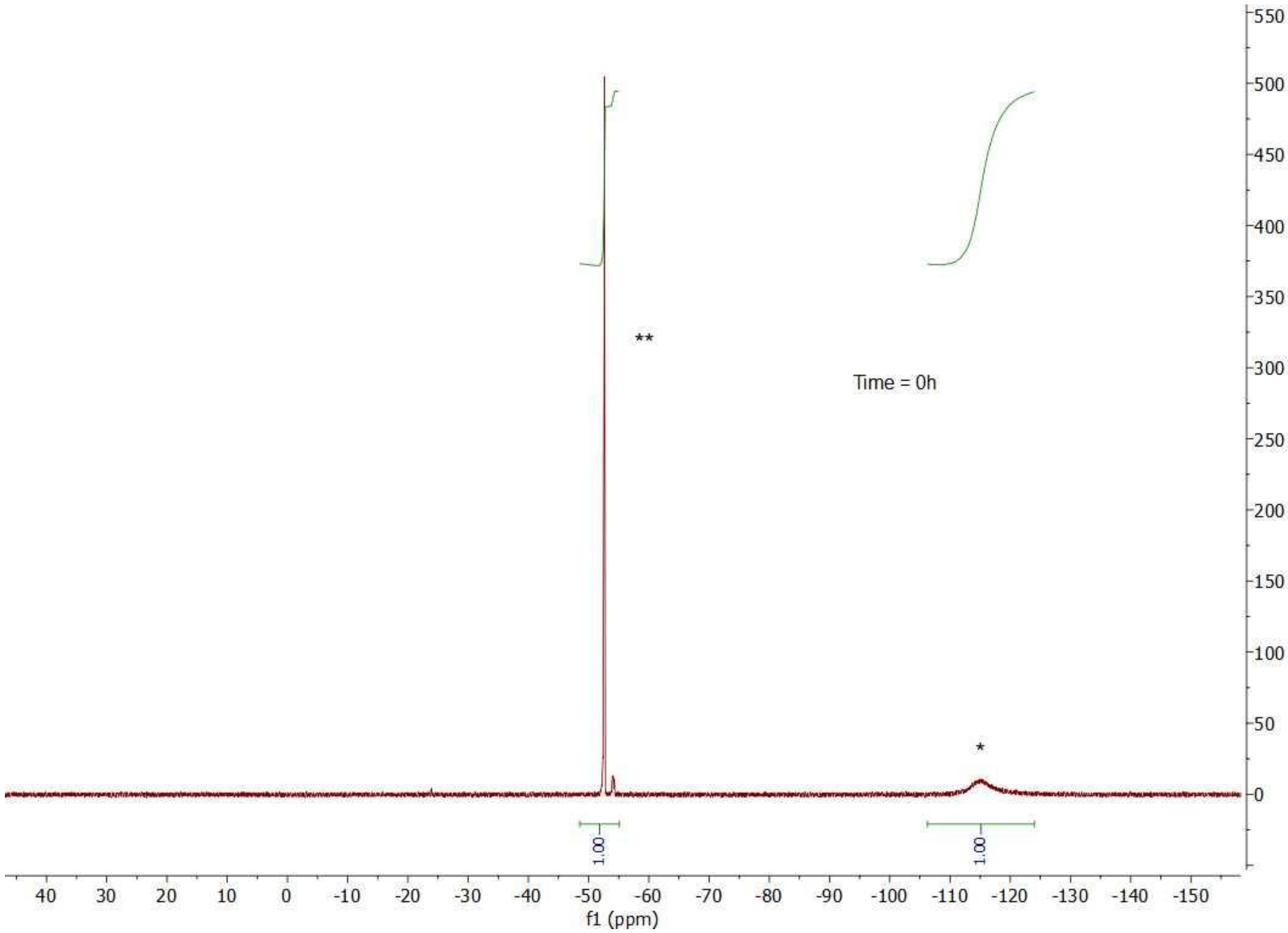

Figure S212: Hydrophosphination of 0.5 mmol 2-vinyl pyridine with 2 equivalents of $PhPH_2$ in DMSO-d_6 using 5 mol% **1** - 0 h – $^{31}P\{^1H\}$ NMR.

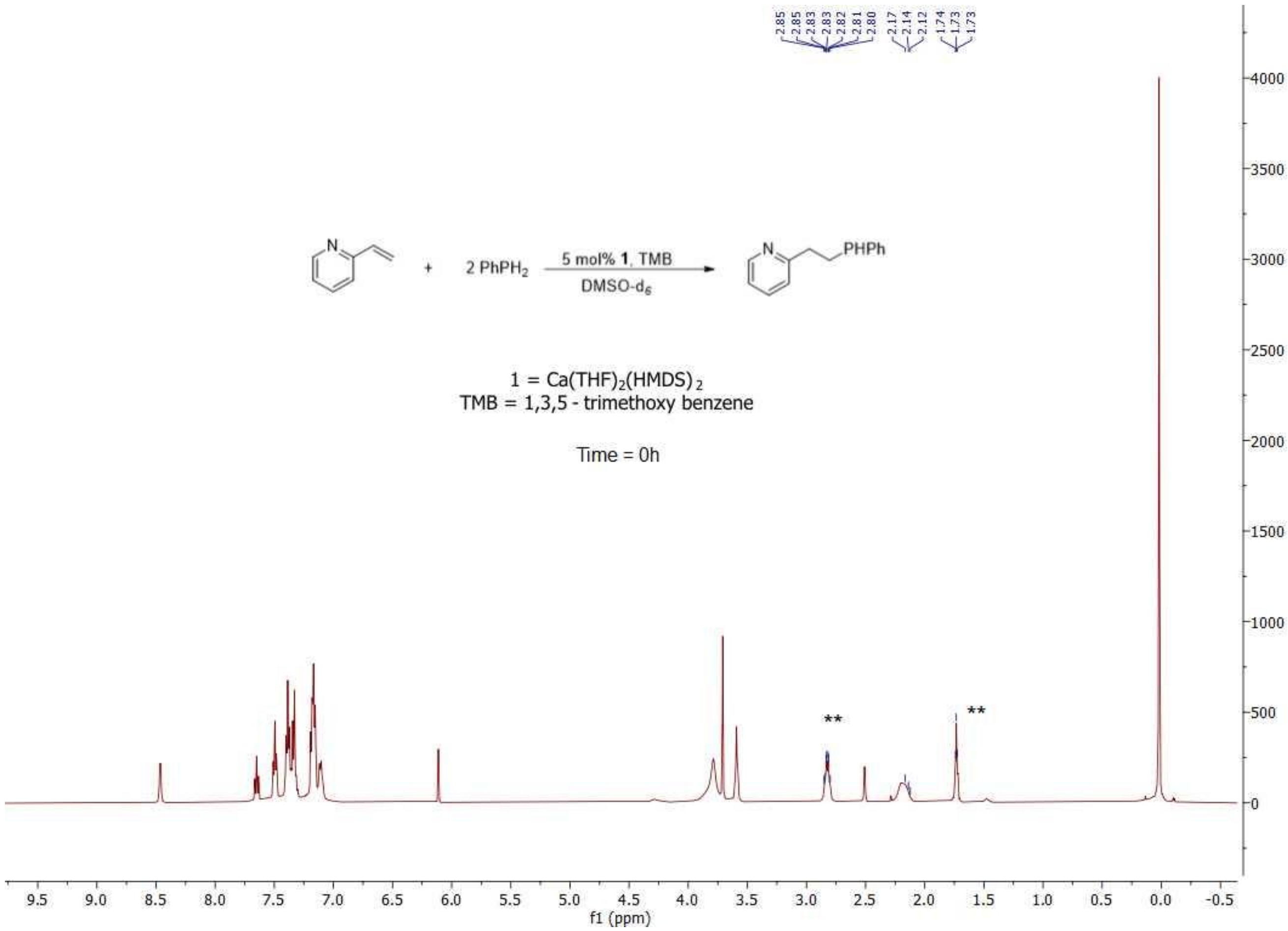

Figure S213: Hydrophosphination of 0.5 mmol 2-vinyl pyridine with 2 equivalents of $PhPH_2$ in DMSO-d_6 using 5 mol% **1** - 0 h –^{1}H NMR.

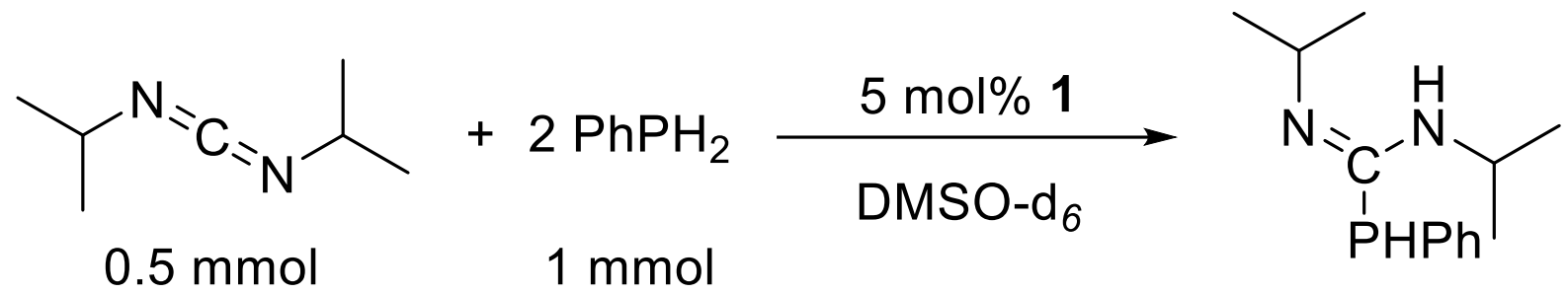

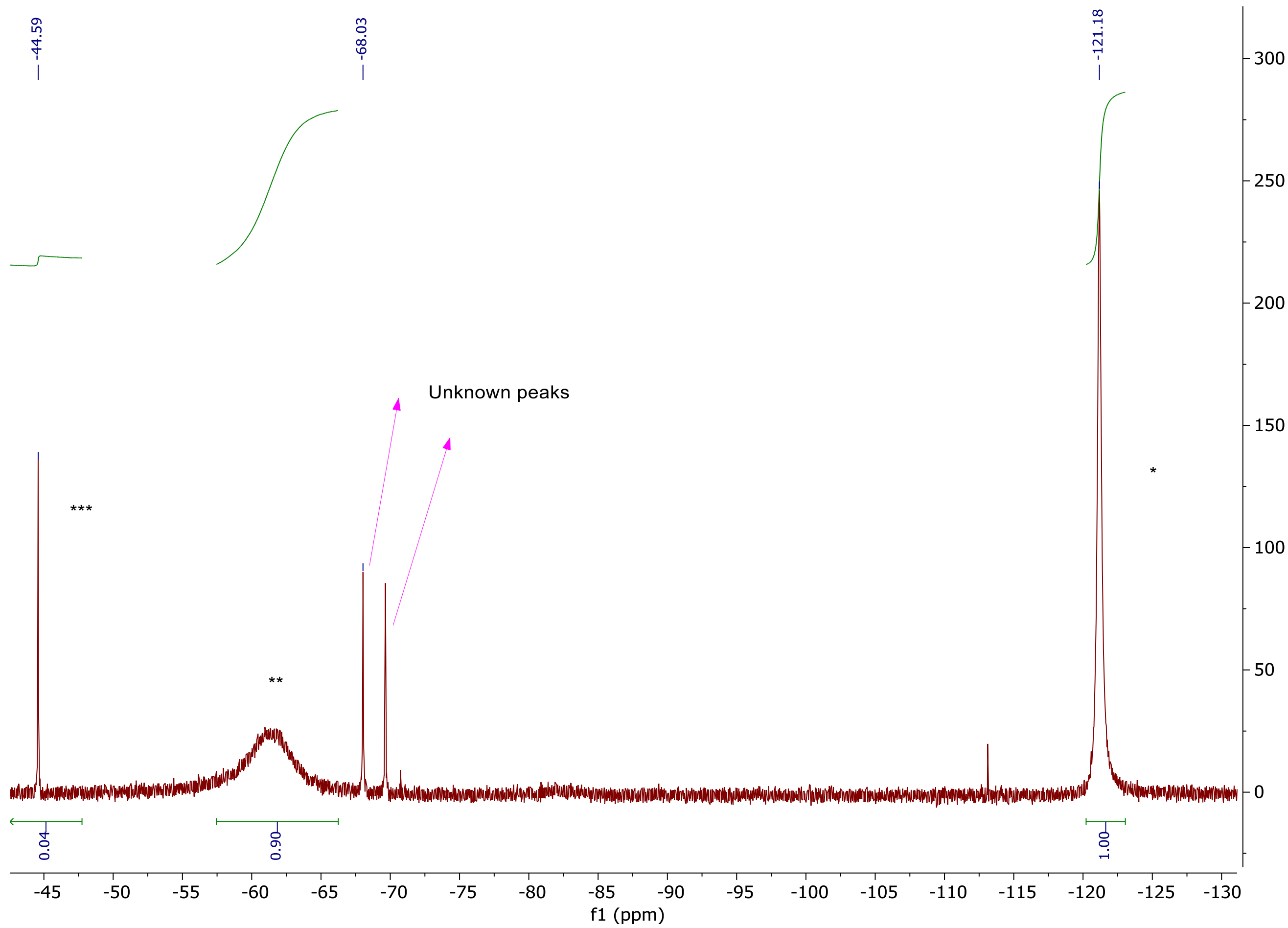

Figure S214: Hydrophosphination of 0.5 mmol N, N' – diisopropyl carbodiimide with 2 equivalents of $PhPH_2$ in DMSO-d_6 using 5 mol% **1** - 0 h – $^{31}P\{^1H\}$ NMR.

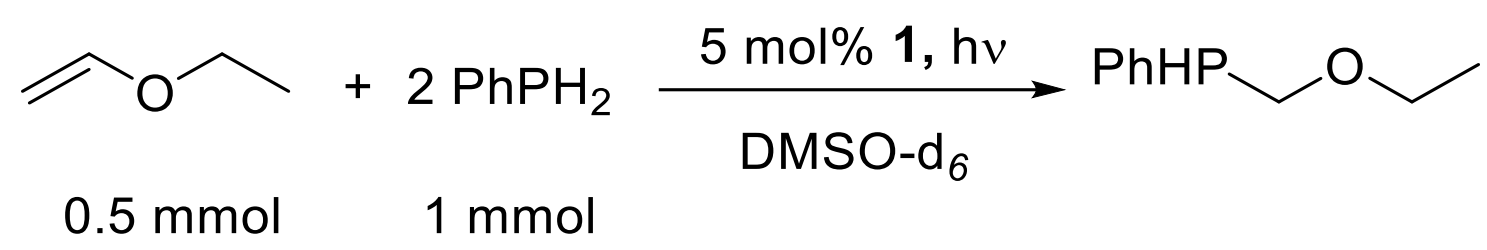

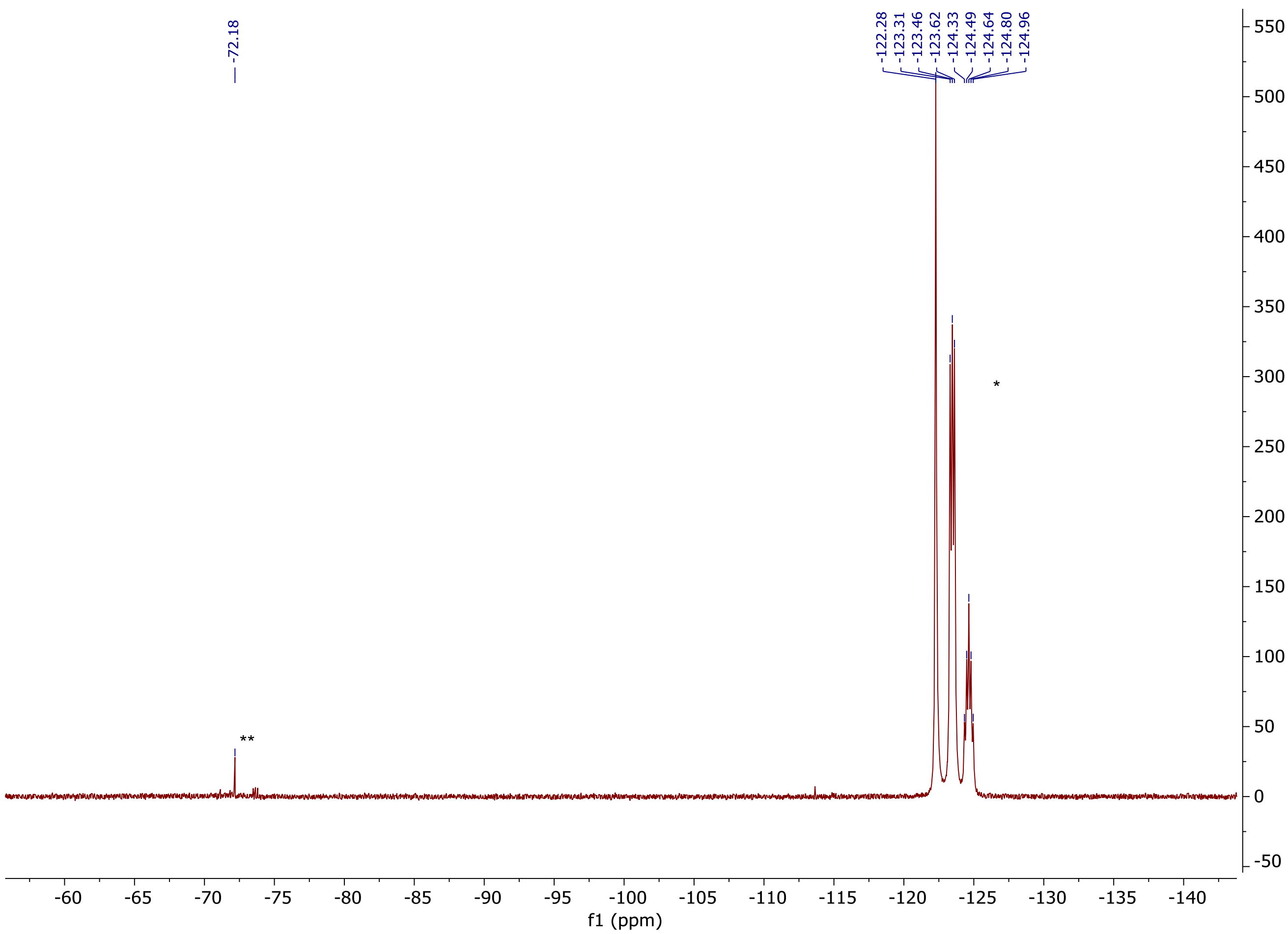

Figure S215: Hydrophosphination of 0.5 mmol vinyl ethyl ether with 2 equivalents of $PhPH_2$ in DMSO-d_6 using 5 mol% **1** under UV 360 nm irradiation - 24 h – $^{31}P\{^1H\}$ NMR

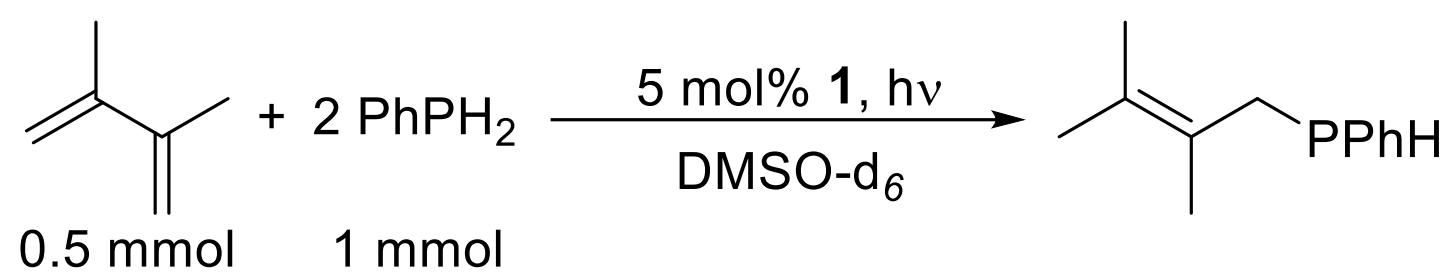

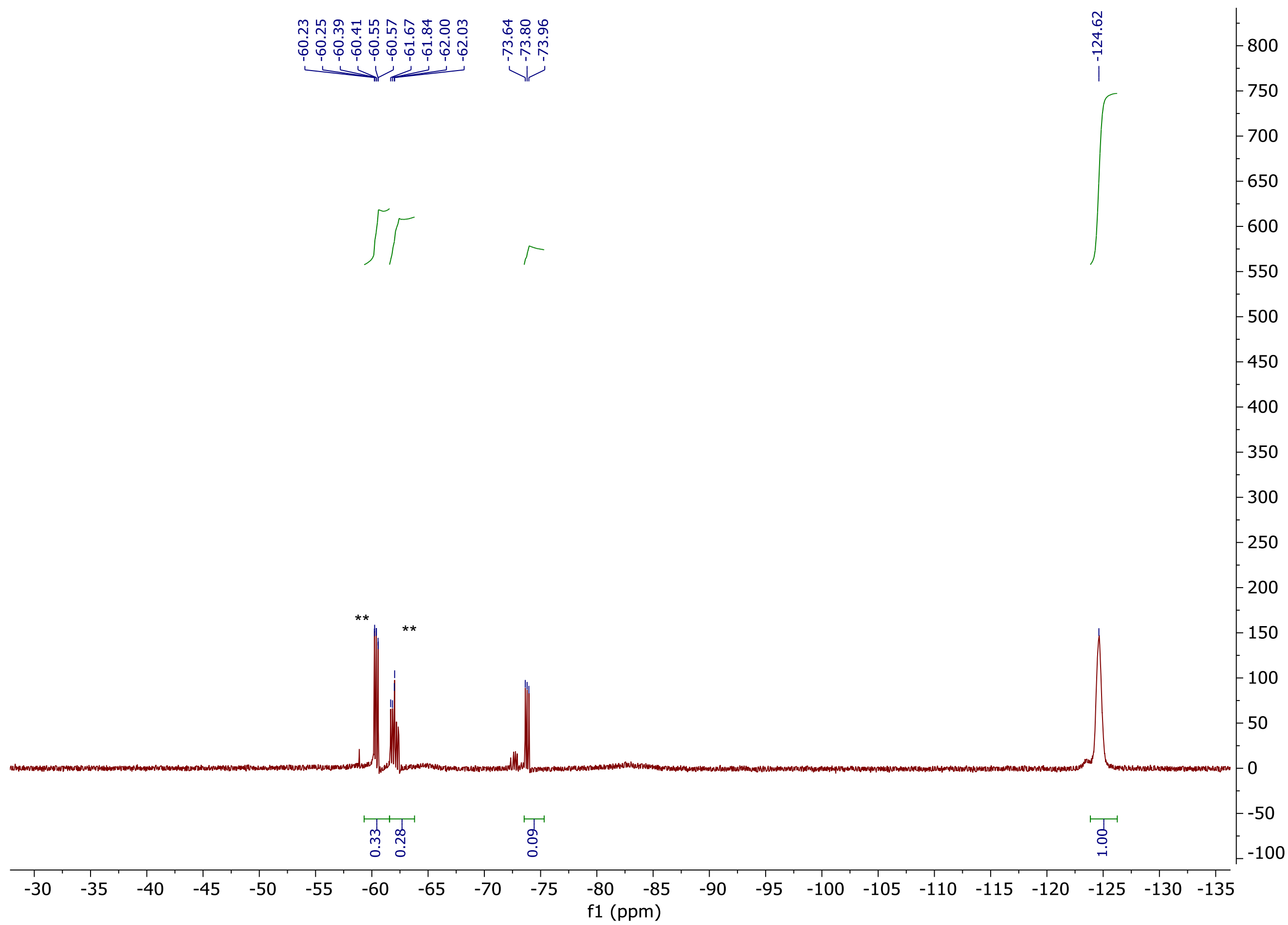

Figure S216: Hydrophosphination of 0.5 mmol 2,3-dimethyl-1,3-butadiene with 2 equivalents of $PhPH_2$ in DMSO-d_6 using 5 mol% **1** under UV 360 nm irradiation - 48 h – $^{31}P\{^1H\}$ NMR

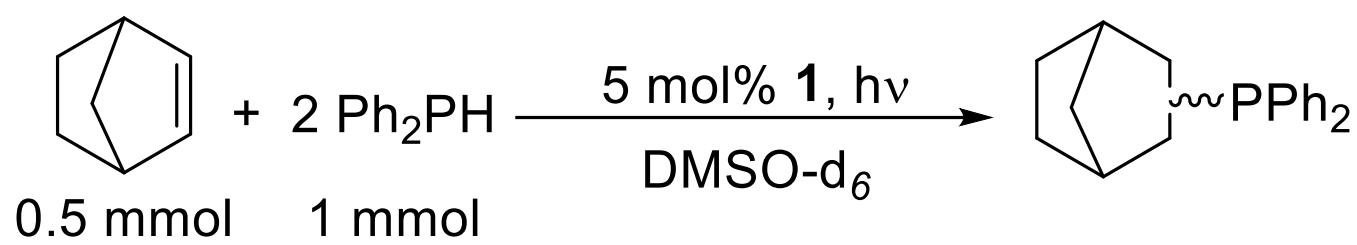

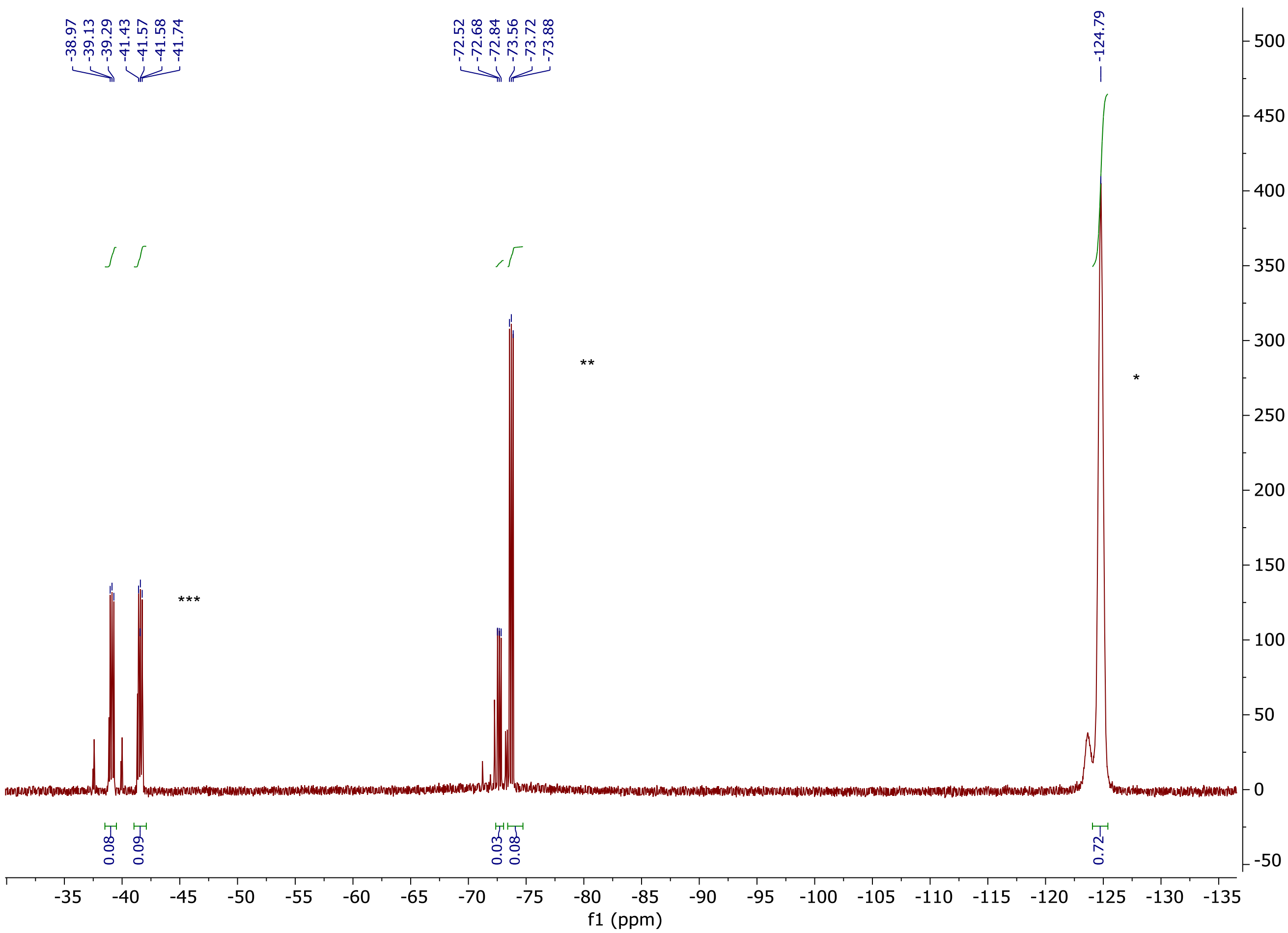

Figure S217: Hydrophosphination of 0.5 mmol norborene with 2 equivalents of $PhPH_2$ in DMSO-d_6 using 5 mol% **1** under UV 360 nm irradiation - 48 h – $^{31}P\{^1H\}$ NMR

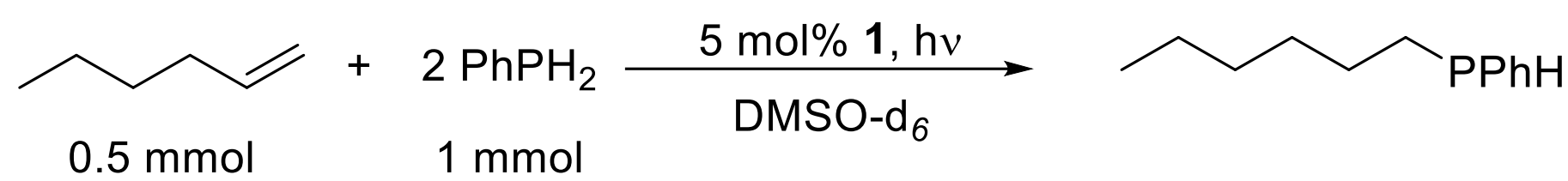

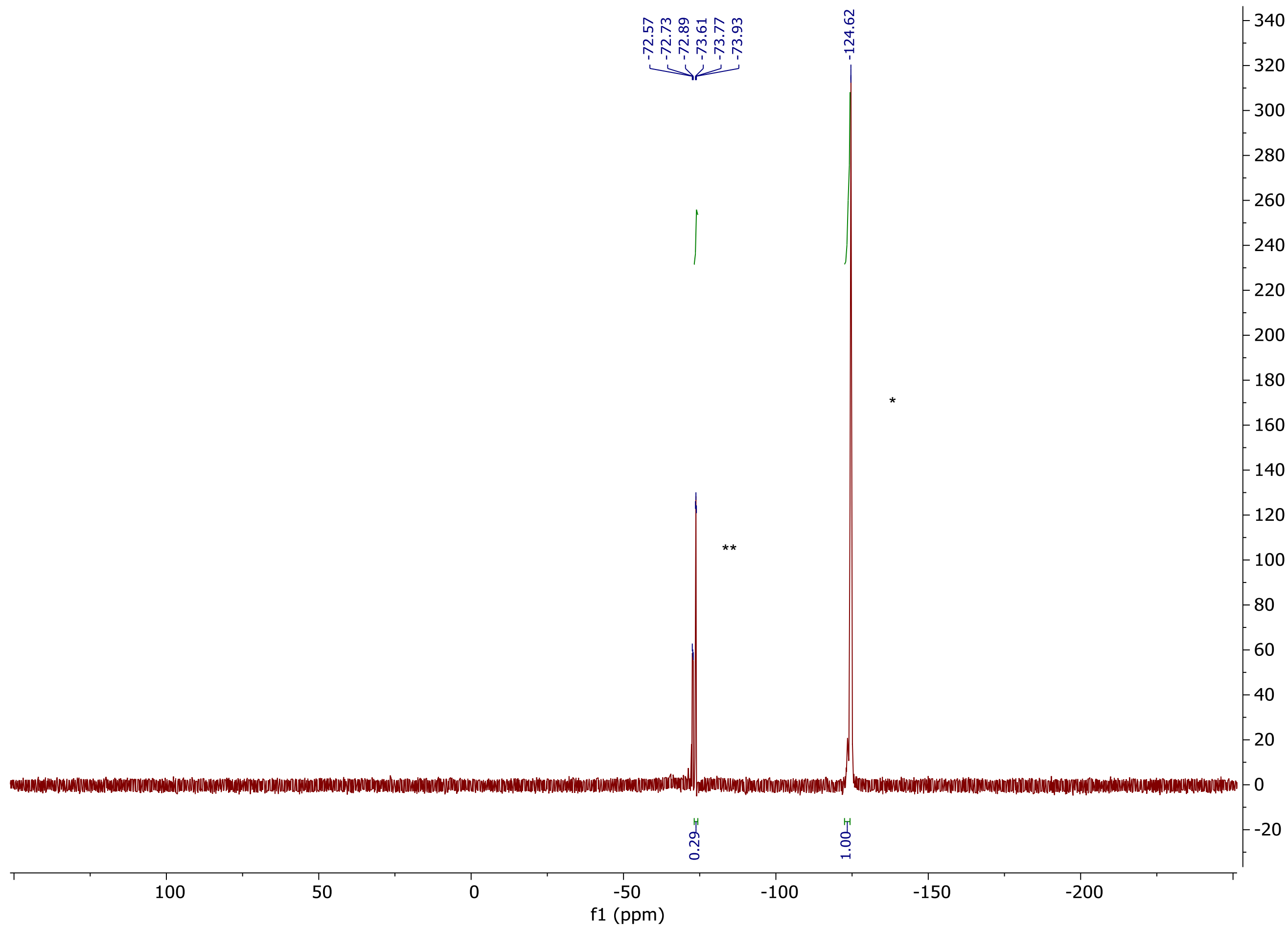

Figure S218: Hydrophosphination of 0.5 mmol 1-hexene with 2 equivalents of $PhPH_2$ in DMSO-d_6 using 5 mol% **1** under UV 360 nm irradiation - 24 h – $^{31}P\{^1H\}$ NMR

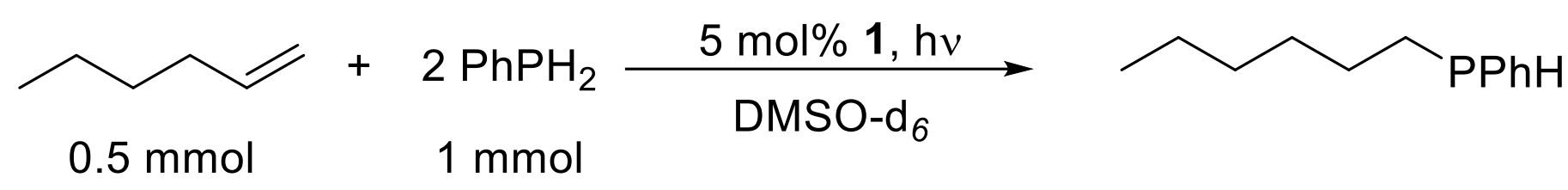

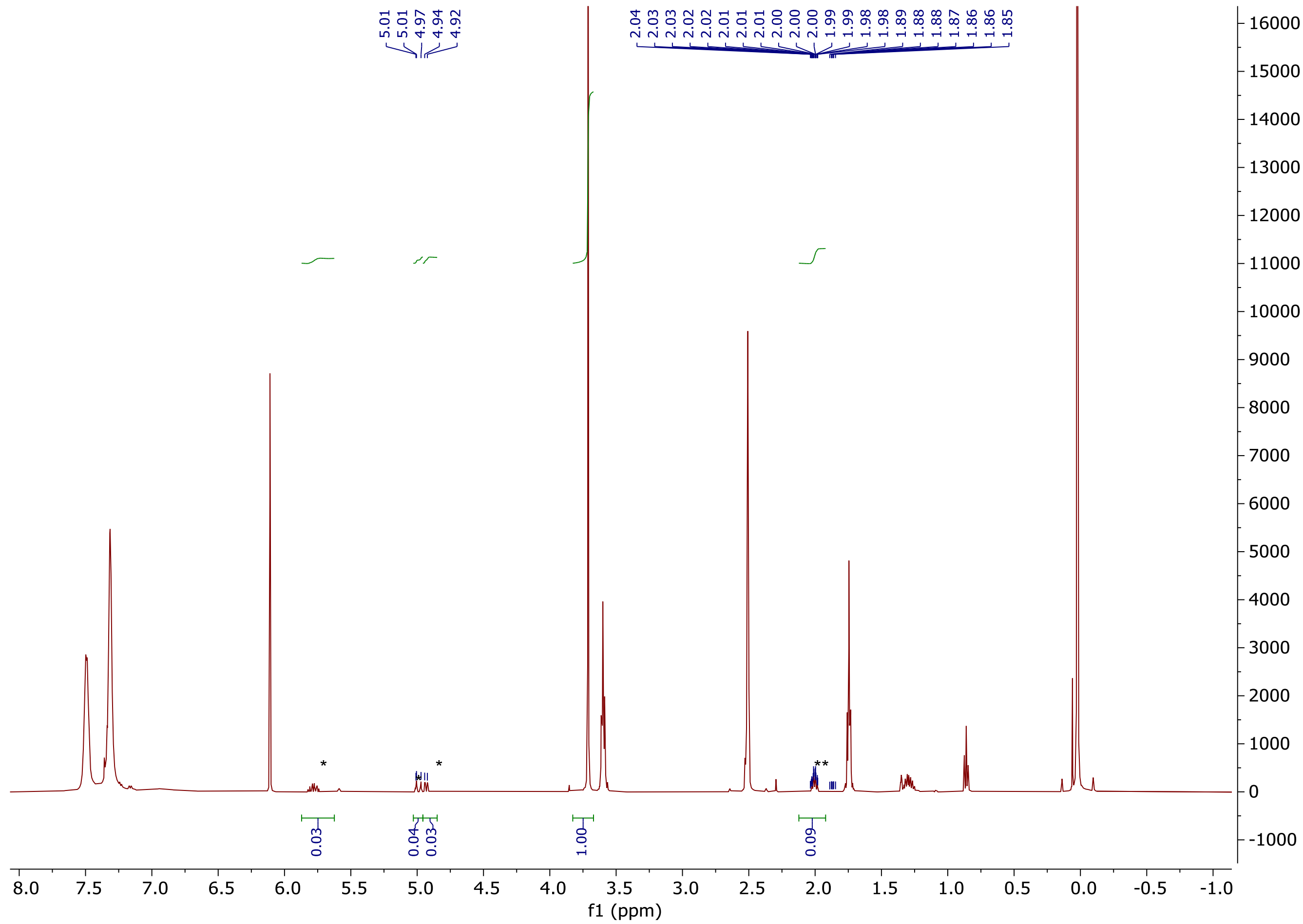

Figure S219: Hydrophosphination of 0.5 mmol 1-hexene with 2 equivalents of $PhPH_2$ in DMSO-d_6 using 5 mol% **1** under UV 360 nm irradiation - 48 h – ^1H NMR

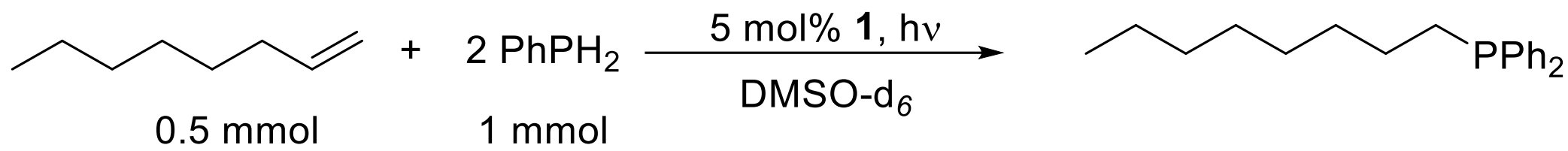

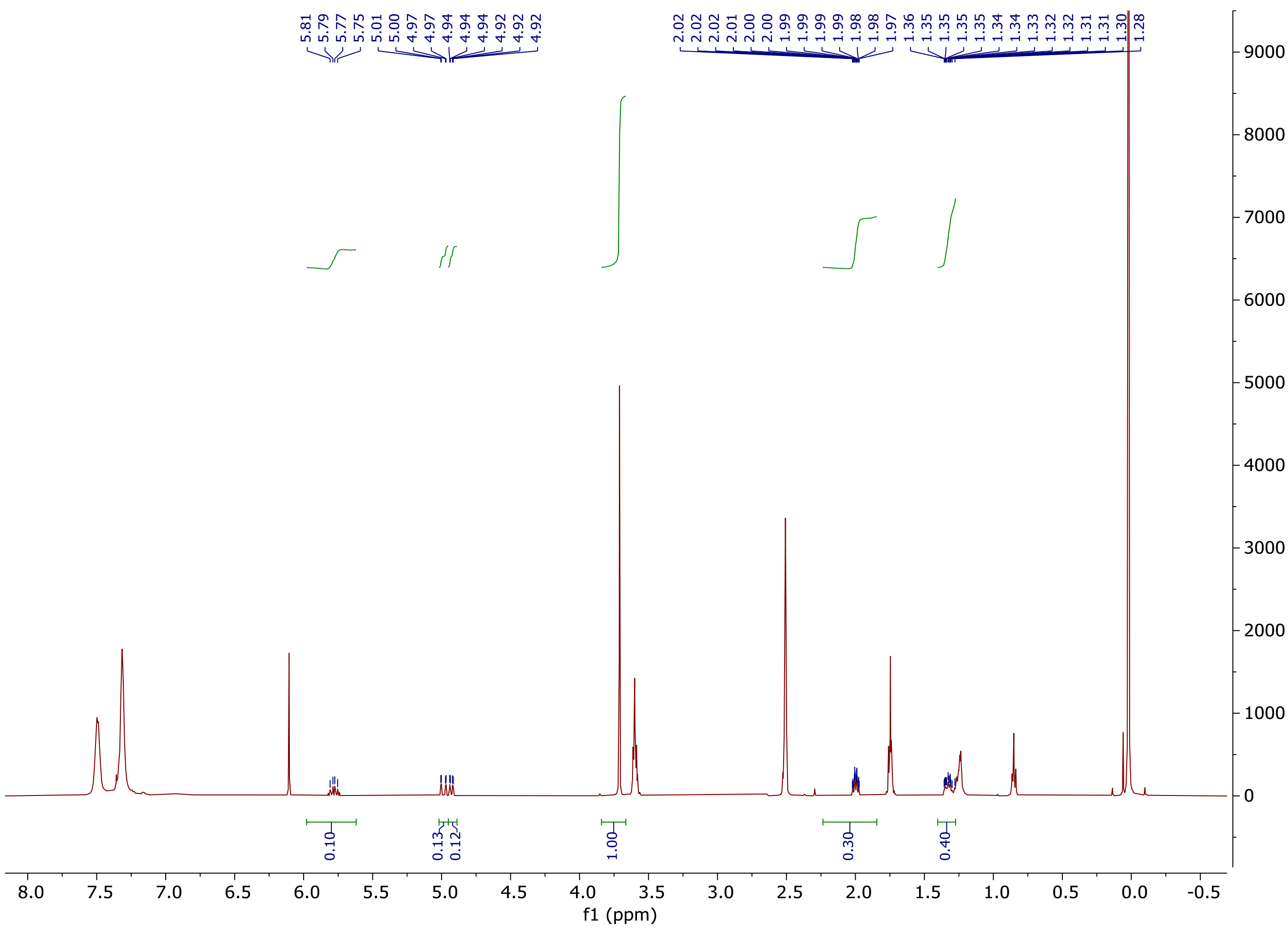

Figure S220: Hydrophosphination of 0.5 mmol 1-octene with 2 equivalents of $PhPH_2$ in DMSO-d_6 using 5 mol% **1** under UV 360 nm irradiation - 72h – ^{1}H NMR

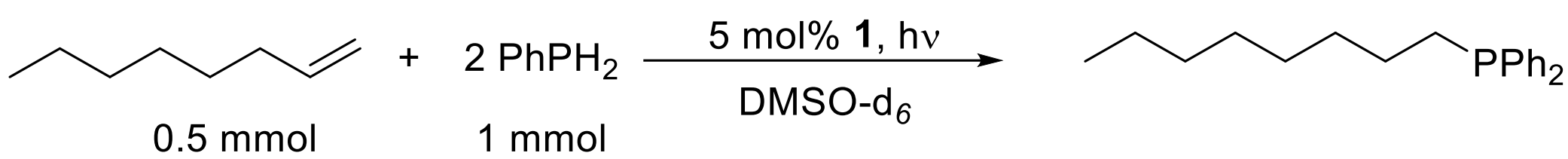

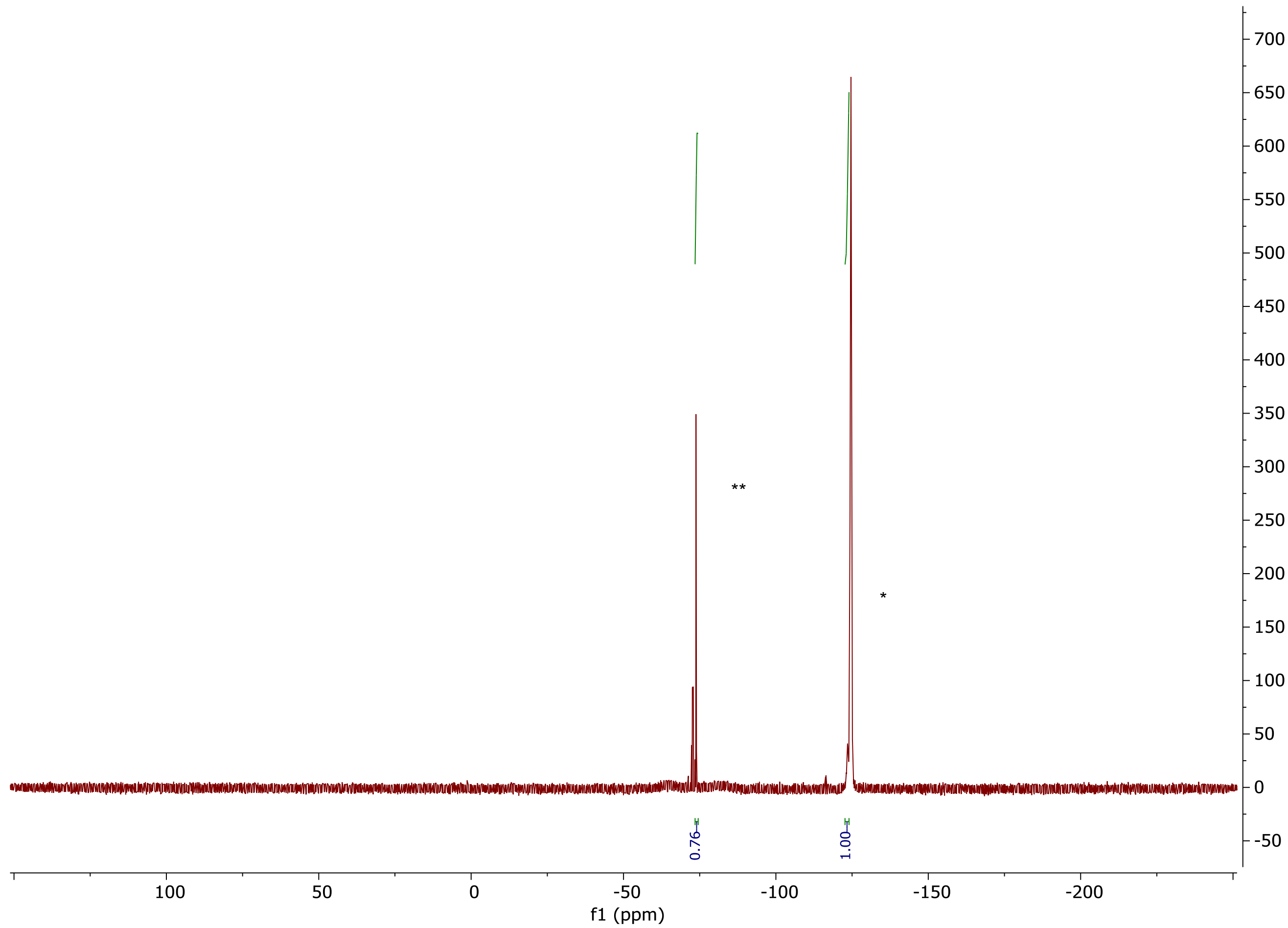

Figure S221: Hydrophosphination of 0.5 mmol 1- octene with 2 equivalents of $PhPH_2$ in $DMSO-d_6$ using 5 mol% **1** under UV 360 nm irradiation - 72 h – $^{31}P\{^1H\}$ NMR

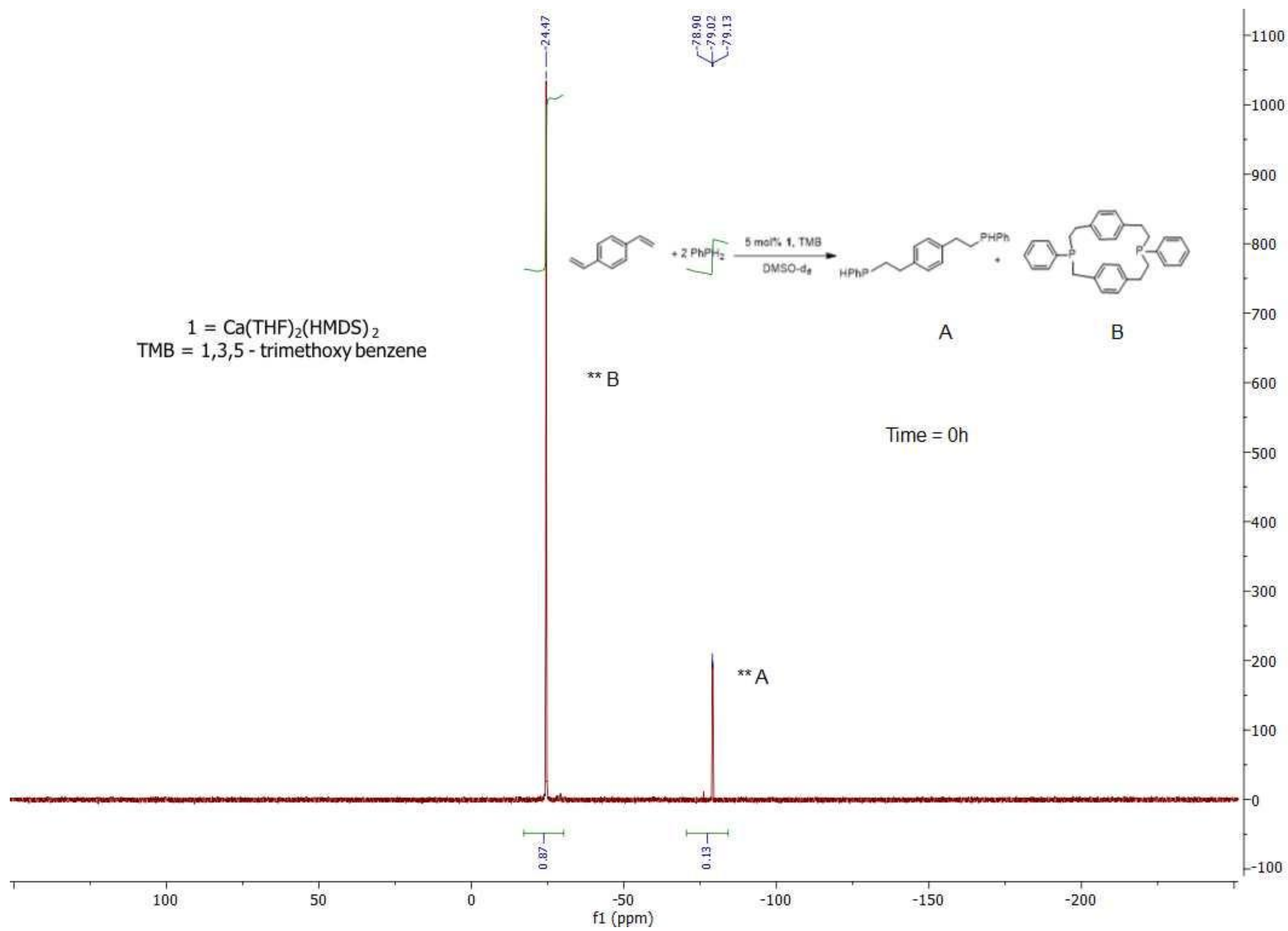

Figure S222: Hydrophosphination of 0.5 mmol of 1,4-divinyl benzene with 1 equivalent of $PhPH_2$ in DMSO-d_6 using 5 mol% **1** - 0 h – $^{31}P\{^1H\}$ NMR (compound A is 13% and compound B is 83%)

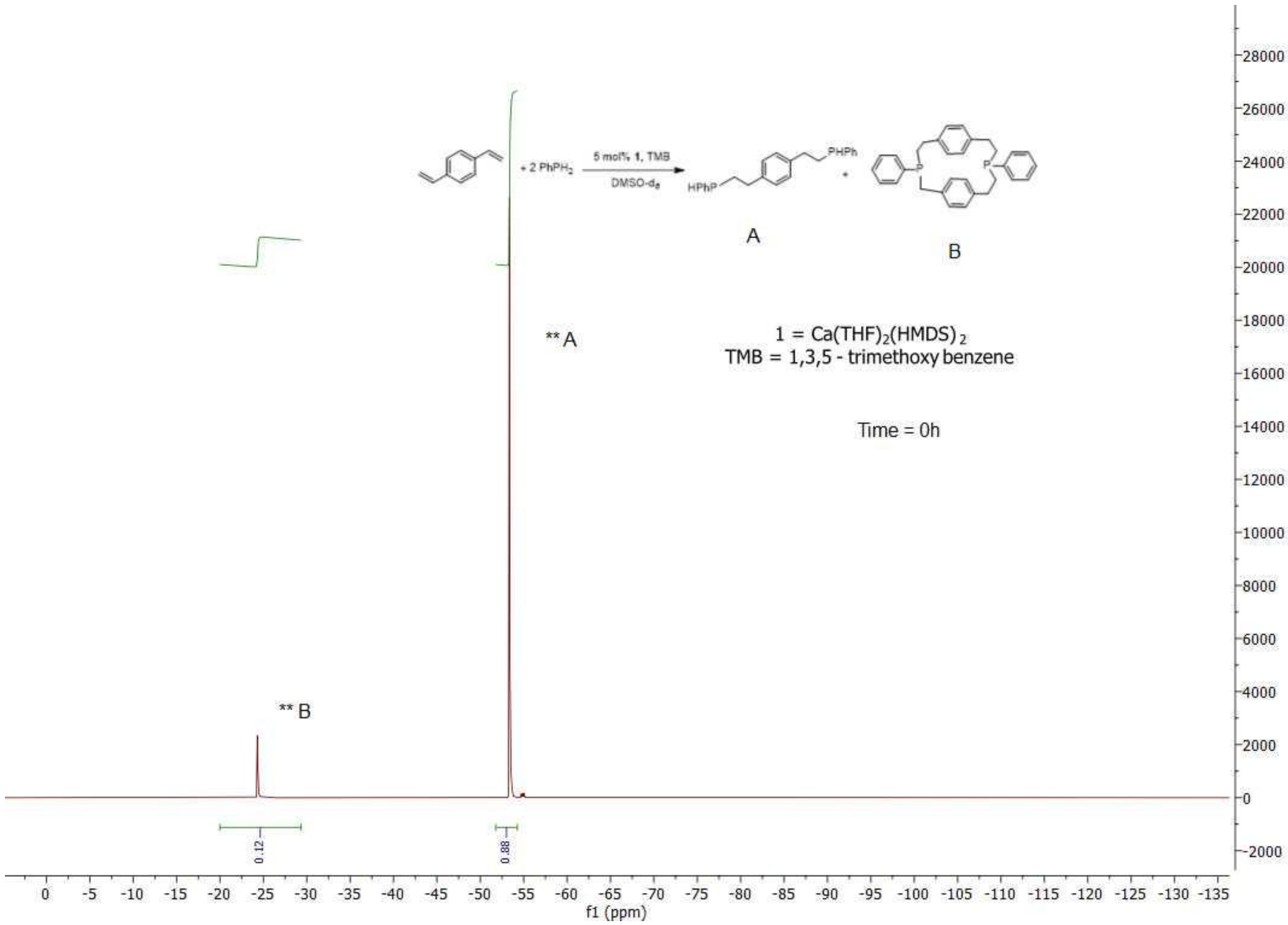

Figure S223: Hydrophosphination of 0.5 mmol of 1,4-divinyl benzene with 2 equivalents of $PhPH_2$ in DMSO-d_6 using 5 mol% **1** - 0 h – $^{31}P\{^1H\}$ NMR (compound A is 88% and compound B is 12%)

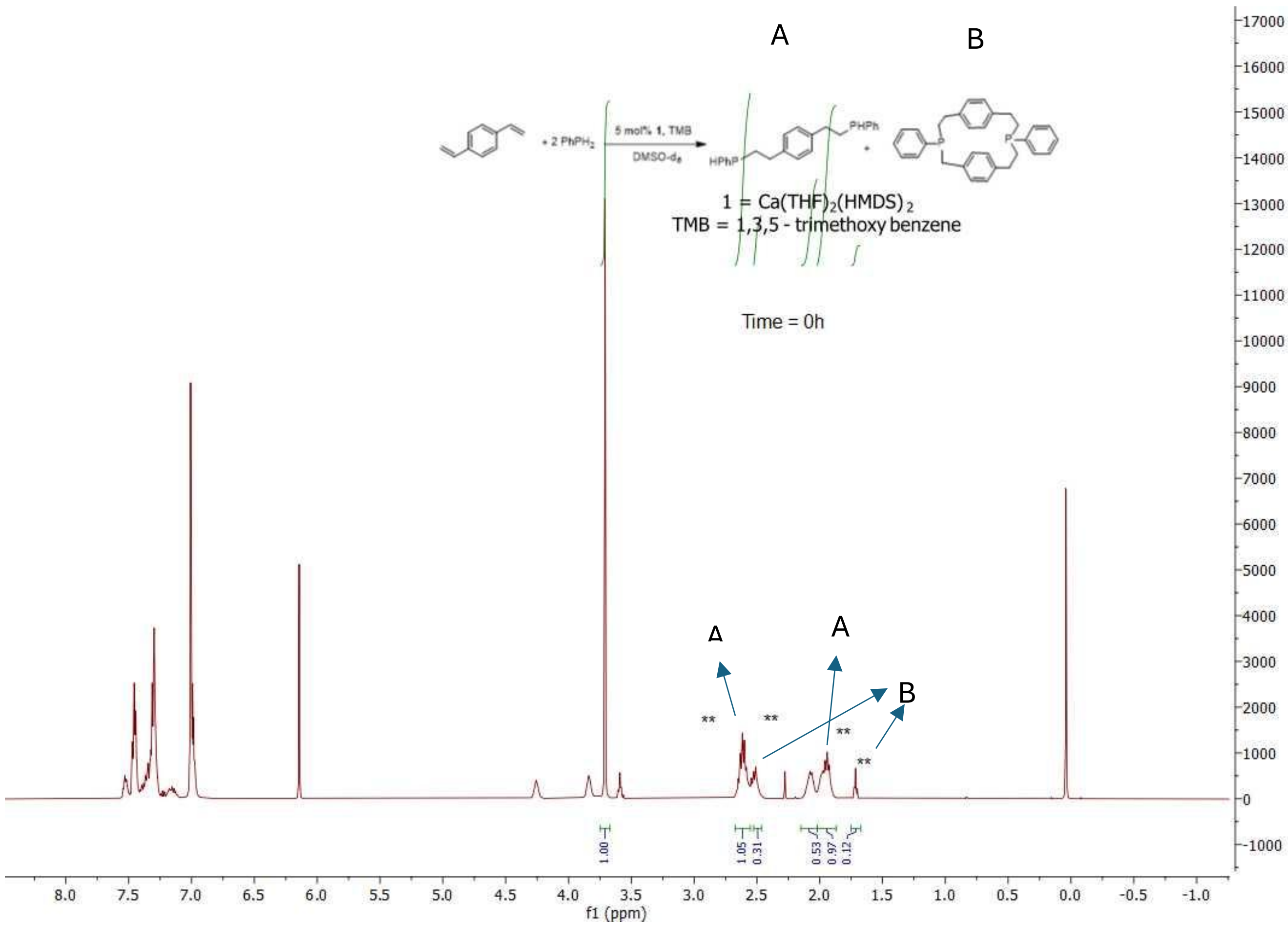

Figure S224: Hydrophosphination of 0.5 mmol of 1,4-divinyl benzene with 2 equivalents of $PhPH_2$ in DMSO-d_6 using 5 mol% **1** - 0 h – ^{1}H NMR (compound A is 88% and compound B is 12%)

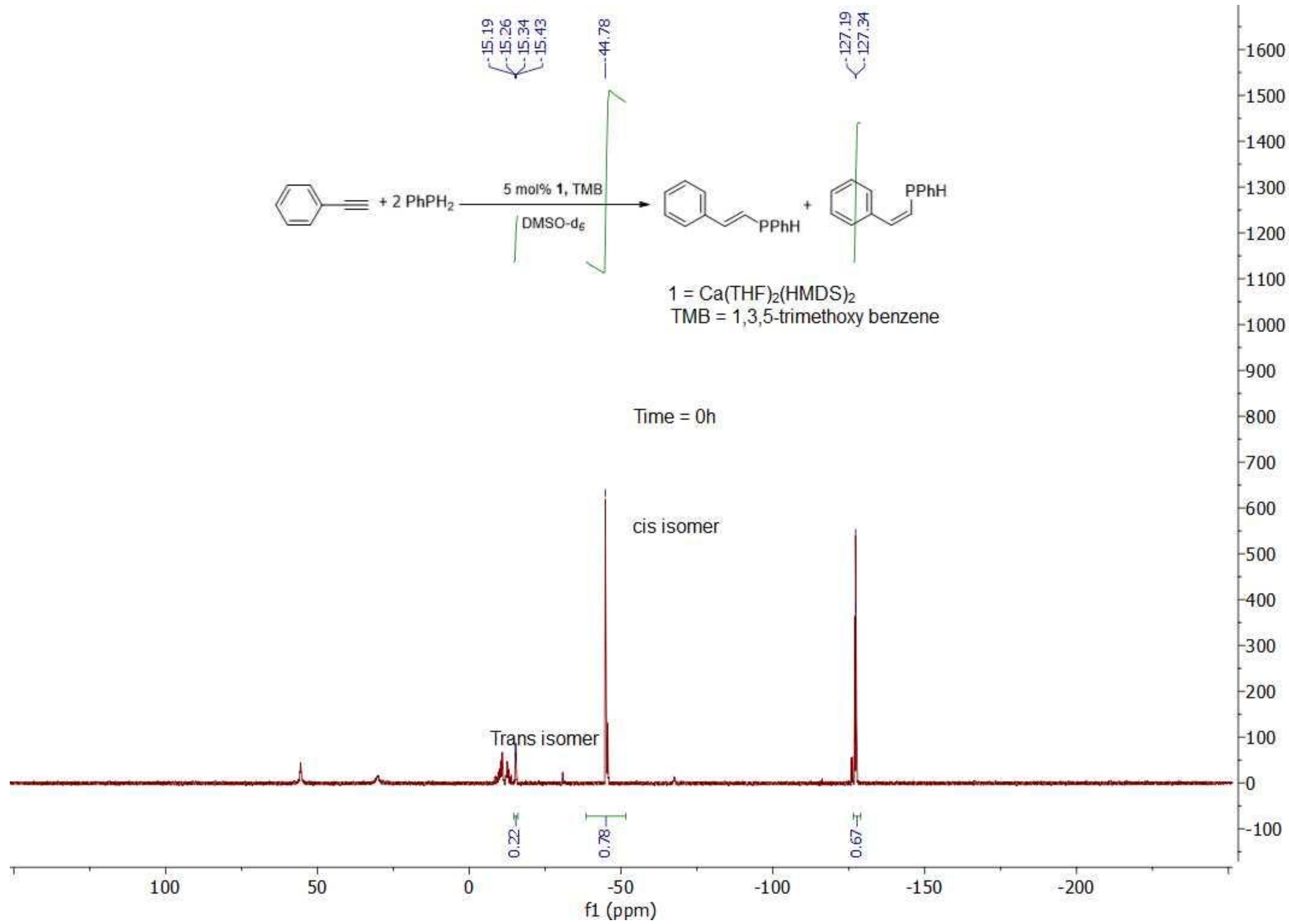

Figure S225: Hydrophosphination of 0.5 mmol of phenyl acetylene with 2 equivalents $PhPH_2$ in DMSO-d_6 using 5 mol% **1** - 0 h – $^{31}P\{^1H\}$ NMR

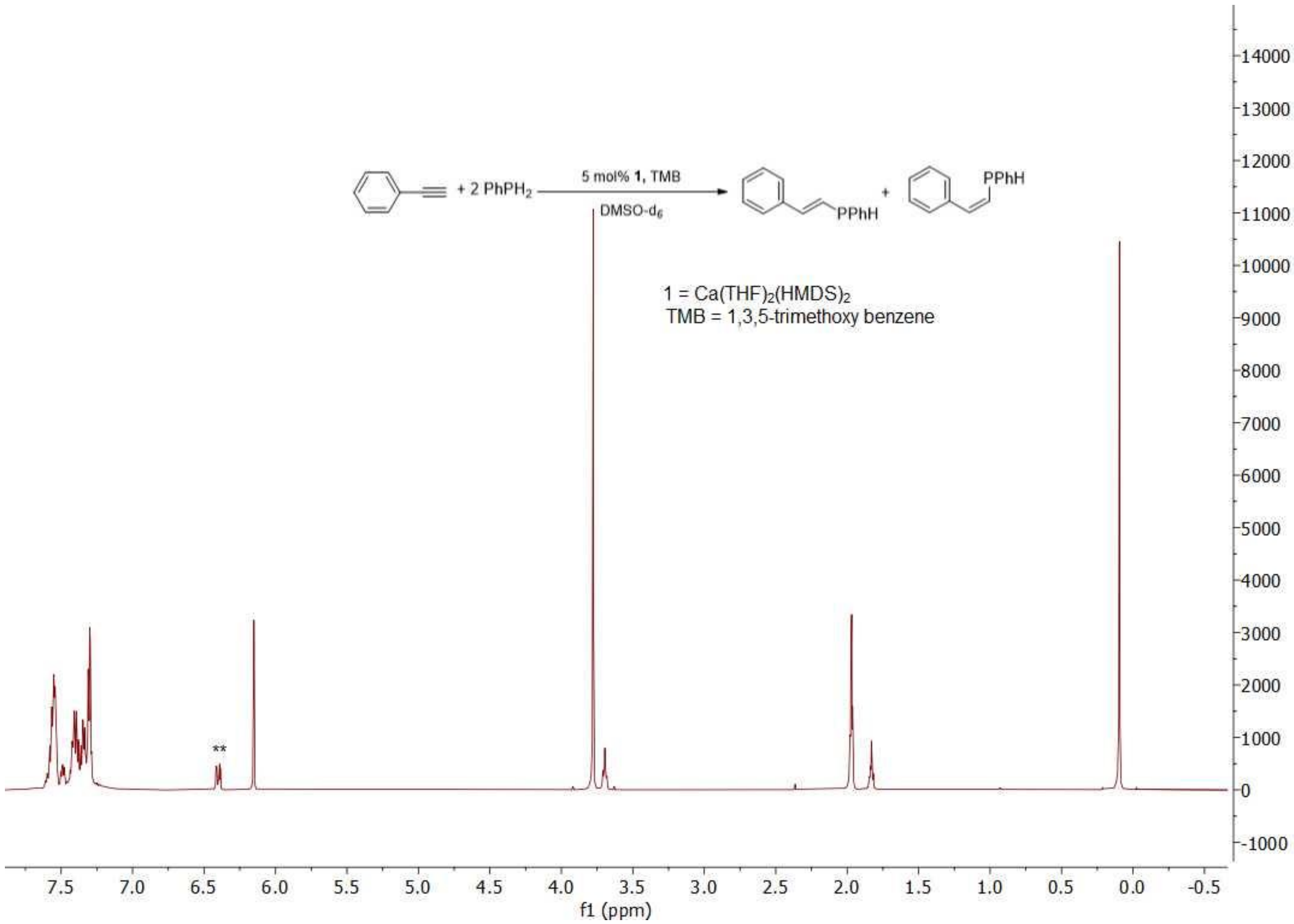

Figure S226: Hydrophosphination of 0.5 mmol of phenyl acetylene with 2 equivalents $PhPH_2$ in DMSO-d_6 using 5 mol% **1** - 0 h – ^{1}H NMR

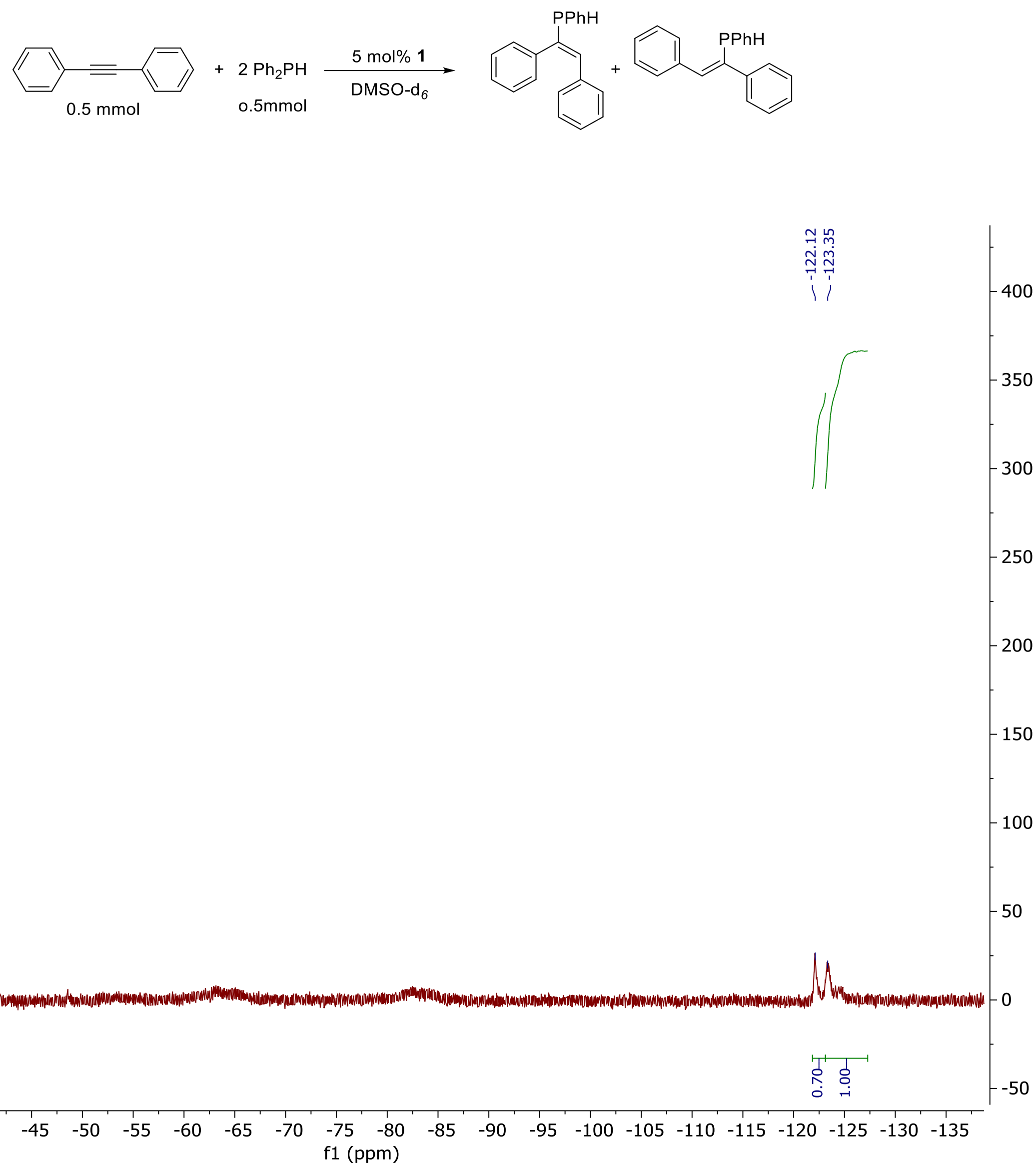

Figure S227: Hydrophosphination of 0.5 mmol of diphenyl acetylene with 2 equivalents $PhPH_2$ in DMSO-d_6 using 5 mol% **1** - 0 h – ^{1}H NMR

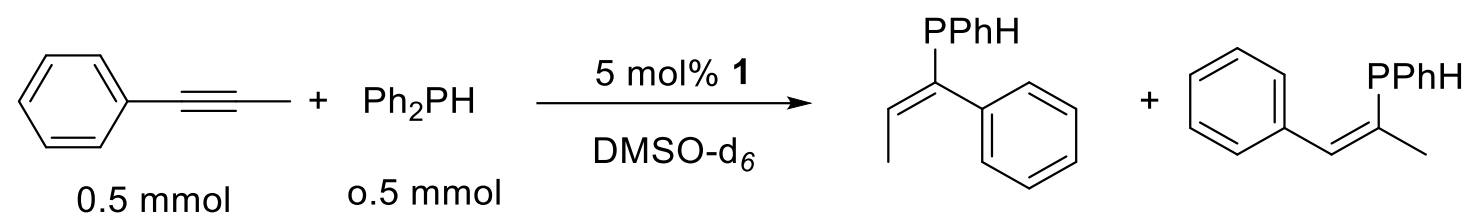

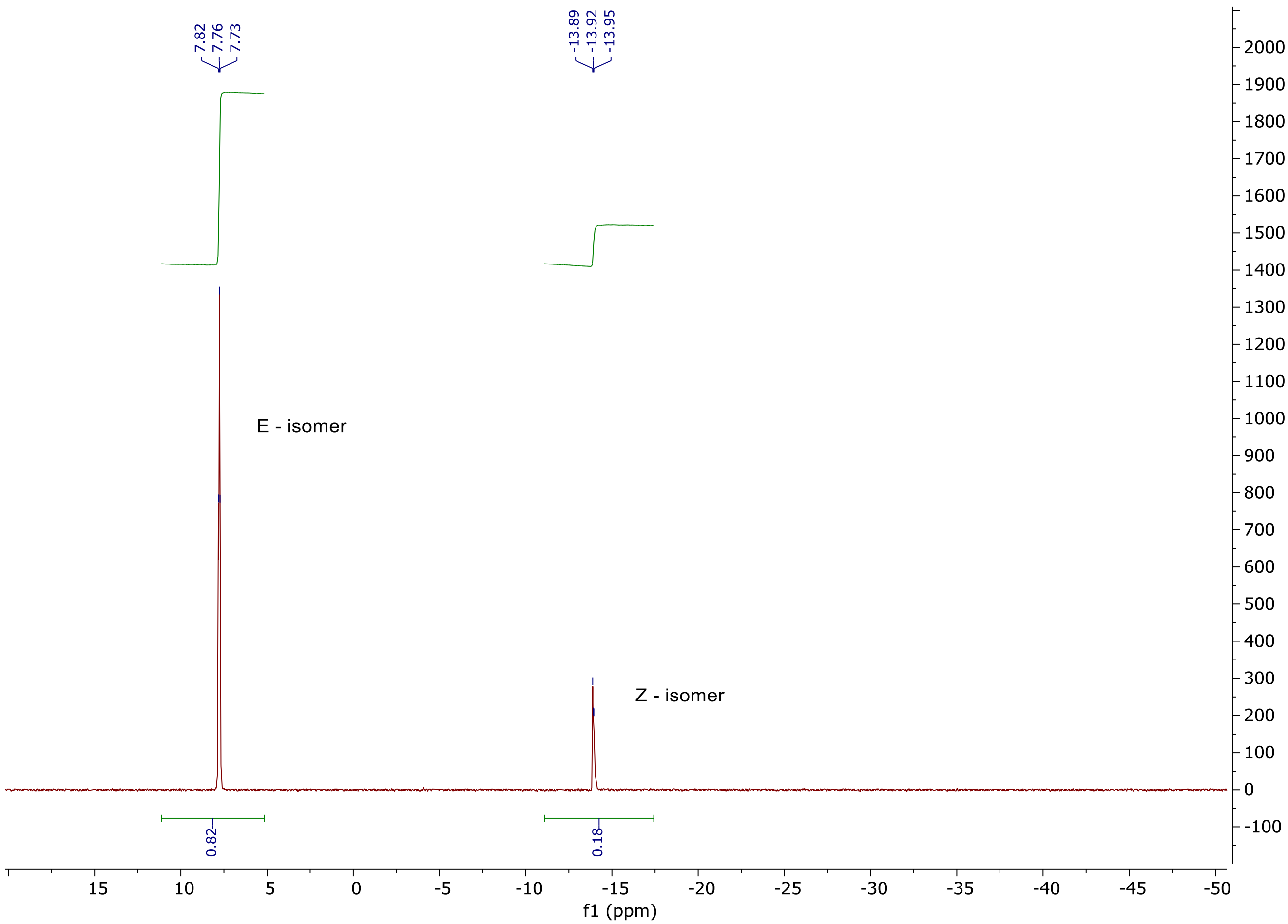

Figure S228: Hydrophosphination of 0.5 mmol of 1-phenyl 1-propyne with 1 equivalents $PhPH_2$ in DMSO-d_6 using 5 mol% **1** - 16 h – $^{31}P\{^1H\}$ NMR

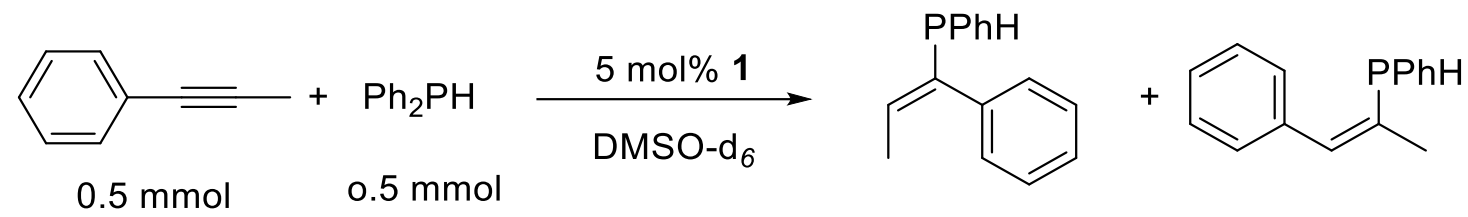

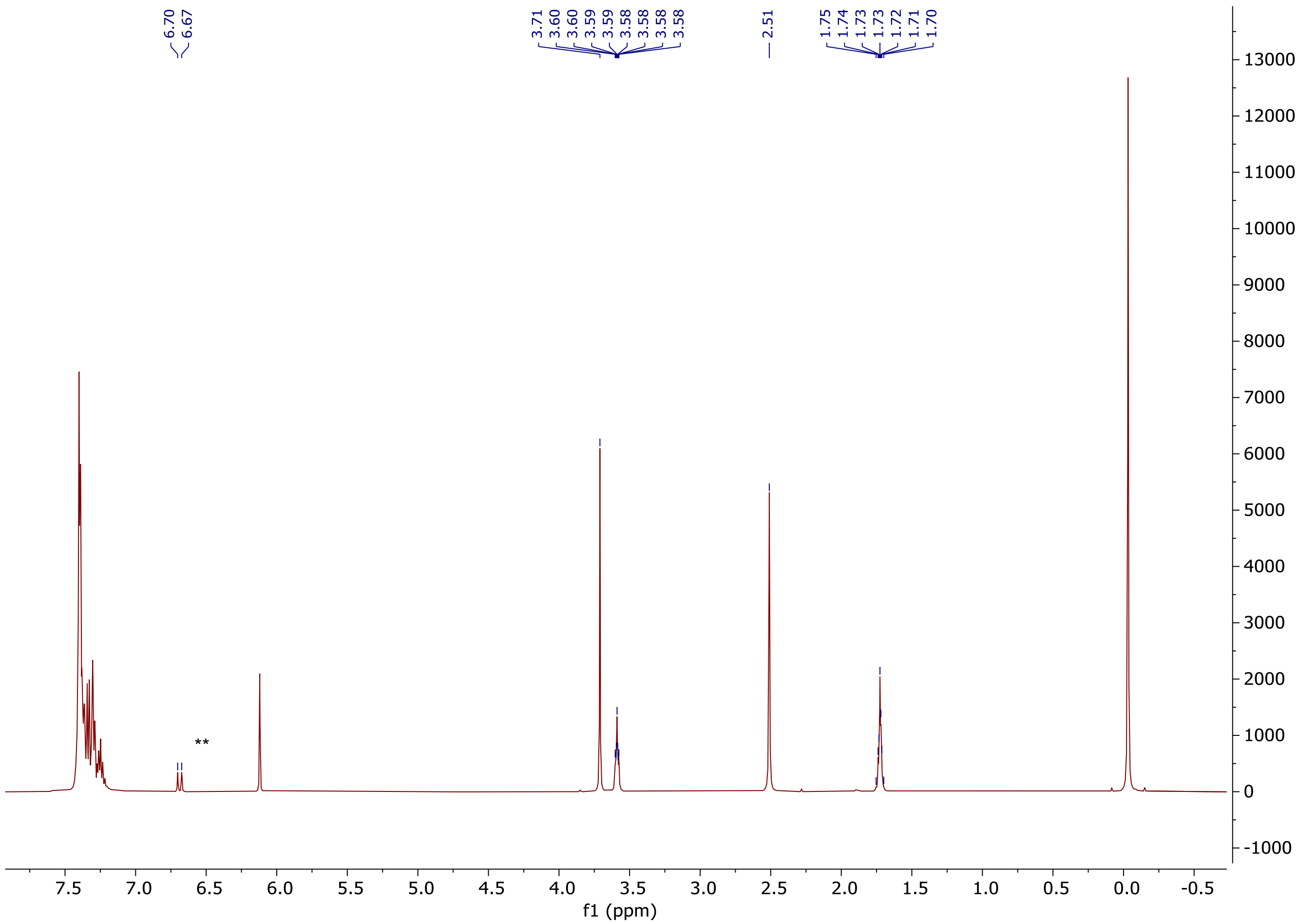

Figure S229: Hydrophosphination of 0.5 mmol of 1-phenyl 1-propyne with 1 equivalents $PhPH_2$ in DMSO-d_6 using 5 mol% **1** – 16 h – ^{1}H NMR

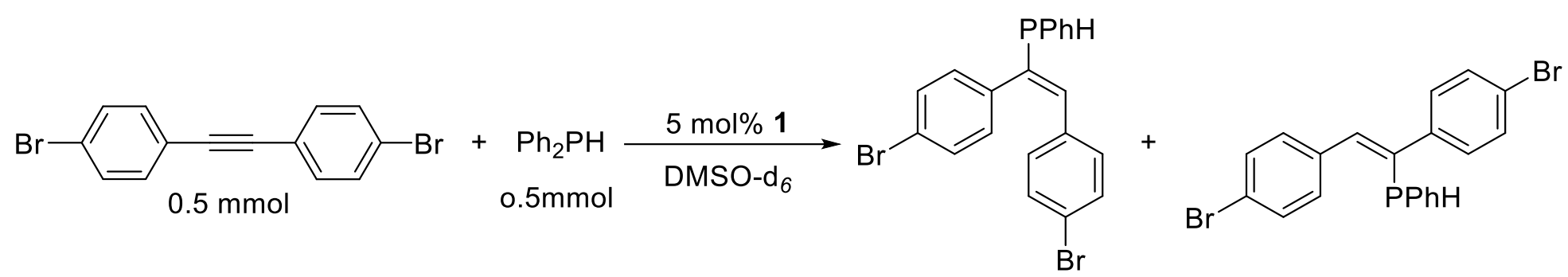

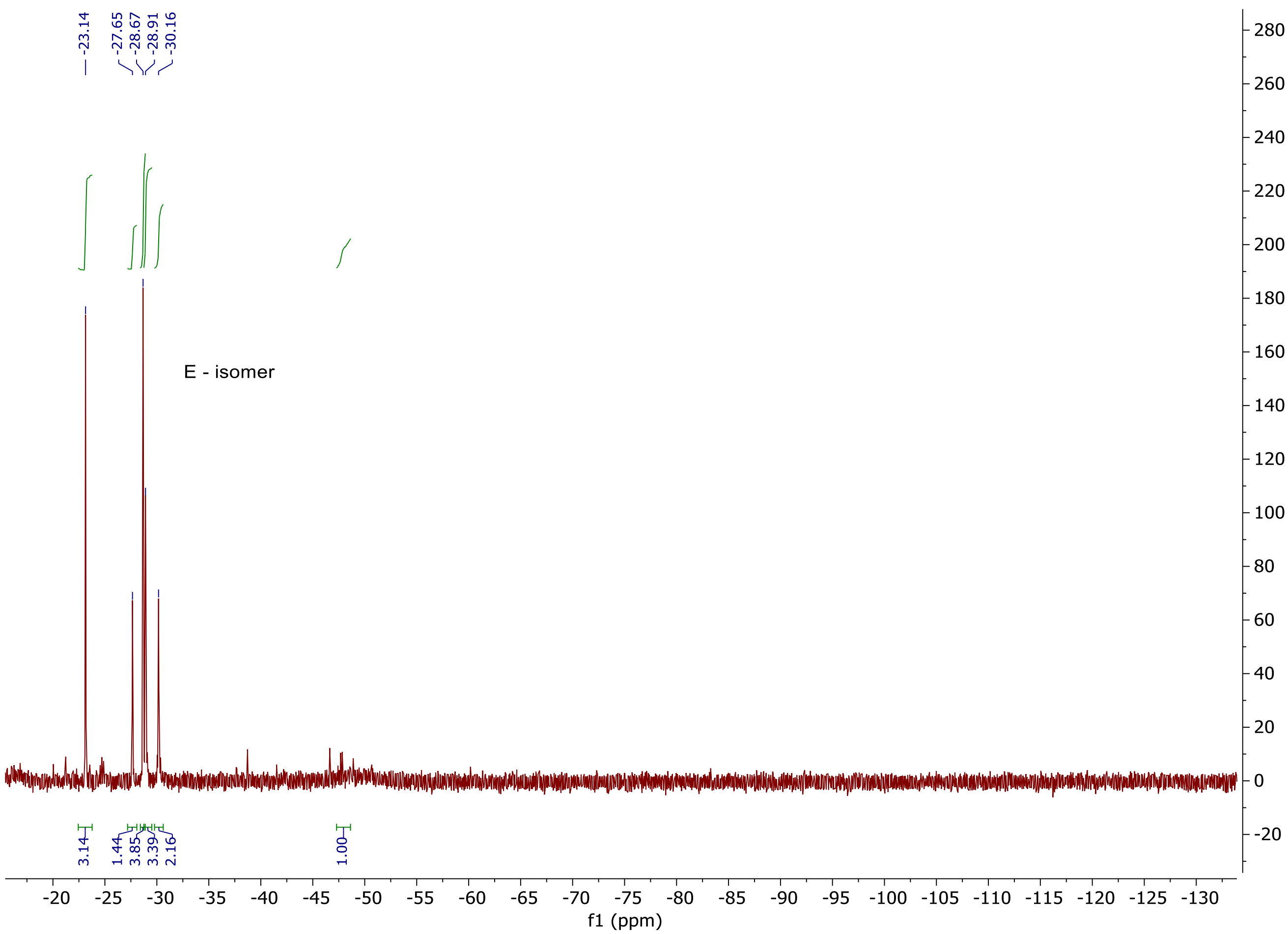

Figure S230: Hydrophosphination of 0.5 mmol of bis (4-bromo phenyl acetylene) with equimolar $PhPH_2$ in DMSO-d_6 using 5 mol% **1** - 0 h – ^{1}H NMR

Hydrophosphination of unsaturated compounds with cyclohexyl phosphine in DMSO-d_6 using 5 mol% **1**

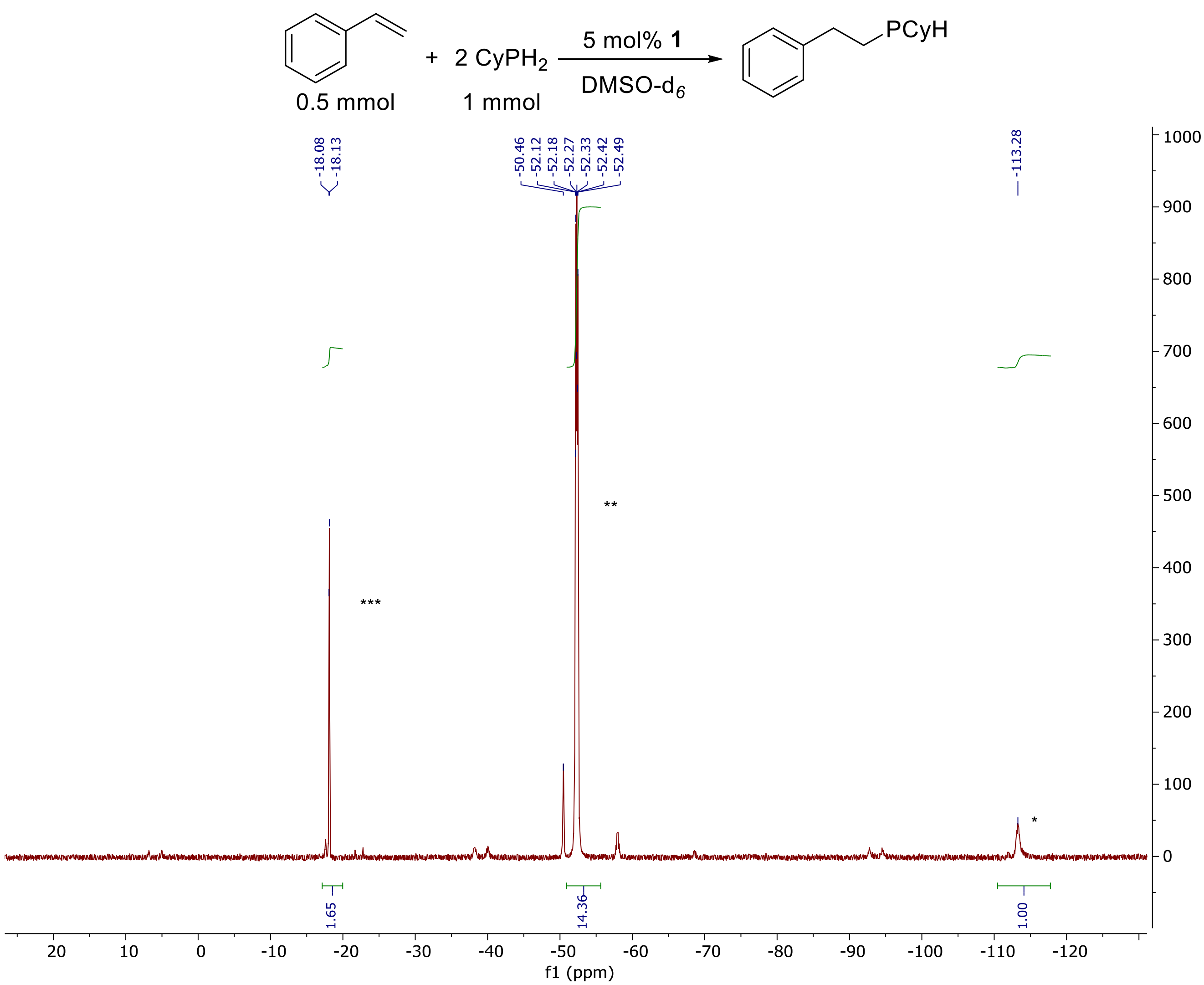

Figure S231: Hydrophosphination of 0.5 mmol of styrene with 2 equivalents $CyPH_2$ in DMSO-d_6 using 5 mol% **1** - 0 h – $^{31}P\{^1H\}$ NMR

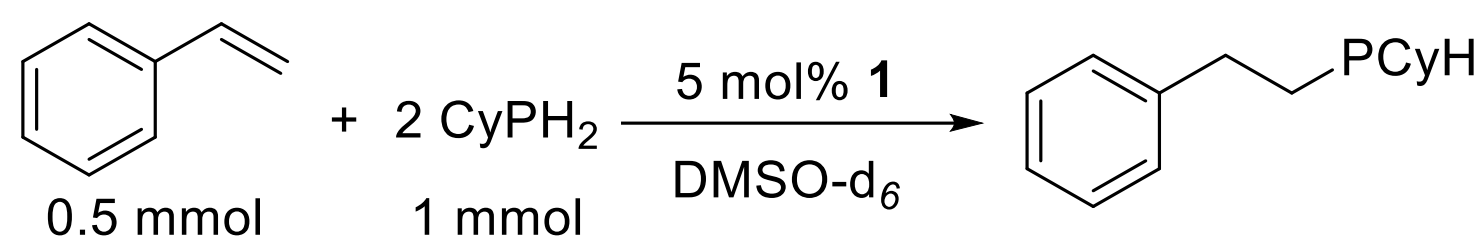

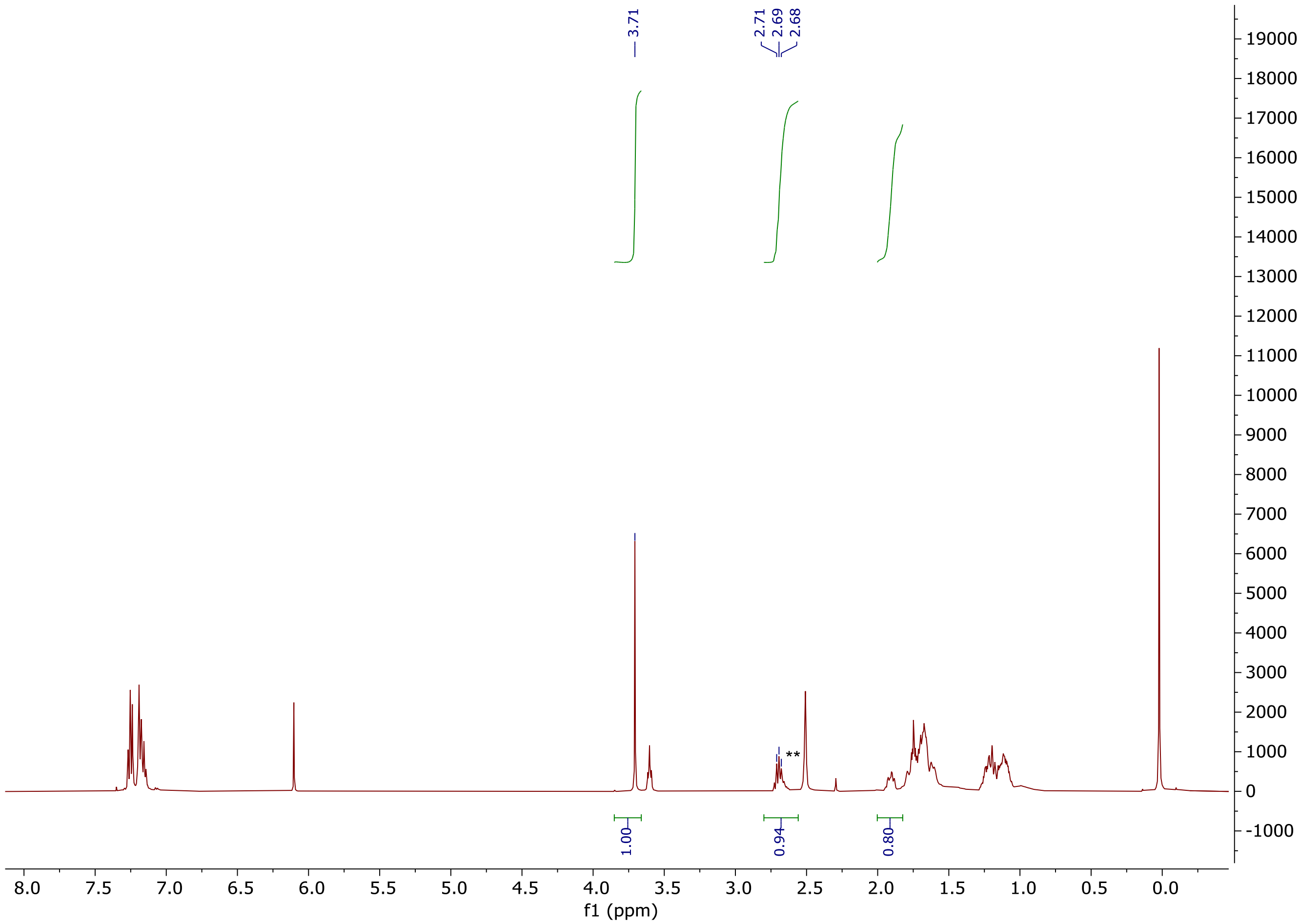

Figure S232: Hydrophosphination of 0.5 mmol of styrene with 2 equivalents $CyPH_2$ in DMSO-d_6 using 5 mol% **1** - 0 h – ^{1}H NMR

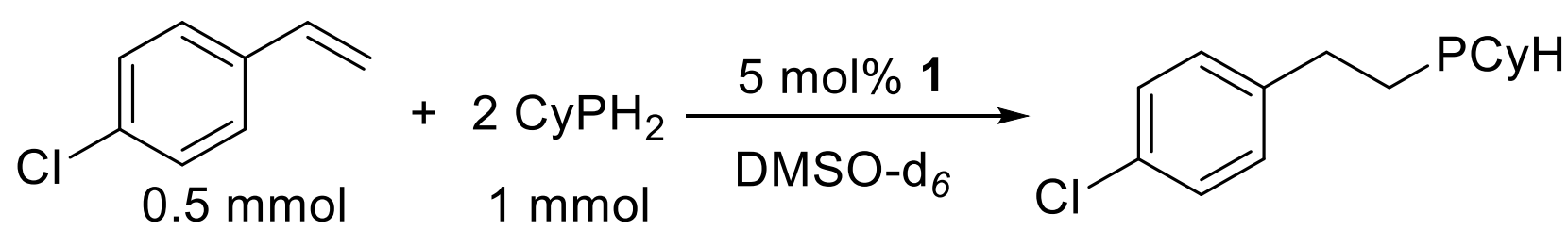

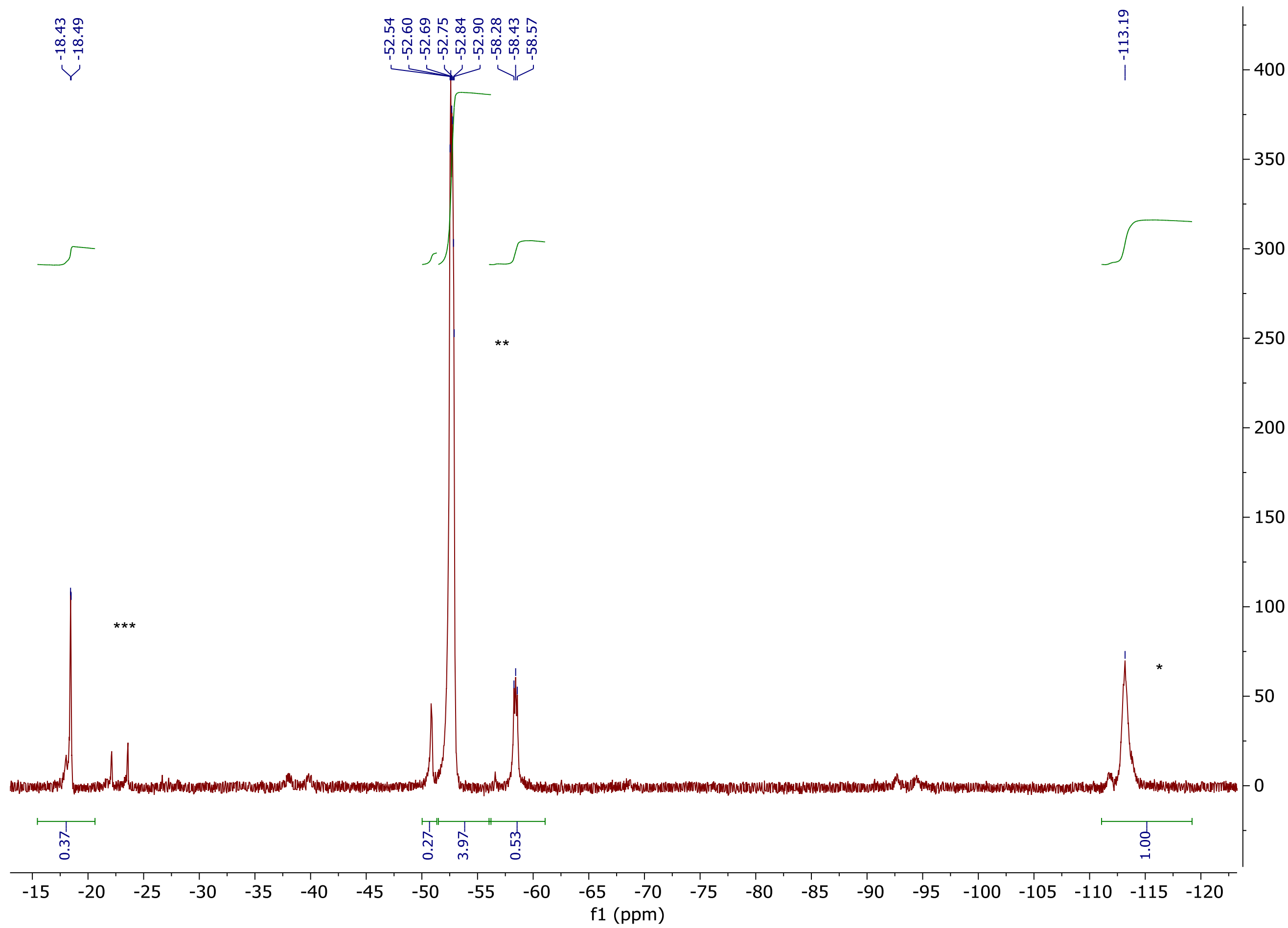

Figure S233: Hydrophosphination of 0.5 mmol of 4-chloro styrene with 2 equivalents $CyPH_2$ in DMSO-d_6 using 5 mol% **1** - 0 h – $^{31}P\{^1H\}$ NMR

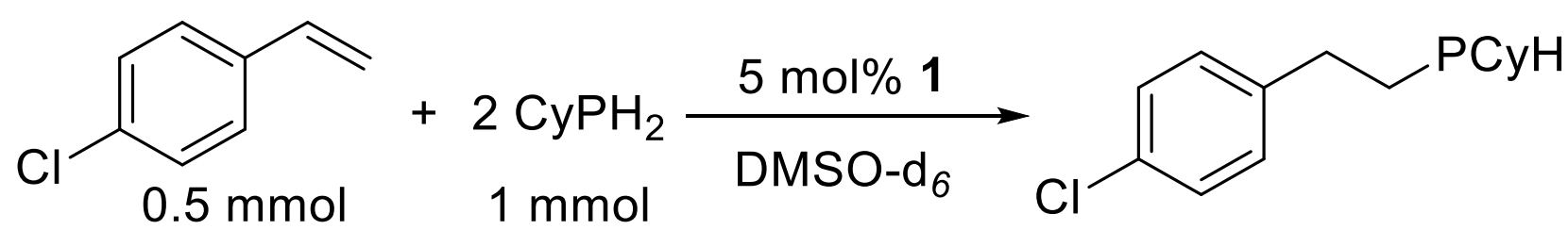

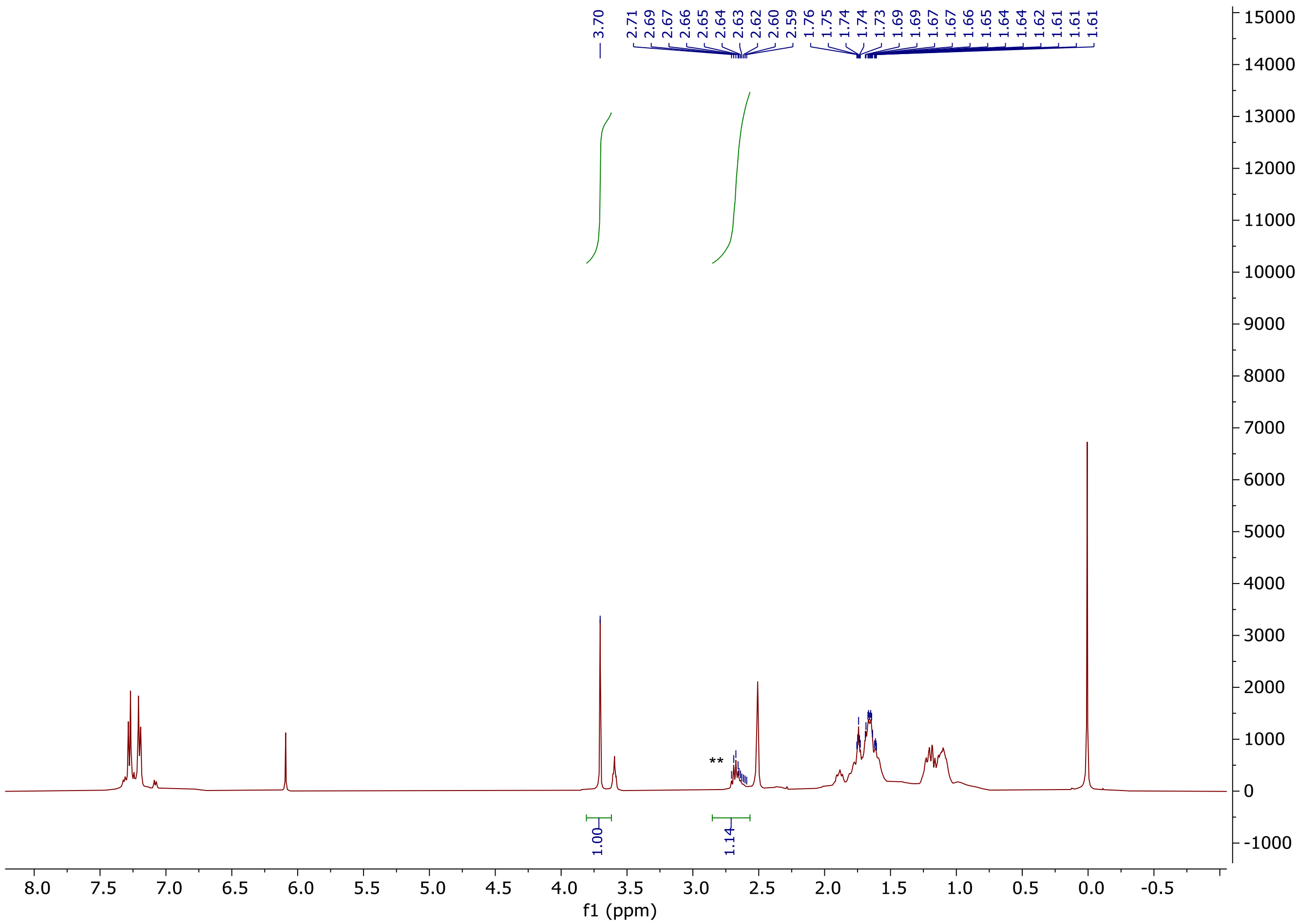

Figure S234: Hydrophosphination of 0.5 mmol of 4-chloro styrene with 2 equivalents $CyPH_2$ in DMSO-d_6 using 5 mol% **1** - 0 h – ^{1}H NMR

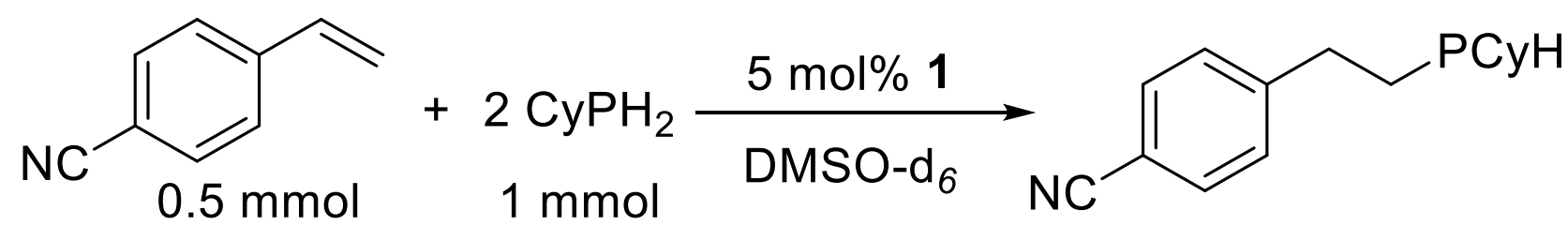

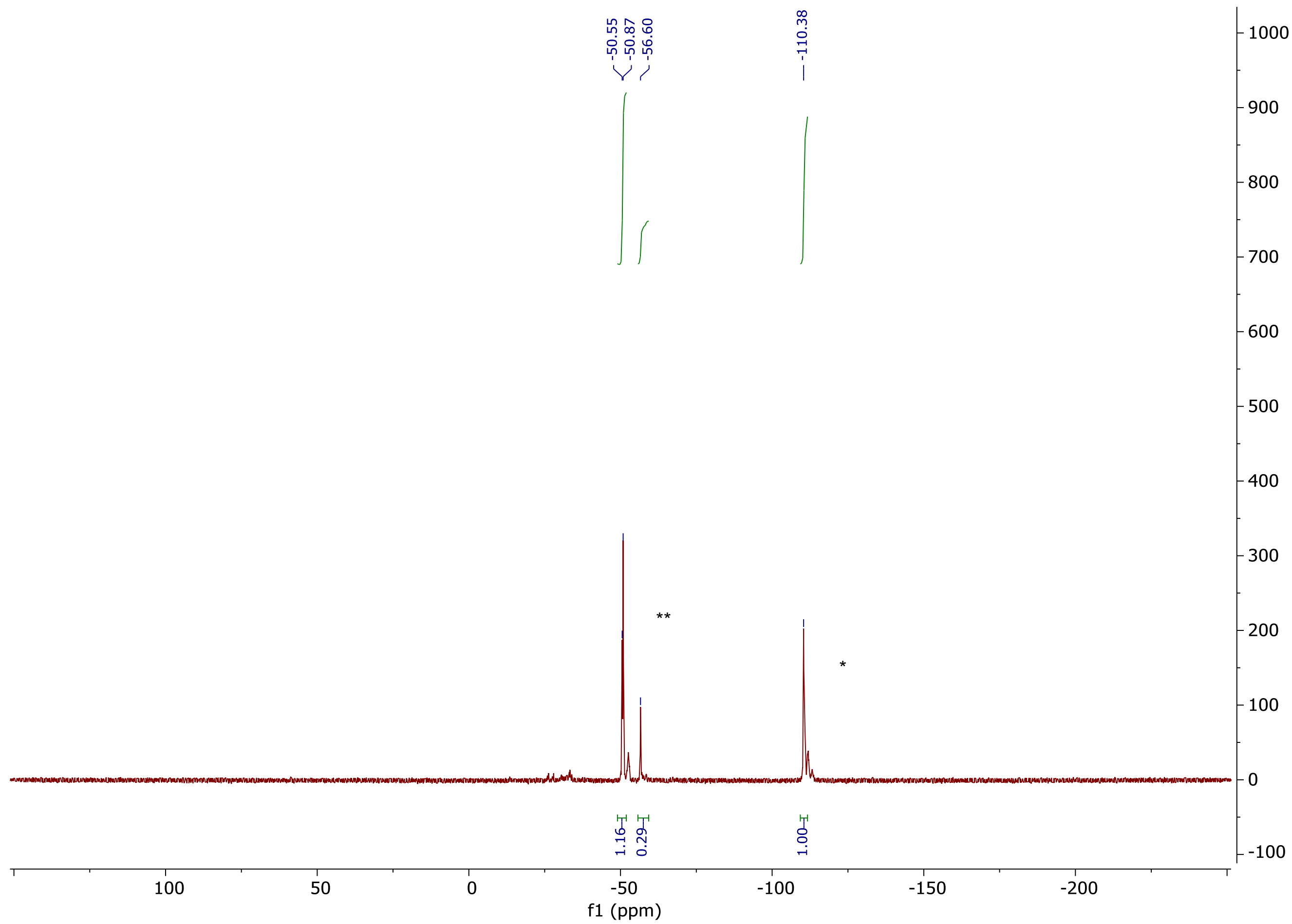

Figure S235: Hydrophosphination of 0.5 mmol of 4-cyano styrene with 2 equivalents $CyPH_2$ in DMSO-d_6 using 5 mol% **1** - 0 h – $^{31}P\{^1H\}$ NMR

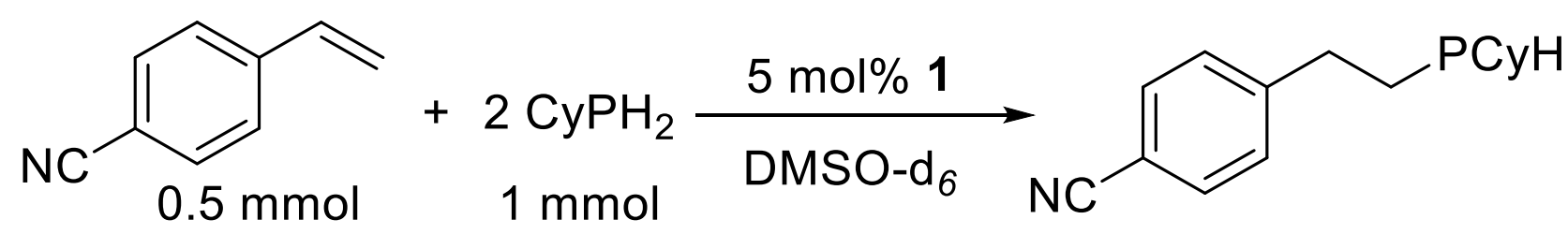

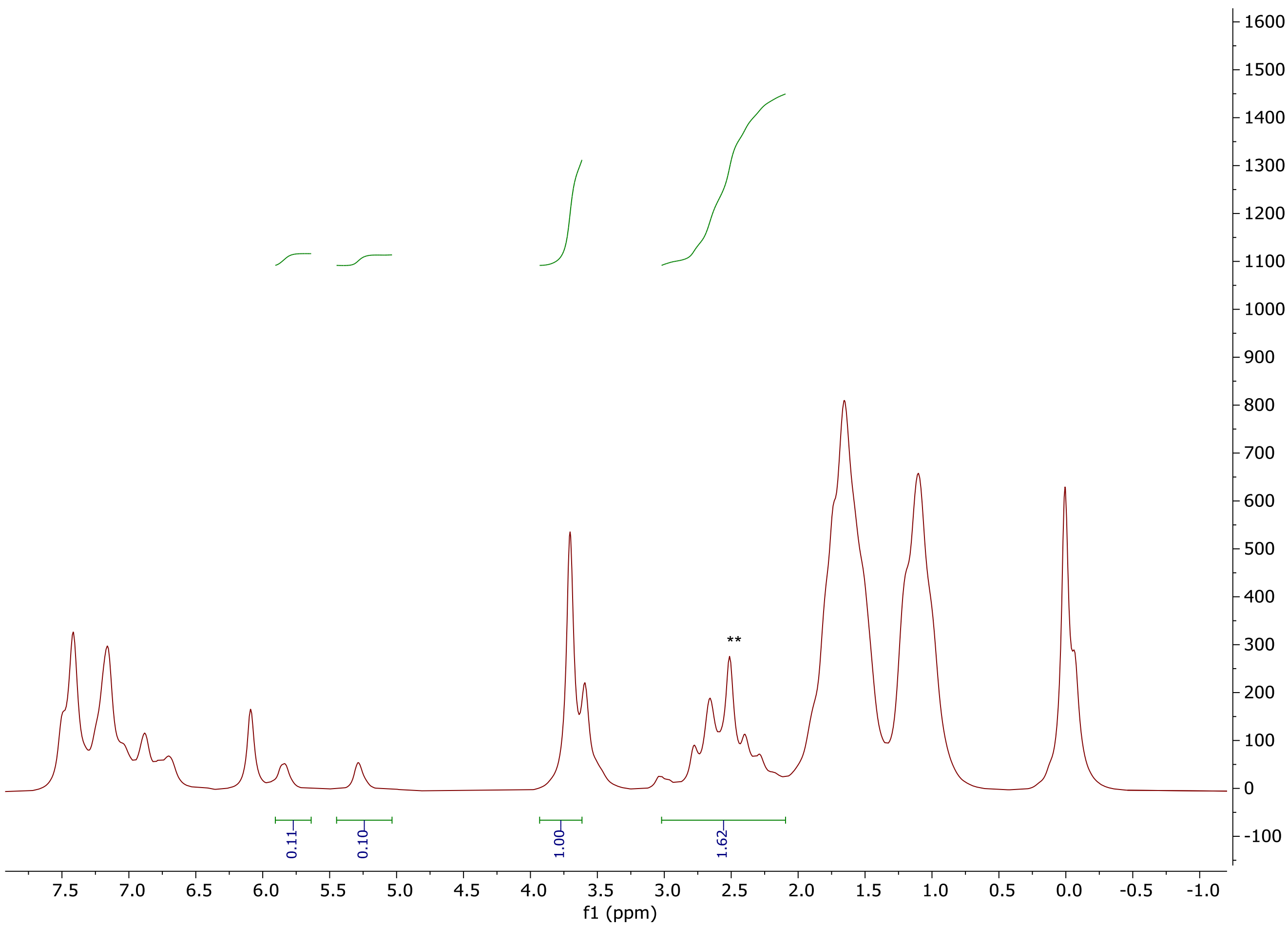

Figure S236: Hydrophosphination of 0.5 mmol of 4-cyano styrene with 2 equivalents $CyPH_2$ in DMSO-d_6 using 5 mol% **1** - 0 h – ^{1}H NMR

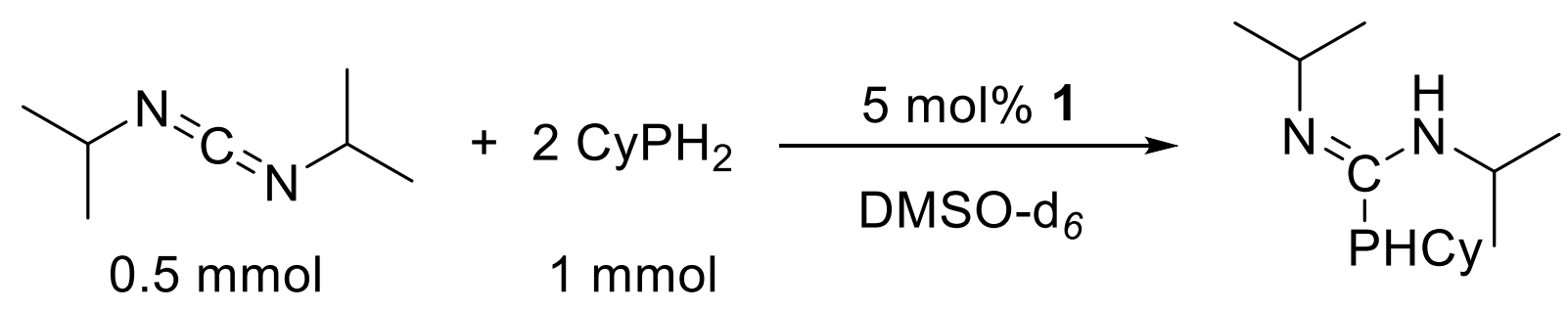

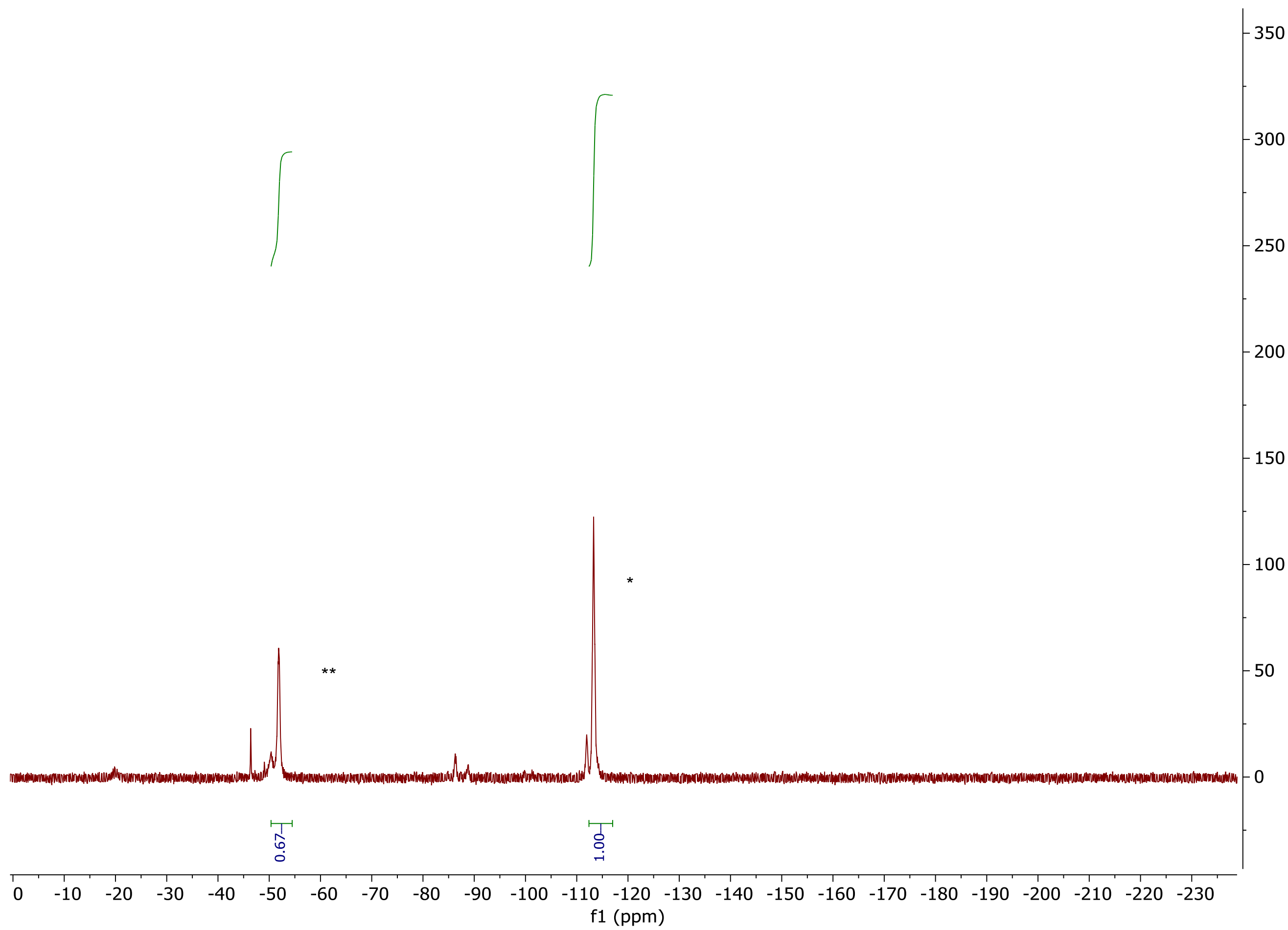

Figure S237: Hydrophosphination of 0.5 mmol of N,N'-diisopropylcarbodiimide with 2 equivalents $CyPH_2$ in DMSO-d_6 using 5 mol% **1** - 0 h – $^{31}P\{^1H\}$ NMR

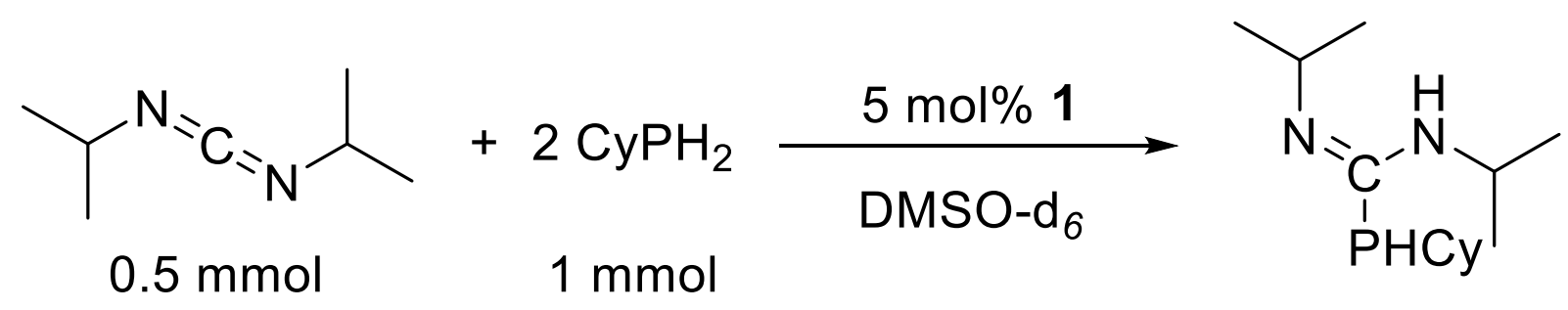

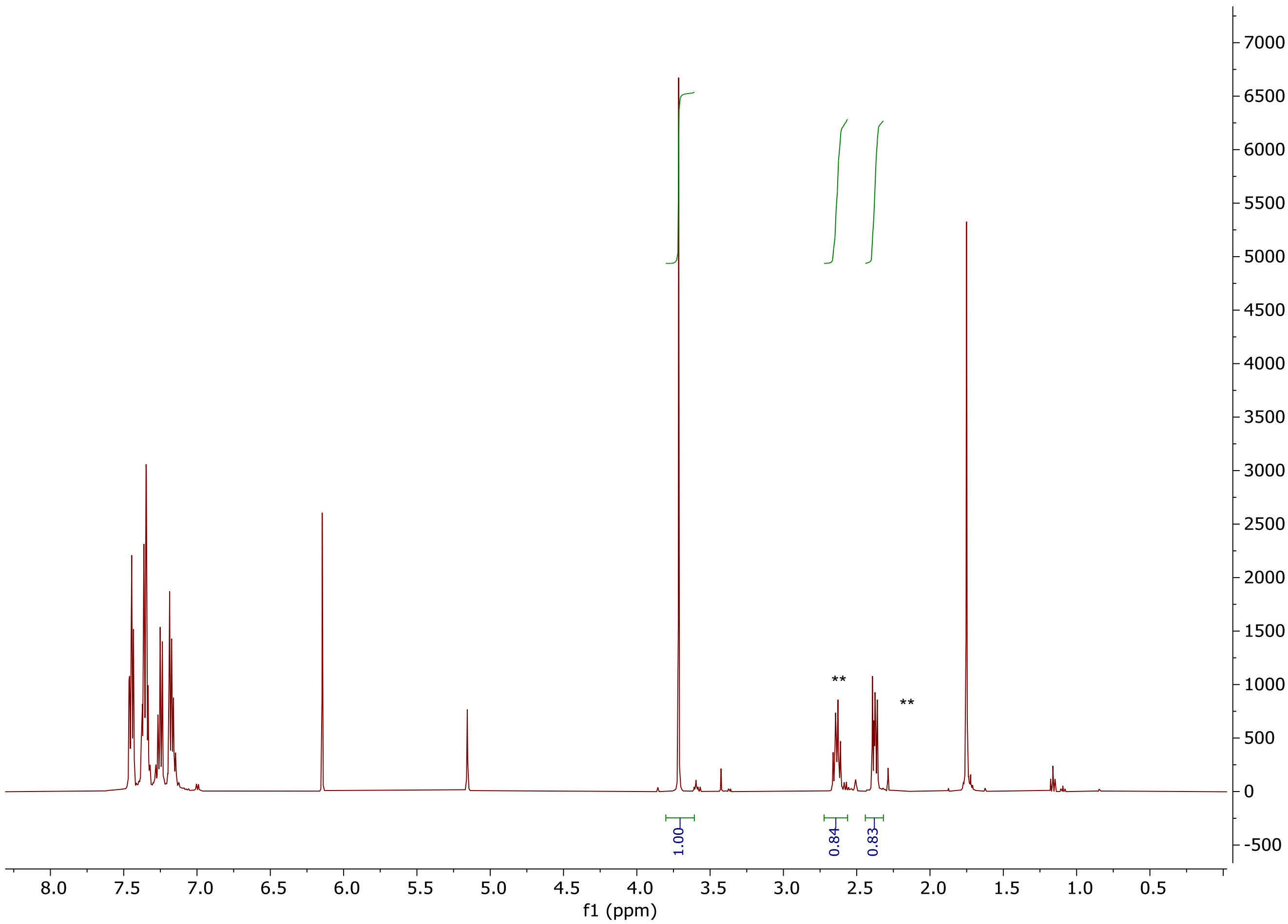

Figure S238: Hydrophosphination of 0.5 mmol of N,N'-diisopropylcarbodiimide with 2 equivalents $CyPH_2$ in DMSO-d_6 using 5 mol% **1** - 0 h – ^1H NMR

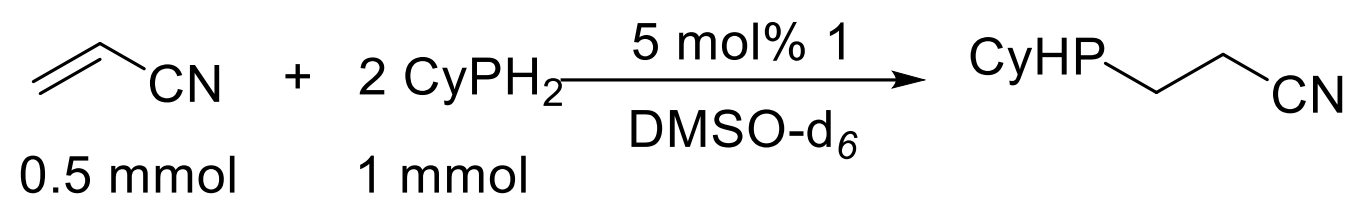

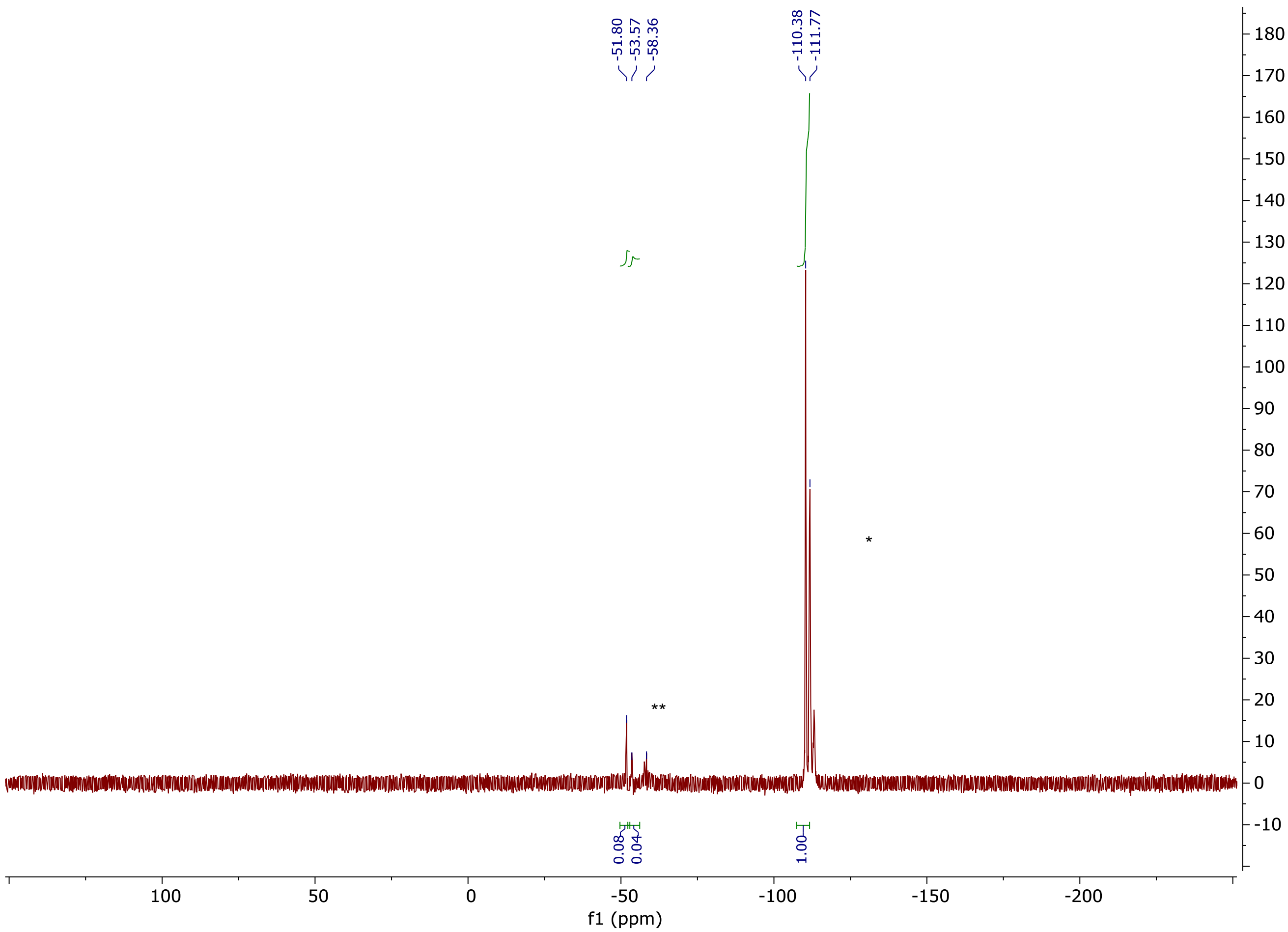

Figure S239: Hydrophosphination of 0.5 mmol of acrylonitrile with 2 equivalents $CyPH_2$ in DMSO-d_6 using 5 mol% **1** - 0 h – $^{31}P\{^1H\}$ NMR

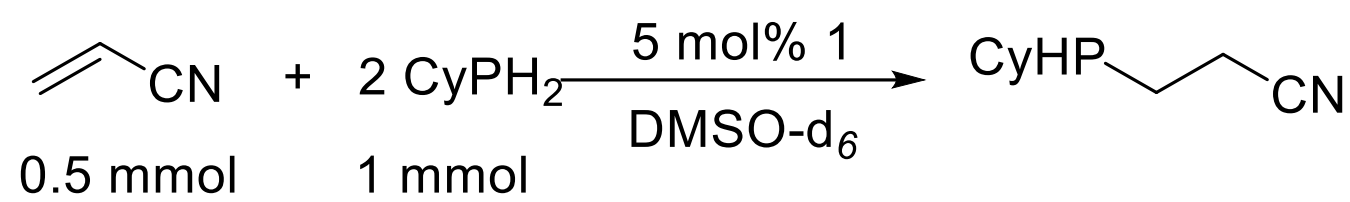

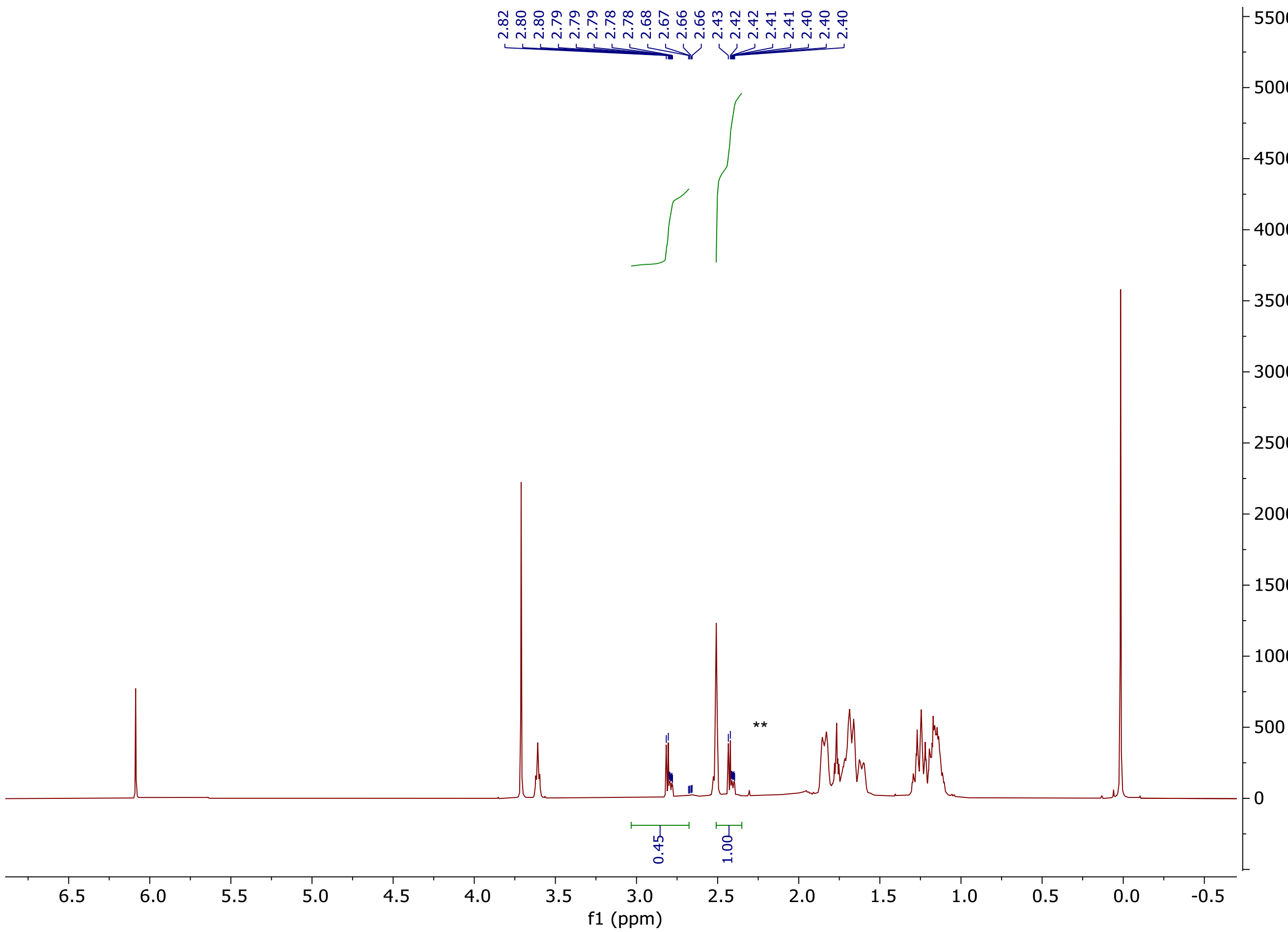

Figure S240: Hydrophosphination of 0.5 mmol of acrylonitrile with 2 equivalents $CyPH_2$ in DMSO-d_6 using 5 mol% **1** - 0 h – ^{1}H NMR

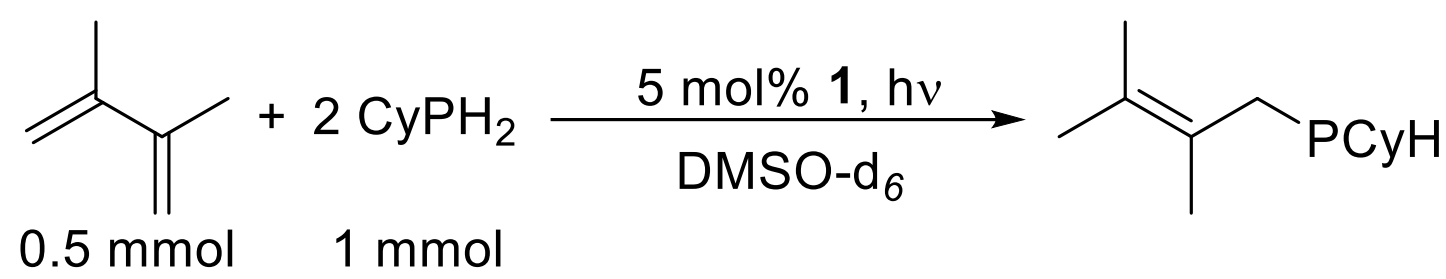

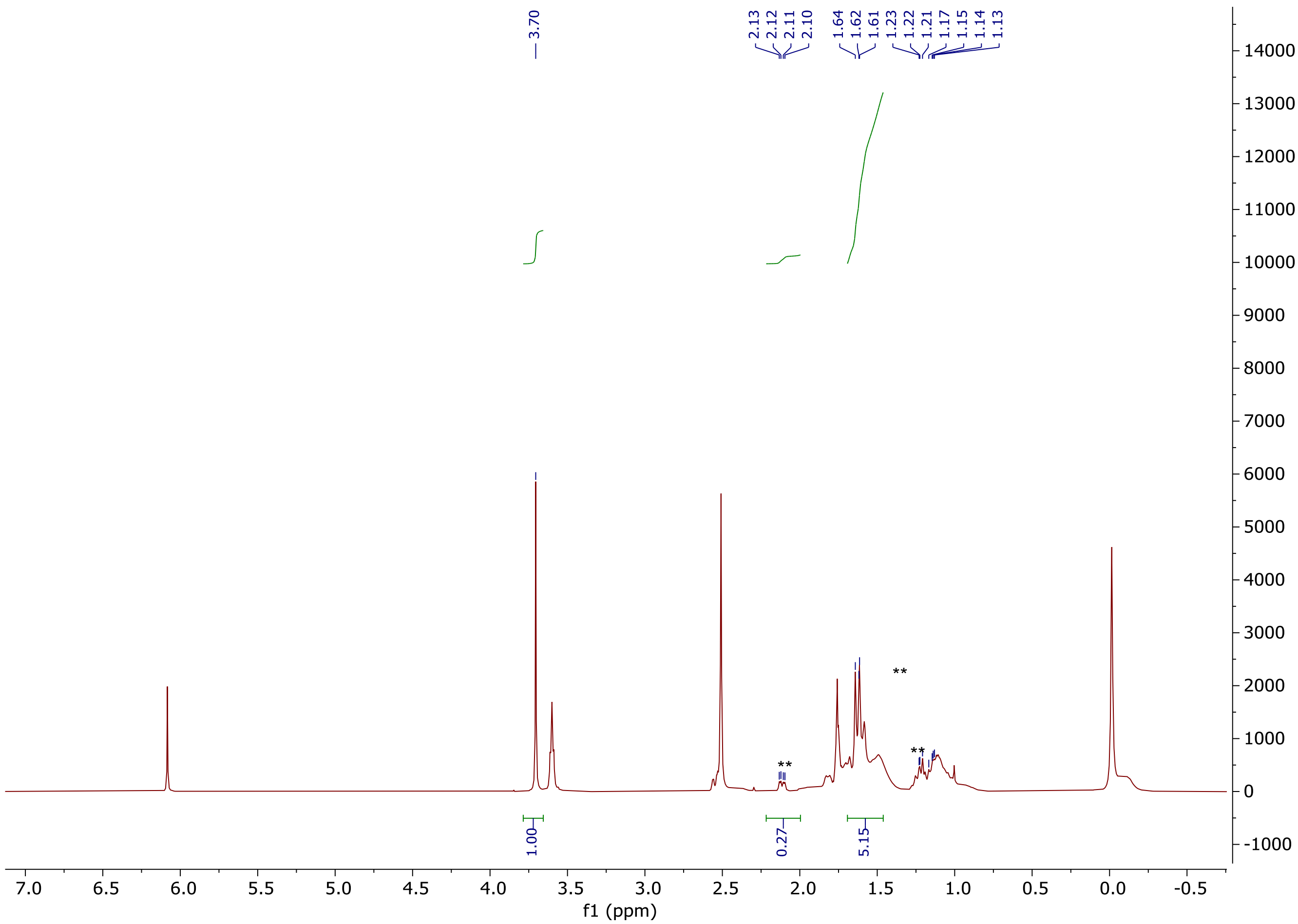

Figure S241: Hydrophosphination of 0.5 mmol of 2,3-dimethyl 1,3-butadiene with 2 equivalents $CyPH_2$ in DMSO-d_6 using 5 mol% **1** - 0 h – ^{1}H NMR

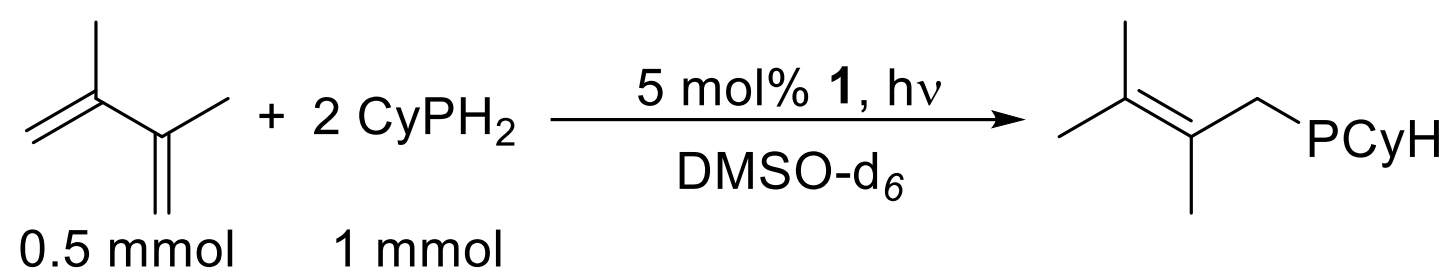

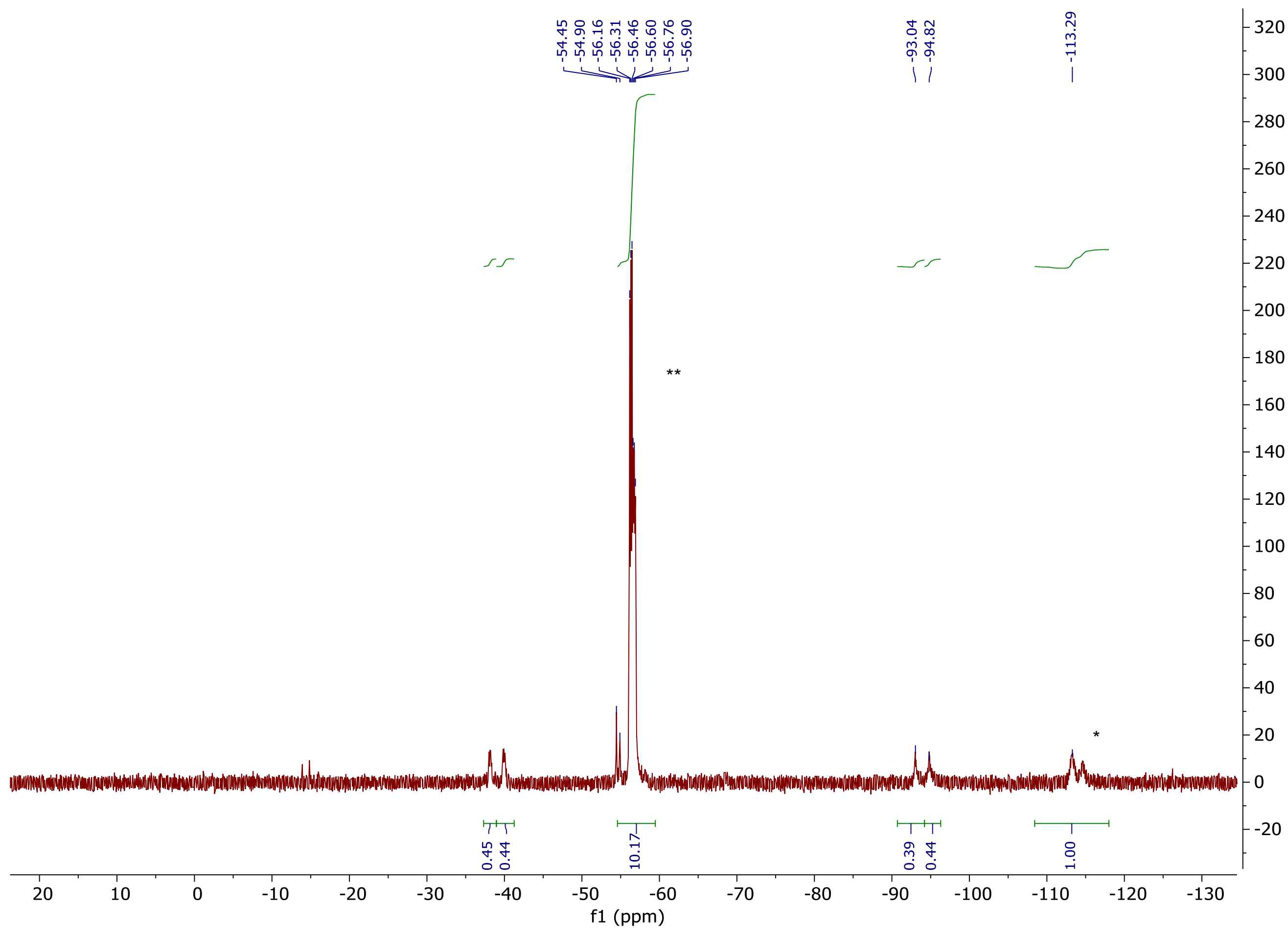

Figure S242: Hydrophosphination of 0.5 mmol of 2,3-dimethyl 1,3-butadiene with 2 equivalents $CyPH_2$ in DMSO-d_6 using 5 mol% **1** - 0 h – $^{31}P\{^1H\}$ NMR

Hydrophosphination of unsaturated compounds with dicyclohexyl phosphine in DMSO-d6 using 5 mol% **1**

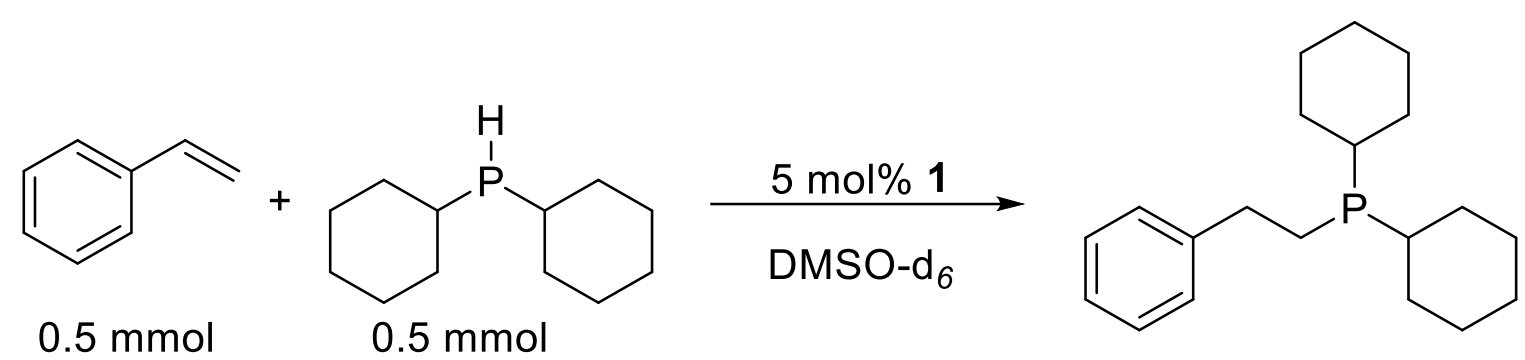

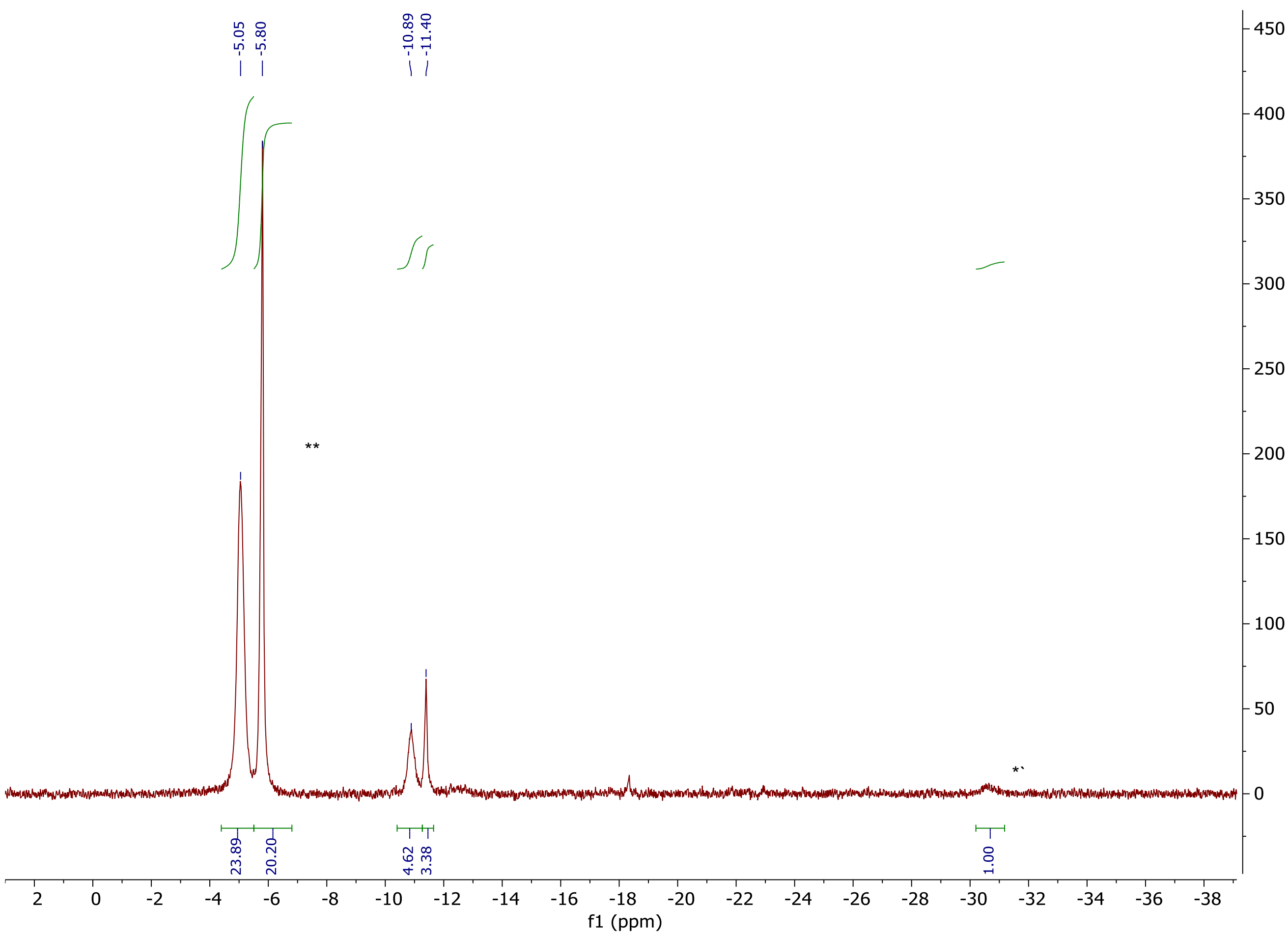

Figure S243: Hydrophosphination of 0.5 mmol of styrene with equimolar Cy_2PH in DMSO-d6 using 5 mol% **1** - 0 h – $^{31}P\{^1H\}$ NMR

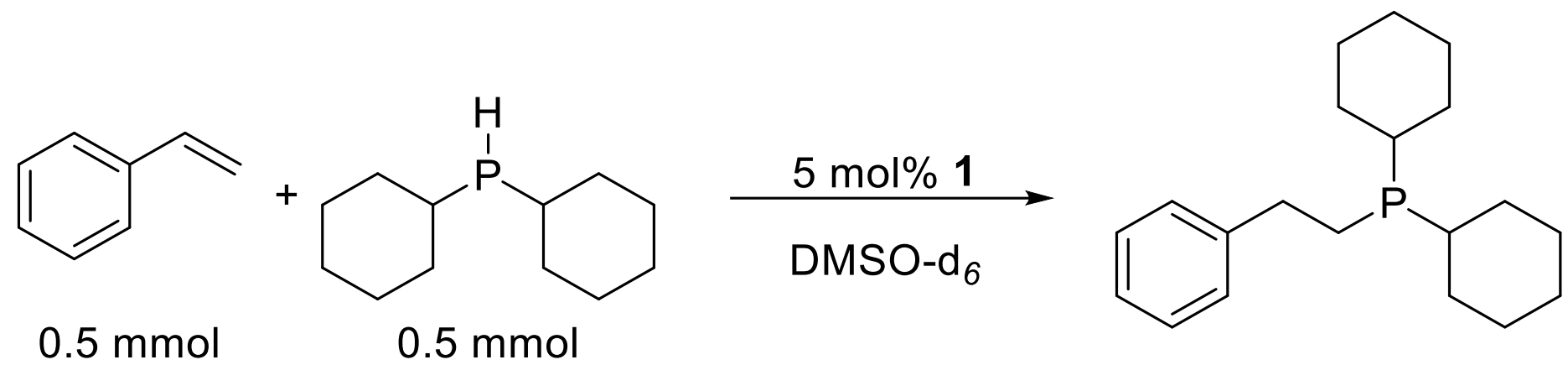

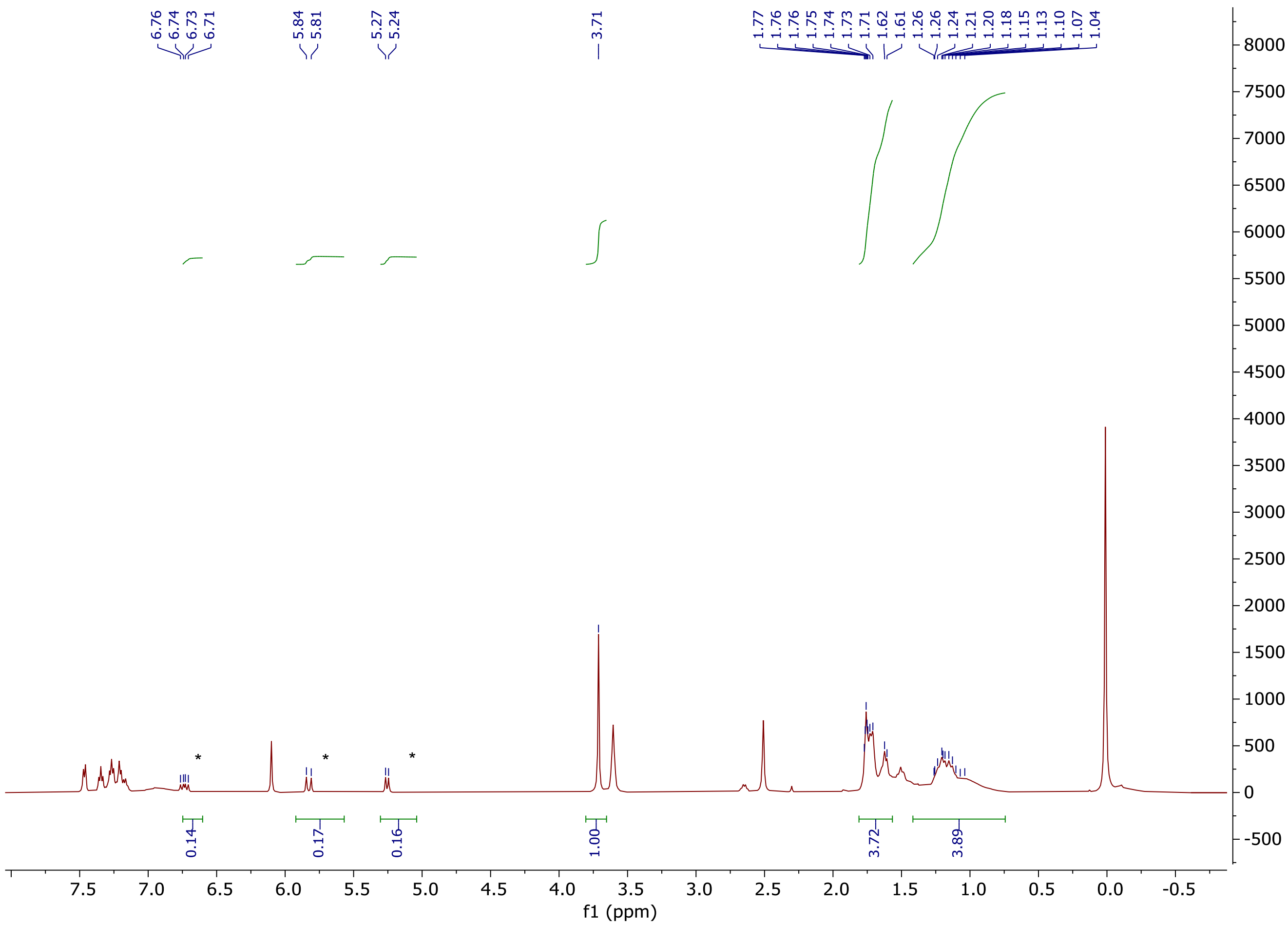

Figure S244: Hydrophosphination of 0.5 mmol of styrene with equimolar Cy_2PH in DMSO-d_6 using 5 mol% **1** - 0 h – ^1H NMR

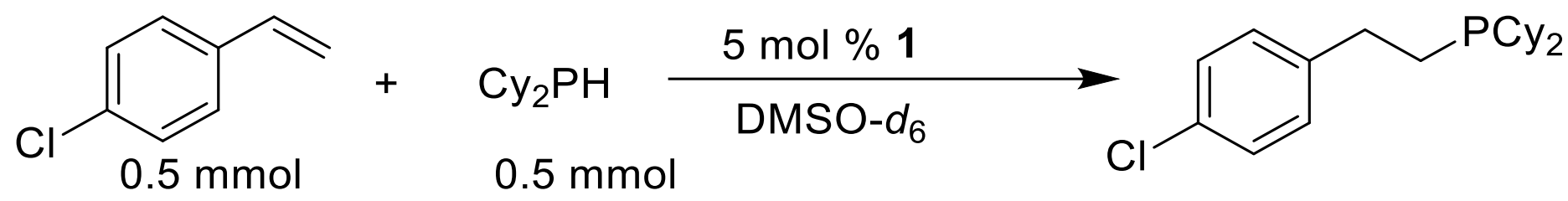

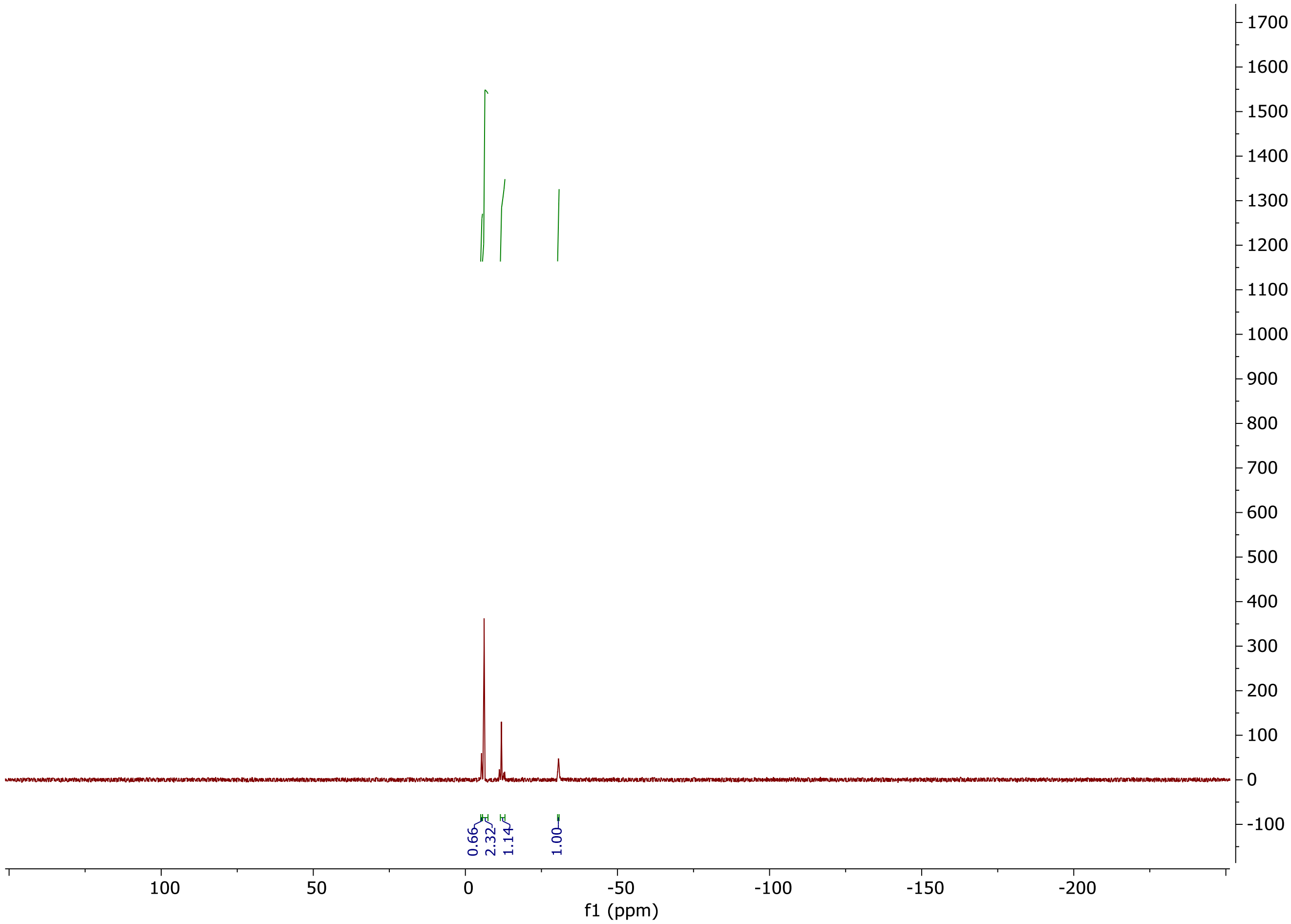

Figure S245: Hydrophosphination of 0.5 mmol of 4-chloro styrene with equimolar Cy_2PH in DMSO-d_6 using 5 mol% **1** - 0 h – $^{31}P\{^1H\}$ NMR

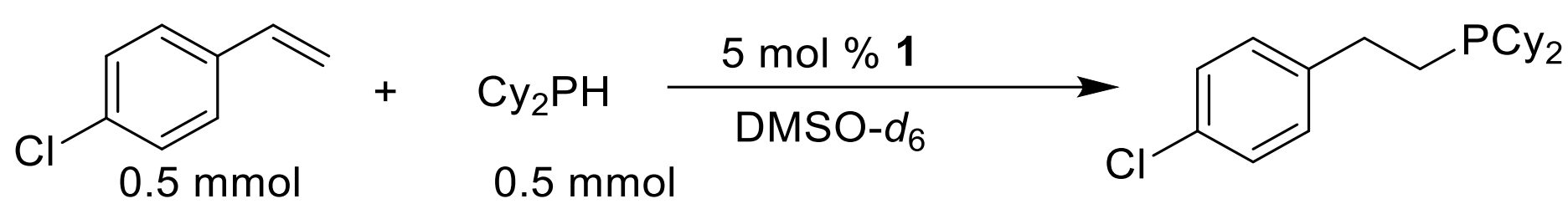

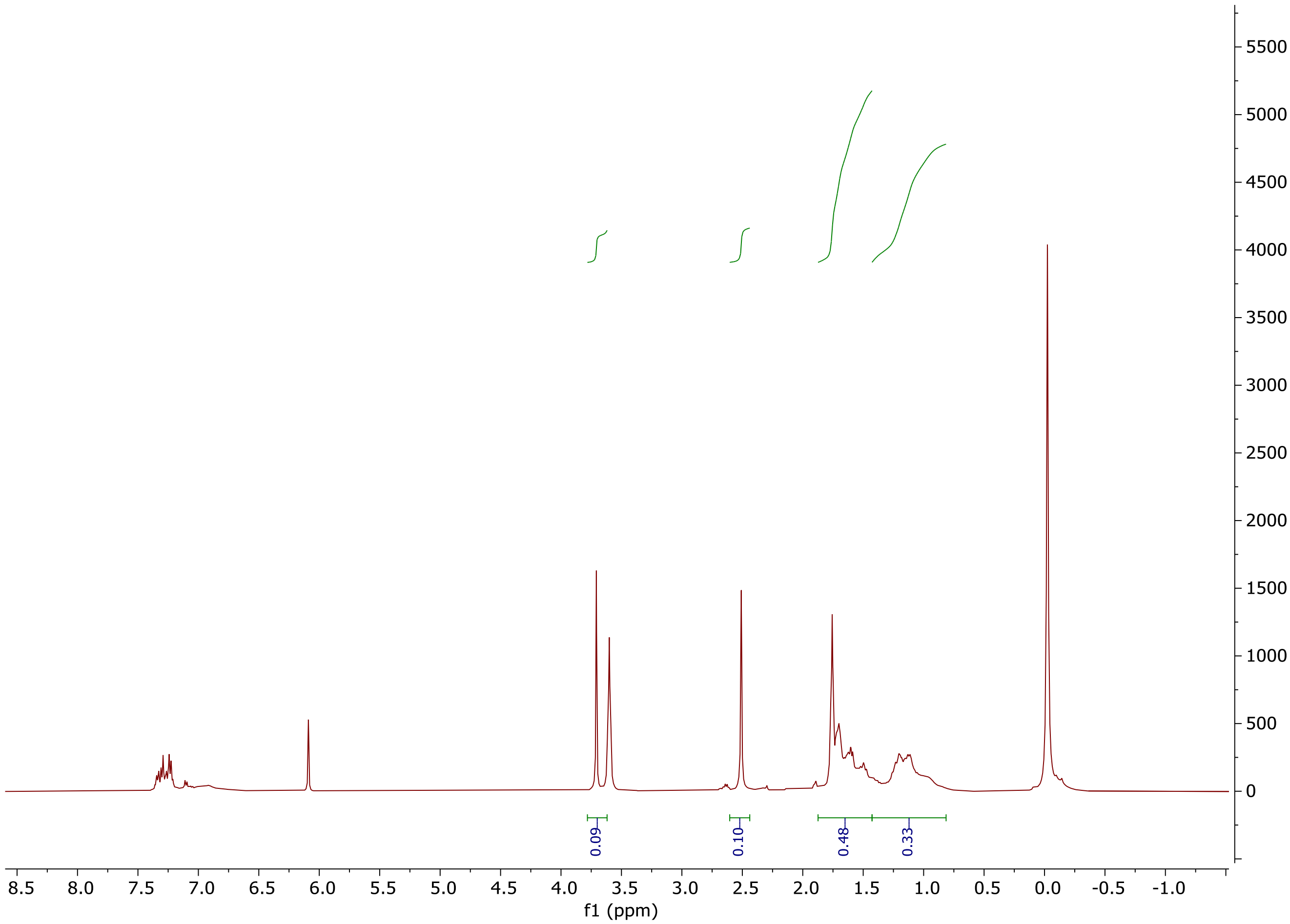

Figure S246: Hydrophosphination of 0.5 mmol of 4-chloro styrene with equimolar Cy_2PH in DMSO-d_6 using 5 mol% **1** - 0 h – ^{1}H NMR

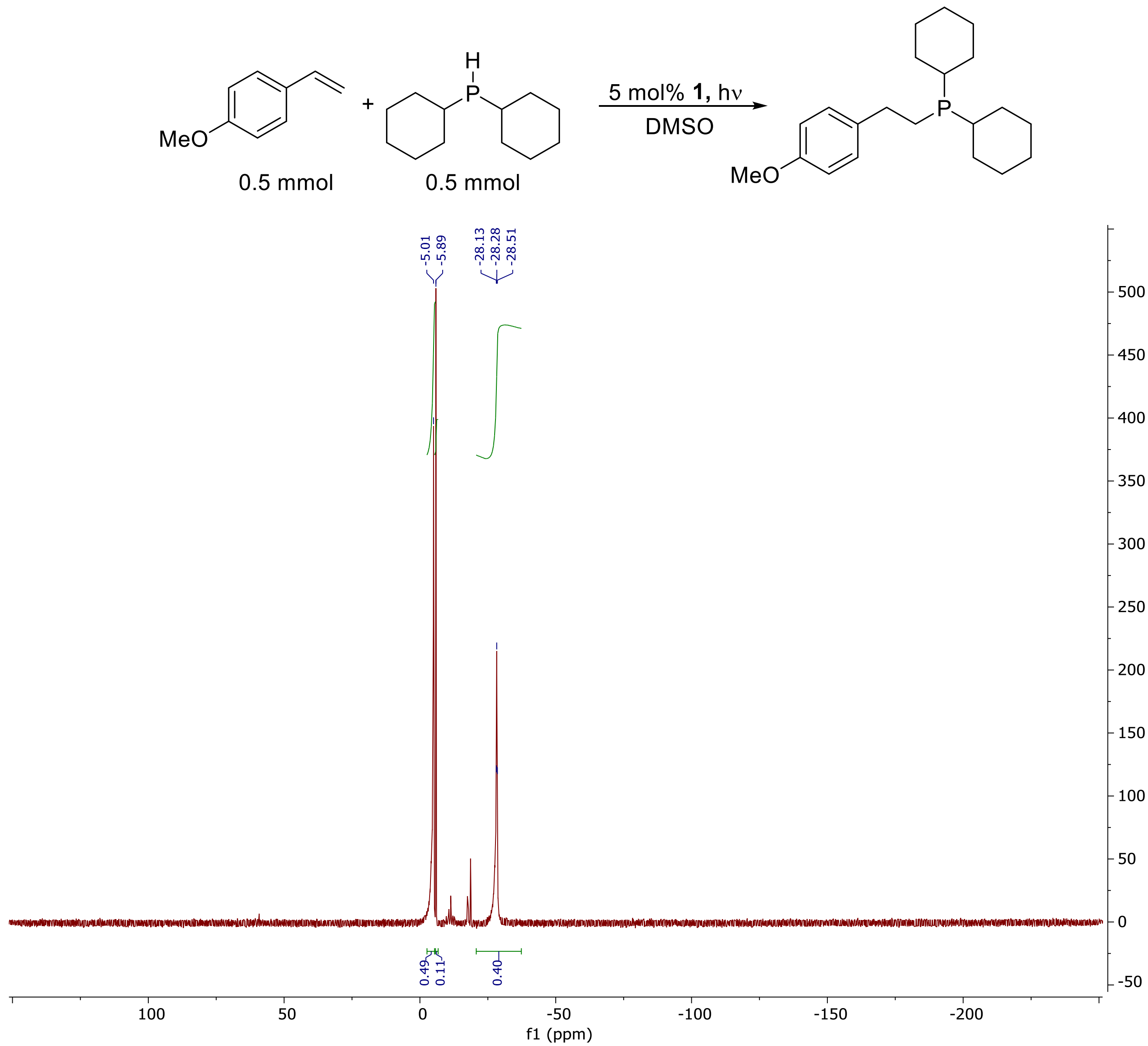

Figure S247: Hydrophosphination of 0.5 mmol of 4-methoxy styrene with equimolar Cy_2PH in DMSO using 5 mol% **1** - 0 h – $^{31}P\{^1H\}$ NMR

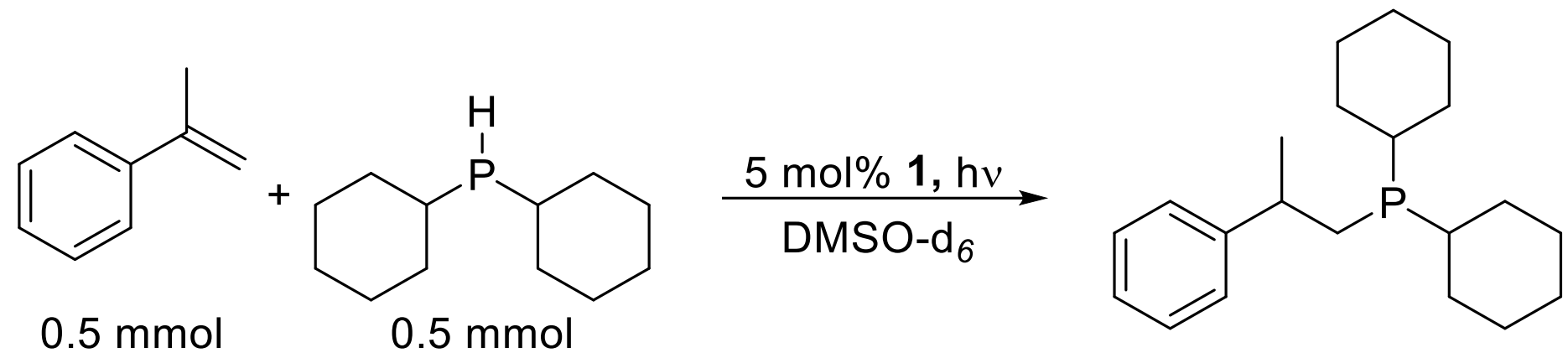

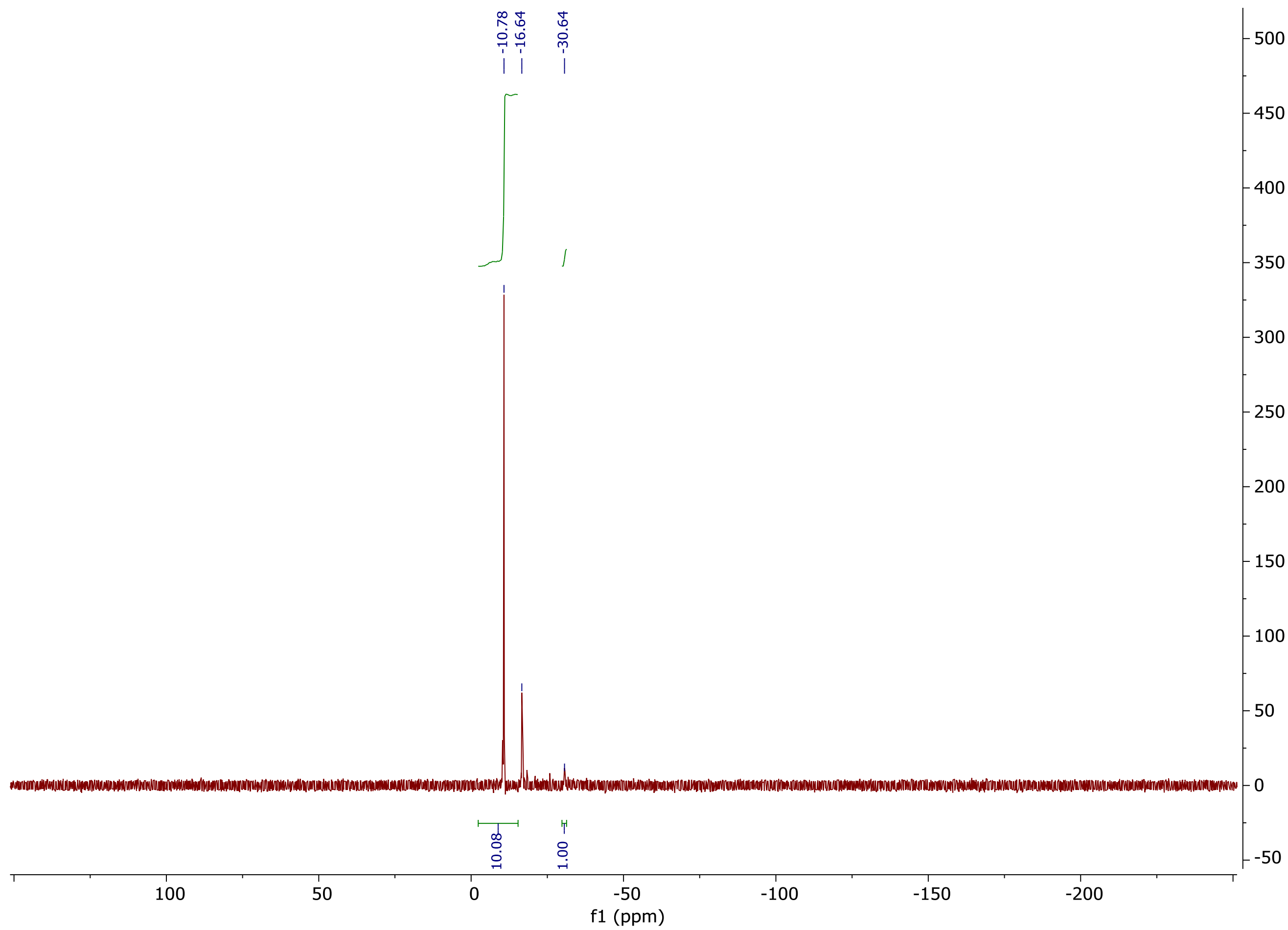

Figure S248: Hydrophosphination of 0.5 mmol of α-methyl styrene with equimolar Cy_2PH in DMSO-d_6 using 5 mol% **1** - 0 h – $^{31}P\{^{1}H\}$ NMR

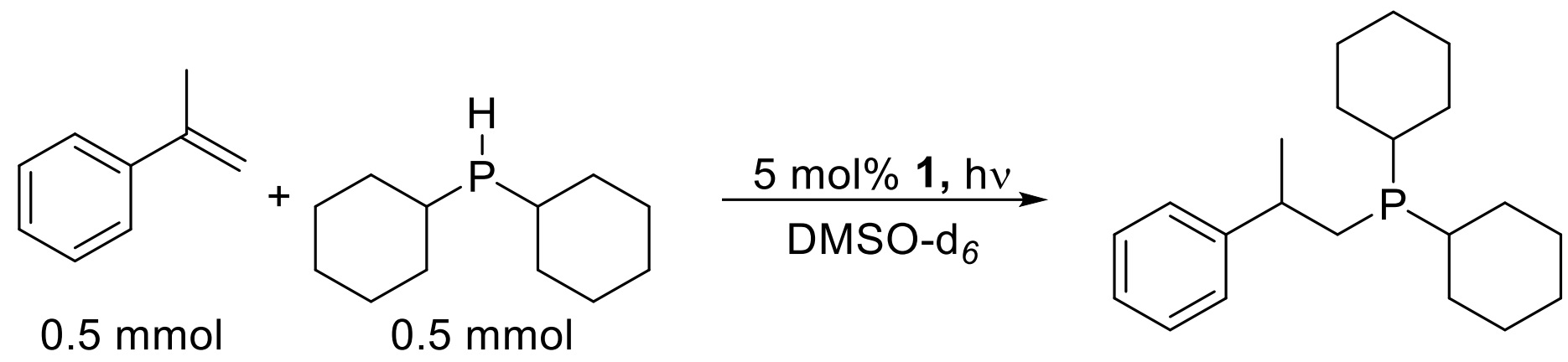

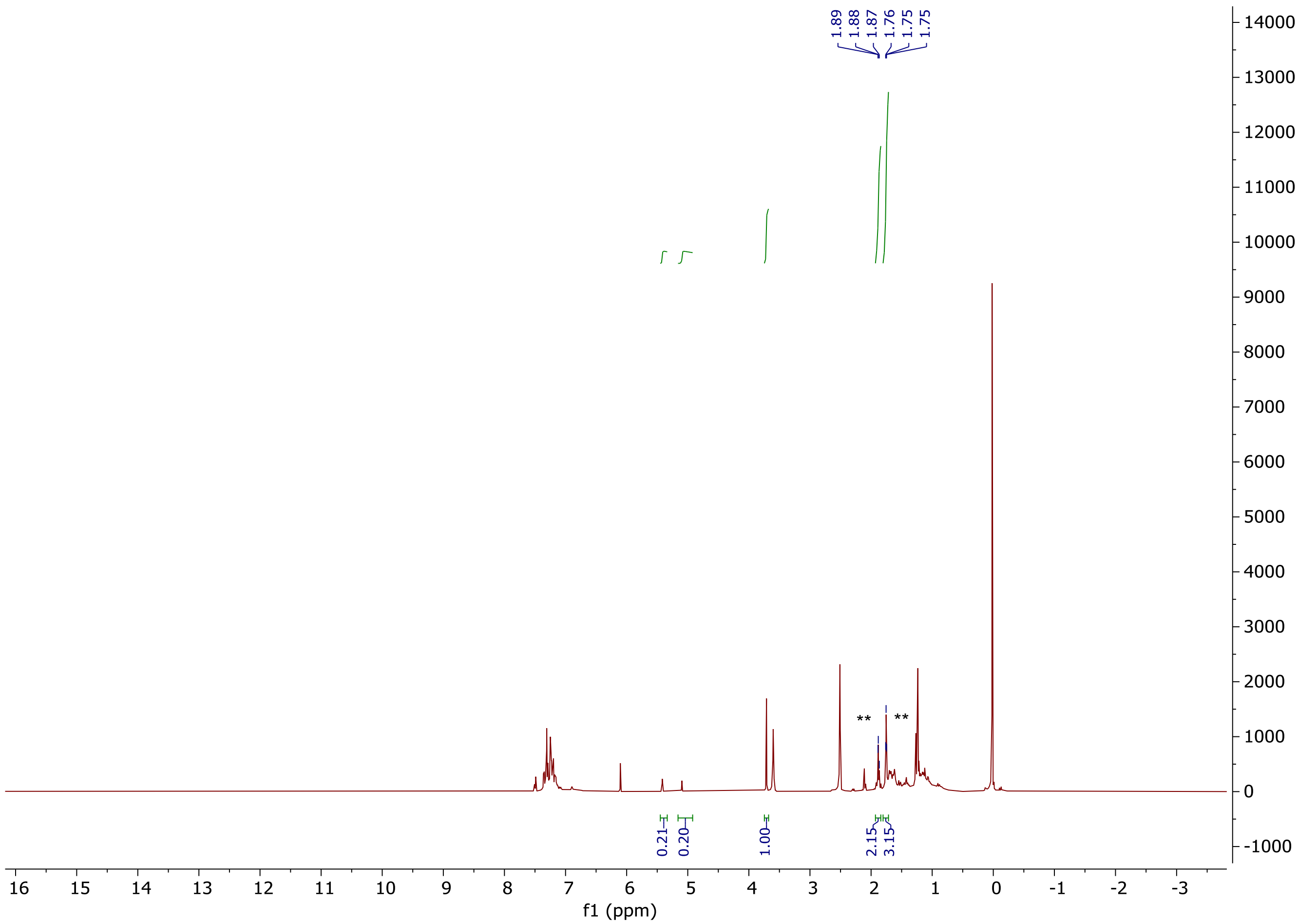

Figure S249: Hydrophosphination of 0.5 mmol of α – methyl styrene with equimolar Cy_2PH in DMSO-d_6 using 5 mol% **1** - 0 h – ^1H NMR

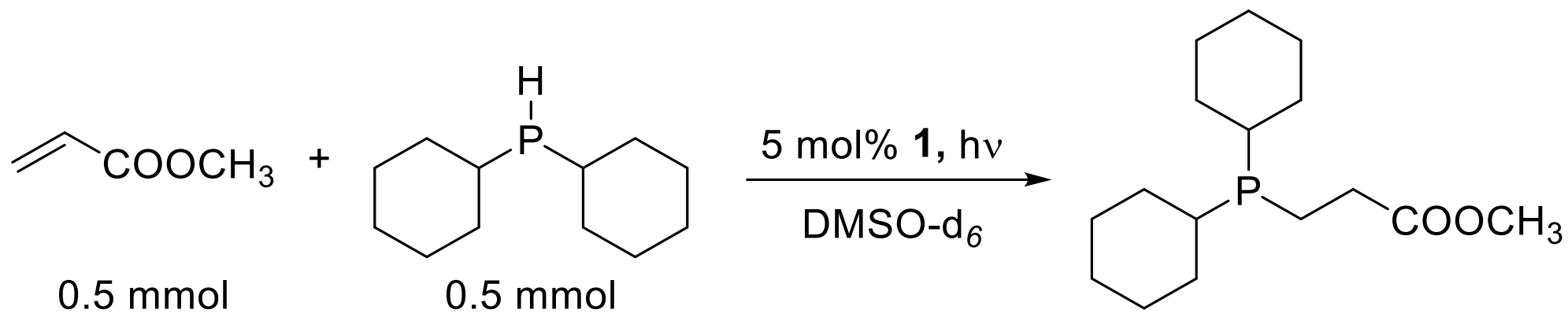

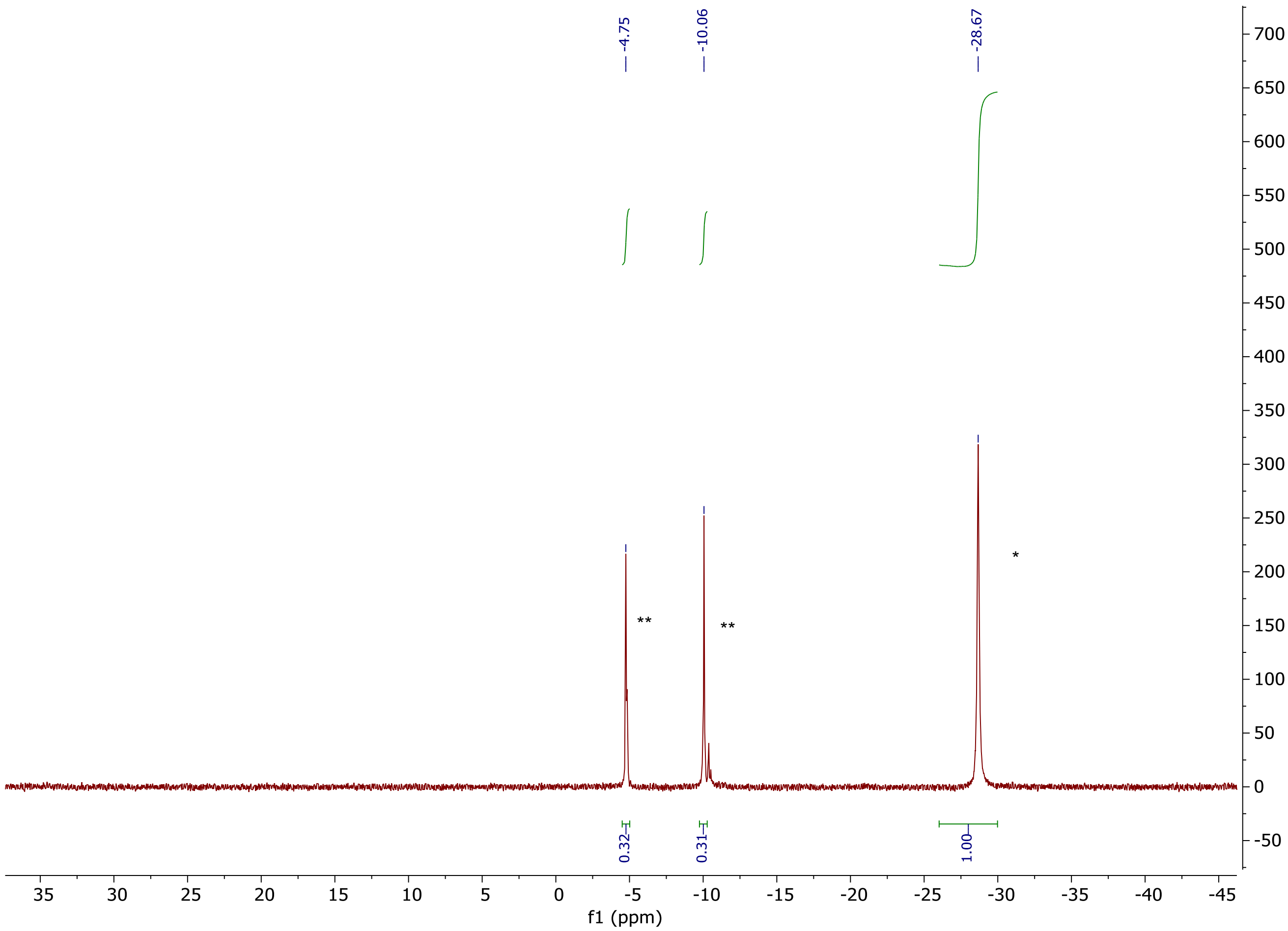

Figure S250: Hydrophosphination of 0.5 mmol of methyl acrylate with equimolar Cy_2PH in DMSO-d_6 using 5 mol% **1** under UV 360 nm irradiation - 31 h – ^1H NMR

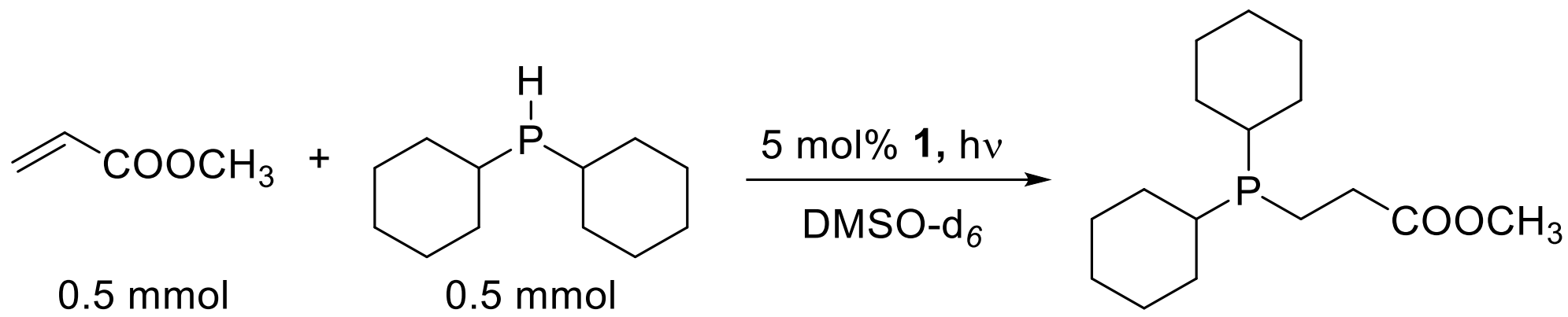

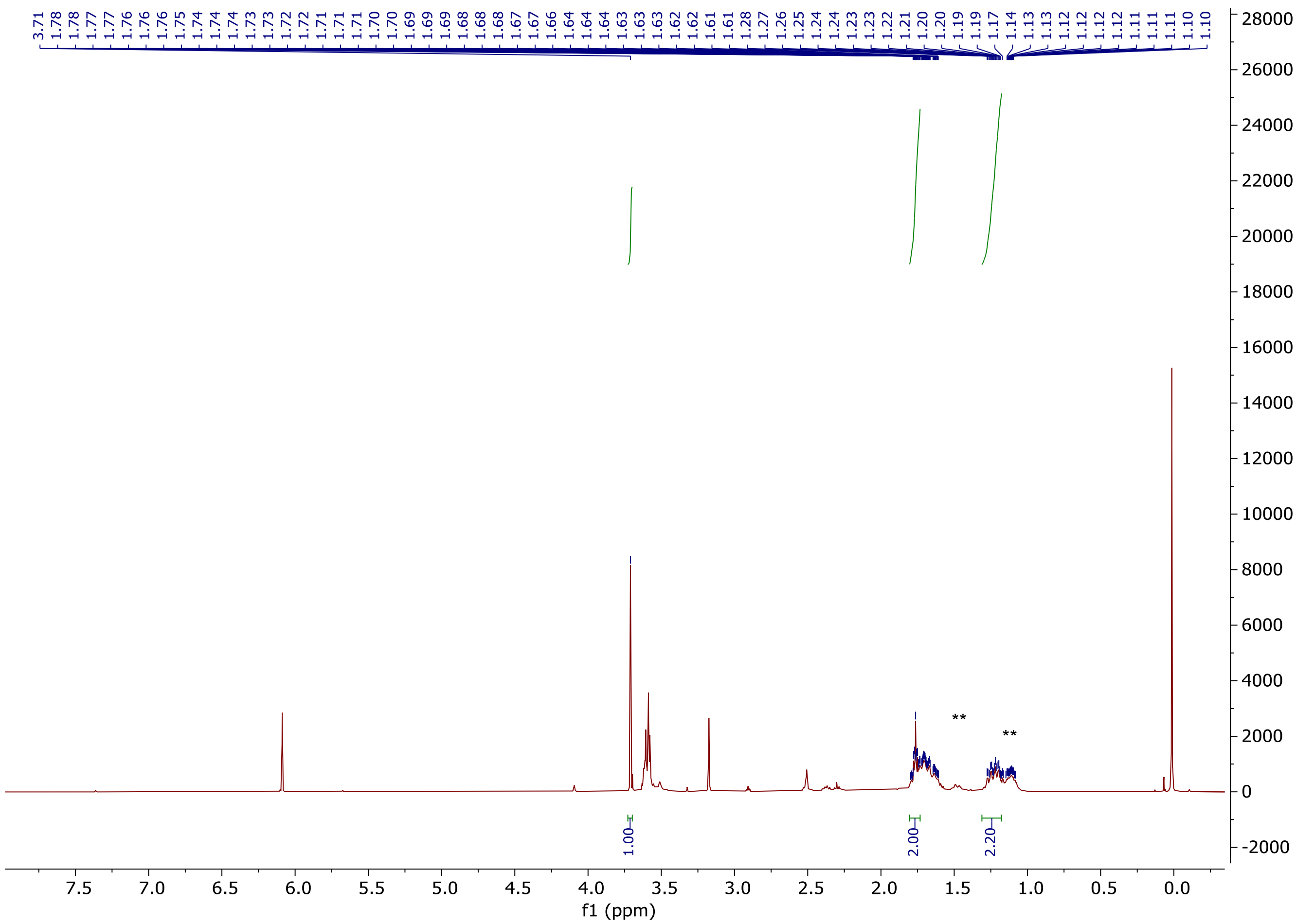

Figure S251: Hydrophosphination of 0.5 mmol of methyl acrylate with equimolar Cy_2PH in $DMSO-d_6$ using 5 mol% **1** under UV 360 nm irradiation - 31 h – ^1H NMR

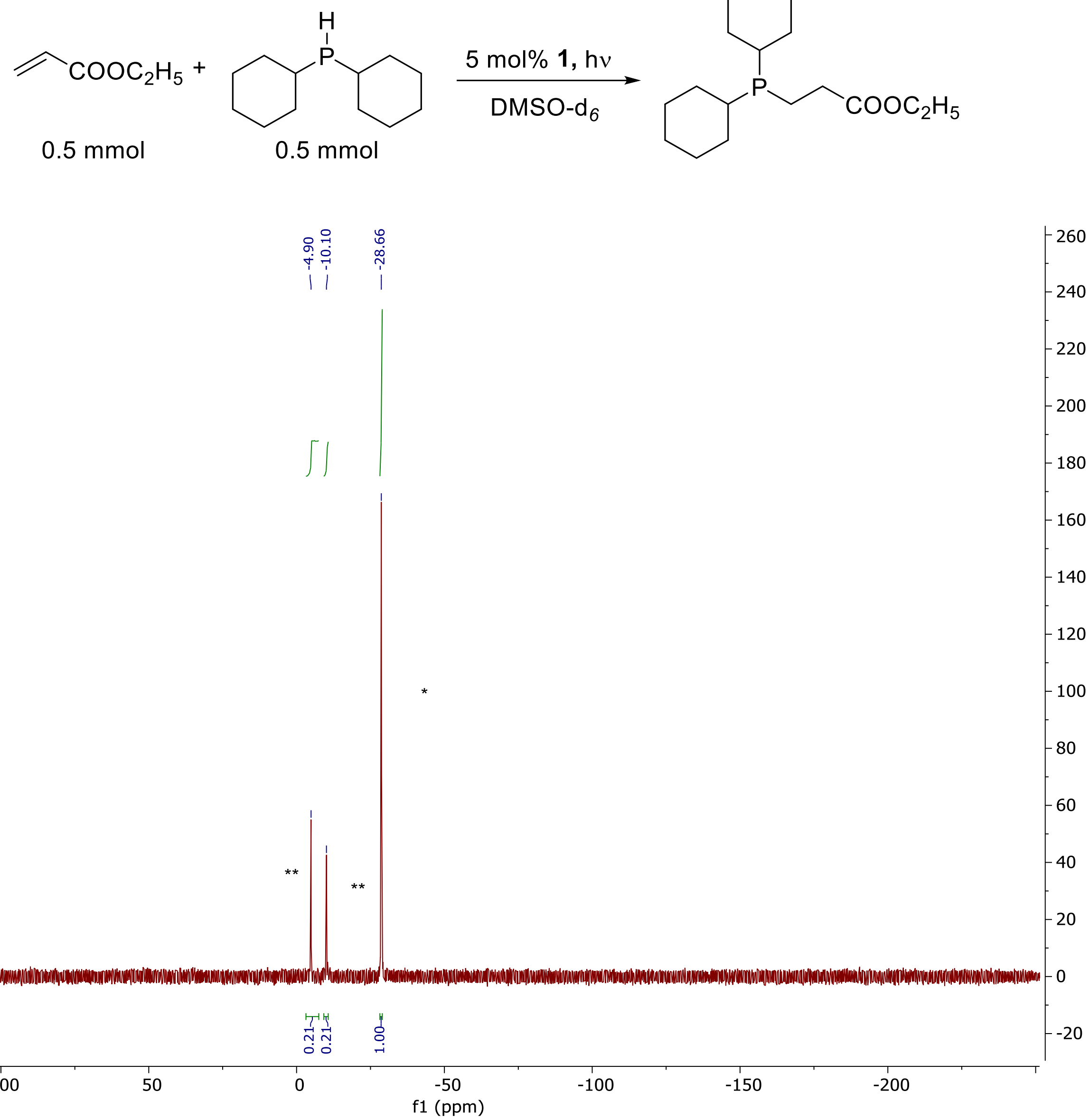

Figure S252: Hydrophosphination of 0.5 mmol of ethyl acrylate with equimolar Cy_2PH in DMSO-d_6 using 5 mol% **1** under UV 360 nm irradiation - 13 h – $^{31}P\{^{1}H\}$ NMR

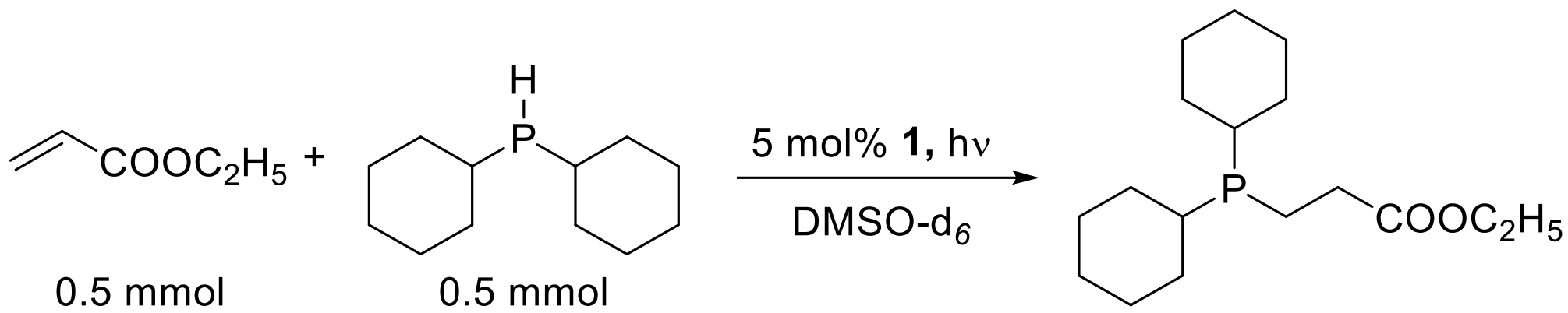

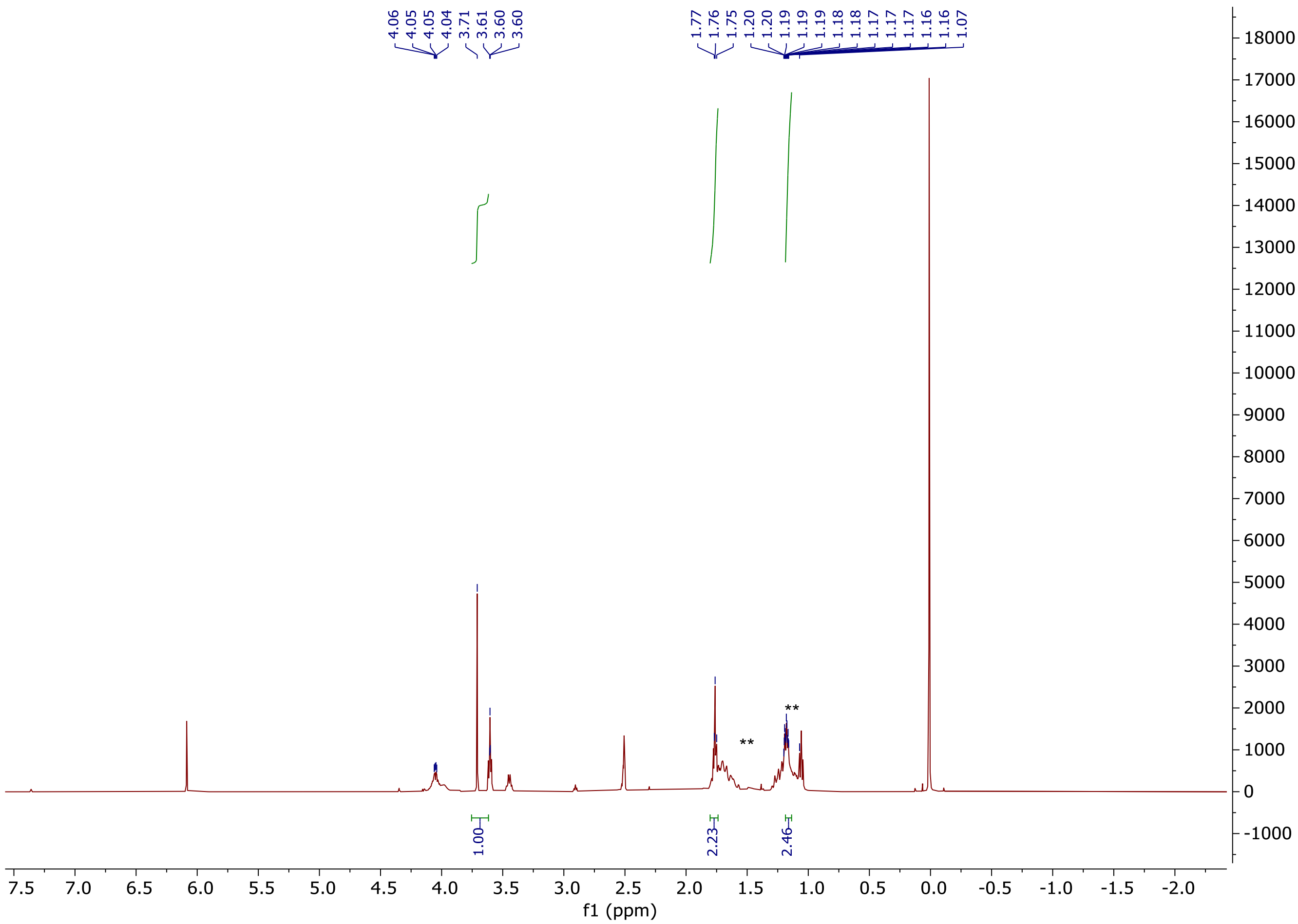

Figure S253: Hydrophosphination of 0.5 mmol of ethyl acrylate with equimolar Cy_2PH in DMSO-d_6 using 5 mol% **1** under UV 360 nm irradiation - 13 h – ^1H NMR

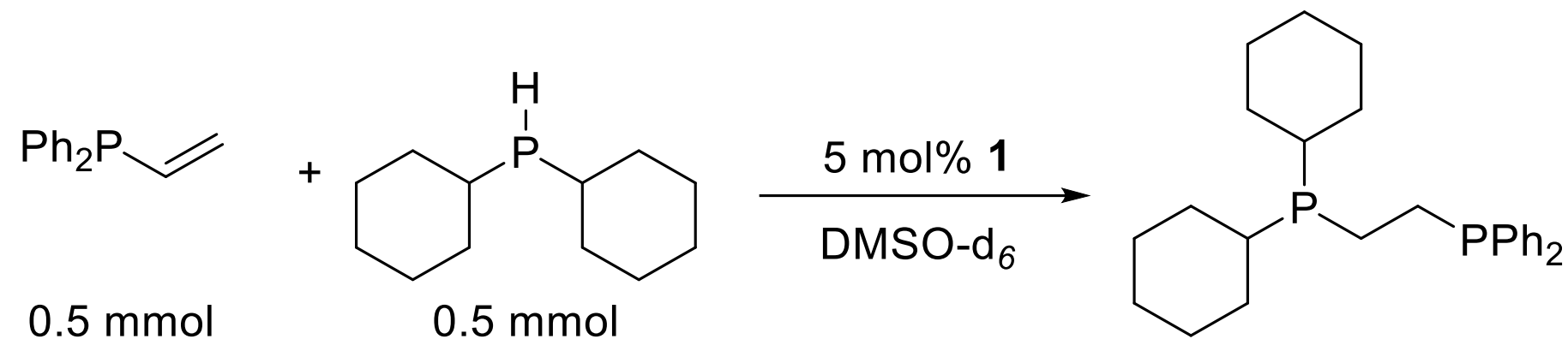

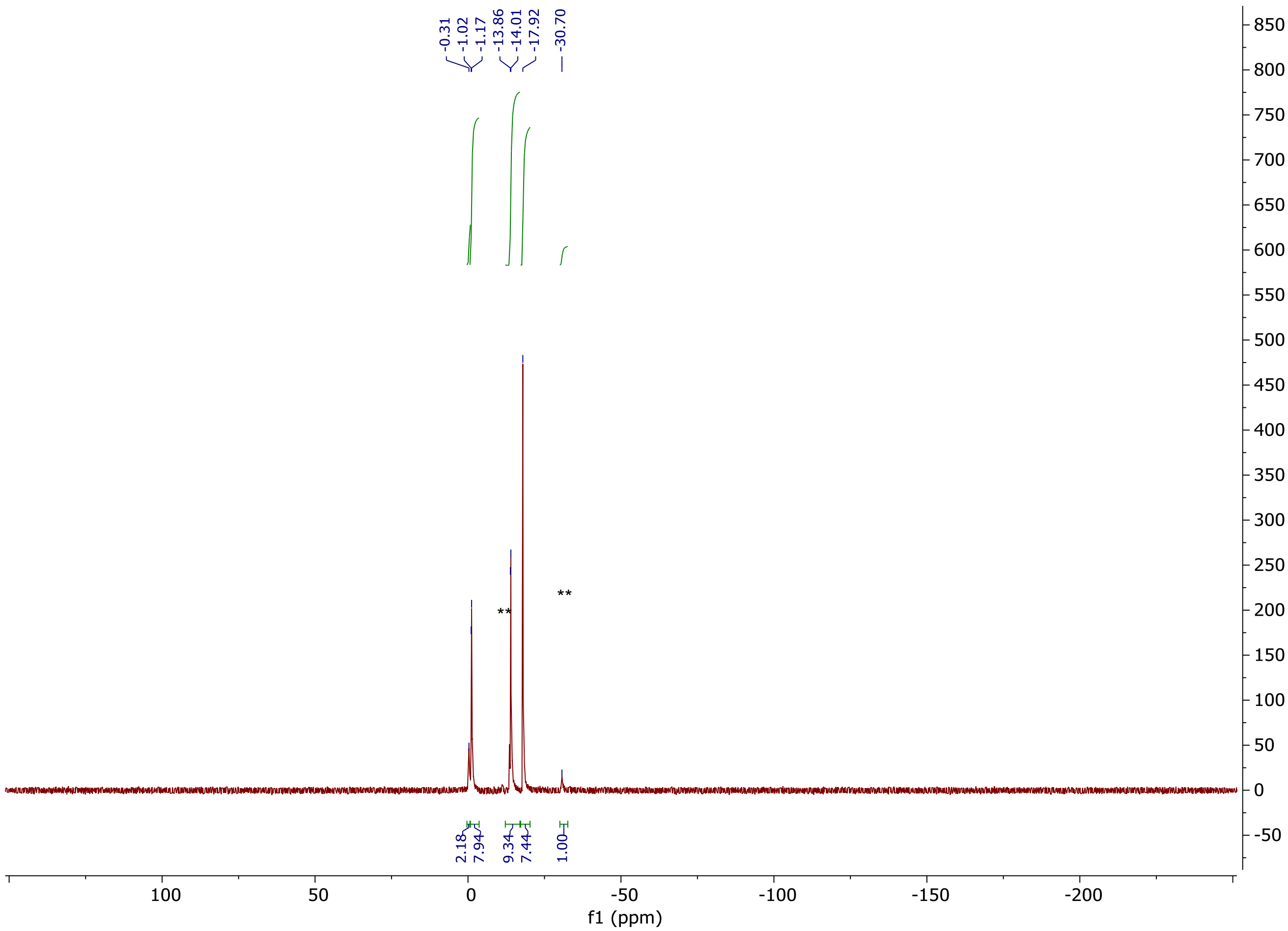

Figure S254: Hydrophosphination of 0.5 mmol of vinyl diphenylphosphine with equimolar Cy_2PH in DMSO-d_6 using 5 mol% **1** - 0 h – $^{31}P\{^1H\}$ NMR

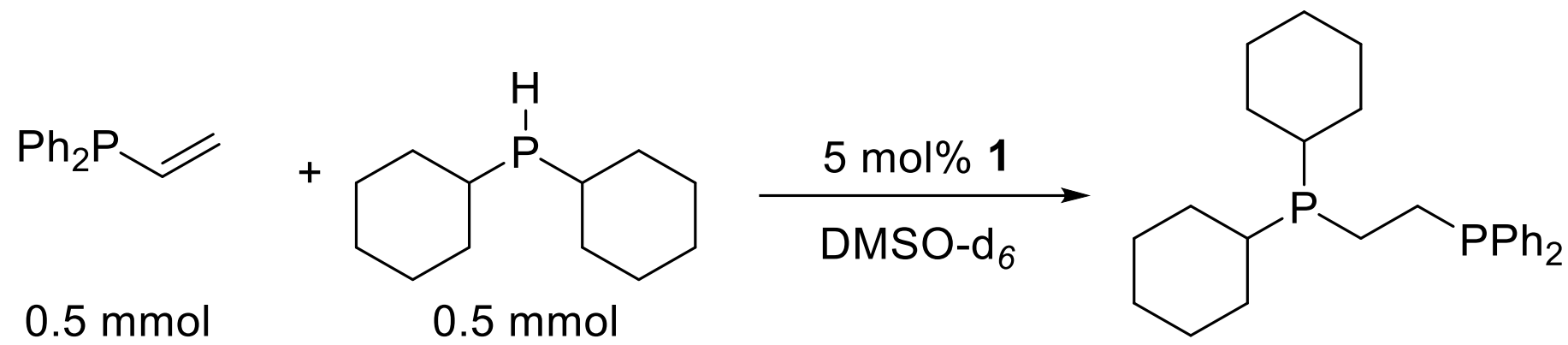

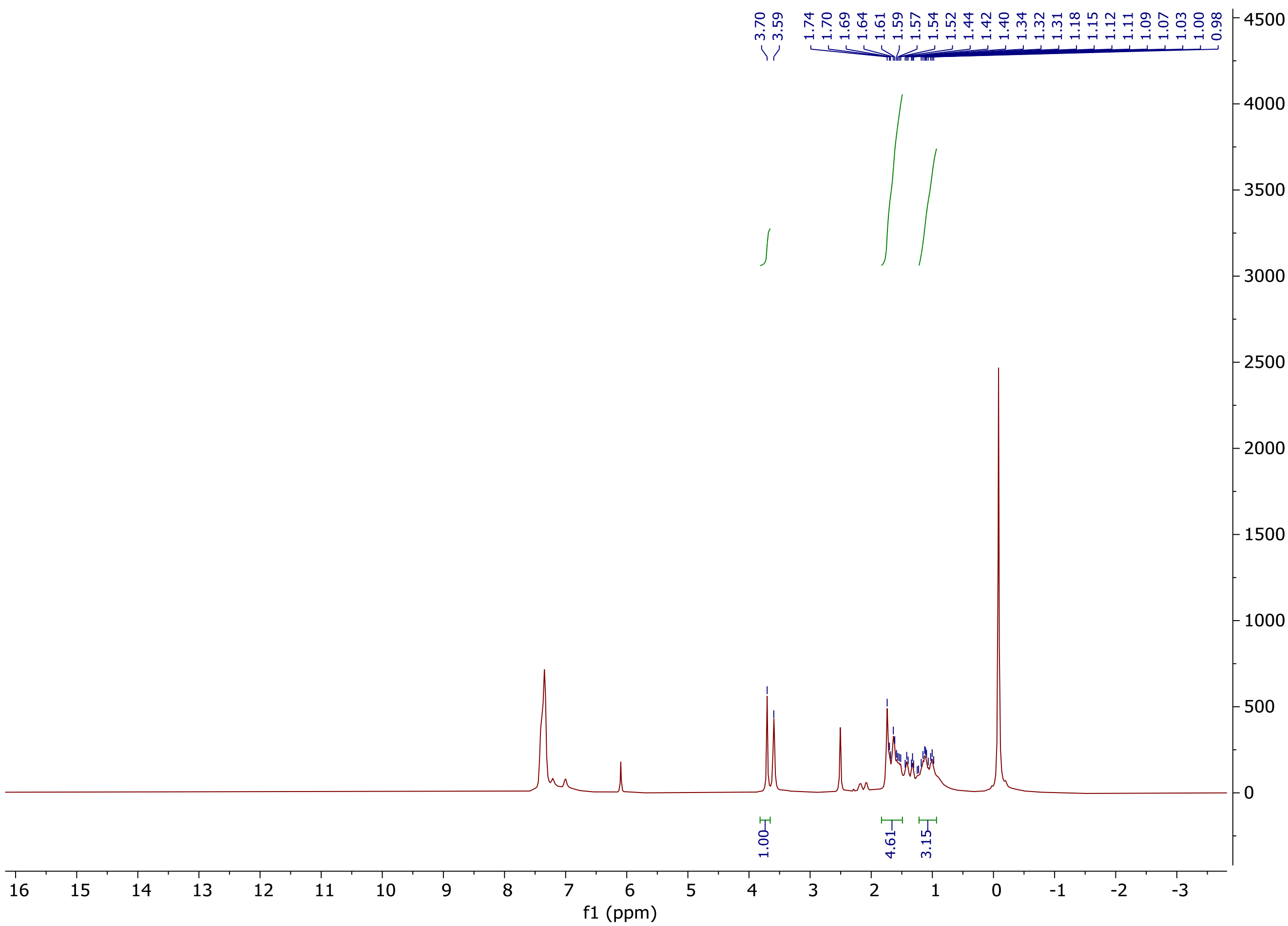

Figure S255: Hydrophosphination of 0.5 mmol of vinyl diphenylphosphine with equimolar Cy_2PH in DMSO-d_6 using 5 mol% **1** - 0 h – ^{1}H NMR

Hydrophosphination of unsaturated compounds with mesityl phosphine in DMSO-d_6 using 5 mol% **1**

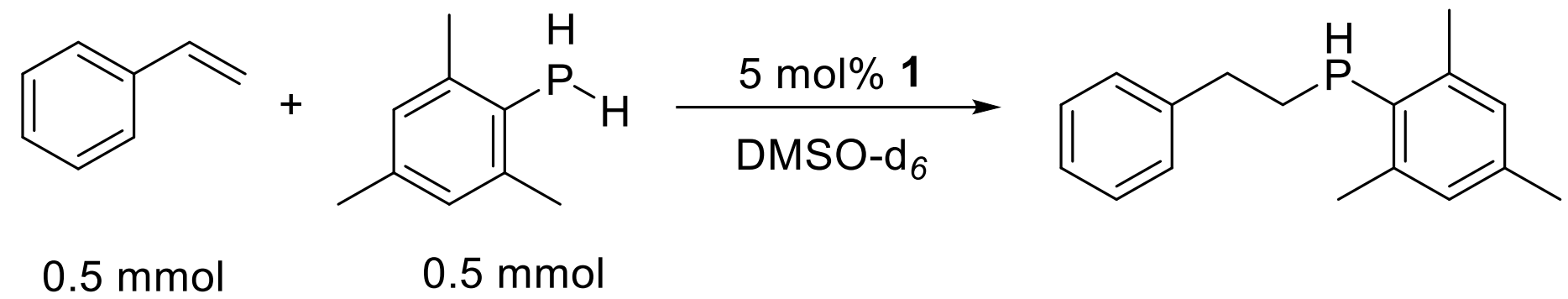

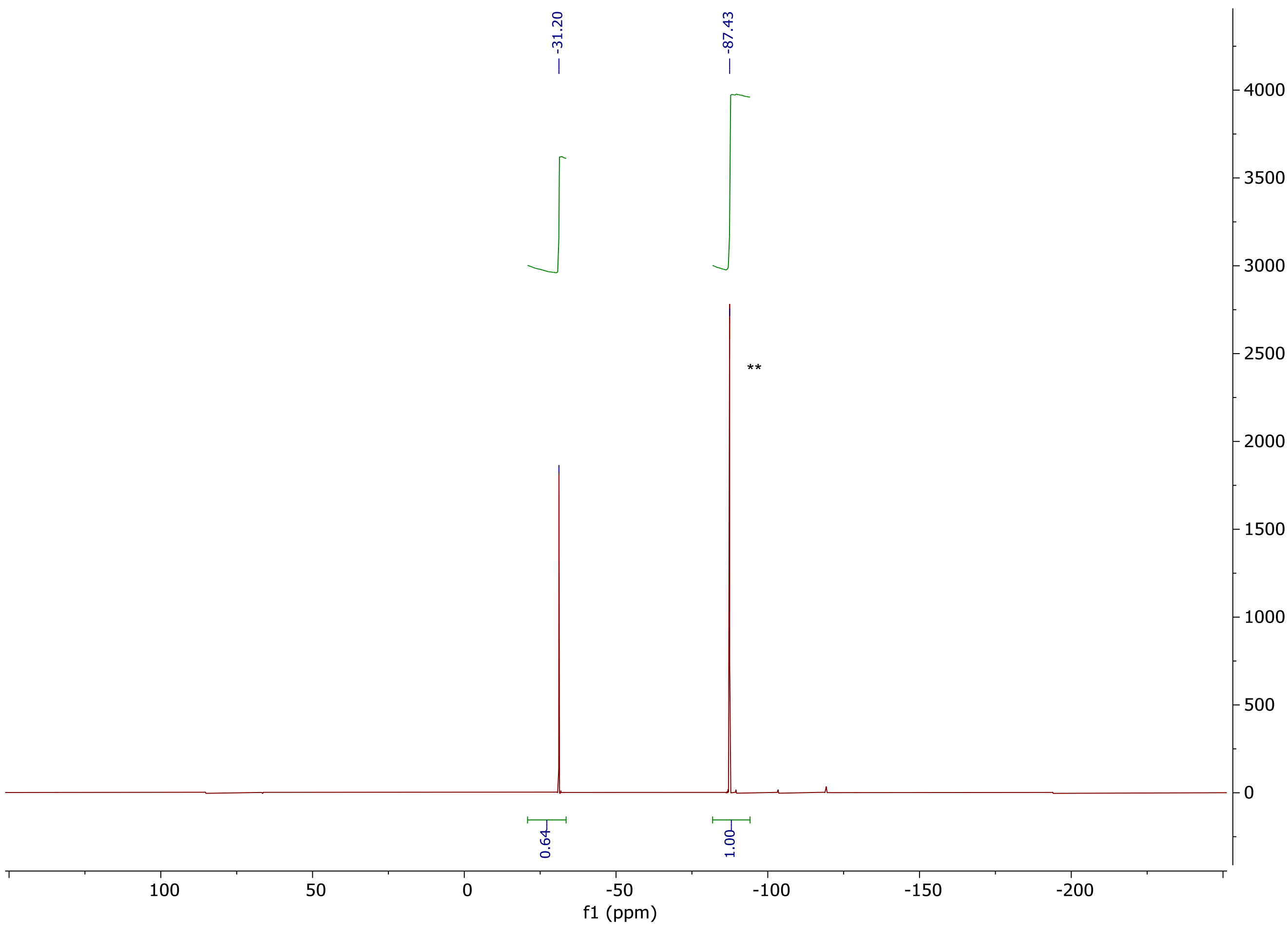

Figure S256: Hydrophosphination of 0.5 mmol of styrene with equimolar mesitylphosphine in DMSO-d_6 using 5 mol% **1** - 0 h – $^{31}P\{^1H\}$ NMR

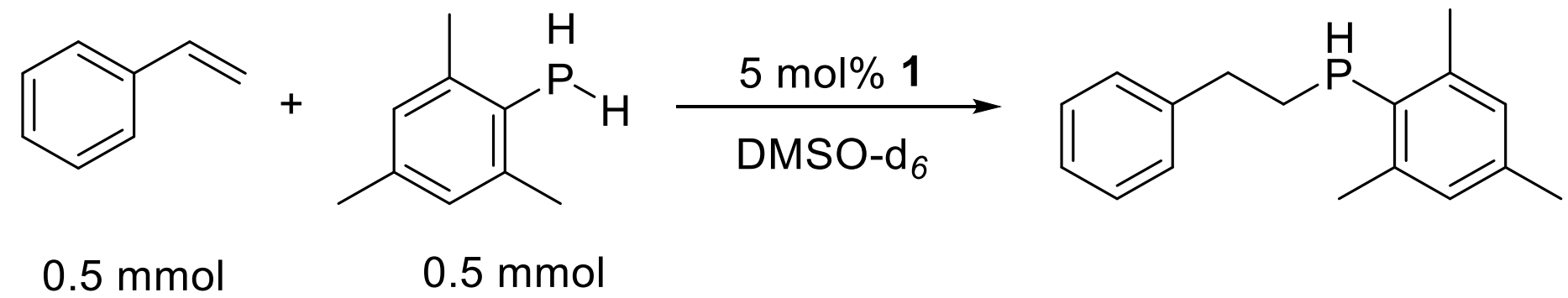

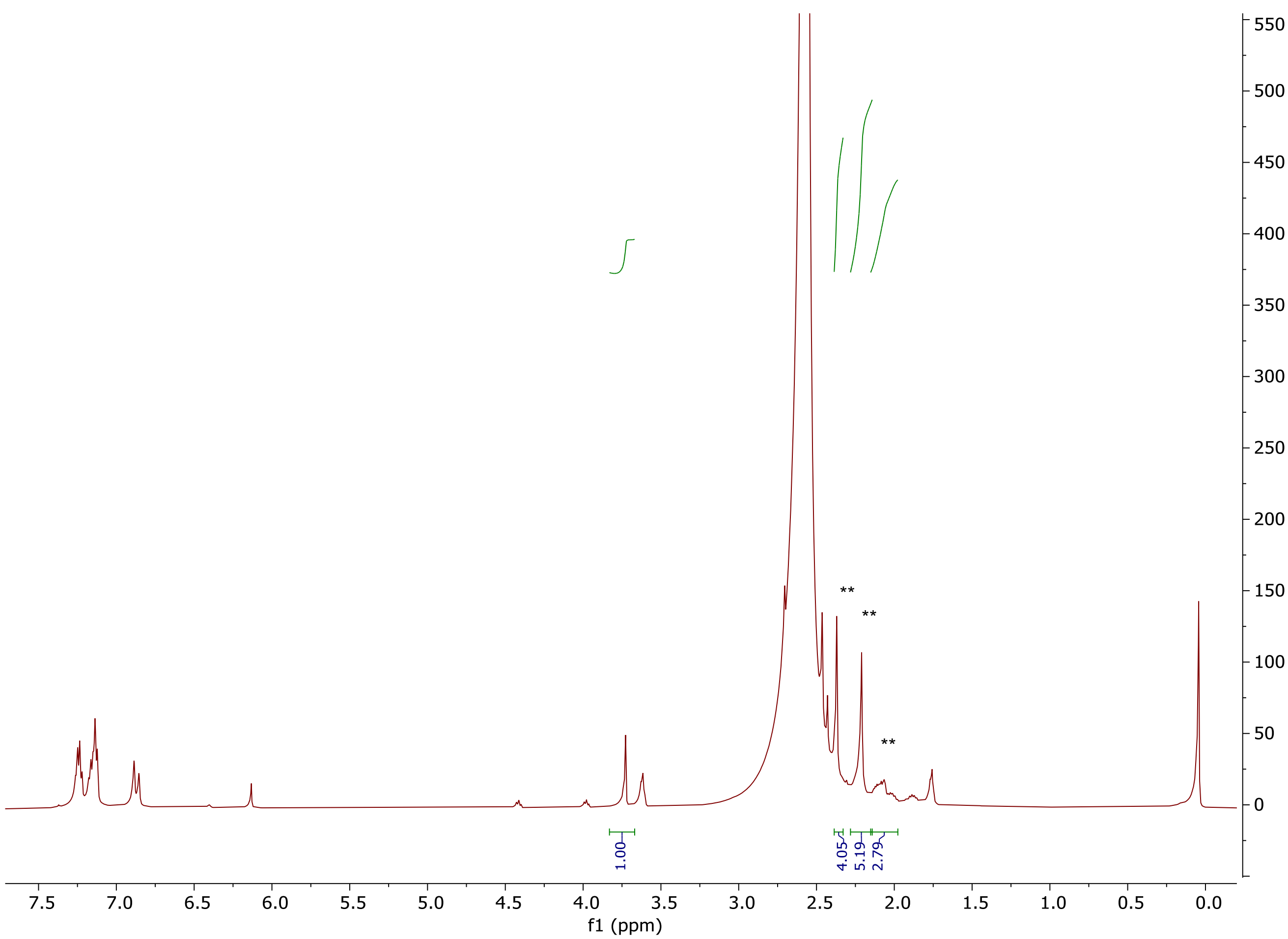

Figure S257: Hydrophosphination of 0.5 mmol of styrene with mesitylphosphine in DMSO-d6 using 5 mol% **1** - 0 h – ^{1}H NMR

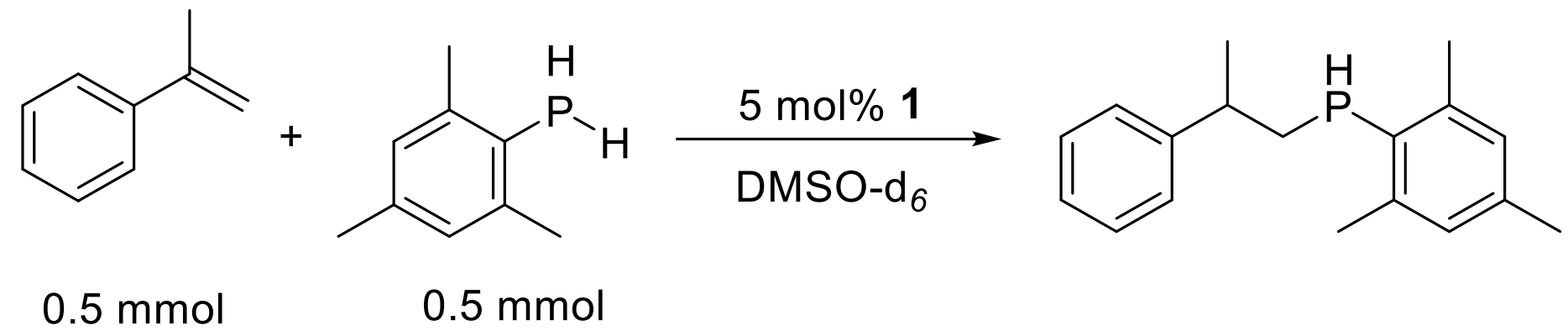

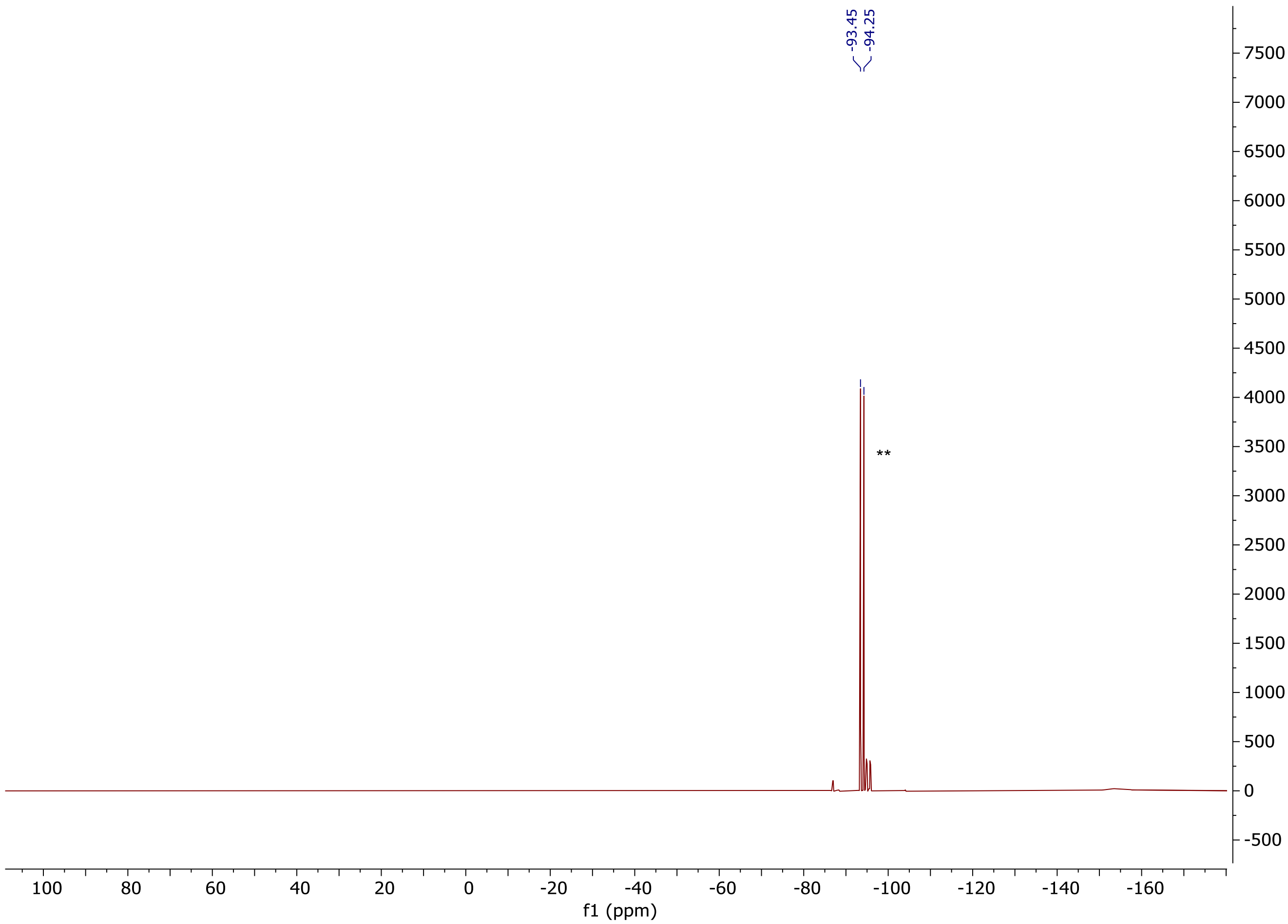

Figure S258: Hydrophosphination of 0.5 mmol of α-methyl styrene with mesitylphosphine in DMSO-d6 using 5 mol% **1** - 0 h – $^{31}P\{^{1}H\}$ NMR

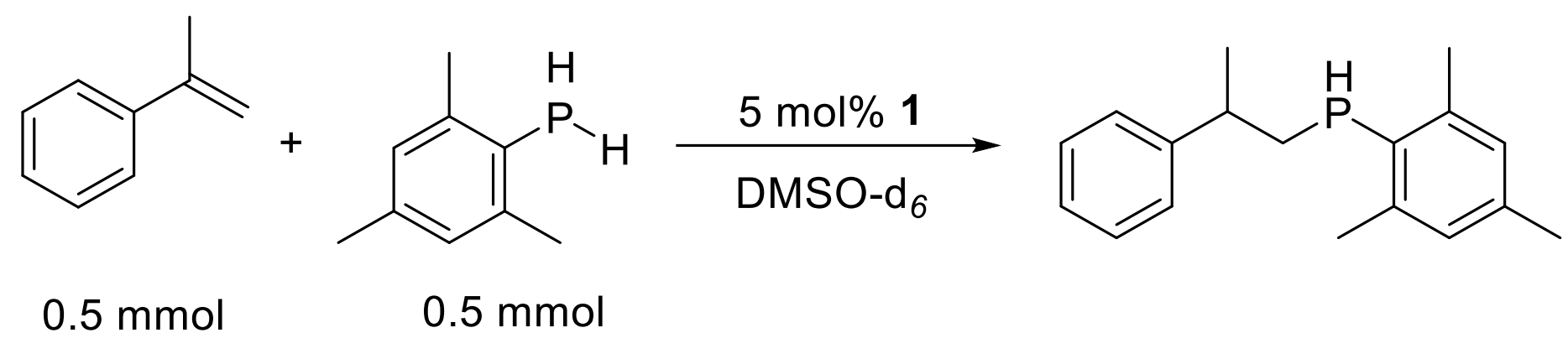

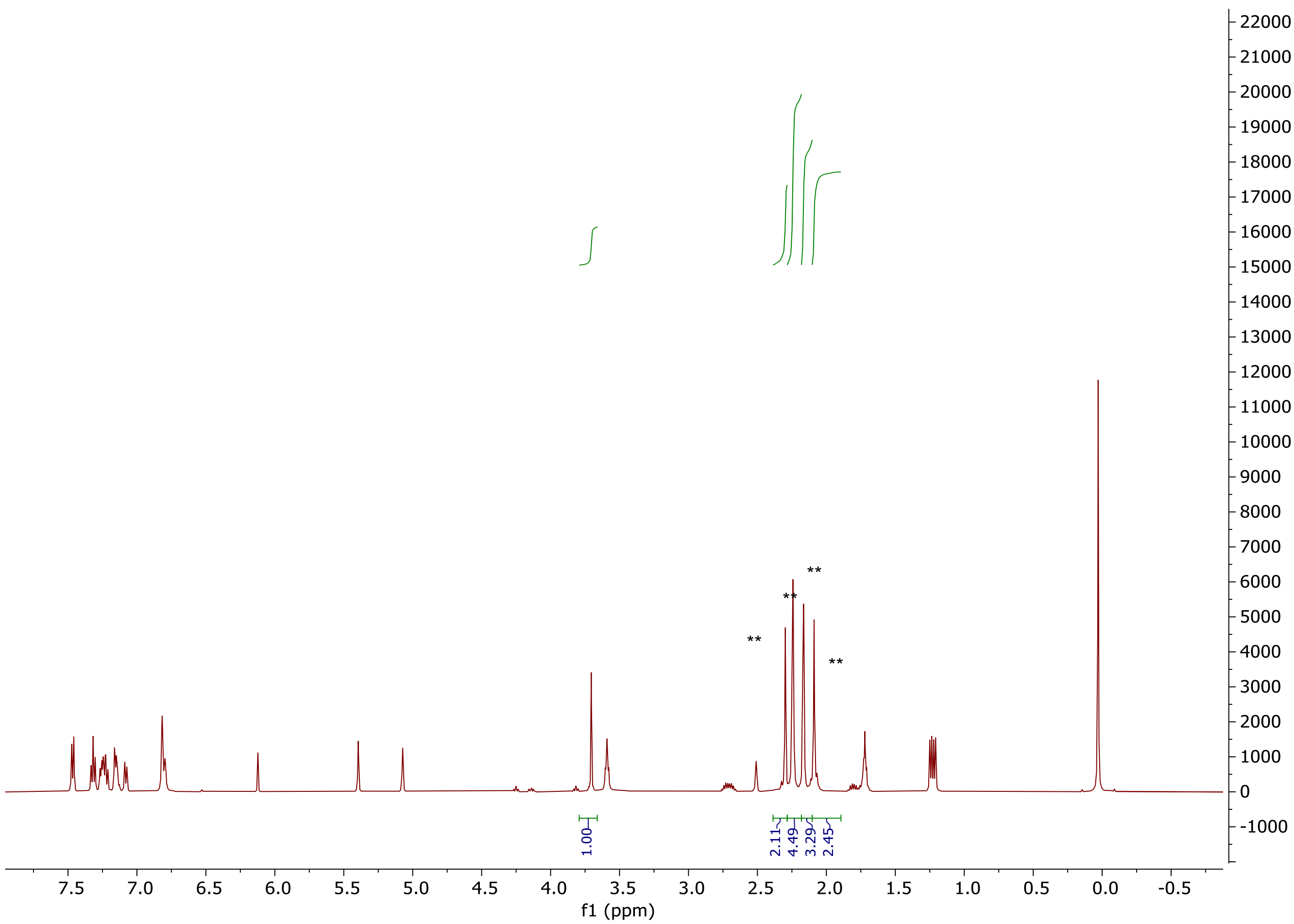

Figure S259: Hydrophosphination of 0.5 mmol of α-methyl styrene with mesitylphosphine in DMSO-d_6 using 5 mol% **1** - 0 h – ^{1}H NMR

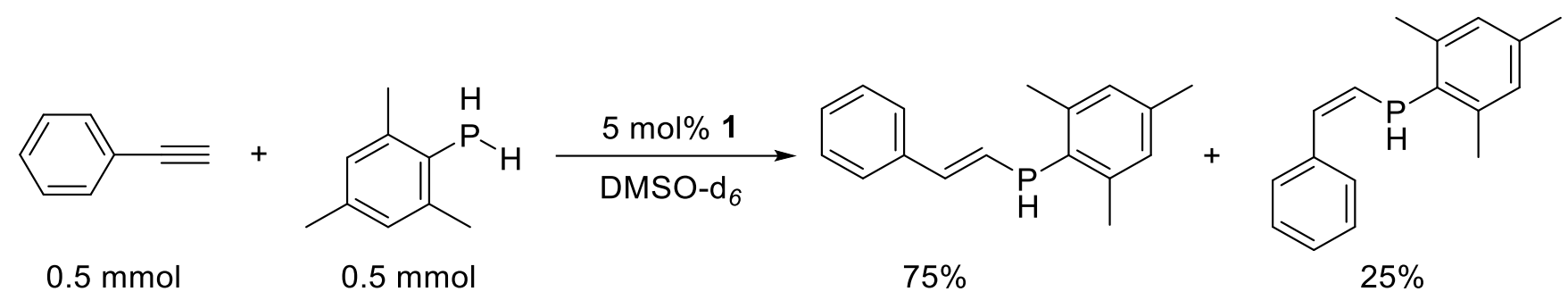

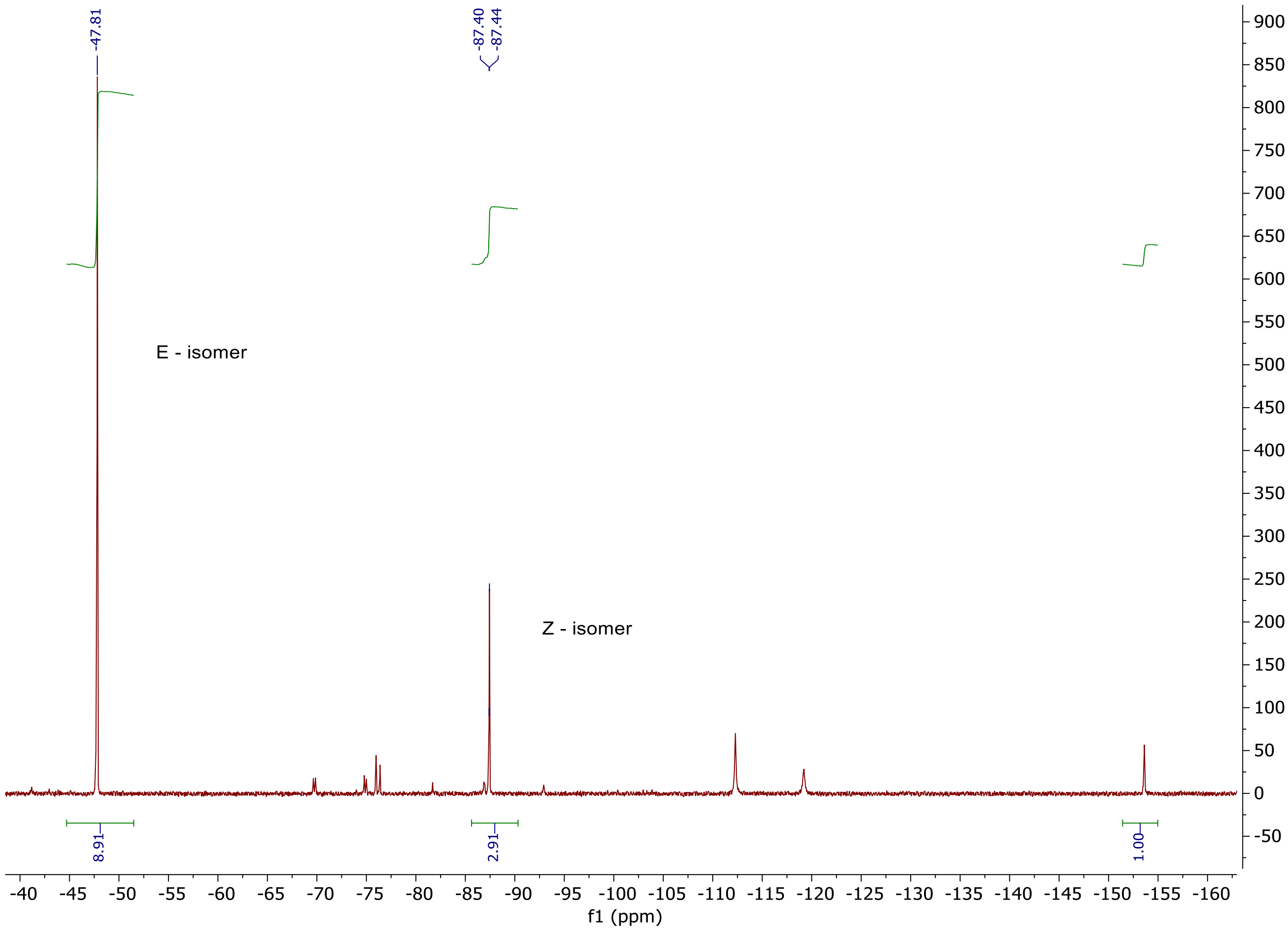

Figure S260: Hydrophosphination of 0.5 mmol of phenyl acetylene with mesitylphosphine in DMSO-d$_6$ using 5 mol% **1** - 0 h – $^{31}P\{^1H\}$ NMR

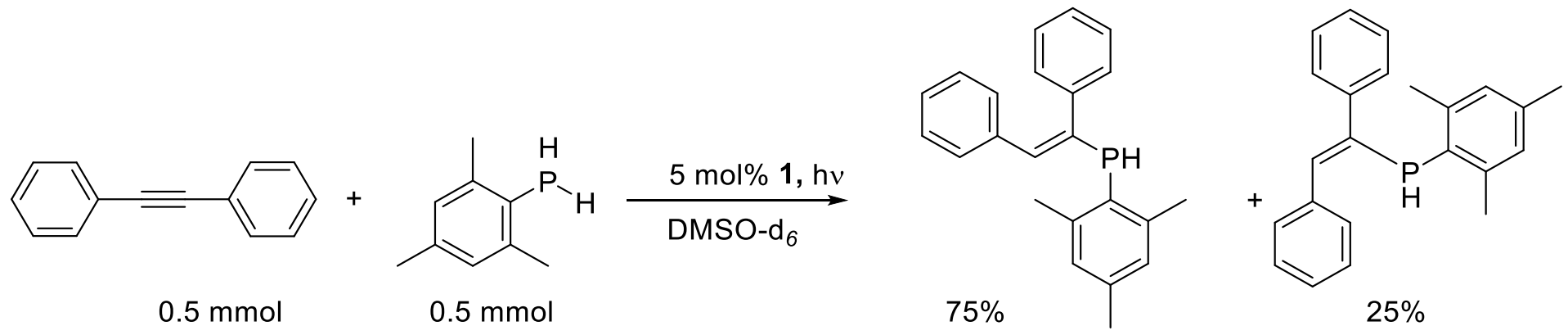

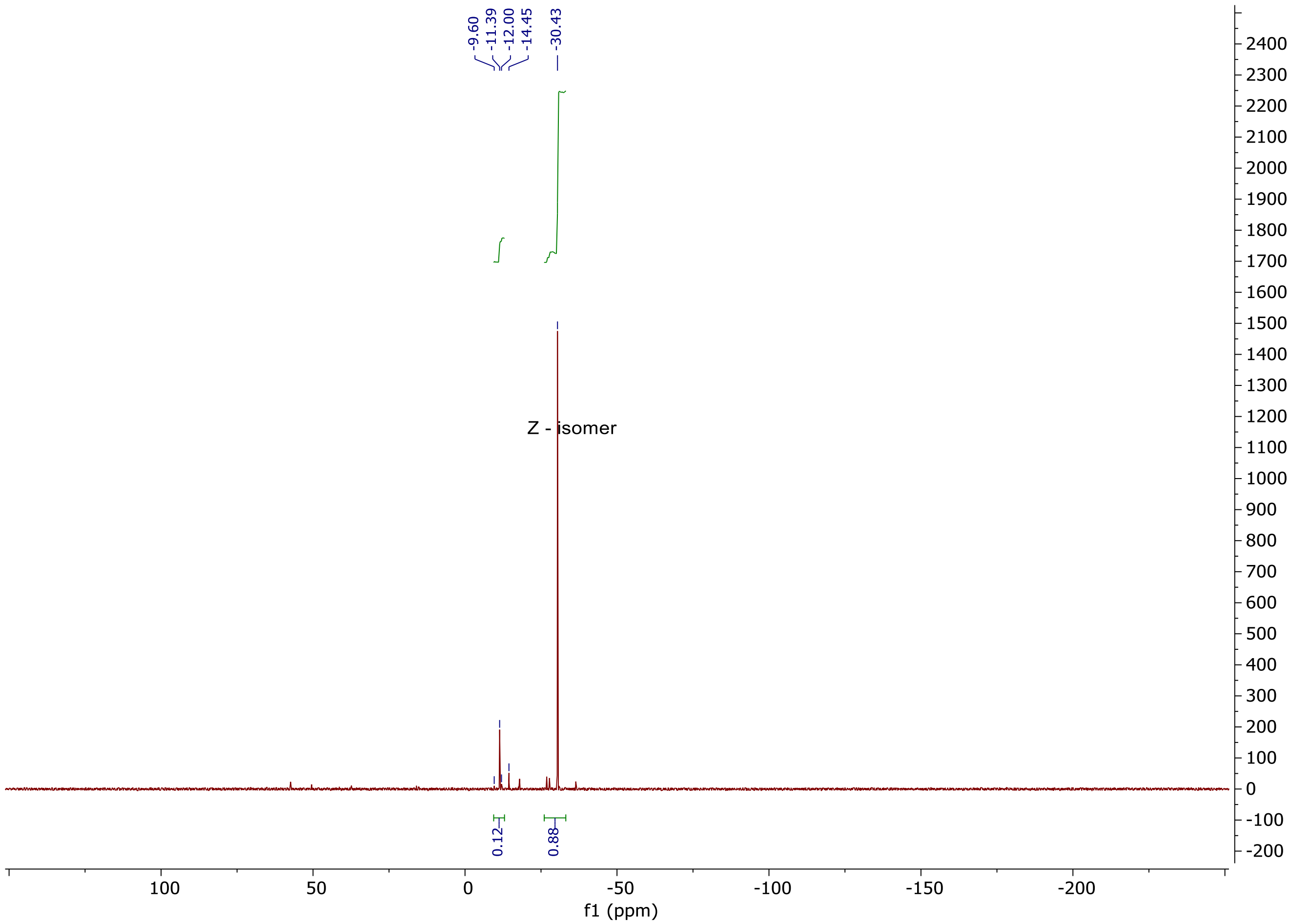

Figure S261: Hydrophosphination of 0.5 mmol of diphenyl acetylene with equimolar mesitylphosphine in DMSO-d_6 using 5 mol% **1** under UV 360 nm irradiation - 40 h –^{1}H NMR

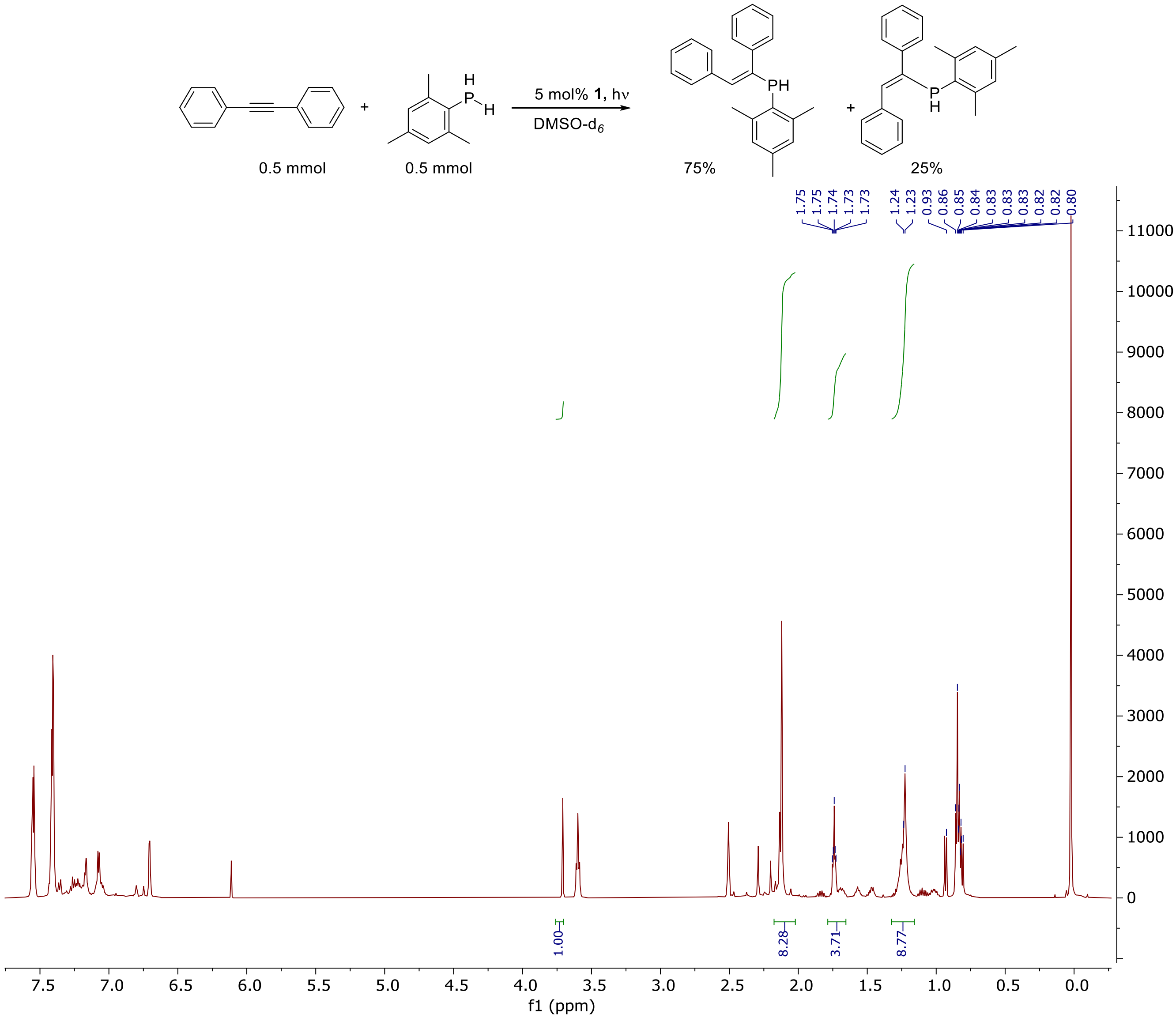

Figure S262: Hydrophosphination of 0.5 mmol of diphenyl acetylene with mesitylphosphine in DMSO-d_6 using 5 mol% **1** under UV 360 nm irradiation - 40 h –^{1}H NMR

Hydrophosphination of unsaturated compounds with ditertiary butyl phosphine in DMSO-d_6 using 5 mol% **1**

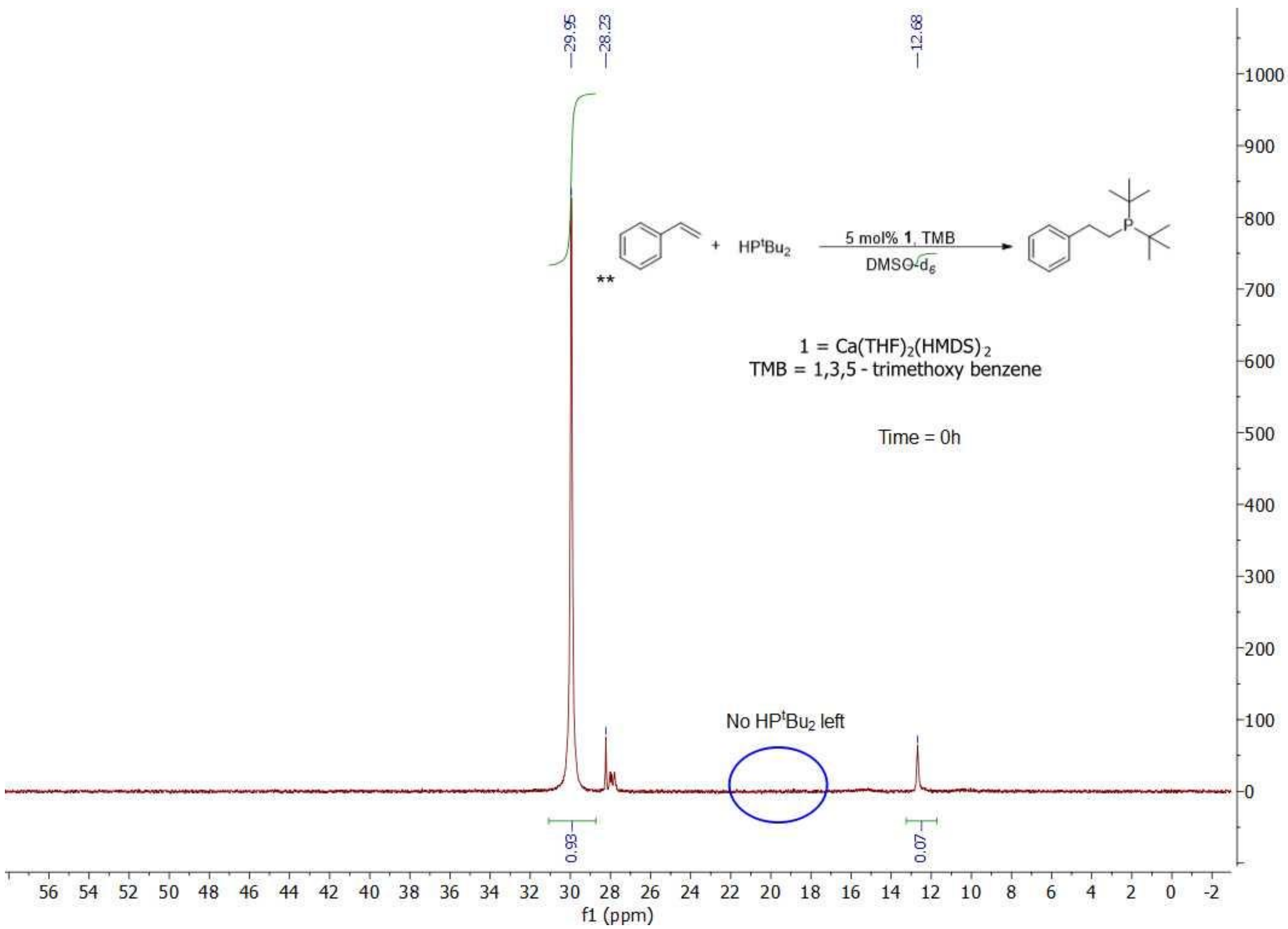

Figure S263: Hydrophosphination of 0.5 mmol of styrene with ditertiarybutyl phosphine in DMSO-d_6 using 5 mol% **1** - 0 h –$^{31}P\{^1H\}$ NMR

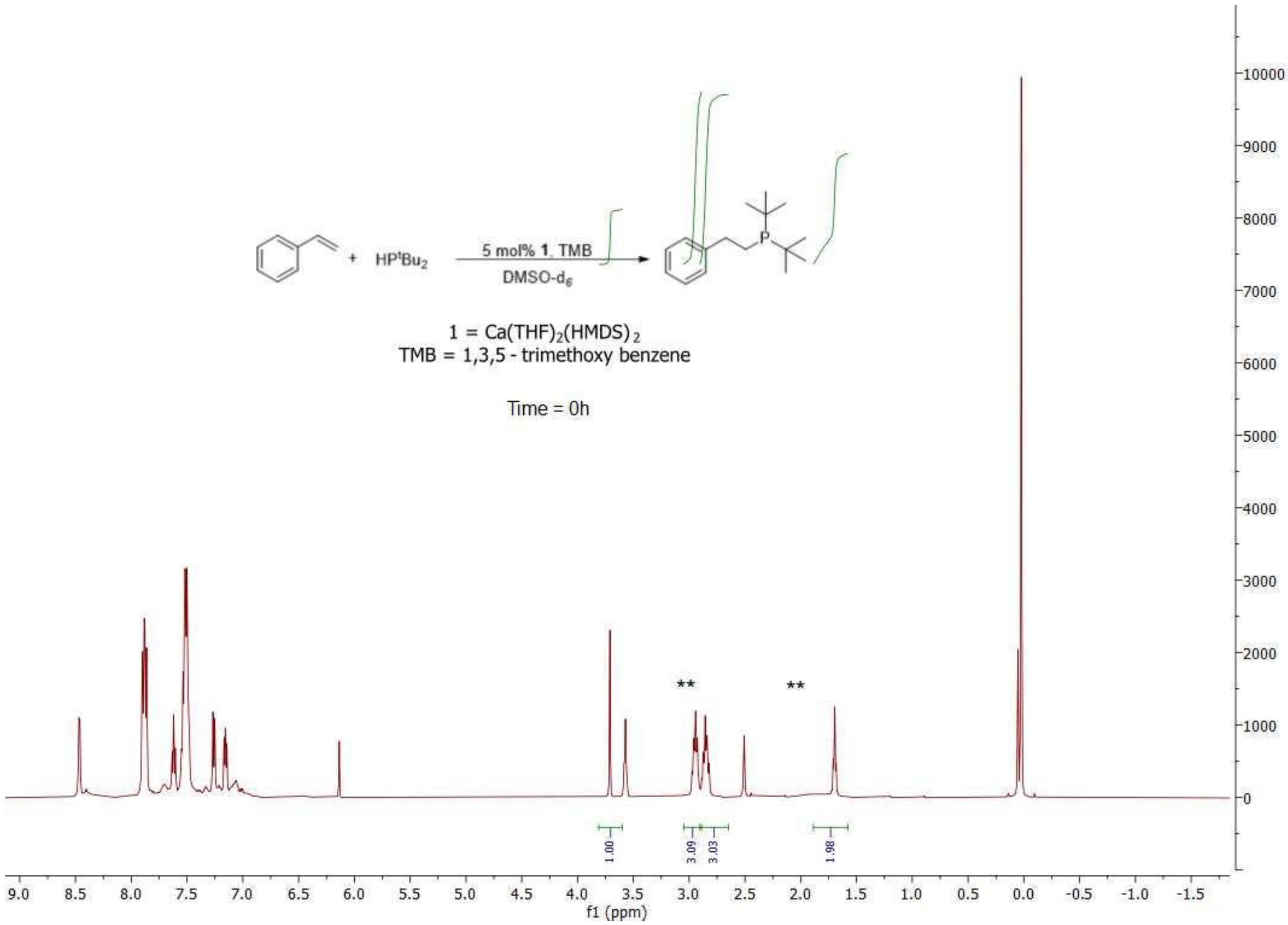

Figure S264: Hydrophosphination of 0.5 mmol of styrene with ditertiarybutyl phosphine in DMSO-d_6 using 5 mol% **1** - 0 h –^{1}H NMR

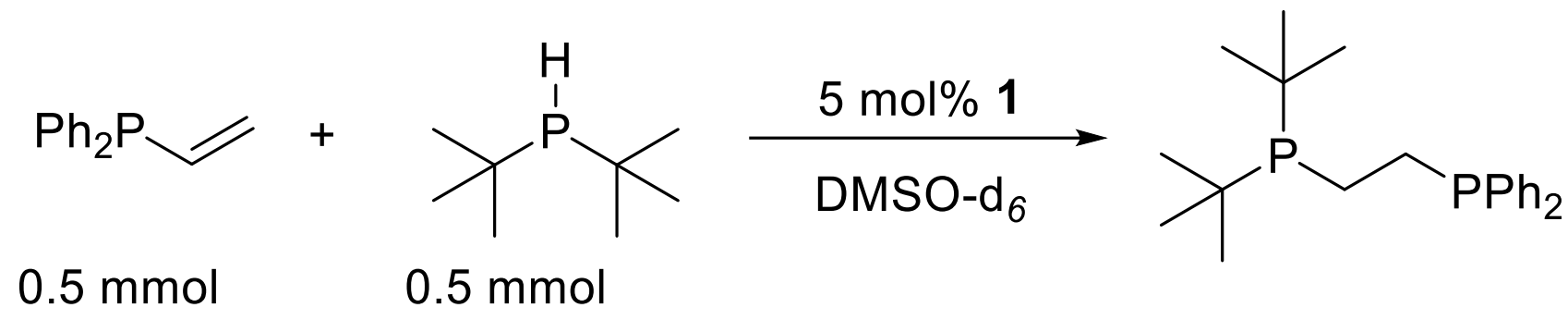

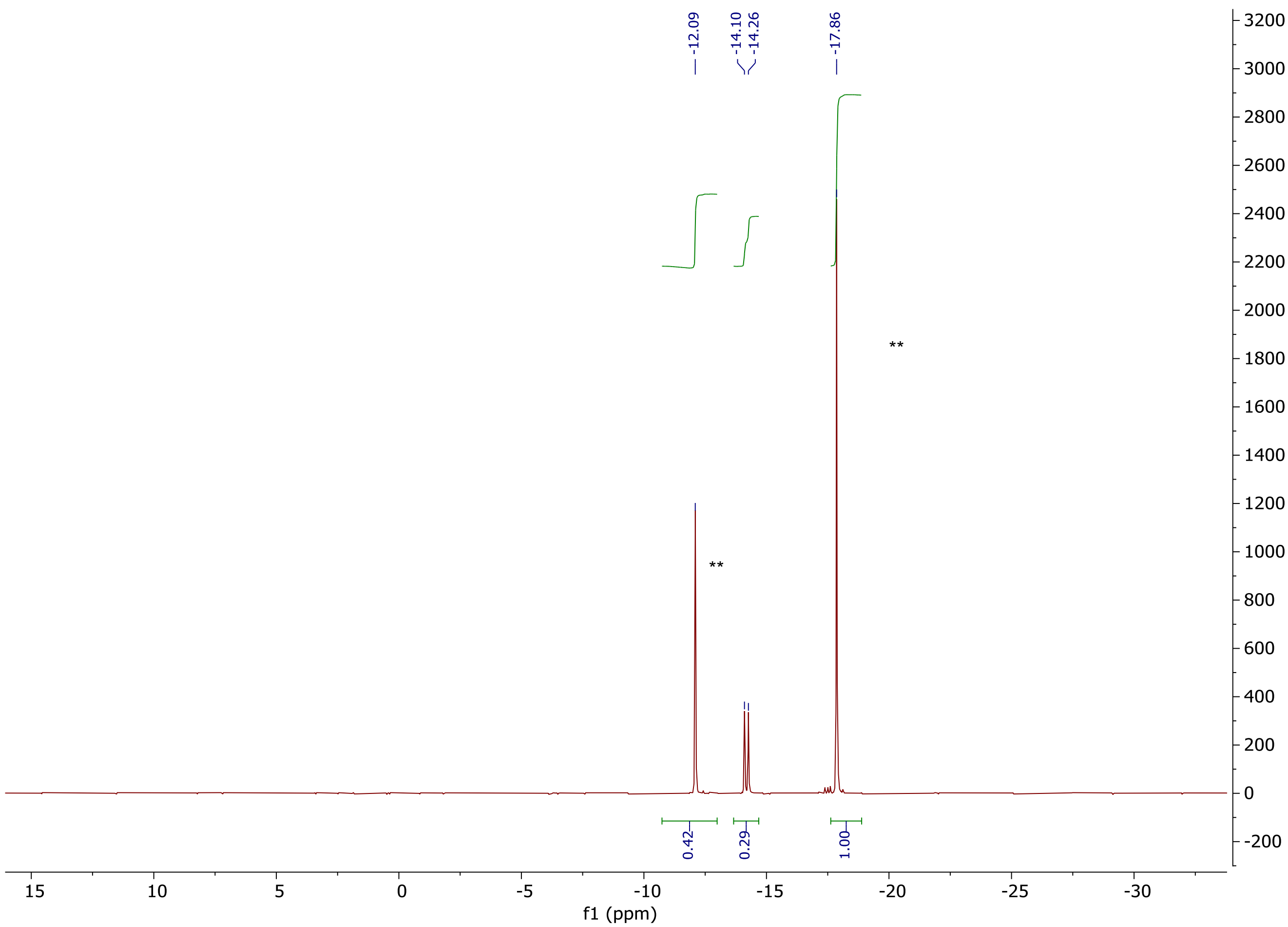

Figure S265: Hydrophosphination of 0.5 mmol of vinyl diphenylphosphine with ditertiarybutyl phosphine in DMSO-d_6 using 5 mol% **1** under UV 360 nm irradiation - 8 h – $^{31}P\{^1H\}$ NMR

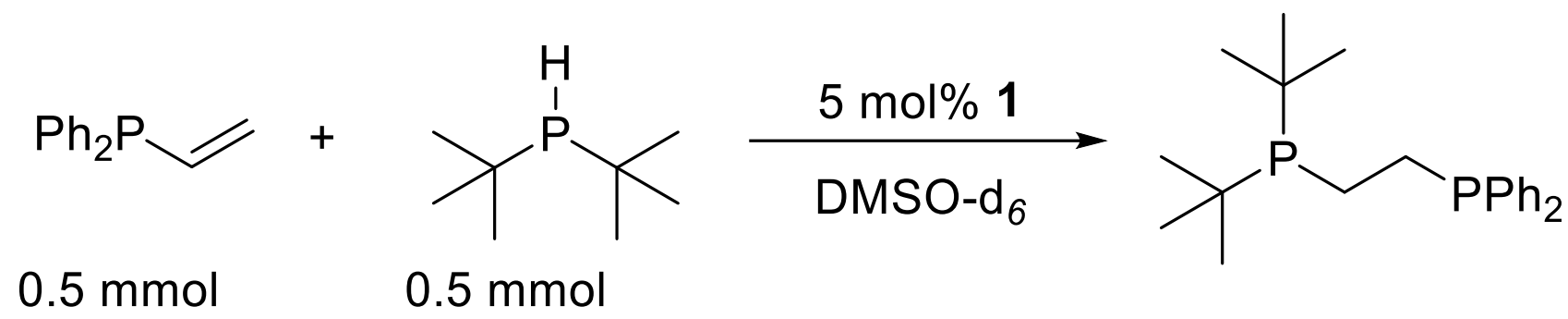

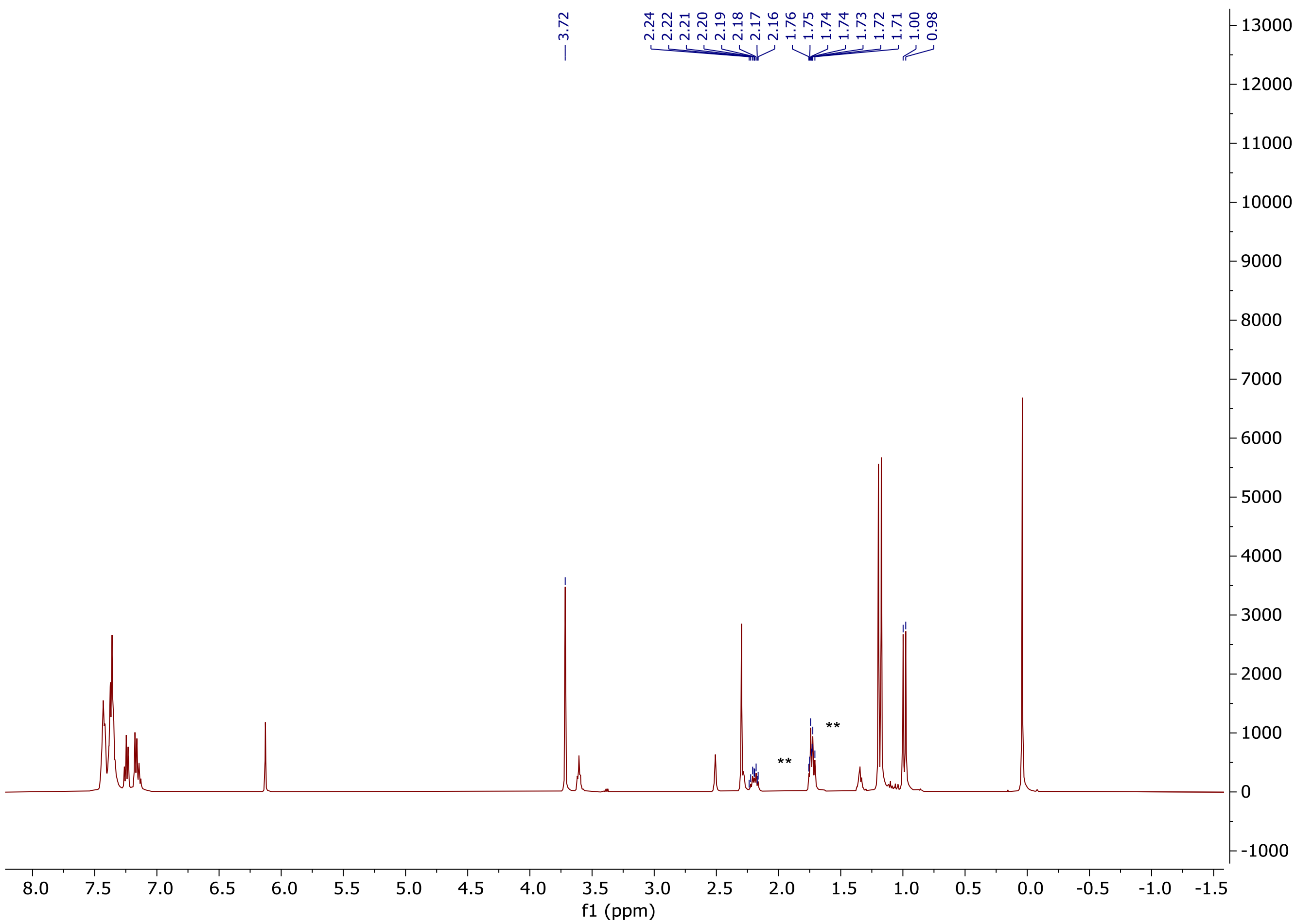

Figure S266: Hydrophosphination of 0.5 mmol of vinyl diphenylphosphine with ditertiarybutyl phosphine in DMSO-d$_6$ using 5 mol% **1** under UV 360 nm irradiation - 8 h – ^{31}P{^{1}H} NMR

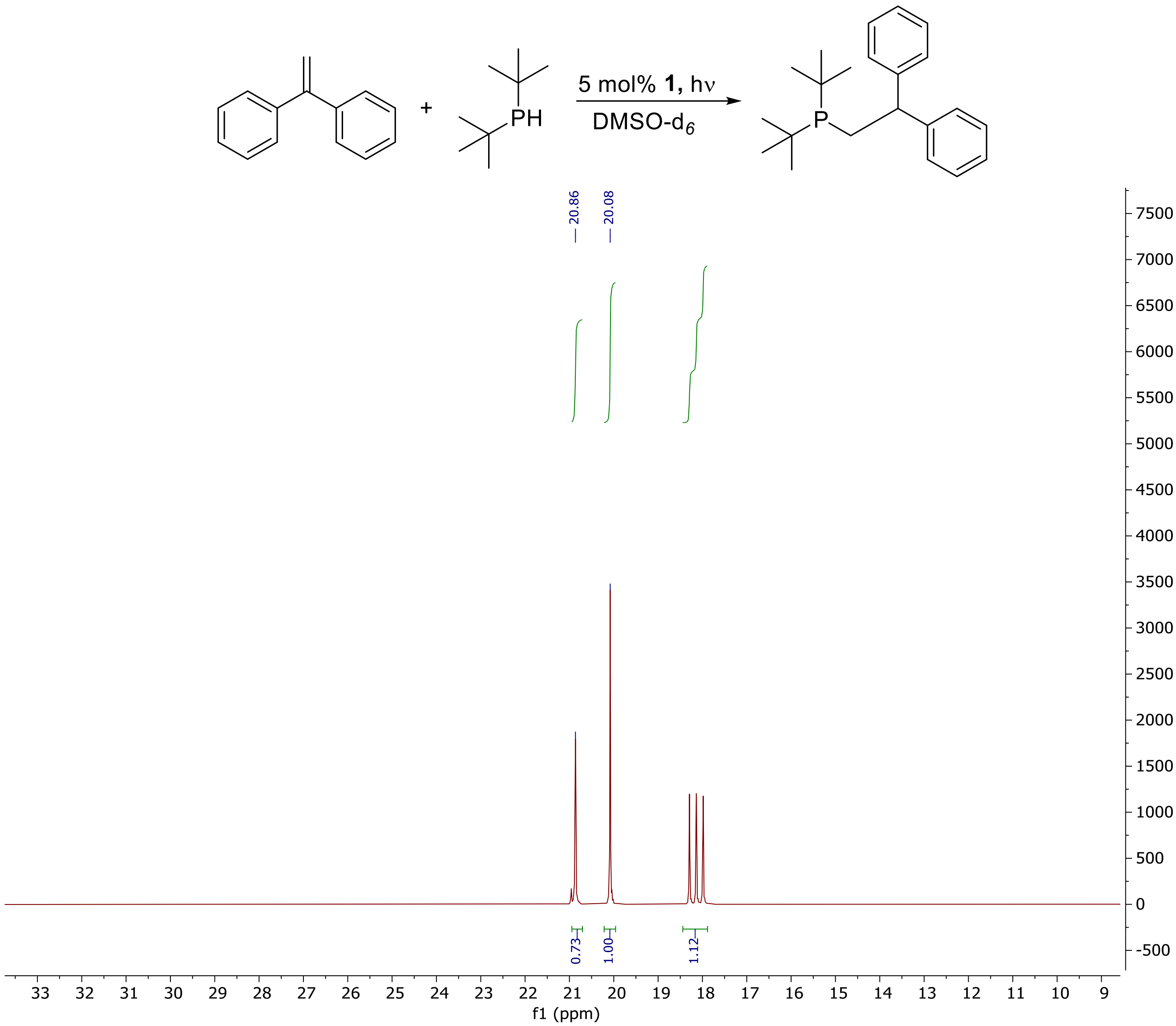

Figure S267: Hydrophosphination of 0.5 mmol of 1,1-diphenyl ethene with ditertiarybutyl phosphine in DMSO-d$_6$ using 5 mol% **1** under UV 360 nm irradiation- 0 h – ^{31}P{^{1}H} NMR

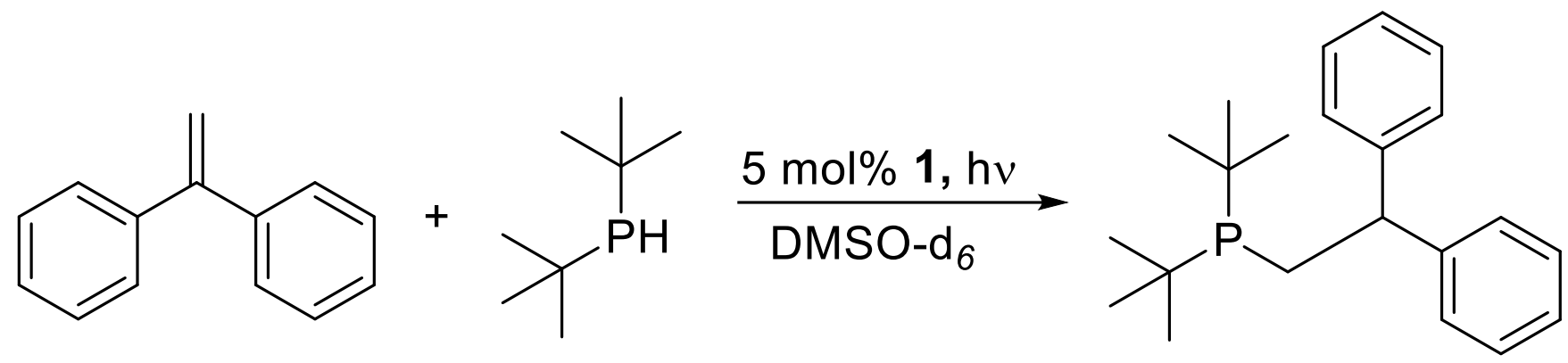

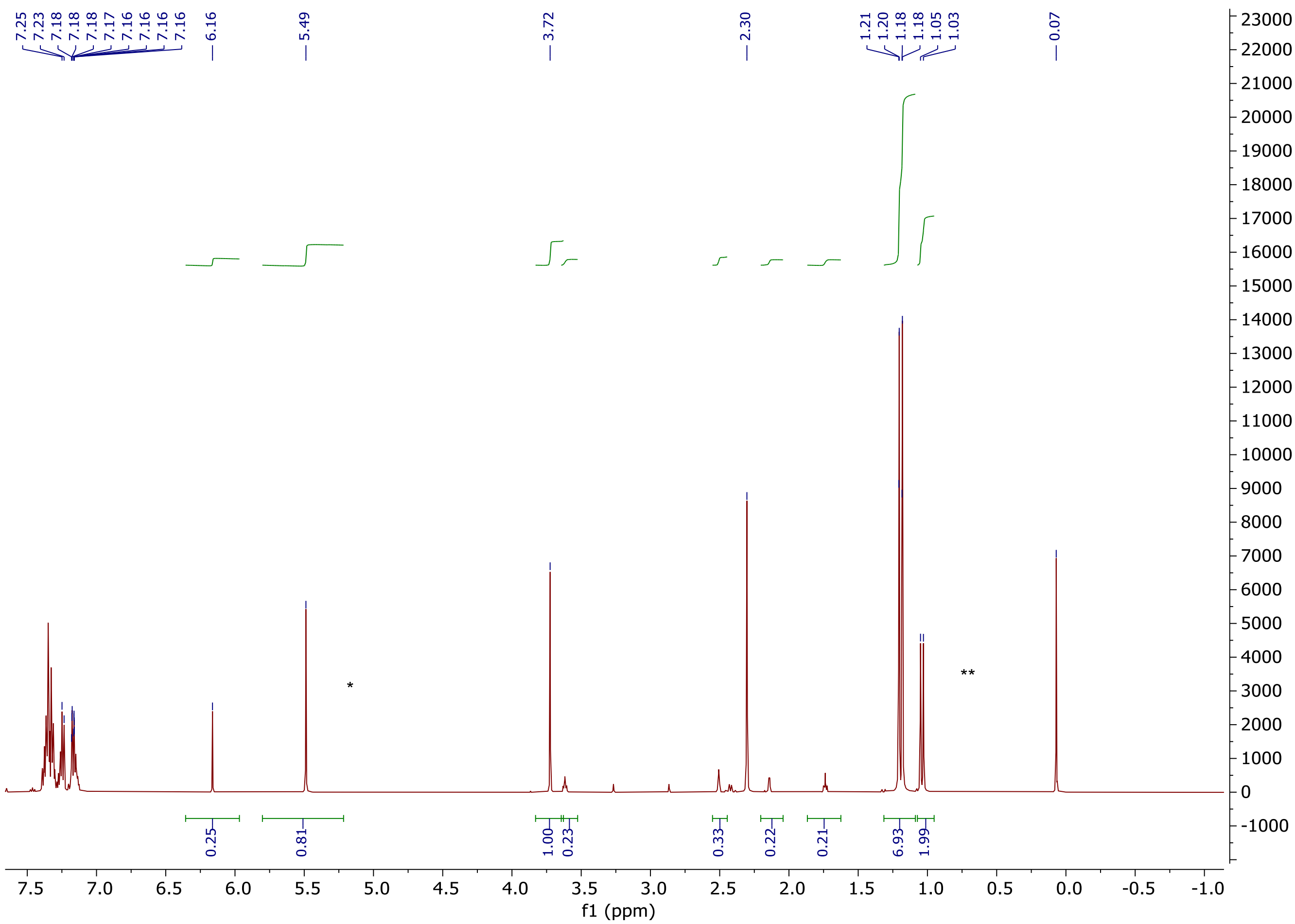

Figure S268: Hydrophosphination of 0.5 mmol of 1,1-diphenyl ethene with ditertiarybutyl phosphine in DMSO-d_6 using 5 mol% **1** under UV 360 nm irradiation - 0 h –^{1}H NMR

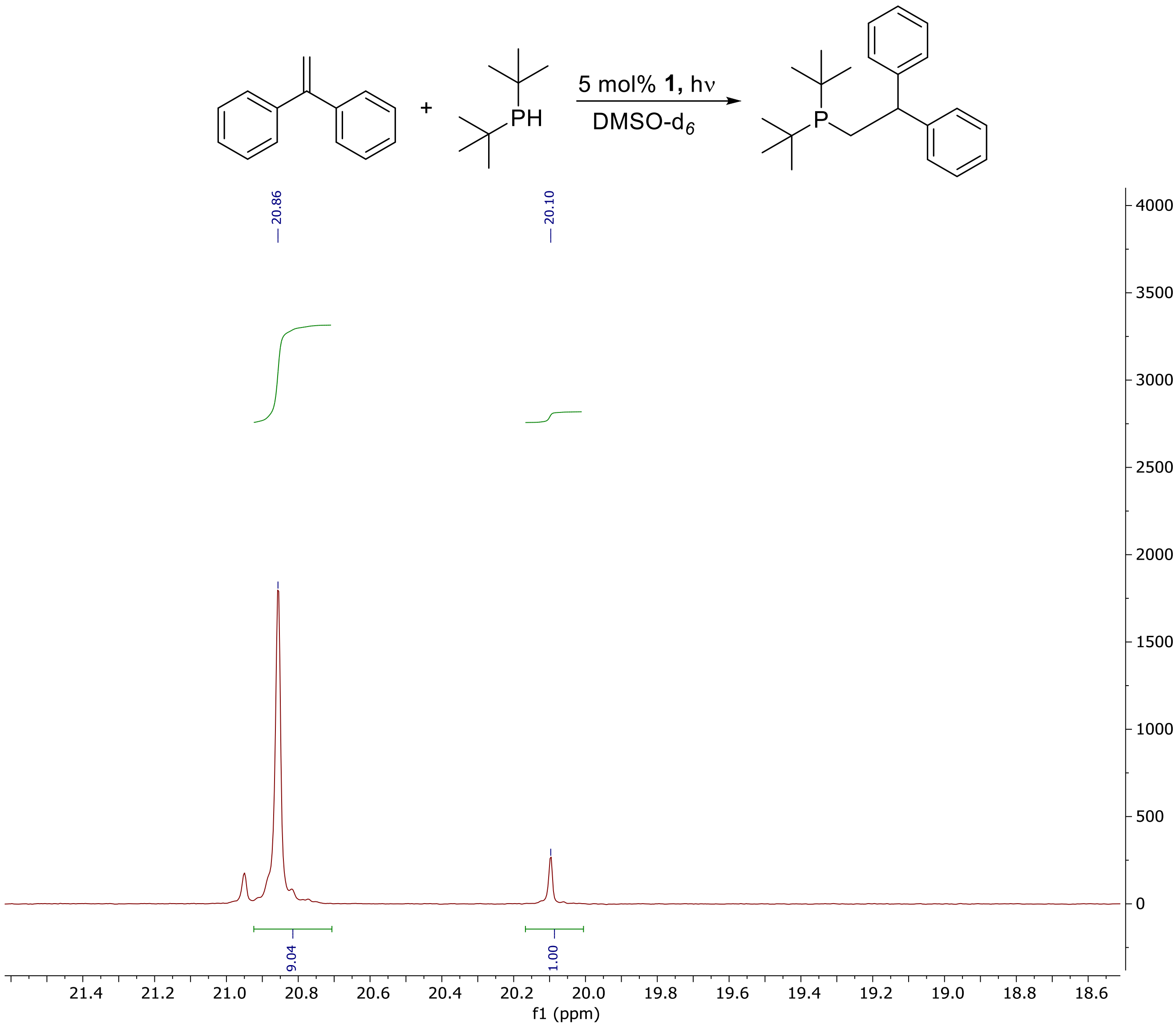

Figure S269: Hydrophosphination of 0.5 mmol of 1,1-diphenyl ethene with ditertiarybutyl phosphine in DMSO-d_6 using 5 mol% **1** under UV 360 nm irradiation - 2 h – $^{31}P\{^1H\}$ NMR

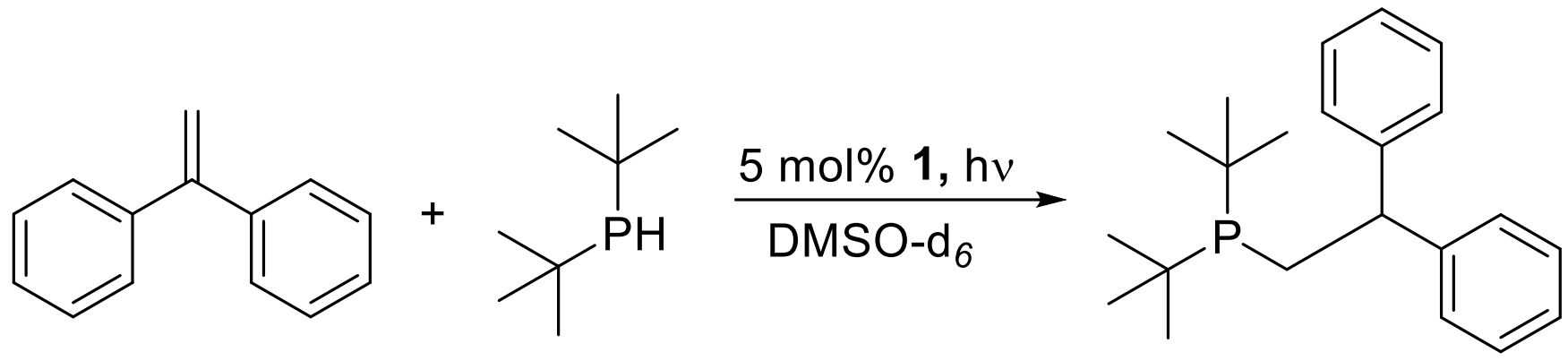

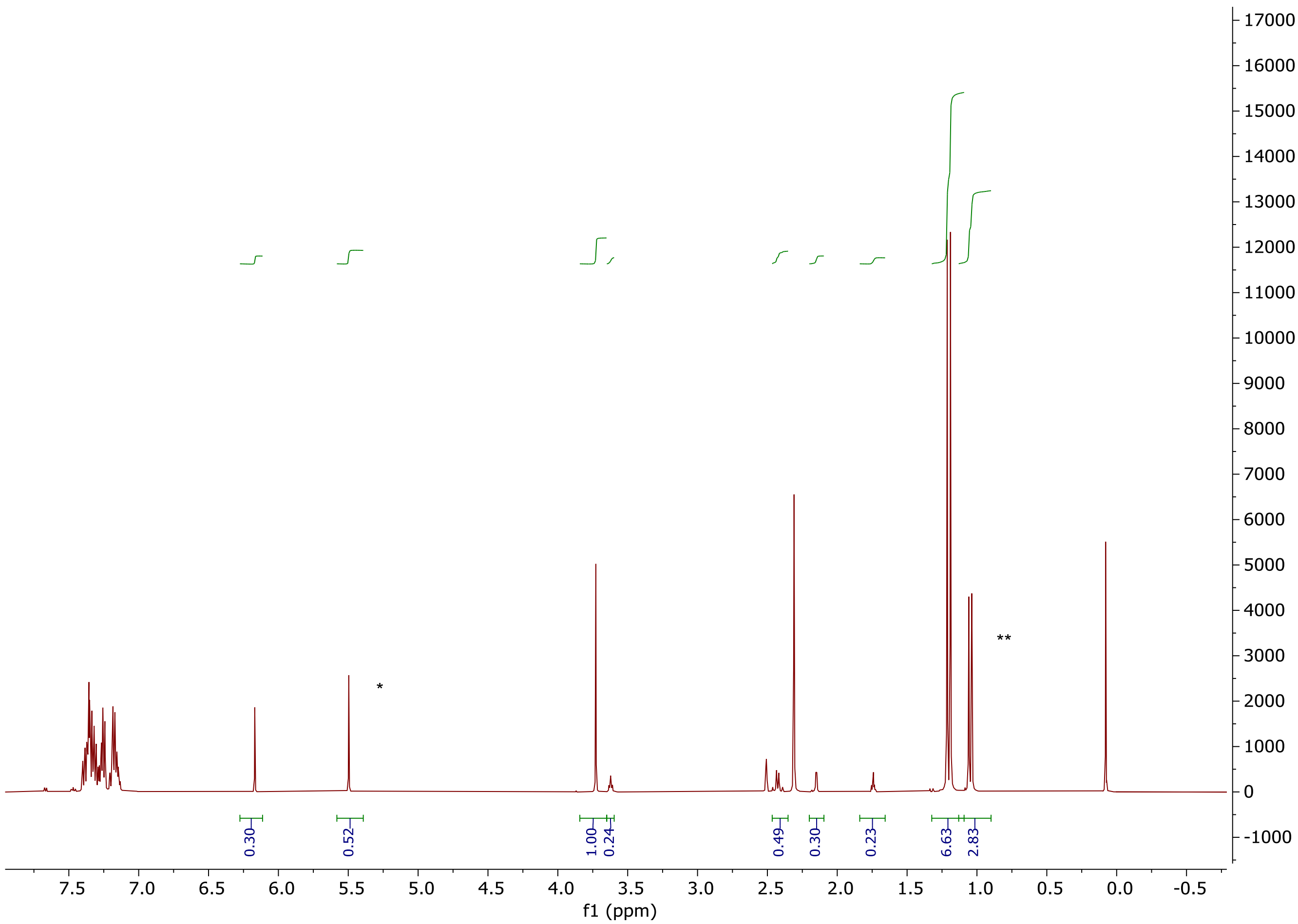

Figure S270: Hydrophosphination of 0.5 mmol of 1,1-diphenyl ethene with ditertiarybutyl phosphine in DMSO-d$_6$ using 5 mol% **1** under UV 360 nm irradiation - 2 h – ^{1}H NMR

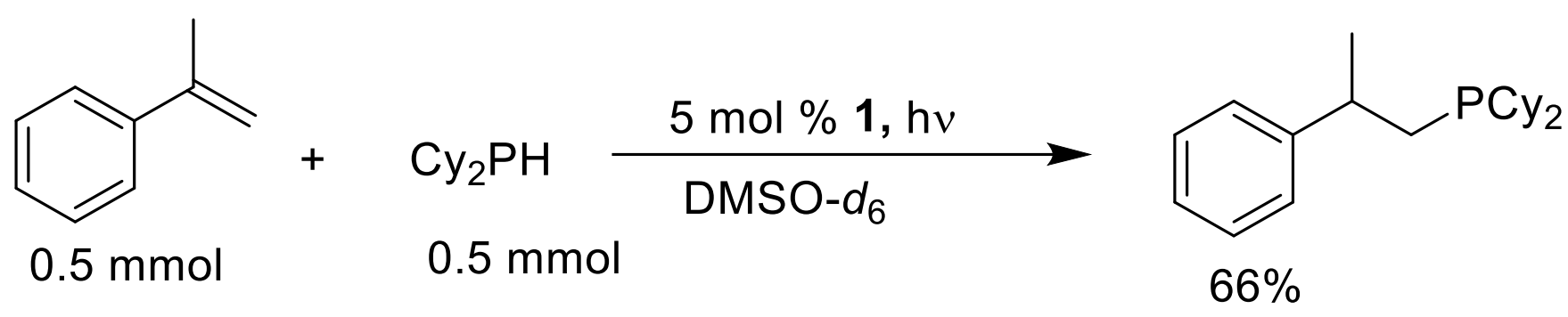

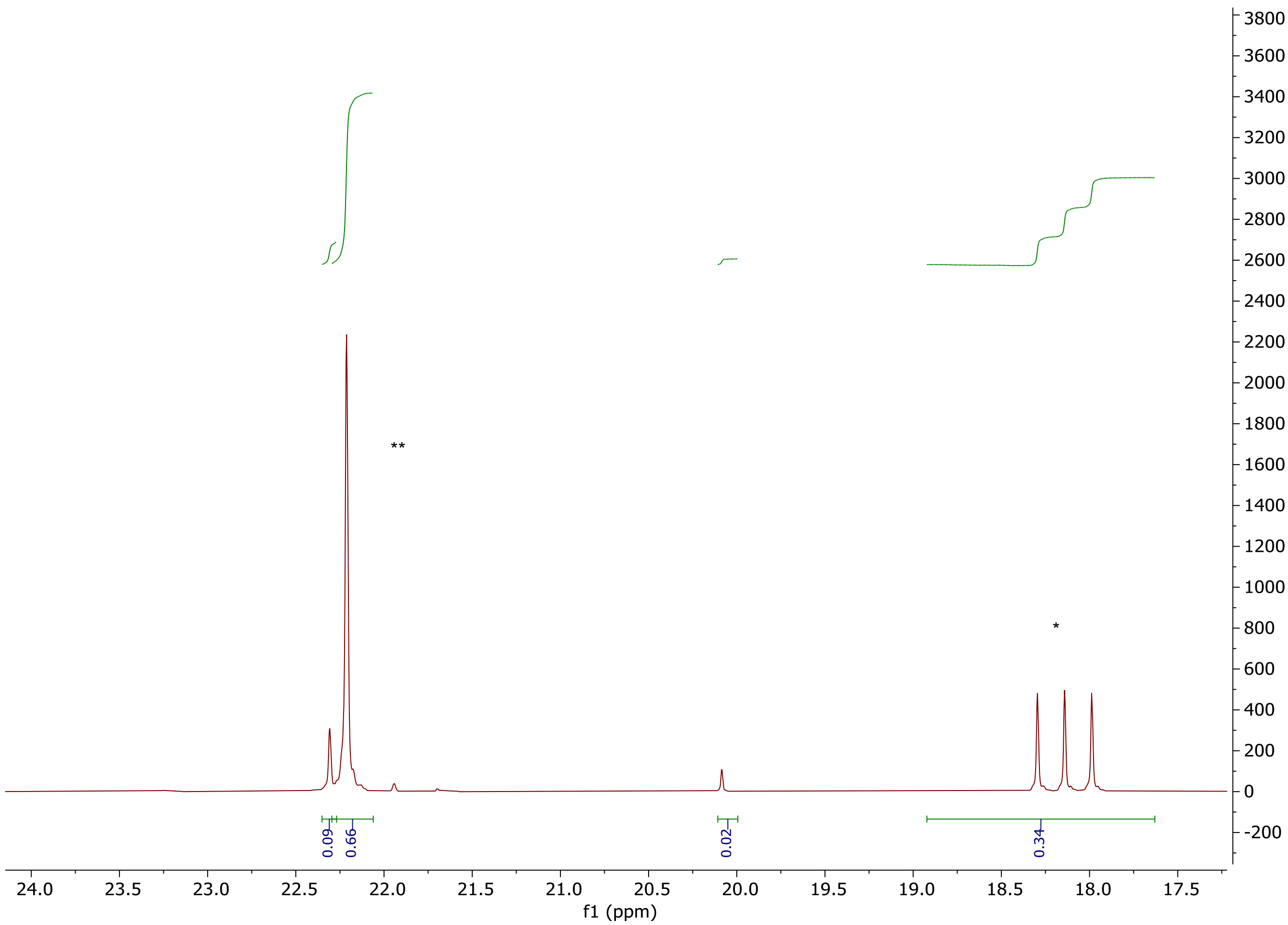

Figure S271: Hydrophosphination of 0.5 mmol of α-methyl styrene with ditertiarybutyl phosphine in DMSO-d_6 using 5 mol% **1** under UV 360 nm irradiation - 48 h – $^{31}P\{^1H\}$ NMR

Inside a nitrogen glovebox, to a J-Young tube, 0.4 ml of DMSO-d_6 was charged, and 0.5 mmol of diphenylphosphine, 5 equivalent of benzaldehyde were added subsequently. In the reaction mixture, 5 mol% of 1 was added and NMR was taken immediately. According to $^{31}P\{^1H\}$ NMR spectroscopy, all diphenylphosphine was consumed. The nucleophilic addition product of benzaldehyde and diphenylphosphine $Ph_2PCH(Ph)OH$ was observed near quantitative conversion. The reaction mixture was again brought back to the glovebox, and charged with 0.5 mmol of methylacrylate, and NMR was taken immediately. The product 3a was found having 85% yield.

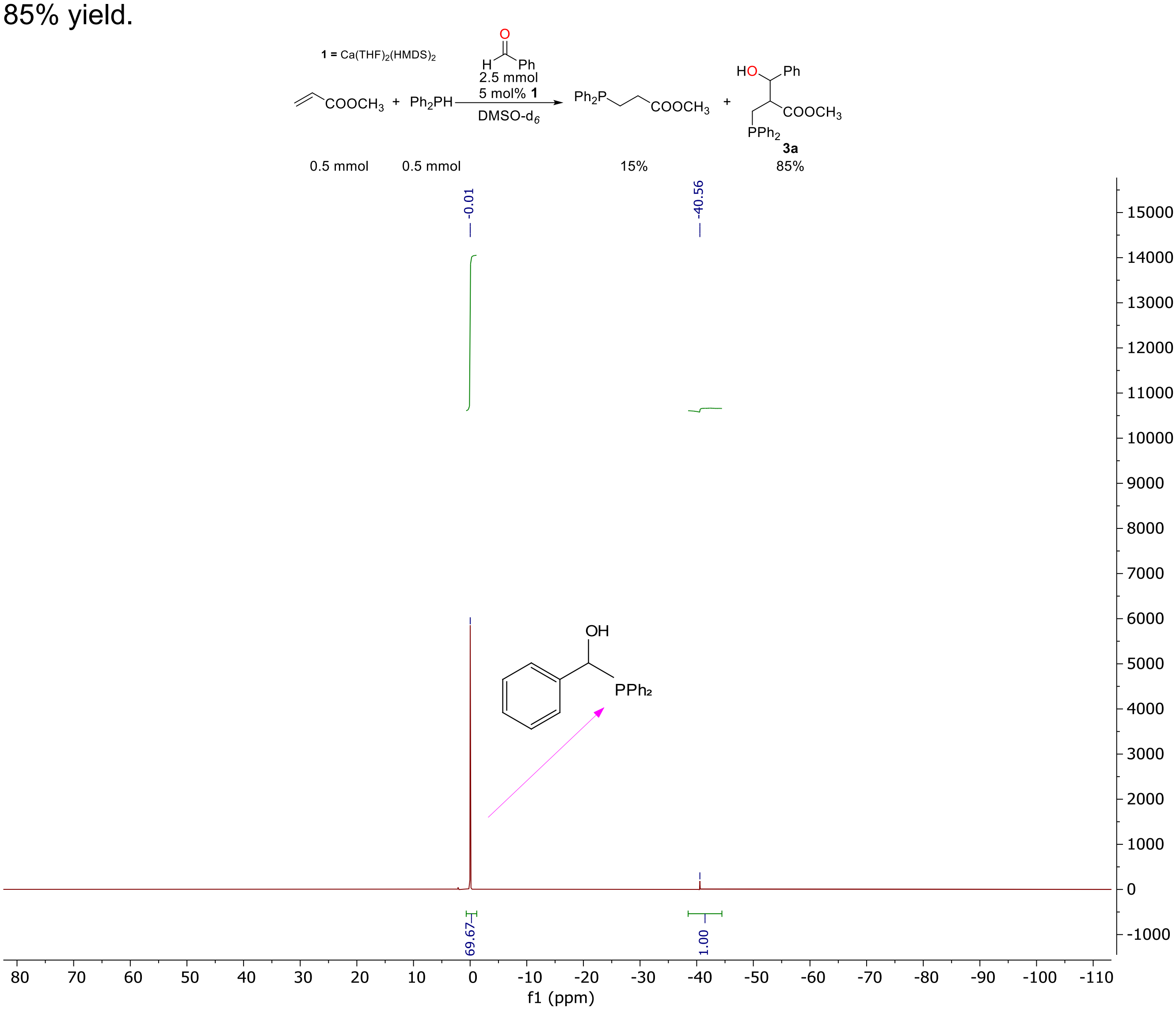

Figure S272: $^{31}P\{^1H\}$ NMR spectra of 2.5 mmol of benzaldehyde, 0.5 mmol of diphenylphosphine mixture in DMSO-d_6 in presence of 5 mol% **1** at ambient conditions**.**

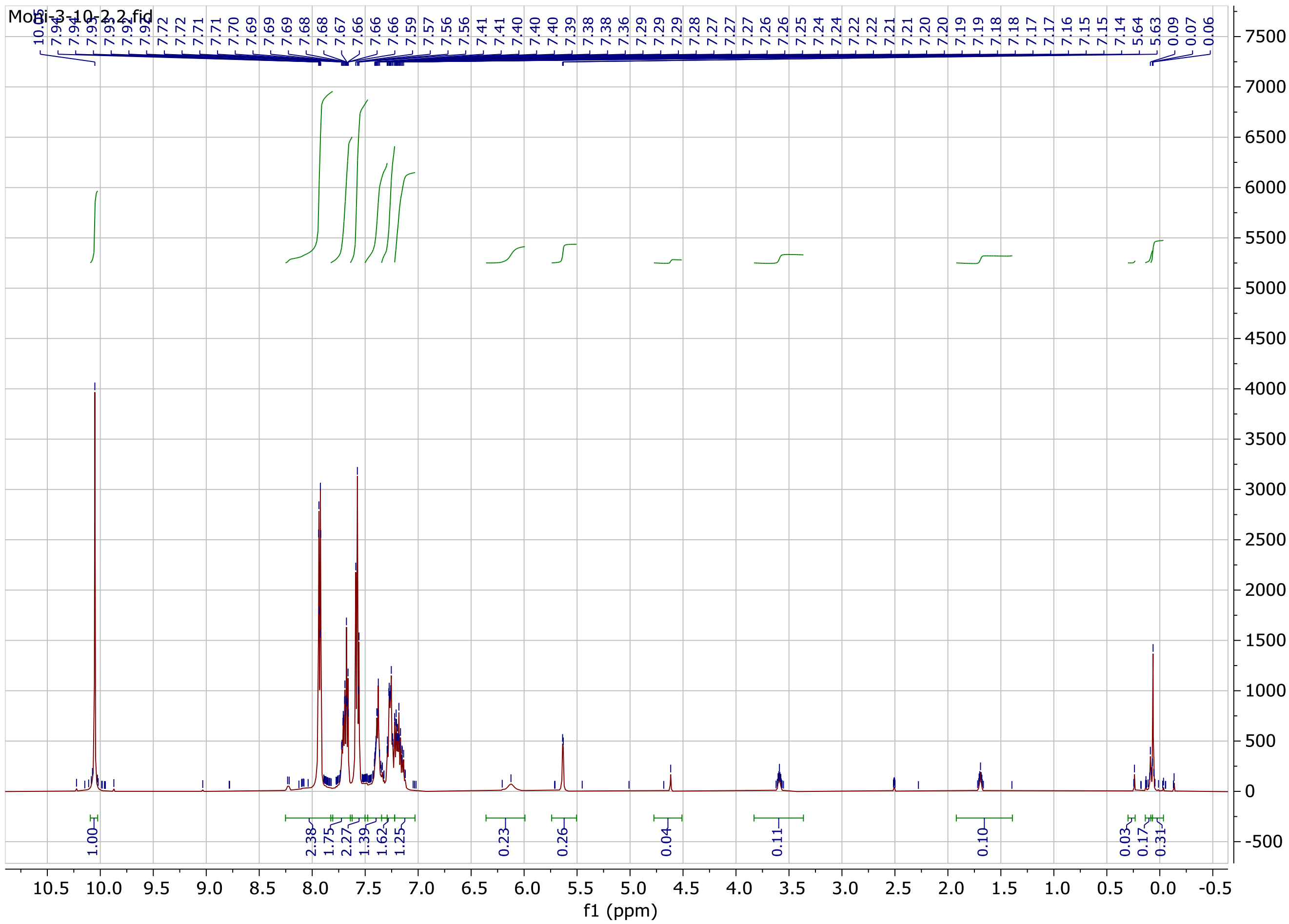

Figure S273: ^{1}H NMR spectra of 2.5 mmol of benzaldehyde, 0.5 mmol of diphenylphosphine mixture in DMSO-d_6 in presence of 5 mol% **1** at ambient conditions**.**

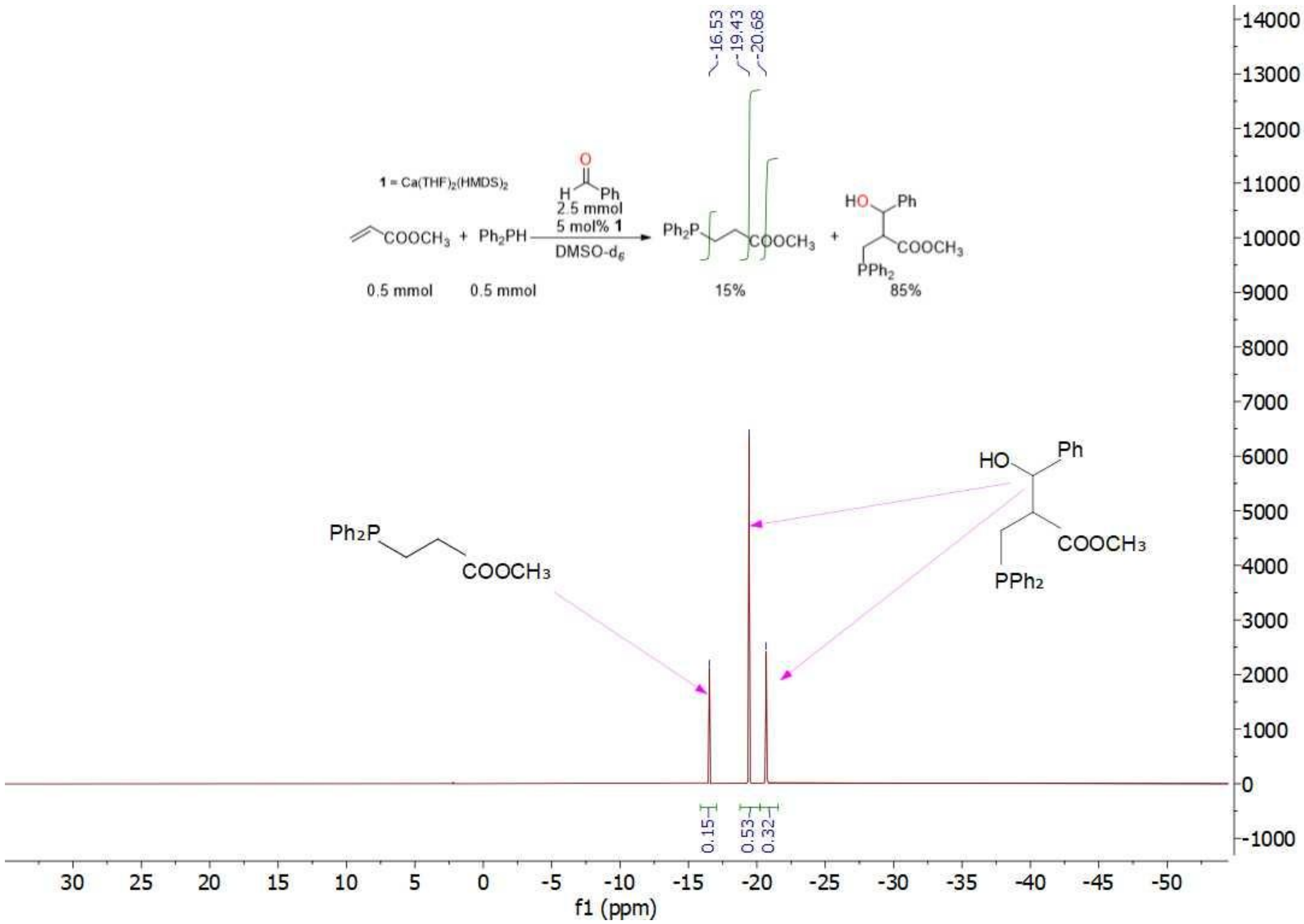

Figure S274: $^{31}P\{^{1}H\}$ NMR spectra of 2.5 mmol of benzaldehyde, 0.5 mmol of diphenylphosphine, 0.5 mmol of methyl acrylate mixture in DMSO-d_6 in presence of 5 mol% **1** at ambient conditions**.**

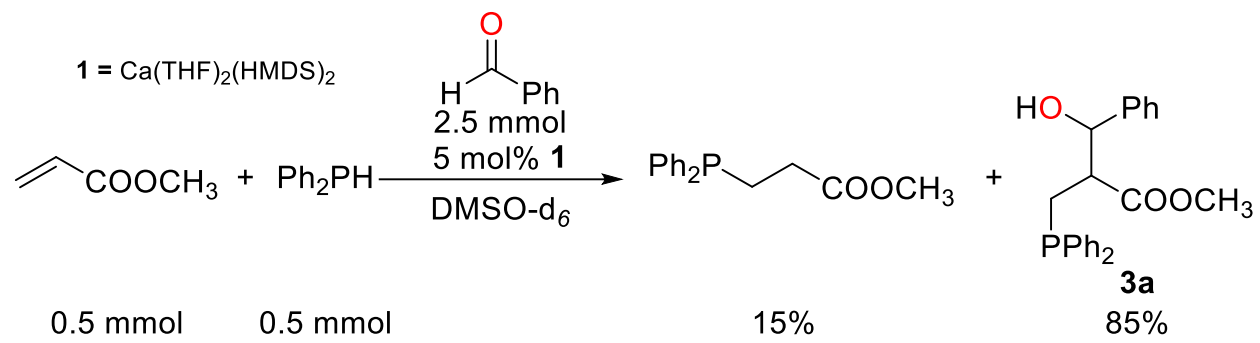

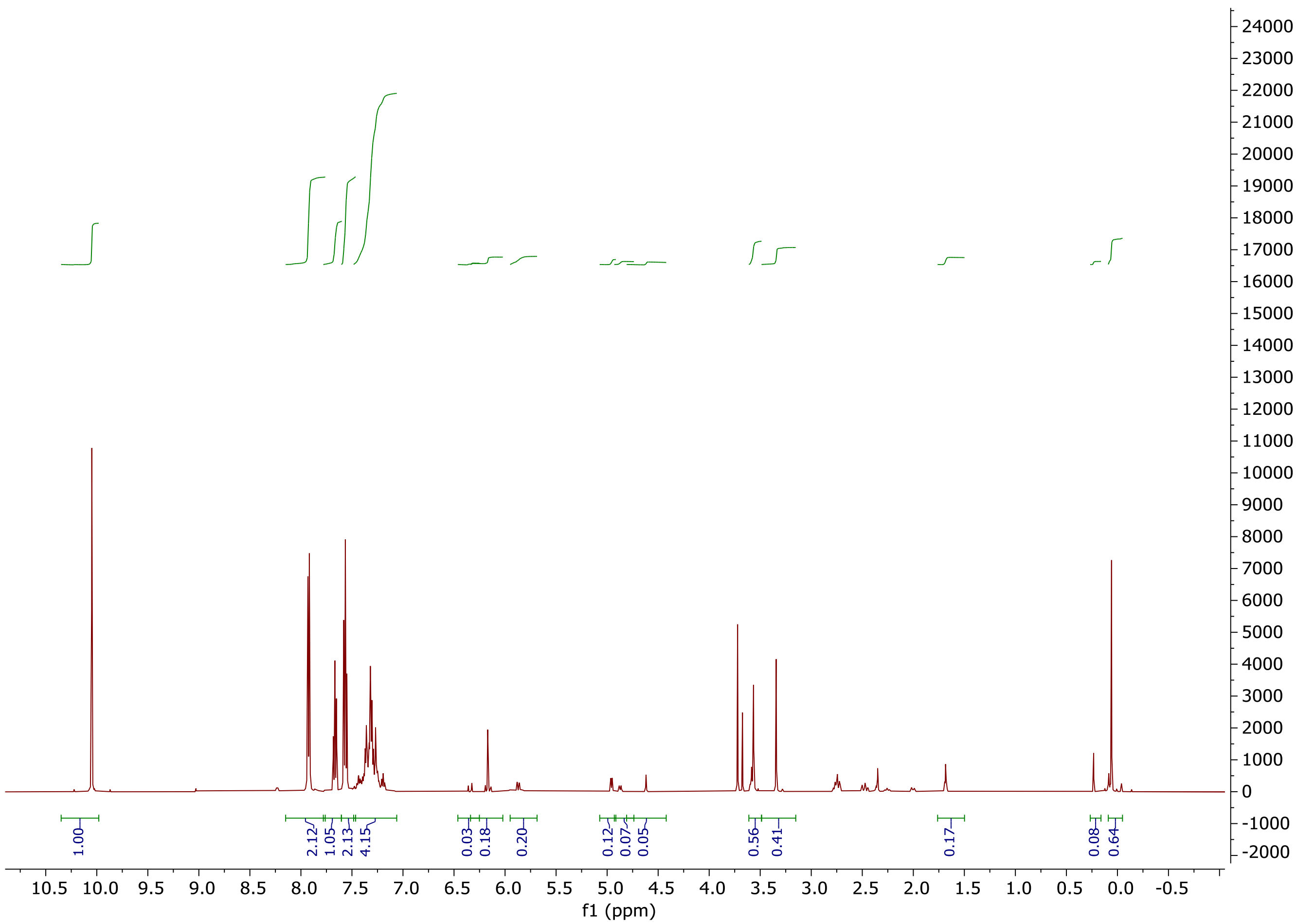

Figure S275: ^{1}H NMR spectra of 2.5 mmol of benzaldehyde, 0.5 mmol of diphenylphosphine, 0.5 mmol of methylacrylate mixture in DMSO-d_6 in presence of 5 mol% **1** at ambient conditions.

Radical trapping experiments

NMR Spectra for chloromethyl cyclopropane as radical trapping reagent

Prepared according to general procedures in DMSO-d$_6$, 0.5 mmol of styrene, 0.5 mmol of diphenylphosphine, 1 mmol of chloromethyl cyclopropane in presence of 5 mol% **1**. The reaction was monitored in three different time points under similar conditions. Initially 30% (diphenylphosphido methyl) cyclopropane was detected with only 4% phenethyl diphenylphosphine, and no 1-butene was detected. The hydrophosphination product yield increased to 33% after 60 h while 39% (diphenylphosphido methyl) cyclopropane with no olefinic signals in the 1H NMR.

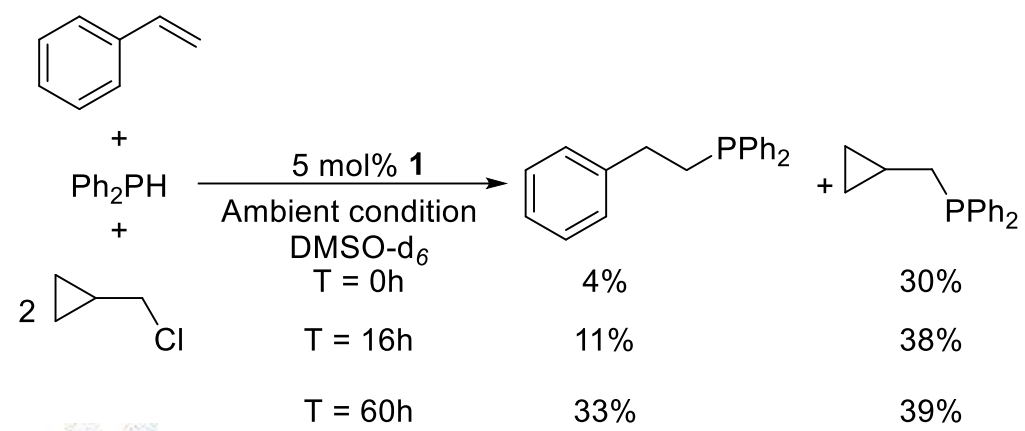

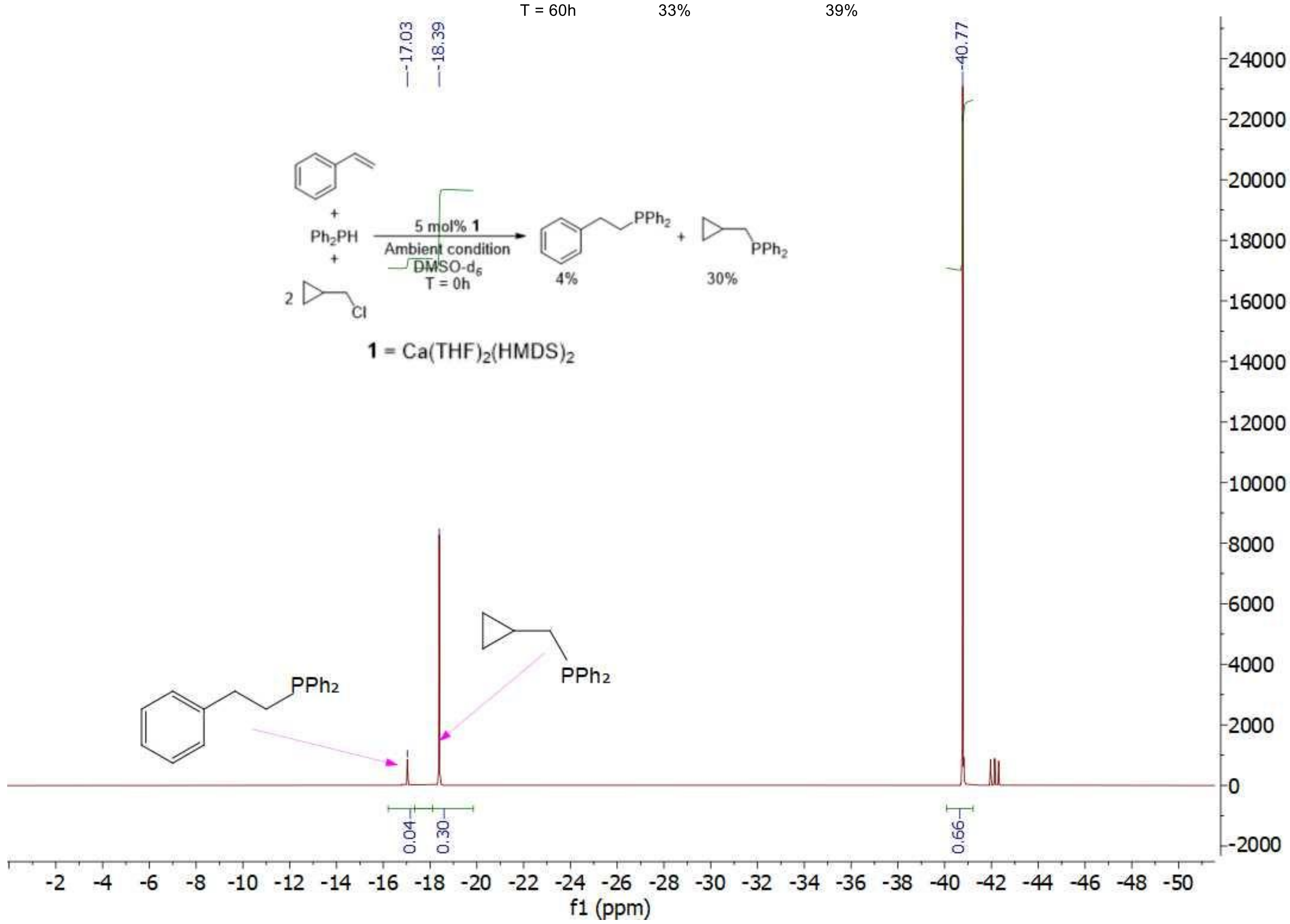

Figure S276: Radical trapping experiment using 2 equivalents of chloromethyl cyclopropane in the standard reaction conditions at ambient temperature – 0 h – $^{31}P\{^1H\}$ NMR

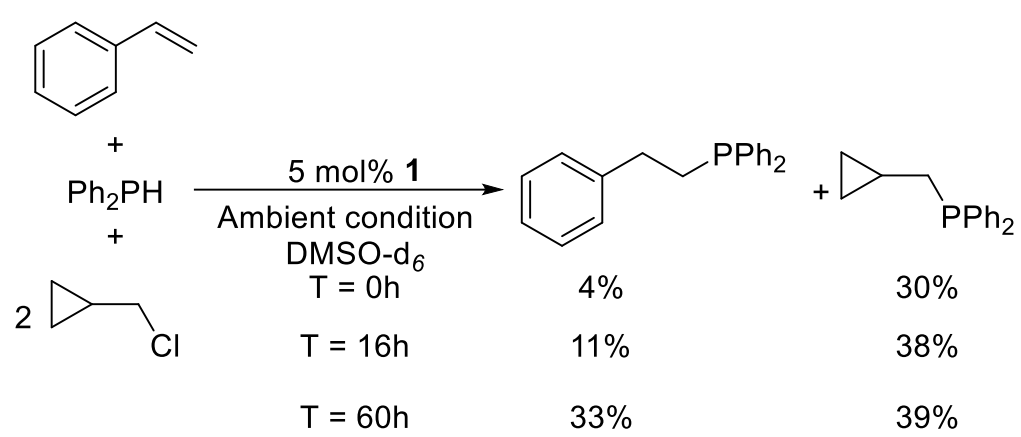

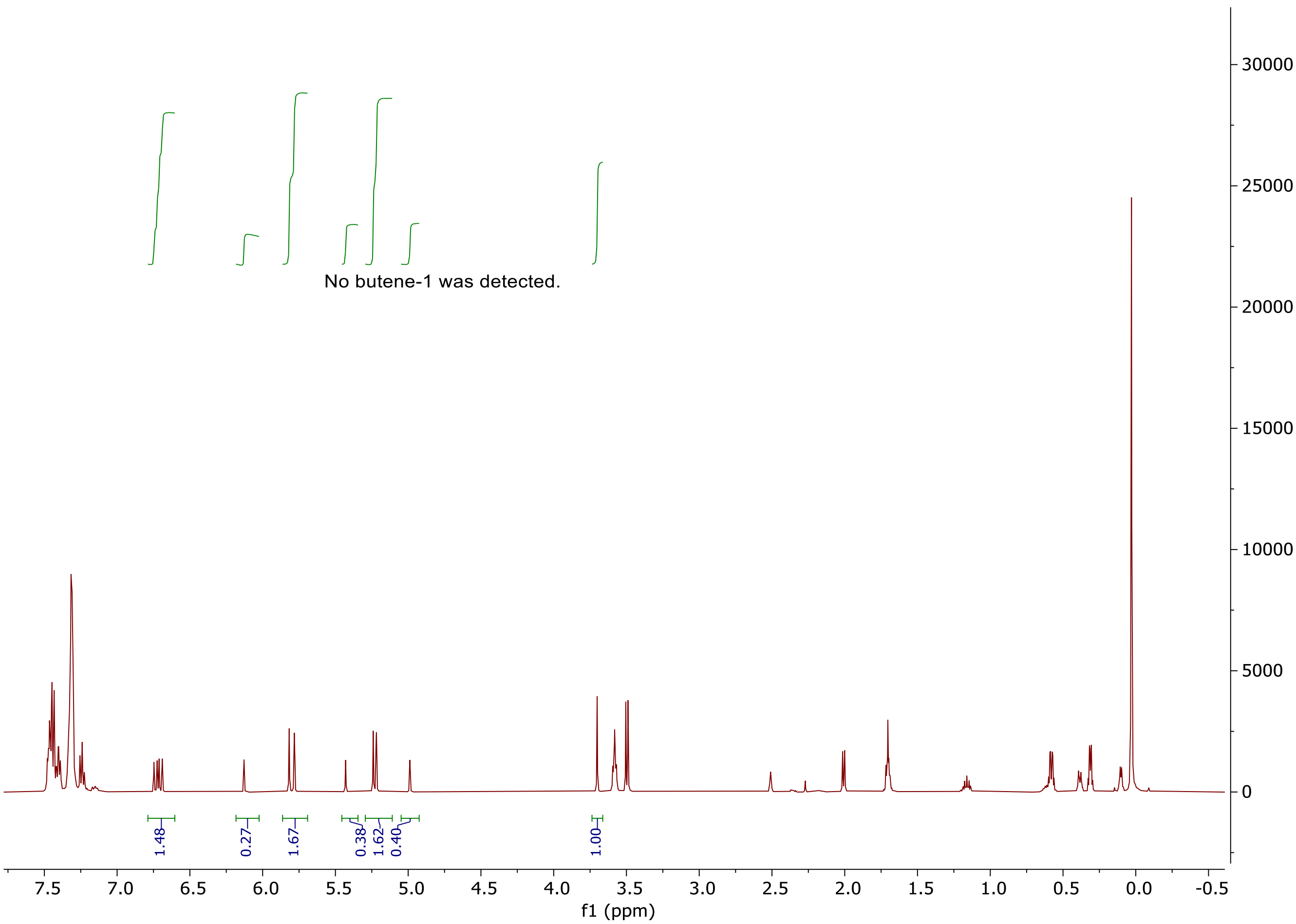

Figure S277: Radical trapping experiment using 2 equivalents of chloromethyl cyclopropane in the standard reaction conditions at ambient temperature – 0 h – ^{1}H NMR

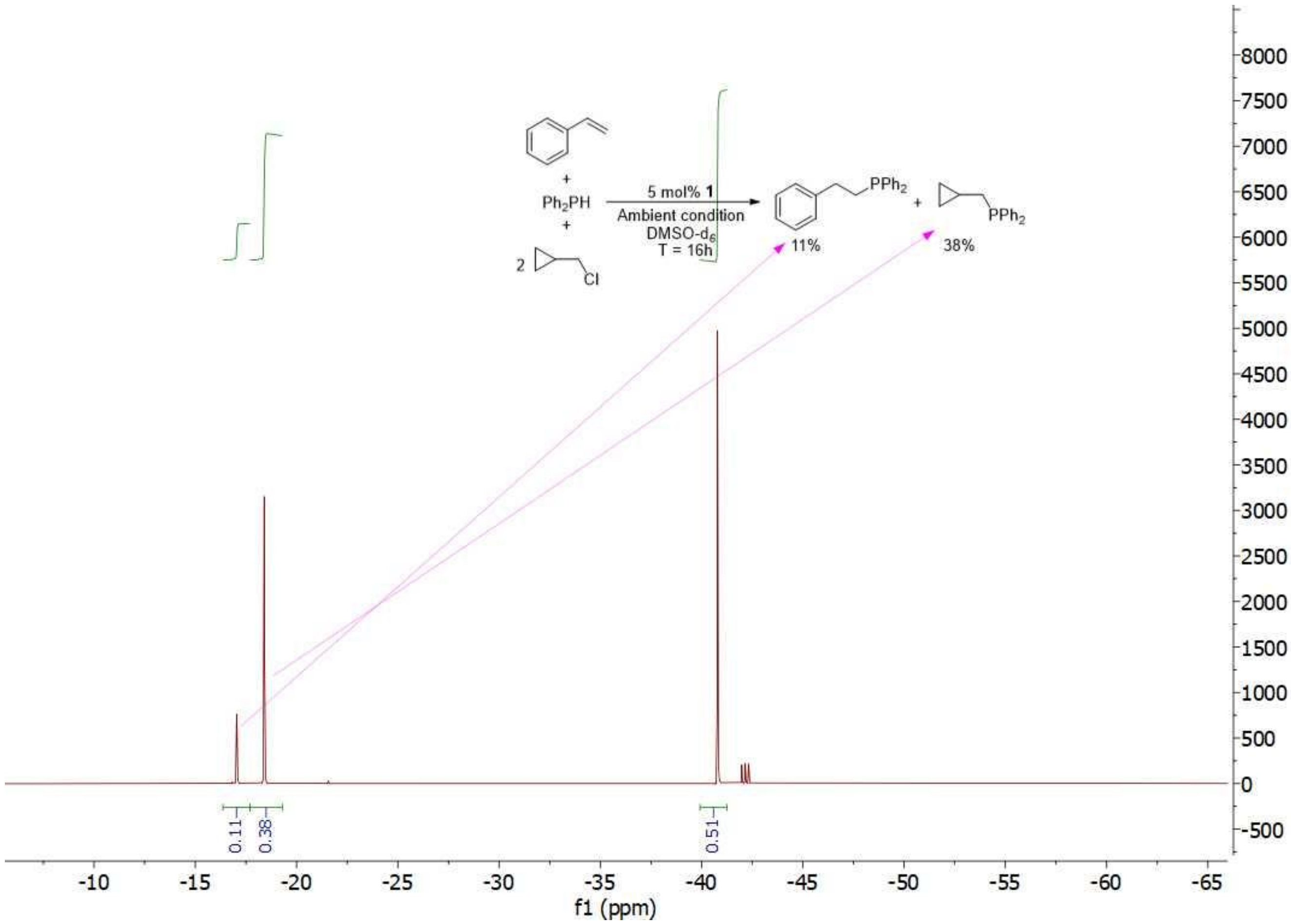

Figure S278: Radical trapping experiment using 2 equivalents of chloromethyl cyclopropane in the standard reaction conditions at ambient temperature – 16 h – $^{31}P\{^{1}H\}$ NMR

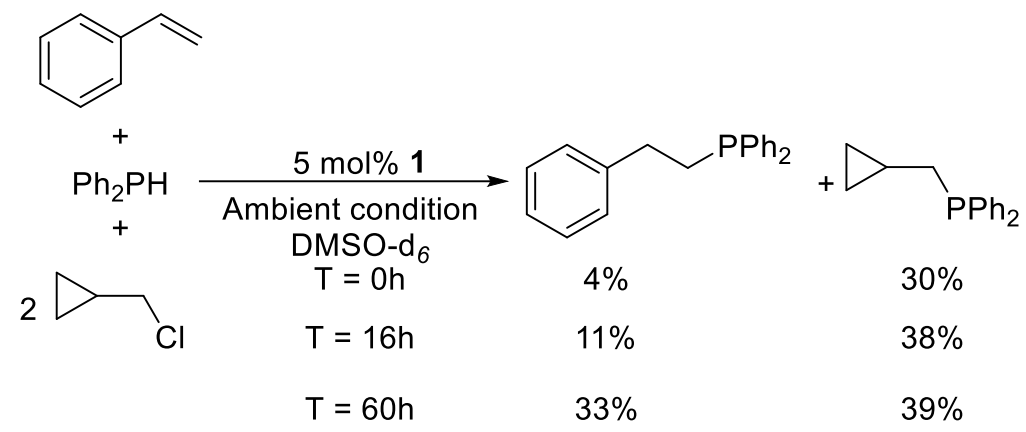

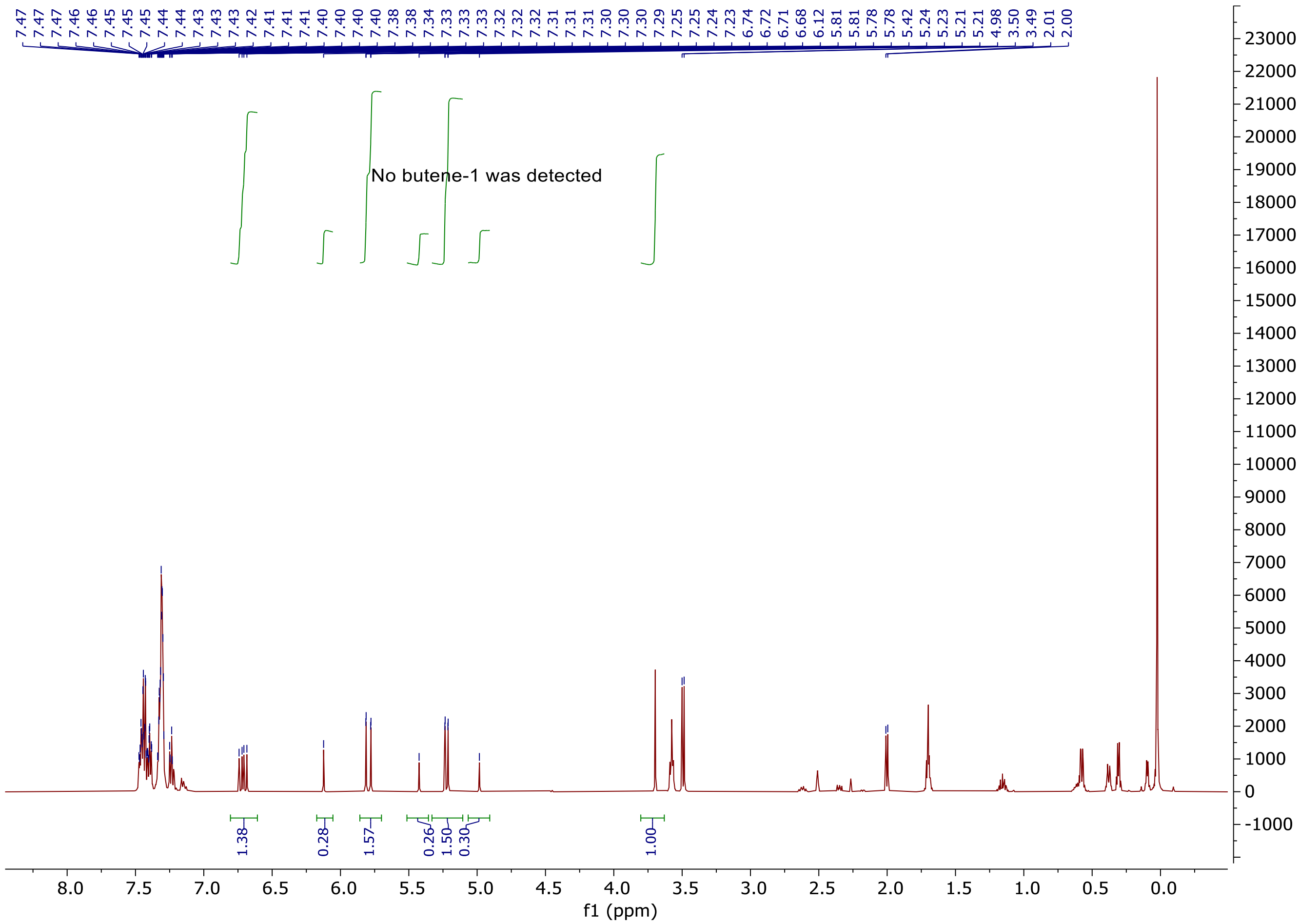

Figure S279: Radical trapping experiment using 2 equivalents of chloromethyl cyclopropane in the standard reaction conditions at ambient temperature – 16 h – ^{1}H NMR

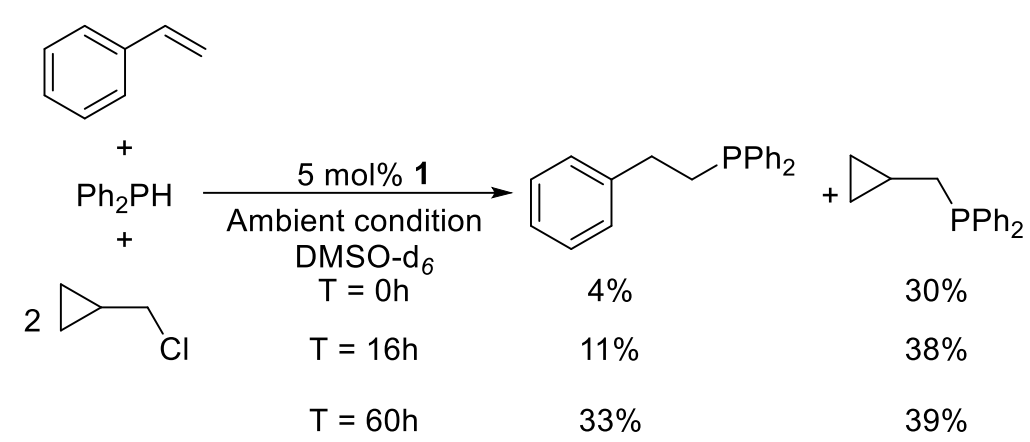

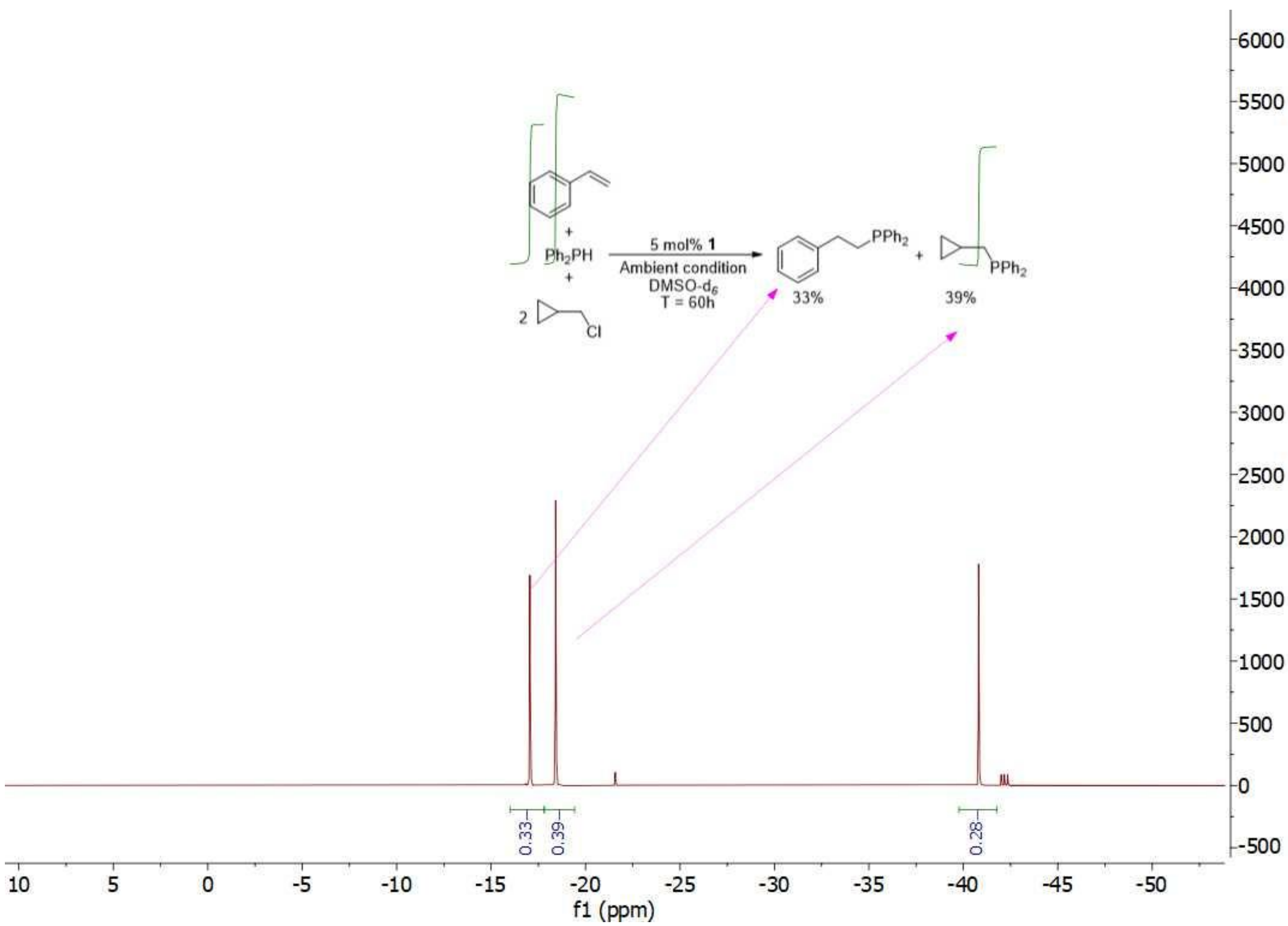

Figure S280: Radical trapping experiment using 2 equivalents of chloromethyl cyclopropane in the standard reaction conditions at ambient temperature – 60 h – $^{31}P\{^{1}H\}$ NMR

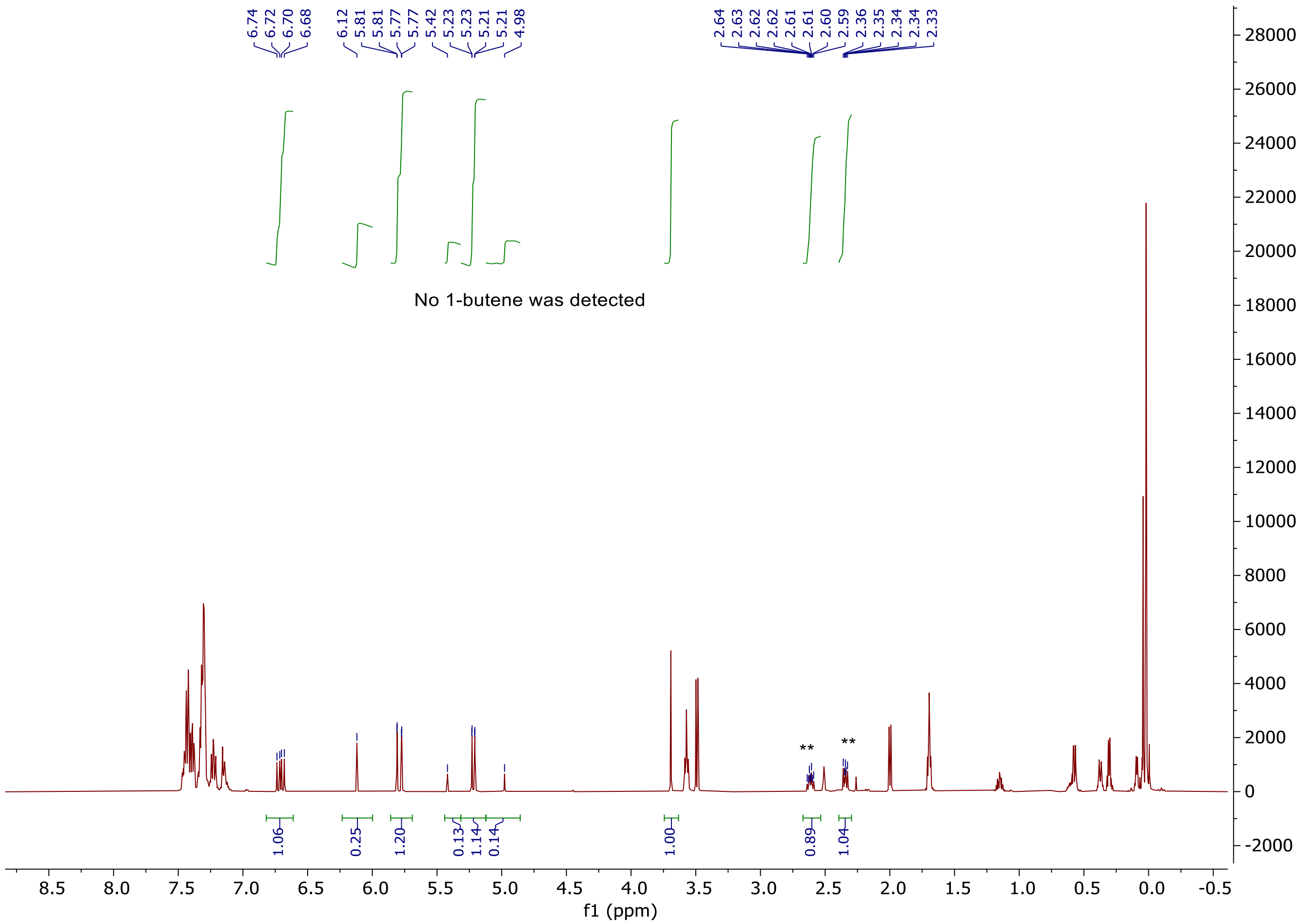

Figure S281: Radical trapping experiment using 2 equivalents of chloromethyl cyclopropane in the standard reaction conditions at ambient temperature – 60 h – ^{1}H NMR

NMR and HRMS spectra for radical trap reaction using α-cyclopropyl styrene

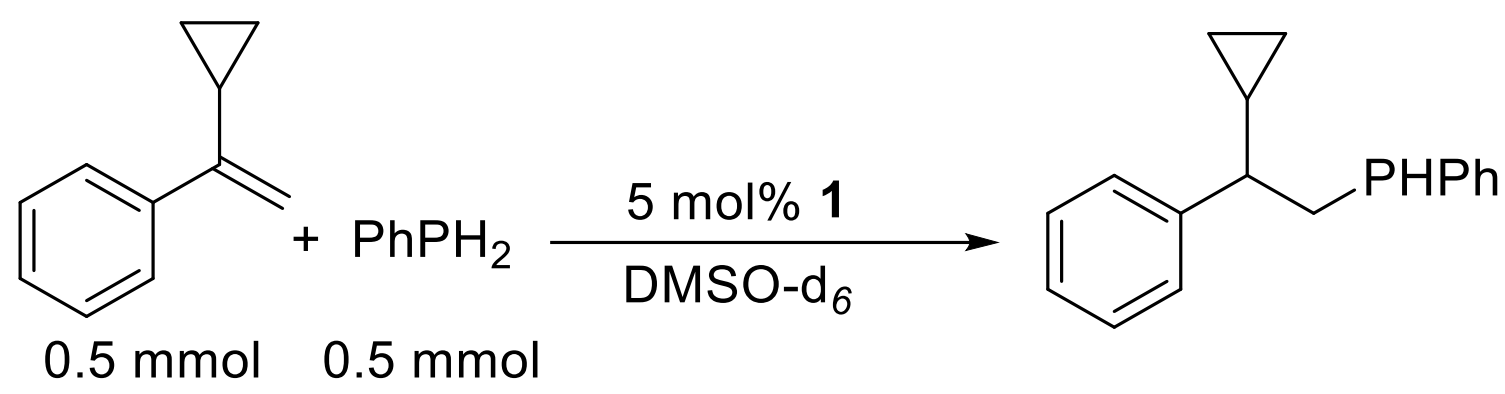

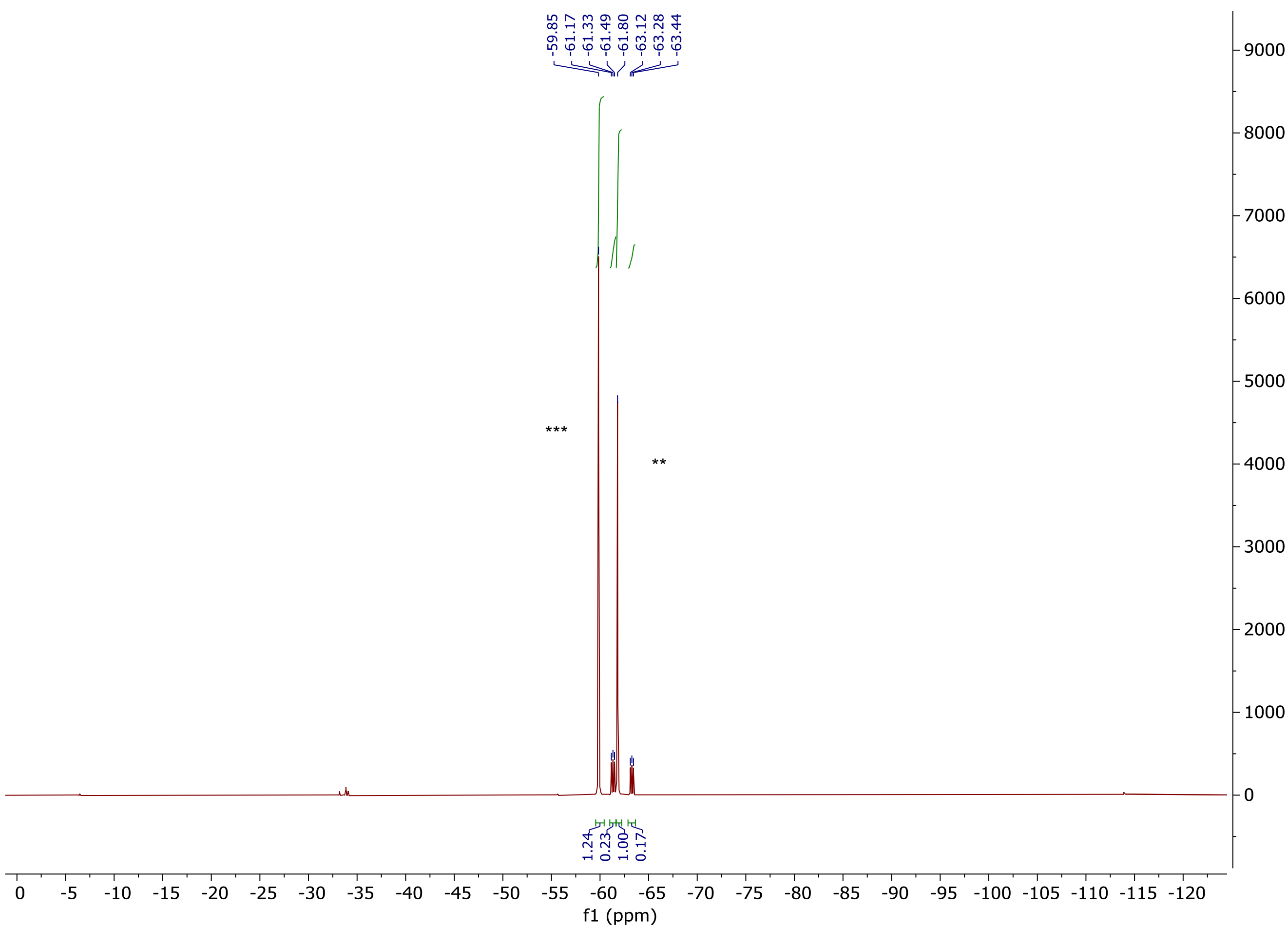

Figure S282: Radical clock experiment using 0.5 mmol of α-cyclopropyl styrene and equimolar phenyl phosphine in the standard reaction conditions at ambient temperature – 0 h – $^{31}P\{^{1}H\}$ NMR

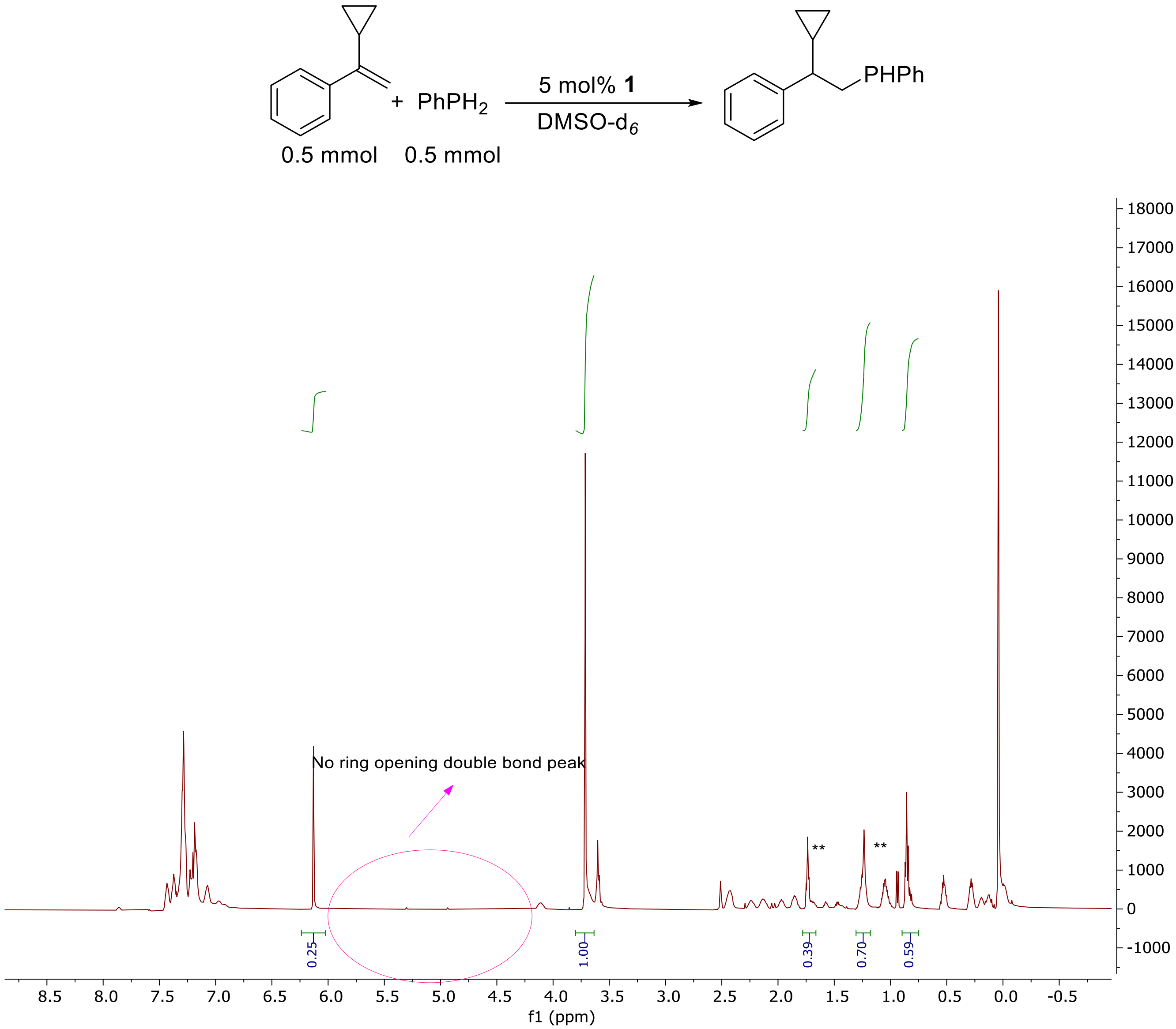

Figure S283: Radical clock experiment using 0.5 mmol of α-cyclopropyl styrene and equimolar phenyl phosphine in the standard reaction conditions at ambient temperature – 0 h – ^{1}H NMR

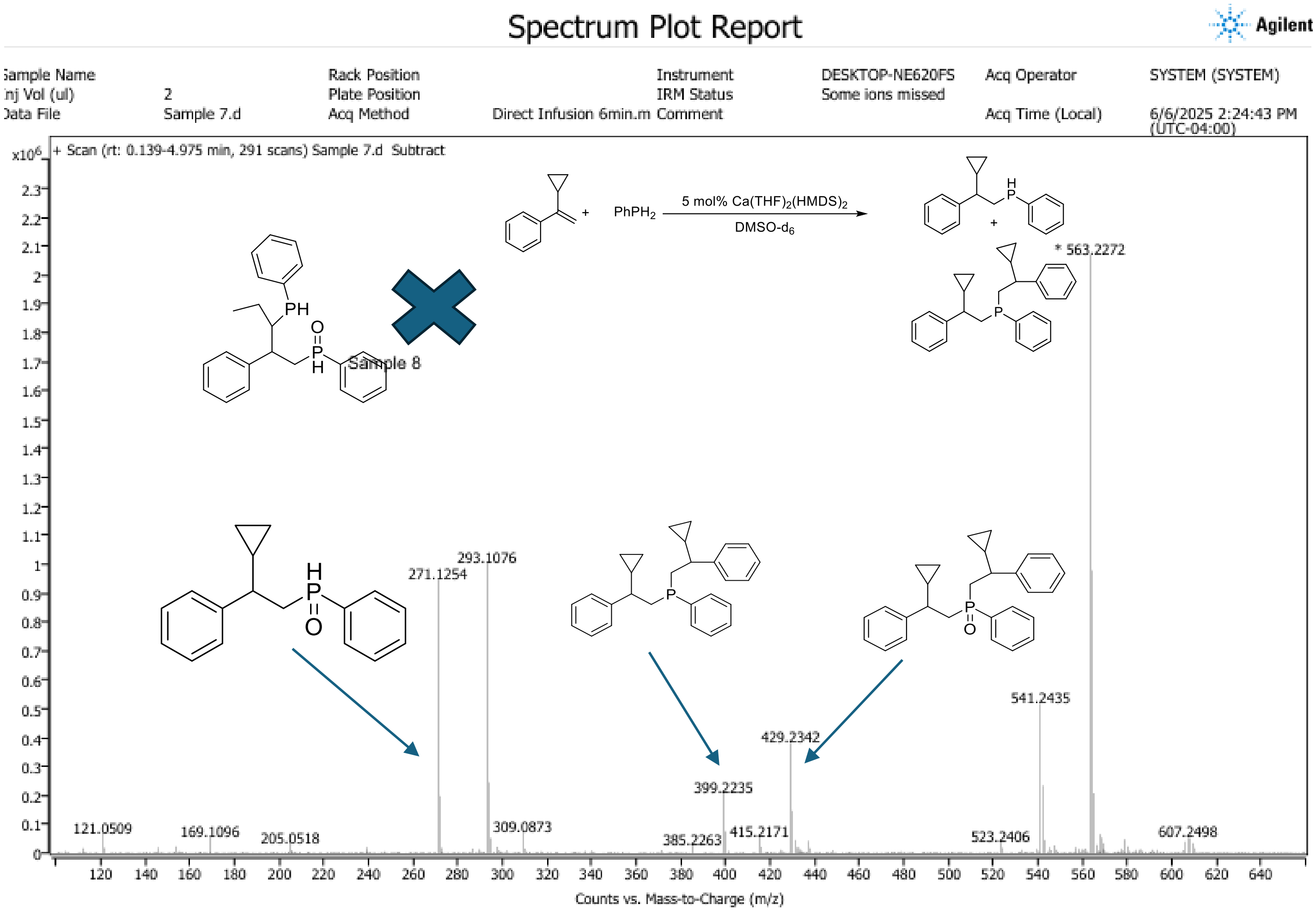

Figure S284: HRMS spectra for radical clock experiment using 0.5 mmol of α-cycloprop styrene and equimolar phenyl phosphine in the standard reaction conditions at ambient temperature – 0 h – ^{1}H NMR (No ring opening product was observed)

NMR spectra for radical trap experiment using BHT as radical scavenger

Standard BHT reaction condition involved equimolar amounts of styrene and PPh_2H, 5 mol% catalyst **1**, 2 equivalents of BHT in DMSO-d_6 at ambient temperature, with reaction progress monitored by $^{31}P\{^1H\}$ and ^1H NMR spectroscopy.

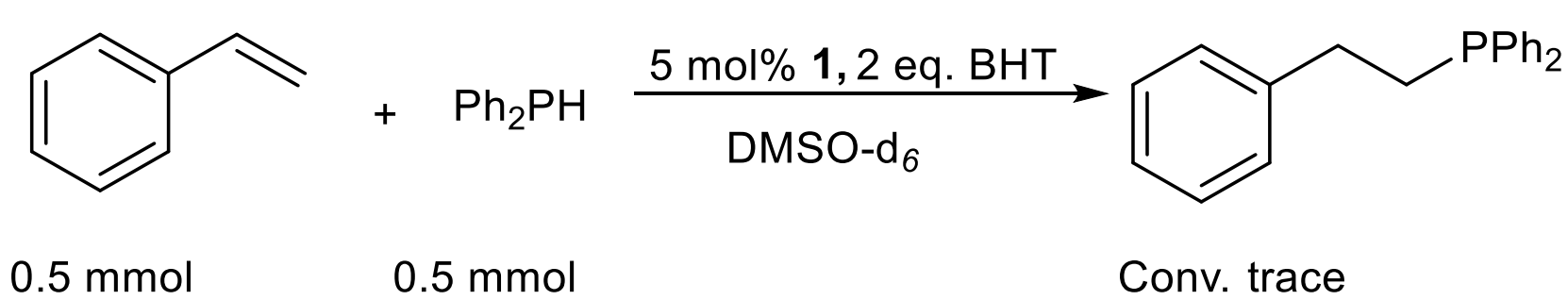

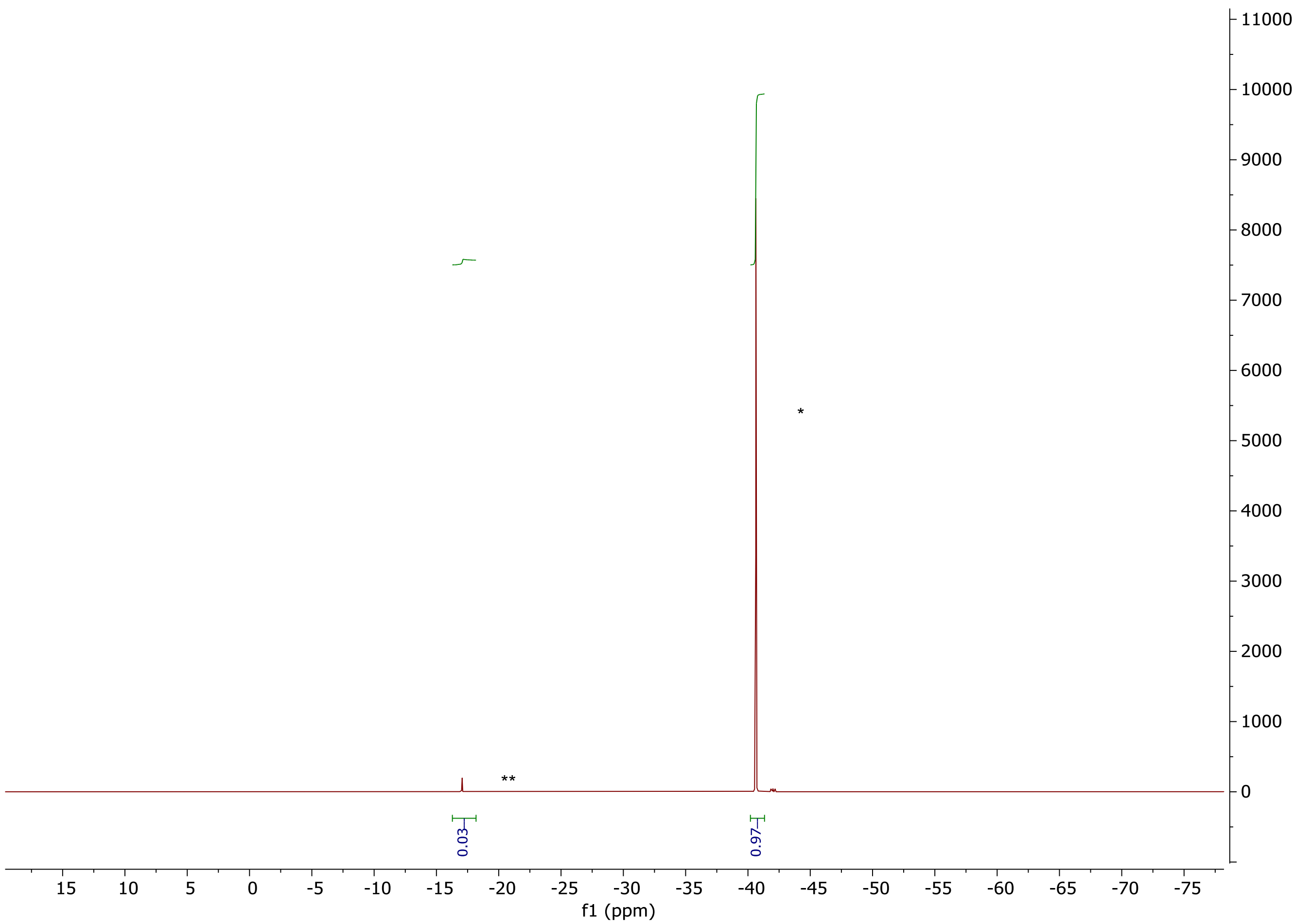

Figure S285: Radical trapping experiment using 2 equivalents of BHT in the standard reaction conditions at ambient temperature – 0 h – $^{31}P\{^1H\}$ NMR

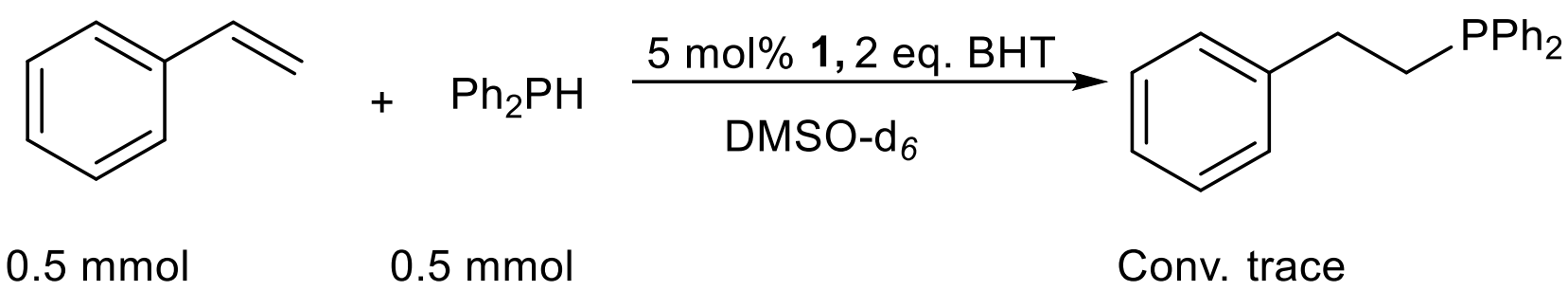

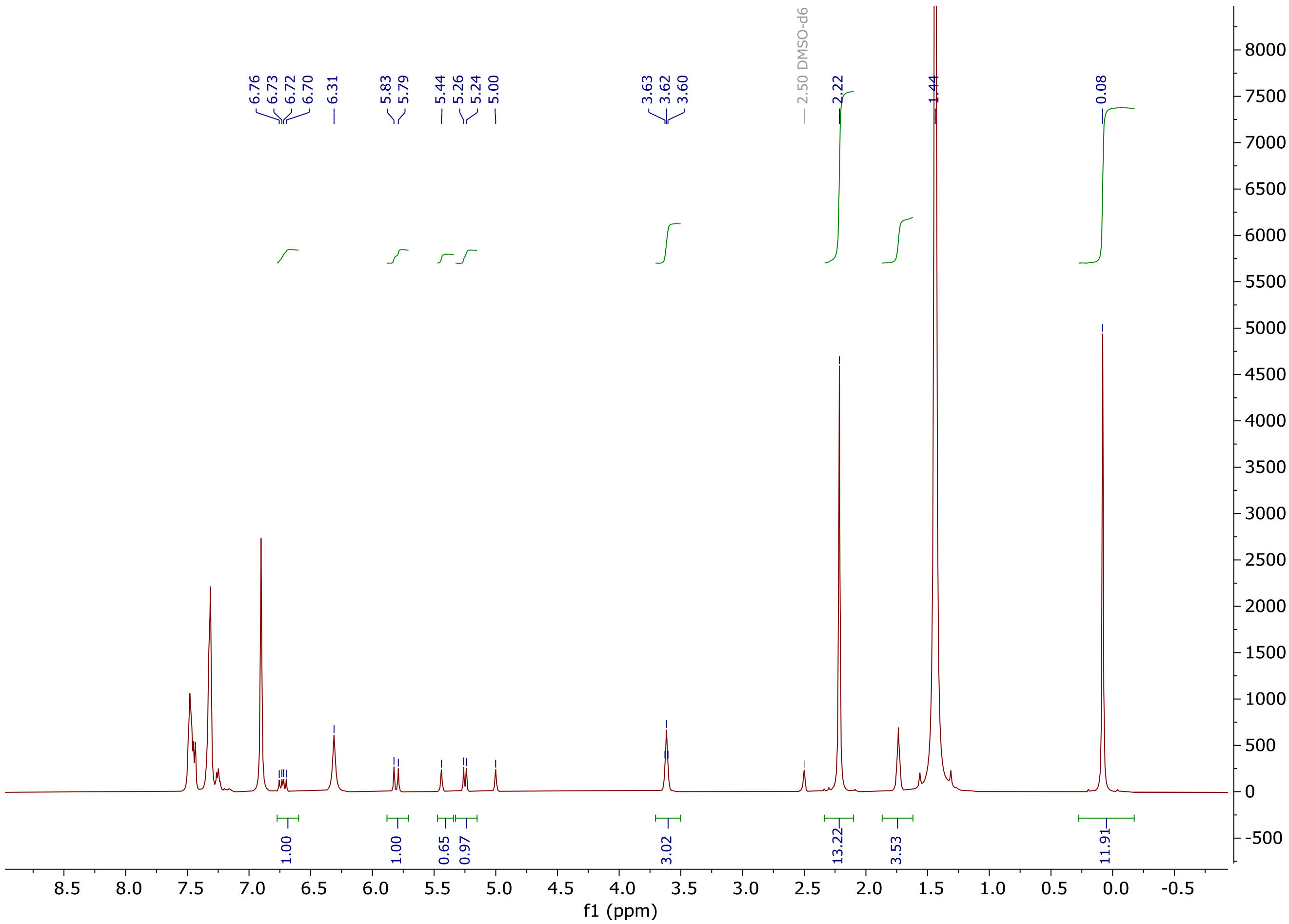

Figure S 286: Radical trapping experiment using 2 equivalents of BHT in the standard reaction conditions at ambient temperature – 0 h – {^{1}H} NMR

NMR spectra for radical trap experiment using hydroquinone as radical scavenger

Standard radical trap reaction using hydroquinone involved equimolar amounts of styrene and PPh_2H, 5 mol% catalyst **1**, 2 equivalents of hydroquione in DMSO-d_6 at ambient temperature, with reaction progress monitored by $^{31}P\{^1H\}$ and ^1H NMR spectroscopy.

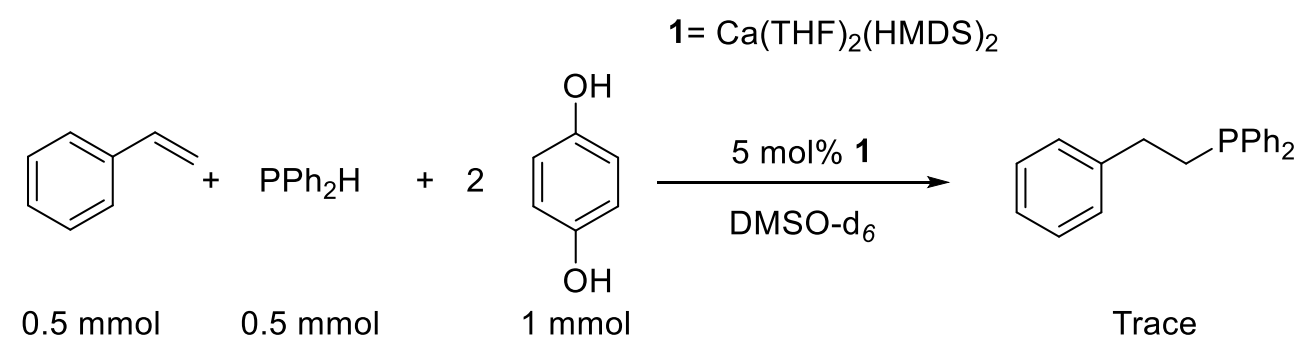

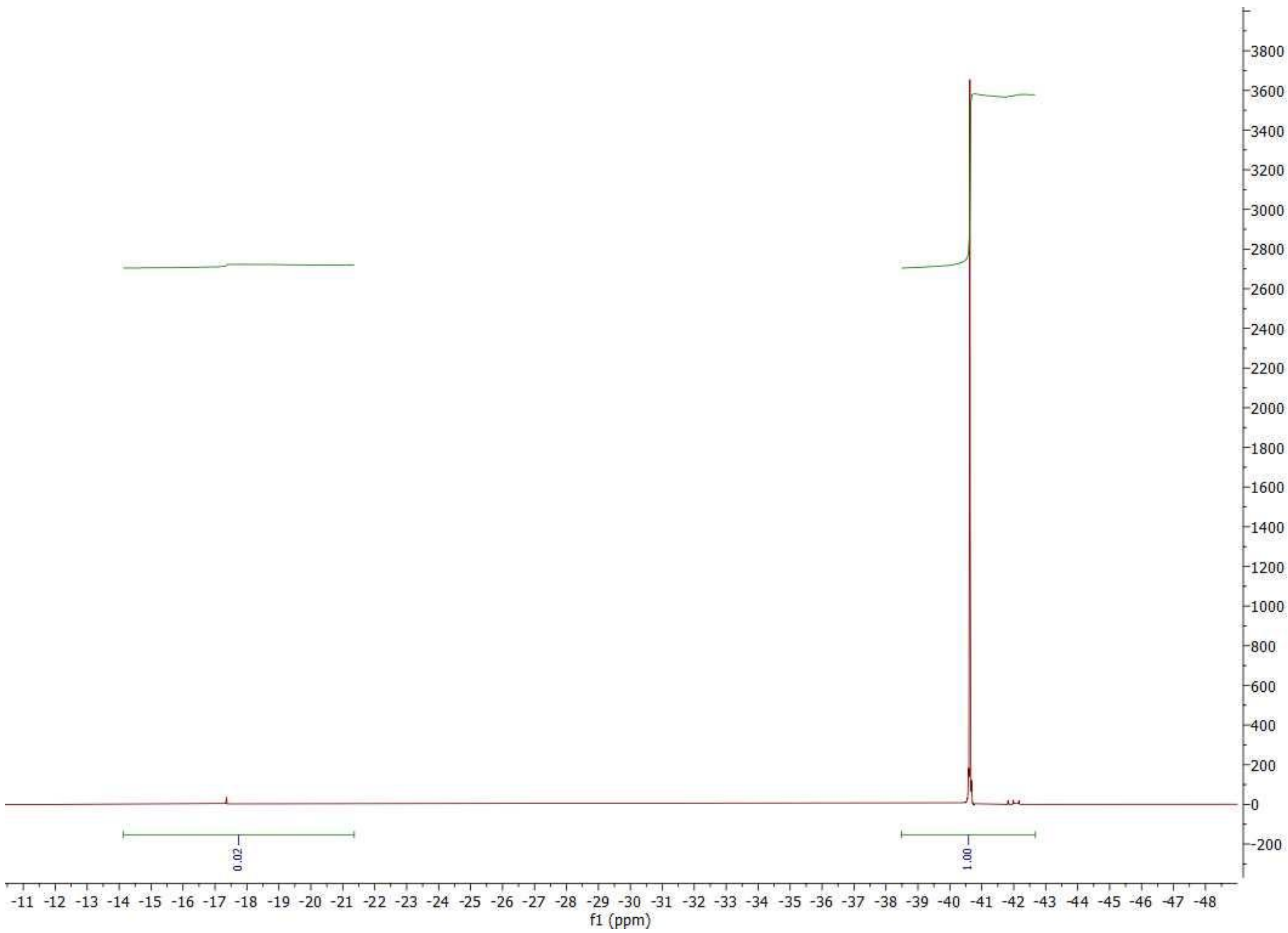

Figure S287: Radical trapping experiment using 2 equivalents of hydroquinone in the standard reaction conditions at ambient temperature – 0 h – $^{31}P\{^1H\}$ NMR

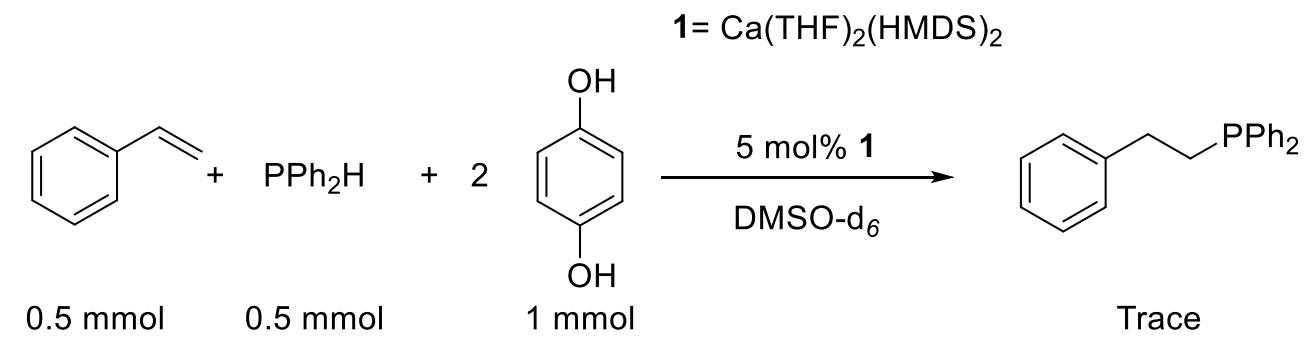

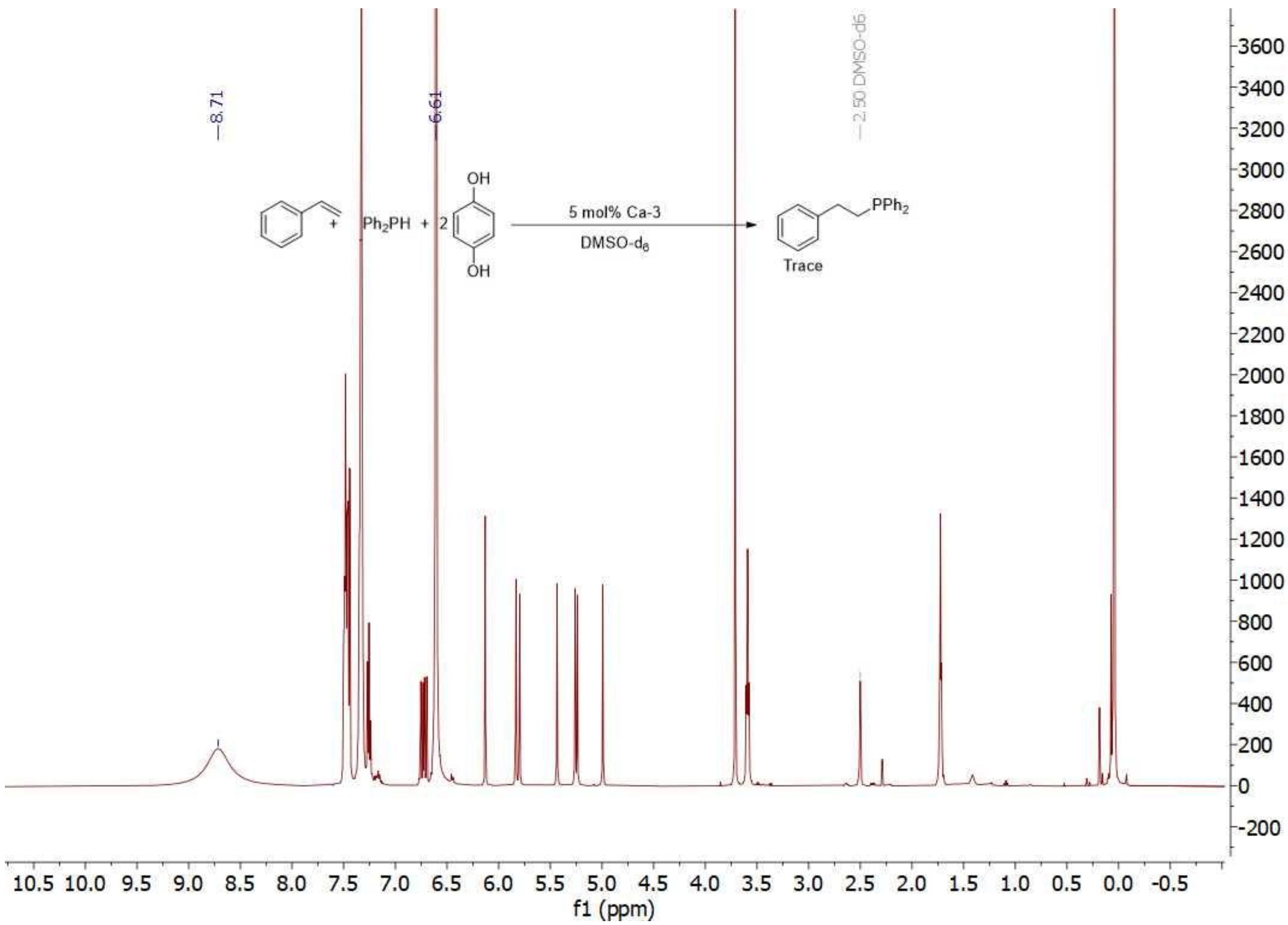

Figure S288: Radical trapping experiment using 2 equivalents of hydroquinone in the standard reaction conditions at ambient temperature – 0 h – ^{1}H NMR (-OH peak is at δ = 8.71 ppm)

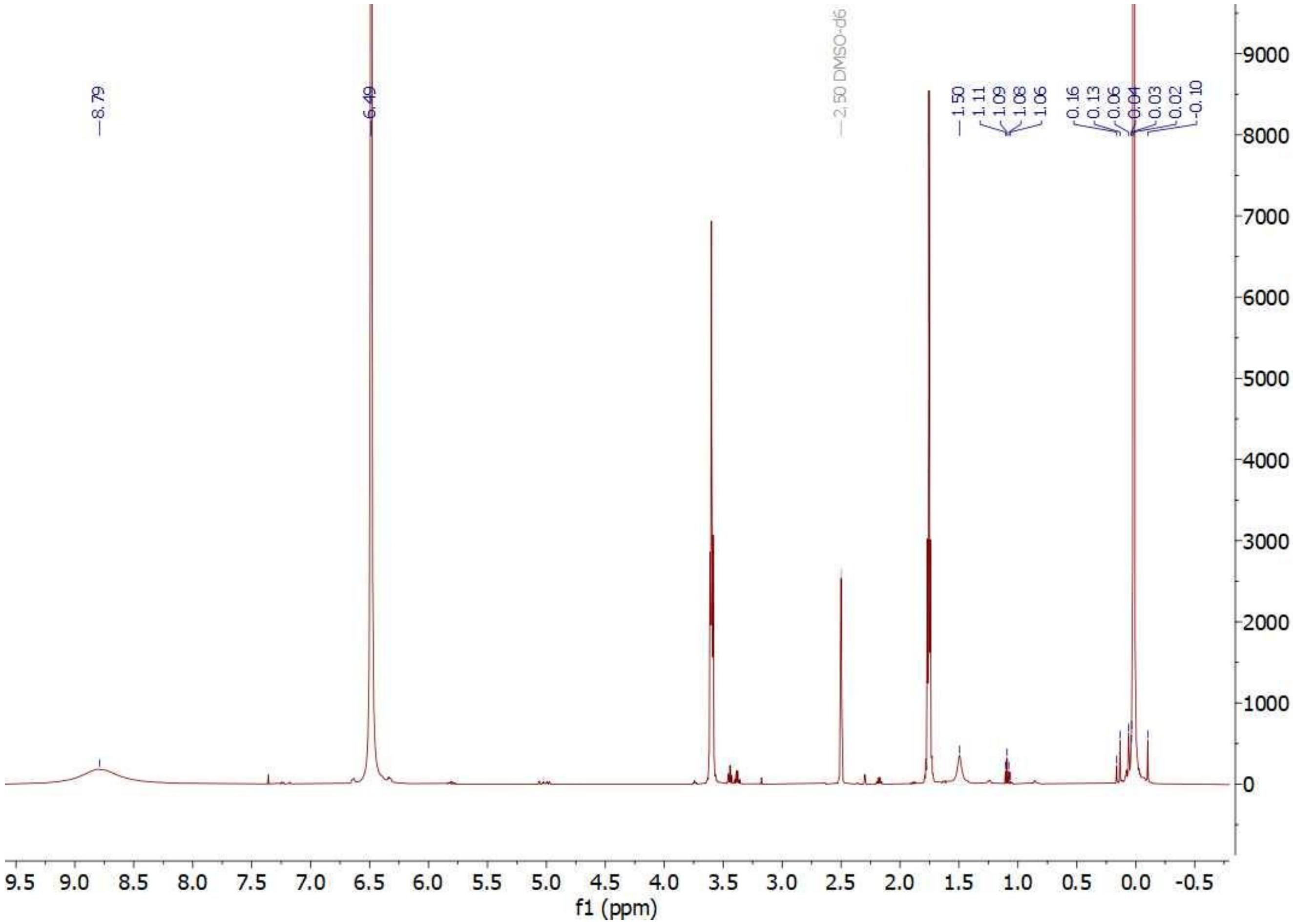

Figure S289: Stoichiometric reaction between 0.5 mmol hydroquinone and 0.5 mmol **1** – ^{1}H NMR (-OH peak in different chemical shift, changes its position indicating coordination with catalyst) (-OH peak is at δ = 8.89 ppm)

NMR spectra for radical trap experiment using 1,4-cyclohexadiene as radical scavenger

Standard radical trap reaction using 1,4-cyclohexadiene involved equimolar amounts of styrene and PPh_2H, 5 mol% catalyst **1**, 5 equivalents of 1,4-cyclohexadiene in DMSO-d_6 at ambient temperature, with reaction progress monitored by $^{31}P\{^1H\}$ and ^1H NMR spectroscopy.

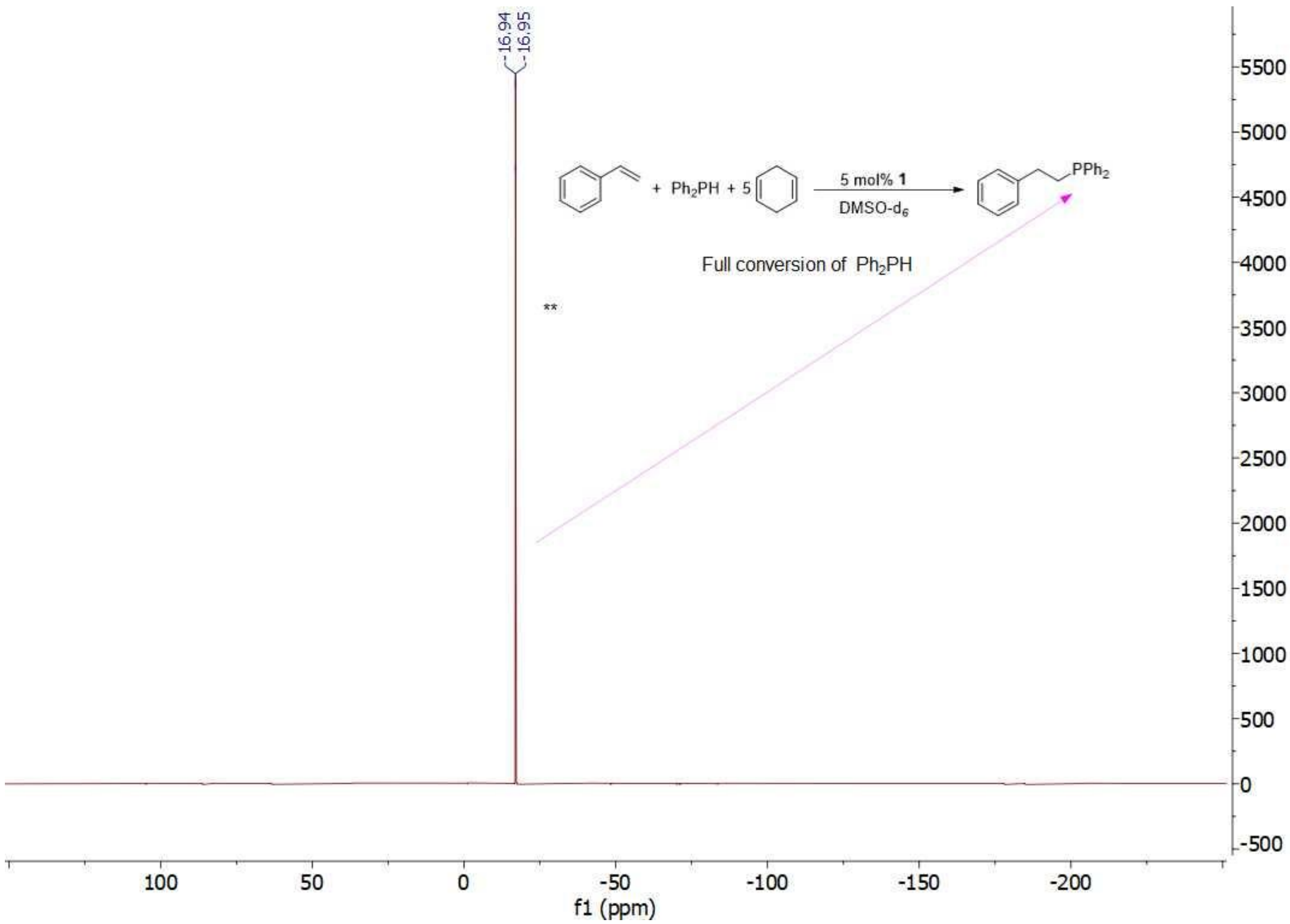

Figure S290: Radical trapping experiment using 5 equivalents of 1,4-cyclohexadiene in the standard reaction conditions at ambient temperature – 0 h – $^{31}P\{^1H\}$ NMR

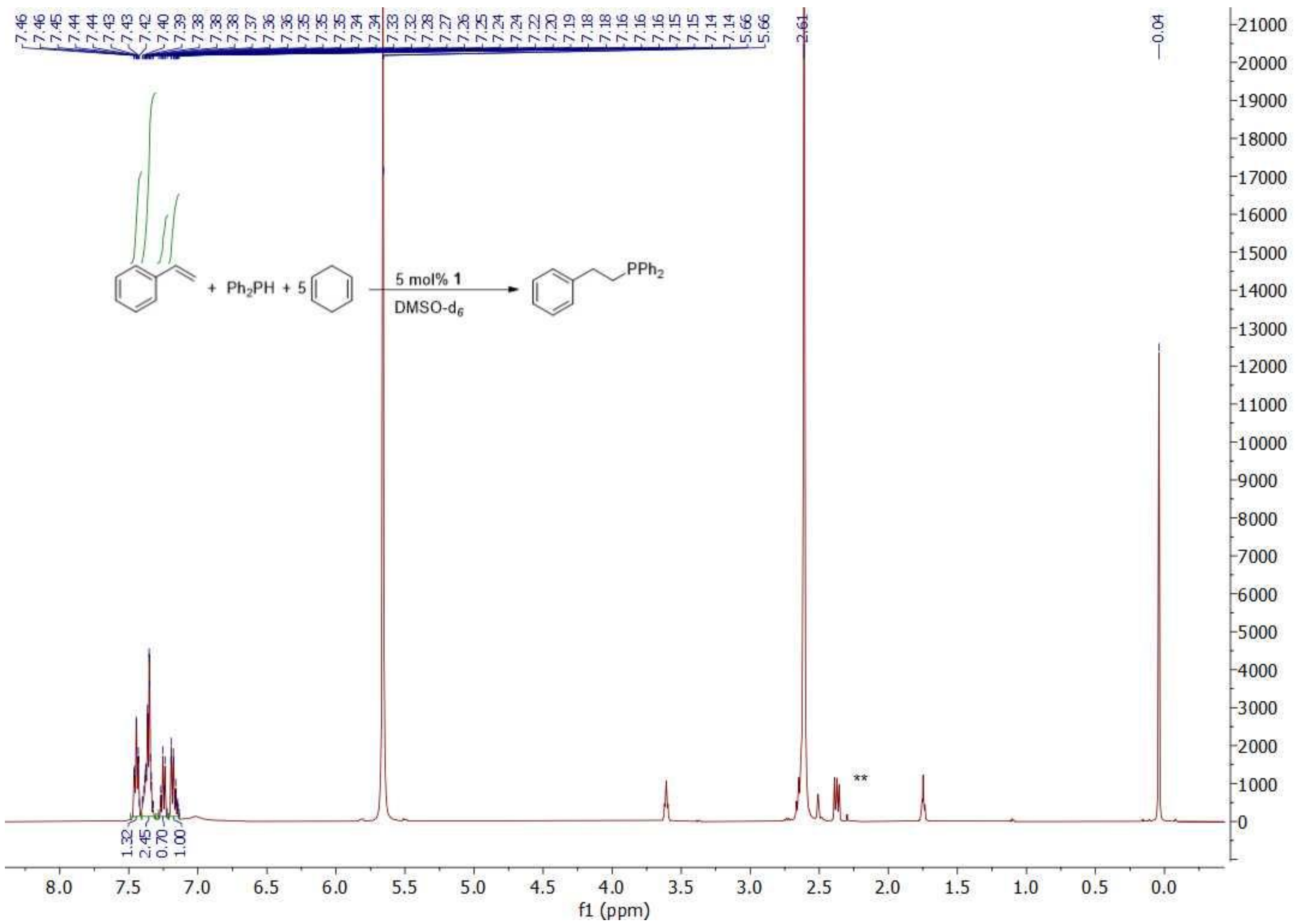

Figure S291: Radical trapping experiment using 5 equivalents of 1,4-cyclohexadiene in the standard reaction conditions at ambient temperature – 0 h – ^{1}H NMR. (No new phenyl peak group was observed in ^{1}H NMR)

NMR spectra for radical trap experiment using diphenyl amine as radical scavenger

Standard radical trap reaction using diphenylamine involved equimolar amounts of styrene and PPh_2H, 5 mol% catalyst **1**, 2 equivalents of diphenylamine in DMSO-d_6 at ambient temperature, with reaction progress monitored by $^{31}P\{^1H\}$ and ^1H NMR spectroscopy.

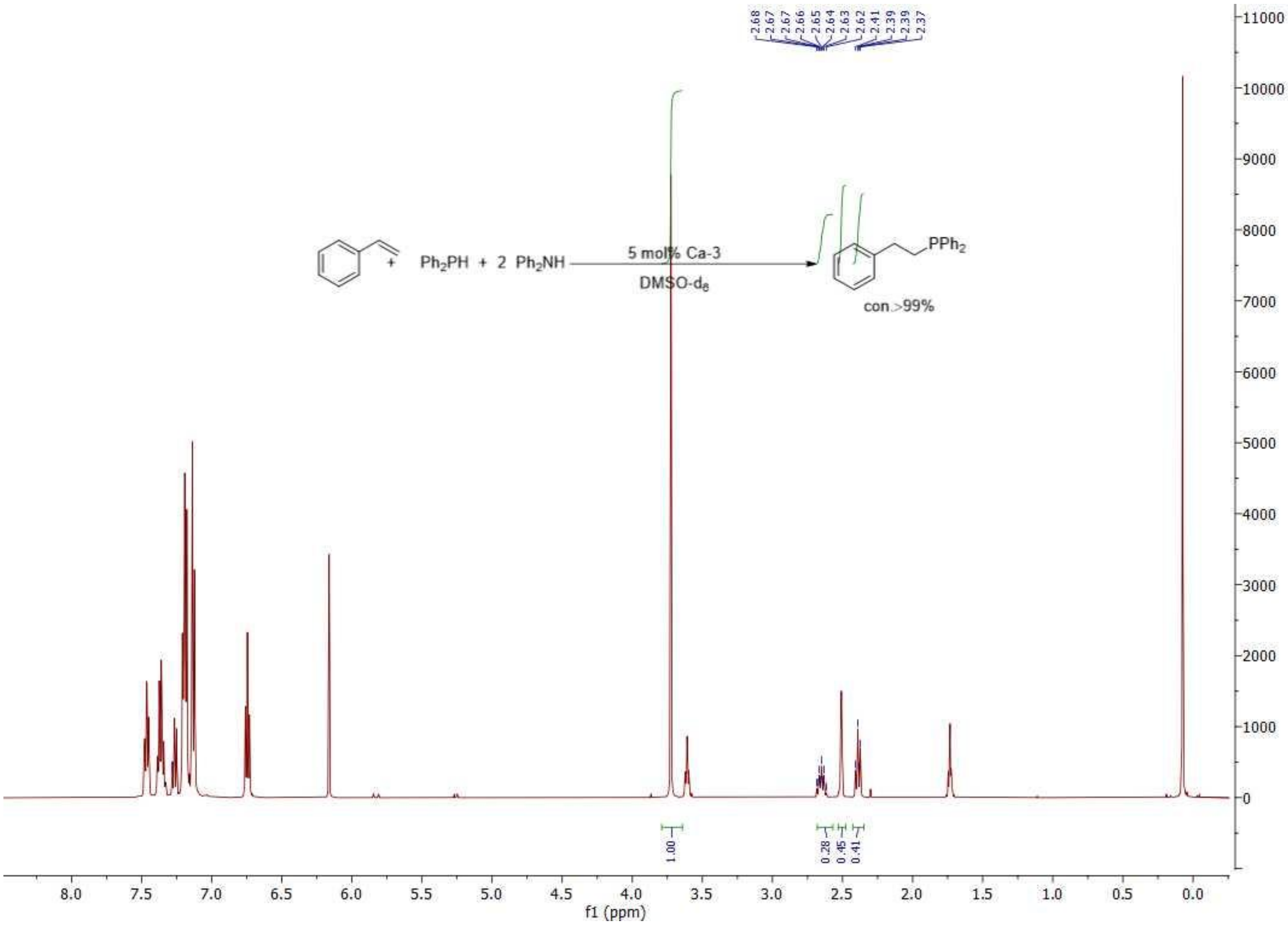

Figure S292: Radical trapping experiment using 2 equivalents of diphenylamine in the standard reaction conditions at ambient temperature – 0 h – ^1H NMR

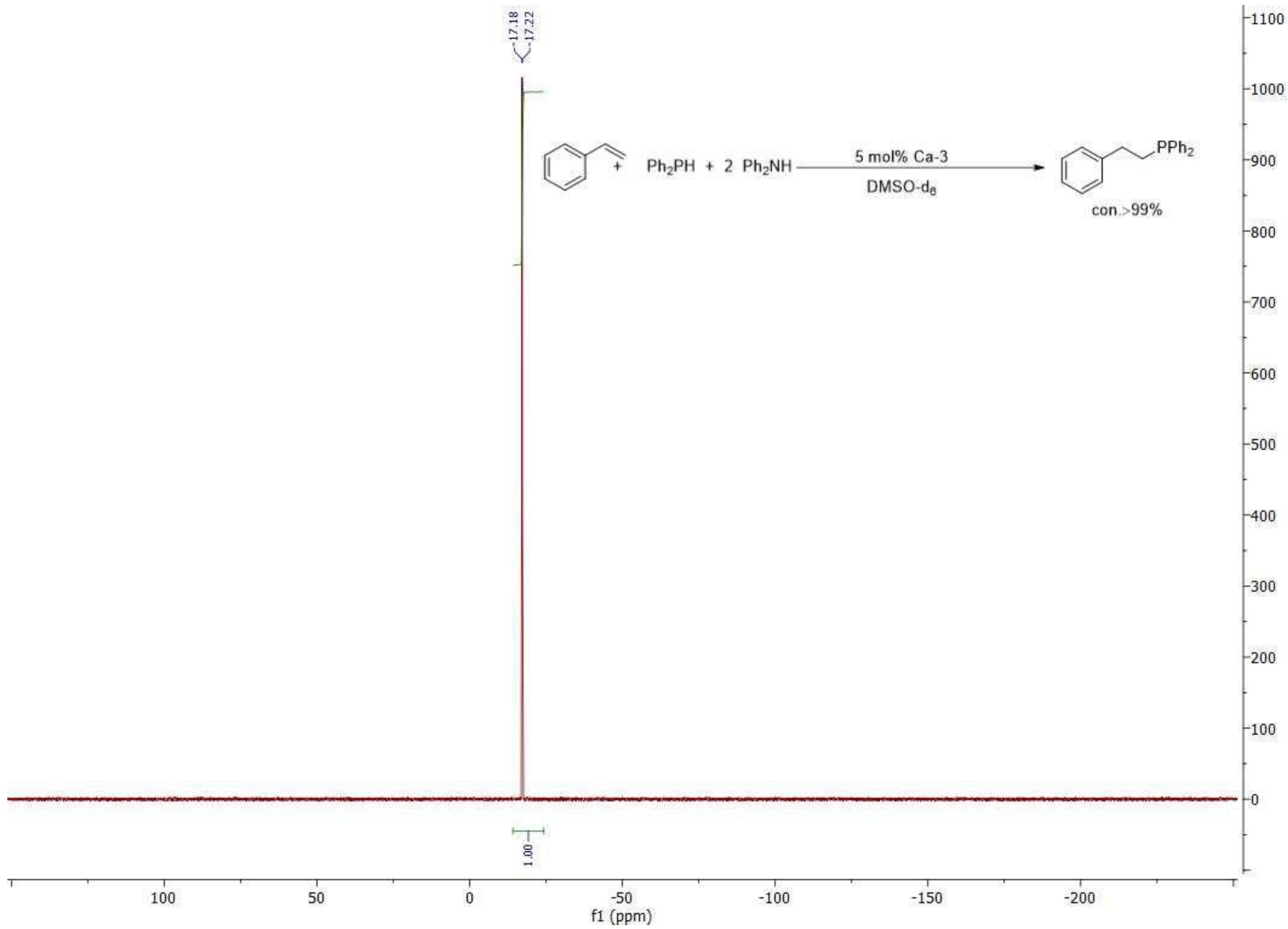

Figure S293: Radical trapping experiment using 2 equivalents of diphenylamine in the standard reaction conditions at ambient temperature – 0 h – $^{31}P\{^1H\}$ NMR

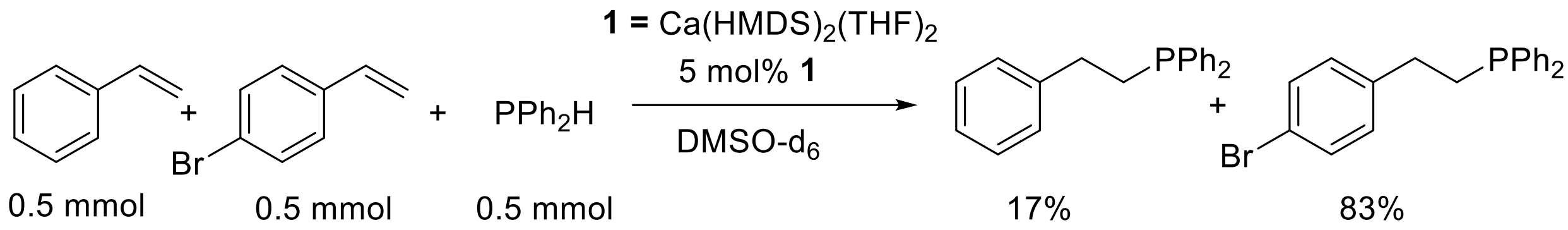

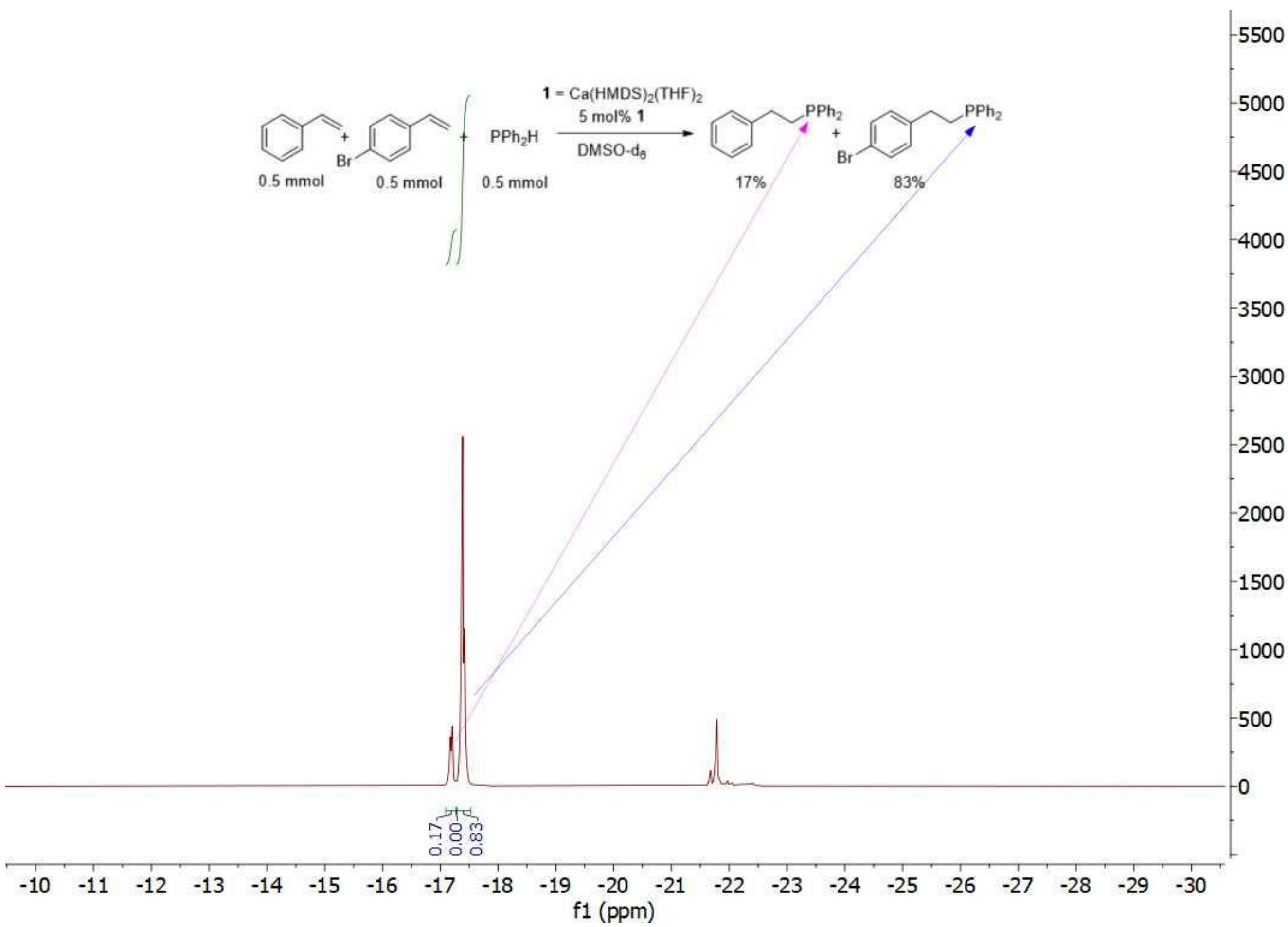

Figure S294: Hammett plot - $^{31}P\{^1H\}$ NMR spectrum [Reaction of 0.5 mmol 4-bromostyrene, 0.5 mmol styrene, 0.5 mmol Ph_2PH presence of 5 mol% **1** in DMSO-d_6]

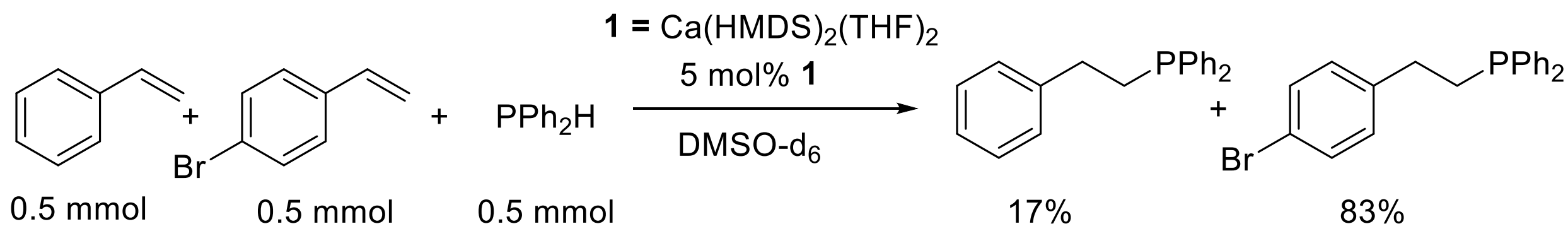

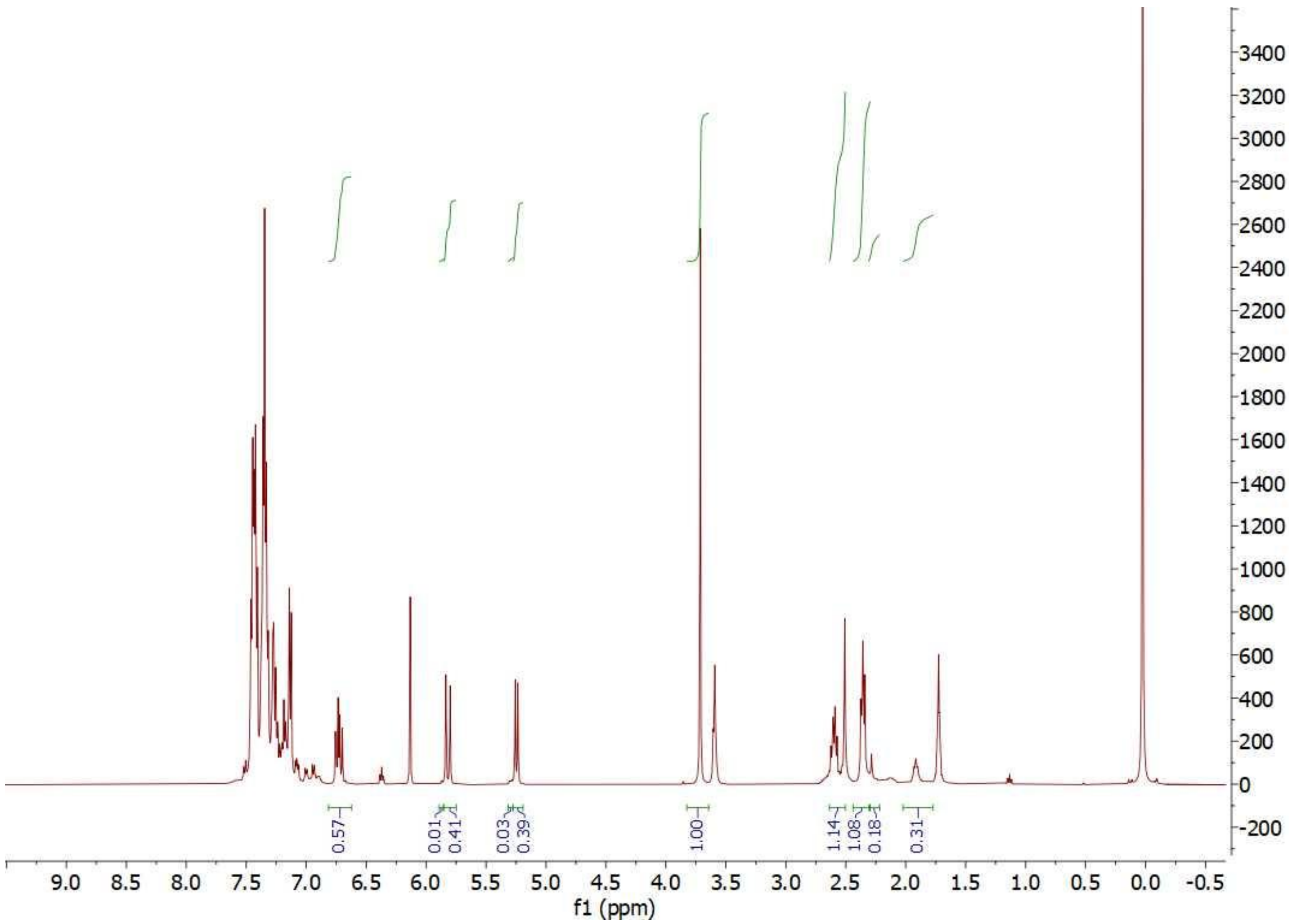

Figure S295: Hammett plot ^{1}H NMR spectrum [Reaction of 0.5 mmol 4-bromostyrene, 0.5 mmol styrene, 0.5 mmol Ph_2PH in presence of 5 mol% **1** in DMSO-d_6].

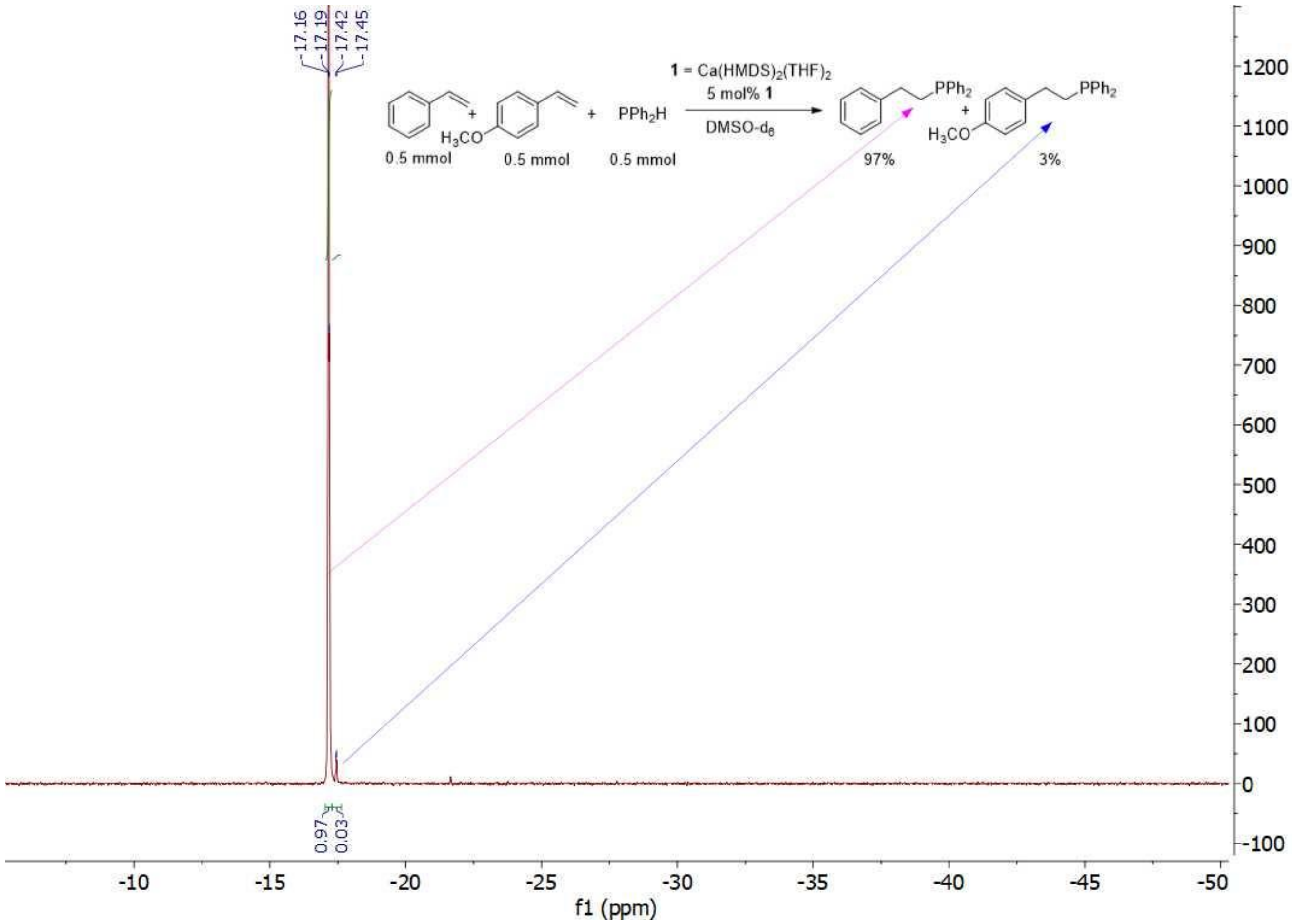

Figure S296: Hammett plot - $^{31}P\{^1H\}$ NMR spectrum [Reaction of 0.5 mmol 4-methoxystyrene, 0.5 mmol styrene, 0.5 mmol Ph_2PH in presence of 5 mol% **1** in DMSO-d_6.]

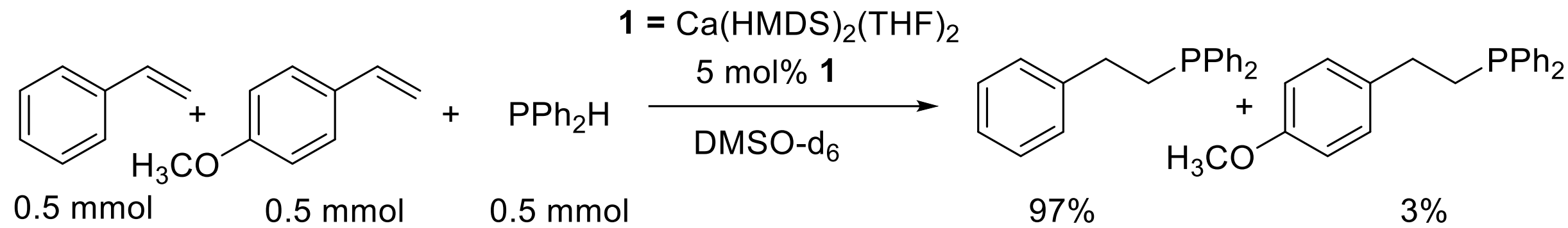

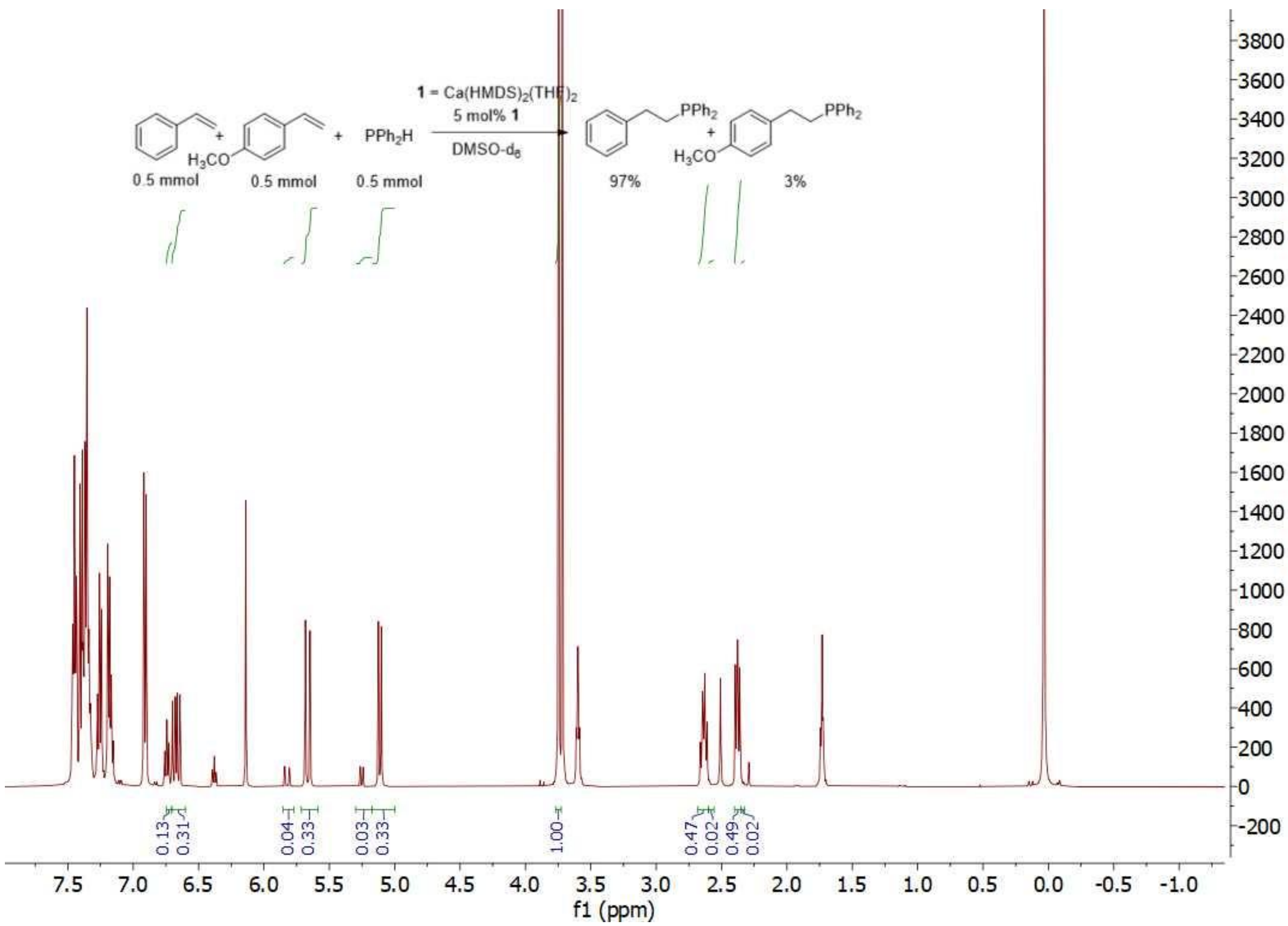

Figure S297: Hammett plot ^{1}H NMR spectrum [Reaction of 4-methoxystyrene, styrene, Ph_2PH in presence of 5 mol% **1** in DMSO]

Hammett Plot using 5 mol% **1** as catalyst at ambient condition

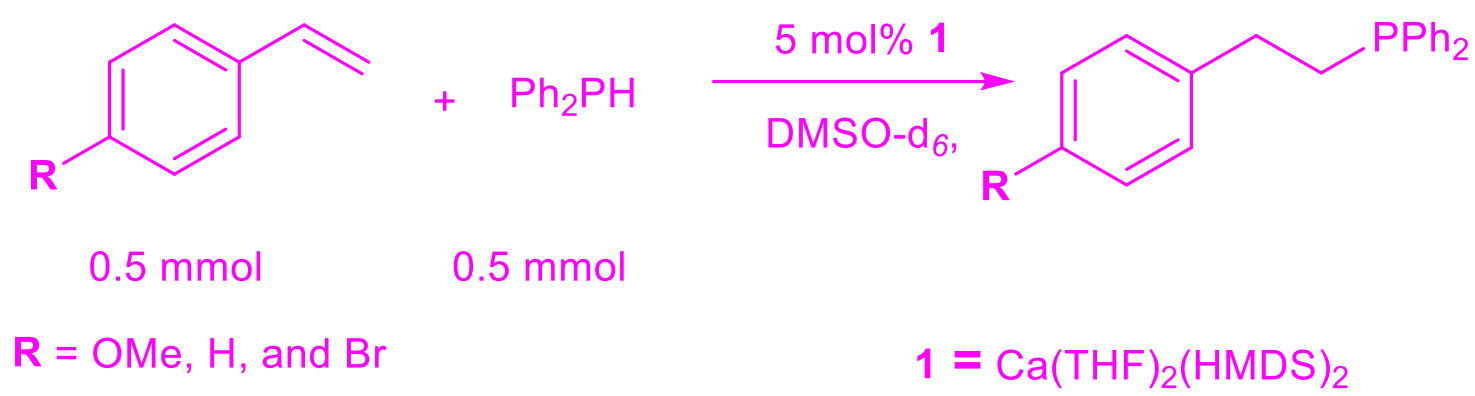

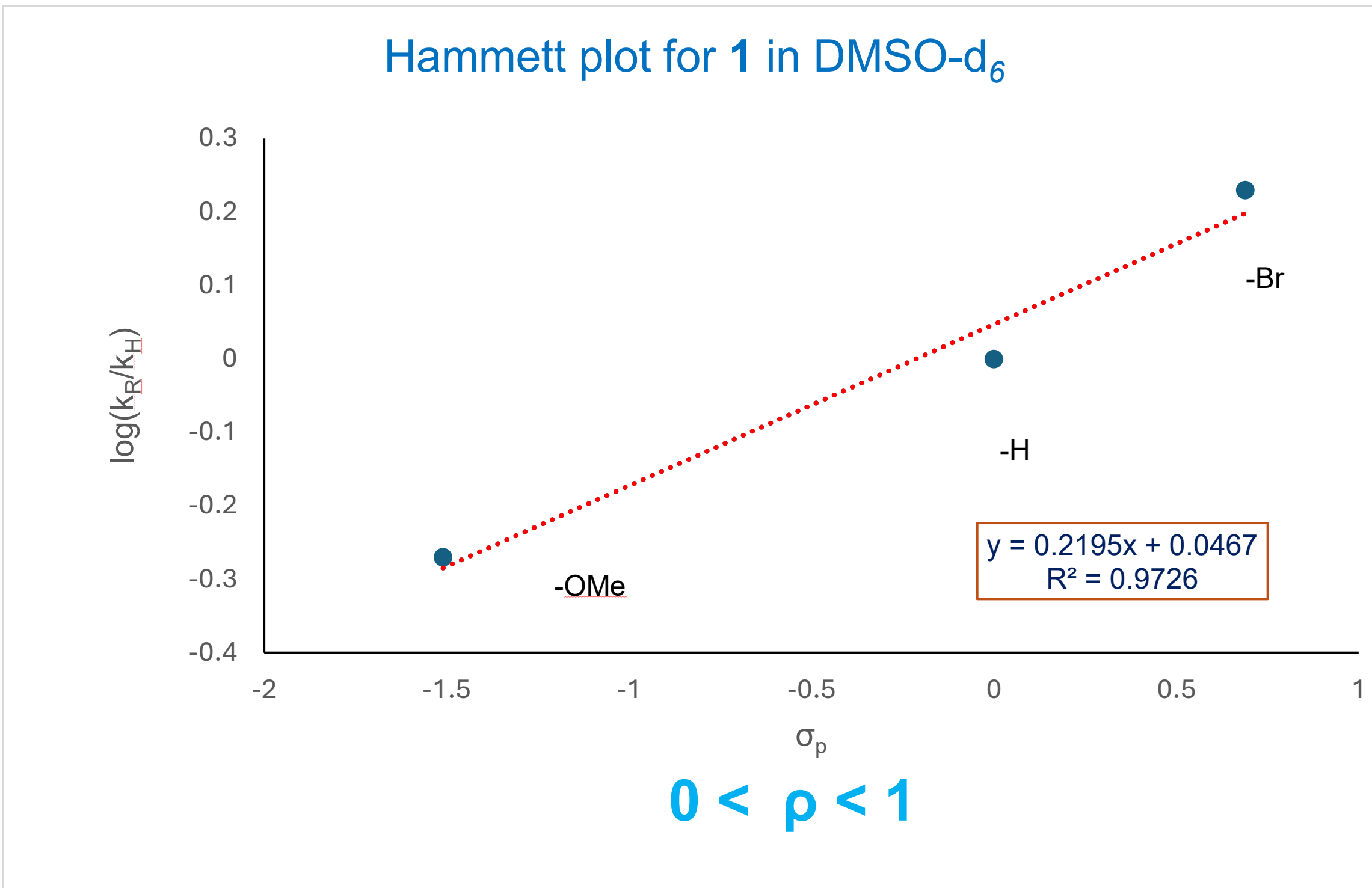

Figure S298: Hammett study of para-substituted styrene derivatives, styrene, and $HPPh_2$ using 5 mol% **1** as catalyst.

NMR spectra for deuterium labelling reactions

Deuterium labeling experiments were performed in a J-Young tube by charging 0.5 mmol styrene, 1 equivalent of diphenylphosphine-d_1 (80% deuteration) and 5 mol% of **1** and the reaction was monitored following ^1H, ^2H, $^{31}P\{^1H\}$, $^{31}P\{^1H\}$-^1H HMBC NMR spectroscopy. The ^1H NMR spectroscopy reveals that multiplet at 2.35 ppm contains proton, from our previous report it was confirmed that multiplet at 2.35 ppm is for protons bonded with carbon, α with respect to phenyl ring, and multiplet at 2.6 ppm is for protons bonded with carbon β with respect to phenyl ring, analysis was done by ^{31}P-^1H HMBC, 13C-^1H HMBC spectroscopy. From 2H NMR, deuteriation is only at β position with respect to phenyl ring.

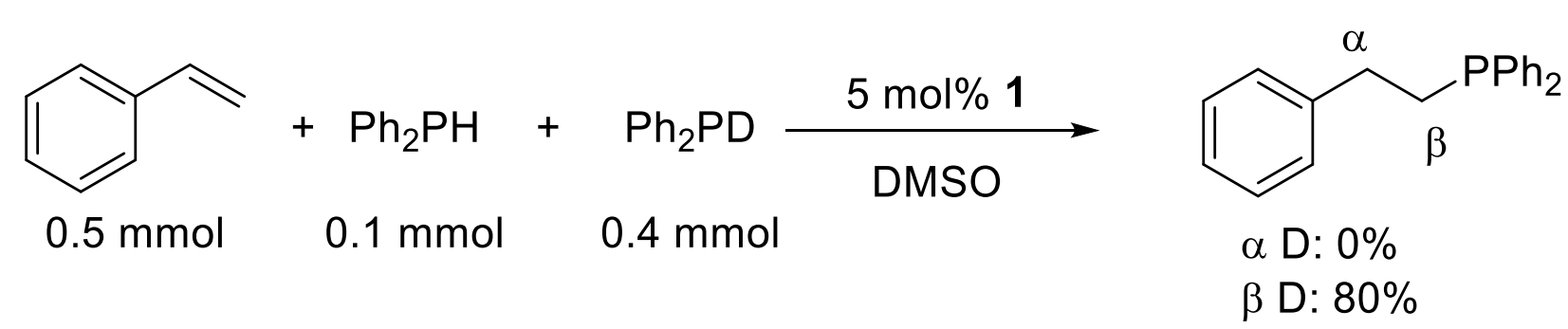

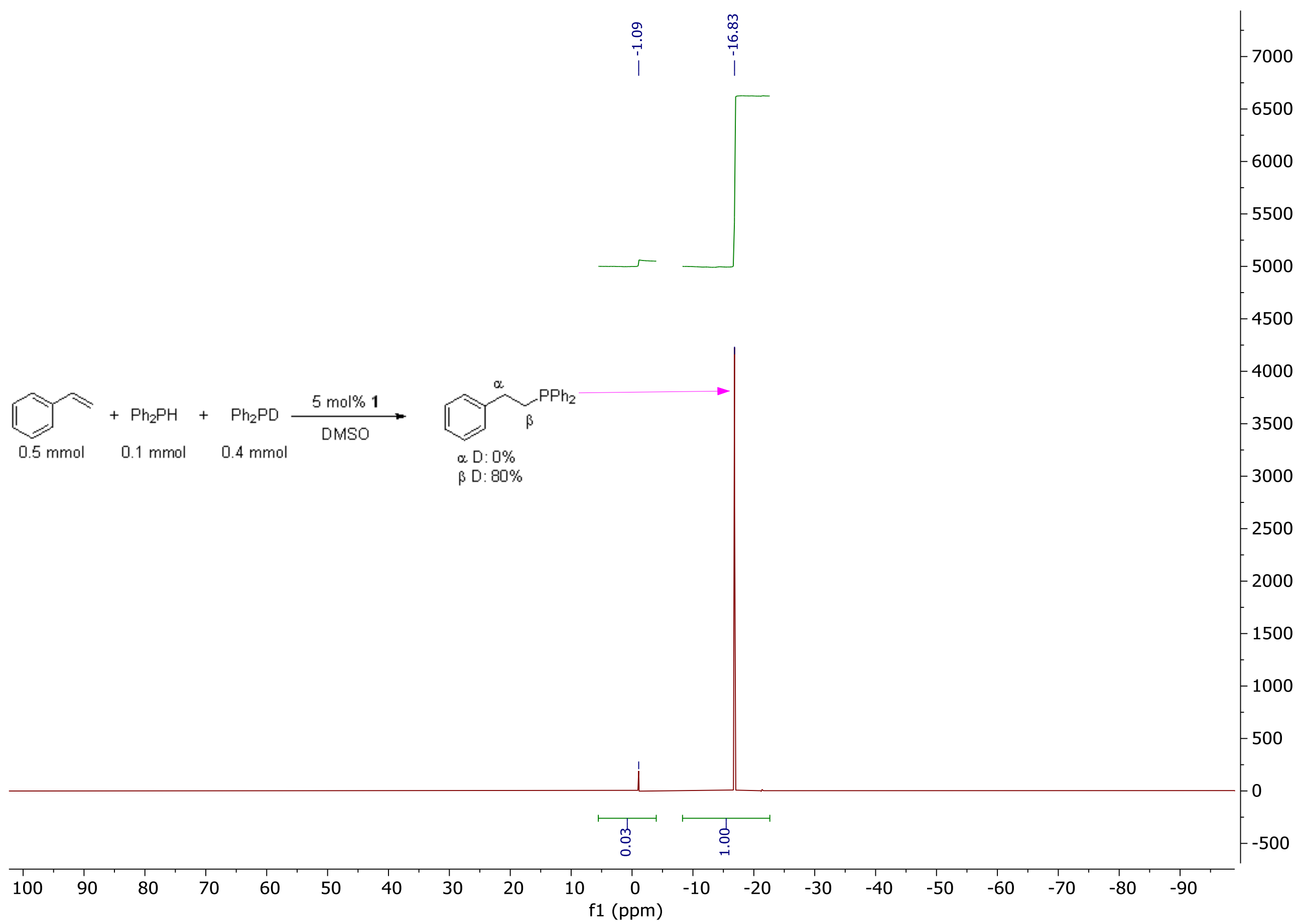

Figure S299: Deuterium Labeling reaction of Ph_2PD, Ph_2PH, styrene in DMSO using catalyst **1** – $^{31}P\{^1H\}$ NMR

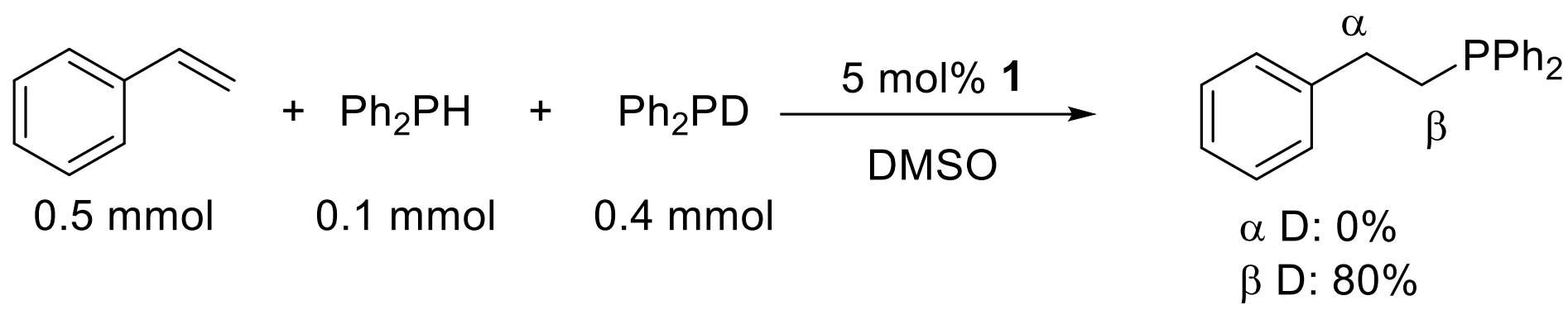

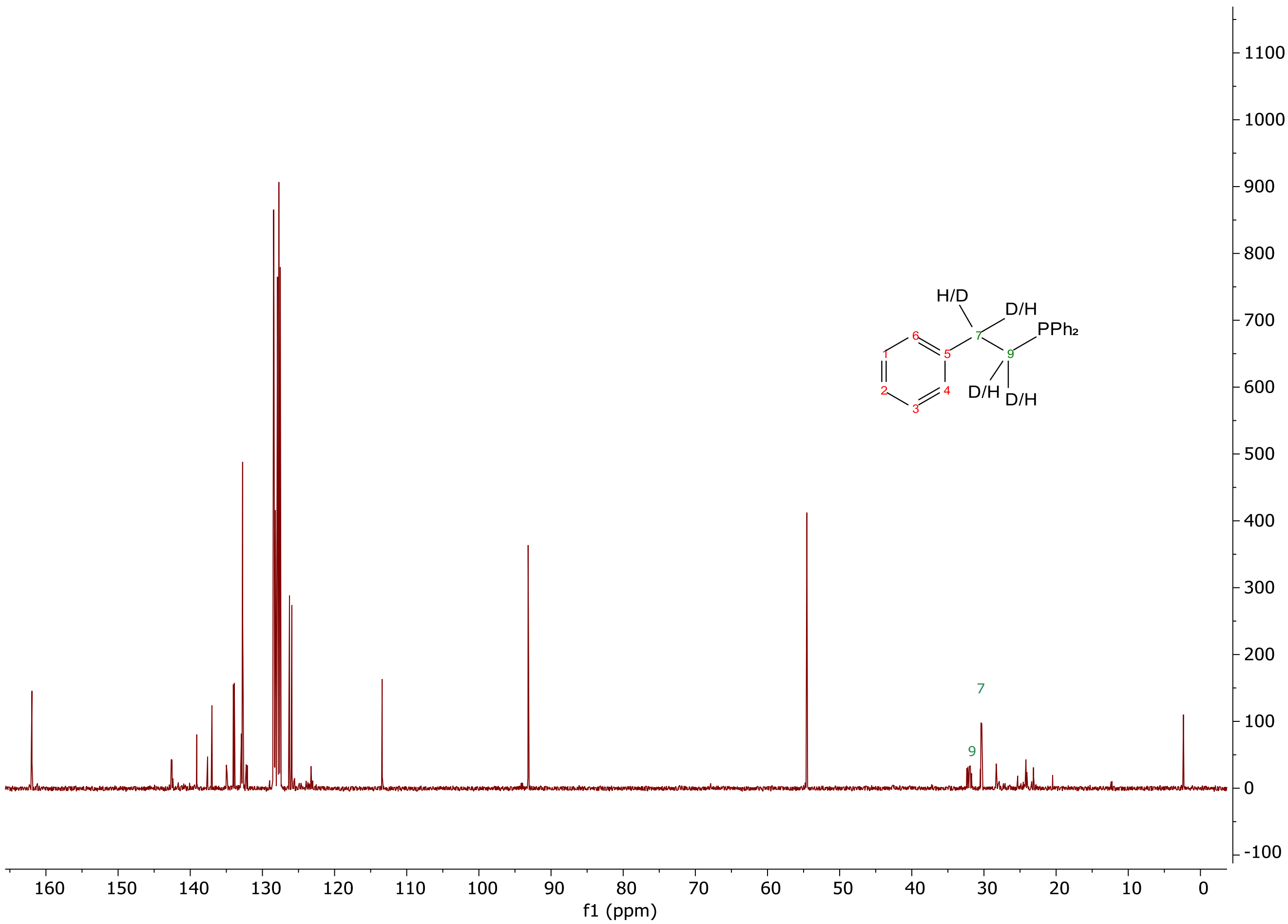

Figure S300: Deuterium Labeling reaction of Ph_2PD, Ph_2PH, styrene in DMSO using catalyst **1** - $^{13}C\{^1H\}$ NMR

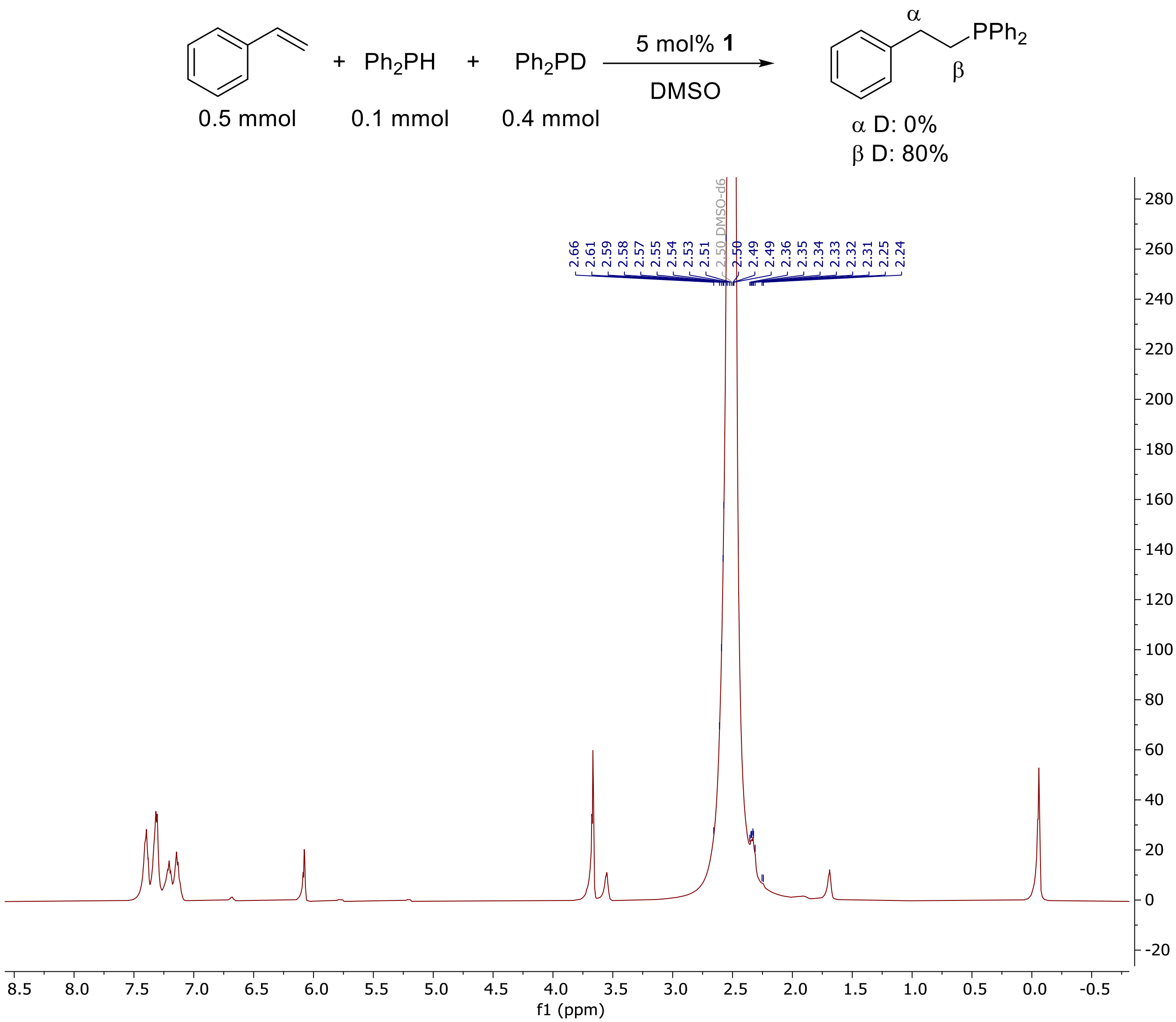

Figure S301: Deuterium Labeling reaction of Ph₂PD, Ph₂PH, styrene in DMSO using catalyst **1** - ^{1}H NMR

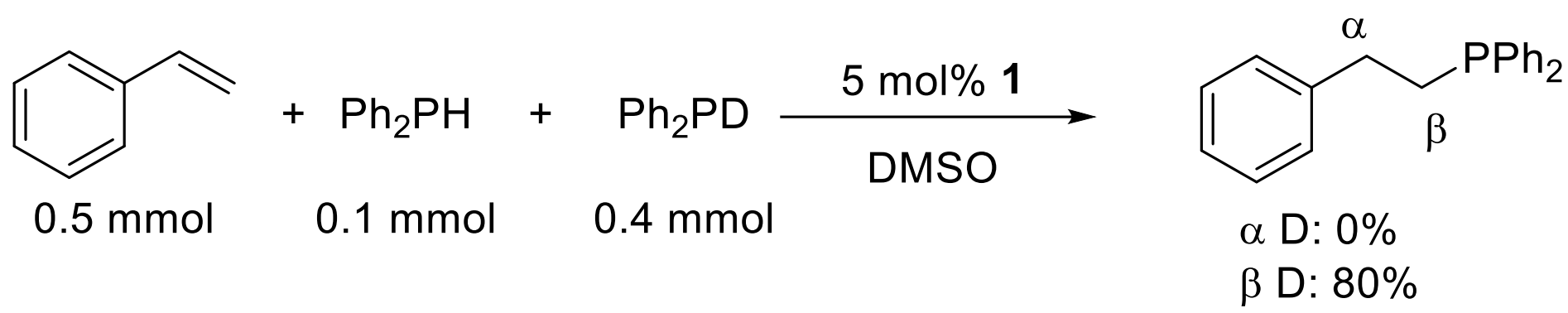

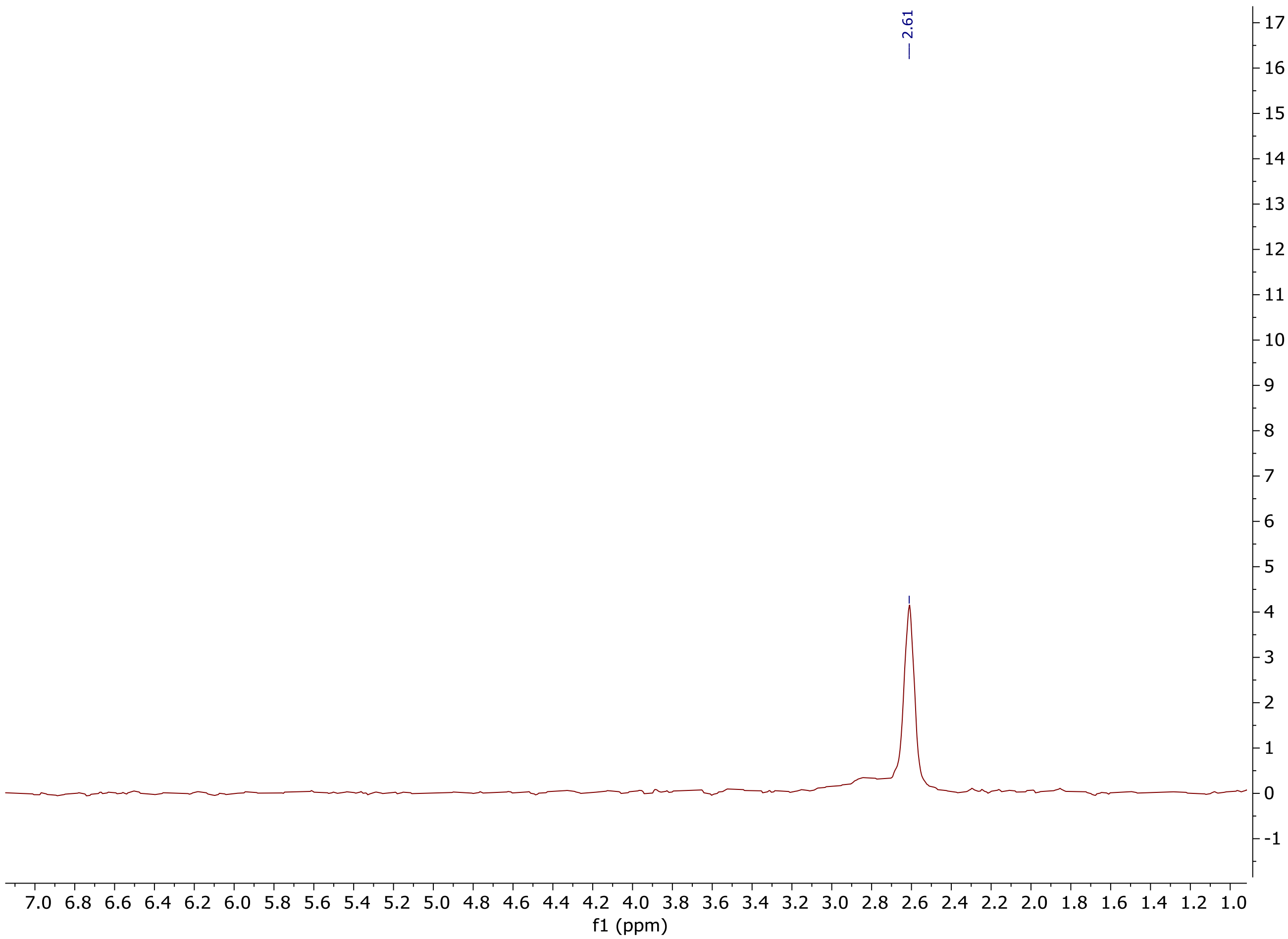

Figure S302: Deuterium Labeling reaction of Ph_2PD, Ph_2PH, styrene in using DMSO catalyst **1** - ^2H NMR

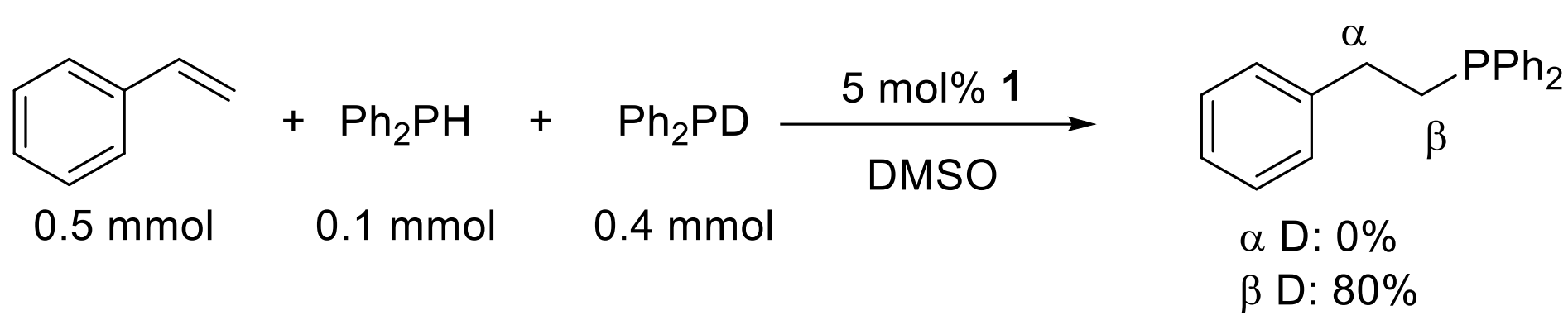

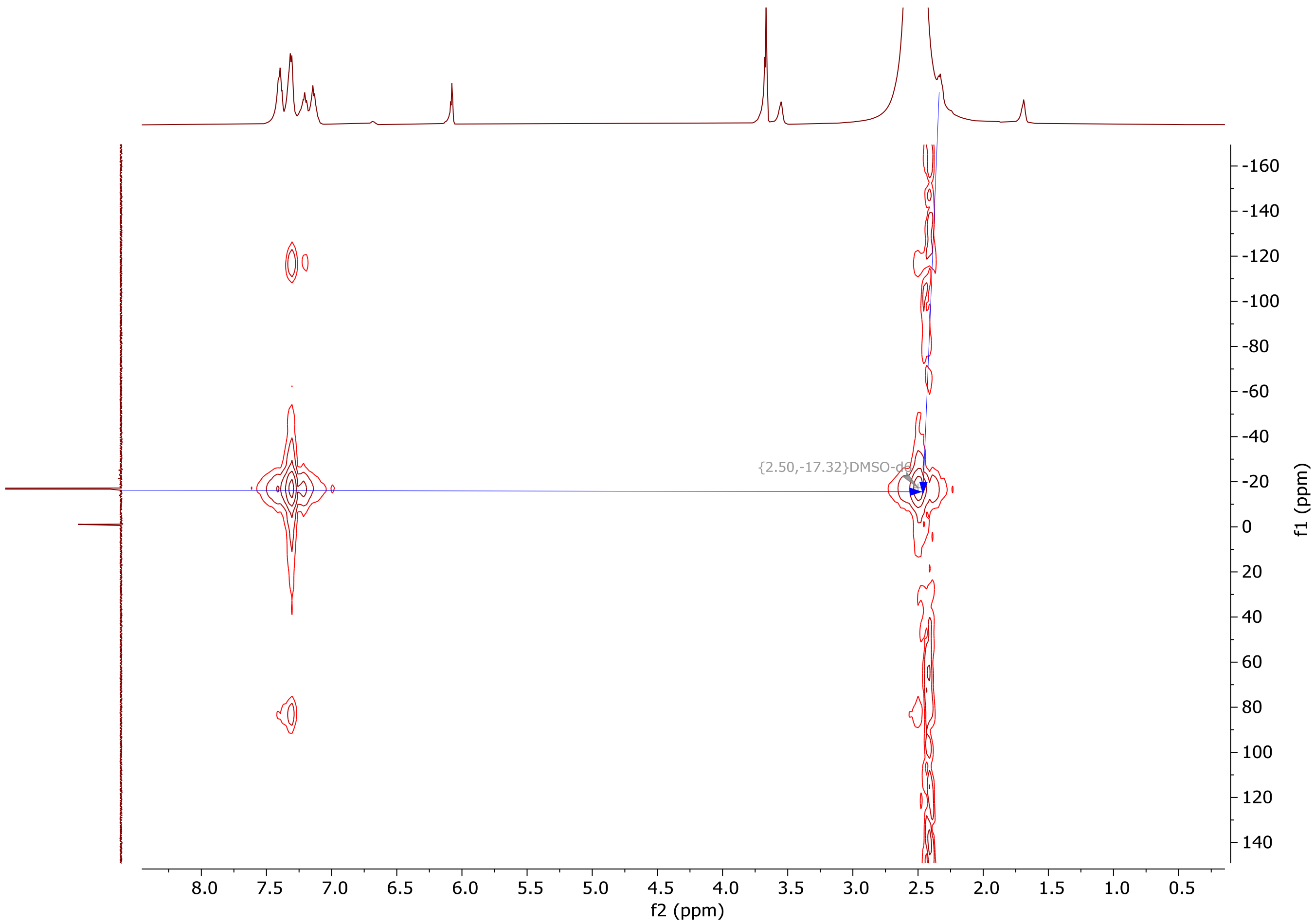

Figure S303: Deuterium Labeling reaction of Ph_2PD, Ph_2PH, styrene in DMSO using catalyst **1** - (^{31}P-^{1}H) HMBC NMR

EPR spectra for only catalyst and hydrophosphination reaction in DMSO at ambient conditions

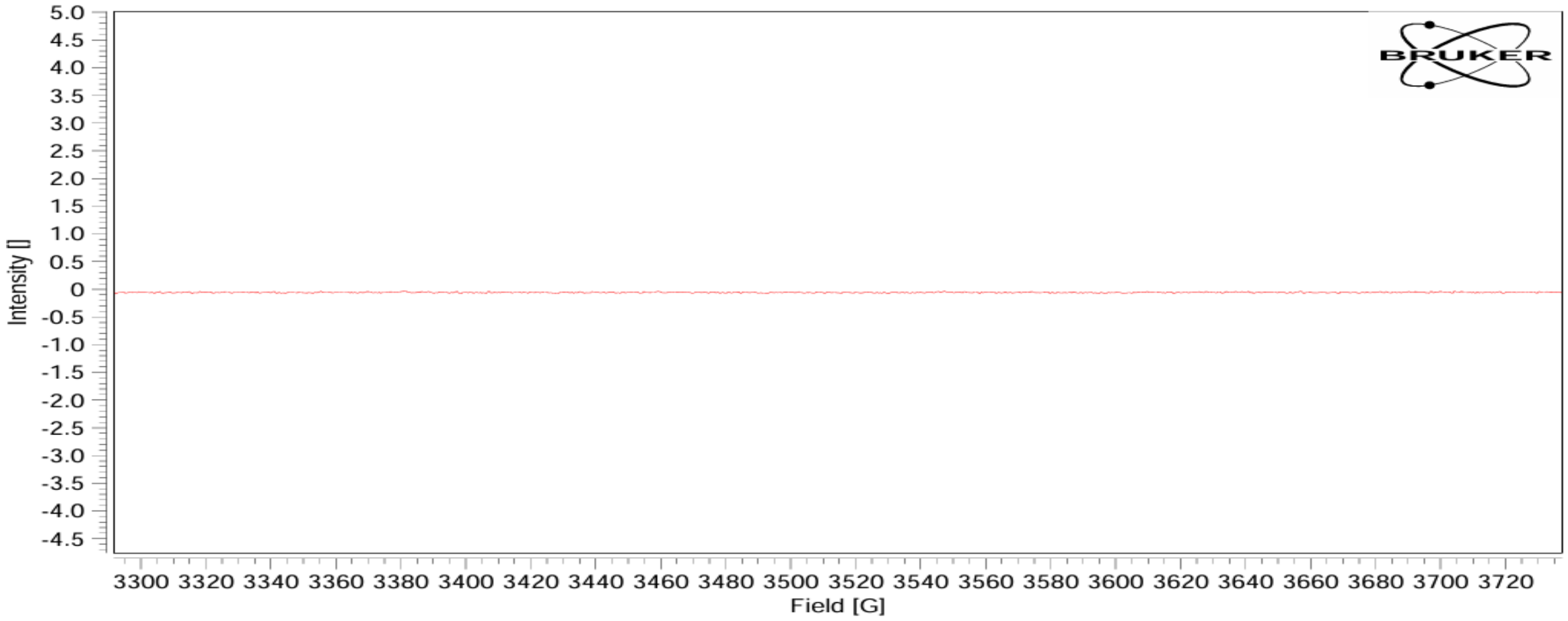

Figure S304: EPR spectra of **1** in DMSO. Microwave frequency: 9.88 G, microwave power: 1.8 mW, modulation amplitude: 1.00 G, modulation frequency: 100.00 KHz, time constant: 0.01 ms, conversion time: 30.0 ms.

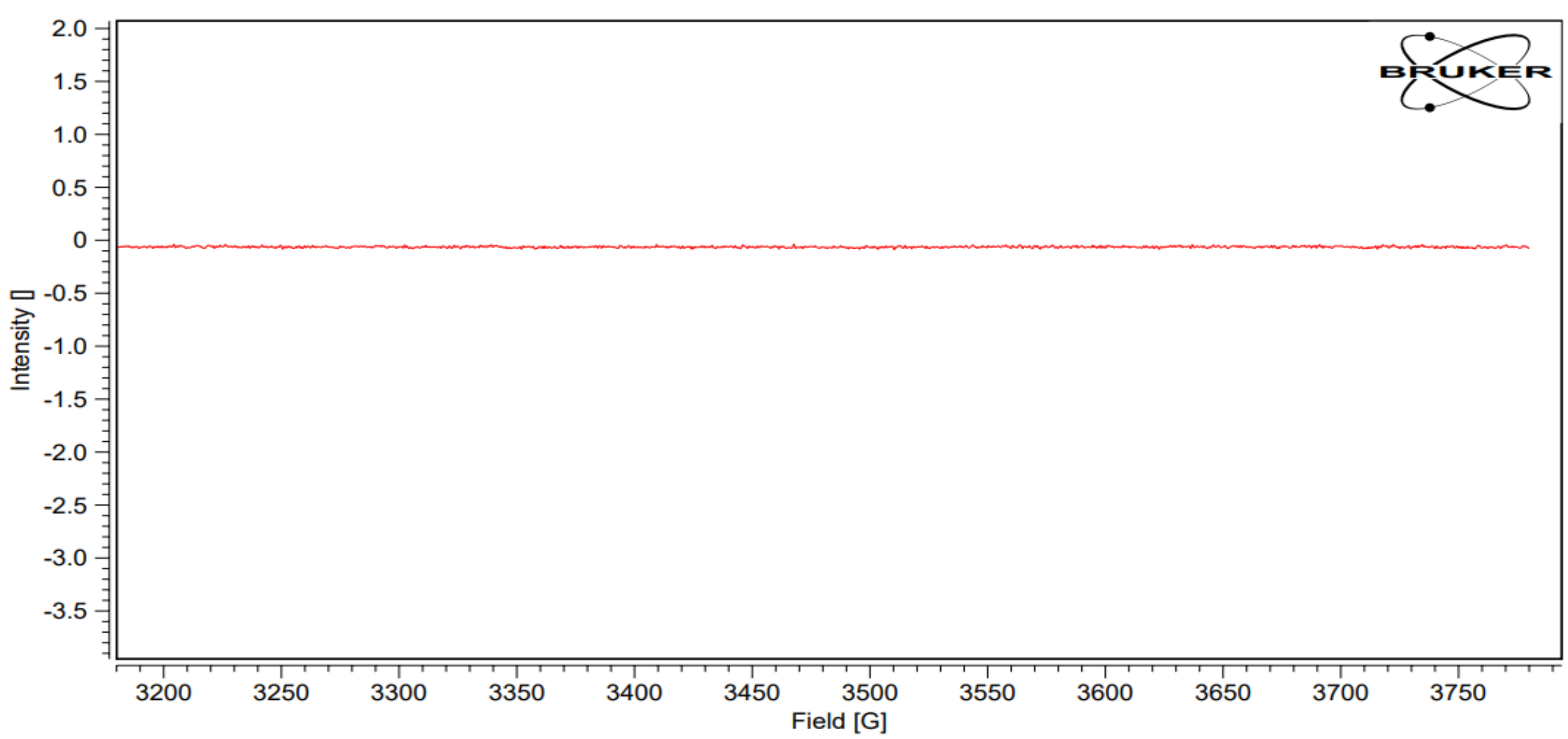

Figure S305: EPR spectra of the hydrophosphination mixture of 0.5 mmol styrene, 0.5 mmol Ph2PH, 5 mol% **1** in DMSO. Microwave frequency: 9.88 G, microwave power: 1.8 mW, modulation amplitude: 1.00 G, modulation frequency: 100.00 KHz,, time constant: 0.01 ms, conversion time: 30.0 ms.

UV-visible spectra for hydrophosphination reaction in DMSO

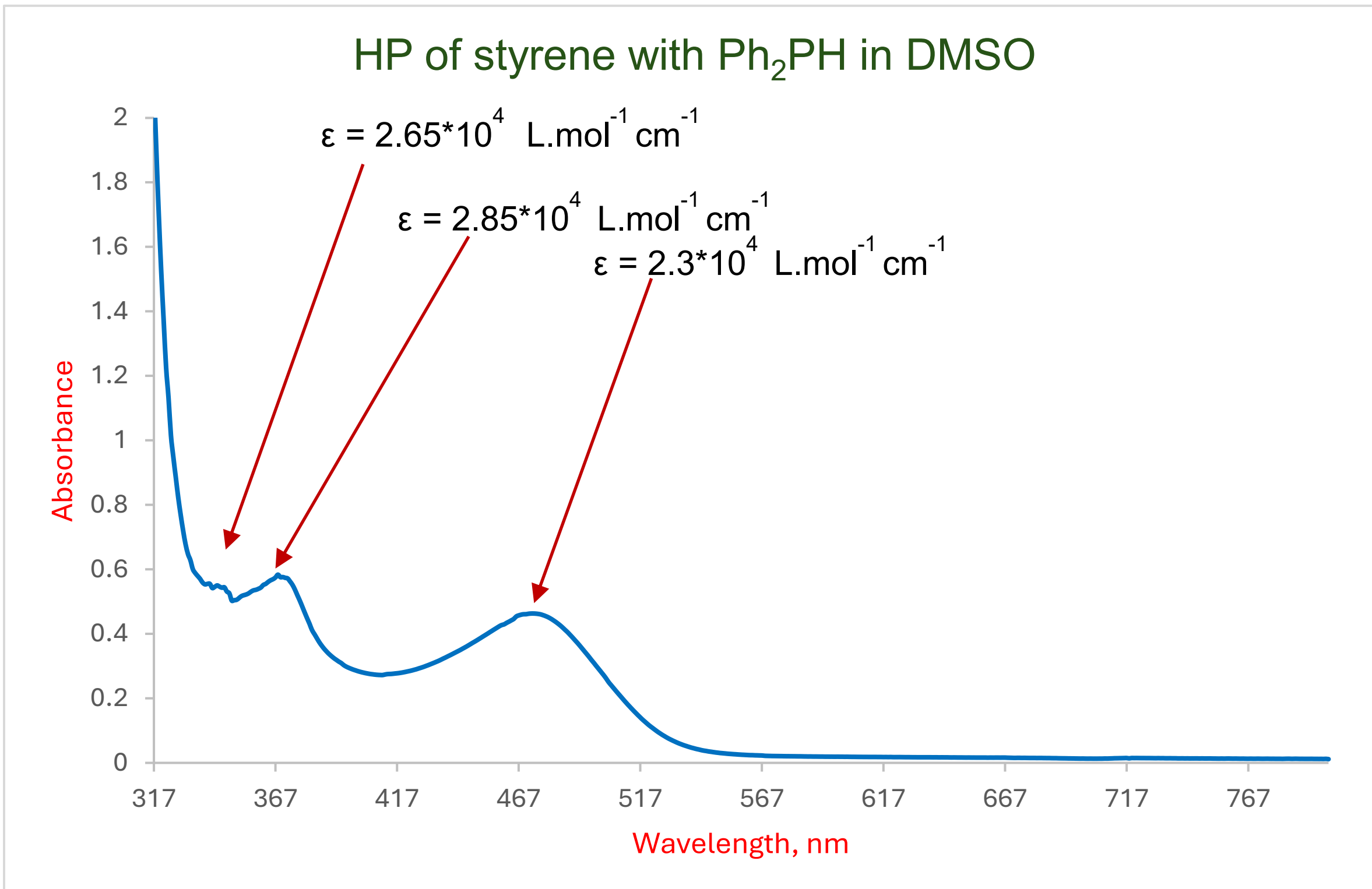

Figure S306: UV-visible spectra of the hydrophosphination reaction mixture of standard reaction using 0.5 mmol of styrene, equimolar Ph_2PH, 5 mol% catalysts **1** in DMSO. The concentration of the mixture is 10^{-4}M.

Hydrophosphination of vinyl arenes using 2 mol % NHCCa(HMDS)$_2$ in DMSO-d_6

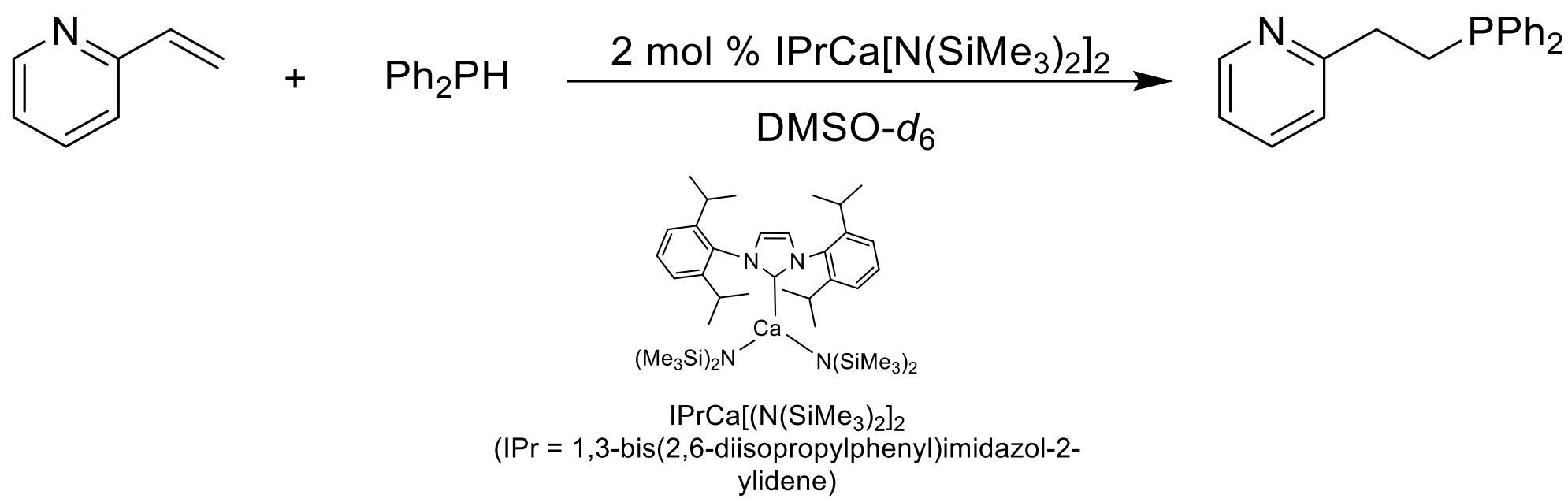

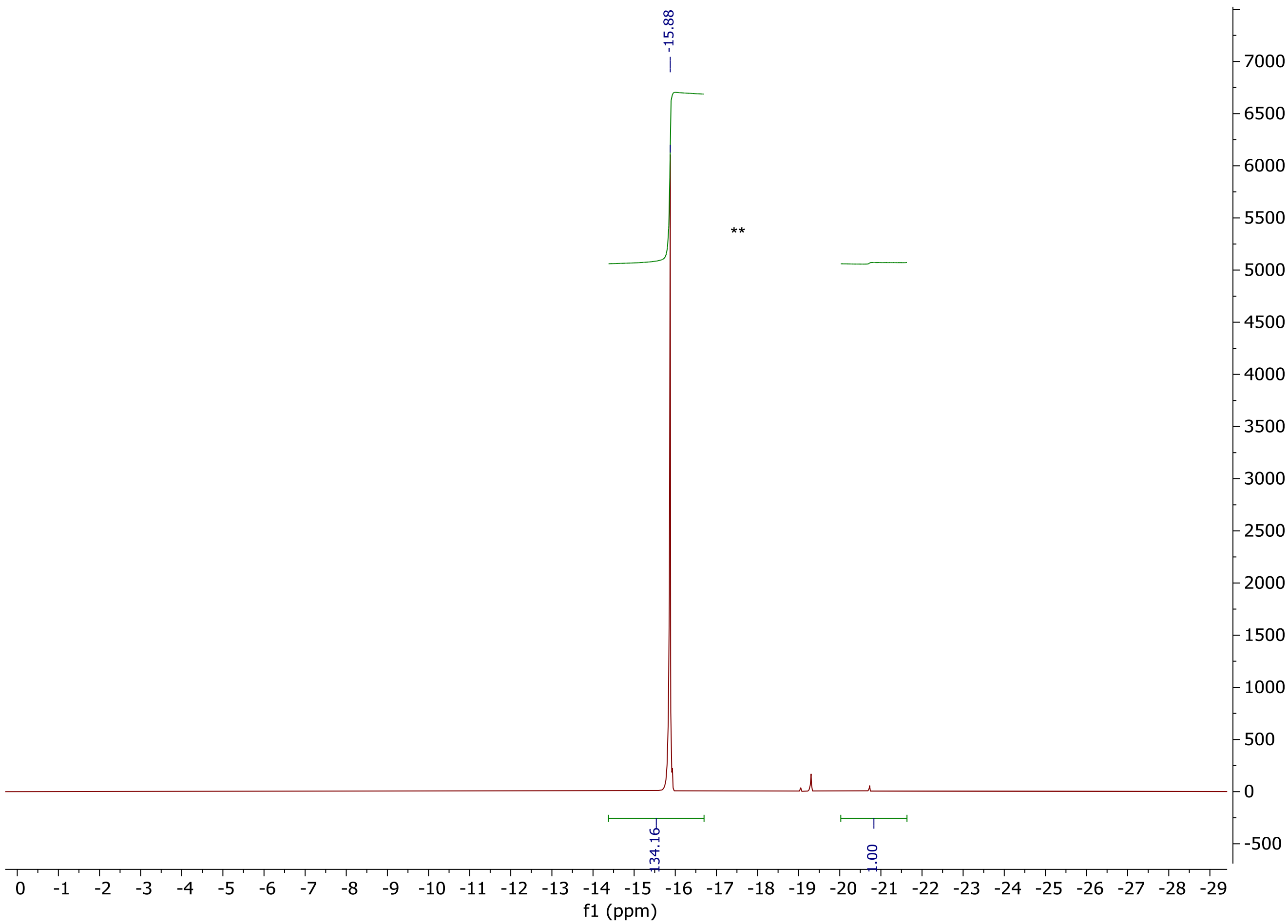

Figure S307: Hydrophosphination of 0.5 mmol 2-vinyl pyridine with equimolar of Ph_2PH in DMSO-d_6 using 2 mol % NHCCa(HMDS)$_2$ at ambient conditions – 0 h - $^{31}P\{^1H\}$ NMR

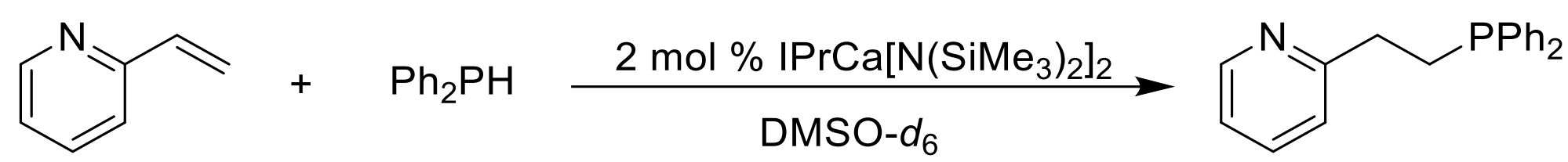

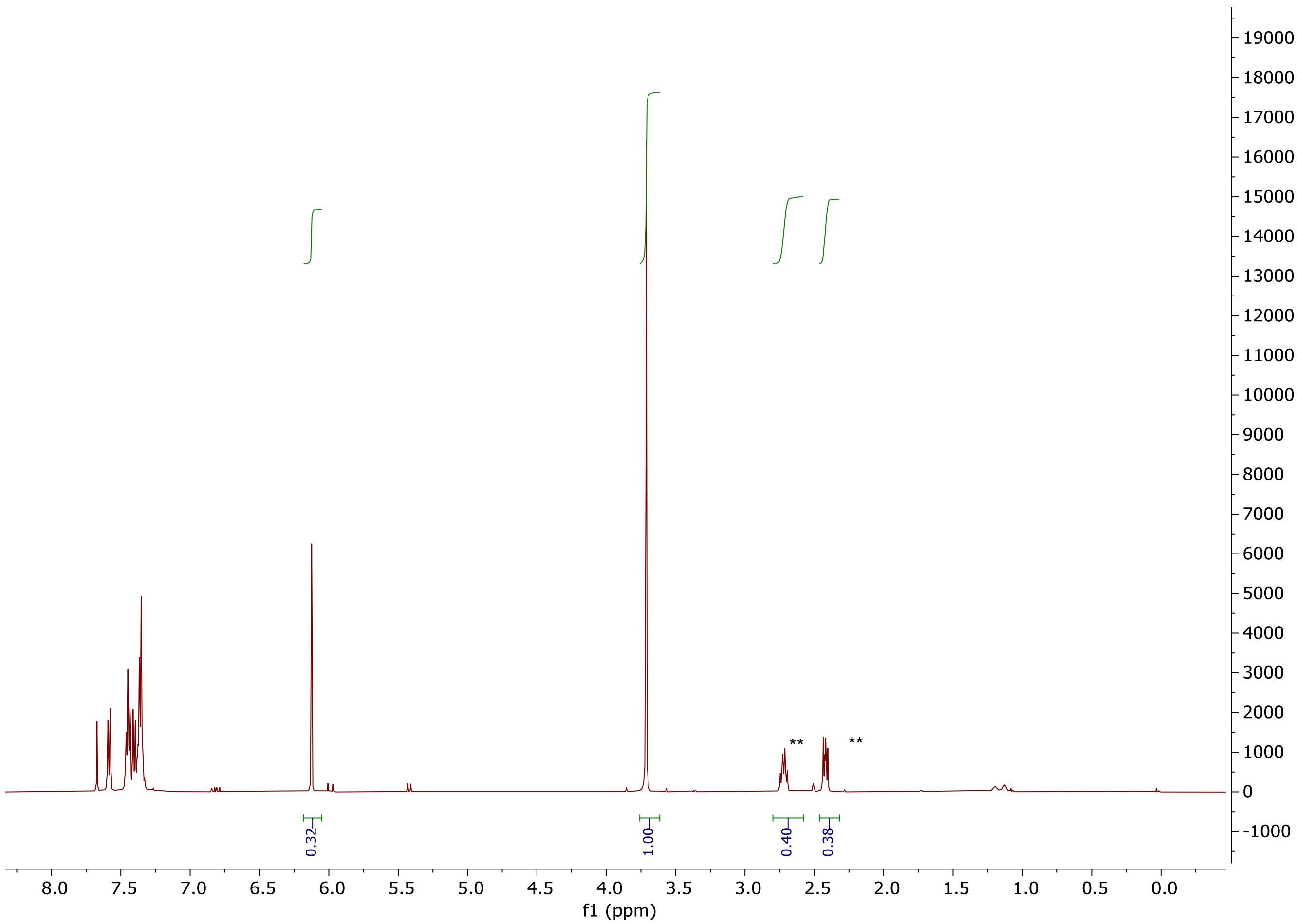

Figure S308: Hydrophosphination of 0.5 mmol 2-vinyl pyridine with equimolar of Ph_2PH in DMSO-d_6 using 2 mol % $NHCCa(HMDS)_2$ under ambient conditions – 0 h - ^{1}H NMR

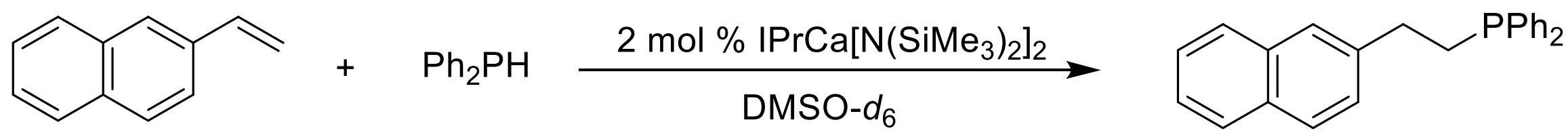

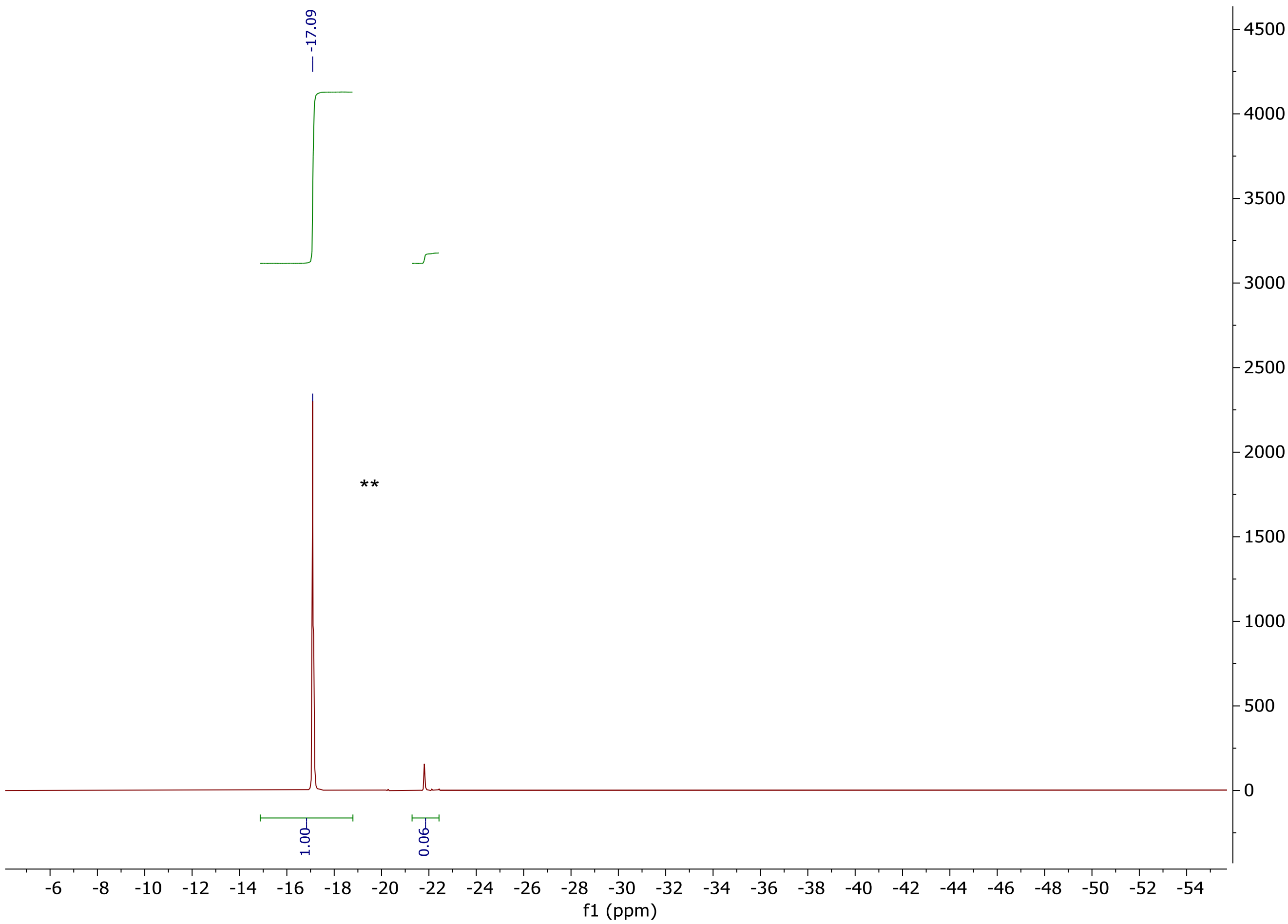

Figure S309: Hydrophosphination of 0.5 mmol 2-vinyl naphthalene with equimolar of Ph_2PH in DMSO-d_6 using 2 mol % NHCCa(HMDS)$_2$ under ambient conditions – 0 h – $^{31}P\{^1H\}$ NMR

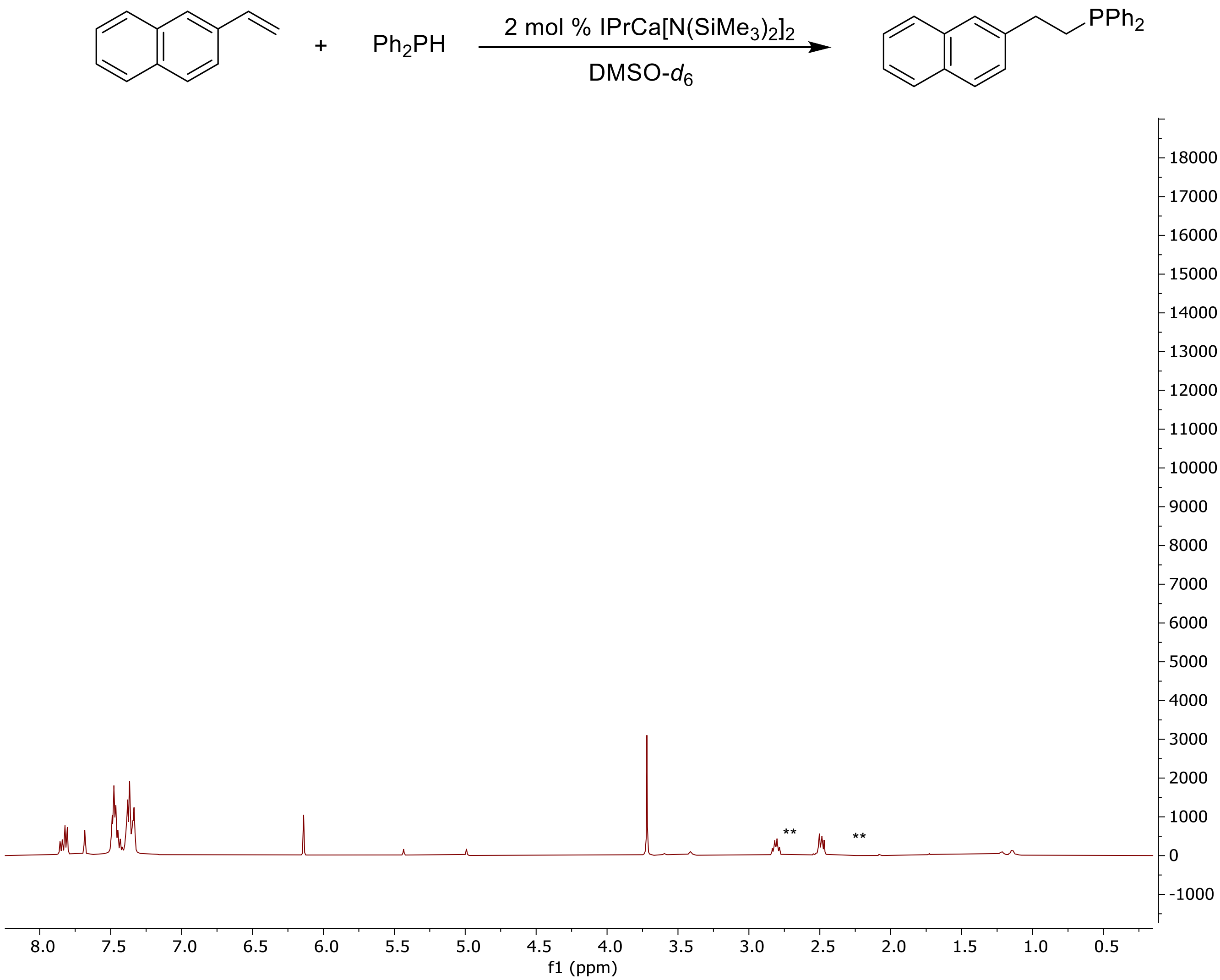

Figure S310: Hydrophosphination of 0.5 mmol 2-vinyl naphthalene with equimolar of Ph_2PH in DMSO-d_6 using 2 mol % $NHCCa(HMDS)_2$ under ambient conditions – 0 h – ^1H NMR

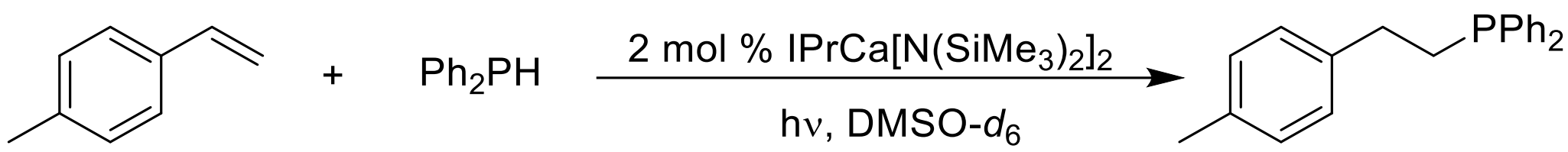

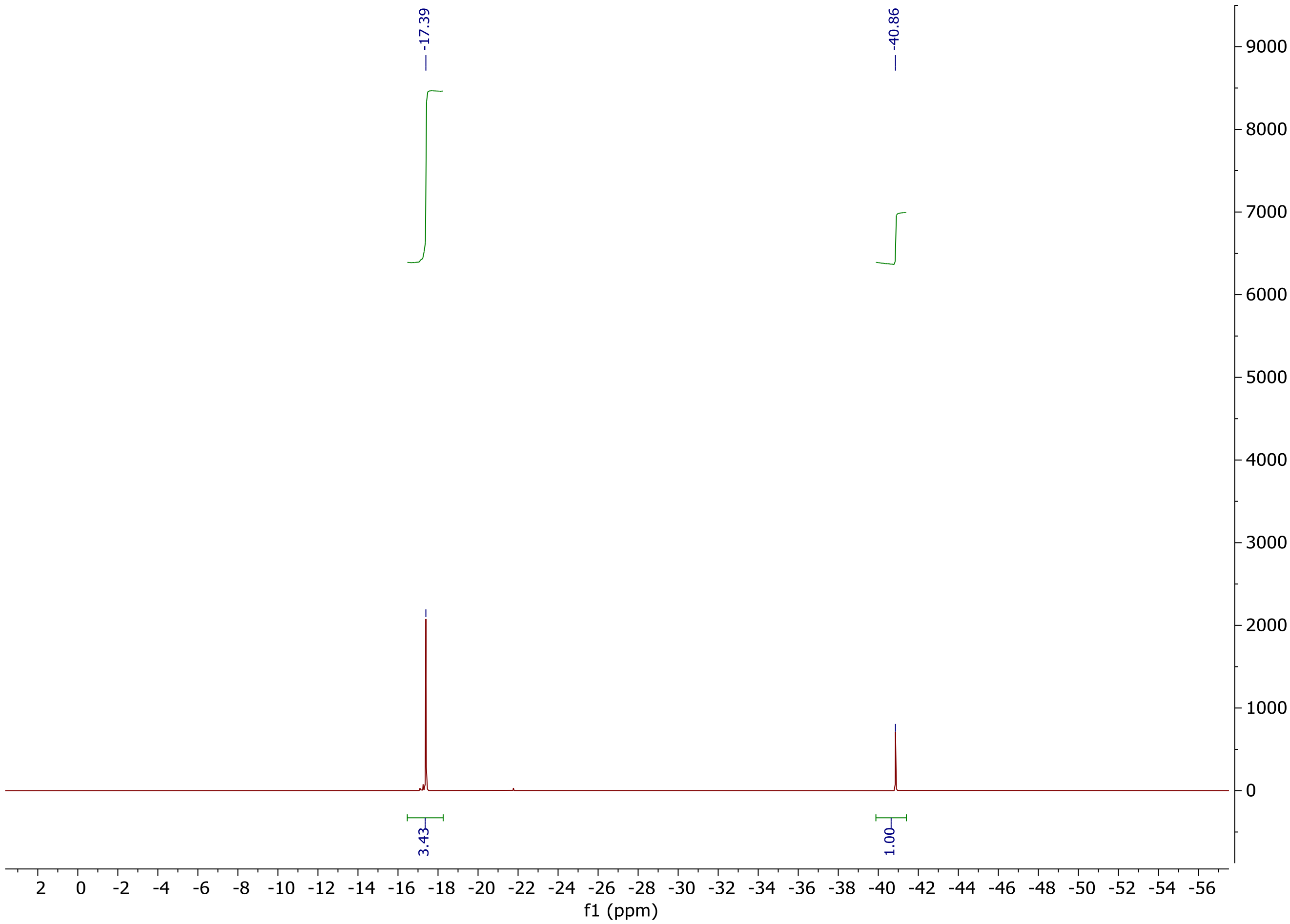

Figure S311: Hydrophosphination of 0.5 mmol 4-methyl styrene with equimolar of Ph_2PH in DMSO-d_6 using 2 mol % NHCCa(HMDS)$_2$ under ambient conditions – 10 h under UV 360 nm irradiation – $^{31}P\{^1H\}$ NMR

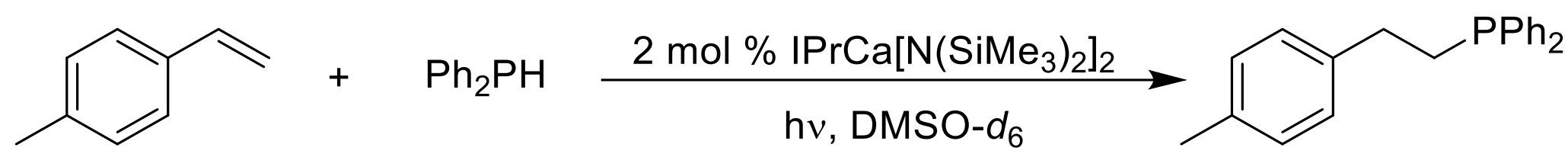

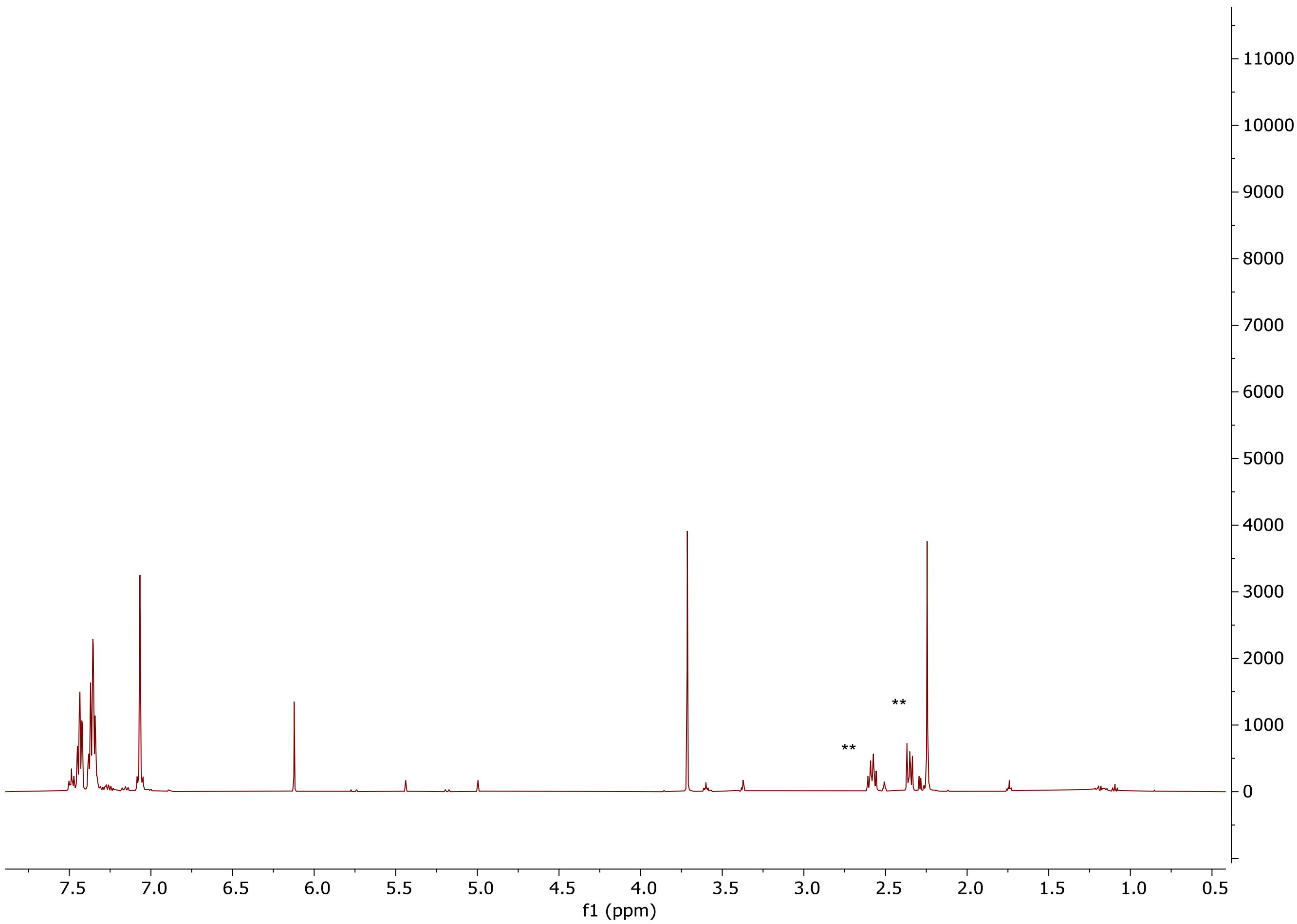

Figure S312: Hydrophosphination of 0.5 mmol 4-methyl styrene with equimolar of Ph_2PH in DMSO-d_6 using 2 mol % NHCCa(HMDS)$_2$ under ambient conditions – 10 h under UV 360 nm – ^{1}H NMR

References:

[1] Bedard, J.; Caschera, A.; Foucher, D. A. RSC Adv. **2021**, 11 (10), 5548-5555, 10.1039/D1RA00555C. DOI: 10.1039/D1RA00555C.

[2] C. R. Woof, D. J. Durand, N. Fey, E. Richards, R. L. Webster, *Chem. Eur. J.* **2021**, *27*, 5972–5977.

[3] M. R. Crimmin, I. J. Casely, M. S. Hill, *J. Am. Chem. Soc.* **2005**, *127*, 2042–2043.

[4] Dannenberg, S. G.; Seth, D. M., Jr.; Finfer, E. J.; Waterman, R. ACS Catal. **2023**, 13 (1), 550-562. DOI: 10.1021/acscatal.2c05221.
